# Expansion of the quality of care index on breast cancer and its risk factors using the global burden of disease study 2019

**DOI:** 10.1002/cam4.4951

**Published:** 2022-06-30

**Authors:** Sina Azadnajafabad, Sahar Saeedi Moghaddam, Mohammad Keykhaei, Parnian Shobeiri, Negar Rezaei, Erfan Ghasemi, Esmaeil Mohammadi, Naser Ahmadi, Azin Ghamari, Sarvenaz Shahin, Nazila Rezaei, Mahdi Aghili, Ahmad Kaviani, Bagher Larijani, Farshad Farzadfar

**Affiliations:** ^1^ Non‐Communicable Diseases Research Center, Endocrinology and Metabolism Population Sciences Institute Tehran University of Medical Sciences Tehran Iran; ^2^ Breast Disease Research Center Tehran University of Medical Sciences Tehran Iran; ^3^ Department of Surgery Tehran University of Medical Sciences Tehran Iran; ^4^ Feinberg Cardiovascular and Renal Research Institute Northwestern University School of Medicine Chicago USA; ^5^ Endocrinology and Metabolism Research Center, Endocrinology and Metabolism Clinical Sciences Institute Tehran University of Medical Sciences Tehran Iran; ^6^ Radiation Oncology Research Center Tehran University of Medical Sciences Tehran Iran; ^7^ Department of Surgical Oncology University of Montreal Montreal Quebec Canada

**Keywords:** breast cancer, global burden of disease, health care quality, quality of care index, risk factors

## Abstract

**Background:**

Breast cancer (BC), as the top neoplasm in prevalence and mortality in females, imposes a heavy burden on health systems. Evaluation of quality of care and management of patients with BC and its responsible risk factors was the aim of this study.

**Methods:**

We retrieved epidemiologic data of BC from the Global Burden of Disease (GBD) 1990–2019 database. Epidemiology and burden of BC and its risk factors were explored besides the Quality of Care Index (QCI) introduced before, to assess the provided care for patients with BC in various scales. Provided care for BC risk factors was investigated by their impact on years of life lost and years lived with disability by a novel risk factor quality index (rQCI). We used the socio‐demographic index (SDI) to compare results in different socio‐economic levels.

**Results:**

In 2019, 1,977,212 (95% UI: 1,807,615–2,145,215) new cases of BC in females and 25,143 (22,231–27,786) in males was diagnosed and this major cancer caused 688,562 (635,323–739,571) deaths in females and 12,098 (10,693–13,322) deaths in males, globally. The all‐age number of deaths and disability‐adjusted life years attributed to BC risk factors in females had an increasing pattern, with a more prominent pattern in metabolic risks. The global estimated age‐standardized QCI for BC in females in 2019 was 78.7. The estimated QCI was highest in high SDI regions (95.7). The top countries with the highest calculated QCI in 2019 were Iceland (100), Japan (99.8), and Finland (98.8), and the bottom countries were Mozambique (16.0), Somalia (8.2), and Central African Republic (5.3). The global estimated age‐standardized rQCI for females was 82.2 in 2019.

**Conclusion:**

In spite of the partially restrained burden of BC in recent years, the attributable burden to risk factors has increased remarkably. Countries with higher SDI provided better care regarding both the condition and its responsible risk factors.

## Graphical abstract

Breast cancer (BC), as the top neoplasm in prevalence and mortality in females requires evaluation of the quality of care and burden estimates. Using the latest Global Burden of Disease dataset, we provided the updated global, regional, and national picture of BC burden and quality of care for the disease and its responsible risk factors

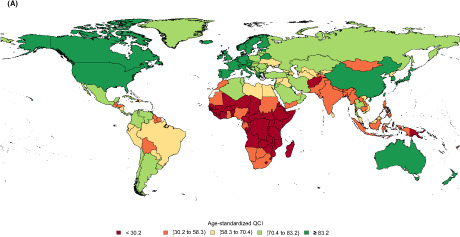

## 1 INTRODUCTION

Breast cancer (BC) is the most common diagnosed cancer and cause of death due to cancers in women globally.^1^, ^2^ Various regions and countries have experienced different patterns of BC epidemiology; however, this cancer and the imposed burden remain the major public health concern among the female population.^3^, ^4^ BC occurrence is proved to be linked with two groups of inherent risk factors—including age, sex, ethnicity, genetic, and other intrinsic factors—which are not simply modifiable, and extrinsic factors—including lifestyle, diet, metabolic, and hormonal therapies—which are modifiable and could be altered by interventions.^2^, ^5^ Since the exposure to major extrinsic BC risk factors is growing, the significant share of the attributable burden to the risk factors is one of the major obstacles in the management of the BC pandemic.^6^ Also, BC in males as a distinct cause of cancer, had different epidemiological patterns and need more investigation.^7^, ^8^

Besides the importance of the general concept of quality of care in providing healthcare services to patients, ensuring the quality of cancer care plays a major role in the management of patients diagnosed with various cancers to reach the desired outcomes.^9^ Due to various clinical and socio‐economic underlying causes, the quality of cancer care has faced many gaps and has yet to be improved in some regions and countries worldwide, especially in developing countries and those with limited resources, leading to delayed presentation and diagnosis of this cancer.^10^, ^11^, ^12^, ^13^ In this regard, BC patients, as a major group of patients with cancers, showed to be vulnerable in perceived quality of care and exposed to poor quality in terms of too much unnecessary care, too little care, or the wrong received care and with considerable disparities in some areas.^14^, ^15^, ^16^ Besides, variations in different aspects of BC care exist, including screening, diagnosis, treatment, and follow‐up stages of the disease management.^17^ Therefore, providing evidence‐based information on BC quality of care, especially through large‐scale population‐based studies, could help BC care.

To provide an insightful vision on the global, regional, and national burden and quality of care of BC and its major risk factors, we aimed to conduct this study using the recent Global Burden of Disease (GBD) 2019 data and the developed measures for quality of care assessment. The beneficial results of this study potentially provide health authorities and clinicians worldwide with the essential data on how to re‐allocate resources to curb the heavy burden of this cancer through appropriate measures.

## 2 MATERIALS AND METHODS

### 2.1 Data source

To evaluate the epidemiologic pattern and quality of care of BC we utilized the Global Burden of Disease (GBD) data from 1990 to 2019, prepared by the Institute for Health Metrics and Evaluation and available through an online tool (http://ghdx.healthdata.org/gbd‐results‐tool).^18^ In the latest update of GBD 2019, data of global, regional, and national burden of 369 diseases and injuries and 87 risk factors in 204 countries and territories are provided. Detailed methods of GBD estimations on the burden of diseases and risk factors during 1990–2019 are provided in previous publications.^19^, ^20^ We investigated features of the specific cause of BC in this study coded as B.1.14 in the hierarchal classification of causes on GBD. GBD maps International Classification of Diseases and Injuries‐10 (ICD‐10) codes C50‐C50.9, D05‐D05.9, D24‐D24.9, D48.6, D49.3, and ICD‐9 codes 174–175.9, 217–217.8, 233.0, 238.3, 239.3, 610–610.9 to this specific GBD cause as BC mortality data and ICD‐10 codes C50‐C50.629, C50.8‐C50.929, Z12.3‐Z12.39, Z80.3, Z85.3, Z86.000, and ICD‐9 codes 174–175.9, V10.3, V16.3 to BC incidence data, to include available epidemiologic data on BC all around the world.^19^, ^21^, ^22^ This study is designed and conducted according to the Guidelines for Accurate and Transparent Health Estimates Reporting (GATHER) statement.^23^

### 2.2 Study variables

A set of epidemiologic variables including incidence, prevalence, deaths, years of life lost (YLLs), years lived with disability (YLDs), and disability‐adjusted life years (DALYs) of BC were explored to investigate epidemiology and burden of BC in global, regional, and national levels. Various risk factors for BC in females approved by Institute for Health Metrics and Evaluation were included in two main categories of behavioral risk factors including alcohol use, diet high in red meat, low physical activity, smoking, and secondhand smoke, and metabolic risk factors comprising high body‐mass index (BMI) and high fasting plasma glucose (FPG).^24^ Attributed deaths, YLLs, YLDs, and DALYs were explored for the mentioned BC risk factors in this study. Besides Quality of care index (QCI) for BC and responsible risk factors are presented in this study using the methods explained later. We used the socio‐demographic index (SDI) to compare results in different socio‐economic levels, which is a composite indicator of income per capita, average education in years of schooling, and total fertility rate in women aged under 25 years old.^25^ Global, national, and six World Health Organization (WHO) regions, including the European region, the region of the Americas, the Western Pacific region, the Eastern Mediterranean region, the South‐East Asia region, and the African region, were explored as various geographical scales in this study.

### 2.3 Quality of care index

To assess the quality of care parameters in BC management, we used the Quality of Care Index (QCI) developed and implemented before to investigate the quality of care of various diseases and conditions. Calculation details and codes of QCI are available in a published protocol.^26^ Also, other published articles utilizing the QCI to evaluate quality of care are available to prove the efficacy of this proxy.^27^, ^28^, ^29^, ^30^, ^31^, ^32^, ^33^, ^34^, ^35^, ^36^, ^37^, ^38^ In summary, QCI is generated from the integration of four indices of mortality to incidence ratio (MIR), DALYs to prevalence ratio, prevalence to incidence ratio, and YLLs to YLDs ratio compiled by the principal component analysis (PCA) method.^39^ The QCI scores are scaled into 0 to 100, as higher scores indicate better quality of care. Validation of QCI in BC was made by comparing it to the healthcare access and quality index (HAQI) as a confirmed tool for assessing the quality of care in various diseases.^40^ In this order, we designed a linear mixed effect model including QCI of BC as a dependent variable and inpatient and outpatient healthcare utilization,^41^ risk factor exposure, mortality, and prevalence of BC as independent variables, and the estimated correlation with HAQI was 0.90, indicating the efficacy of this index in evaluation of BC care.

In order to investigate the quality of care of responsible risk factors of BC in GBD, as a tool to highlight the importance of modifiable risk factors of this cancer with the final goal of promoting a healthier lifestyle, we chose one of four components used in the generation of QCI, YLLs/YLDs ratio. This useful ratio recruits the age‐standardized rates of YLLs and YLDs attributable to each known risk factor of BC and lower values of this ratio indicate a better overall care of each risk, since postponing death due to a specific risk of a cause and extending patients' lives even with years living with disability, represents better health coverage and quality of care provided by health systems. To accumulate care for risk factors, we generated one unique index by recruiting PCA method on the ratios of seven risks in different scales, named rQCI (risk factor QCI) in this study. Also, to make rQCI reflect the better management of the BC risk factors, we subtracted the rescaled calculated values from 100, to reversely scale this measure. Therefore, the higher values of rQCI indicate a better overall care for risk factors responsible for BC in terms of lowering YLLs compared to YLDs and lower rQCI values represent a worse risk factor management regarding BC. Due to multiple deficiencies in risk factors data for the male population with BC, this index was only calculated for females in this study. Also, due to the lack of risk factor data for Somalia, the calculation of rQCI for this country was not possible.

(1) $$\begin{array}{c} \begin{array}{c} \;\mathrm{BC}\;\text{rQCI}=100-(\mathrm{PCA}\;[\;\frac{\mathrm{YLL}}{\mathrm{YLD}}\;\left(\text{high}\;\mathrm{FPG}\right),\;\frac{\mathrm{YLL}}{\mathrm{YLD}}\;\left(\text{high}\;\mathrm{BMI}\right),\;\\ \frac{\mathrm{YLL}}{\mathrm{YLD}}\;\left(\text{smoking}\right),\;\frac{\mathrm{YLL}}{\mathrm{YLD}}\;\left(\text{secondhand smoke}\right),\;\\ \frac{\mathrm{YLL}}{\mathrm{YLD}}\;\left(\mathrm{low}\;\text{physical activity}\right),\;\\ \frac{\mathrm{YLL}}{\mathrm{YLD}}\;\left(\text{alcohol}\;\mathrm{use}\right),\;\frac{\mathrm{YLL}}{\mathrm{YLD}}\;\left(\text{diet high in}\;\mathrm{red}\;\text{meat}\right)]). \end{array} \end{array}$$

### 2.4 Statistical analysis

Epidemiologic indices values were reported with a 95% uncertainty interval (UI) in all‐age numbers and age‐standardized rates per 100,000 population. Age was classified into three categories of 15–49, 50–74, and 75 plus, based on the clinical significance. Percent changes for each measure were generated by dividing the subtracted value of the first year (1990) from the last year (2019) to the value of the first year. Bivariate correlation using the Pearson correlation coefficient was used to assess the correlation between QCI and rQCI values, with 0.05 as the statistical significance level for the test. Results were investigated by sex (male, female), as compiling results of both sexes in the evaluation of BC would be misleading. All the statistical analyses and visualizations in this study were performed by R for windows v4.0.3 (http://www.r‐project.org/, RRID: SCR_001905).^42^

## 3 RESULTS

### 3.1 Epidemiology of BC

In 2019, the global all‐ages new cases of BC were 1,977,212 (95% UI: 1,807,615–2,145,215) for females and 25,143 (22,231–27,786) for males with a 57% (43.9–70.3) and 86.2% (60.2–110) increase compared to 1990, respectively. This cancer caused 688,562 (635,323–739,571) deaths in females and 12,098 (10,693–13,322) deaths in males in 2019, with a 26.5% (16.8–35.6) and 42.6% (21.5–62.2) increase respectively, in the study period. The global DALYs due to BC were 20,310,187 (18,744,799–21,866,646) in females and 315,126 (278,546–349,292) in males in 2019, with a 21.4% (11.5–30.5) and 41.7% (20.2–61.7) increase, respectively. Investigating the age‐standardized rates by sex revealed that incidence rate of BC in females increased 14.3% (4.7–24) during the study period. Also, the age‐standardized incidence rate in males increased more prominently by 22.0% (5.1–37.2). The age‐standardized mortality rates of BC in females decreased by 10.5% (−17.3 to −4.2) in the last three decades, and this rate had a similar reduction by 10.6% (−23.5 to 1.8) in males. The age‐standardized rates of DALYs to BC in females decreased by 9.7% (−17.1 to −3) in the study period, and with a slight decrease in DALY rates in males by 2.7% (−17.1 to 10.5) (Table 1; Table S1).

**TABLE 1:** The global trend of epidemiologic indices of breast cancer, all‐age numbers and age‐standardized rates, for each sex, in 1990 and 2019 and percent of changes in the 1990–2019 period

Measure	Year	1990		2019		1990–2019 percent change (%)	
	Metric	Female	Male	Female	Male	Female	Male
Incidence	Number	867,621 (840,400 to 894,764)^a^	9372 (8809 to 9965)	1,977,212 (1,807,615 to 2,145,215)	25,143 (22,231 to 27,786)	57 (43.9 to 70.3)	86.2 (60.2 to 110)
	Rate	40.1 (38.8 to 41.3)	0.5 (0.5 to 0.6)	45.9 (41.9 to 49.8)	0.7 (0.6 to 0.7)	14.3 (4.7 to 24)	22 (5.1 to 37.2)
Prevalence	Number	8,530,273 (7,930,451 to 9,258,183)	70,400 (64,878 to 76,734)	19,084,906 (17,547,052 to 20,568,942)	198,795 (177,781 to 218,546)	54.1 (43 to 66.1)	96 (73.8 to 117.5)
	Rate	397.8 (369.3 to 433.5)	3.9 (3.6 to 4.3)	441.5 (406.2 to 475.9)	5.1 (4.6 to 5.7)	11 (2.6 to 19.8)	30.9 (16.9 to 45)
Deaths	Number	375,016 (358,978 to 390,820)	5889 (5425 to 6371)	688,562 (635,323 to 739,571)	12,098 (10,693 to 13,322)	26.5 (16.8 to 35.6)	42.6 (21.5 to 62.2)
	Rate	17.8 (16.9 to 18.5)	0.4 (0.3 to 0.4)	15.9 (14.7 to 17.1)	0.3 (0.3 to 0.4)	−10.5 (−17.3 to −4.2)	−10.6 (−23.5 to 1.8)
YLLs (years of life lost)	Number	10,919,861 (10,484,224 to 11,440,520)	147,797 (136,607 to 160,159)	18,943,447 (17,533,330 to 20,455,079)	296,598 (261,481 to 328,528)	19.5 (9.4 to 29)	39.3 (17.8 to 59.5)
	Rate	496.8 (476.9 to 520.2)	7.8 (7.2 to 8.4)	442.1 (409 to 477.5)	7.5 (6.6 to 8.3)	−11 (−18.6 to −3.9)	−4.3 (−18.8 to 9.3)
YLDs (years lived with disability)	Number	606,822 (427,202 to 821,210)	6578 (4629 to 8752)	1,366,740 (956,851 to 1,845,097)	18,527 (13,227 to 25,061)	55.1 (43.5 to 68.1)	95.5 (70.8 to 118.7)
	Rate	28.1 (19.8 to 37.9)	0.4 (0.3 to 0.5)	31.7 (22.2 to 42.8)	0.5 (0.3 to 0.6)	12.9 (4.4 to 22.4)	30.4 (14.2 to 45.6)
DALYs (disability‐adjusted life years)	Number	11,526,683 (11,021,135 to 12,107,827)	154,375 (142,696 to 166,951)	20,310,187 (18,744,799 to 21,866,646)	315,126 (278,546 to 349,292)	21.4 (11.5 to 30.5)	41.7 (20.2 to 61.7)
	Rate	524.9 (501.8 to 551.1)	8.2 (7.5 to 8.8)	473.8 (437.3 to 510.5)	8 (7 to 8.8)	−9.7 (−17.1 to −3)	−2.7 (−17.1 to 10.5)

^a^ Data in parentheses are 95% uncertainty intervals.

### 3.2 The attributable burden to risk factors

The attributed number of deaths to BC included risk factors in GBD for females had an increasing pattern in all risk factors including high BMI (percent change: 197.2%), high FPG (160.4%), low physical activity (92.1%), secondhand smoke (70.2%), diet in high red meat (65.6%), alcohol use (36.5%), and smoking (16.3%). The top three risk factors with highest attributable mortality in BC patients in 2019 were high FPG (51,060 [9896–113,534]), high BMI (45,203 [18,772–81,168]), and alcohol use (37,718 [30,668–45,084]) which the first two experienced increase in rank during the study period. The all‐age number of DALYs attributed to BC risk factors in this period also all have increased as high BMI (231.8%), high FPG (155.9%), low physical activity (79.9%), secondhand smoke (63.8%), a diet high in red meat (58.8%), alcohol use (28.2%), and smoking (7.8%). The top three risk factors with highest attributable DALYs in 2019 were high FPG (1,239,759 [237,905–2,787,940]), alcohol use (1,087,753 [880,435–1,302,871]), and high BMI (958,187 [305,774–1,822,234]), which the first two experienced increase in rank during the study period, indicating greater impact of the metabolic risk factors in BC (Table 2; Table S2).

**TABLE 2:** The global all‐age numbers and age‐standardized rates of deaths and disability‐adjusted life years (DALYs) attributed to various breast cancer risk factors for females, in 1990 and 2019 and the percentages of change in this period

Risk factor	Measure	Metric	Year		% Change (1990 to 2019)
			1990	2019	
All risk factors	Deaths	Number	93,034 (71,550 to 118,362)^a^	174,752 (124,414 to 238,420)	87.8 (66.2 to 107.8)
		Rate	4.5 (3.4 to 5.7)	4 (2.8 to 5.4)	−10.4 (−20.4 to −1.3)
	DALYs	Number	2,604,747 (2,028,595 to 3,284,783)	4,568,126 (3,273,418 to 6,178,581)	75.4 (54.5 to 95.3)
		Rate	120.6 (93.6 to 152)	104.8 (75.1 to 141.7)	−13.1 (−23.2 to −3.6)
Behavioral risks	Deaths	Number	65,507 (54,529 to 75,841)	96,176 (77,861 to 114,207)	46.8 (37.5 to 55.9)
		Rate	3.1 (2.6 to 3.6)	2.2 (1.8 to 2.6)	−28.8 (−33.4 to −24.3)
	DALYs	Number	1,992,711 (1,628,409 to 2,316,758)	2,771,233 (2,195,102 to 3,302,081)	39.1 (29.7 to 48.4)
		Rate	91.4 (74.8 to 106.1)	64.3 (50.9 to 76.6)	−29.6 (−34.5 to −24.6)
Alcohol use	Deaths	Number	27,041 (22,267 to 32,290)	36,319 (29,459 to 43,523)	34.3 (27.4 to 41.5)
		Rate	1.3 (1.1 to 1.5)	0.8 (0.7 to 1)	−35 (−38.2 to −31.6)
	DALYs	Number	831,729 (685,936 to 985,548)	1,049,228 (849,510 to 1,258,594)	26.2 (19.7 to 33.7)
		Rate	38.1 (31.4 to 45.2)	24.4 (19.7 to 29.2)	−36.1 (−39.3 to −32.2)
Diet high in red meat	Deaths	Number	13,432 (6455 to 17,755)	22,105 (10,446 to 29,673)	64.6 (52.5 to 76.5)
		Rate	0.6 (0.3 to 0.8)	0.5 (0.2 to 0.7)	−20.2 (−25.9 to −14.6)
	DALYs	Number	406,210 (201,759 to 539,830)	641,072 (306,863 to 857,825)	57.8 (45.4 to 70.5)
		Rate	18.6 (9.2 to 24.7)	14.9 (7.1 to 19.9)	−19.8 (−26.2 to −13.5)
Low physical activity	Deaths	Number	4412 (2006 to 7693)	8475 (4078 to 14,305)	92.1 (73.5 to 113.5)
		Rate	0.2 (0.1 to 0.4)	0.2 (0.1 to 0.3)	−11 (−18.8 to −0.9)
	DALYs	Number	109,946 (51,946 to 202,204)	197,797 (97,517 to 345,136)	79.9 (63.2 to 100.3)
		Rate	5.1 (2.4 to 9.4)	4.6 (2.3 to 8)	−11.2 (−19.3 to −0.6)
Smoking	Deaths	Number	16,295 (11,864 to 21,002)	18,958 (13,578 to 24,787)	16.3 (9.8 to 22.7)
		Rate	0.8 (0.6 to 1)	0.4 (0.3 to 0.6)	−43.7 (−46.8 to −40.5)
	DALYs	Number	476,106 (341,399 to 620,974)	513,437 (361,957 to 674,442)	7.8 (1.6 to 13.6)
		Rate	22 (15.8 to 28.7)	11.8 (8.3 to 15.5)	−46.5 (−49.8 to −43.5)
Secondhand smoke	Deaths	Number	9903 (2357 to 16,946)	16,833 (3956 to 29,039)	70 (54.2 to 86.2)
		Rate	0.5 (0.1 to 0.8)	0.4 (0.1 to 0.7)	−15 (−22.9 to −6.9)
	DALYs	Number	340,940 (81,842 to 580,568)	557,662 (134,193 to 967,772)	63.6 (47.8 to 78.8)
		Rate	15.4 (3.7 to 26.2)	13.1 (3.1 to 22.7)	−15.2 (−23.4 to −7.2)
Metabolic risks	Deaths	Number	33,646 (13,124 to 62,346)	91,986 (40,256 to 161,745)	173.4 (142.3 to 244.7)
		Rate	1.6 (0.6 to 3)	2.1 (0.9 to 3.7)	26.6 (12.2 to 58.8)
	DALYs	Number	747,640 (254,066 to 1,448,363)	2,103,186 (844,005 to 3,802,259)	181.3 (144 to 296)
		Rate	35.7 (12.4 to 68.8)	47.3 (18.6 to 85.7)	32.7 (15.5 to 80.4)
High body‐mass index	Deaths	Number	15,210 (4467 to 30,773)	45,203 (18,772 to 81,168)	197.2 (142.4 to 369.5)
		Rate	0.7 (0.2 to 1.5)	1 (0.4 to 1.8)	35.4 (10 to 105)
	DALYs	Number	288,796 (40,552 to 644,192)	958,187 (305,774 to 1,822,234)	231.8 (140 to 677.2)
		Rate	14.2 (2.5 to 31.1)	21 (6.3 to 40.6)	48.2 (7.3 to 194.3)
High fasting plasma glucose	Deaths	Number	19,608 (3730 to 43,213)	51,060 (9896 to 113,534)	160.4 (140.9 to 184.4)
		Rate	1 (0.2 to 2.1)	1.2 (0.2 to 2.6)	22.3 (13.4 to 33)
	DALYs	Number	484,386 (88,936 to 1,094,215)	1,239,759 (237,905 to 2,787,940)	155.9 (136 to 179.3)
		Rate	22.7 (4.2 to 51.1)	28.5 (5.5 to 64)	25.4 (15.6 to 36.9)

^a^ Data in parentheses are 95% uncertainty intervals.

### 3.3 QCI of BC

**FIGURE 1:**
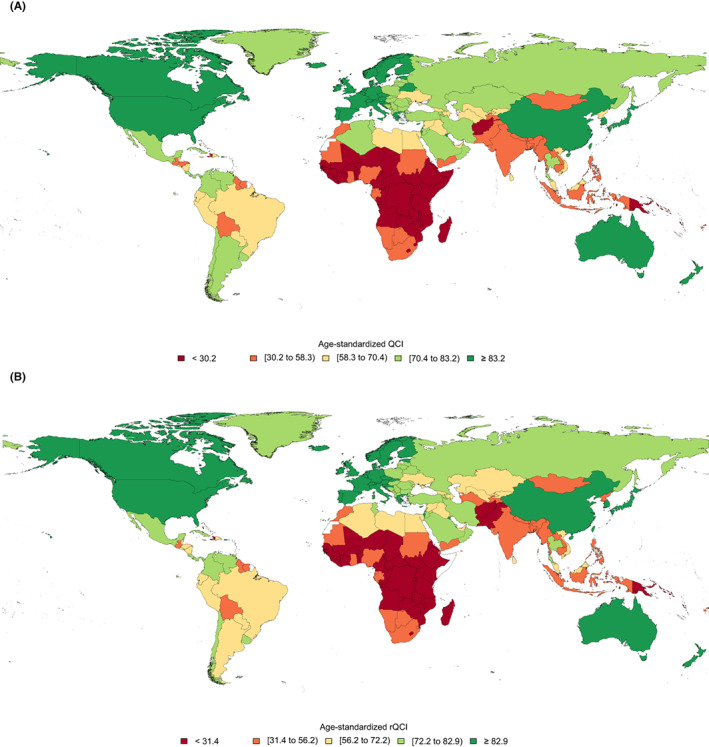
Global distribution of age‐standardized breast cancer (A) QCI and (B) rQCI for females, in 2019.

**FIGURE 2:**
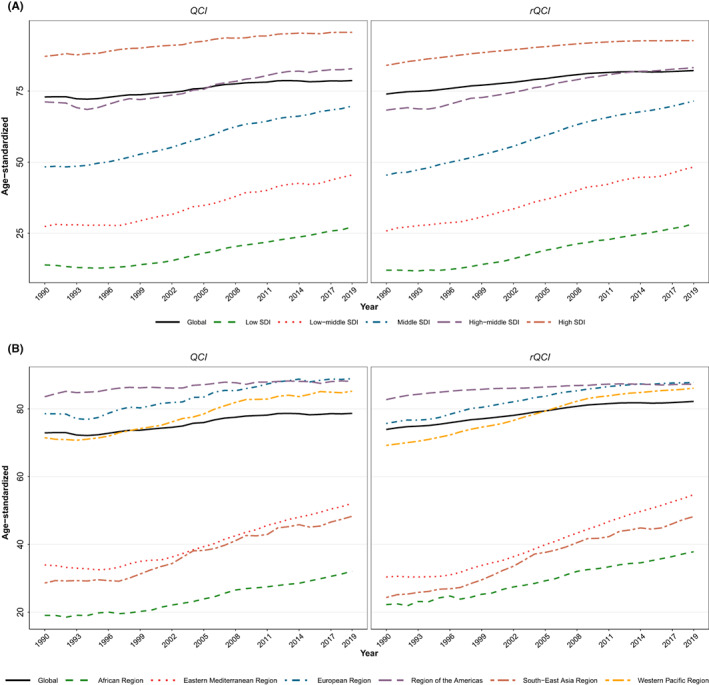
Time trend of age‐standardized breast cancer QCI and rQCI for females among (A) socio‐demographic index (SDI) quintiles and (B) six WHO regions compared to global trend, during 1990–2019.

**FIGURE 3:**
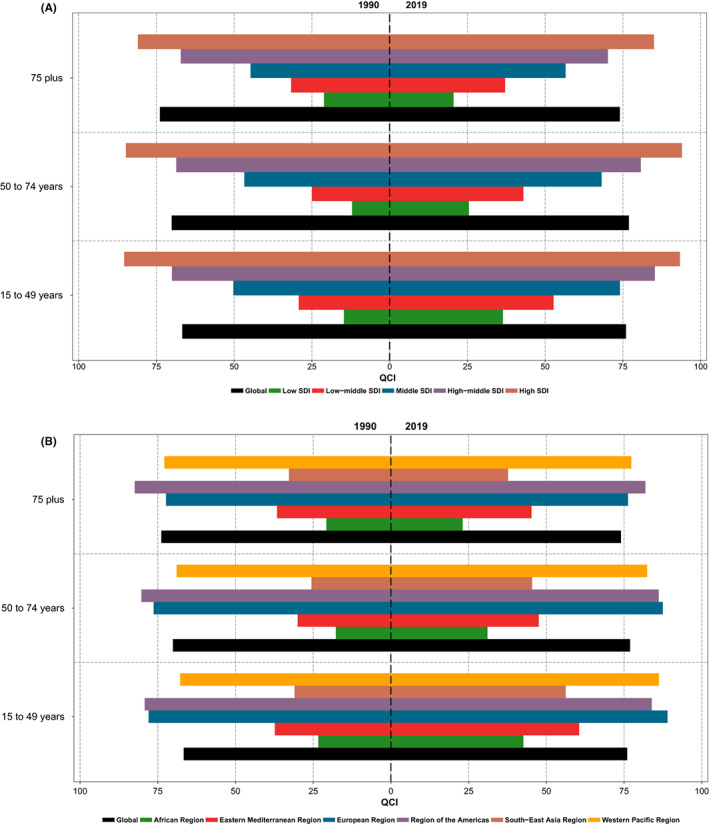
Patterns of global, (A) socio‐demographic index (SDI) quintiles, and (B) six WHO regions breast cancer QCI for females among three age categories of study.

The global estimated age‐standardized QCI for BC in 2019 was 78.7 for females and 69.9 for males. The details of QCI numbers presented here are focused on the female population. The global distribution of age‐standardized QCI for BC in females had various patterns in different countries (Figure 1A). The global BC QCI for females experienced an increasing trend from 72.9 in 1990 to 78.7 in 2019 showing a 7.9% increase (Figure 2). Investigation of global QCI in 2019 in three age categories showed 15–49 yeas, 50–74 years, and 75 plus age categories respectively had QCI of 76.0, 76.9, and 74.0. Also, the youngest age group had the highest improvement of QCI during the study period (Figure 3). Among the six WHO regions, the age‐standardized QCI in 2019 was highest in the European region as 88.9, followed by the region of the America as 88.1, the Western Pacific region as 85.2, the Eastern Mediterranean region as 52.1, the South‐East Asia region as 48.3, and the African region had the lowest QCI as 32.1. Among quintiles of SDI, the estimated QCI was 95.7 for high SDI, 82.9 for high‐middle SDI, 69.7 for middle SDI, 45.5 for low‐middle SDI, and 27.3 for low SDI regions. The five countries with the highest calculated QCI in 2019 were Iceland (100), Japan (99.8), Finland (98.8), Italy (98.6), and Norway (98.1). In contrast, the five countries with the lowest QCI were Eritrea (16.6), South Sudan (16.0), Mozambique (16.0), Somalia (8.2), and Central African Republic (5.3). Inequity among countries regarding BC QCI has reduced during the study period as the highest to lowest QCI was 54.9 in 1990 versus 18.9 in 2019 (Table S3).

### 3.4 Quality of care of BC risk factors (rQCI)

**FIGURE 4:**
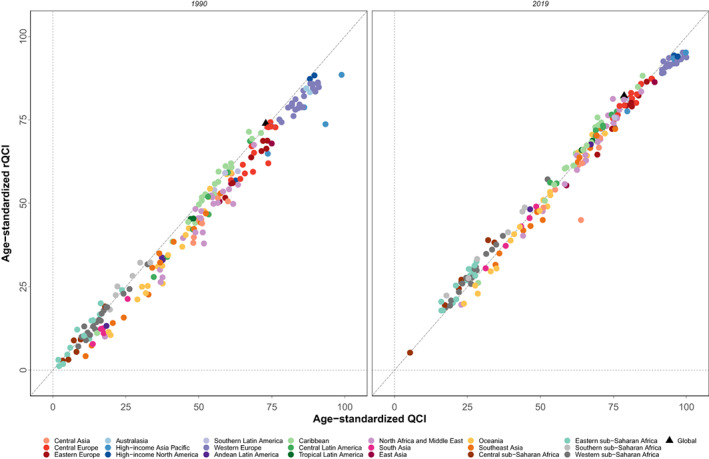
Age‐standardized breast cancer QCI against rQCI for females in countries of 21 Global Burden of Disease (GBD) regions and worldwide, in 1990 and 2019.

**FIGURE 5:**
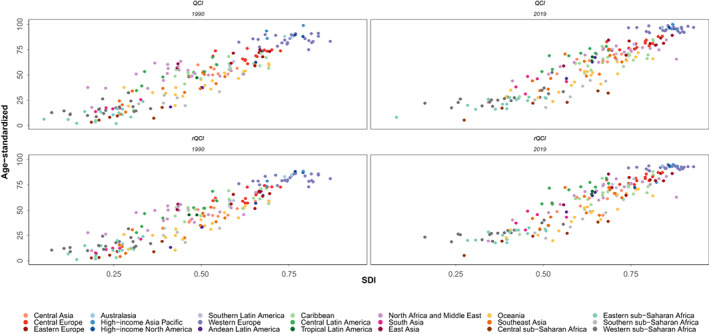
Age‐standardized breast cancer QCI and rQCI for females in countries of 21 Global Burden of Disease (GBD) regions against socio‐demographic index (SDI), in 1990 and 2019.

The global estimated age‐standardized rQCI for females was 82.2 in 2019. This index was estimated to be 73.9 in 1990 showing an 11.2% increase of BC rQCI. Among the WHO regions, the age‐standardized rQCI was highest in the European region as 87.8, followed by the region of the Americas as 87.3, the Western Pacific region as 86.1, the Eastern Mediterranean region as 54.7, the South‐East Asia region as 48.2, and the African region had the lowest rQCI as 37.8. Among quintiles of SDI, the calculated rQCI was 92.7 for high SDI, 83.2 for high‐middle SDI, 71.5 for middle SDI, 48.3 for low‐middle SDI, and 28.4 for low SDI regions. The five countries with the highest estimated rQCI in 2019 were Japan (95.2), Finland (95.2), Canada (94.3), New Zealand (94.0), and Sweden (94.0). On the other extreme, the five countries with the lowest rQCI were Guinea‐Bissau (19.3), Chad (18.8), Eritrea (17.9), South Sudan (17.9), and Central African Republic (5.2) (Figure 1B). Inequity among countries regarding BC QCI has reduced during the study period as the highest to lowest rQCI was 73.8 in 1990 versus 18.3 in 2019. Significant correlation between age‐standardized rQCI and QCI values statistically (correlation coefficient: 0.9934, *p*‐value < 0.001, in 2019; Figure 4), and also in various categories and years, represent this new index as a promising tool for assessing quality of care of diseases' risk factors (Figure 5; Table S3).

## 4 DISCUSSION

The major findings of this study were the increasing trend of global all‐ages number of BC incidence, mortality, and DALYs during the study period. Although the age‐standardized BC incidence rate increased, the mortality and DALY rates decreased in the past three decades. The attributable burden to BC risk factors increased both in numbers and rates, and responsible metabolic risks including high FPG and high BMI were at the top of attributable burden to risk factors. Estimated QCI was higher in regions with higher SDI and countries in these regions had a better status in this regard. In contrast, countries in lower SDI regions had a worse QCI of BC. Also, rQCI had a similar pattern and was higher in more developed countries. Investigating the trend of numbers, both QCI and rQCI improved during the study period showing improved care of BC and its responsible risk factors in the past decades. Also, the disparity of BC quality of care decreased among countries during the investigated period.

One of the important findings of this study was the increasing trend of incidence and burden of BC in terms of deaths and DALYs in all‐ages number, while inspecting the age‐standardized rates revealed a decreasing pattern for imposed burden. This finding which was more prominent in more developed regions could be attributed to many factors. Population aging, early menarche and late menopause ‐which both pose the breast tissue more to estrogen‐, lower parity, lower breast‐feeding prevalence, increased consumption of contraceptive hormones, the emergence of hormone replacement therapy for various aims, increased intake of alcohol and smoking, lower physical activity, increased prevalence of obesity and overweight, genetic predisposition, and higher exposure to radiation as in medical procedures, has been proposed to be responsible for the increasing trend of BC.^5^, ^6^, ^43^, ^44^ The emergence of screening programs and diagnostic measures like mammography with widespread use in many regions of the world is suggested to be another reason for this trend, although in some cases this issue led to higher detection of asymptomatic cases.^3^, ^7^ The decreasing rates of BC deaths and burden, especially in more developed areas, is thought to be due to the efficacy of the screening programs in detecting the lesions in early stages and availability of more novel treatments like chemo‐radiation therapies in such countries.^45^ A possible explanation for lower statistics of BC incidence except for the insufficient screening programs, in lesser developed areas, could be the major flaws in the cancer registry of these countries, leading to imprecise estimations.^46^, ^47^

Another main finding of this study was the assessed index of quality of care for BC which provided information in different scales. The most noticeable result was the higher quality of care in regions with higher SDI which means a better provided care for patients with BC in countries with better socio‐economic status. The fact that quality of care depends on a timely access to care and appropriate treatment, which is more available in higher SDI areas, could be the mainstay of this finding.^45^ In fact, the lower socio‐economic level and limited resources in the less developed areas are associated with low education and awareness, suboptimal standards of life, insufficient social support, risk‐leveraging behaviors and lifestyle, and ill‐timed and inadequate access to healthcare services and all these lead to poor quality of care.^48^, ^49^, ^50^ Therefore, resource allocation and appropriate funding of various healthcare sectors are needed to efficiently handle the burden of BC in such countries.^51^

In order to find out the strategies that countries with the highest BC QCI have implemented, we investigated the national programs of BC management in these countries. The Icelandic Cancer Society (ICS) at Iceland, with the highest score of QCI in this study, started the national BC screening program in 1989 and conducts mammography investigation for women aged 40–69 years old every 2 years. Preliminary results of this program published in 2007 revealed that an almost 40% reduction in BC mortality happened by the implemented screening program.^52^ The clinics of this precious program also recruited more than 80,000 women in a cohort study named the Cancer Detection Clinic (CDC) cohort between the years 1979–1995 and by conducting cervical and breast cancers screening provided valuable evidence for cancer prevention in this country through improving material for effective public health policies.^53^, ^54^ The second rank was Japan in this study. Japan was one of the pioneers of BC screening in the world and the first country in South‐East Asia that started its national screening program in 1987 clinical breast examination and later added mammography, for women aged ≥40 years old in 2 years intervals, and had an enormous number of 227.3 mammography units per million women aged 50–69 years in 2013.^55^, ^56^ Also, Japan routinely provides updated guidelines as The Japanese Breast Cancer Society Clinical Practice Guidelines for Breast Cancer Screening and Diagnosis to refine its BC screening program and keep up with the latest findings.^55^, ^57^ Finland, at the third rank in this study, was one of the first countries which started the Finnish population‐based BC screening program in 1987 at screening centers of the cancer society of Finland.^58^ A unique feature of the screening program in Finland was the subsequent massive follow‐up sessions which included large groups of its population.^59^ As an example, in a study between the years 1991–2000, the Finnish cancer centers invited one million women aged 50–64 years old to the BC screening program.^60^ in addition to the importance of cancer screening programs in providing quality care for BC patients, these countries were also successful in providing and updating the treatment approaches to benefit the diagnosed patients with this cancer in different stages of the disease.^61^, ^62^, ^63^

This study provided an additional view on BC risk factors epidemiology and quality of care with the novel index introduced in this study. The fast‐growing trend of BC extrinsic and modifiable risk factors is a major concern as the burden attributable to these measurable risks has raised during the study periods. Findings of quality assessment showed that countries with higher BC QCI almost had a consistent higher rQCI, which highlights the importance of controlling the outgrowing BC risk factors in better BC epidemic prevention, before the incidence of the diseases in individuals. An effective management of these risk factors could avert the risk of the disease in populations in long‐term.^64^, ^65^ The noticeable attributable burden to high FPG, high BMI, and alcohol use as the main modifiable risk factors assessed in this study highlight the role of metabolic and behavioral risks in the development of BC. Widespread pandemic of overweight and obesity is a major concern in BC too, as this risk factor is associated with higher BC incidence in postmenopausal women.^6^, ^66^, ^67^, ^68^ Both high BMI and FPG contribute to metabolic syndrome which is showed to be highly correlated with BC risk in women after menopause.^69^, ^70^ The modifiable nature of these risk factors call attention to the significance of primary prevention by modifying the underlying causes leading to disease, in handling the burden of BC.^71^

To ensure desirable BC quality of care, various global guidelines and initiatives provide updated guides for clinicians and health authorities. The National Comprehensive Cancer Network (NCCN) Clinical Practice Guidelines in Oncology are example of these guides that provide beneficial information on screening, diagnosis, and treatment options of BC in various stages that could be adopted by health systems to improve the BC quality of care.^72^, ^73^ A precious effort to improve the quality of cancer care in the United States is the National Initiative on Cancer Care Quality (NICCQ) promoted by the American Society of Clinical Oncology (ASCO) that could be adjusted and implemented in other countries to ensure BC quality of care.^74^, ^75^ In this regard, some international efforts like the Breast Health Global Initiative (BHGI) have been made to provide evidence‐based and resource‐oriented protocols to manage BC, especially in low‐ and middle‐income countries which are exposed to the hard to control BC burden.^76^, ^77^ Considering the different epidemiology of BC and availability of resources in developing countries, proposing a unique guideline for all limited resources countries is an “out‐of‐date” and inefficient recommendation and encouraging the countries to have their own protocols based on their resources, cultures, values, and health priorities is highly recommended.^78^, ^79^

Some useful quality indicators (QIs) specified for BC have been introduced and validated that could be used to assess BC quality of care in clinical settings to provide information for clinicians and health managers. The most practical one is developed by the European Society of Breast Cancer Specialists (EUSOMA) and consists of a set of QIs for BC diagnosis, surgery and loco‐regional treatment, radiation therapy and local control, surgery and quality of life, systemic treatment, and staging, counseling, follow‐up and rehabilitation aspects with subset indicators.^80^ It is showed that higher adherence to QIs is associated with better survival rates and disease outcomes in patients with BC.^17^, ^81^

The global disparity in BC quality of care showed to decrease among countries in the past three decades in terms of BC and its risk factors care quality in this study. One practical approach to reduce cancer care disparities and improve disease outcomes is patient navigation.^82^ Patient navigation is an individualized service delivery intervention to provide timely cancer diagnosis and treatment by removing obstacles to proper care.^48^ Strong evidence has proved the supporting and effective role of patient navigation in improving many aspects of BC care.^83^, ^84^ This beneficial strategy is also highly effective in reducing disparities and gaps in cancer care of racial minorities and patients with limited income and health budgets.^10^, ^85^

The substantial limitations of this study return to the availability and quality of the primary data and data processing and modeling complications in GBD studies. Although these limitations exist, GBD study tries to reassess the data processing method for each round of study. For example, for the GBD 2019 study, the clarification of the reference and alternative methods for measuring outcomes and enhancements in modelings by implementing standard locations for estimating effects were conducted to provide the most precise estimations.^19^, ^20^ In GBD 2019 risk studies, reassessments of dose–response relationships and further investigations on the combined effects of risk factors were taken to make the risk estimations more accurate.^20^ One of the major limitations of the GBD database on BC risk factors, are the many missing proven risks of the disease including the major demographic, clinical, genetic, and environmental factors which need to be added in future estimations. Regarding the rQCI, restriction of this method to only females because of limited data on BC risk factors in males, exclusion of one country (Somalia) because of deficient data in GBD database, and estimation of this index only for age‐standardized rates of YLL and YLD were the major limitations. A major limitation of the acquired database regarding studying the epidemiology of cancers, is the cancer survival measures and metrics, which is highly suggested to be added in future to make the investigations more robust. Although the QCI and rQCI indices try to measure and depict the BC and its risk factors quality of care, we should keep in mind that these methods are proxies of care and the true perceived and patient‐centered quality care would happen and be measured beyond these proxies and aggregated data. However, using such robust methods could benefit many areas of the world to initiate plans and policies and improve the care of patients with BC and the existing risk factors. The most important strength of this study is the updated provided data on epidemiology and burden of BC and its risk factors besides the quality of care of this condition in various scales. Also, the new index for assessment of the quality of care of BC risk factors is the novelty of this study and could be used to evaluate management of other conditions, too.

## 5 CONCLUSION

In spite of the increasing trend of BC incidence, the imposed burden has been restrained partially in recent years. In contrast, the attributable burden to BC risk factors has increased remarkably and is the major concern in BC pandemic management. Countries with better socio‐economic status were more successful in controlling the BC burden and provided better quality of care regarding both the condition and its responsible risk factors. Furthermore, equitable efforts and resource allocation are needed to address different aspects of BC globally.

## AUTHORS' CONTRIBUTION

Farshad Farzadfar designed the study. Sina Azadnajafabad and Sahar Saeedi Moghaddam developed the new codes, analyzed the data, and prepared the study results. Sina Azadnajafabad investigated the results and drafted the first manuscript. Mohammad Keykhaei, Negar Rezaei, Esmaeil Mohammadi, Azin Ghamari, and Sarvenaz Shahin helped in the literature search and interpretation of the findings. Parnian Shobeiri, Erfan Ghasemi, and Naser Ahmadi contributed to data curation and development of codes. Nazila Rezaei managed administrative aspects of study. Sina Azadnajafabad, Sahar Saeedi Moghaddam, Mahdi Aghili, Ahmad Kaviani, Bagher Larijani, and Farshad Farzadfar commented on and revised majorly the manuscript. All authors critically commented on the draft and provided their approval for the submission. Farshad Farzadfar was the principal investigator and supervised all stages of this study.

## FUNDING INFORMATION

This study had no funding support and received no grants.

## CONFLICT OF INTEREST

The authors of this study declare no conflicts of interest.

## ETHICAL STATEMENT

This study was approved by the institutional review board of Endocrinology and Metabolism Research Institute at Tehran University of Medical Sciences (IR.TUMS.EMRI.REC.1400.031). Data reported in this study was aggregated epidemiologic data and no individual data was reported.

## Supporting information


Table S1

## Data Availability

The dataset supporting the conclusions of this article is available in the GBD repository, [http://ghdx.healthdata.org/gbd‐results‐tool].
