# Supplementary material for: Expansion of the quality of care index on breast cancer and its risk factors using the global burden of disease study 2019
Source: Cancer Med. 2022 Jun 30;12(2):1729–43. doi: 10.1002/cam4.4951 (PMC9883412; doi:10.1002/cam4.4951)
Supplement: Supplementary file 1 — Table S1 [file CAM4-12-1729-s001.pdf]

Supplementary table 1. The regional, socio-demographic index (SDI) quintiles, and national trend of epidemiologic indices of breast cancer, number for all-ages and age-standardized rates with 95% uncertainty interval (UI), for each and both sexes, in 1990 and 2019.

		1990												2019											
		Incidence		Prevalence		Deaths		YLLs (Years of Life Lost)		YLDs (Years Lived with Disability)		DALYs (Disability-Adjusted Life Years)		Incidence		Prevalence		Deaths		YLLs (Years of Life Lost)		YLDs (Years Lived with Disability)		DALYs (Disability-Adjusted Life Years)	
Location	Sex	Number	Rate	Number	Rate	Number	Rate	Number	Rate	Number	Rate	Number	Rate	Number	Rate	Number	Rate	Number	Rate	Number	Rate	Number	Rate	Number	Rate
WHO regions																									
African Region	Both	28096 (24284 to 32118)	11.4 (9.8 to 13.1)	190845 (168582 to 216032)	76 (67 to 86)	21231 (18468 to 24597)	9.3 (8.1 to 10.8)	677881 (591562 to 787120)	254.1 (221 to 295.4)	15954 (11363 to 21645)	6.3 (4.5 to 8.4)	693836 (603852 to 803996)	260.4 (226.2 to 302.8)	92057 (78647 to 107212)	16.4 (14.2 to 18.8)	657577 (570373 to 754512)	113.2 (99.4 to 128.9)	58945 (49834 to 68355)	11.7 (10 to 13.4)	1847571 (1539414 to 2183572)	305.6 (257.4 to 357.2)	53922 (37311 to 74116)	9.3 (6.5 to 12.6)	1901493 (1586921 to 2244956)	314.9 (264.9 to 366.9)
	Female	26736 (22964 to 30701)	21.1 (18 to 24.3)	184044 (161967 to 208781)	143.3 (125.9 to 162.8)	20004 (17348 to 23339)	16.8 (14.5 to 19.6)	648037 (564614 to 758877)	477.9 (415.5 to 558.4)	15207 (10771 to 20575)	11.7 (8.3 to 15.8)	663244 (576201 to 773253)	489.6 (425.9 to 573.8)	89504 (76303 to 104339)	30 (25.8 to 34.6)	642647 (557442 to 739377)	209.6 (183.5 to 239.1)	56906 (47929 to 66423)	20.9 (17.9 to 24.1)	1798449 (1492978 to 2124462)	564.4 (472.9 to 663.3)	52401 (36154 to 71996)	17.1 (11.9 to 23.1)	1850849 (1537100 to 2189049)	581.4 (487.6 to 680.1)
	Male	1360 (1148 to 1589)	1.4 (1.2 to 1.6)	6801 (5878 to 7864)	6.4 (5.5 to 7.4)	1227 (1031 to 1442)	1.4 (1.1 to 1.6)	29844 (25181 to 35012)	27.3 (23 to 32)	747 (500 to 1019)	0.7 (0.5 to 1)	30591 (25782 to 35903)	28.1 (23.7 to 32.9)	2553 (1981 to 3338)	1.3 (1 to 1.6)	14930 (11912 to 19032)	6.7 (5.4 to 8.5)	2039 (1611 to 2667)	1.1 (0.9 to 1.4)	49123 (38058 to 64747)	21.2 (16.7 to 27.9)	1521 (1007 to 2207)	0.7 (0.5 to 1)	50644 (39297 to 66574)	21.9 (17.3 to 28.8)
Eastern Mediterranean Region	Both	27177 (22383 to 33173)	12.9 (10.5 to 16)	210473 (180725 to 248827)	100.7 (86.6 to 119.8)	18352 (14883 to 22913)	9.3 (7.5 to 11.8)	614998 (506053 to 751643)	277.6 (226.5 to 343)	16340 (11204 to 23497)	7.7 (5.2 to 11.1)	631338 (520320 to 770836)	285.3 (233.1 to 352.5)	123055 (106073 to 143980)	23.2 (20.1 to 27)	993214 (877022 to 1137741)	186.7 (166.7 to 212.6)	59880 (50719 to 71958)	12.5 (10.7 to 14.8)	2027259 (1694222 to 2457205)	366.6 (309.5 to 441.7)	76870 (54066 to 105716)	14.3 (10.1 to 19.5)	2104129 (1767865 to 2538114)	380.9 (323 to 454.8)
	Female	26380 (21586 to 32374)	26 (21 to 32.3)	206161 (176633 to 244449)	205.9 (176.1 to 246.3)	17630 (14107 to 22187)	18.5 (14.5 to 23.6)	598542 (488807 to 735051)	562.3 (455.1 to 700.1)	15889 (10869 to 22965)	15.5 (10.5 to 22.5)	614431 (502904 to 754446)	577.8 (467.8 to 719)	121096 (104300 to 142149)	47.6 (41 to 55.6)	980703 (864296 to 1125232)	384.5 (342.7 to 438.2)	58460 (49389 to 70621)	25.2 (21.4 to 30.2)	1991787 (1661070 to 2417053)	751.7 (632 to 909.2)	75639 (53062 to 104208)	29.3 (20.7 to 40.2)	2067425 (1736241 to 2504651)	781 (659.5 to 936.7)
	Male	797 (636 to 988)	1 (0.7 to 1.2)	4313 (3609 to 5149)	4.6 (3.8 to 5.6)	722 (569 to 901)	0.9 (0.7 to 1.2)	16456 (13351 to 20210)	17.6 (14.1 to 21.7)	451 (308 to 616)	0.5 (0.3 to 0.7)	16907 (13768 to 20675)	18.1 (14.5 to 22.3)	1958 (1545 to 2464)	0.9 (0.7 to 1.2)	12511 (10147 to 15408)	5.6 (4.5 to 6.8)	1420 (1103 to 1789)	0.7 (0.6 to 0.9)	35472 (27617 to 45070)	15.3 (11.9 to 19.3)	1231 (811 to 1694)	0.6 (0.4 to 0.8)	36703 (28696 to 46530)	15.9 (12.4 to 19.9)
European Region	Both	343405 (333745 to 350030)	33.7 (32.8 to 34.3)	3606775 (3311242 to 3959581)	349.7 (323.2 to 383)	144156 (138348 to 146980)	14 (13.4 to 14.3)	3706668 (3616241 to 3763348)	366.1 (357.3 to 371.8)	250631 (176476 to 339633)	24.4 (17.2 to 33.1)	3957299 (3840699 to 4064437)	390.5 (379.1 to 401)	532667 (475502 to 596044)	37.1 (33.2 to 41.7)	5650712 (5160881 to 6204279)	386.3 (353 to 425.4)	170529 (155141 to 181504)	10.8 (9.9 to 11.5)	3742280 (3508246 to 3978708)	264.3 (249 to 280.8)	392303 (273621 to 538859)	27.2 (18.9 to 37.5)	4134584 (3850777 to 4430530)	291.5 (272.6 to 312.6)
	Female	341107 (331425 to 347780)	60.2 (58.7 to 61.3)	3586481 (3292323 to 3937642)	616.5 (572.7 to 668.3)	143070 (137301 to 145893)	23.8 (23 to 24.3)	3680376 (3589966 to 3737462)	664.1 (649.8 to 674.3)	248789 (175223 to 337260)	43.5 (30.5 to 58.9)	3929165 (3813848 to 4036175)	707.6 (688.6 to 726)	527648 (470623 to 590560)	68.3 (61.2 to 76.8)	5605276 (5120219 to 6154869)	708.4 (648.4 to 780.8)	168748 (153465 to 179630)	19.1 (17.6 to 20.3)	3704377 (3471969 to 3941794)	489.1 (461.2 to 520.8)	388205 (270802 to 533993)	50 (34.6 to 69.1)	4092582 (3809617 to 4386078)	539.1 (504.6 to 578.2)
	Male	2298 (2198 to 2395)	0.5 (0.5 to 0.6)	20294 (18614 to 22225)	4.7 (4.3 to 5.2)	1085 (1041 to 1132)	0.3 (0.3 to 0.3)	26292 (25217 to 27458)	5.9 (5.7 to 6.2)	1843 (1316 to 2476)	0.4 (0.3 to 0.6)	28134 (26852 to 29459)	6.4 (6.1 to 6.7)	5019 (4412 to 5644)	0.7 (0.7 to 0.8)	45436 (40562 to 50956)	6.8 (6.1 to 7.6)	1780 (1626 to 1924)	0.3 (0.2 to 0.3)	37903 (34781 to 40955)	5.8 (5.3 to 6.2)	4098 (2880 to 5576)	0.6 (0.4 to 0.8)	42001 (38327 to 45727)	6.4 (5.8 to 6.9)
Region of the Americas	Both	255927 (247708 to 261536)	41.7 (40.4 to 42.6)	2540194 (2389484 to 2733219)	415.7 (391.2 to 448.2)	78707 (75358 to 80525)	13 (12.4 to 13.3)	2094118 (2041421 to 2130205)	340 (331.2 to 346)	180842 (128208 to 244965)	29.5 (20.9 to 39.9)	2274960 (2202012 to 2350597)	369.5 (357.4 to 381.9)	433809 (379718 to 493905)	35 (30.6 to 39.9)	4418468 (3942250 to 4921028)	354.3 (315.7 to 394.4)	119077 (110684 to 125852)	9.4 (8.8 to 10)	2931868 (2777321 to 3094734)	240.5 (227.7 to 254.1)	305149 (213048 to 415729)	24.6 (17.2 to 33.5)	3237017 (3049704 to 3445690)	265.1 (249.8 to 282.1)

		1990												2019											
		Incidence		Prevalence		Deaths		YLLs (Years of Life Lost)		YLDs (Years Lived with Disability)		DALYs (Disability-Adjusted Life Years)		Incidence		Prevalence		Deaths		YLLs (Years of Life Lost)		YLDs (Years Lived with Disability)		DALYs (Disability-Adjusted Life Years)	
Location	Sex	Number	Rate	Number	Rate	Number	Rate	Number	Rate	Number	Rate	Number	Rate	Number	Rate	Number	Rate	Number	Rate	Number	Rate	Number	Rate	Number	Rate
	Female	253801 (245573 to 259372)	76.7 (74.4 to 78.4)	2519974 (2370872 to 2711568)	757.7 (715 to 813)	78004 (74647 to 79840)	23.4 (22.4 to 23.9)	2077023 (2024371 to 2113215)	635.7 (620.7 to 646.7)	179110 (126904 to 242677)	53.9 (38.2 to 73.1)	2256133 (2183922 to 2331442)	689.6 (668 to 712.3)	429443 (374960 to 489456)	65.2 (57.1 to 74.3)	4378078 (3903111 to 4881044)	657.4 (584.6 to 733.2)	117666 (109358 to 124410)	17.2 (16.1 to 18.1)	2899437 (2746515 to 3061600)	451.2 (427.5 to 476.9)	301673 (210576 to 411683)	45.7 (31.9 to 62.4)	3201110 (3015772 to 3408746)	496.9 (467.8 to 528.9)
	Male	2126 (2040 to 2216)	0.8 (0.7 to 0.8)	20221 (18672 to 22216)	7.4 (6.8 to 8.1)	703 (678 to 728)	0.3 (0.3 to 0.3)	17095 (16534 to 17678)	6.1 (5.9 to 6.3)	1732 (1234 to 2295)	0.6 (0.4 to 0.8)	18827 (18019 to 19659)	6.7 (6.4 to 7)	4366 (3727 to 5122)	0.7 (0.6 to 0.9)	40389 (34994 to 46847)	6.9 (6 to 8)	1412 (1307 to 1510)	0.2 (0.2 to 0.3)	32431 (30314 to 34697)	5.6 (5.2 to 6)	3476 (2403 to 4723)	0.6 (0.4 to 0.8)	35906 (33440 to 38546)	6.2 (5.7 to 6.6)
South-East Asia Region	Both	74752 (66072 to 83795)	8.8 (7.9 to 9.9)	564436 (504923 to 628434)	67.7 (60.5 to 75.9)	51672 (45486 to 58308)	6.6 (5.8 to 7.5)	1827660 (1605043 to 2060630)	202.2 (178.3 to 228.1)	44500 (30825 to 59628)	5.2 (3.6 to 7)	1872160 (1644110 to 2113920)	207.4 (183 to 233.6)	253080 (214124 to 295646)	13.4 (11.3 to 15.6)	2039248 (1766067 to 2352060)	107.3 (93.2 to 123.1)	137718 (115463 to 162319)	7.7 (6.5 to 9)	4408510 (3690361 to 5214249)	227.5 (190.8 to 269.2)	156593 (108747 to 217542)	8.2 (5.7 to 11.4)	4565103 (3832557 to 5373939)	235.7 (198.1 to 277.4)
	Female	73049 (64441 to 82111)	17.5 (15.5 to 19.6)	554364 (495472 to 617995)	135 (120.8 to 151.2)	50253 (44123 to 56859)	12.9 (11.3 to 14.6)	1789268 (1567944 to 2029285)	404.4 (355.5 to 457.3)	43480 (30043 to 58310)	10.3 (7.1 to 13.8)	1832748 (1606960 to 2074388)	414.7 (365.1 to 468.5)	250266 (211148 to 292317)	26 (22 to 30.4)	2018939 (1746793 to 2330746)	209.2 (181.5 to 240.6)	135751 (113674 to 160384)	14.7 (12.3 to 17.4)	4357350 (3636515 to 5165128)	445 (372.1 to 527.6)	154668 (107140 to 215120)	16 (11.1 to 22.2)	4512018 (3779387 to 5324973)	461 (386.4 to 543.8)
	Male	1703 (1404 to 2034)	0.5 (0.4 to 0.6)	10072 (8429 to 11829)	2.8 (2.3 to 3.3)	1419 (1177 to 1695)	0.5 (0.4 to 0.6)	38392 (31655 to 45954)	10.5 (8.7 to 12.5)	1020 (676 to 1402)	0.3 (0.2 to 0.4)	39412 (32749 to 47135)	10.8 (9 to 12.8)	2814 (2256 to 3400)	0.3 (0.3 to 0.4)	20309 (16671 to 24072)	2.4 (2 to 2.8)	1967 (1562 to 2363)	0.3 (0.2 to 0.3)	51160 (40601 to 61740)	5.8 (4.6 to 7)	1925 (1315 to 2670)	0.2 (0.2 to 0.3)	53085 (42248 to 63888)	6.1 (4.8 to 7.3)
Western Pacific Region	Both	144946 (129669 to 160839)	11.3 (10.2 to 12.5)	1463594 (1298310 to 1629771)	117.9 (104.8 to 132)	65698 (58278 to 73500)	5.5 (4.9 to 6.1)	2114312 (1857967 to 2395215)	159.3 (140.5 to 179.7)	103382 (70903 to 141225)	8.1 (5.6 to 11.1)	2217694 (1968608 to 2502191)	167.5 (149.1 to 188.2)	559786 (471584 to 654430)	20.8 (17.5 to 24.3)	5451621 (4716294 to 6224472)	201.7 (174.5 to 230.2)	152144 (131692 to 174664)	5.7 (4.9 to 6.5)	4219762 (3639707 to 4870801)	156.8 (135.1 to 180.9)	395224 (266142 to 552147)	14.7 (9.9 to 20.4)	4614985 (4021923 to 5267122)	171.5 (149.3 to 195.5)
	Female	143889 (128641 to 159815)	22.1 (19.8 to 24.5)	1455110 (1290803 to 1620261)	227.4 (201.7 to 253.9)	64986 (57564 to 72800)	10.4 (9.2 to 11.6)	2095075 (1840488 to 2375168)	315.4 (277.4 to 356.6)	102619 (70399 to 140158)	15.8 (10.9 to 21.6)	2197694 (1949853 to 2481468)	331.2 (294.3 to 373)	551442 (463836 to 646269)	40.2 (33.7 to 47.3)	5387097 (4650507 to 6156335)	390.1 (336.9 to 447.2)	148704 (128533 to 170771)	10.6 (9.1 to 12.2)	4130152 (3555789 to 4776147)	303.3 (260.9 to 351)	389012 (260749 to 544120)	28.4 (19 to 39.6)	4519164 (3927197 to 5172192)	331.7 (288.1 to 379.6)
	Male	1056 (919 to 1202)	0.2 (0.2 to 0.2)	8484 (7309 to 10023)	1.5 (1.3 to 1.7)	712 (611 to 822)	0.2 (0.1 to 0.2)	19236 (16485 to 22285)	3.3 (2.8 to 3.8)	764 (527 to 1044)	0.1 (0.1 to 0.2)	20000 (17210 to 23070)	3.4 (3 to 3.9)	8344 (6596 to 10226)	0.6 (0.5 to 0.8)	64525 (52030 to 78145)	4.8 (3.9 to 5.8)	3440 (2774 to 4202)	0.3 (0.2 to 0.3)	89610 (71309 to 110346)	6.7 (5.3 to 8.1)	6212 (4079 to 8774)	0.5 (0.3 to 0.7)	95821 (77099 to 116738)	7.1 (5.8 to 8.6)
SDI Quintiles																									
High-middle SDI	Both	228474 (220963 to 236309)	20.9 (20.2 to 21.6)	2275451 (2104669 to 2483654)	210.5 (194 to 230.7)	104535 (100730 to 108322)	10 (9.6 to 10.3)	3009691 (2898295 to 3133701)	271.7 (261.6 to 282.8)	159733 (112868 to 215243)	14.6 (10.4 to 19.7)	3169424 (3050872 to 3304081)	286.4 (275.4 to 298.4)	516502 (464353 to 574104)	26 (23.3 to 28.9)	5060863 (4610380 to 5543887)	253.3 (231.3 to 277.7)	165934 (152738 to 179587)	8.3 (7.6 to 9)	4209032 (3887629 to 4580010)	212.3 (196.2 to 230.8)	361221 (248880 to 490409)	18.2 (12.5 to 24.7)	4570253 (4209331 to 4954003)	230.4 (212.2 to 249.9)
	Female	226752 (219258 to 234580)	38.5 (37.2 to 39.9)	2261068 (2090814 to 2468283)	382.3 (354.1 to 416.3)	103595 (99756 to 107384)	17.7 (17 to 18.3)	2984992 (2874164 to 3108809)	507.5 (488.3 to 528.9)	158421 (111907 to 213533)	26.8 (19 to 36.1)	3143413 (3024849 to 3277610)	534.3 (513.6 to 557.3)	510299 (458379 to 567966)	48.9 (43.8 to 54.5)	5009632 (4556925 to 5490445)	475.7 (433.3 to 522.4)	163520 (150455 to 177185)	14.9 (13.8 to 16.2)	4150729 (3829777 to 4520391)	400.8 (369.6 to 436.9)	356436 (245880 to 484148)	34.1 (23.5 to 46.5)	4507165 (4147354 to 4888828)	435 (400.7 to 473.3)
	Male	1722 (1635 to 1821)	0.4 (0.4 to 0.4)	14383 (13172 to 15846)	3.1 (2.8 to 3.4)	941 (887 to 1002)	0.2 (0.2 to 0.2)	24699 (23301 to 26327)	5 (4.7 to 5.4)	1311 (931 to 1776)	0.3 (0.2 to 0.4)	26011 (24391 to 27794)	5.3 (5 to 5.7)	6204 (5442 to 7073)	0.7 (0.6 to 0.8)	51231 (45201 to 58099)	5.5 (4.9 to 6.3)	2414 (2160 to 2691)	0.3 (0.2 to 0.3)	58303 (51928 to 65517)	6.2 (5.6 to 7)	4785 (3344 to 6569)	0.5 (0.4 to 0.7)	63088 (56278 to 70527)	6.7 (6 to 7.5)

		1990												2019											
		**Incidence**		**Prevalence**		**Deaths**		**YLLs (Years of Life Lost)**		**YLDs (Years Lived with Disability)**		**DALYs (Disability-Adjusted Life Years)**		**Incidence**		**Prevalence**		**Deaths**		**YLLs (Years of Life Lost)**		**YLDs (Years Lived with Disability)**		**DALYs (Disability-Adjusted Life Years)**	
**Location**	**Sex**	**Number**	**Rate**	**Number**	**Rate**	**Number**	**Rate**	**Number**	**Rate**	**Number**	**Rate**	**Number**	**Rate**	**Number**	**Rate**	**Number**	**Rate**	**Number**	**Rate**	**Number**	**Rate**	**Number**	**Rate**	**Number**	**Rate**
High SDI	Both	431076 (416846 to 440218)	43.2 (41.8 to 44.1)	4594024 (4254086 to 5032994)	456.3 (424.9 to 496.3)	137445 (130744 to 140804)	13.5 (12.9 to 13.9)	3392746 (3293843 to 3447991)	345.5 (335.9 to 351.1)	321046 (226067 to 433570)	32 (22.5 to 43.4)	3713793 (3586308 to 3845100)	377.5 (364.8 to 390.7)	678945 (606880 to 753696)	41.2 (36.9 to 45.6)	7509107 (6838190 to 8196814)	444.5 (403.8 to 483.4)	167553 (151809 to 176807)	9 (8.4 to 9.5)	3569297 (3370811 to 3711615)	221.6 (211.8 to 230)	514552 (357984 to 693878)	31.1 (21.4 to 42)	4083850 (3816265 to 4354647)	252.7 (237.7 to 269.3)
	Female	428085 (413890 to 437161)	79.3 (77 to 80.9)	4564740 (4226893 to 5000570)	828.1 (774 to 893.8)	136472 (129811 to 139840)	23.9 (22.8 to 24.4)	3370463 (3272079 to 3425938)	644.5 (628.6 to 654.5)	318518 (224194 to 429954)	58.5 (41 to 79.6)	3688982 (3562259 to 3819433)	703.1 (680.2 to 726.6)	673148 (601265 to 747674)	79.2 (70.8 to 87.7)	7451715 (6782098 to 8137959)	849.9 (771.3 to 923.9)	165968 (150337 to 175159)	16.7 (15.6 to 17.5)	3536857 (3339275 to 3678535)	427.9 (409.3 to 443.9)	509631 (354158 to 687426)	59.6 (40.9 to 80.6)	4046488 (3779696 to 4315580)	487.5 (459.8 to 518.8)
	Male	2991 (2876 to 3107)	0.7 (0.6 to 0.7)	29284 (26882 to 32139)	6.7 (6.1 to 7.3)	972 (939 to 1003)	0.2 (0.2 to 0.2)	22283 (21681 to 22926)	5 (4.9 to 5.1)	2528 (1799 to 3371)	0.6 (0.4 to 0.8)	24811 (23732 to 25928)	5.6 (5.3 to 5.8)	5797 (5055 to 6621)	0.7 (0.6 to 0.8)	57392 (50405 to 65367)	7 (6.2 to 8)	1586 (1460 to 1703)	0.2 (0.2 to 0.2)	32440 (30172 to 34629)	4.1 (3.8 to 4.4)	4921 (3411 to 6646)	0.6 (0.4 to 0.8)	37361 (34536 to 40383)	4.7 (4.3 to 5.1)
Low-middle SDI	Both	66098 (58235 to 74326)	9.6 (8.4 to 10.7)	489370 (441720 to 546573)	71.3 (64.1 to 80.1)	46520 (40557 to 52302)	7.2 (6.2 to 8.1)	1578921 (1396792 to 1780258)	214.7 (188.6 to 241.8)	38598 (27084 to 51428)	5.5 (3.9 to 7.4)	1617519 (1427988 to 1826025)	220.2 (193.3 to 247.9)	230747 (202809 to 259274)	15.4 (13.5 to 17.3)	1788970 (1603096 to 1996342)	118.3 (106.2 to 131.9)	127410 (110474 to 144919)	8.9 (7.8 to 10.2)	4055578 (3497018 to 4633463)	262.5 (227.4 to 299.5)	139272 (100005 to 187971)	9.2 (6.6 to 12.3)	4194850 (3622482 to 4785991)	271.7 (235.4 to 309.3)
	Female	64493 (56625 to 72656)	18.8 (16.3 to 21.1)	480405 (432595 to 536840)	141.3 (126.8 to 158.9)	45114 (39220 to 50962)	14 (11.9 to 15.8)	1543465 (1358404 to 1749350)	425.6 (371.7 to 480.7)	37672 (26407 to 50341)	10.9 (7.6 to 14.6)	1581138 (1390110 to 1789990)	436.5 (382 to 493)	227241 (199107 to 256008)	29.5 (25.9 to 33.2)	1765817 (1580328 to 1973610)	227.8 (204.3 to 254.2)	124911 (107972 to 142594)	16.9 (14.6 to 19.2)	3991633 (3433361 to 4576866)	505.9 (436.8 to 579.1)	136988 (98261 to 185102)	17.6 (12.7 to 23.7)	4128622 (3554632 to 4720565)	523.5 (452.1 to 597.2)
	Male	1605 (1338 to 1899)	0.6 (0.5 to 0.7)	8965 (7567 to 10436)	3 (2.5 to 3.5)	1406 (1176 to 1667)	0.6 (0.5 to 0.7)	35456 (29867 to 41988)	11.8 (9.9 to 13.9)	925 (629 to 1259)	0.3 (0.2 to 0.4)	36381 (30708 to 43063)	12.1 (10.1 to 14.3)	3506 (2968 to 4066)	0.5 (0.5 to 0.6)	23153 (19919 to 26400)	3.4 (3 to 3.9)	2500 (2104 to 2917)	0.4 (0.3 to 0.5)	63945 (54041 to 74443)	9.3 (7.8 to 10.8)	2284 (1599 to 3045)	0.3 (0.2 to 0.5)	66228 (56224 to 77023)	9.6 (8.1 to 11.2)
Low SDI	Both	24854 (20722 to 28759)	9.2 (7.7 to 10.8)	165833 (144562 to 188465)	61.1 (53.2 to 69.8)	19514 (16313 to 22898)	7.8 (6.5 to 9.3)	652513 (550780 to 754607)	223.7 (188 to 261.3)	13922 (9786 to 18843)	5.1 (3.6 to 6.8)	666435 (563641 to 771958)	228.7 (192.2 to 266.8)	81318 (70923 to 92857)	13.4 (11.8 to 15.1)	574998 (507286 to 647769)	92.4 (82.3 to 103.4)	54467 (47490 to 62057)	9.8 (8.6 to 11.1)	1790131 (1555486 to 2051605)	276.2 (240.8 to 316)	47250 (33428 to 63957)	7.6 (5.4 to 10.1)	1837382 (1599786 to 2101742)	283.8 (248.2 to 323.4)
	Female	23569 (19452 to 27524)	17.4 (14.2 to 20.5)	159609 (138159 to 182481)	118.1 (102 to 135.3)	18319 (15135 to 21683)	14.4 (11.8 to 17.4)	623105 (522154 to 724562)	428.3 (355.5 to 503)	13226 (9240 to 17927)	9.6 (6.7 to 13)	636330 (533366 to 739558)	437.9 (363.6 to 513.7)	79445 (69198 to 90893)	25.7 (22.5 to 29.1)	562126 (494493 to 635877)	177.7 (158.1 to 199.5)	52546 (45728 to 60015)	18.3 (16 to 20.8)	1743172 (1510816 to 2000181)	529.6 (461.8 to 607.4)	45927 (32241 to 62245)	14.5 (10.3 to 19.3)	1789099 (1554122 to 2051715)	544 (475.6 to 621.6)
	Male	1284 (1070 to 1506)	1.2 (1 to 1.4)	6224 (5288 to 7203)	5.4 (4.6 to 6.2)	1196 (992 to 1386)	1.3 (1 to 1.5)	29408 (24723 to 34194)	24.8 (20.7 to 28.8)	696 (471 to 935)	0.6 (0.4 to 0.8)	30104 (25344 to 35016)	25.5 (21.2 to 29.5)	1873 (1488 to 2452)	0.8 (0.6 to 1.1)	12872 (10519 to 16304)	5.2 (4.3 to 6.5)	1920 (1525 to 2525)	0.9 (0.7 to 1.2)	46959 (36996 to 62598)	18.2 (14.4 to 24.1)	1323 (907 to 1962)	0.5 (0.4 to 0.8)	48283 (38036 to 64317)	18.8 (14.9 to 24.7)
Middle SDI	Both	125965 (116279 to 136062)	10.7 (9.9 to 11.5)	1071229 (978418 to 1167494)	91.7 (83.3 to 100.8)	72640 (67619 to 78672)	6.7 (6.2 to 7.2)	2426282 (2252955 to 2631891)	195.7 (181.9 to 212.2)	79755 (55704 to 106840)	6.7 (4.7 to 8.9)	2506037 (2334448 to 2721721)	202.4 (188.6 to 218.9)	492444 (436591 to 552088)	18.5 (16.4 to 20.7)	4338486 (3886139 to 4807950)	162.3 (145.5 to 179.4)	184787 (166334 to 205342)	7.3 (6.6 to 8.1)	5601205 (5039739 to 6230665)	207.8 (187.1 to 230.7)	322153 (225480 to 441351)	12 (8.4 to 16.5)	5923359 (5318524 to 6558618)	219.9 (197.4 to 243.2)
	Female	124201 (114516 to 134278)	20.8 (19.3 to 22.5)	1059721 (967220 to 1155963)	179.3 (163.1 to 197)	71269 (66322 to 77262)	12.7 (11.8 to 13.7)	2390423 (2220434 to 2595136)	386.4 (358.4 to 419.1)	78642 (54924 to 105362)	13.1 (9.2 to 17.5)	2469065 (2297104 to 2681304)	399.5 (372 to 433.1)	485834 (430215 to 545187)	35.5 (31.5 to 39.8)	4284433 (3833491 to 4750888)	312.5 (279.6 to 346.6)	181116 (162719 to 201671)	13.7 (12.3 to 15.2)	5506431 (4944792 to 6125478)	399.8 (359.1 to 444.9)	316949 (221711 to 433539)	23.1 (16.2 to 31.6)	5823380 (5223617 to 6455655)	422.9 (378.6 to 468.9)

		1990												2019											
		Incidence		Prevalence		Deaths		YLLs (Years of Life Lost)		YLDs (Years Lived with Disability)		DALYs (Disability-Adjusted Life Years)		Incidence		Prevalence		Deaths		YLLs (Years of Life Lost)		YLDs (Years Lived with Disability)		DALYs (Disability-Adjusted Life Years)	
Location	Sex	Number	Rate	Number	Rate	Number	Rate	Number	Rate	Number	Rate	Number	Rate	Number	Rate	Number	Rate	Number	Rate	Number	Rate	Number	Rate	Number	Rate
	Male	1765 (1581 to 1957)	0.4 (0.3 to 0.4)	11508 (10249 to 12880)	2.2 (2 to 2.5)	1371 (1218 to 1528)	0.3 (0.3 to 0.4)	35859 (32212 to 39814)	6.9 (6.2 to 7.7)	1113 (785 to 1484)	0.2 (0.2 to 0.3)	36973 (33171 to 41010)	7.1 (6.4 to 7.9)	6611 (5511 to 7679)	0.5 (0.5 to 0.6)	54053 (45424 to 62785)	4.3 (3.7 to 5)	3670 (3126 to 4227)	0.3 (0.3 to 0.4)	94774 (80587 to 109525)	7.5 (6.4 to 8.7)	5205 (3586 to 7207)	0.4 (0.3 to 0.6)	99979 (84797 to 115476)	7.9 (6.7 to 9.1)
Countries																									
Afghanistan	Both	671 (524 to 858)	9 (7.1 to 11.5)	4836 (3961 to 5873)	64.8 (53.1 to 78.8)	526 (414 to 671)	7.3 (5.8 to 9.2)	17856 (13703 to 23102)	234.3 (180.6 to 301.6)	390 (260 to 567)	5.2 (3.5 to 7.5)	18245 (14012 to 23568)	239.5 (184.4 to 307.6)	1988 (1486 to 2606)	11.6 (8.8 to 15.1)	14052 (10896 to 17995)	79.6 (63.6 to 99.8)	1311 (987 to 1705)	8.7 (6.6 to 11.2)	48054 (35874 to 63721)	255.7 (192.3 to 334.9)	1157 (744 to 1685)	6.5 (4.3 to 9.2)	49211 (36828 to 65071)	262.3 (198.1 to 342.8)
	Female	644 (497 to 829)	17.1 (13.4 to 21.7)	4693 (3835 to 5734)	124.9 (102.8 to 151.6)	501 (389 to 642)	13.9 (11 to 17.5)	17181 (13140 to 22140)	437 (336 to 561.2)	374 (248 to 547)	9.8 (6.6 to 14.2)	17555 (13344 to 22678)	446.8 (342.1 to 573.5)	1951 (1451 to 2561)	22.3 (16.8 to 29.1)	13823 (10691 to 17817)	153.1 (121.8 to 192.8)	1281 (964 to 1673)	16.5 (12.5 to 21.3)	47124 (35205 to 62592)	493.7 (370.6 to 648.3)	1134 (729 to 1655)	12.5 (8.2 to 17.8)	48258 (36046 to 63895)	506.2 (380.9 to 663.4)
	Male	28 (13 to 52)	0.8 (0.4 to 1.4)	142 (77 to 252)	3.8 (2.2 to 6.5)	25 (13 to 47)	0.7 (0.4 to 1.3)	674 (317 to 1272)	17.7 (8.5 to 32.9)	16 (7 to 30)	0.4 (0.2 to 0.8)	690 (324 to 1301)	18.1 (8.7 to 33.6)	37 (21 to 61)	0.6 (0.3 to 0.9)	229 (144 to 361)	3.3 (2.2 to 4.9)	30 (17 to 49)	0.5 (0.3 to 0.8)	930 (510 to 1578)	12.1 (7.1 to 19.4)	23 (12 to 39)	0.3 (0.2 to 0.6)	953 (526 to 1615)	12.4 (7.4 to 19.9)
Albania	Both	226 (206 to 248)	9.5 (8.7 to 10.4)	2475 (2198 to 2777)	109.6 (96.2 to 125.1)	113 (104 to 123)	5.1 (4.7 to 5.6)	3639 (3351 to 3966)	148.5 (136.8 to 161.5)	165 (113 to 230)	7.1 (4.8 to 9.8)	3804 (3499 to 4148)	155.5 (143 to 169)	764 (555 to 1031)	20.7 (15.1 to 28)	7725 (6119 to 9889)	204.6 (160.1 to 264.8)	258 (190 to 343)	6.5 (4.8 to 8.7)	6838 (5042 to 9255)	185 (136.3 to 251.4)	540 (334 to 813)	14.6 (8.9 to 22.2)	7379 (5418 to 9965)	199.6 (145.6 to 271.4)
	Female	221 (201 to 243)	18.8 (17.1 to 20.7)	2431 (2156 to 2730)	213.1 (187.7 to 241.7)	110 (101 to 120)	9.7 (8.9 to 10.6)	3562 (3273 to 3887)	297.9 (274.5 to 325.2)	162 (110 to 225)	13.8 (9.4 to 19.2)	3724 (3423 to 4063)	311.7 (285.9 to 340.4)	743 (541 to 1004)	39 (28.3 to 52.7)	7554 (5984 to 9677)	386.9 (302.3 to 501.5)	249 (184 to 332)	11.9 (8.8 to 16)	6648 (4891 to 8996)	348.1 (256.3 to 474.2)	524 (323 to 789)	27.4 (16.7 to 41.6)	7172 (5262 to 9714)	375.5 (273.6 to 512.8)
	Male	5 (4 to 6)	0.5 (0.4 to 0.6)	44 (37 to 51)	4.2 (3.6 to 5)	3 (3 to 3)	0.3 (0.3 to 0.4)	77 (68 to 87)	7.1 (6.3 to 8.1)	4 (3 to 5)	0.4 (0.3 to 0.5)	81 (71 to 92)	7.5 (6.6 to 8.5)	21 (14 to 30)	1.1 (0.8 to 1.6)	172 (122 to 235)	8.9 (6.3 to 12.3)	9 (6 to 12)	0.5 (0.3 to 0.6)	191 (133 to 262)	10.1 (7.1 to 13.8)	16 (10 to 25)	0.8 (0.5 to 1.3)	207 (144 to 282)	11 (7.7 to 14.8)
Algeria	Both	1721 (1354 to 2167)	13.1 (10.5 to 16.3)	15452 (12685 to 18675)	113.5 (94.2 to 135)	981 (786 to 1221)	8.5 (6.9 to 10.3)	32219 (25422 to 40625)	232.2 (185.5 to 288.9)	1138 (740 to 1655)	8.4 (5.6 to 12)	33357 (26362 to 41954)	240.6 (192.6 to 299.6)	7052 (5307 to 8987)	18.1 (13.8 to 22.7)	63585 (49824 to 79009)	162.6 (129.7 to 199.5)	2662 (2013 to 3352)	7.6 (5.8 to 9.5)	84425 (62833 to 108274)	212.4 (159.8 to 269.9)	4730 (3071 to 6885)	12 (7.9 to 17.3)	89155 (67273 to 114020)	224.4 (170.3 to 285.4)
	Female	1526 (1186 to 1937)	22.1 (17.5 to 27.9)	14307 (11660 to 17462)	206.6 (171 to 249.7)	829 (657 to 1045)	13.2 (10.8 to 16.4)	28701 (22421 to 36735)	396.8 (311.9 to 505)	1024 (669 to 1513)	14.6 (9.6 to 21.2)	29725 (23246 to 37929)	411.4 (324.4 to 522.1)	6621 (4954 to 8490)	34 (25.6 to 43.3)	60497 (47142 to 75557)	311.4 (248.2 to 384.3)	2407 (1823 to 3047)	13.9 (10.7 to 17.3)	78779 (58625 to 101604)	396.6 (296.9 to 507)	4445 (2884 to 6539)	22.6 (14.8 to 32.8)	83225 (62174 to 107206)	419.3 (315.9 to 537.8)
	Male	195 (138 to 262)	4 (2.9 to 5.4)	1145 (841 to 1520)	19.9 (14.8 to 26.3)	152 (108 to 203)	3.6 (2.6 to 4.9)	3518 (2552 to 4770)	63.4 (45.4 to 84)	114 (69 to 172)	2.1 (1.3 to 3.1)	3632 (2629 to 4926)	65.5 (46.8 to 86.8)	430 (272 to 630)	2.7 (1.7 to 3.8)	3088 (2045 to 4388)	18.1 (12.2 to 25.6)	254 (166 to 364)	1.7 (1.1 to 2.5)	5646 (3677 to 7999)	32.9 (21.4 to 46.4)	285 (164 to 441)	1.7 (1 to 2.6)	5931 (3858 to 8439)	34.6 (22.5 to 48.9)
American Samoa	Both	4 (3 to 6)	16.8 (12.9 to 20.4)	39 (31 to 46)	142.5 (115.6 to 168.5)	3 (2 to 3)	11.5 (8.8 to 13.9)	84 (63 to 105)	296.6 (225.9 to 364.7)	3 (2 to 4)	10.3 (6.8 to 14.3)	87 (66 to 108)	306.8 (234.4 to 375.6)	14 (11 to 18)	29 (23.1 to 36.4)	118 (97 to 144)	230.7 (190.6 to 280.8)	8 (6 to 10)	16.8 (13.5 to 20.7)	225 (178 to 284)	441.4 (350.1 to 552.4)	9 (6 to 12)	17.5 (11.9 to 24.1)	234 (185 to 294)	458.9 (364 to 575.2)
	Female	4 (3 to 5)	34 (26 to 41.5)	38 (31 to 46)	289.9 (235 to 343.3)	3 (2 to 3)	22.8 (17.4 to 27.5)	84 (63 to 104)	607.2 (461.1 to 750.2)	3 (2 to 4)	20.9 (13.8 to 29.3)	86 (65 to 108)	628.1 (478.7 to 772.3)	14 (11 to 18)	56 (44.3 to 70.3)	117 (96 to 144)	448.3 (369.3 to 546.8)	8 (6 to 10)	31.8 (25.5 to 39.3)	224 (177 to 283)	856.9 (677.8 to 1074.2)	9 (6 to 12)	34 (22.9 to 46.9)	233 (184 to 293)	890.8 (704.6 to 1117.5)
	Male	0 (0 to 0)	0.2 (0.1 to 0.2)	0 (0 to 0)	1.3 (1 to 1.7)	0 (0 to 0)	0.1 (0.1 to 0.2)	0 (0 to 1)	2.8 (1.9 to 4)	0 (0 to 0)	0.1 (0.1 to 0.2)	0 (0 to 1)	2.9 (2 to 4.1)	0 (0 to 0)	0.3 (0.2 to 0.4)	0 (0 to 1)	2 (1.5 to 2.5)	0 (0 to 0)	0.2 (0.1 to 0.3)	1 (1 to 1)	4.3 (3.1 to 5.7)	0 (0 to 0)	0.2 (0.1 to 0.3)	1 (1 to 1)	4.4 (3.3 to 5.9)
Andorra	Both	18 (13 to 25)	31.2 (23.1 to 42.9)	201 (159 to 257)	350.7 (278.3 to 446.9)	5 (4 to 8)	10.4 (7.8 to 14.2)	150 (111 to 206)	257.9 (190.8 to 354.7)	14 (9 to 21)	24.2 (15.4 to 35.8)	164 (121 to 225)	282.1 (209.1 to 386.8)	55 (39 to 74)	40.5 (28.7 to 54.1)	610 (480 to 764)	444.8 (348.4 to 558)	13 (9 to 17)	9 (6.5 to 12)	306 (221 to 413)	223.2 (161.8 to 300.8)	42 (27 to 63)	31.2 (19.5 to 46.4)	348 (250 to 473)	254.5 (183.1 to 345.1)
	Female	17 (13 to 24)	62.8 (46.2 to 86.8)	196 (155 to 251)	709.4 (563.2 to 908.4)	5 (4 to 7)	20 (14.8 to 27.6)	145 (106 to 199)	520.8 (384.6 to 716)	14 (9 to 20)	48.7 (30.9 to 73)	158 (116 to 217)	569.5 (420 to 785)	54 (38 to 72)	80.6 (56.7 to 107.9)	595 (467 to 746)	890.8 (689.5 to 1119.7)	13 (9 to 17)	17.3 (12.4 to 23.1)	296 (213 to 399)	443.1 (318.4 to 599.5)	41 (26 to 61)	62.2 (38.6 to 92.3)	337 (241 to 456)	505.4 (361.6 to 687.1)
	Male	1 (0 to 1)	2.2 (1.4 to 3.3)	5 (3 to 7)	17.8 (12.2 to 25.1)	0 (0 to 0)	0.9 (0.6 to 1.4)	5 (4 to 8)	18.9 (12.6 to 27.8)	0 (0 to 1)	1.7 (1 to 2.7)	6 (4 to 9)	20.5 (13.7 to 30.5)	2 (1 to 2)	2.4 (1.7 to 3.4)	15 (11 to 20)	21.4 (15.8 to 28.8)	1 (0 to 1)	0.7 (0.5 to 1)	10 (7 to 14)	14.5 (10.2 to 20)	1 (1 to 2)	1.9 (1.1 to 2.9)	11 (8 to 16)	16.4 (11.5 to 22.5)

		1990												2019											
		Incidence		Prevalence		Deaths		YLLs (Years of Life Lost)		YLDs (Years Lived with Disability)		DALYs (Disability-Adjusted Life Years)		Incidence		Prevalence		Deaths		YLLs (Years of Life Lost)		YLDs (Years Lived with Disability)		DALYs (Disability-Adjusted Life Years)	
Location	Sex	Number	Rate	Number	Rate	Number	Rate	Number	Rate	Number	Rate	Number	Rate	Number	Rate	Number	Rate	Number	Rate	Number	Rate	Number	Rate	Number	Rate
Angola	Both	396 (274 to 530)	8.5 (5.9 to 11.2)	2576 (1916 to 3310)	54.3 (42 to 67.8)	319 (225 to 431)	7.4 (5.4 to 9.8)	11145 (7739 to 15386)	211.2 (148.9 to 286.2)	221 (137 to 325)	4.6 (2.9 to 6.7)	11366 (7919 to 15669)	215.8 (152.7 to 291.6)	1978 (1408 to 2660)	14.9 (11.1 to 19.7)	13491 (9883 to 17787)	95.3 (72.9 to 122.5)	1338 (967 to 1790)	11.5 (8.8 to 15)	44861 (31503 to 60996)	302 (219.1 to 403.5)	1136 (696 to 1727)	8.1 (5.3 to 12.1)	45997 (32317 to 62266)	310.1 (224.2 to 414.8)
	Female	383 (263 to 520)	16.2 (11.1 to 21.6)	2508 (1861 to 3250)	104.9 (80.8 to 131.2)	308 (214 to 417)	14 (9.9 to 18.6)	10793 (7407 to 14884)	410.2 (285.4 to 559.1)	213 (132 to 316)	8.8 (5.6 to 12.9)	11007 (7571 to 15180)	419 (292.5 to 570.5)	1940 (1376 to 2621)	26.4 (19.5 to 35.2)	13269 (9675 to 17539)	171.6 (129.8 to 222.7)	1308 (944 to 1762)	20 (15 to 26.3)	43962 (30889 to 59899)	543.6 (391.9 to 733)	1113 (678 to 1689)	14.5 (9.3 to 21.7)	45075 (31524 to 61099)	558.1 (402.5 to 751.5)
	Male	13 (8 to 20)	0.6 (0.4 to 0.9)	68 (44 to 100)	3.2 (2.2 to 4.5)	11 (7 to 18)	0.6 (0.4 to 0.9)	352 (210 to 555)	15.2 (9.2 to 23.4)	8 (4 to 12)	0.4 (0.2 to 0.6)	359 (215 to 566)	15.6 (9.4 to 23.9)	37 (24 to 55)	0.7 (0.5 to 1)	221 (148 to 321)	3.9 (2.7 to 5.4)	30 (19 to 43)	0.6 (0.4 to 0.9)	899 (569 to 1381)	14.8 (9.5 to 21.5)	23 (13 to 38)	0.4 (0.3 to 0.7)	923 (583 to 1418)	15.2 (9.8 to 22.1)
Antigua and Barbuda	Both	14 (13 to 16)	28.2 (24.8 to 31.9)	119 (106 to 133)	233.7 (207.7 to 262.1)	7 (6 to 8)	13.4 (11.9 to 15)	180 (160 to 203)	357.7 (318.8 to 403.8)	9 (6 to 11)	16.8 (11.8 to 22.6)	188 (168 to 212)	374.4 (333.7 to 422.8)	45 (38 to 54)	42.8 (36.4 to 50.3)	373 (317 to 439)	347.8 (297.5 to 407.9)	16 (14 to 19)	16.4 (14.3 to 18.9)	435 (368 to 515)	404.8 (344.2 to 476.7)	27 (19 to 37)	25.3 (17.7 to 34.6)	462 (391 to 547)	430.1 (366.2 to 506.4)
	Female	14 (13 to 16)	51 (44.6 to 57.8)	118 (105 to 132)	425.9 (377.7 to 479.5)	7 (6 to 8)	23.2 (20.7 to 26.2)	177 (158 to 200)	651.9 (578.4 to 737.2)	8 (6 to 11)	30.4 (21.2 to 41)	186 (166 to 209)	682.3 (606.8 to 774.1)	45 (38 to 53)	80.4 (68.3 to 94.9)	371 (315 to 437)	658.1 (561.9 to 773)	16 (14 to 19)	30.2 (26.1 to 34.8)	431 (364 to 511)	761.5 (645.6 to 897.8)	27 (18 to 37)	47.7 (33.2 to 65.3)	458 (387 to 542)	809.2 (688.3 to 952.9)
	Male	0 (0 to 0)	0.7 (0.6 to 0.9)	1 (1 to 1)	4.2 (3.5 to 5.1)	0 (0 to 0)	0.5 (0.5 to 0.7)	2 (2 to 3)	11.1 (9.3 to 13.2)	0 (0 to 0)	0.4 (0.3 to 0.6)	3 (2 to 3)	11.5 (9.7 to 13.7)	0 (0 to 0)	0.7 (0.5 to 0.9)	2 (2 to 3)	4.3 (3.3 to 5.5)	0 (0 to 0)	0.4 (0.3 to 0.6)	4 (3 to 5)	8.3 (6.5 to 10.6)	0 (0 to 0)	0.4 (0.3 to 0.6)	4 (3 to 5)	8.8 (6.8 to 11.1)
Argentina	Both	8956 (8549 to 9406)	28 (26.7 to 29.3)	80528 (73833 to 88830)	250.7 (229.6 to 276.7)	5473 (5243 to 5698)	17.4 (16.6 to 18.1)	144076 (139103 to 149395)	447.6 (431.7 to 464.2)	5976 (4227 to 8130)	18.6 (13.1 to 25.3)	150052 (144620 to 155832)	466.2 (449.4 to 483.9)	17868 (13834 to 22719)	34.3 (26.5 to 43.7)	165358 (136541 to 200357)	316.8 (260.4 to 385.6)	8476 (7818 to 9135)	15.8 (14.6 to 17.1)	197317 (184161 to 210825)	382.5 (356.5 to 408.6)	12336 (8264 to 17751)	23.7 (15.9 to 34.2)	209653 (194978 to 225664)	406.2 (377.1 to 437.3)
	Female	8879 (8475 to 9329)	51 (48.7 to 53.5)	79995 (73287 to 88299)	457.9 (420.4 to 503.7)	5416 (5187 to 5643)	30.9 (29.6 to 32.2)	142766 (137833 to 148109)	828.7 (799.4 to 859.8)	5922 (4187 to 8062)	34 (24 to 46.3)	148688 (143323 to 154524)	862.7 (831.1 to 895.8)	17745 (13747 to 22569)	62.7 (48.4 to 80.3)	164398 (135787 to 199062)	580.8 (475.8 to 709.2)	8403 (7742 to 9066)	27.9 (25.8 to 30)	195718 (182508 to 209212)	705.5 (657.4 to 753.6)	12243 (8208 to 17627)	43.5 (29.1 to 62.7)	207961 (193317 to 223994)	749 (694.5 to 808.1)
	Male	78 (68 to 88)	0.6 (0.5 to 0.6)	533 (460 to 617)	3.8 (3.3 to 4.3)	57 (50 to 64)	0.4 (0.4 to 0.5)	1310 (1165 to 1465)	9.1 (8.1 to 10.2)	55 (37 to 76)	0.4 (0.3 to 0.5)	1364 (1213 to 1523)	9.5 (8.5 to 10.6)	123 (88 to 165)	0.5 (0.4 to 0.7)	961 (727 to 1251)	4.1 (3.1 to 5.3)	73 (60 to 89)	0.3 (0.3 to 0.4)	1599 (1342 to 1903)	6.8 (5.7 to 8.1)	93 (59 to 140)	0.4 (0.3 to 0.6)	1692 (1420 to 2021)	7.2 (6 to 8.6)
Armenia	Both	926 (865 to 991)	31.4 (29.5 to 33.6)	7956 (7342 to 8700)	272.3 (251 to 297.8)	470 (444 to 497)	16.6 (15.6 to 17.5)	15122 (14233 to 16057)	501.6 (473.1 to 530)	597 (418 to 805)	20.3 (14.2 to 27.3)	15719 (14739 to 16683)	521.8 (491.8 to 551.8)	1363 (1116 to 1641)	34.2 (27.9 to 41.2)	13254 (11316 to 15408)	329.4 (280.1 to 383.6)	558 (465 to 662)	13.8 (11.4 to 16.4)	14712 (12172 to 17746)	369.2 (303.6 to 446.1)	934 (632 to 1291)	23.4 (15.8 to 32.3)	15646 (12961 to 18825)	392.6 (321.8 to 472.4)
	Female	926 (864 to 990)	57.5 (53.8 to 61.4)	7947 (7333 to 8690)	495.4 (458.5 to 541.7)	470 (443 to 497)	29.6 (28 to 31.3)	15108 (14218 to 16045)	926.8 (874.1 to 981.5)	596 (417 to 804)	37 (25.9 to 50.1)	15704 (14724 to 16668)	963.8 (907.6 to 1021.5)	1350 (1105 to 1627)	61.2 (49.8 to 73.7)	13163 (11223 to 15288)	589.6 (499.9 to 686.9)	552 (459 to 654)	23.9 (19.9 to 28.5)	14566 (12035 to 17578)	662.6 (543.4 to 802.5)	925 (625 to 1280)	41.8 (28.2 to 57.8)	15491 (12803 to 18634)	704.5 (576.7 to 849.3)
	Male	1 (1 to 1)	0.1 (0.1 to 0.1)	9 (7 to 11)	0.7 (0.6 to 0.8)	0 (0 to 1)	0 (0 to 0)	14 (13 to 16)	1.1 (0.9 to 1.2)	1 (0 to 1)	0.1 (0 to 0.1)	15 (14 to 17)	1.1 (1 to 1.2)	13 (10 to 17)	0.7 (0.6 to 0.9)	91 (68 to 118)	5.1 (3.9 to 6.6)	6 (5 to 8)	0.4 (0.3 to 0.4)	146 (115 to 178)	8.2 (6.5 to 9.9)	9 (6 to 14)	0.5 (0.3 to 0.8)	155 (122 to 191)	8.7 (6.9 to 10.6)
Australia	Both	7976 (7587 to 8331)	42.1 (40.1 to 44)	87961 (80900 to 97287)	461.2 (426 to 508.6)	2605 (2471 to 2701)	13.8 (13.1 to 14.4)	67637 (65366 to 69715)	360.8 (348.4 to 372)	6113 (4247 to 8339)	32.1 (22.3 to 43.9)	73750 (70513 to 76985)	392.9 (375.9 to 409.8)	15656 (12089 to 20129)	42.6 (32.9 to 55.1)	176676 (147339 to 213221)	468.9 (388.1 to 571.8)	3711 (3324 to 4031)	9.1 (8.3 to 9.9)	80925 (74741 to 86669)	223.9 (207.7 to 239.3)	12080 (8010 to 17498)	32.7 (21.5 to 47.6)	93004 (85086 to 102208)	256.5 (235.5 to 281.6)
	Female	7932 (7545 to 8284)	79.7 (75.9 to 83.3)	87548 (80530 to 96823)	864.7 (800.1 to 949)	2593 (2459 to 2690)	25.3 (24.1 to 26.2)	67362 (65090 to 69433)	695.4 (671.8 to 717.1)	6075 (4213 to 8294)	60.6 (41.9 to 82.7)	73436 (70207 to 76662)	756 (723.8 to 788.3)	15545 (12011 to 20007)	81.5 (62.8 to 105.5)	175627 (146353 to 211663)	896.1 (740.6 to 1095.4)	3687 (3306 to 4008)	17.1 (15.6 to 18.4)	80436 (74321 to 86180)	429.7 (398.8 to 459.6)	11986 (7946 to 17362)	62.5 (40.9 to 91.1)	92423 (84549 to 101565)	492.1 (451.7 to 540.6)
	Male	45 (37 to 53)	0.5 (0.4 to 0.6)	413 (349 to 485)	4.7 (4 to 5.5)	12 (10 to 13)	0.1 (0.1 to 0.2)	275 (244 to 308)	3.1 (2.8 to 3.5)	38 (26 to 54)	0.4 (0.3 to 0.6)	314 (277 to 354)	3.6 (3.1 to 4)	110 (78 to 150)	0.6 (0.4 to 0.8)	1049 (788 to 1395)	5.9 (4.4 to 7.8)	24 (19 to 30)	0.1 (0.1 to 0.2)	488 (405 to 590)	2.7 (2.3 to 3.3)	93 (59 to 143)	0.5 (0.3 to 0.8)	581 (477 to 718)	3.3 (2.7 to 4)

		1990												2019											
		Incidence		Prevalence		Deaths		YLLs (Years of Life Lost)		YLDs (Years Lived with Disability)		DALYs (Disability-Adjusted Life Years)		Incidence		Prevalence		Deaths		YLLs (Years of Life Lost)		YLDs (Years Lived with Disability)		DALYs (Disability-Adjusted Life Years)	
Location	Sex	Number	Rate	Number	Rate	Number	Rate	Number	Rate	Number	Rate	Number	Rate	Number	Rate	Number	Rate	Number	Rate	Number	Rate	Number	Rate	Number	Rate
Austria	Both	4589 (4283 to 4898)	43.3 (40.5 to 46.2)	50498 (45674 to 56180)	466.2 (424.4 to 513.2)	1709 (1611 to 1778)	15.2 (14.3 to 15.7)	39826 (38130 to 41218)	381.9 (366.7 to 395.3)	3491 (2442 to 4756)	32.7 (22.7 to 44.8)	43317 (41295 to 45264)	414.6 (395.2 to 433.5)	5698 (4584 to 6999)	37.1 (29.8 to 45.6)	63235 (54588 to 74020)	403.2 (344.3 to 474.9)	1776 (1598 to 1927)	9.8 (9 to 10.6)	34292 (31626 to 36768)	222.6 (207 to 238.2)	4339 (2961 to 6062)	28.2 (19 to 40)	38632 (35426 to 41911)	250.8 (230.5 to 271.3)
	Female	4565 (4259 to 4876)	77.9 (72.5 to 83.3)	50269 (45443 to 55905)	824.4 (753.7 to 904.8)	1698 (1600 to 1768)	25.6 (24.3 to 26.6)	39591 (37883 to 40980)	692 (664.9 to 717.5)	3471 (2428 to 4730)	58.4 (40.5 to 80.1)	43062 (41053 to 44992)	750.4 (716.1 to 785.5)	5635 (4529 to 6920)	69.9 (55.9 to 86.1)	62651 (54108 to 73392)	758.2 (644.4 to 893.4)	1755 (1578 to 1909)	17.6 (16.1 to 18.9)	33901 (31272 to 36374)	419.2 (390.1 to 448.8)	4286 (2930 to 5992)	53.1 (35.6 to 75.6)	38187 (35017 to 41385)	472.3 (433.1 to 512.2)
	Male	24 (20 to 29)	0.5 (0.5 to 0.7)	229 (192 to 272)	5.2 (4.3 to 6.1)	11 (9 to 12)	0.2 (0.2 to 0.3)	235 (204 to 268)	5.2 (4.6 to 6)	20 (13 to 29)	0.5 (0.3 to 0.6)	255 (223 to 292)	5.7 (5 to 6.5)	63 (46 to 85)	0.8 (0.6 to 1.1)	584 (450 to 752)	7.7 (6 to 9.9)	21 (17 to 26)	0.3 (0.2 to 0.3)	392 (324 to 477)	5.3 (4.4 to 6.3)	53 (33 to 78)	0.7 (0.4 to 1)	445 (366 to 545)	6 (5 to 7.3)
Azerbaijan	Both	952 (859 to 1056)	17.5 (15.9 to 19.5)	8357 (7492 to 9324)	156.3 (140.3 to 174.5)	548 (496 to 605)	10.5 (9.5 to 11.6)	17952 (16217 to 19788)	321.4 (290.7 to 355)	616 (419 to 861)	11.4 (7.7 to 15.7)	18569 (16765 to 20623)	332.8 (301 to 367.8)	2300 (1834 to 2862)	20.8 (16.6 to 25.7)	20125 (16619 to 24203)	181.4 (151.6 to 215.3)	993 (792 to 1234)	9.8 (7.9 to 12.1)	32022 (25323 to 39961)	281.5 (224.6 to 348)	1510 (1001 to 2182)	13.5 (9.2 to 19.3)	33531 (26534 to 41824)	295.1 (235.9 to 364.2)
	Female	951 (857 to 1054)	31.6 (28.4 to 35)	8342 (7475 to 9311)	277.6 (249.8 to 309.4)	547 (495 to 604)	18.3 (16.6 to 20.2)	17924 (16191 to 19761)	587.1 (531.8 to 648.4)	615 (418 to 859)	20.4 (13.8 to 28.3)	18539 (16736 to 20593)	607.4 (548.6 to 671.7)	2287 (1823 to 2841)	38.5 (30.7 to 47.6)	20025 (16496 to 24108)	336.6 (280 to 401.1)	986 (785 to 1225)	17.8 (14.2 to 22)	31825 (25101 to 39773)	525.1 (418 to 649.6)	1500 (994 to 2169)	25.2 (17 to 35.8)	33325 (26340 to 41639)	550.2 (438.6 to 682.2)
	Male	1 (1 to 2)	0.1 (0 to 0.1)	15 (12 to 19)	0.6 (0.5 to 0.8)	1 (1 to 1)	0 (0 to 0)	28 (24 to 34)	1.1 (0.9 to 1.3)	1 (1 to 2)	0 (0 to 0.1)	29 (25 to 35)	1.1 (0.9 to 1.3)	13 (8 to 20)	0.3 (0.2 to 0.4)	100 (63 to 149)	2.1 (1.4 to 3.1)	7 (4 to 10)	0.2 (0.1 to 0.2)	197 (120 to 294)	4.1 (2.6 to 5.9)	10 (5 to 16)	0.2 (0.1 to 0.3)	207 (127 to 308)	4.3 (2.8 to 6.2)
Bahamas	Both	75 (67 to 84)	42.3 (37.9 to 47.4)	598 (532 to 668)	335.2 (298.8 to 373.8)	33 (30 to 37)	20.2 (18.2 to 22.4)	1071 (954 to 1195)	579.6 (516.6 to 643.9)	44 (30 to 60)	24.8 (17.1 to 33.6)	1116 (997 to 1251)	604.5 (540 to 676.4)	211 (169 to 263)	49.9 (40.2 to 62)	1760 (1444 to 2175)	414.2 (342 to 507.2)	80 (65 to 99)	20 (16.4 to 24.6)	2397 (1921 to 3008)	556.7 (448.7 to 698.4)	127 (84 to 182)	30 (20 to 42.5)	2524 (2015 to 3160)	586.7 (473.2 to 732.6)
	Female	74 (66 to 83)	77 (68.8 to 86.4)	594 (529 to 664)	614 (547.4 to 686.8)	33 (29 to 36)	35.6 (32 to 39.4)	1059 (942 to 1182)	1064.5 (945.3 to 1186.9)	44 (30 to 60)	45.3 (31.2 to 61.5)	1103 (984 to 1237)	1109.8 (988.5 to 1243.3)	209 (167 to 261)	92.1 (74.1 to 114.9)	1749 (1434 to 2161)	768.4 (633.3 to 942.6)	79 (65 to 97)	35.9 (29.5 to 44.4)	2370 (1898 to 2974)	1031.7 (829.7 to 1294.7)	126 (83 to 180)	55.5 (36.9 to 78.7)	2496 (1991 to 3131)	1087.2 (874 to 1359.3)
	Male	1 (1 to 1)	1 (0.9 to 1.2)	4 (4 to 5)	6 (5.1 to 7.1)	0 (0 to 1)	0.8 (0.7 to 0.9)	13 (11 to 14)	17.4 (15.1 to 19.8)	0 (0 to 1)	0.6 (0.4 to 0.9)	13 (11 to 15)	18 (15.6 to 20.5)	2 (1 to 2)	1 (0.8 to 1.2)	11 (9 to 15)	6.2 (4.9 to 7.9)	1 (1 to 1)	0.7 (0.5 to 0.8)	27 (21 to 34)	14.3 (11.3 to 18.1)	1 (1 to 2)	0.6 (0.4 to 0.9)	28 (21 to 36)	15 (11.8 to 18.9)
Bahrain	Both	47 (39 to 56)	19.8 (16.9 to 23.2)	390 (331 to 456)	160.8 (139.2 to 186.8)	25 (21 to 29)	12.5 (10.7 to 14.4)	834 (702 to 984)	320.9 (272.8 to 376.7)	30 (20 to 42)	12.1 (8.5 to 16.6)	864 (725 to 1018)	333 (283.4 to 390.3)	349 (273 to 437)	26.2 (21.1 to 31.9)	3115 (2527 to 3807)	226 (190.3 to 268.4)	104 (83 to 128)	10.6 (8.7 to 12.8)	3393 (2700 to 4225)	236.6 (190.1 to 289.1)	231 (153 to 334)	16.8 (11.6 to 23.5)	3625 (2883 to 4525)	253.4 (203 to 311)
	Female	47 (39 to 56)	45.8 (38.9 to 54)	388 (329 to 453)	373.4 (322.9 to 433.4)	24 (21 to 29)	27.4 (23.5 to 31.8)	830 (698 to 979)	760.4 (646.3 to 891.3)	30 (20 to 42)	28.2 (19.5 to 38.9)	859 (721 to 1013)	788.6 (671 to 925.1)	346 (271 to 433)	67.5 (54 to 83.1)	3093 (2507 to 3783)	591.5 (492.6 to 708.6)	103 (82 to 127)	25.2 (20.4 to 30.5)	3362 (2676 to 4186)	624.6 (500.5 to 769)	229 (151 to 331)	43.7 (29.7 to 61.3)	3592 (2857 to 4487)	668.3 (533.3 to 827.7)
	Male	0 (0 to 0)	0.2 (0.1 to 0.3)	2 (2 to 3)	1.8 (1.4 to 2.3)	0 (0 to 0)	0.1 (0.1 to 0.2)	4 (3 to 5)	3.2 (2.3 to 4.4)	0 (0 to 0)	0.1 (0.1 to 0.2)	4 (3 to 6)	3.4 (2.4 to 4.6)	3 (2 to 4)	0.5 (0.3 to 0.7)	23 (16 to 31)	3.7 (2.5 to 5.2)	1 (1 to 2)	0.2 (0.1 to 0.3)	31 (20 to 44)	4.7 (3 to 6.8)	2 (1 to 3)	0.3 (0.2 to 0.6)	33 (21 to 47)	5.1 (3.2 to 7.3)
Bangladesh	Both	5046 (3753 to 6524)	8.8 (6.7 to 11.3)	35026 (26969 to 43566)	61.8 (49 to 75.3)	3738 (2804 to 4777)	6.9 (5.3 to 8.7)	135128 (98142 to 176055)	223.1 (165.9 to 287.5)	2881 (1802 to 4150)	5 (3.2 to 7.1)	138009 (100436 to 179687)	228.1 (169.1 to 294.1)	17812 (13804 to 22827)	12.5 (9.7 to 15.9)	140999 (113756 to 176102)	98.6 (79.9 to 122.5)	9884 (7761 to 12395)	7.2 (5.7 to 9)	316977 (246020 to 401809)	218.4 (170.3 to 276.4)	10903 (7258 to 15829)	7.6 (5.1 to 11)	327880 (254782 to 416587)	226 (176.3 to 286.6)
	Female	4952 (3670 to 6426)	18.9 (14.3 to 24.2)	34600 (26557 to 43066)	133.8 (105.5 to 163.4)	3650 (2726 to 4693)	14.7 (11.1 to 18.7)	133070 (96464 to 174324)	477.3 (353.7 to 615.7)	2829 (1762 to 4082)	10.6 (6.7 to 15.3)	135899 (98709 to 177807)	487.9 (360.4 to 630.9)	17661 (13693 to 22672)	25 (19.6 to 31.8)	140095 (113068 to 174878)	198.2 (160.9 to 245)	9771 (7661 to 12280)	14.5 (11.5 to 18.1)	314287 (243368 to 399058)	435.1 (339.9 to 550.3)	10807 (7200 to 15671)	15.2 (10.1 to 22.1)	325094 (252941 to 413603)	450.2 (352.4 to 570.3)
	Male	94 (64 to 136)	0.4 (0.3 to 0.6)	427 (297 to 612)	1.6 (1.1 to 2.3)	88 (59 to 127)	0.4 (0.3 to 0.6)	2058 (1401 to 2985)	7.9 (5.4 to 11.4)	52 (31 to 81)	0.2 (0.1 to 0.3)	2110 (1433 to 3062)	8.1 (5.5 to 11.7)	151 (87 to 249)	0.2 (0.1 to 0.4)	904 (551 to 1476)	1.3 (0.8 to 2.1)	113 (65 to 186)	0.2 (0.1 to 0.3)	2690 (1551 to 4419)	3.9 (2.3 to 6.4)	96 (51 to 172)	0.1 (0.1 to 0.3)	2786 (1604 to 4560)	4 (2.4 to 6.6)
Barbados	Both	102 (92 to 112)	39.2 (35.4 to 43.1)	819 (738 to 900)	316.6 (285.3 to 348)	49 (45 to 53)	18 (16.5 to 19.6)	1210 (1108 to 1321)	478.9 (437.3 to 525)	60 (42 to 80)	23.2 (16 to 30.8)	1270 (1161 to 1383)	502.1 (457 to 548.3)	254 (206 to 305)	55.5 (44.9 to 66.6)	2098 (1734 to 2491)	458.4 (375.6 to 546.8)	95 (78 to 112)	20 (16.4 to 23.7)	2279 (1826 to 2723)	507.5 (406 to 609.2)	152 (105 to 211)	33.2 (22.8 to 46.6)	2431 (1964 to 2905)	540.7 (434.1 to 649.4)

		1990												2019											
		Incidence		Prevalence		Deaths		YLLs (Years of Life Lost)		YLDs (Years Lived with Disability)		DALYs (Disability-Adjusted Life Years)		Incidence		Prevalence		Deaths		YLLs (Years of Life Lost)		YLDs (Years Lived with Disability)		DALYs (Disability-Adjusted Life Years)	
Location	Sex	Number	Rate	Number	Rate	Number	Rate	Number	Rate	Number	Rate	Number	Rate	Number	Rate	Number	Rate	Number	Rate	Number	Rate	Number	Rate	Number	Rate
	Female	101 (91 to 111)	69.7 (62.7 to 76.7)	813 (732 to 895)	566.7 (509.7 to 622.5)	48 (44 to 53)	31 (28.3 to 33.7)	1196 (1093 to 1308)	858.5 (782.4 to 942.9)	60 (41 to 80)	41.3 (28.6 to 55)	1256 (1147 to 1370)	899.9 (816.3 to 984.6)	252 (204 to 303)	102.3 (82.6 to 123)	2086 (1723 to 2477)	849.6 (694.6 to 1014.5)	94 (77 to 111)	36.1 (29.5 to 42.7)	2256 (1809 to 2693)	938.4 (749.5 to 1129)	150 (104 to 209)	61.4 (42.1 to 86.2)	2407 (1944 to 2873)	999.7 (800 to 1202.9)
	Male	1 (1 to 1)	0.8 (0.7 to 0.9)	5 (5 to 6)	4.6 (3.9 to 5.4)	1 (1 to 1)	0.6 (0.5 to 0.6)	13 (12 to 15)	11.6 (10.3 to 13)	1 (0 to 1)	0.5 (0.3 to 0.6)	14 (12 to 16)	12 (10.6 to 13.5)	2 (1 to 2)	0.9 (0.7 to 1.1)	12 (10 to 16)	5.7 (4.4 to 7.4)	1 (1 to 1)	0.6 (0.4 to 0.7)	23 (18 to 29)	10.7 (8.4 to 13.5)	1 (1 to 2)	0.6 (0.4 to 0.8)	24 (19 to 30)	11.2 (8.8 to 14.2)
Belarus	Both	3142 (2935 to 3342)	25.3 (23.6 to 26.8)	31443 (28696 to 34806)	250.5 (228.5 to 275.9)	1376 (1314 to 1447)	10.9 (10.4 to 11.4)	40643 (38877 to 42593)	327.2 (312.8 to 343.2)	2180 (1530 to 2992)	17.5 (12.2 to 24)	42824 (40820 to 44890)	344.7 (328.5 to 361.2)	4133 (3140 to 5455)	28 (21.2 to 37)	41067 (33282 to 51330)	274.6 (220 to 345.9)	1297 (1006 to 1668)	8.4 (6.5 to 10.8)	33305 (25281 to 43732)	226.7 (171.3 to 296.9)	2901 (1859 to 4261)	19.6 (12.6 to 28.8)	36206 (27659 to 47348)	246.2 (187.5 to 324.4)
	Female	3138 (2931 to 3338)	44.2 (41.2 to 47)	31406 (28659 to 34772)	429.5 (393.5 to 469.2)	1373 (1311 to 1444)	18.3 (17.5 to 19.2)	40580 (38818 to 42524)	580.7 (554.4 to 608.6)	2177 (1527 to 2987)	30.4 (21.1 to 41.8)	42756 (40752 to 44828)	611.1 (581.6 to 641.7)	4115 (3126 to 5431)	48.9 (36.9 to 64.8)	40934 (33169 to 51140)	475.9 (379.2 to 604.9)	1291 (1000 to 1660)	14 (10.8 to 18.1)	33141 (25170 to 43554)	399.4 (301.1 to 526.6)	2887 (1848 to 4243)	34.1 (21.8 to 50.4)	36028 (27520 to 47158)	433.5 (329.2 to 573.1)
	Male	5 (4 to 6)	0.1 (0.1 to 0.1)	37 (31 to 45)	0.8 (0.6 to 0.9)	2 (2 to 3)	0.1 (0 to 0.1)	63 (55 to 73)	1.3 (1.1 to 1.5)	4 (2 to 5)	0.1 (0.1 to 0.1)	67 (59 to 77)	1.4 (1.2 to 1.6)	18 (12 to 26)	0.3 (0.2 to 0.4)	134 (92 to 192)	2.2 (1.6 to 3.1)	6 (5 to 9)	0.1 (0.1 to 0.2)	164 (117 to 228)	2.7 (2 to 3.7)	14 (8 to 22)	0.2 (0.1 to 0.4)	178 (126 to 248)	3 (2.1 to 4)
Belgium	Both	7605 (7096 to 8119)	54.9 (51.3 to 58.7)	78846 (71784 to 86351)	559.3 (512.8 to 609.7)	2964 (2785 to 3099)	20.3 (19.2 to 21.2)	69243 (66386 to 71907)	508.5 (489 to 527.2)	5601 (3907 to 7654)	40.2 (28 to 54.9)	74844 (71334 to 78136)	548.7 (524.4 to 572)	9697 (7576 to 12408)	49.9 (38.7 to 64.5)	107879 (90955 to 129780)	542.6 (450.8 to 662)	2919 (2586 to 3184)	12.7 (11.5 to 13.7)	56394 (52101 to 60765)	291.7 (272.4 to 313.3)	7374 (4883 to 10603)	37.9 (24.8 to 54.9)	63769 (58318 to 70252)	329.6 (302.9 to 362.1)
	Female	7570 (7063 to 8082)	102 (95.1 to 109.3)	78523 (71451 to 86008)	1031.2 (947 to 1120.5)	2948 (2770 to 3083)	35.7 (33.9 to 37.2)	68912 (66042 to 71574)	954.9 (919.6 to 989.7)	5572 (3886 to 7620)	74.6 (51.8 to 102.1)	74484 (71028 to 77757)	1029.5 (984.6 to 1073.3)	9625 (7524 to 12320)	95.2 (73.9 to 123.6)	107213 (90363 to 128917)	1034 (855.8 to 1267.1)	2896 (2566 to 3163)	23.1 (21.1 to 24.9)	55956 (51715 to 60285)	557.5 (520.8 to 599.1)	7315 (4843 to 10484)	72.3 (47.2 to 104.9)	63270 (57849 to 69765)	629.8 (579.8 to 693.2)
	Male	35 (28 to 42)	0.5 (0.4 to 0.7)	323 (268 to 395)	5.1 (4.3 to 6.2)	16 (13 to 18)	0.3 (0.2 to 0.3)	331 (286 to 382)	5.2 (4.5 to 6)	29 (20 to 42)	0.5 (0.3 to 0.7)	360 (311 to 414)	5.7 (4.9 to 6.5)	71 (50 to 102)	0.7 (0.5 to 1)	666 (497 to 895)	6.8 (5.1 to 9.2)	24 (19 to 29)	0.2 (0.2 to 0.3)	438 (360 to 532)	4.6 (3.8 to 5.5)	60 (36 to 92)	0.6 (0.4 to 0.9)	498 (408 to 609)	5.2 (4.3 to 6.3)
Belize	Both	9 (8 to 10)	9.3 (8.3 to 10.5)	77 (68 to 86)	79.8 (71.1 to 89.7)	5 (5 to 6)	5.3 (4.8 to 5.9)	141 (125 to 159)	142.1 (126.7 to 160.2)	5 (4 to 7)	5.7 (4 to 7.7)	146 (130 to 164)	147.8 (132.1 to 165.9)	44 (37 to 53)	13.8 (11.6 to 16.3)	366 (310 to 431)	113.4 (97 to 132.1)	18 (16 to 22)	6.2 (5.3 to 7.3)	585 (490 to 694)	175.9 (147.5 to 208.4)	27 (18 to 38)	8.2 (5.6 to 11.5)	612 (512 to 726)	184.1 (154.5 to 217.7)
	Female	9 (8 to 10)	18.5 (16.4 to 20.9)	75 (67 to 85)	159.2 (142.1 to 179.3)	5 (4 to 5)	10.3 (9.2 to 11.5)	136 (121 to 155)	282 (250.6 to 318.8)	5 (4 to 7)	11.2 (7.8 to 15.2)	142 (126 to 160)	293.2 (261 to 329.4)	43 (36 to 52)	27 (22.7 to 32)	359 (304 to 423)	223.3 (191.2 to 260.5)	18 (15 to 21)	11.9 (10.1 to 14)	568 (475 to 674)	340.3 (285.2 to 403.5)	26 (18 to 37)	16.1 (11 to 22.6)	594 (497 to 706)	356.3 (299.2 to 421.4)
	Male	0 (0 to 0)	0.5 (0.4 to 0.6)	1 (1 to 2)	2.9 (2.4 to 3.3)	0 (0 to 0)	0.4 (0.4 to 0.5)	4 (4 to 5)	8.6 (7.4 to 9.8)	0 (0 to 0)	0.3 (0.2 to 0.4)	4 (4 to 5)	8.8 (7.7 to 10.2)	1 (1 to 1)	0.7 (0.6 to 0.9)	7 (5 to 8)	4.5 (3.7 to 5.5)	1 (1 to 1)	0.5 (0.4 to 0.6)	18 (15 to 21)	11.7 (9.7 to 13.9)	1 (0 to 1)	0.5 (0.3 to 0.6)	18 (15 to 22)	12.1 (10.1 to 14.3)
Benin	Both	177 (132 to 226)	8.2 (6.1 to 10.4)	1202 (952 to 1486)	55.3 (44 to 67.4)	140 (104 to 177)	6.8 (5 to 8.5)	4379 (3244 to 5663)	191.7 (142.2 to 246.1)	99 (64 to 142)	4.5 (3 to 6.4)	4478 (3325 to 5784)	196.2 (145.7 to 252.4)	638 (452 to 874)	11.3 (8.2 to 15.1)	4418 (3246 to 5889)	75.9 (57.8 to 98.4)	440 (319 to 590)	8.6 (6.4 to 11.1)	14012 (9778 to 19466)	228.8 (163.9 to 310.9)	368 (219 to 553)	6.3 (3.9 to 9.3)	14381 (10041 to 20040)	235.1 (168.7 to 319.4)
	Female	176 (130 to 224)	15.5 (11.5 to 19.6)	1195 (944 to 1478)	104.8 (83.4 to 128.1)	139 (103 to 176)	12.8 (9.5 to 16.1)	4346 (3210 to 5626)	361.2 (267.6 to 463.7)	99 (64 to 142)	8.5 (5.6 to 12.1)	4444 (3288 to 5746)	369.7 (274.6 to 475.2)	634 (448 to 869)	21.2 (15.4 to 28.3)	4394 (3224 to 5863)	143.4 (108.4 to 186.4)	437 (316 to 586)	15.8 (11.7 to 20.5)	13919 (9713 to 19342)	432.9 (308.6 to 589.9)	366 (217 to 550)	11.9 (7.3 to 17.5)	14285 (9975 to 19877)	444.8 (317.4 to 606.6)
	Male	1 (1 to 2)	0.1 (0.1 to 0.2)	7 (5 to 10)	0.7 (0.5 to 0.9)	1 (1 to 2)	0.1 (0.1 to 0.2)	33 (24 to 46)	3.1 (2.2 to 4.4)	1 (0 to 1)	0.1 (0 to 0.1)	34 (24 to 47)	3.2 (2.2 to 4.5)	4 (3 to 6)	0.2 (0.1 to 0.3)	24 (16 to 34)	0.9 (0.6 to 1.3)	3 (2 to 5)	0.2 (0.1 to 0.2)	93 (63 to 137)	3.5 (2.3 to 5.2)	3 (2 to 4)	0.1 (0.1 to 0.2)	96 (64 to 141)	3.6 (2.4 to 5.3)
Bermuda	Both	33 (29 to 37)	51.4 (45.7 to 57.3)	267 (237 to 297)	414.4 (368.1 to 460.3)	13 (11 to 14)	21.1 (19 to 23.2)	327 (291 to 362)	507 (453.1 to 561.1)	20 (14 to 27)	30.6 (22.1 to 41.7)	347 (309 to 384)	537.7 (478.6 to 594.9)	50 (41 to 63)	43.1 (34.8 to 54.4)	453 (378 to 552)	389.6 (323 to 477.6)	14 (11 to 18)	11 (8.9 to 13.7)	283 (231 to 354)	248.6 (203.2 to 311.7)	31 (21 to 44)	26.9 (18 to 37.9)	314 (257 to 393)	275.5 (224.8 to 346.9)
	Female	33 (29 to 37)	92.7 (82 to 103.9)	266 (236 to 296)	755.5 (670 to 842.8)	13 (11 to 14)	36.4 (32.7 to 40.2)	325 (289 to 360)	924.7 (824.9 to 1023.6)	20 (14 to 27)	55.5 (39.8 to 75.8)	344 (307 to 382)	980.2 (870 to 1087.5)	50 (40 to 63)	80.2 (64.6 to 101.2)	451 (376 to 550)	730.3 (603.2 to 897.1)	14 (11 to 17)	19.3 (15.7 to 24.2)	280 (229 to 351)	464.6 (379.8 to 582.3)	31 (21 to 44)	50.2 (33.6 to 71)	311 (255 to 390)	514.8 (419.3 to 649.1)

		1990												2019											
		Incidence		Prevalence		Deaths		YLLs (Years of Life Lost)		YLDs (Years Lived with Disability)		DALYs (Disability-Adjusted Life Years)		Incidence		Prevalence		Deaths		YLLs (Years of Life Lost)		YLDs (Years Lived with Disability)		DALYs (Disability-Adjusted Life Years)	
Location	Sex	Number	Rate	Number	Rate	Number	Rate	Number	Rate	Number	Rate	Number	Rate	Number	Rate	Number	Rate	Number	Rate	Number	Rate	Number	Rate	Number	Rate
	Male	0 (0 to 0)	0.6 (0.5 to 0.7)	1 (1 to 1)	3.7 (3.1 to 4.5)	0 (0 to 0)	0.4 (0.3 to 0.5)	2 (2 to 3)	8 (6.8 to 9.4)	0 (0 to 0)	0.4 (0.3 to 0.5)	2 (2 to 3)	8.4 (7.2 to 9.9)	0 (0 to 0)	0.5 (0.4 to 0.7)	2 (2 to 3)	3.7 (2.8 to 4.8)	0 (0 to 0)	0.2 (0.2 to 0.3)	2 (2 to 3)	4.4 (3.5 to 5.5)	0 (0 to 0)	0.4 (0.2 to 0.5)	3 (2 to 3)	4.8 (3.7 to 6)
Bhutan	Both	23 (15 to 32)	7.6 (5.1 to 10.7)	158 (112 to 211)	53.8 (40 to 69.8)	17 (11 to 24)	6.2 (4.1 to 8.6)	600 (391 to 860)	185.2 (122.5 to 261.2)	13 (8 to 19)	4.3 (2.6 to 6.5)	613 (399 to 877)	189.4 (126.1 to 266.3)	63 (44 to 87)	10.2 (7.2 to 13.9)	500 (366 to 675)	80 (59.9 to 106.1)	35 (24 to 47)	6 (4.3 to 8.1)	1042 (708 to 1455)	163.4 (112.3 to 225.3)	39 (23 to 61)	6.2 (3.8 to 9.6)	1081 (733 to 1511)	169.6 (116.7 to 233.7)
	Female	22 (15 to 31)	14.9 (9.8 to 21.2)	156 (110 to 209)	105.8 (77 to 139)	17 (11 to 24)	11.8 (7.8 to 16.7)	590 (383 to 846)	372.2 (243.7 to 524.7)	13 (7 to 19)	8.4 (5 to 12.8)	603 (392 to 865)	380.6 (249.6 to 534.7)	62 (43 to 87)	20.9 (14.6 to 28.8)	495 (362 to 669)	164.6 (122.6 to 218.8)	34 (24 to 46)	12 (8.5 to 16.4)	1027 (694 to 1440)	335.3 (228.2 to 464.4)	38 (23 to 60)	12.6 (7.7 to 19.7)	1065 (719 to 1493)	347.9 (236.9 to 482.3)
	Male	0 (0 to 1)	0.4 (0.2 to 0.6)	2 (1 to 3)	1.6 (1 to 2.5)	0 (0 to 1)	0.4 (0.3 to 0.6)	10 (6 to 15)	7.8 (4.8 to 11.8)	0 (0 to 0)	0.2 (0.1 to 0.3)	10 (6 to 16)	8 (4.9 to 12.1)	1 (0 to 1)	0.3 (0.2 to 0.5)	5 (3 to 8)	1.7 (1 to 2.9)	1 (0 to 1)	0.2 (0.1 to 0.4)	15 (8 to 24)	5 (2.8 to 8.1)	1 (0 to 1)	0.2 (0.1 to 0.3)	15 (9 to 25)	5.2 (2.9 to 8.5)
Bolivia (Plurinational State of)	Both	426 (327 to 550)	11.8 (9.3 to 15.1)	2825 (2229 to 3565)	77 (61.6 to 95.7)	307 (239 to 396)	9 (7.1 to 11.4)	9968 (7572 to 12986)	260.8 (200.7 to 338.8)	226 (145 to 321)	6.2 (4 to 8.6)	10194 (7737 to 13287)	266.9 (205.7 to 346)	1704 (1282 to 2278)	18.1 (13.7 to 23.9)	12596 (9603 to 16528)	131.2 (101.6 to 171.9)	902 (689 to 1167)	10.1 (7.9 to 12.9)	26127 (19306 to 34779)	269.8 (200.9 to 355.9)	958 (590 to 1381)	10.1 (6.2 to 14.4)	27086 (19973 to 35998)	279.8 (208.9 to 367.8)
	Female	419 (321 to 541)	22 (17.1 to 28.2)	2789 (2192 to 3529)	144.5 (115.5 to 180.3)	301 (234 to 389)	16.5 (13 to 21.1)	9795 (7419 to 12817)	489.3 (374.2 to 637.7)	222 (141 to 316)	11.5 (7.5 to 16.2)	10017 (7571 to 13119)	500.8 (383.4 to 651.2)	1685 (1268 to 2252)	34.5 (26.2 to 45.8)	12482 (9512 to 16414)	252.1 (194.6 to 330.6)	888 (678 to 1152)	19 (14.8 to 24.4)	25777 (18981 to 34364)	516.9 (383.6 to 683.2)	947 (579 to 1365)	19.2 (11.9 to 27.6)	26724 (19663 to 35508)	536.1 (398.9 to 707.7)
	Male	7 (5 to 11)	0.5 (0.3 to 0.8)	36 (24 to 56)	2.3 (1.5 to 3.5)	7 (4 to 10)	0.5 (0.3 to 0.7)	172 (113 to 267)	10.7 (7 to 16.6)	4 (2 to 7)	0.3 (0.2 to 0.4)	176 (116 to 273)	11 (7.2 to 17)	19 (12 to 29)	0.5 (0.3 to 0.7)	113 (75 to 166)	2.6 (1.7 to 3.8)	14 (9 to 22)	0.4 (0.2 to 0.6)	350 (225 to 522)	8 (5.1 to 11.9)	12 (7 to 18)	0.3 (0.2 to 0.4)	362 (232 to 542)	8.3 (5.2 to 12.4)
Bosnia and Herzegovina	Both	647 (591 to 712)	14.5 (13.3 to 15.9)	6651 (5941 to 7484)	153.1 (136 to 172)	324 (298 to 352)	7.7 (7.1 to 8.4)	10125 (9303 to 11046)	220.6 (203.5 to 240.1)	452 (315 to 622)	10.2 (7.1 to 14.1)	10577 (9737 to 11518)	230.8 (213 to 251.2)	1635 (1253 to 2105)	29.9 (22.9 to 38.7)	15103 (12303 to 18817)	274.1 (221.2 to 342.1)	668 (523 to 838)	11.7 (9.2 to 14.7)	15864 (12301 to 20128)	290.6 (224.7 to 370.6)	1087 (723 to 1550)	19.9 (13.2 to 28.5)	16951 (13093 to 21750)	310.5 (240.6 to 401)
	Female	644 (589 to 709)	26.7 (24.5 to 29.4)	6617 (5912 to 7444)	277.5 (246.9 to 311)	323 (297 to 351)	13.8 (12.7 to 15)	10088 (9268 to 11010)	412 (379.3 to 448.9)	450 (313 to 620)	18.7 (13 to 25.7)	10537 (9694 to 11478)	430.7 (397.4 to 469.1)	1612 (1234 to 2079)	55.5 (42.2 to 72.2)	14925 (12170 to 18591)	509.8 (409.6 to 639.2)	657 (513 to 825)	20.9 (16.4 to 26.3)	15645 (12121 to 19837)	540.8 (416.6 to 692.5)	1070 (708 to 1527)	36.9 (24.3 to 53.1)	16715 (12896 to 21467)	577.8 (444.9 to 746.1)
	Male	2 (2 to 3)	0.1 (0.1 to 0.2)	34 (27 to 41)	1.9 (1.6 to 2.4)	1 (1 to 2)	0.1 (0.1 to 0.1)	38 (33 to 44)	1.9 (1.6 to 2.2)	2 (2 to 3)	0.1 (0.1 to 0.2)	40 (35 to 46)	2 (1.8 to 2.3)	23 (16 to 30)	0.9 (0.6 to 1.1)	179 (130 to 234)	6.7 (4.9 to 8.6)	11 (8 to 14)	0.4 (0.3 to 0.6)	219 (157 to 288)	8.2 (5.9 to 10.7)	17 (10 to 26)	0.6 (0.4 to 0.9)	236 (169 to 310)	8.9 (6.4 to 11.6)
Botswana	Both	90 (64 to 126)	14.7 (10.8 to 20.2)	652 (484 to 863)	103.5 (79.5 to 133.9)	64 (47 to 87)	11.5 (8.6 to 15.3)	1981 (1391 to 2762)	299.3 (214.7 to 412)	53 (32 to 80)	8.3 (5.2 to 12.3)	2034 (1424 to 2820)	307.6 (220.7 to 423.2)	448 (283 to 669)	28.2 (18.5 to 41.1)	3356 (2190 to 4894)	202 (139.1 to 286.6)	235 (151 to 342)	17.3 (11.6 to 24.5)	7369 (4546 to 11005)	432.5 (276.4 to 628.9)	269 (156 to 432)	16.3 (9.8 to 25.5)	7638 (4706 to 11389)	448.8 (288.5 to 656.2)
	Female	87 (61 to 122)	25.4 (18.3 to 35)	634 (471 to 845)	181 (137.6 to 236.5)	62 (45 to 85)	19.3 (14.1 to 26.2)	1915 (1341 to 2677)	525.7 (372.8 to 729)	51 (31 to 77)	14.4 (8.9 to 21.6)	1966 (1366 to 2744)	540.2 (382 to 748.5)	438 (276 to 655)	49 (31.7 to 72.2)	3293 (2142 to 4813)	356.8 (240.8 to 510.8)	230 (147 to 335)	28.6 (19 to 41)	7192 (4415 to 10806)	763.9 (483.3 to 1129.6)	263 (152 to 424)	28.5 (17 to 45.2)	7455 (4571 to 11175)	792.5 (503.9 to 1179.1)
	Male	3 (2 to 4)	1.2 (0.7 to 1.6)	18 (12 to 24)	6.7 (4.9 to 9)	2 (1 to 3)	1 (0.7 to 1.4)	66 (41 to 97)	23.4 (15.1 to 33.4)	2 (1 to 3)	0.7 (0.4 to 1.1)	68 (43 to 100)	24.2 (15.5 to 34.2)	9 (6 to 13)	1.6 (1 to 2.2)	63 (43 to 88)	9.8 (7 to 13.4)	6 (4 to 9)	1.2 (0.8 to 1.6)	177 (111 to 257)	25.6 (17.1 to 36.6)	6 (4 to 9)	1 (0.6 to 1.5)	183 (115 to 265)	26.6 (17.8 to 37.6)
Brazil	Both	16875 (16237 to 17474)	17 (16.2 to 17.6)	131278 (125391 to 137951)	130.1 (123.7 to 137.2)	8883 (8554 to 9203)	9.7 (9.2 to 10)	278870 (269756 to 288610)	267.7 (258.6 to 277.1)	9638 (6909 to 12805)	9.5 (6.9 to 12.7)	288508 (279019 to 298920)	277.2 (267.3 to 287)	52473 (49142 to 55904)	21.5 (20.1 to 22.9)	441520 (414506 to 470953)	180.5 (169.3 to 192.6)	20121 (18758 to 21373)	8.4 (7.8 to 9)	566622 (534087 to 600761)	230.3 (216.9 to 244.2)	31437 (22505 to 42077)	12.9 (9.2 to 17.2)	598059 (563114 to 636739)	243.2 (229.1 to 258.8)
	Female	16745 (16111 to 17344)	31.9 (30.6 to 33.1)	130390 (124548 to 137011)	246 (234.1 to 259.4)	8789 (8465 to 9105)	17.9 (17.1 to 18.5)	276262 (267172 to 285982)	507.1 (490.2 to 525.4)	9555 (6847 to 12690)	18 (13 to 23.9)	285818 (276300 to 296119)	525.1 (506.7 to 543.9)	51856 (48535 to 55276)	39.6 (37.1 to 42.3)	437220 (410282 to 466293)	334.2 (313.6 to 356.8)	19764 (18415 to 21001)	15.1 (14.1 to 16.1)	557413 (524342 to 591705)	425.7 (400.7 to 451.7)	31035 (22234 to 41560)	23.7 (17 to 31.8)	588448 (553385 to 627133)	449.4 (422.8 to 478.8)
	Male	131 (121 to 141)	0.3 (0.3 to 0.3)	888 (808 to 987)	1.9 (1.8 to 2.1)	94 (87 to 101)	0.2 (0.2 to 0.3)	2608 (2420 to 2795)	5.7 (5.3 to 6.1)	83 (59 to 111)	0.2 (0.1 to 0.2)	2691 (2501 to 2887)	5.8 (5.4 to 6.3)	617 (557 to 684)	0.6 (0.5 to 0.6)	4300 (3864 to 4782)	3.9 (3.5 to 4.3)	357 (326 to 395)	0.3 (0.3 to 0.4)	9209 (8420 to 10113)	8.2 (7.5 to 9)	402 (286 to 540)	0.4 (0.3 to 0.5)	9611 (8779 to 10553)	8.6 (7.8 to 9.4)

		1990												2019											
		Incidence		Prevalence		Deaths		YLLs (Years of Life Lost)		YLDs (Years Lived with Disability)		DALYs (Disability-Adjusted Life Years)		Incidence		Prevalence		Deaths		YLLs (Years of Life Lost)		YLDs (Years Lived with Disability)		DALYs (Disability-Adjusted Life Years)	
Location	Sex	Number	Rate	Number	Rate	Number	Rate	Number	Rate	Number	Rate	Number	Rate	Number	Rate	Number	Rate	Number	Rate	Number	Rate	Number	Rate	Number	Rate
Brunei Darussalam	Both	32 (25 to 39)	20.9 (17.1 to 25.5)	308 (251 to 371)	227.1 (191.2 to 268.4)	13 (10 to 16)	10 (8.4 to 12)	495 (390 to 613)	307.9 (251 to 377.1)	23 (15 to 34)	15.8 (10.5 to 22.6)	518 (408 to 641)	323.7 (263.5 to 393.8)	142 (115 to 175)	34.7 (28.8 to 41.7)	1356 (1144 to 1621)	343.9 (296.1 to 400.9)	43 (36 to 52)	12.4 (10.5 to 14.5)	1495 (1225 to 1823)	349.3 (291.5 to 417.9)	103 (70 to 146)	25.4 (17.4 to 35)	1599 (1312 to 1935)	374.6 (312.7 to 447)
	Female	31 (24 to 39)	42.9 (34.7 to 52.4)	304 (247 to 366)	456.6 (383 to 540.8)	13 (10 to 16)	19.5 (16.2 to 23.5)	486 (381 to 605)	640.5 (518.7 to 785.9)	23 (15 to 34)	32.1 (21.2 to 46.3)	509 (399 to 629)	672.6 (542.1 to 825.6)	141 (114 to 174)	67.7 (55.5 to 82.1)	1346 (1136 to 1611)	666.3 (570.4 to 785.2)	43 (36 to 52)	22.9 (19.3 to 27)	1480 (1208 to 1803)	688.3 (569.8 to 827.4)	103 (69 to 145)	49.5 (33.8 to 68.9)	1582 (1291 to 1918)	737.8 (612.5 to 882.3)
	Male	0 (0 to 1)	1.2 (0.8 to 1.8)	4 (3 to 5)	7.4 (5.3 to 10.2)	0 (0 to 0)	0.9 (0.6 to 1.4)	9 (6 to 13)	17.3 (11.4 to 25.3)	0 (0 to 1)	0.8 (0.4 to 1.2)	9 (6 to 13)	18.1 (11.9 to 26.7)	1 (1 to 2)	1 (0.8 to 1.4)	10 (8 to 13)	7.4 (5.8 to 9.6)	1 (0 to 1)	0.7 (0.5 to 0.9)	16 (12 to 21)	11.6 (8.8 to 15.2)	1 (1 to 1)	0.7 (0.4 to 1)	17 (12 to 22)	12.3 (9.3 to 16)
Bulgaria	Both	3382 (3111 to 3652)	29 (26.7 to 31.3)	33403 (30249 to 37008)	282.8 (257.4 to 311.4)	1265 (1182 to 1350)	10.8 (10 to 11.5)	37143 (34538 to 39802)	320.9 (298.2 to 343.9)	2371 (1654 to 3247)	20.2 (14.1 to 27.8)	39514 (36781 to 42410)	341.1 (317.1 to 366.1)	5122 (4003 to 6482)	41.9 (32.4 to 53.3)	48341 (39616 to 58938)	389.7 (312.9 to 481.6)	1710 (1364 to 2121)	12.9 (10.2 to 16)	40641 (31831 to 51298)	342.4 (264.8 to 436.3)	3523 (2310 to 5030)	28.7 (18.3 to 41.3)	44163 (34651 to 55947)	371.1 (287.5 to 473.3)
	Female	3362 (3090 to 3630)	55.3 (50.8 to 59.7)	33200 (30060 to 36790)	535.8 (488.1 to 589.8)	1258 (1175 to 1343)	20.1 (18.7 to 21.5)	36948 (34351 to 39605)	615 (571 to 659.6)	2354 (1642 to 3227)	38.5 (26.8 to 52.9)	39302 (36594 to 42188)	653.4 (606.7 to 701.5)	5044 (3941 to 6398)	78.2 (59.9 to 100.3)	47723 (38965 to 58192)	725.8 (578.7 to 902.2)	1685 (1343 to 2090)	23 (18.1 to 28.8)	40071 (31398 to 50525)	645.1 (496.1 to 828.7)	3463 (2278 to 4963)	53.4 (34.3 to 77.4)	43535 (34114 to 55155)	698.5 (537.5 to 895.5)
	Male	20 (16 to 25)	0.4 (0.3 to 0.4)	203 (169 to 244)	3.7 (3.1 to 4.4)	8 (6 to 9)	0.1 (0.1 to 0.2)	195 (166 to 227)	3.5 (3 to 4)	17 (11 to 25)	0.3 (0.2 to 0.4)	212 (180 to 248)	3.8 (3.3 to 4.4)	78 (55 to 106)	1.4 (1 to 1.9)	618 (453 to 839)	10.9 (8 to 14.8)	26 (19 to 34)	0.4 (0.3 to 0.6)	569 (418 to 750)	10.1 (7.4 to 13.4)	59 (36 to 89)	1 (0.6 to 1.5)	628 (461 to 831)	11.1 (8.2 to 14.7)
Burkina Faso	Both	527 (404 to 664)	11.3 (8.8 to 14.1)	3386 (2703 to 4164)	70.2 (57.3 to 85.3)	417 (323 to 523)	9.6 (7.6 to 11.9)	13111 (9979 to 16771)	261.1 (200.1 to 330)	289 (198 to 420)	6 (4.2 to 8.6)	13400 (10197 to 17152)	267.1 (204.1 to 338)	1575 (1203 to 2013)	15 (11.8 to 18.5)	10720 (8356 to 13414)	97 (78 to 118.6)	1088 (854 to 1366)	11.5 (9.2 to 14)	34921 (26289 to 45083)	302.4 (234.8 to 382.2)	906 (581 to 1306)	8.2 (5.4 to 11.8)	35827 (26850 to 46134)	310.7 (240.8 to 391.7)
	Female	523 (401 to 661)	21.1 (16.5 to 26.4)	3369 (2687 to 4147)	131.3 (107 to 159.4)	415 (321 to 520)	18 (14.1 to 22.2)	13032 (9893 to 16696)	485.3 (370.4 to 612.7)	288 (197 to 417)	11.2 (7.8 to 16)	13320 (10120 to 17067)	496.5 (378.4 to 628.9)	1566 (1196 to 2002)	27.1 (21.3 to 33.7)	10672 (8321 to 13365)	176.8 (141.9 to 216.8)	1081 (848 to 1353)	20.5 (16.4 to 25.1)	34735 (26134 to 44886)	551.6 (425.7 to 697.7)	900 (578 to 1300)	15 (9.8 to 21.4)	35635 (26711 to 45954)	566.5 (436.6 to 714.7)
	Male	3 (2 to 5)	0.2 (0.1 to 0.2)	17 (12 to 26)	0.8 (0.5 to 1.1)	3 (2 to 4)	0.1 (0.1 to 0.2)	78 (51 to 121)	3.4 (2.2 to 5.3)	2 (1 to 3)	0.1 (0.1 to 0.1)	80 (52 to 124)	3.5 (2.3 to 5.4)	8 (6 to 12)	0.2 (0.1 to 0.3)	47 (33 to 69)	1 (0.7 to 1.5)	7 (5 to 10)	0.2 (0.1 to 0.3)	187 (127 to 276)	4 (2.7 to 5.9)	5 (3 to 8)	0.1 (0.1 to 0.2)	192 (131 to 283)	4.1 (2.8 to 6.1)
Burundi	Both	321 (236 to 427)	12.6 (9.4 to 16.7)	1920 (1471 to 2459)	73.3 (57.3 to 93.6)	266 (198 to 352)	11.1 (8.3 to 14.5)	8508 (6117 to 11527)	310.4 (227.7 to 414.9)	175 (114 to 262)	6.7 (4.4 to 10)	8683 (6257 to 11757)	317.1 (233 to 423.6)	600 (413 to 847)	11.3 (8 to 15.7)	4017 (2887 to 5489)	71.9 (54.2 to 95.5)	436 (303 to 611)	9.2 (6.6 to 12.8)	14295 (9773 to 20289)	242.3 (168.5 to 340.7)	345 (212 to 537)	6.2 (3.9 to 9.5)	14640 (9996 to 20722)	248.5 (173.7 to 348.4)
	Female	288 (206 to 393)	20.3 (14.7 to 27.5)	1783 (1345 to 2301)	123.6 (95.2 to 158.9)	234 (169 to 315)	17.3 (12.6 to 23.2)	7767 (5445 to 10662)	513.8 (366.9 to 703.1)	159 (100 to 243)	11 (7 to 16.7)	7925 (5570 to 10871)	524.7 (375.1 to 719.3)	548 (375 to 772)	20.7 (14.5 to 28.8)	3754 (2711 to 5117)	137.4 (102.7 to 183.1)	389 (269 to 547)	16.2 (11.3 to 22.5)	13161 (8909 to 18674)	454.1 (314.7 to 640.1)	316 (192 to 497)	11.6 (7.2 to 18)	13477 (9149 to 19108)	465.6 (322.4 to 657.7)
	Male	33 (21 to 50)	3.5 (2.2 to 5.3)	137 (91 to 199)	13.6 (9.2 to 19.4)	32 (20 to 48)	3.6 (2.3 to 5.4)	741 (455 to 1114)	72.1 (45.2 to 109.4)	17 (9 to 27)	1.7 (0.9 to 2.8)	758 (465 to 1141)	73.8 (46.3 to 112.6)	52 (31 to 82)	2.7 (1.6 to 4.1)	264 (167 to 404)	12 (7.8 to 17.6)	47 (28 to 73)	2.6 (1.6 to 4)	1134 (664 to 1794)	49.6 (29.9 to 77.6)	29 (15 to 48)	1.3 (0.7 to 2.2)	1163 (681 to 1839)	51 (30.7 to 79.6)
Cabo Verde	Both	24 (20 to 28)	11.4 (9.7 to 13.4)	180 (155 to 209)	85 (73 to 99.2)	17 (15 to 20)	8.1 (7 to 9.4)	484 (408 to 576)	235.1 (197.3 to 280.9)	14 (9 to 20)	6.7 (4.5 to 9.6)	498 (420 to 591)	241.8 (202.4 to 289.3)	69 (56 to 85)	15.2 (12.2 to 18.7)	534 (441 to 641)	116.6 (96.4 to 140)	38 (30 to 47)	8.8 (6.9 to 10.7)	967 (778 to 1181)	207.5 (166.7 to 251.9)	42 (29 to 58)	9.1 (6.2 to 12.6)	1009 (811 to 1235)	216.6 (173.8 to 264)
	Female	24 (20 to 28)	19.4 (16.4 to 22.8)	179 (154 to 209)	145.2 (124.6 to 169.3)	17 (15 to 20)	13.8 (11.9 to 16.1)	482 (407 to 575)	399.1 (334.6 to 476.7)	14 (9 to 20)	11.4 (7.7 to 16.4)	496 (419 to 590)	410.5 (344.8 to 490.4)	69 (55 to 85)	27.7 (22.2 to 34.2)	532 (440 to 639)	215.5 (177.8 to 259.2)	38 (30 to 47)	15.4 (12.2 to 18.8)	962 (774 to 1174)	385.3 (309.9 to 470.2)	42 (28 to 58)	16.8 (11.5 to 23.2)	1004 (807 to 1229)	402.1 (323.1 to 491.4)
	Male	0 (0 to 0)	0.1 (0 to 0.1)	0 (0 to 0)	0.4 (0.3 to 0.5)	0 (0 to 0)	0 (0 to 0.1)	1 (1 to 2)	1.2 (0.9 to 1.5)	0 (0 to 0)	0 (0 to 0.1)	1 (1 to 2)	1.2 (0.9 to 1.5)	0 (0 to 0)	0.2 (0.1 to 0.2)	2 (1 to 3)	1 (0.6 to 1.4)	0 (0 to 0)	0.1 (0.1 to 0.2)	5 (3 to 7)	2.4 (1.6 to 3.4)	0 (0 to 0)	0.1 (0.1 to 0.2)	5 (3 to 7)	2.5 (1.6 to 3.6)
Cambodia	Both	457 (348 to 603)	8.2 (6.2 to 10.7)	3451 (2767 to 4365)	63.6 (51.4 to 79.1)	333 (252 to 436)	6.3 (4.8 to 8.2)	12238 (9310 to 16270)	208.3 (157.8 to 274.2)	275 (177 to 404)	4.9 (3.3 to 7.1)	12512 (9526 to 16641)	213.2 (161.4 to 281.2)	1816 (1374 to 2290)	13.3 (10.2 to 16.5)	14281 (11276 to 17524)	103.4 (82.7 to 125.5)	1029 (794 to 1283)	8.1 (6.3 to 9.9)	33758 (25486 to 42929)	239.6 (182.7 to 301.3)	1122 (731 to 1645)	8.1 (5.4 to 11.8)	34880 (26336 to 44358)	247.7 (188.9 to 311.1)

		1990												2019											
		Incidence		Prevalence		Deaths		YLLs (Years of Life Lost)		YLDs (Years Lived with Disability)		DALYs (Disability-Adjusted Life Years)		Incidence		Prevalence		Deaths		YLLs (Years of Life Lost)		YLDs (Years Lived with Disability)		DALYs (Disability-Adjusted Life Years)	
Location	Sex	Number	Rate	Number	Rate	Number	Rate	Number	Rate	Number	Rate	Number	Rate	Number	Rate	Number	Rate	Number	Rate	Number	Rate	Number	Rate	Number	Rate
	Female	450 (341 to 597)	14.2 (10.7 to 18.5)	3420 (2739 to 4329)	110.6 (88.8 to 137.6)	328 (246 to 429)	10.9 (8.1 to 14.1)	12084 (9116 to 16161)	361.4 (272.7 to 477.4)	271 (173 to 399)	8.5 (5.6 to 12.3)	12355 (9349 to 16485)	369.9 (278.5 to 488.6)	1788 (1352 to 2261)	23.5 (17.9 to 29.4)	14123 (11112 to 17385)	184.4 (146.3 to 225)	1009 (772 to 1259)	13.9 (10.7 to 17.1)	33209 (24987 to 42334)	428.3 (326.1 to 541.5)	1104 (718 to 1622)	14.4 (9.5 to 21)	34314 (25890 to 43586)	442.7 (336.4 to 558.7)
	Male	6 (4 to 9)	0.3 (0.2 to 0.5)	31 (20 to 46)	1.4 (0.9 to 2.1)	5 (3 to 8)	0.3 (0.2 to 0.5)	154 (97 to 234)	7.2 (4.4 to 10.8)	4 (2 to 6)	0.2 (0.1 to 0.3)	158 (99 to 239)	7.3 (4.6 to 11)	28 (18 to 39)	0.6 (0.4 to 0.8)	158 (104 to 228)	2.9 (1.9 to 4.1)	20 (14 to 28)	0.4 (0.3 to 0.6)	549 (363 to 782)	10 (6.7 to 14)	18 (10 to 28)	0.3 (0.2 to 0.5)	567 (372 to 806)	10.4 (6.9 to 14.5)
Cameroon	Both	551 (436 to 683)	10.9 (8.7 to 13.4)	3629 (2962 to 4402)	70.5 (58.1 to 84.5)	423 (331 to 523)	8.9 (7.1 to 11)	13728 (10723 to 17142)	251.6 (197.7 to 312.1)	306 (202 to 432)	5.9 (3.9 to 8.2)	14034 (10899 to 17507)	257.6 (201.7 to 319.4)	2256 (1523 to 3301)	16.1 (11.1 to 22.9)	15635 (10963 to 22404)	106.5 (77.3 to 147.9)	1462 (1004 to 2098)	11.6 (8.3 to 16.2)	47508 (31558 to 70073)	309.8 (210.3 to 448.5)	1310 (766 to 2063)	9 (5.4 to 13.8)	48818 (32371 to 72033)	318.7 (218.4 to 464.2)
	Female	548 (433 to 679)	21.1 (16.8 to 25.9)	3607 (2942 to 4377)	136.8 (112.6 to 163.8)	419 (328 to 519)	17.2 (13.7 to 21.2)	13634 (10632 to 17030)	488.5 (383.3 to 607.2)	304 (200 to 429)	11.4 (7.5 to 15.9)	13938 (10828 to 17385)	499.9 (391.7 to 620.5)	2242 (1511 to 3285)	30.8 (21.1 to 44.3)	15550 (10886 to 22289)	206.7 (149.2 to 289)	1451 (998 to 2087)	21.9 (15.6 to 30.8)	47189 (31302 to 69578)	601.5 (407.2 to 875.5)	1301 (761 to 2051)	17.3 (10.4 to 26.7)	48490 (32109 to 71522)	618.8 (422.3 to 901.4)
	Male	4 (2 to 6)	0.2 (0.1 to 0.2)	22 (15 to 31)	0.9 (0.6 to 1.2)	3 (2 to 5)	0.2 (0.1 to 0.2)	94 (61 to 136)	3.7 (2.4 to 5.5)	2 (1 to 4)	0.1 (0.1 to 0.2)	96 (63 to 139)	3.8 (2.5 to 5.7)	14 (9 to 22)	0.2 (0.2 to 0.4)	85 (56 to 124)	1.3 (0.8 to 1.9)	11 (7 to 17)	0.2 (0.1 to 0.3)	319 (208 to 481)	4.7 (3 to 7.2)	9 (5 to 15)	0.1 (0.1 to 0.2)	328 (213 to 492)	4.9 (3.1 to 7.4)
Canada	Both	16637 (15606 to 17749)	52.6 (49.3 to 56.2)	170428 (157805 to 186280)	537.1 (497.5 to 586.3)	4663 (4422 to 4840)	14.7 (13.9 to 15.3)	116769 (112650 to 120348)	372.1 (358.9 to 383.5)	12337 (8540 to 16881)	38.9 (26.9 to 53)	129107 (123019 to 135084)	411 (392.3 to 430)	28616 (22312 to 36219)	47.2 (36.8 to 60.5)	309384 (256869 to 371612)	498.9 (409.7 to 607.4)	6565 (5890 to 7168)	9.8 (8.9 to 10.6)	138252 (127187 to 148865)	232.7 (215 to 249.9)	21600 (14425 to 31314)	35.4 (23.4 to 51.4)	159852 (144867 to 175827)	268.2 (244.4 to 294.2)
	Female	16591 (15564 to 17700)	98.8 (92.6 to 105.6)	169883 (157302 to 185566)	998.8 (925.9 to 1086.7)	4644 (4403 to 4821)	26.5 (25.2 to 27.5)	116323 (112190 to 119879)	703.9 (679.9 to 724.9)	12294 (8509 to 16824)	72.8 (50.2 to 99.4)	128618 (122498 to 134617)	776.7 (742.1 to 812.7)	28469 (22222 to 36022)	90.1 (70.1 to 115.6)	307829 (255609 to 369903)	949.9 (779.5 to 1159.7)	6515 (5843 to 7113)	18 (16.4 to 19.5)	137243 (126174 to 147832)	443.9 (410.1 to 476.5)	21472 (14322 to 31121)	67.6 (44.6 to 98)	158715 (143706 to 174308)	511.5 (465 to 561.2)
	Male	46 (38 to 55)	0.3 (0.3 to 0.4)	545 (452 to 649)	3.8 (3.2 to 4.6)	20 (17 to 22)	0.1 (0.1 to 0.2)	446 (399 to 494)	3.1 (2.8 to 3.4)	43 (28 to 62)	0.3 (0.2 to 0.4)	489 (432 to 547)	3.4 (3 to 3.8)	146 (99 to 207)	0.5 (0.3 to 0.7)	1554 (1191 to 2043)	5.1 (3.9 to 6.6)	51 (41 to 62)	0.2 (0.1 to 0.2)	1009 (829 to 1216)	3.4 (2.8 to 4)	128 (76 to 206)	0.4 (0.3 to 0.7)	1137 (927 to 1382)	3.8 (3.2 to 4.6)
Central African Republic	Both	165 (125 to 214)	12.4 (9.6 to 15.8)	1013 (791 to 1261)	74.2 (59.7 to 91)	134 (102 to 172)	11 (8.4 to 13.9)	4580 (3427 to 5978)	310.3 (236.4 to 399.6)	90 (59 to 129)	6.6 (4.4 to 9.3)	4670 (3494 to 6107)	316.8 (242.1 to 408.6)	339 (212 to 547)	13.4 (8.6 to 21.6)	2098 (1414 to 3213)	79.1 (56.1 to 117.5)	267 (169 to 429)	11.7 (7.6 to 18.6)	9169 (5697 to 14972)	317.3 (200.9 to 509.2)	185 (104 to 318)	7 (4.2 to 11.6)	9354 (5800 to 15283)	324.3 (206.4 to 520.4)
	Female	160 (121 to 206)	22.1 (16.8 to 28.2)	987 (772 to 1235)	133.2 (106.5 to 163.8)	129 (98 to 167)	19.1 (14.5 to 24.2)	4438 (3341 to 5795)	560.3 (425.5 to 724.7)	87 (57 to 125)	11.7 (7.7 to 16.5)	4525 (3402 to 5921)	571.9 (434.3 to 741.3)	331 (207 to 537)	23.7 (15 to 38.4)	2057 (1369 to 3164)	142.6 (99.3 to 213.2)	261 (165 to 420)	20.2 (13.1 to 32.3)	8948 (5538 to 14564)	580.7 (366 to 936.5)	180 (101 to 311)	12.5 (7.3 to 20.9)	9128 (5662 to 14866)	593.2 (374.3 to 952.6)
	Male	5 (3 to 9)	0.9 (0.5 to 1.5)	25 (16 to 40)	4.3 (2.8 to 6.7)	5 (3 to 8)	0.9 (0.5 to 1.4)	143 (81 to 241)	22.4 (12.9 to 37.6)	3 (2 to 5)	0.5 (0.3 to 0.9)	146 (83 to 246)	23 (13.2 to 38.3)	8 (4 to 13)	0.7 (0.4 to 1.2)	41 (25 to 67)	3.7 (2.5 to 5.6)	7 (4 to 11)	0.7 (0.4 to 1.1)	221 (121 to 376)	17.7 (10.3 to 29.1)	5 (2 to 8)	0.4 (0.2 to 0.7)	225 (124 to 384)	18.1 (10.6 to 29.8)
Chad	Both	204 (150 to 272)	6.8 (5.1 to 9.1)	1387 (1084 to 1770)	46.4 (36.7 to 58.9)	166 (124 to 220)	5.8 (4.3 to 7.6)	5199 (3773 to 7052)	166.3 (121.6 to 223.4)	115 (72 to 169)	3.8 (2.4 to 5.5)	5313 (3857 to 7194)	170 (124.8 to 228.4)	555 (384 to 752)	8.4 (5.9 to 11.2)	3686 (2681 to 4858)	54.4 (40.9 to 69.4)	406 (285 to 544)	6.7 (4.8 to 8.8)	13471 (9153 to 18610)	187.8 (130.5 to 253.8)	314 (193 to 487)	4.6 (2.9 to 7)	13785 (9380 to 18992)	192.4 (134.1 to 260.5)
	Female	202 (149 to 270)	12.9 (9.6 to 17.1)	1378 (1076 to 1762)	87.9 (69.4 to 111.3)	164 (123 to 219)	10.9 (8.1 to 14.4)	5160 (3742 to 7018)	312.6 (228.9 to 420.3)	114 (71 to 168)	7.1 (4.5 to 10.5)	5273 (3823 to 7159)	319.7 (234.9 to 430.5)	550 (380 to 746)	17.7 (12.5 to 23.6)	3660 (2659 to 4830)	115.1 (86.6 to 147.1)	402 (281 to 540)	14 (10 to 18.6)	13359 (9068 to 18469)	392.9 (272 to 532.3)	311 (191 to 483)	9.7 (6.1 to 14.7)	13670 (9269 to 18850)	402.6 (279.4 to 542.9)
	Male	2 (1 to 2)	0.1 (0.1 to 0.2)	9 (6 to 12)	0.6 (0.4 to 0.8)	1 (1 to 2)	0.1 (0.1 to 0.2)	39 (27 to 57)	2.6 (1.7 to 3.9)	1 (1 to 2)	0.1 (0 to 0.1)	40 (27 to 58)	2.7 (1.8 to 4)	5 (3 to 7)	0.1 (0.1 to 0.2)	26 (18 to 36)	0.7 (0.5 to 1)	4 (3 to 6)	0.1 (0.1 to 0.2)	112 (76 to 162)	3.2 (2.1 to 4.7)	3 (2 to 4)	0.1 (0 to 0.1)	115 (78 to 166)	3.3 (2.2 to 4.8)
Chile	Both	1739 (1639 to 1845)	16.7 (15.7 to 17.7)	17048 (15478 to 18863)	164 (148.4 to 182.6)	961 (918 to 1004)	9.8 (9.3 to 10.3)	27095 (25816 to 28407)	252.6 (240.9 to 264.7)	1224 (849 to 1685)	11.6 (8.1 to 15.9)	28319 (26865 to 29653)	264.2 (251.3 to 276.8)	4937 (3837 to 6318)	21.2 (16.4 to 27.1)	48329 (39563 to 59222)	206.3 (168.6 to 253.4)	1831 (1667 to 2003)	7.7 (7 to 8.5)	43499 (39768 to 47499)	187 (171.2 to 203.2)	3534 (2303 to 5062)	15.2 (9.9 to 21.7)	47034 (42569 to 51588)	202.2 (183.5 to 221.8)
	Female	1727 (1626 to 1832)	30.7 (28.8 to 32.5)	16949 (15385 to 18757)	301.6 (273.1 to 334.6)	952 (909 to 995)	17.7 (16.9 to 18.6)	26894 (25625 to 28195)	469.3 (447.7 to 492.3)	1215 (841 to 1672)	21.4 (14.9 to 29.4)	28108 (26666 to 29439)	490.7 (466.1 to 514.2)	4907 (3808 to 6287)	39.4 (30.5 to 50.5)	48062 (39374 to 58932)	383.7 (313.5 to 472.2)	1816 (1651 to 1986)	13.9 (12.7 to 15.2)	43190 (39446 to 47184)	349 (319.4 to 379.4)	3510 (2285 to 5032)	28.2 (18.3 to 40.5)	46700 (42251 to 51248)	377.2 (341.6 to 414.1)

		1990												2019											
		Incidence		Prevalence		Deaths		YLLs (Years of Life Lost)		YLDs (Years Lived with Disability)		DALYs (Disability-Adjusted Life Years)		Incidence		Prevalence		Deaths		YLLs (Years of Life Lost)		YLDs (Years Lived with Disability)		DALYs (Disability-Adjusted Life Years)	
Location	Sex	Number	Rate	Number	Rate	Number	Rate	Number	Rate	Number	Rate	Number	Rate	Number	Rate	Number	Rate	Number	Rate	Number	Rate	Number	Rate	Number	Rate
	Male	13 (11 to 15)	0.3 (0.2 to 0.3)	99 (85 to 118)	2.2 (1.9 to 2.7)	9 (8 to 10)	0.2 (0.2 to 0.2)	201 (176 to 232)	4.3 (3.8 to 5)	9 (6 to 13)	0.2 (0.1 to 0.3)	211 (185 to 242)	4.6 (4 to 5.3)	30 (21 to 42)	0.3 (0.2 to 0.4)	267 (202 to 354)	2.5 (1.9 to 3.3)	16 (12 to 19)	0.2 (0.1 to 0.2)	309 (252 to 371)	2.9 (2.4 to 3.5)	25 (15 to 38)	0.2 (0.1 to 0.4)	334 (271 to 400)	3.1 (2.5 to 3.8)
China	Both	81623 (66873 to 97103)	8.5 (7.1 to 10.1)	769644 (645635 to 895193)	82.5 (69.8 to 95.2)	41804 (34550 to 49509)	4.7 (4 to 5.6)	1379561 (1129052 to 1645184)	139.9 (114.9 to 166.3)	55544 (37300 to 76891)	5.8 (3.9 to 8)	1435105 (1184138 to 1708681)	145.7 (121.1 to 172.6)	375484 (296626 to 469983)	18.3 (14.5 to 22.9)	3528138 (2864089 to 4260071)	171.6 (139.7 to 207)	96306 (77323 to 118090)	4.9 (3.9 to 5.9)	2698059 (2150312 to 3360102)	131.5 (104.9 to 163.6)	259395 (168945 to 367512)	12.7 (8.2 to 17.9)	2957454 (2408511 to 3590166)	144.2 (117.3 to 175)
	Female	81074 (66340 to 96525)	17.1 (14 to 20.3)	764798 (641383 to 889871)	163.4 (137.9 to 189)	41429 (34153 to 49151)	9.2 (7.6 to 10.8)	1368351 (1117748 to 1634935)	282.5 (231.5 to 336.5)	55135 (36999 to 76349)	11.6 (7.8 to 16)	1423486 (1173659 to 1695997)	294 (243.5 to 349.1)	368375 (290086 to 463336)	35.6 (28.1 to 44.8)	3473262 (2806059 to 4203027)	334.3 (270.4 to 404.7)	93499 (74511 to 115420)	9 (7.2 to 11.1)	2623126 (2075330 to 3276201)	253.4 (200.6 to 316.5)	254115 (164595 to 360800)	24.6 (15.9 to 34.9)	2877240 (2323688 to 3513542)	278 (224.3 to 339.9)
	Male	549 (445 to 657)	0.1 (0.1 to 0.1)	4846 (3922 to 6034)	1.1 (0.9 to 1.3)	375 (303 to 452)	0.1 (0.1 to 0.1)	11210 (9063 to 13671)	2.4 (1.9 to 2.8)	409 (274 to 590)	0.1 (0.1 to 0.1)	11618 (9457 to 14003)	2.4 (2 to 2.9)	7110 (5341 to 9084)	0.7 (0.5 to 0.9)	54876 (42454 to 68847)	5.3 (4.2 to 6.7)	2808 (2149 to 3533)	0.3 (0.2 to 0.4)	74933 (56160 to 95078)	7.2 (5.5 to 9.1)	5280 (3307 to 7654)	0.5 (0.3 to 0.7)	80213 (61437 to 100882)	7.7 (6 to 9.6)
Colombia	Both	3015 (2846 to 3195)	15.1 (14.2 to 16)	23739 (22171 to 25278)	117.3 (109.5 to 125)	1446 (1384 to 1512)	7.8 (7.4 to 8.1)	45672 (43823 to 47686)	217.2 (208.1 to 226.9)	1747 (1248 to 2366)	8.6 (6.1 to 11.6)	47419 (45444 to 49651)	225.8 (216.1 to 236.2)	11162 (8522 to 14373)	21.2 (16.2 to 27.3)	96864 (75929 to 122446)	184.3 (144.2 to 233)	3401 (2645 to 4331)	6.4 (5 to 8.1)	95283 (72988 to 123845)	181 (139 to 235.3)	6770 (4477 to 9717)	12.9 (8.5 to 18.6)	102053 (78322 to 132897)	193.8 (149 to 252.2)
	Female	2997 (2828 to 3177)	29.2 (27.6 to 31)	23629 (22051 to 25173)	228.3 (213 to 243.6)	1433 (1371 to 1498)	14.9 (14.2 to 15.6)	45326 (43451 to 47352)	421.9 (404.1 to 440.9)	1736 (1240 to 2352)	16.8 (11.9 to 22.6)	47062 (45058 to 49297)	438.7 (419.6 to 458.9)	11117 (8483 to 14317)	39.4 (30.1 to 50.8)	96547 (75683 to 122075)	342.9 (267.6 to 434.1)	3376 (2627 to 4297)	11.7 (9.1 to 15)	94707 (72566 to 123199)	337 (258.8 to 439)	6740 (4457 to 9678)	24 (15.8 to 34.6)	101447 (77878 to 132064)	360.9 (276.9 to 470.1)
	Male	18 (15 to 21)	0.2 (0.2 to 0.2)	109 (92 to 129)	1.1 (1 to 1.4)	13 (11 to 14)	0.2 (0.1 to 0.2)	346 (304 to 395)	3.6 (3.1 to 4.2)	11 (7 to 15)	0.1 (0.1 to 0.2)	357 (313 to 406)	3.7 (3.3 to 4.3)	46 (32 to 63)	0.2 (0.1 to 0.3)	318 (223 to 440)	1.3 (0.9 to 1.8)	24 (18 to 33)	0.1 (0.1 to 0.1)	576 (414 to 786)	2.4 (1.7 to 3.2)	30 (18 to 46)	0.1 (0.1 to 0.2)	606 (437 to 825)	2.5 (1.8 to 3.4)
Comoros	Both	23 (11 to 33)	9.8 (5.2 to 13.7)	145 (81 to 198)	60.9 (36.5 to 81.9)	19 (10 to 27)	8.6 (4.9 to 11.9)	570 (251 to 841)	231.2 (110.3 to 334.4)	12 (6 to 19)	5.3 (2.7 to 7.9)	583 (257 to 860)	236.5 (112.8 to 340.2)	78 (57 to 103)	15.1 (11.3 to 19.7)	522 (394 to 671)	97.7 (74.9 to 124)	56 (43 to 73)	11.5 (8.9 to 14.9)	1636 (1181 to 2187)	299.7 (220.3 to 401.4)	44 (28 to 65)	8.3 (5.3 to 12)	1680 (1208 to 2237)	308 (225.3 to 409.9)
	Female	21 (10 to 30)	16.9 (8.7 to 24.3)	135 (76 to 187)	109.3 (65.1 to 148.8)	17 (9 to 24)	14.4 (7.8 to 20.4)	526 (229 to 787)	408.4 (189.8 to 601)	11 (5 to 17)	9.2 (4.5 to 13.8)	537 (235 to 803)	417.5 (195.8 to 613.4)	74 (53 to 99)	26.5 (19.4 to 35.1)	501 (373 to 652)	176.6 (134.1 to 228.3)	53 (39 to 69)	19.7 (14.8 to 25.8)	1557 (1103 to 2112)	539.8 (384.8 to 725)	42 (26 to 62)	14.7 (9.2 to 21.6)	1598 (1128 to 2158)	554.5 (396.9 to 744.4)
	Male	2 (1 to 4)	2.3 (1.2 to 3.6)	10 (5 to 15)	9.5 (5.2 to 14.4)	2 (1 to 3)	2.4 (1.3 to 3.8)	45 (20 to 75)	43.7 (21.3 to 71.4)	1 (0 to 2)	1.1 (0.5 to 1.9)	46 (21 to 77)	44.8 (22.1 to 73.1)	4 (2 to 7)	2.2 (1.2 to 3.6)	21 (12 to 35)	10.1 (5.9 to 16.1)	4 (2 to 6)	2.1 (1.1 to 3.5)	80 (40 to 136)	37.6 (19.4 to 63)	2 (1 to 4)	1.1 (0.5 to 2)	82 (41 to 140)	38.8 (19.9 to 64.5)
Congo	Both	201 (139 to 278)	17.4 (12.4 to 23.7)	1239 (893 to 1654)	102.6 (76 to 133.8)	158 (111 to 217)	14.8 (10.6 to 19.9)	5124 (3477 to 7225)	406.5 (281.5 to 561.6)	110 (68 to 163)	9.2 (5.8 to 13.4)	5234 (3549 to 7373)	415.7 (287.6 to 573)	661 (392 to 989)	21.2 (13 to 31)	4435 (2807 to 6464)	134.1 (87.8 to 189.8)	441 (268 to 653)	16.1 (10 to 23.1)	14513 (8498 to 21982)	421.5 (256.4 to 625.7)	380 (210 to 612)	11.6 (6.7 to 18.2)	14893 (8715 to 22497)	433.2 (264.1 to 644.1)
	Female	196 (135 to 272)	30.5 (21.3 to 42)	1214 (872 to 1627)	182.1 (133.7 to 240.6)	154 (107 to 212)	25.5 (18.2 to 34.5)	4998 (3372 to 7076)	725.6 (496.3 to 1008.8)	107 (65 to 159)	16.1 (10.1 to 23.7)	5105 (3457 to 7219)	741.7 (507.3 to 1033.4)	651 (385 to 977)	40.5 (24.5 to 59.7)	4374 (2752 to 6391)	258.9 (167.5 to 369.3)	433 (261 to 643)	29.9 (18.5 to 43.3)	14275 (8263 to 21697)	815.6 (488.8 to 1217.9)	373 (204 to 602)	22.3 (12.6 to 35.4)	14648 (8521 to 22213)	837.9 (503.5 to 1247.9)
	Male	5 (3 to 7)	1 (0.6 to 1.4)	25 (16 to 35)	4.8 (3.3 to 6.8)	4 (3 to 6)	0.9 (0.6 to 1.3)	125 (72 to 185)	23.2 (14 to 33.8)	3 (2 to 4)	0.6 (0.3 to 0.9)	128 (74 to 189)	23.8 (14.4 to 34.5)	10 (7 to 15)	0.8 (0.5 to 1.2)	61 (41 to 87)	4.4 (3.1 to 6.1)	8 (5 to 12)	0.7 (0.5 to 1)	238 (152 to 356)	16.1 (10.4 to 23.2)	6 (4 to 10)	0.5 (0.3 to 0.8)	245 (155 to 365)	16.6 (10.7 to 23.9)
Cook Islands	Both	5 (4 to 7)	37.9 (29.1 to 48.9)	43 (33 to 54)	302.8 (238.6 to 383.4)	3 (2 to 3)	20.6 (16.1 to 26.4)	79 (61 to 103)	559.7 (433.7 to 725.6)	3 (2 to 5)	23.5 (15.2 to 34.2)	82 (63 to 107)	583.3 (450.9 to 752.2)	11 (8 to 14)	47.4 (35 to 60.8)	92 (73 to 113)	402.8 (309 to 503.7)	5 (4 to 6)	20.1 (15.9 to 24.9)	115 (88 to 148)	508.1 (378 to 654.8)	7 (5 to 10)	30.3 (19.9 to 43.6)	122 (93 to 156)	538.5 (401.4 to 692.5)
	Female	5 (4 to 7)	78.2 (60 to 101.2)	42 (33 to 54)	631.2 (497 to 800.5)	3 (2 to 3)	41.6 (32.4 to 53.3)	78 (60 to 102)	1161.7 (895.1 to 1513.1)	3 (2 to 5)	48.7 (31.4 to 70.9)	82 (63 to 106)	1210.4 (932.1 to 1567.3)	11 (8 to 14)	90.9 (67.4 to 117)	91 (73 to 112)	776.6 (596.8 to 970)	5 (4 to 6)	38.2 (30.2 to 47.5)	114 (87 to 147)	976 (727.9 to 1255.9)	7 (5 to 10)	58.3 (38.4 to 83.8)	121 (93 to 155)	1034.3 (773.3 to 1329.8)

		1990												2019											
		Incidence		Prevalence		Deaths		YLLs (Years of Life Lost)		YLDs (Years Lived with Disability)		DALYs (Disability-Adjusted Life Years)		Incidence		Prevalence		Deaths		YLLs (Years of Life Lost)		YLDs (Years Lived with Disability)		DALYs (Disability-Adjusted Life Years)	
Location	Sex	Number	Rate	Number	Rate	Number	Rate	Number	Rate	Number	Rate	Number	Rate	Number	Rate	Number	Rate	Number	Rate	Number	Rate	Number	Rate	Number	Rate
	Male	0 (0 to 0)	0.7 (0.4 to 1)	0 (0 to 0)	4.5 (3.2 to 6.6)	0 (0 to 0)	0.5 (0.3 to 0.7)	1 (0 to 1)	10.2 (6.7 to 15.4)	0 (0 to 0)	0.5 (0.3 to 0.8)	1 (0 to 1)	10.6 (7 to 16)	0 (0 to 0)	0.6 (0.4 to 0.8)	1 (0 to 1)	4.6 (3.5 to 6.1)	0 (0 to 0)	0.3 (0.2 to 0.4)	1 (1 to 1)	7.3 (5.4 to 9.7)	0 (0 to 0)	0.4 (0.3 to 0.7)	1 (1 to 1)	7.7 (5.7 to 10.3)
Costa Rica	Both	382 (351 to 414)	20 (18.4 to 21.6)	3184 (2925 to 3465)	166 (152.7 to 180.3)	140 (131 to 149)	7.7 (7.2 to 8.2)	4211 (3952 to 4485)	214 (200.7 to 227.6)	229 (164 to 312)	11.9 (8.5 to 16.1)	4440 (4152 to 4751)	225.9 (211 to 241.3)	1514 (1159 to 1926)	29.1 (22.4 to 37)	13057 (10269 to 16350)	251.3 (197.6 to 314.1)	438 (345 to 551)	8.5 (6.6 to 10.6)	11240 (8661 to 14421)	215 (166.1 to 275.4)	917 (603 to 1331)	17.7 (11.6 to 25.7)	12157 (9401 to 15609)	232.6 (180 to 298.7)
	Female	376 (346 to 408)	38.5 (35.3 to 41.6)	3147 (2891 to 3424)	321.4 (295.7 to 349.8)	136 (127 to 145)	14.6 (13.5 to 15.5)	4123 (3867 to 4397)	410.8 (385.2 to 437.2)	226 (161 to 307)	22.9 (16.3 to 31.1)	4349 (4065 to 4658)	433.8 (404.9 to 463.7)	1505 (1153 to 1915)	54.2 (41.5 to 68.9)	12994 (10217 to 16270)	468.6 (367.8 to 586.5)	434 (339 to 545)	15.5 (12.1 to 19.5)	11139 (8571 to 14290)	399.7 (307.9 to 512.6)	911 (599 to 1322)	32.9 (21.6 to 47.8)	12050 (9317 to 15485)	432.5 (334.6 to 556.6)
	Male	6 (5 to 7)	0.7 (0.6 to 0.8)	37 (32 to 44)	4.2 (3.5 to 5)	4 (3 to 4)	0.5 (0.4 to 0.5)	87 (77 to 98)	9.9 (8.7 to 11)	4 (3 to 5)	0.4 (0.3 to 0.6)	91 (81 to 102)	10.3 (9 to 11.6)	9 (6 to 12)	0.4 (0.3 to 0.5)	63 (45 to 86)	2.6 (1.9 to 3.6)	5 (3 to 6)	0.2 (0.1 to 0.3)	101 (73 to 135)	4.2 (3.1 to 5.7)	6 (4 to 9)	0.3 (0.2 to 0.4)	107 (78 to 143)	4.5 (3.3 to 6)
Croatia	Both	2234 (2033 to 2439)	36 (32.9 to 39.3)	21131 (19197 to 23181)	336.5 (305.8 to 368.8)	894 (826 to 968)	14.7 (13.6 to 15.9)	22971 (21177 to 24904)	367.4 (339.1 to 397.8)	1520 (1068 to 2035)	24.3 (17.1 to 32.6)	24491 (22666 to 26514)	391.8 (362.3 to 424.7)	2865 (2214 to 3646)	37.3 (28.6 to 47.9)	27552 (22734 to 33601)	356.9 (290.9 to 443.1)	1001 (785 to 1234)	11.6 (9.1 to 14.3)	19851 (15235 to 25057)	258.9 (197.8 to 330.2)	1953 (1289 to 2744)	25.5 (16.5 to 36.4)	21804 (16915 to 27534)	284.4 (219.3 to 361.5)
	Female	2211 (2007 to 2415)	64.2 (58.4 to 70.4)	20931 (19019 to 22994)	598.9 (544.8 to 657.4)	885 (817 to 958)	24.8 (22.9 to 26.8)	22737 (20949 to 24682)	660.4 (610.3 to 716.2)	1502 (1055 to 2011)	43.4 (30.4 to 58.5)	24239 (22428 to 26275)	703.9 (649.9 to 763.9)	2824 (2182 to 3588)	68.3 (52 to 88.3)	27196 (22470 to 33143)	656 (531.6 to 822.3)	987 (774 to 1215)	20 (15.5 to 24.9)	19570 (15041 to 24726)	474.5 (361.4 to 606.2)	1922 (1268 to 2696)	46.8 (30.2 to 67.5)	21492 (16652 to 27067)	521.3 (401.3 to 664.8)
	Male	23 (19 to 29)	0.9 (0.7 to 1.1)	200 (164 to 247)	7.5 (6.2 to 9.2)	9 (8 to 11)	0.4 (0.3 to 0.5)	234 (198 to 277)	8.6 (7.4 to 10.1)	18 (12 to 26)	0.7 (0.5 to 1)	252 (214 to 298)	9.3 (7.9 to 11)	41 (28 to 56)	1.1 (0.8 to 1.6)	355 (261 to 475)	9.9 (7.3 to 13.2)	14 (10 to 18)	0.4 (0.3 to 0.5)	281 (204 to 368)	7.9 (5.8 to 10.4)	32 (20 to 48)	0.9 (0.6 to 1.3)	312 (228 to 411)	8.8 (6.4 to 11.5)
Cuba	Both	2619 (2461 to 2790)	25.5 (23.9 to 27.1)	22260 (20779 to 23879)	215.9 (201.5 to 231.6)	1020 (973 to 1064)	10.1 (9.6 to 10.5)	28110 (26958 to 29388)	272.9 (261.7 to 285.4)	1594 (1135 to 2136)	15.4 (11 to 20.7)	29705 (28350 to 31226)	288.4 (275.1 to 303.2)	5590 (4479 to 6915)	31.1 (25 to 38.6)	48482 (40065 to 58836)	270.9 (223 to 330.2)	1776 (1460 to 2163)	9.4 (7.7 to 11.5)	41352 (33455 to 51181)	233.1 (188.5 to 287.6)	3425 (2334 to 4759)	19.2 (12.9 to 26.7)	44777 (35928 to 55471)	252.3 (202.2 to 311.6)
	Female	2587 (2430 to 2757)	49.7 (46.6 to 53)	22064 (20591 to 23668)	423.4 (394.8 to 454.3)	997 (950 to 1041)	19.4 (18.4 to 20.2)	27679 (26511 to 28950)	532 (509.8 to 556.4)	1575 (1119 to 2108)	30.2 (21.5 to 40.4)	29253 (27913 to 30762)	562.1 (536.1 to 591.3)	5543 (4447 to 6852)	59.4 (47.6 to 73.7)	48153 (39835 to 58409)	518.3 (426.6 to 632.5)	1750 (1438 to 2129)	17.5 (14.4 to 21.4)	40855 (33004 to 50535)	445 (358.9 to 550.3)	3394 (2316 to 4716)	36.6 (24.5 to 51.1)	44249 (35534 to 54782)	481.6 (385.5 to 596.5)
	Male	32 (27 to 37)	0.6 (0.5 to 0.8)	196 (165 to 230)	3.9 (3.2 to 4.5)	22 (19 to 26)	0.5 (0.4 to 0.5)	432 (374 to 493)	8.6 (7.4 to 9.8)	20 (13 to 27)	0.4 (0.3 to 0.5)	451 (391 to 516)	9 (7.8 to 10.3)	47 (34 to 62)	0.5 (0.4 to 0.7)	329 (244 to 428)	3.8 (2.8 to 4.9)	26 (19 to 34)	0.3 (0.2 to 0.4)	497 (370 to 646)	5.7 (4.2 to 7.4)	31 (20 to 47)	0.4 (0.2 to 0.5)	528 (394 to 686)	6 (4.5 to 7.8)
Cyprus	Both	268 (231 to 315)	33.5 (29 to 39.3)	3054 (2642 to 3552)	374.3 (326.2 to 434.1)	106 (92 to 123)	13.8 (12 to 16)	2753 (2356 to 3215)	343.2 (295.2 to 401.7)	207 (143 to 288)	25.5 (17.7 to 35.4)	2961 (2538 to 3457)	368.8 (316.5 to 429.6)	985 (816 to 1185)	53.8 (44.6 to 64.5)	10309 (8903 to 11988)	557.6 (481.7 to 648.6)	221 (191 to 252)	12.2 (10.6 to 13.9)	5018 (4310 to 5808)	275.1 (236.8 to 318.1)	739 (496 to 1043)	40.1 (26.8 to 56.5)	5757 (4911 to 6733)	315.2 (268.2 to 368.2)
	Female	261 (223 to 307)	62.5 (53.9 to 73.5)	2993 (2583 to 3483)	702.8 (612.4 to 815)	102 (88 to 119)	25.5 (21.9 to 29.6)	2659 (2267 to 3110)	638.5 (547.1 to 748.3)	202 (139 to 282)	47.7 (32.8 to 66.3)	2860 (2447 to 3335)	686.2 (588.2 to 800.9)	975 (806 to 1174)	101.3 (83.6 to 122.2)	10206 (8805 to 11891)	1051.8 (904.5 to 1228.2)	217 (188 to 249)	22.1 (19.2 to 25.3)	4953 (4256 to 5746)	516.6 (443.4 to 600.6)	730 (488 to 1033)	75.6 (50.4 to 107.7)	5683 (4840 to 6650)	592.2 (502.3 to 694.1)
	Male	8 (5 to 11)	2.1 (1.4 to 2.9)	61 (43 to 83)	16.2 (11.6 to 21.7)	4 (3 to 6)	1.2 (0.8 to 1.6)	95 (66 to 129)	25.2 (17.7 to 34.1)	6 (3 to 9)	1.5 (0.9 to 2.3)	100 (71 to 136)	26.7 (18.8 to 36.2)	10 (7 to 15)	1.1 (0.8 to 1.6)	104 (77 to 138)	11.6 (8.8 to 15.2)	3 (2 to 4)	0.4 (0.3 to 0.5)	65 (47 to 89)	7.2 (5.4 to 9.9)	9 (5 to 14)	1 (0.6 to 1.5)	73 (54 to 99)	8.2 (6.1 to 11)
Czechia	Both	4422 (4179 to 4685)	33.6 (31.8 to 35.6)	41637 (38360 to 45811)	314.8 (291 to 344.1)	1911 (1828 to 1983)	14.3 (13.7 to 14.9)	46706 (44985 to 48369)	356.6 (343.9 to 369.3)	2985 (2071 to 3997)	22.6 (15.8 to 30.3)	49691 (47558 to 51856)	379.3 (363.4 to 395.4)	5748 (4615 to 7085)	31 (24.8 to 38.3)	59269 (50445 to 70155)	313.7 (264.4 to 372.5)	1904 (1556 to 2285)	9.2 (7.6 to 11.1)	38548 (31436 to 46647)	206.6 (168.2 to 253.1)	4033 (2755 to 5714)	21.7 (14.8 to 30.9)	42582 (34701 to 51934)	228.2 (184.7 to 279.5)
	Female	4398 (4157 to 4661)	60.4 (57.1 to 64.2)	41431 (38169 to 45569)	565.6 (523.2 to 616)	1900 (1818 to 1972)	24.4 (23.4 to 25.3)	46454 (44751 to 48109)	644.8 (621.5 to 667.4)	2966 (2058 to 3969)	40.8 (28.3 to 54.6)	49420 (47298 to 51590)	685.6 (657.7 to 715.7)	5700 (4571 to 7023)	58.3 (46.6 to 72.3)	58833 (50051 to 69624)	589.1 (494.6 to 702.4)	1888 (1544 to 2266)	16.3 (13.3 to 19.7)	38215 (31171 to 46239)	387.5 (314.8 to 477.2)	3994 (2727 to 5666)	40.8 (27.6 to 58.4)	42210 (34331 to 51506)	428.3 (345.8 to 526.6)
	Male	24 (20 to 28)	0.4 (0.4 to 0.5)	206 (174 to 245)	3.7 (3.2 to 4.4)	11 (9 to 12)	0.2 (0.2 to 0.2)	252 (221 to 288)	4.5 (4 to 5.1)	19 (13 to 27)	0.3 (0.2 to 0.5)	272 (238 to 308)	4.9 (4.3 to 5.5)	48 (33 to 64)	0.5 (0.4 to 0.7)	436 (324 to 562)	4.9 (3.7 to 6.2)	16 (12 to 21)	0.2 (0.1 to 0.2)	333 (246 to 432)	3.8 (2.9 to 4.9)	39 (24 to 58)	0.4 (0.3 to 0.6)	372 (276 to 480)	4.3 (3.2 to 5.5)
Democratic People's Republic of Korea	Both	2185 (1539 to 3060)	11.6 (8.3 to 15.9)	19735 (15026 to 26141)	108.2 (84.9 to 140.5)	1151 (824 to 1593)	6.6 (4.8 to 8.8)	38185 (26823 to 54083)	198.6 (141.1 to 278)	1446 (857 to 2241)	7.7 (4.7 to 11.7)	39632 (27993 to 56270)	206.3 (146.6 to 288.9)	4718 (3329 to 6521)	14.2 (10.1 to 19.6)	41190 (30701 to 54201)	124 (92.9 to 162.6)	2384 (1761 to 3161)	7.3 (5.5 to 9.6)	72591 (51252 to 99903)	215.8 (151.4 to 298.5)	3064 (1896 to 4688)	9.2 (5.7 to 14)	75655 (53147 to 103855)	225 (158.5 to 310.2)

		1990												2019											
		Incidence		Prevalence		Deaths		YLLs (Years of Life Lost)		YLDs (Years Lived with Disability)		DALYs (Disability-Adjusted Life Years)		Incidence		Prevalence		Deaths		YLLs (Years of Life Lost)		YLDs (Years Lived with Disability)		DALYs (Disability-Adjusted Life Years)	
Location	Sex	Number	Rate	Number	Rate	Number	Rate	Number	Rate	Number	Rate	Number	Rate	Number	Rate	Number	Rate	Number	Rate	Number	Rate	Number	Rate	Number	Rate
	Female	2166 (1522 to 3033)	20.3 (14.3 to 28.3)	19609 (14914 to 25990)	186.4 (143.2 to 244.7)	1139 (813 to 1578)	11 (8 to 15)	37806 (26524 to 53468)	350.8 (247.3 to 491.3)	1433 (848 to 2225)	13.5 (8.1 to 20.7)	39239 (27704 to 55682)	364.3 (257.5 to 512.3)	4658 (3268 to 6447)	26.6 (18.5 to 37.1)	40801 (30359 to 53795)	231.7 (169.8 to 309.7)	2347 (1726 to 3126)	13 (9.5 to 17.4)	71507 (49968 to 98653)	409.3 (282.5 to 572.4)	3023 (1863 to 4635)	17.3 (10.5 to 26.6)	74530 (52142 to 102531)	426.6 (293 to 595.1)
	Male	19 (12 to 30)	0.3 (0.2 to 0.4)	126 (81 to 197)	1.7 (1.1 to 2.5)	12 (8 to 18)	0.2 (0.1 to 0.3)	380 (243 to 578)	5 (3.3 to 7.3)	13 (7 to 22)	0.2 (0.1 to 0.3)	393 (251 to 597)	5.2 (3.4 to 7.6)	61 (40 to 89)	0.4 (0.3 to 0.6)	389 (253 to 581)	2.6 (1.7 to 3.8)	37 (24 to 54)	0.3 (0.2 to 0.4)	1085 (699 to 1621)	7.2 (4.7 to 10.3)	41 (23 to 64)	0.3 (0.2 to 0.4)	1125 (725 to 1689)	7.4 (4.9 to 10.7)
Democratic Republic of the Congo	Both	2092 (1617 to 2704)	11.6 (9.1 to 14.6)	13635 (10994 to 16959)	72.8 (59.4 to 89.1)	1635 (1260 to 2094)	9.9 (7.7 to 12.3)	55204 (41977 to 71921)	278.2 (214.2 to 357.9)	1158 (757 to 1638)	6.2 (4.1 to 8.6)	56362 (43119 to 73403)	284.3 (217.9 to 366.3)	6528 (4644 to 8824)	16 (11.4 to 21.8)	42883 (31967 to 55497)	98.9 (74.7 to 126.8)	4704 (3342 to 6355)	12.9 (9.2 to 17.5)	151887 (106712 to 203037)	334.4 (237.6 to 451)	3644 (2276 to 5515)	8.5 (5.3 to 12.7)	155532 (109555 to 207861)	342.9 (244.4 to 462)
	Female	2038 (1568 to 2609)	21.1 (16.7 to 26.6)	13344 (10727 to 16477)	133.5 (108.9 to 162.9)	1588 (1220 to 2027)	18 (14.1 to 22.3)	53841 (40797 to 70144)	506.9 (388.6 to 648.9)	1126 (735 to 1592)	11.3 (7.5 to 15.6)	54967 (41764 to 71736)	518.2 (397 to 665.3)	6415 (4506 to 8717)	28.8 (20.2 to 39.5)	42216 (31440 to 54799)	181.2 (136.2 to 234.6)	4612 (3262 to 6237)	22.5 (15.8 to 30.6)	149092 (104824 to 200062)	617.3 (436.8 to 835.8)	3574 (2227 to 5394)	15.4 (9.6 to 23.1)	152667 (107284 to 205032)	632.8 (449.5 to 857.1)
	Male	54 (31 to 118)	0.7 (0.4 to 1.6)	291 (181 to 564)	3.8 (2.4 to 7.2)	47 (28 to 104)	0.7 (0.4 to 1.6)	1364 (785 to 2925)	16.6 (9.6 to 36.6)	32 (16 to 64)	0.4 (0.2 to 0.9)	1396 (810 to 2984)	17 (9.9 to 37.4)	113 (61 to 257)	0.7 (0.4 to 1.6)	668 (403 to 1364)	3.8 (2.4 to 7.5)	92 (50 to 212)	0.6 (0.3 to 1.5)	2795 (1534 to 6245)	14.5 (7.9 to 33.5)	70 (35 to 148)	0.4 (0.2 to 0.9)	2865 (1574 to 6406)	14.9 (8.1 to 34.5)
Denmark	Both	3634 (3460 to 3816)	50.8 (48.3 to 53.3)	36254 (33118 to 40022)	498.2 (456.9 to 543.6)	1667 (1590 to 1733)	21.9 (20.9 to 22.7)	39551 (38112 to 41002)	565.3 (545 to 585.4)	2601 (1831 to 3536)	36.2 (25.5 to 49.3)	42152 (40434 to 43969)	601.5 (578.1 to 627.6)	4290 (3313 to 5449)	43.7 (33.5 to 56.1)	46716 (39169 to 56034)	463.5 (383 to 566.6)	1399 (1243 to 1543)	12.3 (11 to 13.5)	27222 (24693 to 29536)	273.9 (250.7 to 296.9)	3228 (2163 to 4651)	32.7 (21.6 to 47.6)	30451 (27299 to 33847)	306.6 (275.7 to 339.8)
	Female	3616 (3444 to 3797)	95.6 (90.8 to 100.5)	36089 (32958 to 39858)	932.2 (855.4 to 1015.3)	1659 (1581 to 1724)	39.5 (37.9 to 41)	39370 (37921 to 40828)	1073.9 (1035 to 1112.9)	2586 (1820 to 3518)	68.2 (47.8 to 93)	41955 (40237 to 43783)	1142.1 (1097.3 to 1191.5)	4266 (3293 to 5419)	84.5 (64.6 to 108.7)	46470 (38962 to 55742)	893.7 (736.5 to 1089.8)	1391 (1235 to 1536)	22.8 (20.5 to 24.9)	27070 (24560 to 29388)	528 (483.5 to 572.9)	3207 (2151 to 4623)	63.2 (41.8 to 92.5)	30277 (27127 to 33655)	591.2 (531.7 to 655.7)
	Male	18 (15 to 22)	0.5 (0.5 to 0.6)	165 (139 to 198)	4.9 (4.2 to 5.9)	8 (7 to 10)	0.3 (0.2 to 0.3)	181 (162 to 205)	5.5 (4.9 to 6.2)	15 (10 to 22)	0.4 (0.3 to 0.6)	196 (175 to 222)	5.9 (5.3 to 6.7)	24 (17 to 34)	0.5 (0.3 to 0.7)	246 (188 to 325)	4.9 (3.8 to 6.3)	8 (6 to 10)	0.2 (0.1 to 0.2)	153 (124 to 185)	3.2 (2.7 to 3.8)	21 (13 to 33)	0.4 (0.3 to 0.6)	174 (141 to 213)	3.6 (3 to 4.4)
Djibouti	Both	16 (10 to 22)	9.4 (6.2 to 12.6)	107 (74 to 142)	60 (44.1 to 76.9)	12 (8 to 16)	8.1 (5.4 to 10.8)	412 (259 to 577)	209.2 (134.3 to 286.2)	9 (5 to 14)	5.1 (3.1 to 7.5)	421 (265 to 589)	214.4 (139.3 to 293.2)	100 (66 to 145)	14.2 (10.2 to 19.4)	698 (466 to 992)	91.9 (66.6 to 123.8)	65 (44 to 92)	10.7 (8.1 to 14.3)	2125 (1348 to 3140)	267.3 (183.2 to 374.3)	59 (34 to 91)	7.9 (4.9 to 11.8)	2184 (1389 to 3216)	275.2 (188.5 to 385.5)
	Female	15 (9 to 20)	16.8 (10.9 to 22.9)	100 (69 to 133)	112.4 (82.2 to 144.9)	11 (7 to 15)	13.9 (9.2 to 18.6)	382 (239 to 536)	389.4 (252.3 to 535.8)	8 (5 to 13)	9.3 (5.6 to 13.7)	390 (245 to 547)	398.7 (259 to 547.5)	94 (60 to 137)	27.6 (19 to 37.8)	662 (439 to 942)	185.3 (131.8 to 250.7)	60 (40 to 85)	20 (14.6 to 27.1)	1992 (1246 to 2957)	529.9 (353.4 to 754.4)	55 (31 to 86)	15.5 (9.4 to 23.3)	2048 (1280 to 3025)	545.4 (365 to 772)
	Male	1 (1 to 2)	2.3 (1.3 to 3.9)	7 (4 to 11)	10.4 (6.4 to 16.9)	1 (1 to 2)	2.4 (1.4 to 4)	30 (16 to 53)	44.2 (23.6 to 77.1)	1 (0 to 1)	1.2 (0.6 to 2)	31 (16 to 54)	45.4 (24.3 to 79.3)	7 (3 to 12)	2.5 (1.3 to 4.4)	36 (20 to 63)	12.3 (7.3 to 20.5)	5 (3 to 10)	2.3 (1.3 to 4.1)	133 (67 to 244)	43.1 (22.1 to 77.5)	4 (2 to 7)	1.3 (0.6 to 2.5)	136 (69 to 250)	44.5 (22.9 to 79.4)
Dominica	Both	20 (18 to 23)	31.8 (27 to 36.6)	161 (140 to 184)	252.3 (217 to 291)	11 (9 to 12)	16.3 (14 to 18.5)	271 (231 to 313)	435.8 (369.7 to 506.7)	12 (8 to 16)	18.5 (12.9 to 25.3)	283 (240 to 326)	454.3 (385.6 to 526.8)	28 (23 to 35)	33 (26.3 to 41.4)	220 (180 to 272)	258.8 (211 to 321.1)	14 (12 to 17)	16.1 (13.2 to 19.8)	346 (274 to 436)	412.4 (324 to 523.5)	16 (11 to 23)	19.1 (12.8 to 27)	362 (287 to 457)	431.5 (341 to 547.5)
	Female	20 (17 to 23)	56.6 (48 to 65.2)	160 (139 to 183)	452.4 (387.3 to 521.5)	11 (9 to 12)	28 (24 to 31.9)	268 (228 to 309)	785.7 (666.2 to 919.3)	12 (8 to 16)	33.1 (23.2 to 45.3)	279 (237 to 322)	818.8 (694.2 to 954.4)	28 (22 to 35)	65.4 (52 to 82.4)	218 (179 to 270)	520.2 (422 to 646.4)	14 (11 to 17)	30.8 (25.1 to 38.4)	341 (271 to 431)	824.4 (645.5 to 1047)	16 (11 to 23)	38.1 (25.5 to 54.3)	357 (283 to 452)	862.5 (678.5 to 1098.3)
	Male	0 (0 to 0)	0.7 (0.6 to 0.9)	1 (1 to 2)	4.1 (3.4 to 5.1)	0 (0 to 0)	0.5 (0.4 to 0.7)	3 (3 to 4)	11.2 (9.1 to 13.8)	0 (0 to 0)	0.4 (0.3 to 0.6)	3 (3 to 4)	11.6 (9.4 to 14.2)	0 (0 to 0)	0.7 (0.5 to 0.9)	2 (1 to 2)	4.2 (3.2 to 5.6)	0 (0 to 0)	0.5 (0.4 to 0.7)	5 (3 to 6)	10.5 (7.5 to 14.1)	0 (0 to 0)	0.4 (0.3 to 0.6)	5 (3 to 6)	10.9 (7.9 to 14.7)
Dominican Republic	Both	397 (342 to 458)	9.3 (8.1 to 10.6)	3362 (2944 to 3840)	79.3 (69.3 to 90.1)	224 (196 to 254)	5.7 (5 to 6.5)	7448 (6463 to 8527)	165.2 (143.1 to 188.1)	240 (164 to 330)	5.6 (3.8 to 7.6)	7688 (6638 to 8808)	170.8 (147.4 to 194.7)	1850 (1321 to 2499)	18.6 (13.4 to 25)	15281 (11292 to 20161)	153.1 (114.5 to 200.6)	798 (590 to 1044)	8.4 (6.3 to 10.9)	23934 (16968 to 32280)	236.5 (168.9 to 317.5)	1103 (698 to 1655)	11 (7 to 16.5)	25037 (17808 to 33618)	247.6 (177.5 to 330.7)

		1990												2019											
		Incidence		Prevalence		Deaths		YLLs (Years of Life Lost)		YLDs (Years Lived with Disability)		DALYs (Disability-Adjusted Life Years)		Incidence		Prevalence		Deaths		YLLs (Years of Life Lost)		YLDs (Years Lived with Disability)		DALYs (Disability-Adjusted Life Years)	
Location	Sex	Number	Rate	Number	Rate	Number	Rate	Number	Rate	Number	Rate	Number	Rate	Number	Rate	Number	Rate	Number	Rate	Number	Rate	Number	Rate	Number	Rate
	Female	392 (337 to 451)	18 (15.6 to 20.6)	3330 (2914 to 3804)	154.4 (135 to 175.5)	220 (192 to 250)	11 (9.6 to 12.4)	7335 (6353 to 8411)	318.8 (275.6 to 364)	237 (162 to 326)	10.8 (7.4 to 14.7)	7572 (6538 to 8680)	329.7 (284.4 to 375.8)	1826 (1302 to 2460)	36.1 (25.9 to 48.4)	15132 (11178 to 19930)	298.6 (222.8 to 391)	782 (577 to 1024)	15.9 (11.8 to 20.8)	23535 (16675 to 31778)	459.7 (326.8 to 618.1)	1088 (690 to 1633)	21.4 (13.6 to 32)	24623 (17539 to 33003)	481.1 (344.5 to 643.5)
	Male	5 (4 to 7)	0.3 (0.2 to 0.4)	33 (26 to 41)	1.7 (1.3 to 2.1)	4 (3 to 5)	0.3 (0.2 to 0.3)	113 (87 to 144)	5.6 (4.3 to 7.1)	3 (2 to 4)	0.2 (0.1 to 0.2)	116 (90 to 149)	5.7 (4.5 to 7.3)	24 (13 to 38)	0.5 (0.3 to 0.8)	149 (87 to 236)	3.2 (1.9 to 5)	16 (9 to 25)	0.4 (0.2 to 0.6)	399 (221 to 649)	8.5 (4.7 to 13.6)	15 (8 to 25)	0.3 (0.2 to 0.5)	414 (230 to 671)	8.8 (4.9 to 14)
Ecuador	Both	473 (440 to 505)	7.9 (7.4 to 8.5)	3630 (3359 to 3943)	60.3 (55.8 to 65.7)	298 (279 to 318)	5.3 (4.9 to 5.6)	9483 (8856 to 10096)	151 (141.2 to 160.9)	271 (193 to 362)	4.5 (3.2 to 6)	9753 (9120 to 10389)	155.4 (145.3 to 165.7)	2553 (1973 to 3323)	16.2 (12.5 to 21)	20845 (16559 to 26655)	130.8 (104.2 to 166.9)	1044 (818 to 1346)	6.9 (5.4 to 8.8)	30145 (23127 to 39302)	188.1 (144.9 to 244.3)	1508 (975 to 2175)	9.5 (6.2 to 13.6)	31654 (24201 to 41342)	197.6 (151.9 to 257.1)
	Female	463 (431 to 496)	15.3 (14.3 to 16.4)	3575 (3309 to 3885)	117.6 (108.6 to 128.2)	290 (271 to 309)	10.2 (9.5 to 10.9)	9277 (8654 to 9879)	292.2 (273 to 311.4)	265 (188 to 355)	8.6 (6.1 to 11.6)	9542 (8915 to 10170)	300.8 (281.2 to 320.9)	2538 (1961 to 3304)	31 (24 to 40.3)	20738 (16473 to 26532)	252 (200.6 to 322)	1034 (811 to 1333)	13.1 (10.3 to 16.7)	29922 (22975 to 39044)	361.8 (278.5 to 470.5)	1498 (969 to 2164)	18.2 (11.8 to 26.2)	31420 (24034 to 41071)	380.1 (291.9 to 495.2)
	Male	10 (8 to 11)	0.4 (0.3 to 0.4)	55 (47 to 64)	2 (1.7 to 2.3)	8 (7 to 10)	0.3 (0.3 to 0.4)	206 (177 to 239)	7.3 (6.3 to 8.6)	6 (4 to 8)	0.2 (0.1 to 0.3)	212 (181 to 246)	7.5 (6.4 to 8.8)	15 (10 to 22)	0.2 (0.1 to 0.3)	107 (77 to 151)	1.4 (1 to 2)	10 (7 to 14)	0.1 (0.1 to 0.2)	223 (157 to 321)	3 (2.1 to 4.3)	10 (6 to 15)	0.1 (0.1 to 0.2)	233 (164 to 334)	3.1 (2.2 to 4.4)
Egypt	Both	2519 (2294 to 2759)	7.1 (6.5 to 7.7)	23693 (21100 to 26603)	70.4 (62.9 to 80)	1588 (1457 to 1730)	4.8 (4.4 to 5.2)	55624 (50598 to 61008)	149.5 (136.9 to 163.3)	1685 (1165 to 2327)	4.8 (3.3 to 6.6)	57309 (52073 to 62758)	154.3 (141.2 to 168.2)	10637 (7384 to 14572)	13.6 (9.4 to 18.6)	93577 (69435 to 123123)	121.6 (91.8 to 157.8)	4672 (3173 to 6369)	6.4 (4.4 to 8.7)	156733 (108406 to 216177)	195.7 (134.2 to 267.5)	7009 (4271 to 10483)	9 (5.5 to 13.2)	163742 (113133 to 224191)	204.7 (140.4 to 278.8)
	Female	2509 (2285 to 2749)	14.2 (13 to 15.5)	23599 (21009 to 26486)	140.8 (125.7 to 159.8)	1581 (1450 to 1723)	9.6 (8.8 to 10.4)	55395 (50382 to 60797)	300.3 (275 to 328.1)	1677 (1160 to 2316)	9.6 (6.6 to 13.2)	57072 (51856 to 62539)	309.9 (283.4 to 338.1)	10600 (7356 to 14525)	29.3 (20.1 to 40)	93258 (69133 to 122756)	262.8 (199.5 to 339.5)	4650 (3159 to 6344)	14.2 (9.6 to 19.2)	156108 (107927 to 215267)	417 (286.2 to 570.2)	6981 (4254 to 10451)	19.3 (11.9 to 28.4)	163089 (112703 to 223017)	436.2 (299.4 to 592)
	Male	10 (7 to 12)	0.1 (0 to 0.1)	94 (71 to 120)	0.6 (0.5 to 0.8)	7 (6 to 9)	0.1 (0 to 0.1)	229 (179 to 281)	1.3 (1 to 1.6)	7 (5 to 11)	0 (0 to 0.1)	237 (186 to 290)	1.3 (1 to 1.7)	37 (22 to 54)	0.1 (0.1 to 0.1)	319 (221 to 445)	0.9 (0.6 to 1.2)	22 (13 to 32)	0.1 (0 to 0.1)	625 (382 to 918)	1.6 (1 to 2.3)	28 (16 to 46)	0.1 (0 to 0.1)	653 (399 to 961)	1.7 (1 to 2.4)
El Salvador	Both	236 (209 to 262)	7.3 (6.5 to 8.2)	1926 (1719 to 2136)	60.3 (53.9 to 66.9)	131 (118 to 146)	4.2 (3.8 to 4.7)	4244 (3788 to 4732)	128.3 (114.3 to 143.2)	139 (95 to 189)	4.3 (2.9 to 5.9)	4382 (3907 to 4897)	132.6 (118.2 to 148.1)	1020 (738 to 1375)	17.4 (12.6 to 23.5)	8466 (6351 to 11223)	144.8 (108.2 to 191.9)	373 (278 to 489)	6.3 (4.7 to 8.2)	10507 (7614 to 14184)	179.2 (129.9 to 241.9)	609 (376 to 899)	10.4 (6.4 to 15.4)	11116 (8050 to 14929)	189.6 (137.7 to 254.4)
	Female	234 (207 to 261)	13.7 (12.1 to 15.3)	1916 (1709 to 2125)	112.9 (100.7 to 125.3)	130 (117 to 145)	7.8 (7 to 8.7)	4207 (3750 to 4695)	240.2 (213.7 to 268.2)	138 (94 to 188)	8 (5.5 to 11)	4344 (3870 to 4859)	248.2 (221 to 277.7)	1014 (733 to 1368)	30.1 (21.7 to 40.7)	8428 (6325 to 11172)	251.1 (187.8 to 332.9)	370 (275 to 484)	10.8 (8 to 14.1)	10418 (7537 to 14069)	309.7 (224.4 to 418)	605 (373 to 894)	18 (11.1 to 26.7)	11023 (7984 to 14803)	327.7 (237.8 to 440.3)
	Male	2 (1 to 2)	0.1 (0.1 to 0.1)	10 (9 to 12)	0.6 (0.6 to 0.8)	1 (1 to 1)	0.1 (0.1 to 0.1)	37 (33 to 42)	2.4 (2.1 to 2.7)	1 (1 to 1)	0.1 (0 to 0.1)	38 (34 to 43)	2.4 (2.1 to 2.8)	6 (4 to 8)	0.2 (0.2 to 0.3)	38 (27 to 51)	1.5 (1.1 to 2)	4 (3 to 5)	0.1 (0.1 to 0.2)	89 (65 to 120)	3.5 (2.5 to 4.8)	4 (2 to 6)	0.2 (0.1 to 0.2)	93 (68 to 125)	3.7 (2.7 to 5)
Equatorial Guinea	Both	21 (14 to 29)	9.5 (6.2 to 13.4)	132 (97 to 172)	59.5 (44.8 to 76.9)	17 (11 to 24)	8.5 (5.6 to 11.9)	573 (372 to 814)	240.6 (157.2 to 341.3)	11 (7 to 17)	5.1 (3.1 to 7.5)	584 (379 to 830)	245.7 (160.6 to 348.5)	127 (70 to 212)	22.8 (13.1 to 36.9)	880 (517 to 1447)	147.4 (90.8 to 234.2)	77 (43 to 126)	16 (9.4 to 25.4)	2372 (1284 to 4050)	388.3 (216.8 to 644)	74 (38 to 132)	12.6 (6.9 to 21.5)	2446 (1329 to 4158)	400.9 (222.7 to 663.3)
	Female	20 (13 to 29)	16.2 (10.5 to 23.1)	128 (94 to 169)	102.7 (76.6 to 134.4)	17 (11 to 24)	14.2 (9 to 20.3)	553 (353 to 793)	415.8 (267.7 to 593.9)	11 (7 to 16)	8.7 (5.2 to 12.9)	564 (359 to 810)	424.4 (273.2 to 607.1)	125 (69 to 210)	39 (22.1 to 63.9)	869 (508 to 1430)	255.8 (155.2 to 409.6)	75 (42 to 124)	26.7 (15.5 to 42.6)	2336 (1253 to 3992)	673.7 (370.7 to 1130)	73 (38 to 131)	21.7 (11.8 to 37.4)	2409 (1297 to 4115)	695.5 (381.1 to 1156.9)
	Male	1 (0 to 1)	0.8 (0.5 to 1.3)	3 (2 to 6)	3.7 (2.5 to 5.8)	1 (0 to 1)	0.8 (0.4 to 1.3)	19 (11 to 33)	19.3 (11.3 to 32.6)	0 (0 to 1)	0.4 (0.2 to 0.8)	20 (12 to 34)	19.7 (11.6 to 33.4)	2 (1 to 3)	0.8 (0.5 to 1.3)	11 (7 to 17)	4.7 (3 to 7)	1 (1 to 2)	0.7 (0.4 to 1.1)	36 (20 to 59)	14.8 (8.7 to 23.3)	1 (1 to 2)	0.5 (0.3 to 0.8)	37 (21 to 60)	15.4 (9.1 to 24)
Eritrea	Both	120 (73 to 173)	10.1 (6.3 to 14.3)	731 (487 to 1009)	60.6 (42.8 to 81.2)	99 (61 to 144)	9.1 (5.8 to 13.1)	3450 (2059 to 5096)	253.7 (157.1 to 367.3)	66 (37 to 104)	5.4 (3.2 to 8.3)	3516 (2101 to 5187)	259.1 (160.2 to 374.8)	534 (380 to 750)	17.2 (12.6 to 23.6)	3486 (2591 to 4765)	105.4 (81.4 to 138.2)	385 (279 to 536)	14 (10.4 to 19.1)	12728 (8878 to 18107)	363.4 (264.2 to 503.1)	299 (185 to 452)	9.2 (5.9 to 13.6)	13027 (9108 to 18510)	372.6 (270.7 to 515)
	Female	112 (65 to 166)	16 (9.5 to 23.7)	698 (449 to 975)	99.4 (67.8 to 136.1)	92 (53 to 137)	14 (8.5 to 20.9)	3242 (1857 to 4879)	419.5 (244.2 to 626.9)	62 (33 to 100)	8.7 (4.8 to 13.7)	3304 (1891 to 4966)	428.2 (249.3 to 640)	512 (360 to 726)	28.7 (20.6 to 39.9)	3369 (2470 to 4671)	181.3 (137.3 to 243.6)	366 (260 to 516)	22.5 (16.2 to 31.2)	12202 (8405 to 17448)	628.6 (443.1 to 890)	286 (174 to 434)	15.5 (9.7 to 23.6)	12488 (8607 to 17849)	644.1 (454.3 to 911.8)

		1990												2019											
		Incidence		Prevalence		Deaths		YLLs (Years of Life Lost)		YLDs (Years Lived with Disability)		DALYs (Disability-Adjusted Life Years)		Incidence		Prevalence		Deaths		YLLs (Years of Life Lost)		YLDs (Years Lived with Disability)		DALYs (Disability-Adjusted Life Years)	
Location	Sex	Number	Rate	Number	Rate	Number	Rate	Number	Rate	Number	Rate	Number	Rate	Number	Rate	Number	Rate	Number	Rate	Number	Rate	Number	Rate	Number	Rate
	Male	8 (4 to 13)	2.3 (1.1 to 3.6)	34 (19 to 53)	8.9 (5.3 to 13.1)	7 (4 to 12)	2.4 (1.2 to 3.7)	208 (105 to 335)	49.4 (24.7 to 77.2)	4 (2 to 7)	1.2 (0.5 to 1.9)	213 (107 to 342)	50.6 (25.3 to 78.8)	23 (13 to 36)	2.5 (1.4 to 3.8)	117 (71 to 184)	11 (6.8 to 16.5)	19 (11 to 30)	2.4 (1.3 to 3.7)	526 (303 to 843)	46.5 (26.4 to 72.6)	13 (6 to 22)	1.3 (0.6 to 2.2)	539 (309 to 863)	47.8 (27 to 74.9)
Estonia	Both	604 (562 to 647)	30.7 (28.6 to 32.9)	5873 (5358 to 6483)	296.6 (270.6 to 325.9)	265 (249 to 280)	13.3 (12.5 to 14.1)	7197 (6771 to 7639)	367.6 (346 to 389.6)	415 (296 to 562)	21.1 (15.1 to 28.4)	7612 (7164 to 8111)	388.6 (365.7 to 413.3)	824 (635 to 1047)	36.9 (28.2 to 47.1)	8192 (6675 to 9904)	361.2 (289.3 to 442.4)	260 (205 to 327)	10.3 (8.1 to 13)	5548 (4307 to 7047)	253.9 (195.9 to 326.8)	579 (385 to 832)	25.8 (17 to 37.2)	6126 (4767 to 7824)	279.7 (213.9 to 359.7)
	Female	601 (558 to 643)	52.4 (48.4 to 56.3)	5851 (5335 to 6460)	500.2 (456.6 to 547.6)	263 (247 to 278)	21.7 (20.4 to 23)	7159 (6735 to 7601)	635.8 (598.3 to 675.3)	413 (295 to 559)	35.8 (25.4 to 48.4)	7572 (7121 to 8071)	671.6 (631.2 to 716.1)	817 (629 to 1038)	65.6 (49.8 to 84.4)	8143 (6629 to 9837)	639.8 (510.1 to 790)	258 (204 to 323)	17.2 (13.4 to 21.7)	5498 (4272 to 6988)	454.8 (349.5 to 588.9)	574 (382 to 825)	45.8 (29.9 to 66.5)	6071 (4717 to 7752)	500.6 (380.6 to 647.4)
	Male	3 (3 to 4)	0.4 (0.4 to 0.5)	22 (19 to 26)	2.9 (2.5 to 3.5)	2 (1 to 2)	0.2 (0.2 to 0.3)	38 (33 to 44)	5.1 (4.5 to 5.8)	2 (2 to 3)	0.3 (0.2 to 0.4)	40 (35 to 46)	5.4 (4.7 to 6.1)	6 (5 to 9)	0.7 (0.5 to 1)	49 (35 to 67)	5.3 (3.8 to 7.2)	3 (2 to 3)	0.3 (0.2 to 0.4)	50 (36 to 67)	5.4 (3.9 to 7.3)	5 (3 to 8)	0.5 (0.3 to 0.8)	55 (39 to 74)	5.9 (4.3 to 7.9)
Eswatini	Both	39 (28 to 51)	12.2 (8.8 to 15.5)	275 (211 to 342)	82.7 (64.6 to 101.5)	30 (21 to 37)	10.1 (7.3 to 12.8)	915 (648 to 1182)	256.7 (185.2 to 326.9)	23 (15 to 32)	6.8 (4.4 to 9.6)	937 (666 to 1213)	263.5 (190.2 to 335.2)	119 (72 to 180)	19.5 (12.3 to 28.5)	826 (532 to 1214)	127.7 (86.6 to 181.7)	80 (50 to 120)	14.8 (9.6 to 21.1)	2392 (1405 to 3712)	357.7 (218.6 to 541)	68 (37 to 111)	10.6 (6.1 to 16.8)	2460 (1442 to 3822)	368.3 (223.9 to 555.2)
	Female	38 (27 to 49)	20.7 (14.7 to 26.3)	267 (202 to 334)	142.6 (109.5 to 176.5)	28 (20 to 36)	16.6 (11.8 to 21.1)	880 (617 to 1140)	445.2 (315.7 to 572.6)	22 (14 to 31)	11.5 (7.4 to 16.3)	901 (636 to 1169)	456.8 (323.2 to 588.8)	116 (68 to 176)	31.6 (19.2 to 47.1)	805 (517 to 1186)	212.5 (140.2 to 305.5)	78 (48 to 117)	23 (14.6 to 33.5)	2311 (1335 to 3591)	597 (355.3 to 918.5)	66 (36 to 108)	17.4 (9.8 to 28)	2377 (1383 to 3697)	614.4 (366.5 to 943.4)
	Male	1 (1 to 2)	1.2 (0.8 to 1.7)	8 (6 to 12)	6.3 (4.3 to 8.5)	1 (1 to 2)	1.1 (0.7 to 1.6)	35 (22 to 51)	24.5 (15.3 to 35)	1 (0 to 1)	0.7 (0.4 to 1.1)	36 (22 to 52)	25.2 (15.8 to 35.9)	4 (2 to 6)	1.5 (0.8 to 2.6)	21 (12 to 34)	8.1 (4.9 to 12.9)	3 (1 to 4)	1.3 (0.7 to 2.1)	81 (41 to 141)	29.8 (15.7 to 50)	2 (1 to 4)	0.9 (0.5 to 1.5)	84 (43 to 144)	30.7 (16.3 to 51.6)
Ethiopia	Both	2740 (2041 to 3465)	12.1 (9.2 to 15.3)	16202 (12324 to 19929)	68.5 (54.5 to 83)	2243 (1694 to 2843)	10.7 (8.3 to 13.8)	76329 (55021 to 97665)	301.9 (227.7 to 381.4)	1492 (973 to 2115)	6.3 (4.2 to 8.9)	77822 (56151 to 99529)	308.3 (232.8 to 389.3)	5900 (4638 to 7416)	12.5 (10.1 to 15.3)	40718 (32450 to 50755)	82.1 (67.1 to 98.5)	4105 (3298 to 4955)	9.7 (8 to 11.6)	126168 (98093 to 157792)	245 (195 to 299.4)	3410 (2291 to 4979)	6.9 (4.8 to 9.8)	129579 (101092 to 161688)	251.9 (201.6 to 306.7)
	Female	2445 (1740 to 3200)	21.2 (15.6 to 27.8)	14917 (10969 to 18715)	126.3 (97.8 to 156.4)	1964 (1413 to 2545)	18.3 (13.4 to 24.4)	69255 (47513 to 90008)	544.2 (390.5 to 705.7)	1338 (830 to 1923)	11.3 (7.3 to 16.3)	70593 (48714 to 91514)	555.4 (399.7 to 721)	5446 (4211 to 6858)	23 (18.4 to 28.1)	38335 (30297 to 47975)	155 (126 to 187)	3702 (2966 to 4509)	17.4 (14.3 to 20.5)	117458 (91110 to 148074)	453.8 (360.7 to 557.5)	3159 (2103 to 4631)	12.8 (8.7 to 18.3)	120617 (93573 to 151557)	466.6 (373 to 569.9)
	Male	296 (220 to 393)	3.3 (2.5 to 4.3)	1286 (980 to 1684)	12.9 (10.1 to 16.6)	279 (205 to 374)	3.3 (2.5 to 4.4)	7074 (5232 to 9331)	68.5 (50.7 to 90.5)	155 (95 to 227)	1.6 (1 to 2.3)	7228 (5363 to 9551)	70.1 (51.9 to 92.6)	454 (284 to 774)	2.5 (1.6 to 4.1)	2383 (1580 to 4009)	12 (8 to 19.6)	404 (259 to 667)	2.3 (1.5 to 3.8)	8711 (5487 to 14700)	43 (27.3 to 72)	251 (133 to 455)	1.3 (0.7 to 2.3)	8962 (5626 to 15071)	44.3 (28.1 to 74.1)
Fiji	Both	103 (80 to 132)	22.3 (17.4 to 28.2)	812 (654 to 1001)	173.8 (143 to 211.6)	64 (50 to 80)	15.7 (12.4 to 19.7)	2246 (1745 to 2851)	452.7 (352.5 to 571.4)	63 (42 to 90)	13.3 (8.9 to 18.6)	2310 (1799 to 2931)	466 (363.7 to 588.9)	251 (185 to 332)	31.2 (23.4 to 40.9)	1927 (1486 to 2478)	230 (181.6 to 291.8)	147 (109 to 193)	20.3 (15.5 to 26.2)	4556 (3348 to 5994)	538 (401.5 to 701.5)	151 (99 to 217)	18.1 (12.1 to 25.7)	4707 (3459 to 6193)	556.1 (415.8 to 722.9)
	Female	102 (79 to 130)	43.6 (33.9 to 55.2)	805 (648 to 993)	340.8 (278.5 to 416.6)	63 (49 to 80)	30.2 (23.7 to 38)	2225 (1725 to 2829)	890.7 (692.8 to 1127.7)	63 (41 to 89)	26 (17.5 to 36.6)	2288 (1784 to 2912)	916.7 (715.4 to 1162.9)	249 (183 to 330)	59.2 (44.2 to 77.7)	1913 (1474 to 2461)	443.2 (345.7 to 567)	145 (108 to 191)	37.1 (28.1 to 48.1)	4517 (3315 to 5956)	1039.6 (770.8 to 1359.4)	149 (99 to 216)	34.8 (23.1 to 49.5)	4667 (3430 to 6155)	1074.4 (796.3 to 1408.8)
	Male	1 (1 to 1)	0.6 (0.4 to 0.9)	7 (5 to 10)	3.9 (2.8 to 5.3)	1 (0 to 1)	0.5 (0.3 to 0.8)	21 (14 to 31)	10.9 (7.2 to 16.3)	1 (0 to 1)	0.4 (0.2 to 0.6)	22 (14 to 32)	11.3 (7.4 to 16.9)	2 (1 to 3)	0.6 (0.4 to 0.9)	14 (10 to 20)	4.2 (3.1 to 5.7)	1 (1 to 2)	0.5 (0.3 to 0.7)	39 (26 to 57)	10.7 (7.2 to 15.5)	1 (1 to 2)	0.4 (0.2 to 0.7)	40 (27 to 60)	11.1 (7.5 to 16.1)
Finland	Both	2527 (2382 to 2667)	37.9 (35.7 to 40)	28905 (26093 to 32323)	427.3 (388.3 to 472.9)	849 (807 to 882)	12.4 (11.8 to 12.9)	21041 (20204 to 21839)	319.1 (306.1 to 331.2)	1966 (1363 to 2713)	29.3 (20.3 to 40.6)	23007 (21911 to 24156)	348.4 (331.8 to 366.3)	4391 (3379 to 5570)	44.1 (33.7 to 56.8)	50725 (42740 to 60396)	488.5 (405.1 to 594.6)	1046 (931 to 1151)	8.9 (8 to 9.7)	20527 (18662 to 22343)	208.5 (190.6 to 225.8)	3428 (2262 to 4894)	33.9 (22 to 49.2)	23955 (21524 to 26714)	242.4 (218.3 to 269.8)
	Female	2516 (2372 to 2655)	69 (64.9 to 72.9)	28809 (26006 to 32214)	761.3 (695.7 to 837.6)	844 (802 to 877)	21.2 (20.2 to 22)	20929 (20094 to 21720)	586.4 (562.4 to 609.8)	1957 (1356 to 2701)	52.8 (36.5 to 73)	22886 (21797 to 24030)	639.2 (608.7 to 671.4)	4371 (3362 to 5543)	84.6 (64.4 to 109.1)	50524 (42565 to 60127)	931.9 (767.5 to 1137.3)	1039 (925 to 1145)	16.2 (14.7 to 17.7)	20402 (18552 to 22217)	400 (366.3 to 433.5)	3410 (2248 to 4870)	65 (41.9 to 94.7)	23812 (21369 to 26551)	465 (419 to 516.3)
	Male	11 (9 to 13)	0.4 (0.3 to 0.5)	97 (82 to 114)	3.5 (3 to 4.2)	5 (4 to 5)	0.2 (0.2 to 0.2)	112 (99 to 127)	4 (3.5 to 4.5)	9 (6 to 13)	0.3 (0.2 to 0.5)	121 (107 to 137)	4.3 (3.8 to 4.9)	20 (14 to 28)	0.4 (0.3 to 0.6)	201 (150 to 264)	4 (3 to 5.2)	6 (5 to 8)	0.1 (0.1 to 0.1)	125 (102 to 152)	2.6 (2.2 to 3.2)	18 (11 to 27)	0.4 (0.2 to 0.5)	142 (116 to 175)	3 (2.4 to 3.6)

		1990												2019											
		Incidence		Prevalence		Deaths		YLLs (Years of Life Lost)		YLDs (Years Lived with Disability)		DALYs (Disability-Adjusted Life Years)		Incidence		Prevalence		Deaths		YLLs (Years of Life Lost)		YLDs (Years Lived with Disability)		DALYs (Disability-Adjusted Life Years)	
Location	Sex	Number	Rate	Number	Rate	Number	Rate	Number	Rate	Number	Rate	Number	Rate	Number	Rate	Number	Rate	Number	Rate	Number	Rate	Number	Rate	Number	Rate
France	Both	30703 (28877 to 32567)	41 (38.7 to 43.5)	341003 (308300 to 381443)	445.9 (407.1 to 492.9)	12633 (11764 to 13219)	15.8 (14.9 to 16.5)	289352 (276954 to 299265)	394.7 (379.7 to 407.4)	23231 (16157 to 32027)	30.8 (21.3 to 42.5)	312582 (297763 to 325414)	425.6 (407.1 to 442.6)	51276 (39714 to 66118)	46.3 (35.4 to 60.2)	588160 (496047 to 705936)	514.7 (425.9 to 633.5)	15326 (13075 to 16868)	11.2 (10 to 12.1)	292998 (266624 to 315901)	266.4 (245.1 to 286.6)	39729 (25708 to 58116)	35.7 (22.8 to 53.1)	332727 (298541 to 365857)	302.1 (274.5 to 332.2)
	Female	30447 (28630 to 32297)	75.4 (71.1 to 80)	338676 (306087 to 379048)	811.2 (742.2 to 892.9)	12515 (11649 to 13102)	27.6 (26.1 to 28.6)	286802 (274447 to 296651)	732.6 (706.3 to 755.2)	23021 (15997 to 31744)	56.5 (38.9 to 78)	309822 (295067 to 322550)	789.1 (756.8 to 820.7)	50715 (39237 to 65459)	86.9 (66.2 to 113.2)	582682 (491130 to 699196)	962.6 (793.1 to 1187.3)	15138 (12908 to 16690)	19.9 (17.9 to 21.5)	289573 (263528 to 312143)	499.4 (460 to 537.2)	39253 (25447 to 57499)	67 (42.6 to 99.5)	328825 (295038 to 361825)	566.3 (514.3 to 623.2)
	Male	257 (219 to 303)	0.8 (0.6 to 0.9)	2327 (1979 to 2727)	6.9 (5.9 to 8.1)	119 (108 to 131)	0.4 (0.3 to 0.4)	2550 (2325 to 2810)	7.6 (6.9 to 8.3)	210 (143 to 299)	0.6 (0.4 to 0.9)	2760 (2515 to 3056)	8.2 (7.4 to 9)	561 (396 to 781)	1 (0.7 to 1.4)	5478 (4196 to 7218)	9.6 (7.4 to 12.6)	188 (151 to 232)	0.3 (0.3 to 0.4)	3426 (2803 to 4115)	6.3 (5.2 to 7.5)	476 (285 to 716)	0.8 (0.5 to 1.3)	3902 (3162 to 4714)	7.1 (5.9 to 8.6)
Gabon	Both	90 (69 to 116)	16 (12.4 to 20.4)	599 (482 to 736)	102.7 (83.3 to 124.8)	70 (54 to 89)	13.3 (10.4 to 16.8)	2039 (1548 to 2629)	341.4 (261.2 to 438.4)	50 (32 to 71)	8.6 (5.6 to 12.1)	2089 (1585 to 2689)	350.1 (267.3 to 447.8)	251 (162 to 356)	22.2 (14.4 to 30.7)	1795 (1238 to 2457)	150.9 (105.3 to 203)	161 (105 to 224)	16 (10.5 to 21.8)	4689 (3006 to 6692)	384.1 (249.1 to 541.1)	145 (86 to 223)	12.3 (7.5 to 18.6)	4834 (3107 to 6896)	396.4 (259.2 to 557.3)
	Female	88 (67 to 112)	28.1 (21.4 to 35.8)	585 (467 to 721)	184.1 (147.2 to 226.2)	68 (52 to 87)	22.6 (17.4 to 28.6)	1978 (1488 to 2560)	618 (466 to 797.5)	49 (31 to 69)	15.3 (10 to 21.7)	2026 (1529 to 2615)	633.3 (476.4 to 815.3)	245 (158 to 349)	40 (25.6 to 56.2)	1761 (1205 to 2420)	279 (193.4 to 378.3)	157 (102 to 220)	27.7 (18 to 38.2)	4575 (2917 to 6568)	706.7 (457 to 1005)	142 (83 to 218)	22.5 (13.5 to 34.3)	4717 (3020 to 6773)	729.2 (470.5 to 1039.2)
	Male	3 (2 to 4)	1 (0.6 to 1.5)	14 (9 to 20)	5.5 (3.5 to 7.5)	2 (1 to 3)	0.9 (0.6 to 1.3)	61 (35 to 90)	22.6 (13.2 to 33.1)	2 (1 to 2)	0.6 (0.3 to 1)	63 (36 to 93)	23.2 (13.6 to 34)	5 (3 to 8)	1.1 (0.7 to 1.7)	34 (21 to 50)	6.4 (4.3 to 9.1)	4 (2 to 6)	0.9 (0.6 to 1.3)	114 (66 to 178)	20.6 (12.4 to 31.7)	3 (2 to 6)	0.7 (0.4 to 1.1)	117 (68 to 183)	21.2 (12.8 to 32.8)
Gambia	Both	18 (13 to 24)	4.4 (3.2 to 5.8)	131 (101 to 169)	32.9 (25.8 to 41.3)	13 (10 to 18)	3.5 (2.7 to 4.6)	433 (304 to 588)	97.7 (69.4 to 131.4)	11 (7 to 16)	2.6 (1.6 to 3.7)	443 (311 to 600)	100.3 (71.2 to 134.4)	96 (62 to 136)	8.9 (5.8 to 12.6)	669 (466 to 916)	60.6 (42.9 to 81.8)	65 (43 to 92)	6.4 (4.2 to 9.1)	2045 (1315 to 2948)	178 (115.4 to 255.3)	56 (32 to 88)	5 (2.9 to 8)	2101 (1356 to 3025)	183 (118.7 to 262)
	Female	17 (12 to 23)	8.9 (6.5 to 12)	127 (97 to 164)	68 (52.9 to 86.6)	13 (9 to 17)	7.1 (5.2 to 9.3)	418 (292 to 570)	204.7 (144.3 to 275.7)	10 (6 to 15)	5.3 (3.3 to 7.7)	428 (300 to 582)	209.9 (148.4 to 281.9)	94 (61 to 134)	17 (11 to 24.1)	656 (455 to 903)	117.6 (82.2 to 159.7)	63 (41 to 91)	12.1 (7.8 to 17.4)	1996 (1281 to 2894)	344.8 (221 to 498)	54 (32 to 86)	9.7 (5.6 to 15.5)	2051 (1312 to 2976)	354.5 (226 to 512.2)
	Male	1 (0 to 1)	0.3 (0.2 to 0.5)	4 (2 to 5)	1.7 (1.2 to 2.5)	1 (0 to 1)	0.3 (0.2 to 0.5)	15 (10 to 23)	7.1 (4.6 to 10.9)	0 (0 to 1)	0.2 (0.1 to 0.3)	16 (10 to 24)	7.3 (4.7 to 11.2)	2 (1 to 3)	0.5 (0.3 to 0.7)	13 (9 to 19)	2.4 (1.6 to 3.5)	2 (1 to 3)	0.4 (0.3 to 0.6)	48 (32 to 74)	9.2 (6 to 14.1)	1 (1 to 2)	0.3 (0.2 to 0.5)	50 (33 to 76)	9.5 (6.2 to 14.5)
Georgia	Both	2324 (2086 to 2565)	38.2 (34.5 to 42.1)	20131 (18201 to 22203)	330.9 (299.2 to 364.1)	1087 (987 to 1191)	17.9 (16.3 to 19.6)	33229 (29923 to 36457)	543.3 (490.7 to 595.2)	1516 (1073 to 2102)	24.9 (17.7 to 34.5)	34746 (31317 to 38197)	568.2 (513.7 to 623.9)	1931 (1591 to 2332)	36.4 (30 to 43.9)	18439 (15859 to 21284)	343.1 (292.8 to 398.2)	905 (755 to 1079)	16 (13.2 to 19.1)	23676 (19610 to 28345)	452.1 (372.4 to 542.9)	1300 (875 to 1784)	24.5 (16.5 to 33.9)	24976 (20575 to 29962)	476.6 (391.6 to 571.4)
	Female	2322 (2084 to 2563)	68 (61.1 to 75)	20115 (18183 to 22186)	585.9 (529.2 to 646.7)	1086 (986 to 1190)	30.8 (28 to 33.7)	33201 (29897 to 36429)	977.6 (881.9 to 1072.7)	1515 (1072 to 2100)	44.4 (31.4 to 61.9)	34716 (31288 to 38167)	1022 (921.4 to 1124.3)	1921 (1582 to 2319)	65.8 (54.2 to 79.5)	18372 (15800 to 21211)	618.2 (524.3 to 719.6)	900 (750 to 1073)	28 (23.1 to 33.5)	23546 (19508 to 28191)	822.2 (676.2 to 988.5)	1293 (870 to 1775)	44.3 (29.7 to 61.2)	24839 (20436 to 29782)	866.6 (709.4 to 1040)
	Male	2 (2 to 2)	0.1 (0.1 to 0.1)	17 (13 to 21)	0.7 (0.5 to 0.8)	1 (1 to 1)	0 (0 to 0)	28 (23 to 33)	1.1 (0.9 to 1.3)	2 (1 to 2)	0.1 (0 to 0.1)	30 (25 to 35)	1.1 (1 to 1.3)	10 (8 to 13)	0.4 (0.3 to 0.6)	67 (50 to 88)	2.9 (2.2 to 3.7)	6 (4 to 7)	0.2 (0.2 to 0.3)	130 (100 to 164)	5.6 (4.3 to 7)	7 (5 to 11)	0.3 (0.2 to 0.5)	137 (106 to 173)	5.9 (4.5 to 7.4)
Germany	Both	46660 (44258 to 49025)	39.8 (37.8 to 41.8)	516030 (465982 to 578689)	432.7 (393.9 to 480.2)	19246 (18204 to 20027)	15.8 (15 to 16.4)	453903 (436579 to 468360)	398.6 (383.6 to 411.1)	35550 (24835 to 48428)	30.1 (21 to 41.1)	489453 (468823 to 509792)	428.6 (410.7 to 445.7)	70173 (53859 to 90348)	44.3 (33.9 to 58)	765284 (639333 to 918572)	470.5 (386.9 to 576.5)	21652 (19398 to 23510)	11.5 (10.5 to 12.4)	417684 (384562 to 446322)	266.4 (248.4 to 283.9)	53047 (35685 to 77083)	33.3 (21.9 to 48.9)	470731 (428737 to 511632)	299.7 (275 to 326)
	Female	46415 (44001 to 48765)	70.8 (67.4 to 74.3)	513707 (463858 to 576093)	758.7 (695.3 to 837.7)	19134 (18096 to 19914)	26.8 (25.6 to 27.8)	451360 (434063 to 465838)	729.5 (703.7 to 752.8)	35344 (24709 to 48190)	53.2 (37.1 to 72.8)	486704 (465991 to 506766)	782.7 (750.9 to 813.7)	69662 (53510 to 89596)	84.7 (64.8 to 111.4)	760184 (634721 to 912523)	897.7 (735.2 to 1104.3)	21486 (19235 to 23348)	21 (19.2 to 22.5)	414466 (381503 to 443160)	510.5 (476.3 to 544.9)	52602 (35409 to 76321)	63.7 (41.8 to 93.8)	467068 (424918 to 508158)	574.1 (525.4 to 625.3)
	Male	245 (211 to 287)	0.5 (0.4 to 0.6)	2323 (1988 to 2734)	4.9 (4.2 to 5.7)	112 (101 to 124)	0.2 (0.2 to 0.3)	2543 (2301 to 2794)	5.2 (4.7 to 5.7)	206 (140 to 291)	0.4 (0.3 to 0.6)	2749 (2475 to 3023)	5.6 (5.1 to 6.2)	512 (362 to 701)	0.6 (0.5 to 0.9)	5100 (3954 to 6545)	6.3 (4.9 to 8.1)	166 (135 to 204)	0.2 (0.2 to 0.2)	3218 (2688 to 3830)	4.2 (3.6 to 4.9)	445 (273 to 681)	0.6 (0.3 to 0.8)	3663 (3035 to 4378)	4.8 (4 to 5.6)

		1990												2019											
		Incidence		Prevalence		Deaths		YLLs (Years of Life Lost)		YLDs (Years Lived with Disability)		DALYs (Disability-Adjusted Life Years)		Incidence		Prevalence		Deaths		YLLs (Years of Life Lost)		YLDs (Years Lived with Disability)		DALYs (Disability-Adjusted Life Years)	
Location	Sex	Number	Rate	Number	Rate	Number	Rate	Number	Rate	Number	Rate	Number	Rate	Number	Rate	Number	Rate	Number	Rate	Number	Rate	Number	Rate	Number	Rate
Ghana	Both	1225 (935 to 1568)	16.6 (12.9 to 20.9)	8102 (6300 to 10197)	107.3 (85.8 to 133.2)	890 (679 to 1130)	13.1 (10.2 to 16.5)	29911 (22380 to 38410)	373.5 (283.1 to 473.5)	689 (447 to 989)	9.1 (6 to 13)	30600 (22896 to 39320)	382.6 (291.2 to 485.6)	4702 (3597 to 6064)	25.1 (19.7 to 31.9)	33710 (26182 to 42940)	173.5 (139.4 to 216)	2902 (2258 to 3703)	17.2 (13.7 to 21.7)	92733 (70057 to 120439)	461 (356.8 to 590.5)	2771 (1771 to 4038)	14.3 (9.4 to 20.6)	95504 (72117 to 124147)	475.3 (367.1 to 609.9)
	Female	1220 (931 to 1562)	32 (24.7 to 40.3)	8074 (6274 to 10164)	207.1 (165.3 to 258.1)	886 (676 to 1125)	24.9 (19.3 to 31.3)	29792 (22258 to 38270)	725.1 (548.9 to 919.5)	686 (445 to 986)	17.5 (11.5 to 25)	30478 (22789 to 39175)	742.6 (564.5 to 944.2)	4691 (3588 to 6049)	45.1 (35.1 to 57.6)	33644 (26121 to 42846)	314.3 (251.4 to 392.4)	2894 (2253 to 3692)	30.3 (24.1 to 38.2)	92505 (69848 to 120156)	837.6 (646.4 to 1077)	2764 (1765 to 4028)	25.8 (16.8 to 37.5)	95269 (71955 to 123925)	863.5 (665.4 to 1110.8)
	Male	5 (3 to 7)	0.1 (0.1 to 0.2)	28 (19 to 39)	0.8 (0.5 to 1.1)	4 (3 to 5)	0.1 (0.1 to 0.2)	119 (81 to 169)	3.2 (2.2 to 4.6)	3 (2 to 5)	0.1 (0.1 to 0.1)	122 (84 to 173)	3.3 (2.2 to 4.7)	10 (7 to 14)	0.1 (0.1 to 0.2)	66 (48 to 89)	0.8 (0.5 to 1)	8 (5 to 10)	0.1 (0.1 to 0.2)	228 (160 to 309)	2.7 (1.9 to 3.6)	7 (4 to 11)	0.1 (0.1 to 0.1)	235 (165 to 318)	2.7 (1.9 to 3.7)
Greece	Both	5293 (4939 to 5664)	38 (35.5 to 40.6)	61254 (54929 to 68199)	429.8 (388.1 to 474.9)	1807 (1712 to 1885)	12.6 (11.9 to 13.1)	45724 (43829 to 47612)	330.9 (317.2 to 344.6)	4134 (2872 to 5620)	29.4 (20.5 to 40)	49859 (47445 to 52442)	360.4 (343.9 to 379.1)	8800 (6819 to 11260)	45.3 (34.6 to 58.8)	95248 (79956 to 115096)	482.7 (396.8 to 590.6)	2822 (2516 to 3080)	11.7 (10.7 to 12.7)	52733 (48453 to 56713)	274.5 (255 to 293.3)	6655 (4470 to 9573)	34.2 (22.8 to 49.8)	59388 (54309 to 65320)	308.7 (283.5 to 337.6)
	Female	5267 (4913 to 5636)	72.2 (67.3 to 77.1)	60985 (54651 to 67872)	810 (732.4 to 894.2)	1796 (1701 to 1874)	23.3 (22.1 to 24.3)	45487 (43582 to 47377)	630.6 (605.3 to 656.5)	4112 (2855 to 5589)	55.6 (38.7 to 75.7)	49599 (47200 to 52165)	686.3 (655.2 to 722.1)	8715 (6756 to 11158)	85.5 (65.1 to 111.3)	94481 (79331 to 114115)	911.4 (748.2 to 1117.6)	2791 (2482 to 3049)	21.6 (19.7 to 23.3)	52185 (47958 to 56154)	518.3 (481.3 to 553.7)	6584 (4422 to 9468)	64.6 (42.8 to 94.2)	58769 (53767 to 64658)	582.9 (535.4 to 637.4)
	Male	26 (22 to 31)	0.4 (0.3 to 0.5)	269 (227 to 321)	4 (3.4 to 4.7)	11 (10 to 12)	0.2 (0.1 to 0.2)	237 (215 to 262)	3.5 (3.2 to 3.8)	23 (15 to 32)	0.3 (0.2 to 0.5)	260 (235 to 290)	3.8 (3.5 to 4.2)	85 (63 to 112)	0.9 (0.6 to 1.1)	766 (592 to 961)	7.7 (5.9 to 9.7)	30 (26 to 35)	0.3 (0.2 to 0.3)	548 (484 to 626)	5.8 (5.2 to 6.6)	71 (44 to 104)	0.7 (0.4 to 1)	619 (540 to 709)	6.5 (5.8 to 7.5)
Greenland	Both	15 (13 to 18)	44.3 (37.3 to 50.8)	120 (103 to 138)	327.5 (284.3 to 372.8)	9 (8 to 11)	31.2 (26.3 to 35.9)	259 (216 to 303)	672.4 (572.9 to 775.1)	10 (7 to 14)	27.2 (18.8 to 36.4)	268 (225 to 315)	699.6 (592.7 to 807.4)	16 (12 to 20)	21.9 (17.3 to 27.7)	156 (130 to 189)	219.7 (184.2 to 262.9)	7 (5 to 8)	9.8 (7.8 to 12.2)	186 (143 to 240)	254.1 (198.2 to 322.1)	11 (7 to 16)	15.6 (10.6 to 22)	198 (151 to 255)	269.7 (209.9 to 343.9)
	Female	10 (8 to 12)	50.6 (41.8 to 61)	92 (78 to 108)	493.3 (424 to 573.6)	5 (4 to 6)	27.9 (23.1 to 33.3)	166 (136 to 202)	814.4 (665.5 to 981)	7 (5 to 9)	35.2 (23.8 to 48.8)	173 (141 to 210)	849.6 (696.2 to 1025.8)	15 (12 to 19)	45.2 (35.3 to 56.9)	151 (125 to 182)	454.1 (379.5 to 540.8)	6 (5 to 8)	19.3 (15.3 to 24)	178 (136 to 228)	522.2 (404.7 to 660.4)	11 (7 to 15)	32.1 (21.6 to 45.3)	188 (144 to 241)	554.3 (427.5 to 705.5)
	Male	5 (4 to 7)	48.2 (38 to 59.8)	28 (22 to 35)	190.6 (148.5 to 238.2)	4 (3 to 5)	43 (33.9 to 53.7)	93 (75 to 112)	666.5 (536.2 to 804)	3 (2 to 4)	23.7 (15.6 to 32.7)	96 (77 to 116)	690.2 (555.8 to 833.8)	1 (0 to 1)	1.9 (1.3 to 2.5)	6 (4 to 7)	16 (12.8 to 19.9)	0 (0 to 1)	1.3 (0.9 to 1.8)	9 (6 to 12)	24.2 (17 to 31.9)	0 (0 to 1)	1.3 (0.8 to 2.2)	9 (6 to 12)	25.6 (17.9 to 33.8)
Grenada	Both	18 (16 to 20)	27.6 (24.3 to 31)	138 (124 to 155)	212.8 (189.1 to 238.6)	10 (9 to 12)	15.1 (13.5 to 16.8)	268 (238 to 300)	420.4 (370 to 471.7)	10 (7 to 14)	16 (11.2 to 21.5)	279 (248 to 311)	436.3 (384.6 to 489.2)	42 (36 to 47)	36.7 (32 to 41.5)	333 (289 to 377)	287.9 (251.8 to 324.7)	18 (16 to 20)	16.5 (14.6 to 18.4)	482 (419 to 547)	417.2 (364.1 to 472.8)	25 (17 to 33)	21.5 (15.1 to 28.6)	506 (441 to 578)	438.7 (383.3 to 499.9)
	Female	18 (16 to 20)	49.4 (43.4 to 55.7)	137 (122 to 153)	384 (340.3 to 430.9)	10 (9 to 11)	26.2 (23.4 to 29.2)	263 (233 to 294)	761.4 (668.7 to 855)	10 (7 to 14)	28.7 (20.2 to 38.8)	274 (243 to 306)	790.1 (695.2 to 887.4)	41 (36 to 47)	70.9 (61.3 to 80.6)	330 (287 to 374)	564.5 (490.6 to 640.1)	17 (15 to 20)	30.1 (26.6 to 33.6)	477 (414 to 542)	816.3 (709.6 to 929.4)	24 (17 to 33)	41.7 (29.3 to 55.7)	501 (437 to 571)	858 (745.2 to 981.8)
	Male	0 (0 to 0)	1 (0.8 to 1.2)	2 (1 to 2)	5.8 (4.8 to 7)	0 (0 to 0)	0.8 (0.7 to 1)	5 (4 to 6)	17.7 (14.4 to 21.2)	0 (0 to 0)	0.6 (0.4 to 0.8)	5 (4 to 6)	18.3 (14.8 to 21.9)	0 (0 to 0)	0.7 (0.5 to 0.8)	3 (2 to 3)	4.8 (3.9 to 5.8)	0 (0 to 0)	0.5 (0.4 to 0.6)	5 (4 to 7)	9.6 (7.8 to 11.7)	0 (0 to 0)	0.4 (0.3 to 0.6)	5 (4 to 7)	10 (8.1 to 12.3)
Guam	Both	13 (11 to 15)	14.6 (12.5 to 16.9)	117 (102 to 135)	131.6 (114.5 to 150.6)	6 (5 to 7)	8.9 (7.7 to 10.4)	188 (160 to 218)	207.4 (177.8 to 240.5)	9 (6 to 12)	9.4 (6.5 to 13)	196 (167 to 229)	216.9 (186 to 251)	35 (29 to 43)	19.1 (15.6 to 23.4)	320 (271 to 379)	171.3 (144.8 to 201.6)	17 (14 to 20)	9.1 (7.5 to 11.1)	454 (369 to 556)	243.7 (199.2 to 296.4)	23 (15 to 33)	12.5 (8.3 to 17.8)	477 (389 to 585)	256.2 (210.1 to 312.5)
	Female	13 (11 to 15)	29.6 (25.4 to 34.1)	117 (102 to 135)	268.7 (233.7 to 307.3)	6 (5 to 7)	17.3 (14.8 to 20)	187 (159 to 218)	423.5 (362.4 to 492)	8 (6 to 12)	19.3 (13.1 to 26.5)	196 (166 to 228)	442.7 (378.4 to 515.3)	35 (29 to 43)	38 (30.9 to 46.7)	320 (270 to 378)	343.2 (289.3 to 404.8)	17 (14 to 20)	17.5 (14.5 to 21.3)	452 (368 to 554)	489.2 (398.3 to 594.1)	23 (15 to 33)	25.1 (16.6 to 35.7)	475 (388 to 583)	514.2 (420.7 to 628.7)
	Male	0 (0 to 0)	0.1 (0.1 to 0.1)	0 (0 to 0)	0.7 (0.6 to 1)	0 (0 to 0)	0.1 (0 to 0.1)	1 (1 to 1)	1.4 (1.1 to 1.8)	0 (0 to 0)	0.1 (0 to 0.1)	1 (1 to 1)	1.5 (1.1 to 1.9)	0 (0 to 0)	0.1 (0.1 to 0.1)	1 (1 to 1)	0.9 (0.7 to 1.1)	0 (0 to 0)	0.1 (0 to 0.1)	1 (1 to 2)	1.5 (1.1 to 2)	0 (0 to 0)	0.1 (0.1 to 0.1)	1 (1 to 2)	1.6 (1.2 to 2.1)
Guatemala	Both	275 (234 to 323)	6.4 (5.5 to 7.5)	2113 (1828 to 2440)	49.2 (42.8 to 56.3)	175 (149 to 202)	4.3 (3.7 to 5)	5996 (5061 to 6946)	131.5 (111.4 to 152.1)	157 (109 to 217)	3.6 (2.5 to 5)	6153 (5179 to 7119)	135.1 (114.8 to 155.9)	1453 (1111 to 1881)	12.2 (9.5 to 15.8)	11434 (8983 to 14561)	95.3 (75.4 to 121)	669 (527 to 848)	6 (4.7 to 7.5)	19986 (15414 to 25695)	163.1 (126.9 to 209.3)	838 (551 to 1211)	7 (4.6 to 10.1)	20825 (16036 to 26673)	170.1 (132 to 218)

		1990												2019											
		Incidence		Prevalence		Deaths		YLLs (Years of Life Lost)		YLDs (Years Lived with Disability)		DALYs (Disability-Adjusted Life Years)		Incidence		Prevalence		Deaths		YLLs (Years of Life Lost)		YLDs (Years Lived with Disability)		DALYs (Disability-Adjusted Life Years)	
Location	Sex	Number	Rate	Number	Rate	Number	Rate	Number	Rate	Number	Rate	Number	Rate	Number	Rate	Number	Rate	Number	Rate	Number	Rate	Number	Rate	Number	Rate
	Female	272 (231 to 319)	12.6 (10.8 to 14.7)	2091 (1805 to 2415)	96.7 (84.1 to 110.5)	172 (146 to 199)	8.5 (7.4 to 9.7)	5915 (4981 to 6870)	256.4 (217.2 to 296.9)	155 (107 to 215)	7.1 (4.9 to 9.8)	6070 (5100 to 7041)	263.5 (223.3 to 304.2)	1446 (1106 to 1873)	22.1 (17 to 28.5)	11378 (8935 to 14486)	172.3 (136.2 to 218.8)	665 (524 to 842)	10.6 (8.4 to 13.4)	19861 (15309 to 25538)	294 (228.4 to 377.3)	834 (547 to 1205)	12.6 (8.3 to 18.2)	20694 (15931 to 26521)	306.6 (237.8 to 392.9)
	Male	3 (3 to 4)	0.2 (0.2 to 0.2)	23 (19 to 27)	1.1 (0.9 to 1.3)	3 (2 to 3)	0.2 (0.1 to 0.2)	81 (67 to 97)	4 (3.2 to 4.8)	2 (1 to 3)	0.1 (0.1 to 0.1)	83 (68 to 100)	4.1 (3.3 to 4.9)	7 (5 to 9)	0.1 (0.1 to 0.2)	56 (44 to 71)	1 (0.8 to 1.3)	5 (4 to 6)	0.1 (0.1 to 0.1)	126 (96 to 162)	2.3 (1.7 to 2.9)	5 (3 to 7)	0.1 (0.1 to 0.1)	130 (100 to 169)	2.3 (1.8 to 3)
Guinea	Both	332 (268 to 404)	9.4 (7.6 to 11.5)	2128 (1779 to 2484)	60 (50.3 to 70)	272 (221 to 330)	8 (6.5 to 9.7)	8472 (6894 to 10205)	230.8 (187.3 to 279.5)	182 (122 to 257)	5.1 (3.4 to 7.2)	8654 (7044 to 10437)	235.9 (191.3 to 286.9)	780 (562 to 1054)	12.7 (9.3 to 16.8)	5175 (3951 to 6755)	82.6 (63.9 to 106.3)	575 (419 to 760)	9.9 (7.3 to 13)	18164 (13089 to 24692)	277.9 (201 to 373.2)	439 (279 to 659)	7 (4.4 to 10.4)	18603 (13383 to 25427)	284.9 (206.6 to 385.2)
	Female	326 (261 to 396)	18.3 (14.7 to 22.3)	2093 (1748 to 2446)	117 (97.8 to 136.4)	266 (215 to 322)	15.5 (12.5 to 18.9)	8313 (6734 to 10001)	446.3 (360.4 to 539.9)	178 (118 to 251)	9.9 (6.6 to 13.9)	8491 (6874 to 10243)	456.2 (368.2 to 552.8)	765 (551 to 1038)	24.6 (17.9 to 32.9)	5095 (3878 to 6680)	161.8 (125.3 to 207.6)	562 (409 to 745)	19.2 (14 to 25.2)	17822 (12784 to 24311)	538.4 (385.6 to 721.4)	430 (272 to 648)	13.6 (8.6 to 20.2)	18252 (13073 to 25017)	552 (395.9 to 746.2)
	Male	7 (5 to 10)	0.4 (0.3 to 0.6)	35 (26 to 49)	2 (1.4 to 2.8)	6 (4 to 9)	0.4 (0.3 to 0.6)	159 (108 to 231)	9 (6.3 to 13.2)	4 (2 to 6)	0.2 (0.1 to 0.4)	163 (111 to 236)	9.3 (6.4 to 13.5)	15 (10 to 23)	0.5 (0.4 to 0.8)	81 (56 to 120)	2.7 (1.8 to 3.9)	13 (9 to 19)	0.5 (0.3 to 0.7)	342 (229 to 519)	11.2 (7.5 to 17.2)	9 (5 to 15)	0.3 (0.2 to 0.5)	351 (235 to 531)	11.5 (7.6 to 17.7)
Guinea-Bissau	Both	52 (36 to 71)	11 (7.7 to 14.8)	330 (240 to 434)	69.5 (52.2 to 89.8)	41 (28 to 55)	9.1 (6.5 to 12)	1410 (958 to 1956)	276.8 (189.8 to 379)	29 (17 to 42)	6 (3.7 to 8.6)	1439 (975 to 1992)	282.7 (194.8 to 385.5)	144 (97 to 211)	16.1 (11.2 to 23.3)	954 (674 to 1355)	104 (76.5 to 143.1)	100 (69 to 145)	12.4 (8.8 to 17.9)	3452 (2321 to 5153)	351.9 (241.9 to 514.2)	81 (49 to 130)	8.8 (5.5 to 14)	3533 (2368 to 5289)	360.7 (247.9 to 524.3)
	Female	52 (36 to 71)	20.7 (14.5 to 27.7)	328 (239 to 432)	130.7 (98.5 to 168.6)	40 (28 to 54)	17.1 (12.1 to 22.5)	1399 (952 to 1944)	518.1 (356.4 to 707.2)	29 (17 to 42)	11.2 (6.9 to 16.1)	1428 (969 to 1977)	529.4 (364.1 to 722.5)	143 (97 to 210)	29.5 (20.4 to 42.7)	950 (671 to 1350)	191.2 (140.2 to 264.2)	99 (69 to 144)	22.3 (15.6 to 32.1)	3433 (2308 to 5134)	649.5 (445.1 to 952.5)	81 (49 to 130)	16.2 (10 to 25.7)	3514 (2354 to 5270)	665.7 (455.3 to 969.3)
	Male	0 (0 to 1)	0.2 (0.1 to 0.3)	2 (1 to 3)	1 (0.6 to 1.5)	0 (0 to 1)	0.2 (0.1 to 0.3)	11 (6 to 18)	4.9 (2.7 to 7.9)	0 (0 to 0)	0.1 (0.1 to 0.2)	11 (6 to 18)	5 (2.8 to 8.1)	1 (1 to 1)	0.2 (0.1 to 0.3)	4 (3 to 6)	1.1 (0.7 to 1.6)	1 (0 to 1)	0.2 (0.1 to 0.3)	19 (12 to 27)	4.7 (3.1 to 6.9)	0 (0 to 1)	0.1 (0.1 to 0.2)	19 (13 to 28)	4.8 (3.2 to 7.1)
Guyana	Both	85 (72 to 101)	19.8 (16.7 to 23.3)	650 (552 to 760)	149.8 (129.1 to 173.3)	50 (42 to 59)	12.5 (10.6 to 14.5)	1591 (1325 to 1894)	348.3 (293.1 to 412.8)	48 (33 to 67)	11.1 (7.6 to 15.3)	1640 (1367 to 1950)	359.4 (302.4 to 426)	181 (135 to 235)	26.1 (19.8 to 33.6)	1421 (1092 to 1812)	202.5 (157.7 to 255.1)	88 (67 to 113)	13.6 (10.6 to 17.2)	2774 (2049 to 3637)	388.2 (290.4 to 505.3)	105 (67 to 151)	15 (9.8 to 21.5)	2878 (2141 to 3761)	403.2 (303.3 to 525.6)
	Female	84 (71 to 100)	37.8 (31.8 to 44.7)	644 (547 to 753)	287.9 (247.6 to 334.2)	49 (41 to 58)	23.5 (20 to 27.4)	1569 (1306 to 1872)	669.2 (562.2 to 796.1)	48 (33 to 66)	21.2 (14.5 to 29.2)	1617 (1347 to 1927)	690.3 (579.8 to 820.8)	180 (134 to 233)	49.4 (37.3 to 63.8)	1411 (1084 to 1798)	384.9 (298.9 to 486.2)	87 (66 to 112)	25.2 (19.5 to 32)	2743 (2028 to 3604)	737.9 (549.9 to 962.6)	104 (67 to 150)	28.4 (18.5 to 40.7)	2847 (2116 to 3727)	766.2 (573.8 to 998.9)
	Male	1 (1 to 1)	0.6 (0.5 to 0.7)	6 (5 to 7)	3 (2.4 to 3.7)	1 (1 to 1)	0.5 (0.4 to 0.6)	22 (18 to 28)	11.2 (9 to 14)	1 (0 to 1)	0.3 (0.2 to 0.5)	23 (19 to 29)	11.5 (9.3 to 14.3)	2 (1 to 2)	0.5 (0.4 to 0.7)	10 (7 to 13)	3.2 (2.4 to 4.3)	1 (1 to 2)	0.4 (0.3 to 0.6)	31 (22 to 42)	9.6 (6.9 to 12.8)	1 (1 to 1)	0.3 (0.2 to 0.5)	32 (23 to 43)	9.9 (7.2 to 13.3)
Haiti	Both	690 (531 to 894)	18.5 (14.6 to 23.3)	4520 (3488 to 5819)	119.3 (95.7 to 148.7)	499 (391 to 634)	14.3 (11 to 17.8)	17145 (12742 to 23131)	429.6 (332 to 555.8)	368 (233 to 539)	9.7 (6.4 to 13.9)	17513 (12997 to 23650)	439.3 (339.8 to 568.9)	1962 (1246 to 2838)	23.3 (15 to 33.2)	13665 (9069 to 19407)	158.1 (108 to 218.7)	1214 (777 to 1746)	15.5 (10.2 to 21.8)	41645 (26388 to 62198)	462 (296.1 to 672.6)	1064 (620 to 1699)	12.4 (7.4 to 19.2)	42709 (27127 to 63472)	474.4 (303.4 to 690.2)
	Female	682 (520 to 887)	35.3 (27.6 to 44.6)	4479 (3456 to 5762)	229.4 (182.4 to 288.2)	492 (386 to 628)	26.8 (20.7 to 33.6)	16942 (12579 to 22789)	827.2 (637.7 to 1071.9)	364 (230 to 532)	18.6 (12.2 to 26.6)	17306 (12821 to 23301)	845.8 (651.3 to 1098.2)	1944 (1233 to 2815)	43.4 (28 to 62.1)	13568 (8981 to 19231)	296 (201.8 to 408.7)	1199 (769 to 1725)	28.8 (18.8 to 40.7)	41240 (26066 to 61772)	861.5 (552.1 to 1259.7)	1054 (612 to 1687)	23.1 (13.8 to 35.9)	42294 (26792 to 63036)	884.6 (564.1 to 1292)
	Male	8 (5 to 16)	0.5 (0.3 to 1)	41 (25 to 75)	2.5 (1.6 to 4.5)	7 (4 to 14)	0.5 (0.3 to 1)	203 (117 to 395)	12 (6.9 to 23.7)	4 (2 to 9)	0.3 (0.2 to 0.6)	207 (119 to 404)	12.3 (7.1 to 24.1)	18 (10 to 32)	0.6 (0.3 to 1)	97 (57 to 169)	2.8 (1.7 to 4.8)	15 (8 to 27)	0.5 (0.3 to 0.9)	405 (222 to 717)	11.4 (6.3 to 20.2)	10 (5 to 20)	0.3 (0.2 to 0.6)	415 (226 to 734)	11.8 (6.4 to 20.8)
Honduras	Both	179 (144 to 219)	7.5 (6 to 9.1)	1383 (1145 to 1647)	58.1 (48.3 to 68.6)	107 (86 to 131)	4.7 (3.8 to 5.7)	3647 (2953 to 4471)	144.5 (116.7 to 177.3)	103 (67 to 143)	4.3 (2.8 to 5.9)	3750 (3034 to 4591)	148.8 (120 to 182.3)	957 (670 to 1326)	14.5 (10.4 to 20)	7337 (5224 to 10109)	109.2 (79.4 to 149.1)	442 (324 to 600)	7.2 (5.4 to 9.5)	13230 (9282 to 18446)	193.9 (139.2 to 267.9)	551 (346 to 847)	8.3 (5.2 to 12.7)	13781 (9676 to 19199)	202.2 (145.3 to 279.2)
	Female	178 (144 to 219)	14.6 (11.7 to 17.8)	1378 (1140 to 1642)	113.6 (94.6 to 134.2)	106 (86 to 130)	9.2 (7.3 to 11.1)	3629 (2934 to 4455)	282.3 (227.9 to 346.4)	102 (67 to 142)	8.3 (5.5 to 11.5)	3731 (3017 to 4573)	290.6 (234.4 to 356.1)	953 (666 to 1323)	27.3 (19.6 to 37.8)	7315 (5208 to 10091)	205.6 (149.2 to 281.3)	440 (321 to 597)	13.5 (10.1 to 17.9)	13168 (9228 to 18392)	364.5 (261.3 to 503.6)	549 (343 to 845)	15.5 (9.8 to 23.9)	13716 (9621 to 19143)	380 (272.3 to 525.8)
	Male	1 (1 to 1)	0.1 (0 to 0.1)	5 (4 to 6)	0.4 (0.3 to 0.5)	1 (0 to 1)	0.1 (0 to 0.1)	19 (14 to 24)	1.5 (1.1 to 2.1)	0 (0 to 1)	0 (0 to 0.1)	19 (14 to 25)	1.6 (1.1 to 2.1)	4 (2 to 6)	0.1 (0.1 to 0.2)	22 (13 to 32)	0.7 (0.4 to 1.1)	3 (1 to 4)	0.1 (0.1 to 0.2)	62 (37 to 96)	2.1 (1.2 to 3.2)	2 (1 to 3)	0.1 (0 to 0.1)	64 (39 to 99)	2.1 (1.2 to 3.3)

		1990												2019											
		Incidence		Prevalence		Deaths		YLLs (Years of Life Lost)		YLDs (Years Lived with Disability)		DALYs (Disability-Adjusted Life Years)		Incidence		Prevalence		Deaths		YLLs (Years of Life Lost)		YLDs (Years Lived with Disability)		DALYs (Disability-Adjusted Life Years)	
Location	Sex	Number	Rate	Number	Rate	Number	Rate	Number	Rate	Number	Rate	Number	Rate	Number	Rate	Number	Rate	Number	Rate	Number	Rate	Number	Rate	Number	Rate
Hungary	Both	4642 (4378 to 4900)	33.7 (31.7 to 35.6)	42989 (39473 to 46999)	308.4 (284 to 335.2)	2181 (2090 to 2267)	15.7 (15.1 to 16.3)	56204 (54029 to 58264)	410.5 (394.7 to 425.4)	3109 (2163 to 4184)	22.4 (15.6 to 30.2)	59313 (56859 to 61751)	433 (414.8 to 450.1)	5974 (4874 to 7315)	35.4 (28.4 to 43.5)	59493 (50633 to 70224)	346.9 (292.2 to 412.9)	2207 (1813 to 2651)	11.8 (9.6 to 14.2)	47584 (38466 to 57601)	282.8 (227 to 344.3)	4147 (2820 to 5828)	24.5 (16.7 to 34.7)	51732 (42000 to 62880)	307.3 (248.8 to 376.4)
	Female	4592 (4319 to 4847)	60 (56.4 to 63.4)	42602 (39105 to 46617)	548.6 (505.6 to 598)	2156 (2065 to 2243)	26.8 (25.7 to 27.8)	55614 (53476 to 57655)	737.3 (708.7 to 764.6)	3071 (2136 to 4130)	40 (27.7 to 54)	58685 (56210 to 61094)	777.4 (744.3 to 808.6)	5919 (4830 to 7251)	64 (51.1 to 79.1)	59017 (50169 to 69618)	624.9 (523.7 to 747.3)	2187 (1795 to 2622)	20.1 (16.3 to 24.2)	47144 (38100 to 57044)	511.4 (407.4 to 624.3)	4104 (2793 to 5773)	44.4 (30.2 to 63.1)	51248 (41612 to 62286)	555.8 (447.8 to 683.2)
	Male	50 (42 to 60)	0.8 (0.7 to 1)	386 (323 to 462)	6.4 (5.4 to 7.6)	25 (21 to 28)	0.4 (0.4 to 0.5)	590 (517 to 678)	9.7 (8.5 to 11)	38 (25 to 54)	0.6 (0.4 to 0.9)	628 (546 to 720)	10.3 (9 to 11.7)	55 (40 to 74)	0.7 (0.5 to 1)	476 (366 to 626)	6.3 (4.9 to 8.1)	20 (16 to 26)	0.3 (0.2 to 0.3)	441 (337 to 566)	5.9 (4.5 to 7.4)	43 (28 to 65)	0.6 (0.4 to 0.9)	484 (370 to 617)	6.4 (5 to 8.1)
Iceland	Both	126 (114 to 139)	47.1 (42.6 to 52)	1347 (1202 to 1499)	498.2 (446.8 to 552.6)	39 (36 to 42)	14.1 (13 to 15.3)	965 (890 to 1043)	365.5 (336.5 to 395)	96 (66 to 131)	35.7 (24.6 to 49.2)	1061 (976 to 1151)	401.2 (369 to 435)	173 (145 to 203)	35.2 (29.7 to 41.1)	2054 (1820 to 2312)	406.7 (359.5 to 460.6)	43 (36 to 49)	7.7 (6.7 to 8.7)	917 (801 to 1029)	188.7 (166 to 212.1)	138 (95 to 194)	27.8 (18.9 to 39)	1055 (921 to 1186)	216.4 (189.1 to 242.7)
	Female	121 (109 to 134)	88 (79.4 to 97.3)	1309 (1170 to 1459)	932.7 (835.2 to 1036.5)	37 (34 to 40)	25.4 (23.3 to 27.6)	926 (853 to 1004)	685.2 (630.9 to 743)	92 (63 to 127)	66.6 (45.7 to 92.4)	1019 (934 to 1105)	751.8 (690.3 to 815.4)	168 (140 to 196)	67.5 (56.9 to 78.9)	1997 (1766 to 2251)	779.6 (685.9 to 884.8)	41 (35 to 47)	14.2 (12.2 to 16.1)	884 (772 to 993)	361.3 (317.2 to 406.6)	133 (91 to 187)	53.1 (36 to 74.4)	1017 (886 to 1145)	414.4 (361.9 to 467.6)
	Male	5 (4 to 6)	3.6 (2.9 to 4.4)	38 (31 to 46)	29.2 (24.1 to 35.2)	2 (2 to 2)	1.4 (1.2 to 1.6)	39 (33 to 44)	30.1 (26 to 34.3)	4 (2 to 5)	2.7 (1.8 to 3.9)	42 (36 to 49)	32.8 (28.2 to 37.7)	6 (4 to 7)	2.3 (1.7 to 2.9)	57 (46 to 70)	22.3 (18 to 27.1)	2 (1 to 2)	0.6 (0.5 to 0.8)	33 (26 to 39)	13 (10.6 to 15.5)	5 (3 to 7)	1.9 (1.2 to 2.7)	37 (31 to 45)	14.9 (12.2 to 17.8)
India	Both	37212 (30838 to 43917)	7 (5.8 to 8.3)	275530 (239155 to 315685)	52.2 (45.3 to 59.8)	26924 (22338 to 31252)	5.6 (4.6 to 6.5)	917493 (767363 to 1064024)	159.5 (132.6 to 185.3)	21701 (14945 to 29194)	4 (2.8 to 5.4)	939194 (785949 to 1085775)	163.5 (136.2 to 189.5)	146090 (112452 to 183482)	11.8 (9.1 to 14.8)	1121651 (888873 to 1382804)	89.8 (71.6 to 109.9)	83510 (64550 to 105994)	7.1 (5.5 to 9)	2609545 (2002942 to 3335792)	204.9 (157.6 to 261.7)	87805 (57874 to 126407)	7 (4.6 to 10)	2697350 (2090173 to 3424541)	212 (164.2 to 268.6)
	Female	35840 (29545 to 42492)	13.9 (11.3 to 16.5)	267361 (230585 to 307320)	104.9 (90.8 to 120.8)	25779 (21135 to 30096)	10.8 (8.8 to 12.7)	886376 (734054 to 1033786)	319.7 (262.4 to 372.8)	20885 (14334 to 28207)	8 (5.5 to 10.7)	907261 (754255 to 1055763)	327.6 (270.1 to 380.6)	144086 (110434 to 181150)	23 (17.8 to 29)	1106547 (874593 to 1364804)	175.7 (139.1 to 215.7)	82099 (63114 to 104727)	13.7 (10.6 to 17.3)	2572826 (1963543 to 3297949)	402.7 (308 to 515.9)	86417 (56726 to 124669)	13.7 (9 to 19.7)	2659243 (2049879 to 3389998)	416.4 (321 to 530.3)
	Male	1371 (1089 to 1682)	0.7 (0.6 to 0.9)	8169 (6709 to 9762)	3.5 (2.9 to 4.2)	1146 (922 to 1393)	0.7 (0.5 to 0.8)	31117 (24915 to 38031)	13.2 (10.7 to 16.1)	816 (526 to 1135)	0.4 (0.2 to 0.5)	31933 (25582 to 38924)	13.6 (11 to 16.5)	2004 (1542 to 2530)	0.4 (0.3 to 0.5)	15104 (12100 to 18408)	2.7 (2.2 to 3.3)	1411 (1083 to 1745)	0.3 (0.2 to 0.4)	36719 (28022 to 45830)	6.3 (4.8 to 7.8)	1388 (932 to 1942)	0.3 (0.2 to 0.3)	38107 (29317 to 47166)	6.5 (5.1 to 8.1)
Indonesia	Both	17057 (14515 to 20365)	13.1 (11.2 to 15.4)	134190 (116094 to 156424)	106.4 (93.2 to 123.1)	11019 (9467 to 13091)	9.1 (7.8 to 10.7)	423161 (359596 to 507409)	310 (265.6 to 368.9)	10508 (7068 to 14575)	8 (5.5 to 11)	433669 (369069 to 519270)	318 (273.5 to 378)	50985 (39200 to 66927)	19.1 (14.9 to 24.7)	432756 (349659 to 542892)	162.7 (133.3 to 202.2)	26420 (20392 to 33925)	10.7 (8.4 to 13.5)	948050 (728692 to 1235076)	345.8 (266.9 to 446.7)	32809 (21858 to 47605)	12.2 (8.2 to 17.5)	980859 (763277 to 1264306)	358 (279.5 to 457.6)
	Female	16967 (14431 to 20287)	25.4 (21.8 to 30)	133550 (115464 to 155872)	206 (180.3 to 238.5)	10944 (9391 to 13019)	17.5 (15 to 20.6)	421050 (356946 to 505580)	605.3 (517.4 to 721.7)	10448 (7028 to 14502)	15.6 (10.6 to 21.3)	431498 (366693 to 517352)	620.8 (533.2 to 739)	50628 (38906 to 66656)	37.4 (29 to 48.6)	430401 (347084 to 541008)	318.9 (259.6 to 397.8)	26166 (20171 to 33676)	20.5 (15.9 to 25.9)	941238 (722445 to 1227283)	680.8 (523.7 to 882.7)	32571 (21708 to 47413)	23.9 (16.1 to 34.6)	973809 (757445 to 1257968)	704.7 (549.2 to 905.8)
	Male	91 (67 to 120)	0.2 (0.1 to 0.3)	640 (498 to 821)	1.3 (1 to 1.6)	75 (56 to 98)	0.2 (0.1 to 0.2)	2111 (1577 to 2784)	4 (3 to 5.3)	60 (38 to 87)	0.1 (0.1 to 0.2)	2171 (1631 to 2854)	4.1 (3.1 to 5.4)	357 (230 to 532)	0.4 (0.2 to 0.5)	2355 (1632 to 3323)	2.2 (1.5 to 3)	254 (166 to 372)	0.3 (0.2 to 0.4)	6812 (4463 to 9924)	6.1 (4.1 to 8.9)	238 (138 to 375)	0.2 (0.1 to 0.4)	7050 (4639 to 10183)	6.4 (4.2 to 9.2)
Iran (Islamic Republic of)	Both	2871 (2389 to 3608)	9.3 (7.5 to 11.8)	26458 (22494 to 31775)	87.9 (73.7 to 106)	1436 (1186 to 1823)	5.1 (4.1 to 6.7)	48718 (41357 to 60770)	150.8 (125.9 to 188.8)	1929 (1304 to 2771)	6.2 (4.2 to 9)	50647 (43014 to 62892)	157 (130.7 to 198.1)	14863 (13372 to 16590)	17.1 (15.4 to 19.1)	135257 (122011 to 150481)	156.9 (141.7 to 173.7)	4760 (4362 to 5250)	6 (5.5 to 6.6)	153082 (140075 to 168502)	174.1 (159.6 to 191.6)	10009 (6956 to 13776)	11.5 (8 to 15.8)	163091 (148806 to 179221)	185.6 (169.7 to 203.4)

		1990												2019											
		Incidence		Prevalence		Deaths		YLLs (Years of Life Lost)		YLDs (Years Lived with Disability)		DALYs (Disability-Adjusted Life Years)		Incidence		Prevalence		Deaths		YLLs (Years of Life Lost)		YLDs (Years Lived with Disability)		DALYs (Disability-Adjusted Life Years)	
Location	Sex	Number	Rate	Number	Rate	Number	Rate	Number	Rate	Number	Rate	Number	Rate	Number	Rate	Number	Rate	Number	Rate	Number	Rate	Number	Rate	Number	Rate
	Female	2835 (2352 to 3575)	18.8 (15.3 to 24.1)	26160 (22174 to 31486)	178.6 (149.7 to 216.4)	1413 (1160 to 1796)	10.3 (8.2 to 13.6)	48016 (40680 to 59999)	307.6 (255.7 to 386.4)	1903 (1285 to 2739)	12.6 (8.5 to 18.4)	49919 (42208 to 62170)	320.2 (265.4 to 405.4)	14743 (13248 to 16469)	34 (30.7 to 37.9)	134187 (120955 to 149206)	312.1 (281.5 to 345.4)	4704 (4306 to 5192)	11.9 (10.8 to 13.1)	151570 (138503 to 166910)	345.9 (316.9 to 380.9)	9916 (6887 to 13657)	22.8 (15.9 to 31.4)	161486 (147227 to 177500)	368.7 (336.7 to 404.3)
	Male	36 (27 to 48)	0.2 (0.2 to 0.3)	298 (233 to 383)	2 (1.6 to 2.6)	23 (18 to 30)	0.2 (0.1 to 0.2)	702 (550 to 895)	4.3 (3.4 to 5.6)	26 (17 to 38)	0.2 (0.1 to 0.3)	728 (574 to 926)	4.5 (3.5 to 5.8)	120 (98 to 143)	0.3 (0.3 to 0.4)	1070 (886 to 1280)	2.8 (2.3 to 3.3)	56 (48 to 63)	0.2 (0.1 to 0.2)	1512 (1296 to 1689)	3.8 (3.2 to 4.2)	93 (62 to 133)	0.2 (0.2 to 0.3)	1605 (1379 to 1790)	4 (3.5 to 4.5)
Iraq	Both	1351 (985 to 1837)	14.6 (10.6 to 20)	10976 (8508 to 14153)	120.7 (94.4 to 154.6)	772 (560 to 1057)	8.8 (6.4 to 12.1)	26921 (19629 to 36630)	283.2 (204.8 to 386.9)	842 (525 to 1269)	9.1 (5.5 to 13.6)	27763 (20292 to 37907)	292.3 (212.2 to 399.4)	7902 (5795 to 10587)	26.2 (19.7 to 34.7)	67929 (51815 to 88894)	227.2 (177.5 to 292.9)	3015 (2252 to 3985)	11.1 (8.5 to 14.4)	105319 (76701 to 142639)	342.7 (254.4 to 457.9)	5093 (3251 to 7684)	16.8 (10.9 to 24.6)	110411 (80899 to 149282)	359.5 (267.4 to 478.9)
	Female	1337 (970 to 1824)	29 (21 to 40)	10887 (8421 to 14073)	239.4 (186 to 307.6)	762 (548 to 1046)	17.3 (12.5 to 23.8)	26651 (19302 to 36443)	565.4 (406.8 to 773.4)	833 (520 to 1258)	18 (11 to 27.2)	27485 (19934 to 37671)	583.4 (421.5 to 798.3)	7819 (5733 to 10484)	52 (38.9 to 68.9)	67317 (51236 to 88245)	451.6 (351.6 to 583.5)	2970 (2217 to 3930)	21.6 (16.4 to 28.1)	103998 (75526 to 140725)	681.6 (505 to 909.9)	5035 (3203 to 7606)	33.3 (21.6 to 49)	109032 (79889 to 147823)	714.9 (529.9 to 957.2)
	Male	13 (9 to 19)	0.3 (0.2 to 0.5)	89 (63 to 122)	2.2 (1.6 to 3)	10 (7 to 15)	0.3 (0.2 to 0.4)	270 (179 to 388)	6.5 (4.2 to 9.2)	9 (5 to 14)	0.2 (0.1 to 0.3)	279 (185 to 400)	6.7 (4.4 to 9.5)	83 (54 to 119)	0.7 (0.4 to 0.9)	612 (402 to 864)	4.8 (3.3 to 6.6)	45 (31 to 62)	0.4 (0.3 to 0.5)	1321 (855 to 1900)	9.9 (6.7 to 13.6)	58 (34 to 92)	0.5 (0.3 to 0.7)	1379 (892 to 1980)	10.4 (7 to 14.2)
Ireland	Both	1842 (1700 to 1970)	49 (45.1 to 52.4)	19034 (17299 to 20939)	499.1 (456.5 to 547.1)	682 (648 to 714)	17.9 (16.9 to 18.7)	17474 (16719 to 18224)	470.1 (448.9 to 490.2)	1366 (970 to 1850)	36 (25.5 to 48.8)	18841 (17963 to 19796)	506.2 (482 to 531.3)	3408 (2616 to 4345)	49.2 (37.4 to 62.8)	37096 (30531 to 44804)	529.2 (429.9 to 643.5)	804 (724 to 886)	11 (9.9 to 12)	18000 (16521 to 19641)	260.6 (238.9 to 284.6)	2596 (1708 to 3792)	37.4 (24.5 to 54.7)	20595 (18570 to 22919)	298 (269.1 to 332.5)
	Female	1833 (1692 to 1961)	93.7 (86.3 to 100.6)	18949 (17223 to 20854)	948.6 (867.5 to 1036.5)	678 (644 to 710)	32.8 (31.2 to 34.3)	17382 (16627 to 18130)	906.4 (865.5 to 946.5)	1358 (963 to 1840)	68.8 (48.5 to 93.4)	18740 (17869 to 19698)	975.2 (928.8 to 1024.9)	3386 (2599 to 4323)	95 (72 to 121.5)	36885 (30382 to 44537)	1020.2 (826.8 to 1243.4)	798 (718 to 879)	20.5 (18.6 to 22.4)	17873 (16418 to 19520)	502.3 (460.3 to 548.3)	2577 (1690 to 3761)	72 (47.1 to 105.9)	20450 (18431 to 22766)	574.4 (517.6 to 641.5)
	Male	9 (7 to 11)	0.5 (0.4 to 0.6)	85 (69 to 103)	4.7 (3.9 to 5.6)	4 (3 to 5)	0.2 (0.2 to 0.3)	93 (80 to 108)	5.1 (4.4 to 5.9)	8 (5 to 11)	0.4 (0.3 to 0.6)	101 (88 to 118)	5.5 (4.8 to 6.4)	22 (15 to 31)	0.6 (0.4 to 0.9)	211 (155 to 288)	6.1 (4.6 to 8.3)	6 (5 to 8)	0.2 (0.1 to 0.2)	127 (100 to 155)	3.8 (3 to 4.6)	19 (11 to 29)	0.5 (0.3 to 0.8)	145 (114 to 180)	4.3 (3.4 to 5.3)
Israel	Both	1893 (1760 to 2022)	41.1 (38.2 to 43.8)	19904 (17992 to 22073)	426.9 (386.8 to 471.6)	797 (756 to 837)	17.4 (16.4 to 18.2)	21013 (20158 to 21919)	459.8 (441.3 to 479.5)	1406 (974 to 1924)	30.3 (21 to 41.6)	22419 (21423 to 23545)	490 (467.9 to 513.7)	4464 (3441 to 5702)	41.8 (32 to 53.4)	48155 (39704 to 57606)	447.4 (366.6 to 537.7)	1361 (1231 to 1476)	11.8 (10.7 to 12.7)	29489 (27287 to 31666)	278.5 (258.4 to 298.7)	3365 (2197 to 4829)	31.5 (20.5 to 45.6)	32853 (30058 to 35655)	310 (284.5 to 337)
	Female	1878 (1746 to 2008)	76.9 (71.4 to 82.2)	19770 (17866 to 21930)	796.2 (721.5 to 878.4)	789 (749 to 829)	32.1 (30.4 to 33.7)	20842 (19992 to 21742)	862.6 (827.7 to 900)	1394 (966 to 1909)	56.5 (39.2 to 77.9)	22236 (21239 to 23354)	919.1 (877.7 to 963.8)	4410 (3398 to 5635)	78.8 (60.2 to 100.9)	47673 (39271 to 57043)	842.9 (689.8 to 1017.1)	1341 (1213 to 1457)	21.5 (19.6 to 23.1)	29102 (26916 to 31204)	525.2 (486.9 to 563.5)	3321 (2167 to 4768)	59.4 (38.5 to 86.1)	32423 (29665 to 35176)	584.6 (536.8 to 635.2)
	Male	15 (12 to 18)	0.7 (0.6 to 0.8)	134 (112 to 161)	6.1 (5.1 to 7.2)	8 (7 to 10)	0.4 (0.3 to 0.4)	171 (148 to 198)	7.9 (6.8 to 9)	12 (8 to 17)	0.5 (0.4 to 0.8)	183 (157 to 212)	8.4 (7.2 to 9.7)	54 (38 to 74)	1 (0.7 to 1.4)	481 (359 to 635)	9.3 (7 to 12.3)	20 (16 to 24)	0.4 (0.3 to 0.5)	386 (315 to 466)	7.6 (6.2 to 9.1)	44 (27 to 68)	0.8 (0.5 to 1.3)	430 (351 to 519)	8.4 (6.9 to 10.2)
Italy	Both	37884 (35702 to 40229)	47.9 (45.1 to 50.8)	422764 (384546 to 465244)	520.7 (477 to 568.5)	12299 (11797 to 12594)	14.8 (14.1 to 15.1)	303888 (295148 to 309993)	386.8 (376.3 to 394.7)	28958 (19976 to 40025)	36.2 (25 to 50.2)	332846 (320422 to 346045)	423.1 (407.5 to 439.6)	53565 (41369 to 68124)	47.1 (36.4 to 60.5)	625993 (520330 to 746939)	530 (438.1 to 643)	14450 (12591 to 15499)	10.2 (9.2 to 10.9)	278990 (256976 to 295069)	244.5 (230.7 to 257.7)	41936 (27292 to 61004)	36.6 (23.3 to 53.8)	320926 (294648 to 346641)	281.1 (260.6 to 302.8)
	Female	37782 (35596 to 40124)	88.9 (83.7 to 94.4)	421458 (383261 to 463738)	954 (875.9 to 1039.7)	12260 (11759 to 12555)	26.2 (25.2 to 26.8)	302956 (294221 to 309071)	721.3 (702.9 to 735.6)	28859 (19911 to 39884)	67 (46.2 to 93.1)	331815 (319382 to 345002)	788.3 (759.9 to 818.8)	53095 (40906 to 67752)	89.2 (68.5 to 114.9)	621334 (515663 to 742482)	998.5 (821.6 to 1217.2)	14309 (12454 to 15346)	18.5 (16.8 to 19.6)	276333 (254466 to 292367)	461.6 (436.5 to 486.3)	41533 (26983 to 60577)	69.1 (44 to 101.9)	317865 (291748 to 343491)	530.8 (492.6 to 571.8)
	Male	103 (88 to 119)	0.3 (0.2 to 0.3)	1306 (1110 to 1547)	3.6 (3.1 to 4.3)	39 (37 to 41)	0.1 (0.1 to 0.1)	931 (891 to 971)	2.5 (2.4 to 2.7)	100 (66 to 144)	0.3 (0.2 to 0.4)	1031 (977 to 1094)	2.8 (2.7 to 3)	470 (346 to 620)	0.8 (0.6 to 1.1)	4659 (3700 to 5872)	8 (6.3 to 10.1)	142 (127 to 156)	0.2 (0.2 to 0.2)	2657 (2430 to 2932)	4.8 (4.4 to 5.3)	403 (257 to 602)	0.7 (0.4 to 1)	3061 (2754 to 3401)	5.5 (5 to 6.1)
Jamaica	Both	395 (365 to 427)	23.3 (21.6 to 25.2)	3287 (3024 to 3583)	193.9 (178 to 211)	190 (178 to 203)	11 (10.3 to 11.8)	5070 (4736 to 5447)	301.5 (281 to 323.9)	236 (168 to 315)	13.9 (9.9 to 18.5)	5307 (4950 to 5706)	315.4 (294 to 340.1)	1224 (942 to 1553)	40.9 (31.5 to 52.1)	10107 (7933 to 12641)	339.7 (267.2 to 425.3)	472 (372 to 582)	15.5 (12.2 to 19.1)	13334 (10334 to 16933)	446.1 (343.9 to 566.8)	729 (478 to 1047)	24.5 (16.1 to 35.3)	14062 (10953 to 17865)	470.5 (366 to 597.6)

		1990												2019											
		Incidence		Prevalence		Deaths		YLLs (Years of Life Lost)		YLDs (Years Lived with Disability)		DALYs (Disability-Adjusted Life Years)		Incidence		Prevalence		Deaths		YLLs (Years of Life Lost)		YLDs (Years Lived with Disability)		DALYs (Disability-Adjusted Life Years)	
Location	Sex	Number	Rate	Number	Rate	Number	Rate	Number	Rate	Number	Rate	Number	Rate	Number	Rate	Number	Rate	Number	Rate	Number	Rate	Number	Rate	Number	Rate
	Female	391 (361 to 423)	44.1 (40.7 to 47.8)	3261 (2998 to 3553)	368.2 (337.7 to 400.5)	187 (175 to 200)	20.4 (19.1 to 21.8)	5008 (4680 to 5379)	573 (533.5 to 616.2)	234 (167 to 312)	26.3 (18.7 to 35.1)	5242 (4888 to 5642)	599.4 (557.4 to 647.3)	1212 (934 to 1538)	79.3 (60.9 to 100.9)	10028 (7864 to 12546)	661 (517.8 to 827.5)	464 (367 to 571)	29.3 (23.1 to 36.4)	13165 (10194 to 16735)	863.8 (665.1 to 1099.3)	721 (472 to 1037)	47.4 (31 to 68.6)	13887 (10810 to 17654)	911.2 (706.6 to 1159.3)
	Male	4 (3 to 5)	0.5 (0.4 to 0.6)	27 (22 to 32)	3.2 (2.7 to 3.8)	3 (2 to 4)	0.4 (0.3 to 0.4)	62 (52 to 74)	7.6 (6.4 to 8.9)	3 (2 to 3)	0.3 (0.2 to 0.4)	65 (55 to 77)	7.9 (6.7 to 9.3)	12 (8 to 16)	0.8 (0.6 to 1.2)	79 (57 to 109)	5.5 (4 to 7.6)	8 (5 to 10)	0.5 (0.4 to 0.8)	168 (119 to 233)	11.7 (8.3 to 16.1)	8 (5 to 12)	0.5 (0.3 to 0.8)	176 (125 to 243)	12.2 (8.7 to 16.8)
Japan	Both	31618 (29714 to 33638)	19.1 (17.9 to 20.3)	392788 (353059 to 441357)	234.8 (211.1 to 262.9)	7949 (7602 to 8154)	4.8 (4.6 to 5)	235189 (228603 to 240092)	142 (138 to 145)	26008 (17742 to 35857)	15.6 (10.7 to 21.5)	261197 (250804 to 272729)	157.7 (151.4 to 164.5)	74597 (59578 to 91312)	31 (24.8 to 38.2)	887472 (754022 to 1045076)	345.6 (288 to 411.2)	16038 (13489 to 17428)	5.4 (4.8 to 5.7)	337888 (307244 to 359272)	148.6 (139.8 to 157)	59886 (39369 to 85805)	24.3 (15.9 to 35.4)	397774 (358070 to 434855)	173 (160.5 to 187.6)
	Female	31483 (29570 to 33515)	36.1 (33.9 to 38.4)	391384 (351640 to 439792)	435.9 (393.7 to 485.4)	7889 (7544 to 8093)	8.8 (8.4 to 9)	233797 (227226 to 238706)	270.9 (263.8 to 276.4)	25889 (17663 to 35692)	29.3 (20 to 40.4)	259686 (249314 to 271167)	300.3 (288.7 to 313)	74260 (59292 to 90978)	60.5 (48.3 to 74.6)	884138 (750254 to 1041945)	669.6 (555.6 to 799.4)	15911 (13374 to 17288)	10.1 (9.2 to 10.8)	335705 (305320 to 356935)	290.8 (275.2 to 307.2)	59587 (39178 to 85407)	47.3 (30.8 to 68.9)	395292 (355850 to 432122)	338.1 (314.5 to 366.5)
	Male	135 (119 to 156)	0.2 (0.2 to 0.2)	1404 (1194 to 1654)	2 (1.7 to 2.3)	59 (57 to 61)	0.1 (0.1 to 0.1)	1392 (1349 to 1436)	1.9 (1.8 to 2)	119 (81 to 167)	0.2 (0.1 to 0.2)	1511 (1453 to 1579)	2.1 (2 to 2.2)	337 (262 to 431)	0.2 (0.2 to 0.3)	3334 (2669 to 4107)	2.4 (2 to 3)	127 (113 to 139)	0.1 (0.1 to 0.1)	2183 (1997 to 2360)	1.7 (1.6 to 1.8)	299 (190 to 435)	0.2 (0.1 to 0.3)	2482 (2251 to 2717)	1.9 (1.8 to 2.1)
Jordan	Both	306 (238 to 381)	17.7 (13.7 to 22.1)	2549 (2100 to 3095)	150 (123.3 to 180.4)	163 (127 to 203)	10.5 (8.1 to 13.3)	5664 (4437 to 6955)	312.3 (242.4 to 385.3)	193 (128 to 283)	11.1 (7.4 to 16.4)	5858 (4575 to 7202)	323.4 (251.6 to 399.1)	2075 (1591 to 2658)	24.9 (19.3 to 31.5)	18566 (14743 to 23262)	223.6 (180.6 to 272.1)	685 (536 to 869)	9.5 (7.5 to 11.9)	22093 (17155 to 28430)	258.5 (202.4 to 328.5)	1384 (874 to 2001)	16.5 (10.6 to 23.6)	23477 (18164 to 30109)	275 (214 to 348.5)
	Female	301 (234 to 377)	35.9 (27.6 to 45.2)	2519 (2065 to 3059)	305.2 (250.3 to 369.8)	160 (123 to 200)	21 (15.9 to 26.7)	5582 (4339 to 6887)	637.2 (492.4 to 792.2)	190 (126 to 279)	22.6 (14.9 to 33.4)	5772 (4486 to 7096)	659.8 (507.8 to 815.4)	2053 (1576 to 2636)	52.9 (41 to 67.4)	18387 (14602 to 23089)	474.7 (383.1 to 580)	674 (526 to 858)	19.9 (15.6 to 24.9)	21809 (16849 to 28024)	548.6 (427.6 to 698.7)	1368 (864 to 1982)	35 (22.3 to 50)	23176 (17862 to 29882)	583.6 (452.8 to 744.7)
	Male	4 (3 to 6)	0.7 (0.5 to 1)	30 (22 to 40)	4.3 (3.2 to 5.8)	3 (2 to 5)	0.6 (0.4 to 0.8)	82 (59 to 116)	11.4 (8.1 to 16.1)	3 (2 to 4)	0.4 (0.3 to 0.7)	85 (61 to 120)	11.8 (8.4 to 16.7)	22 (15 to 31)	0.6 (0.4 to 0.9)	180 (128 to 247)	4.9 (3.6 to 6.8)	11 (7 to 15)	0.4 (0.2 to 0.5)	285 (195 to 411)	7.5 (5.1 to 10.5)	16 (10 to 26)	0.5 (0.3 to 0.7)	301 (206 to 433)	7.9 (5.5 to 11.1)
Kazakhstan	Both	2689 (2488 to 2890)	19.8 (18.4 to 21.2)	23868 (21842 to 26148)	177.8 (162.9 to 195.4)	1435 (1340 to 1529)	11 (10.3 to 11.8)	44372 (41326 to 47524)	319.4 (297.9 to 341.9)	1748 (1221 to 2422)	12.9 (9 to 17.8)	46120 (42923 to 49280)	332.3 (310 to 355.4)	4252 (3558 to 5002)	22.7 (19.1 to 26.5)	38603 (32942 to 44656)	206.7 (177.7 to 238)	1686 (1444 to 1956)	9.6 (8.3 to 11.1)	49054 (41397 to 57434)	258.2 (219.3 to 300.8)	2834 (1951 to 3958)	15.1 (10.4 to 20.9)	51889 (43913 to 61260)	273.3 (231.4 to 319.6)
	Female	2684 (2482 to 2885)	34.4 (31.9 to 37)	23823 (21802 to 26101)	305.8 (281.4 to 334.8)	1432 (1337 to 1526)	18.5 (17.3 to 19.7)	44283 (41234 to 47427)	565.3 (527 to 605.4)	1744 (1218 to 2416)	22.3 (15.6 to 31.2)	46028 (42826 to 49187)	587.6 (547.5 to 629)	4236 (3545 to 4985)	40.1 (33.6 to 47.1)	38481 (32838 to 44550)	364.2 (311.6 to 420.9)	1679 (1437 to 1948)	16.3 (14 to 18.9)	48861 (41237 to 57225)	459.7 (389.6 to 537.6)	2822 (1942 to 3940)	26.7 (18.4 to 36.9)	51683 (43723 to 61053)	486.4 (411.2 to 572.5)
	Male	5 (5 to 6)	0.1 (0.1 to 0.1)	45 (38 to 54)	0.8 (0.7 to 1)	3 (3 to 3)	0.1 (0.1 to 0.1)	89 (79 to 98)	1.6 (1.4 to 1.7)	4 (3 to 6)	0.1 (0.1 to 0.1)	93 (83 to 103)	1.6 (1.5 to 1.8)	16 (13 to 20)	0.2 (0.2 to 0.3)	122 (97 to 153)	1.7 (1.3 to 2.1)	7 (6 to 9)	0.1 (0.1 to 0.1)	194 (158 to 237)	2.6 (2.1 to 3.2)	12 (8 to 18)	0.2 (0.1 to 0.2)	206 (168 to 252)	2.8 (2.3 to 3.4)
Kenya	Both	755 (514 to 1096)	8.3 (5.8 to 11.8)	5205 (3758 to 7252)	55.5 (41.2 to 75.2)	564 (393 to 803)	6.7 (4.8 to 9.4)	17383 (11747 to 25493)	176.6 (121.3 to 253.5)	437 (264 to 698)	4.6 (2.9 to 7.3)	17820 (12059 to 26135)	181.2 (124.4 to 260.7)	3398 (2485 to 4509)	13.3 (10 to 17.3)	24004 (18125 to 31437)	88.5 (68.6 to 113.2)	2321 (1747 to 3132)	10.3 (7.9 to 13.4)	73574 (53850 to 102699)	263.4 (197.5 to 357.5)	2016 (1278 to 3014)	7.5 (4.9 to 11)	75590 (55661 to 104567)	270.9 (203.3 to 365.8)
	Female	668 (441 to 1003)	13.9 (9.3 to 20.5)	4770 (3378 to 6749)	97.9 (71.1 to 134.6)	484 (325 to 711)	10.8 (7.4 to 15.7)	15649 (10251 to 23562)	304.3 (201.7 to 451.7)	390 (230 to 628)	7.9 (4.8 to 12.5)	16039 (10541 to 24116)	312.2 (207.6 to 461.8)	3162 (2250 to 4323)	23 (16.6 to 30.6)	22702 (16737 to 30261)	158.4 (120.2 to 207)	2124 (1547 to 2902)	17 (12.7 to 22.8)	68797 (48989 to 96488)	466.9 (338 to 642.2)	1880 (1179 to 2881)	13.1 (8.4 to 19.7)	70676 (50797 to 98743)	480 (350.6 to 658)
	Male	86 (62 to 138)	2.5 (1.8 to 3.9)	434 (322 to 665)	11.6 (8.7 to 17.4)	80 (58 to 122)	2.5 (1.8 to 3.7)	1735 (1213 to 2736)	45.2 (32.2 to 70.3)	46 (28 to 76)	1.3 (0.8 to 2)	1781 (1246 to 2811)	46.5 (33 to 72.3)	236 (161 to 365)	2.8 (1.9 to 4.2)	1302 (917 to 1972)	13.5 (9.8 to 19.9)	197 (137 to 298)	2.6 (1.8 to 3.9)	4777 (3295 to 7315)	47.7 (33.1 to 72)	136 (80 to 221)	1.5 (0.9 to 2.3)	4913 (3406 to 7568)	49.2 (34.4 to 73.8)
Kiribati	Both	9 (7 to 11)	20.5 (16.1 to 25.1)	66 (54 to 79)	150.3 (125.3 to 177.2)	7 (5 to 8)	16.7 (13.1 to 20.4)	237 (184 to 293)	500.4 (392.2 to 621.2)	5 (4 to 7)	11.7 (7.9 to 16.2)	242 (188 to 300)	512.1 (401.4 to 635.7)	21 (15 to 28)	26 (19 to 35.7)	150 (113 to 195)	180.5 (141.2 to 231.7)	14 (10 to 20)	20.1 (14.9 to 27.4)	488 (344 to 673)	562.6 (404.1 to 775)	12 (8 to 18)	14.5 (9.4 to 21.6)	500 (352 to 686)	577.1 (415 to 790.4)

		1990												2019											
		Incidence		Prevalence		Deaths		YLLs (Years of Life Lost)		YLDs (Years Lived with Disability)		DALYs (Disability-Adjusted Life Years)		Incidence		Prevalence		Deaths		YLLs (Years of Life Lost)		YLDs (Years Lived with Disability)		DALYs (Disability-Adjusted Life Years)	
Location	Sex	Number	Rate	Number	Rate	Number	Rate	Number	Rate	Number	Rate	Number	Rate	Number	Rate	Number	Rate	Number	Rate	Number	Rate	Number	Rate	Number	Rate
	Female	9 (7 to 11)	37.5 (29.5 to 46.4)	65 (54 to 78)	276 (229.5 to 326.5)	7 (5 to 8)	30 (23.5 to 36.8)	235 (183 to 292)	934.8 (732.2 to 1163.1)	5 (3 to 7)	21.5 (14.4 to 29.9)	241 (187 to 298)	956.2 (748.1 to 1189.2)	21 (15 to 28)	46 (33.6 to 63.5)	149 (112 to 194)	322.5 (249.9 to 416.6)	14 (10 to 20)	34.6 (25.5 to 47.6)	486 (343 to 671)	1015.6 (726 to 1406.5)	12 (7 to 18)	25.9 (16.6 to 38.5)	498 (350 to 683)	1041.6 (741.9 to 1433.2)
	Male	0 (0 to 0)	0.3 (0.2 to 0.4)	0 (0 to 0)	1.5 (1.1 to 2)	0 (0 to 0)	0.3 (0.2 to 0.3)	1 (1 to 2)	6.2 (4.3 to 8.4)	0 (0 to 0)	0.2 (0.1 to 0.2)	1 (1 to 2)	6.3 (4.4 to 8.6)	0 (0 to 0)	0.3 (0.2 to 0.5)	1 (0 to 1)	1.9 (1.4 to 2.7)	0 (0 to 0)	0.3 (0.2 to 0.4)	3 (2 to 4)	7.3 (4.8 to 10.8)	0 (0 to 0)	0.2 (0.1 to 0.3)	3 (2 to 4)	7.6 (4.9 to 11.1)
Kuwait	Both	147 (133 to 164)	15.5 (14.1 to 17.1)	1304 (1175 to 1453)	141.1 (127.8 to 156)	52 (48 to 57)	7.1 (6.4 to 7.7)	1812 (1652 to 1997)	184.3 (168.6 to 200.8)	99 (67 to 139)	10.3 (7.1 to 14.2)	1911 (1748 to 2115)	194.6 (178.1 to 212.9)	724 (570 to 930)	18.1 (14.6 to 23.2)	6817 (5541 to 8582)	178 (149.1 to 218.6)	171 (138 to 218)	5.5 (4.5 to 6.9)	5650 (4512 to 7374)	140 (112.6 to 180.2)	498 (329 to 737)	12.5 (8.4 to 18.1)	6148 (4902 to 7991)	152.5 (124 to 195.6)
	Female	145 (131 to 162)	41.3 (37.6 to 45.8)	1289 (1160 to 1439)	376.7 (340.9 to 415)	51 (47 to 55)	17.7 (16.1 to 19.4)	1785 (1626 to 1971)	499 (456.1 to 545.3)	98 (66 to 137)	27.5 (18.9 to 38.1)	1883 (1719 to 2086)	526.5 (481.8 to 578.8)	716 (563 to 923)	42.8 (34.4 to 54.7)	6751 (5464 to 8513)	423.8 (355.1 to 521.9)	168 (135 to 215)	13 (10.6 to 16.6)	5573 (4443 to 7306)	329.4 (264.7 to 426.1)	492 (324 to 730)	29.5 (19.9 to 42.9)	6066 (4831 to 7893)	358.8 (290.3 to 461.8)
	Male	2 (1 to 2)	0.5 (0.4 to 0.7)	15 (12 to 19)	3.8 (3 to 4.9)	1 (1 to 1)	0.3 (0.3 to 0.4)	27 (22 to 34)	6.4 (5.2 to 8.1)	1 (1 to 2)	0.4 (0.2 to 0.5)	28 (23 to 35)	6.8 (5.4 to 8.6)	8 (5 to 11)	0.5 (0.4 to 0.8)	66 (48 to 92)	4.2 (3 to 5.9)	3 (2 to 5)	0.2 (0.2 to 0.4)	77 (55 to 110)	4.6 (3.3 to 6.7)	6 (4 to 9)	0.4 (0.2 to 0.6)	82 (58 to 119)	5 (3.6 to 7.2)
Kyrgyzstan	Both	556 (517 to 602)	17.8 (16.5 to 19.2)	4770 (4336 to 5219)	153.2 (139.2 to 168)	325 (303 to 352)	10.6 (9.9 to 11.4)	9989 (9304 to 10801)	314.7 (293.2 to 338.9)	356 (254 to 482)	11.4 (8 to 15.3)	10345 (9628 to 11201)	326.1 (303.4 to 352.1)	651 (552 to 764)	12.5 (10.6 to 14.5)	5935 (5179 to 6823)	114.7 (100.5 to 130.9)	295 (254 to 342)	6.2 (5.4 to 7.2)	8922 (7597 to 10413)	166.8 (142.9 to 193.8)	436 (292 to 612)	8.3 (5.7 to 11.7)	9358 (7981 to 10980)	175.2 (150.8 to 204.5)
	Female	555 (516 to 601)	31.7 (29.3 to 34.4)	4760 (4327 to 5208)	270.5 (246.2 to 296.3)	324 (303 to 351)	18.3 (17.1 to 19.8)	9972 (9287 to 10782)	571.2 (531.3 to 615.7)	355 (253 to 481)	20.2 (14.4 to 27.3)	10327 (9611 to 11181)	591.4 (548.7 to 638.9)	644 (546 to 757)	22.4 (19.1 to 26.2)	5885 (5128 to 6772)	206.4 (180.8 to 236.3)	292 (251 to 338)	10.8 (9.4 to 12.5)	8826 (7501 to 10305)	302.4 (258.4 to 352.1)	431 (288 to 606)	15 (10.1 to 21)	9257 (7887 to 10867)	317.4 (272.2 to 371)
	Male	1 (1 to 1)	0.1 (0.1 to 0.1)	10 (8 to 12)	0.8 (0.6 to 0.9)	1 (0 to 1)	0 (0 to 0.1)	18 (15 to 20)	1.2 (1.1 to 1.4)	1 (1 to 1)	0.1 (0 to 0.1)	18 (16 to 21)	1.3 (1.1 to 1.5)	7 (5 to 8)	0.3 (0.2 to 0.4)	50 (39 to 63)	2.2 (1.8 to 2.8)	3 (3 to 4)	0.2 (0.1 to 0.2)	96 (76 to 120)	4.2 (3.3 to 5.2)	5 (3 to 7)	0.2 (0.1 to 0.3)	101 (80 to 126)	4.4 (3.5 to 5.5)
Lao People's Democratic Republic	Both	324 (209 to 484)	13.5 (9.1 to 20)	2171 (1515 to 3001)	90.8 (65.3 to 123.4)	253 (167 to 381)	11 (7.6 to 16.1)	8836 (5580 to 13197)	350.9 (227 to 524.4)	182 (103 to 286)	7.5 (4.4 to 11.7)	9018 (5698 to 13495)	358.4 (232.2 to 533.5)	859 (605 to 1227)	16.2 (11.6 to 22.8)	6451 (4770 to 8866)	120.1 (91.4 to 161.4)	535 (384 to 760)	10.9 (8 to 15.2)	18086 (12656 to 26172)	325.1 (231.4 to 465.1)	514 (317 to 803)	9.5 (5.9 to 14.6)	18600 (13034 to 26869)	334.6 (238.8 to 478.4)
	Female	320 (207 to 480)	25.3 (16.9 to 37.6)	2155 (1504 to 2983)	171.3 (122.4 to 234.6)	250 (165 to 376)	20.5 (14.1 to 30.1)	8746 (5527 to 13063)	664.5 (428.5 to 999)	180 (101 to 283)	14.1 (8.2 to 22.1)	8926 (5633 to 13325)	678.6 (437.7 to 1016.2)	851 (599 to 1216)	31.2 (22.4 to 44.3)	6402 (4735 to 8827)	233.4 (176.7 to 314.7)	528 (378 to 752)	20.8 (15.1 to 29.1)	17896 (12509 to 25901)	631.7 (446.3 to 907.1)	509 (313 to 796)	18.4 (11.4 to 28.4)	18404 (12854 to 26638)	650.1 (460.2 to 932.9)
	Male	4 (2 to 6)	0.4 (0.2 to 0.6)	17 (9 to 27)	1.5 (0.9 to 2.4)	3 (2 to 5)	0.3 (0.2 to 0.6)	89 (49 to 144)	8.3 (4.5 to 13.3)	2 (1 to 3)	0.2 (0.1 to 0.3)	91 (50 to 147)	8.5 (4.7 to 13.6)	9 (6 to 13)	0.4 (0.3 to 0.6)	49 (32 to 69)	2.1 (1.4 to 2.9)	7 (4 to 10)	0.4 (0.2 to 0.5)	190 (121 to 276)	8.1 (5.1 to 11.7)	5 (3 to 8)	0.2 (0.1 to 0.4)	196 (124 to 283)	8.3 (5.3 to 12)
Latvia	Both	964 (901 to 1035)	28.1 (26.3 to 30.2)	9304 (8475 to 10249)	268.7 (245.7 to 294.3)	450 (427 to 474)	12.9 (12.3 to 13.6)	12643 (11976 to 13352)	373.4 (354 to 394.8)	655 (456 to 901)	19 (13.2 to 26.1)	13297 (12595 to 14063)	392.5 (371.2 to 415)	1098 (840 to 1428)	32.1 (24.4 to 42.3)	10668 (8704 to 13174)	307.1 (246.5 to 385.5)	422 (324 to 541)	11.2 (8.5 to 14.4)	9359 (7049 to 12206)	285.5 (213.4 to 378.3)	755 (501 to 1088)	22 (14.2 to 31.9)	10114 (7692 to 13134)	307.5 (229.2 to 406)
	Female	961 (898 to 1031)	47.9 (44.8 to 51.5)	9283 (8455 to 10227)	452.4 (414.6 to 493.1)	448 (426 to 472)	21.4 (20.3 to 22.5)	12605 (11938 to 13314)	650.5 (615.7 to 689)	653 (454 to 898)	32.3 (22.3 to 44.6)	13258 (12557 to 14019)	682.8 (645.7 to 723)	1091 (834 to 1422)	55.6 (41.8 to 73.8)	10616 (8651 to 13123)	529.5 (420.7 to 674.3)	419 (322 to 538)	18.5 (14 to 24.2)	9293 (6988 to 12138)	502.2 (371.9 to 669.3)	750 (496 to 1080)	38.1 (24.4 to 55.6)	10043 (7631 to 13039)	540.3 (399.8 to 719.1)
	Male	3 (2 to 3)	0.2 (0.2 to 0.3)	21 (18 to 26)	1.6 (1.4 to 2)	2 (1 to 2)	0.1 (0.1 to 0.1)	38 (33 to 43)	2.9 (2.5 to 3.2)	2 (1 to 3)	0.2 (0.1 to 0.2)	40 (35 to 45)	3 (2.7 to 3.4)	7 (5 to 10)	0.5 (0.4 to 0.7)	52 (38 to 72)	3.9 (2.9 to 5.4)	3 (2 to 4)	0.2 (0.1 to 0.3)	66 (48 to 87)	5 (3.7 to 6.6)	5 (3 to 8)	0.4 (0.3 to 0.6)	71 (52 to 94)	5.4 (4 to 7.1)
Lebanon	Both	623 (490 to 777)	25.3 (20.1 to 31.4)	5291 (4329 to 6419)	215 (178.3 to 258.7)	320 (257 to 401)	13.7 (11.2 to 17)	10293 (8122 to 12921)	408.5 (324.6 to 510.5)	398 (264 to 562)	16.1 (10.8 to 22.5)	10691 (8411 to 13398)	424.6 (336.8 to 530.2)	3553 (2680 to 4666)	67.4 (50.7 to 88.5)	31346 (24485 to 40121)	595.1 (463.6 to 760.7)	1028 (794 to 1344)	19.8 (15.2 to 25.8)	28565 (21576 to 37191)	543.4 (410 to 711)	2354 (1526 to 3448)	44.6 (28.8 to 65.2)	30918 (23510 to 40738)	587.9 (446.3 to 774.7)
	Female	613 (480 to 767)	48.4 (38.3 to 60.4)	5225 (4267 to 6354)	414.1 (342.8 to 500.1)	313 (250 to 392)	26 (21 to 32.3)	10122 (7928 to 12750)	783.4 (617.9 to 983.4)	391 (259 to 554)	30.8 (20.5 to 43.4)	10513 (8225 to 13210)	814.3 (642.1 to 1023.2)	3519 (2655 to 4617)	122.5 (92.1 to 160.7)	31097 (24277 to 39754)	1083.5 (844.5 to 1383.5)	1012 (777 to 1320)	35.5 (27.2 to 46.4)	28245 (21306 to 36799)	985.9 (742.5 to 1290.1)	2330 (1513 to 3414)	81 (52.4 to 118.5)	30575 (23260 to 40346)	1067 (808.6 to 1407.3)
	Male	10 (7 to 14)	1 (0.7 to 1.4)	67 (47 to 92)	6 (4.4 to 8.2)	7 (5 to 11)	0.8 (0.5 to 1.2)	171 (115 to 246)	15.4 (10.4 to 21.6)	7 (4 to 10)	0.6 (0.4 to 0.9)	178 (120 to 254)	16.1 (10.9 to 22.3)	34 (23 to 51)	1.5 (1 to 2.2)	249 (174 to 353)	10.5 (7.4 to 14.9)	16 (11 to 24)	0.7 (0.5 to 1.1)	320 (217 to 459)	13.6 (9.3 to 19.5)	24 (14 to 38)	1 (0.6 to 1.6)	343 (233 to 495)	14.6 (10 to 21)

		1990												2019											
		Incidence		Prevalence		Deaths		YLLs (Years of Life Lost)		YLDs (Years Lived with Disability)		DALYs (Disability-Adjusted Life Years)		Incidence		Prevalence		Deaths		YLLs (Years of Life Lost)		YLDs (Years Lived with Disability)		DALYs (Disability-Adjusted Life Years)	
Location	Sex	Number	Rate	Number	Rate	Number	Rate	Number	Rate	Number	Rate	Number	Rate	Number	Rate	Number	Rate	Number	Rate	Number	Rate	Number	Rate	Number	Rate
Lesotho	Both	96 (72 to 128)	9.6 (7.4 to 12.7)	671 (531 to 864)	66.1 (52.9 to 83.2)	76 (59 to 101)	8.2 (6.3 to 10.8)	2169 (1588 to 2968)	204.6 (152.2 to 276.3)	54 (35 to 83)	5.3 (3.5 to 7.9)	2224 (1636 to 3044)	209.9 (157.2 to 282.9)	294 (182 to 445)	22.2 (14.2 to 33.1)	1845 (1203 to 2729)	132.4 (90.3 to 192.1)	214 (136 to 320)	17.7 (11.6 to 25.6)	6346 (3831 to 9770)	444.6 (277.4 to 670.9)	161 (90 to 259)	11.7 (6.8 to 18.4)	6506 (3913 to 10029)	456.2 (283.9 to 690.3)
	Female	91 (68 to 123)	16.2 (12.2 to 21.6)	648 (507 to 837)	114 (89.8 to 145.4)	73 (55 to 97)	13.3 (10.2 to 17.6)	2070 (1508 to 2859)	357.7 (262.1 to 489.2)	52 (33 to 80)	9.1 (5.9 to 13.8)	2121 (1544 to 2932)	366.7 (268.9 to 499.8)	286 (177 to 436)	37 (23.3 to 55.8)	1805 (1167 to 2677)	226.3 (150 to 330.7)	208 (131 to 312)	28.4 (18.1 to 41.8)	6170 (3703 to 9529)	764.3 (464.6 to 1167.5)	156 (87 to 253)	19.7 (11.3 to 31.5)	6326 (3794 to 9789)	784 (477.7 to 1202.3)
	Male	4 (3 to 6)	1 (0.7 to 1.5)	24 (16 to 33)	5.4 (3.9 to 7.4)	4 (2 to 5)	1 (0.6 to 1.4)	100 (64 to 148)	21.7 (14.2 to 32)	3 (2 to 4)	0.6 (0.4 to 0.9)	102 (65 to 152)	22.3 (14.5 to 32.8)	7 (4 to 11)	1.4 (0.8 to 2.1)	40 (25 to 61)	7 (4.6 to 10.2)	6 (3 to 9)	1.2 (0.8 to 1.8)	176 (99 to 277)	29.4 (17.4 to 45.5)	4 (2 to 7)	0.8 (0.4 to 1.3)	180 (101 to 285)	30.3 (17.8 to 46.5)
Liberia	Both	99 (79 to 124)	8.6 (6.8 to 10.6)	647 (534 to 782)	55.1 (45.7 to 66.5)	80 (64 to 99)	7.2 (5.8 to 8.8)	2473 (1943 to 3110)	203.9 (160.7 to 255)	54 (36 to 76)	4.6 (3.1 to 6.4)	2528 (1987 to 3179)	208.5 (164.6 to 261)	312 (210 to 473)	12.4 (8.6 to 18.7)	2145 (1517 to 3134)	82.5 (60.2 to 117.6)	208 (142 to 315)	9.2 (6.5 to 13.7)	6868 (4569 to 10455)	249.4 (168.2 to 379)	179 (104 to 293)	6.9 (4.1 to 11)	7047 (4696 to 10723)	256.3 (173.1 to 388.1)
	Female	99 (78 to 123)	18 (14.4 to 22.3)	642 (529 to 778)	116.5 (96.2 to 140.4)	79 (63 to 98)	15.1 (12.2 to 18.5)	2453 (1924 to 3087)	428.1 (337.5 to 535.7)	54 (36 to 76)	9.7 (6.5 to 13.5)	2506 (1968 to 3159)	437.8 (346.4 to 547.2)	310 (209 to 471)	25.5 (17.5 to 38.6)	2134 (1507 to 3120)	169.9 (123.7 to 243.9)	207 (141 to 313)	18.8 (13.2 to 28)	6825 (4540 to 10407)	514.1 (346.7 to 783.6)	178 (104 to 291)	14.1 (8.4 to 22.6)	7003 (4650 to 10662)	528.2 (356.6 to 804.2)
	Male	1 (1 to 1)	0.1 (0.1 to 0.2)	5 (3 to 6)	0.7 (0.5 to 1)	1 (1 to 1)	0.1 (0.1 to 0.2)	21 (14 to 30)	3.3 (2.3 to 4.7)	1 (0 to 1)	0.1 (0.1 to 0.1)	21 (15 to 30)	3.4 (2.4 to 4.8)	2 (1 to 3)	0.2 (0.1 to 0.3)	11 (7 to 17)	0.9 (0.6 to 1.4)	2 (1 to 2)	0.2 (0.1 to 0.2)	43 (26 to 66)	3.5 (2.2 to 5.3)	1 (1 to 2)	0.1 (0.1 to 0.2)	44 (27 to 68)	3.6 (2.2 to 5.5)
Libya	Both	216 (169 to 284)	9.8 (7.6 to 13)	1930 (1594 to 2412)	90 (74.3 to 110.4)	116 (91 to 152)	5.6 (4.4 to 7.4)	3815 (2997 to 4997)	168.1 (132 to 221.2)	142 (91 to 206)	6.5 (4.2 to 9.5)	3956 (3105 to 5213)	174.6 (136.8 to 230.4)	1365 (961 to 1906)	20.5 (14.6 to 28)	11646 (8531 to 15855)	176.2 (133.1 to 232.5)	517 (375 to 707)	8.6 (6.3 to 11.5)	17599 (12510 to 24361)	259.1 (186.9 to 352.6)	889 (548 to 1371)	13.3 (8.2 to 20)	18488 (13162 to 25563)	272.4 (195.9 to 371.8)
	Female	209 (164 to 278)	21 (16.4 to 27.8)	1886 (1551 to 2367)	193.6 (159.5 to 240.8)	111 (87 to 148)	11.7 (9.1 to 15.6)	3692 (2893 to 4873)	363.5 (283.8 to 481.5)	137 (88 to 201)	13.8 (8.9 to 20.3)	3830 (3004 to 5070)	377.4 (295.1 to 500.4)	1347 (944 to 1886)	41.4 (29.3 to 56.8)	11506 (8401 to 15649)	357.4 (268.1 to 472.2)	507 (364 to 693)	17.2 (12.5 to 23.2)	17315 (12243 to 24031)	523.6 (375.2 to 716.4)	875 (538 to 1355)	26.9 (16.6 to 40.6)	18190 (12936 to 25290)	550.5 (393.6 to 753.1)
	Male	7 (4 to 10)	0.7 (0.4 to 1)	45 (31 to 63)	4.3 (3 to 6)	5 (3 to 7)	0.5 (0.3 to 0.8)	122 (79 to 178)	11.3 (7.2 to 16.4)	4 (2 to 7)	0.4 (0.2 to 0.7)	127 (82 to 184)	11.7 (7.5 to 17)	19 (11 to 30)	0.7 (0.4 to 1.1)	140 (88 to 213)	5 (3.2 to 7.5)	11 (6 to 16)	0.4 (0.3 to 0.6)	284 (165 to 446)	9.7 (5.6 to 15)	13 (7 to 22)	0.5 (0.3 to 0.8)	298 (173 to 466)	10.1 (5.9 to 15.8)
Lithuania	Both	1198 (1117 to 1286)	27.7 (25.9 to 29.7)	11873 (10866 to 13099)	272.3 (249.5 to 299.5)	500 (474 to 526)	11.4 (10.8 to 12)	14418 (13664 to 15207)	334.5 (317.4 to 352.2)	827 (575 to 1129)	19.1 (13.3 to 26)	15245 (14400 to 16100)	353.6 (334.3 to 373.1)	1367 (1101 to 1679)	28.7 (23.1 to 35.5)	13919 (11814 to 16277)	285.3 (238.8 to 338.4)	537 (437 to 655)	10 (8 to 12.2)	12259 (9801 to 15194)	261.2 (207.2 to 326.1)	956 (658 to 1349)	20 (13.6 to 28.8)	13215 (10628 to 16387)	281.2 (223.7 to 351.1)
	Female	1193 (1112 to 1281)	48.6 (45.3 to 52.2)	11834 (10826 to 13056)	471.5 (432.6 to 517.3)	497 (472 to 523)	19.3 (18.3 to 20.3)	14359 (13599 to 15149)	590.7 (559.9 to 622.5)	823 (573 to 1124)	33.3 (23.2 to 45.7)	15182 (14342 to 16040)	624 (588.1 to 659.2)	1359 (1094 to 1670)	50.5 (40.2 to 62.8)	13856 (11761 to 16201)	498.5 (414.2 to 595.8)	534 (434 to 652)	16.7 (13.4 to 20.5)	12182 (9733 to 15092)	462.6 (365.4 to 579.5)	950 (652 to 1342)	35.1 (23.9 to 50.7)	13132 (10549 to 16270)	497.7 (394.4 to 622.1)
	Male	5 (4 to 6)	0.3 (0.2 to 0.3)	38 (32 to 45)	2.2 (1.8 to 2.5)	2 (2 to 3)	0.1 (0.1 to 0.2)	60 (52 to 68)	3.4 (3 to 3.8)	4 (3 to 5)	0.2 (0.1 to 0.3)	64 (55 to 72)	3.6 (3.1 to 4.1)	8 (6 to 11)	0.4 (0.3 to 0.5)	63 (46 to 82)	3.1 (2.3 to 4)	4 (3 to 5)	0.2 (0.1 to 0.2)	77 (56 to 100)	3.8 (2.8 to 4.9)	6 (4 to 9)	0.3 (0.2 to 0.5)	83 (61 to 108)	4.1 (3 to 5.3)
Luxembourg	Both	255 (233 to 277)	49.4 (45.4 to 53.9)	2662 (2391 to 2988)	511.3 (461.3 to 569.7)	100 (93 to 107)	19 (17.6 to 20.3)	2388 (2221 to 2544)	468.5 (434.9 to 498.9)	189 (134 to 260)	36.5 (25.9 to 50.6)	2577 (2396 to 2750)	505 (468.5 to 539.5)	382 (308 to 474)	40.9 (33 to 50.7)	4276 (3663 to 4978)	453.4 (387.5 to 529.6)	106 (90 to 124)	10.4 (8.9 to 12.2)	2214 (1912 to 2562)	237.5 (205.4 to 274.6)	293 (197 to 416)	31.4 (21 to 44.7)	2507 (2157 to 2912)	268.9 (230.9 to 312.4)
	Female	253 (232 to 275)	90.5 (82.8 to 99)	2648 (2379 to 2972)	927.9 (837.1 to 1032.2)	99 (92 to 106)	32.9 (30.5 to 35.1)	2373 (2205 to 2529)	869.2 (804.5 to 926.4)	188 (133 to 258)	66.6 (47.2 to 93.4)	2560 (2378 to 2733)	935.8 (867.2 to 1000.3)	378 (305 to 471)	79.7 (64 to 98.7)	4244 (3635 to 4942)	879.3 (749.1 to 1030.5)	105 (89 to 123)	19.2 (16.4 to 22.4)	2195 (1895 to 2541)	462.6 (400.2 to 534.5)	290 (195 to 411)	61.1 (40.8 to 86.7)	2485 (2138 to 2888)	523.6 (449.9 to 608.5)
	Male	2 (1 to 2)	0.7 (0.6 to 0.8)	14 (12 to 17)	6.3 (5.4 to 7.4)	1 (1 to 1)	0.3 (0.3 to 0.4)	16 (14 to 18)	7 (6.3 to 7.7)	1 (1 to 2)	0.6 (0.4 to 0.8)	17 (15 to 19)	7.5 (6.7 to 8.4)	3 (2 to 4)	0.7 (0.5 to 0.9)	32 (26 to 40)	7.2 (5.8 to 9)	1 (1 to 1)	0.2 (0.2 to 0.3)	20 (17 to 23)	4.4 (3.7 to 5.2)	3 (2 to 4)	0.6 (0.4 to 0.9)	22 (19 to 27)	5 (4.2 to 6)
Madagascar	Both	568 (466 to 690)	9.7 (8 to 11.5)	3729 (3136 to 4431)	62.4 (52.9 to 73)	446 (368 to 535)	8.2 (6.7 to 9.6)	15072 (12162 to 18528)	238.3 (196.2 to 288.3)	321 (217 to 450)	5.4 (3.7 to 7.4)	15394 (12394 to 18892)	243.7 (200.6 to 293.5)	1629 (1145 to 2213)	12.1 (8.9 to 16.2)	11191 (8269 to 14873)	79.8 (60.8 to 103.7)	1145 (821 to 1544)	9.6 (7.1 to 12.5)	39337 (27651 to 53513)	262.9 (188.8 to 354)	948 (603 to 1400)	6.8 (4.4 to 9.9)	40285 (28384 to 54797)	269.7 (194.3 to 362.8)
	Female	519 (421 to 643)	17.3 (14.2 to 20.9)	3497 (2906 to 4219)	115.5 (97 to 136.6)	399 (327 to 487)	14.2 (11.6 to 16.9)	14028 (11260 to 17604)	433.4 (351.2 to 533.7)	295 (197 to 416)	9.6 (6.5 to 13.4)	14323 (11499 to 17947)	443 (358.1 to 546.2)	1551 (1081 to 2124)	21.9 (15.7 to 29.8)	10774 (7928 to 14411)	147.5 (111.7 to 192.7)	1076 (761 to 1465)	16.8 (12.3 to 22.4)	37645 (26170 to 51508)	483.1 (341.6 to 657.5)	903 (564 to 1332)	12.3 (8 to 18)	38548 (26820 to 52740)	495.4 (350.1 to 674.1)

		1990												2019											
		Incidence		Prevalence		Deaths		YLLs (Years of Life Lost)		YLDs (Years Lived with Disability)		DALYs (Disability-Adjusted Life Years)		Incidence		Prevalence		Deaths		YLLs (Years of Life Lost)		YLDs (Years Lived with Disability)		DALYs (Disability-Adjusted Life Years)	
Location	Sex	Number	Rate	Number	Rate	Number	Rate	Number	Rate	Number	Rate	Number	Rate	Number	Rate	Number	Rate	Number	Rate	Number	Rate	Number	Rate	Number	Rate
	Male	49 (29 to 74)	2.1 (1.3 to 3.3)	233 (155 to 335)	9.5 (6.4 to 13.5)	47 (28 to 72)	2.2 (1.3 to 3.4)	1045 (648 to 1573)	41.4 (24.9 to 62.6)	26 (14 to 45)	1.1 (0.6 to 1.8)	1071 (666 to 1615)	42.5 (25.6 to 64.4)	78 (43 to 131)	1.8 (1 to 3)	416 (258 to 677)	8.7 (5.5 to 13.5)	68 (38 to 114)	1.8 (1 to 2.9)	1693 (942 to 2861)	33.3 (18.7 to 55.4)	44 (23 to 82)	1 (0.5 to 1.7)	1737 (973 to 2948)	34.2 (19.2 to 56.8)
Malawi	Both	421 (325 to 516)	9.8 (7.8 to 12)	2718 (2195 to 3263)	61.3 (50.3 to 73)	331 (259 to 406)	8.3 (6.6 to 10.1)	10725 (8363 to 13340)	228.3 (178 to 281)	236 (156 to 323)	5.3 (3.6 to 7.1)	10961 (8530 to 13646)	233.6 (182.6 to 287.6)	1061 (784 to 1370)	13.5 (10.3 to 17.1)	6955 (5219 to 8831)	83.2 (64.8 to 103.4)	774 (584 to 983)	11 (8.5 to 13.6)	22981 (16551 to 30374)	265 (196.5 to 341.1)	597 (368 to 867)	7.2 (4.7 to 10.4)	23578 (16926 to 31098)	272.3 (201.5 to 350)
	Female	378 (289 to 471)	16.1 (12.4 to 19.9)	2516 (2025 to 3041)	105.8 (85.9 to 126.9)	291 (225 to 362)	13.3 (10.3 to 16.2)	9793 (7536 to 12324)	388.7 (298.1 to 485.2)	213 (141 to 293)	8.9 (5.9 to 12.2)	10007 (7708 to 12606)	397.6 (306.9 to 499)	985 (716 to 1290)	22.2 (16.5 to 28.4)	6557 (4852 to 8442)	141.7 (108 to 178.4)	709 (530 to 907)	17.2 (13.2 to 21.7)	21416 (15163 to 28444)	448 (324.8 to 586.6)	555 (337 to 809)	12 (7.5 to 17.4)	21971 (15490 to 29301)	460 (333.4 to 601.9)
	Male	43 (29 to 62)	2.9 (1.8 to 4.1)	202 (144 to 283)	12.2 (8.7 to 16.8)	40 (26 to 58)	2.9 (1.8 to 4.1)	932 (622 to 1341)	54.2 (35.8 to 78.4)	23 (12 to 36)	1.4 (0.8 to 2.2)	954 (638 to 1372)	55.6 (36.8 to 80.5)	76 (48 to 111)	2.9 (1.8 to 4.1)	397 (263 to 565)	13.4 (9.3 to 18.6)	65 (42 to 95)	2.7 (1.7 to 4)	1565 (979 to 2307)	50.7 (32.8 to 73.7)	42 (23 to 70)	1.5 (0.8 to 2.3)	1607 (1007 to 2375)	52.2 (33.8 to 75.3)
Malaysia	Both	1920 (1709 to 2185)	17.3 (15.3 to 19.7)	15277 (13515 to 17336)	137.3 (121.2 to 155.5)	1140 (1014 to 1295)	11.2 (9.9 to 12.8)	37703 (33582 to 42864)	328.3 (291.3 to 373)	1178 (815 to 1609)	10.5 (7.3 to 14.4)	38881 (34540 to 44164)	338.7 (300.6 to 385)	8663 (6567 to 10970)	29.4 (22.4 to 37.2)	74153 (58424 to 91858)	249.3 (198.2 to 306.2)	3530 (2724 to 4462)	12.9 (10 to 16.2)	105906 (81203 to 135308)	355.5 (273.4 to 452.9)	5625 (3634 to 8024)	18.9 (12.4 to 26.9)	111532 (85615 to 140610)	374.4 (287.8 to 470.4)
	Female	1912 (1700 to 2176)	34.1 (30.3 to 38.9)	15230 (13476 to 17294)	270.9 (239.2 to 306.4)	1134 (1008 to 1290)	21.9 (19.4 to 25.1)	37548 (33439 to 42712)	648.7 (575.2 to 738.3)	1173 (811 to 1602)	20.6 (14.4 to 28.4)	38721 (34385 to 44008)	669.3 (594.9 to 760.3)	8630 (6541 to 10934)	59.5 (45.2 to 75.1)	73940 (58241 to 91588)	504.6 (400.8 to 620.7)	3511 (2709 to 4443)	25.8 (19.8 to 32.5)	105465 (80735 to 134740)	719 (552.4 to 915.9)	5602 (3617 to 7993)	38.2 (25.1 to 54.4)	111068 (85201 to 139953)	757.2 (581.1 to 952.1)
	Male	8 (6 to 11)	0.2 (0.1 to 0.3)	47 (36 to 61)	1 (0.7 to 1.3)	6 (5 to 8)	0.2 (0.1 to 0.2)	155 (119 to 200)	3.2 (2.4 to 4.3)	5 (3 to 8)	0.1 (0.1 to 0.2)	160 (122 to 207)	3.4 (2.5 to 4.4)	33 (22 to 47)	0.3 (0.2 to 0.4)	213 (142 to 303)	1.5 (1 to 2.2)	19 (13 to 27)	0.2 (0.1 to 0.2)	441 (307 to 609)	3.3 (2.3 to 4.5)	23 (14 to 36)	0.2 (0.1 to 0.3)	464 (322 to 638)	3.4 (2.4 to 4.7)
Maldives	Both	14 (8 to 20)	11.9 (7.2 to 17.6)	105 (66 to 147)	90.9 (59.8 to 124.7)	8 (5 to 12)	8 (5 to 11.4)	301 (171 to 446)	253.3 (148.8 to 371.8)	8 (4 to 13)	7.1 (3.7 to 11.4)	310 (176 to 456)	260.4 (152.8 to 381.3)	56 (44 to 68)	14.7 (11.7 to 17.8)	494 (405 to 595)	129.6 (107.3 to 153.5)	20 (16 to 24)	6.2 (5 to 7.5)	588 (470 to 723)	152.8 (122.6 to 187.9)	37 (25 to 53)	9.6 (6.4 to 13.6)	625 (498 to 766)	162.4 (130.9 to 198.4)
	Female	14 (8 to 20)	27.2 (16.6 to 40)	104 (65 to 147)	208 (139.4 to 281.9)	8 (5 to 12)	18.7 (12 to 26.7)	299 (170 to 444)	568.8 (337.8 to 837)	8 (4 to 13)	16.2 (8.5 to 25.7)	308 (174 to 454)	585 (347.1 to 861.4)	55 (44 to 68)	33.8 (26.9 to 40.9)	491 (402 to 592)	298.1 (247 to 355)	20 (16 to 24)	13.6 (11.1 to 16.5)	584 (466 to 718)	351.3 (282 to 430)	37 (24 to 52)	22.1 (14.8 to 31.3)	621 (494 to 761)	373.4 (299.7 to 455.8)
	Male	0 (0 to 0)	0.2 (0.1 to 0.3)	1 (0 to 1)	0.9 (0.5 to 1.3)	0 (0 to 0)	0.1 (0.1 to 0.2)	2 (1 to 3)	3.3 (2 to 5.1)	0 (0 to 0)	0.1 (0.1 to 0.2)	2 (1 to 3)	3.4 (2.1 to 5.3)	0 (0 to 0)	0.2 (0.1 to 0.3)	3 (2 to 3)	1.3 (0.9 to 1.7)	0 (0 to 0)	0.1 (0.1 to 0.1)	4 (3 to 6)	2.2 (1.6 to 2.9)	0 (0 to 0)	0.1 (0.1 to 0.2)	5 (4 to 6)	2.3 (1.7 to 3)
Mali	Both	425 (346 to 513)	9.1 (7.4 to 11)	2774 (2328 to 3260)	59.6 (50.2 to 70)	336 (274 to 405)	7.5 (6.1 to 9)	11238 (9138 to 13553)	226.8 (184.2 to 274.5)	236 (159 to 325)	5 (3.4 to 6.8)	11474 (9388 to 13830)	231.8 (188.9 to 281)	1131 (824 to 1497)	11.3 (8.4 to 14.8)	7920 (6004 to 10221)	78.9 (61.6 to 99.9)	777 (581 to 1014)	8.2 (6.3 to 10.7)	25363 (18198 to 33473)	240.4 (177 to 315.3)	653 (412 to 964)	6.4 (4.1 to 9.5)	26016 (18723 to 34415)	246.8 (181.4 to 323.1)
	Female	424 (345 to 512)	17.8 (14.5 to 21.6)	2765 (2321 to 3249)	117.1 (98.6 to 137.5)	335 (273 to 404)	14.7 (11.9 to 17.7)	11202 (9108 to 13514)	446.5 (362.4 to 541.2)	235 (158 to 322)	9.8 (6.6 to 13.4)	11437 (9356 to 13790)	456.3 (371 to 554.5)	1126 (820 to 1491)	23.2 (17.3 to 30.3)	7892 (5984 to 10192)	161.5 (126.2 to 204.5)	774 (578 to 1010)	16.9 (12.9 to 22)	25257 (18128 to 33323)	490.2 (361.2 to 642.8)	650 (410 to 961)	13.2 (8.4 to 19.5)	25907 (18646 to 34260)	503.3 (370.7 to 657.5)
	Male	1 (1 to 2)	0.1 (0 to 0.1)	8 (6 to 12)	0.3 (0.2 to 0.5)	1 (1 to 2)	0.1 (0 to 0.1)	36 (25 to 50)	1.5 (1 to 2.1)	1 (1 to 1)	0 (0 to 0.1)	37 (26 to 51)	1.5 (1 to 2.1)	5 (3 to 7)	0.1 (0.1 to 0.1)	28 (19 to 40)	0.5 (0.3 to 0.7)	4 (2 to 5)	0.1 (0.1 to 0.1)	107 (72 to 159)	1.9 (1.3 to 3)	3 (2 to 5)	0.1 (0 to 0.1)	109 (74 to 163)	2 (1.3 to 3.1)
Malta	Both	193 (176 to 212)	45.4 (41.4 to 49.7)	1953 (1764 to 2173)	458.8 (414.8 to 510.5)	80 (74 to 86)	19.1 (17.7 to 20.5)	2065 (1916 to 2220)	485.1 (449.8 to 520.5)	141 (98 to 194)	33 (22.9 to 45.4)	2206 (2049 to 2365)	518.1 (481.1 to 555.3)	318 (262 to 384)	41.6 (34.3 to 50.4)	3512 (3060 to 4049)	445 (383.7 to 514.5)	96 (82 to 111)	11.1 (9.6 to 12.8)	2031 (1753 to 2342)	271.6 (237.5 to 311.3)	242 (165 to 343)	31.4 (21.2 to 44.9)	2273 (1962 to 2644)	303 (263.6 to 350.4)
	Female	192 (175 to 211)	83.2 (75.8 to 91.2)	1941 (1753 to 2159)	837.6 (756.8 to 926.1)	79 (74 to 85)	34.1 (31.6 to 36.4)	2053 (1905 to 2208)	895.7 (831.5 to 963.1)	140 (97 to 193)	60.5 (42 to 83.2)	2193 (2036 to 2351)	956.2 (886.4 to 1025.7)	316 (260 to 381)	80.5 (66.3 to 97.5)	3486 (3036 to 4024)	858.3 (738.2 to 994.9)	95 (81 to 111)	20.6 (17.7 to 23.8)	2015 (1739 to 2326)	528.6 (461.7 to 604.8)	240 (164 to 340)	60.7 (40.8 to 87.1)	2255 (1945 to 2628)	589.3 (511.4 to 679.9)
	Male	1 (1 to 1)	0.6 (0.5 to 0.8)	11 (9 to 13)	6 (5 to 7.1)	1 (0 to 1)	0.3 (0.3 to 0.4)	12 (10 to 14)	6.4 (5.5 to 7.4)	1 (1 to 1)	0.5 (0.3 to 0.7)	13 (11 to 15)	6.9 (5.9 to 8)	2 (2 to 3)	0.6 (0.5 to 0.8)	26 (20 to 33)	6.4 (5.2 to 7.9)	1 (1 to 1)	0.2 (0.2 to 0.2)	16 (12 to 19)	4 (3.3 to 4.9)	2 (1 to 3)	0.5 (0.3 to 0.8)	18 (14 to 22)	4.6 (3.7 to 5.6)
Marshall Islands	Both	3 (3 to 4)	16.1 (13.1 to 19.6)	25 (21 to 30)	129.6 (110.2 to 151.5)	2 (2 to 3)	12.5 (10.2 to 15.2)	76 (63 to 93)	357.3 (294.1 to 435.1)	2 (1 to 3)	9.6 (6.6 to 13.3)	78 (64 to 95)	367 (302.3 to 446.9)	12 (8 to 19)	28.6 (18.9 to 42.5)	93 (64 to 135)	208.8 (149 to 294.3)	7 (5 to 11)	19.4 (13.3 to 28.2)	266 (169 to 402)	574.7 (376.7 to 859.6)	7 (4 to 12)	16.6 (10 to 26)	274 (175 to 413)	591.3 (389.4 to 883.7)

		1990												2019											
		Incidence		Prevalence		Deaths		YLLs (Years of Life Lost)		YLDs (Years Lived with Disability)		DALYs (Disability-Adjusted Life Years)		Incidence		Prevalence		Deaths		YLLs (Years of Life Lost)		YLDs (Years Lived with Disability)		DALYs (Disability-Adjusted Life Years)	
Location	Sex	Number	Rate	Number	Rate	Number	Rate	Number	Rate	Number	Rate	Number	Rate	Number	Rate	Number	Rate	Number	Rate	Number	Rate	Number	Rate	Number	Rate
	Female	3 (3 to 4)	31.9 (26 to 38.9)	25 (21 to 30)	257.5 (218.1 to 302.2)	2 (2 to 3)	24 (19.6 to 29.3)	76 (62 to 93)	722.8 (592.3 to 884.5)	2 (1 to 3)	19.2 (13.1 to 26.6)	78 (63 to 95)	741.9 (606.3 to 905.5)	12 (8 to 19)	58.3 (38.7 to 86.8)	93 (63 to 134)	426 (304.4 to 600)	7 (5 to 11)	39.6 (27.1 to 57.8)	265 (168 to 400)	1169.1 (767.2 to 1753.5)	7 (4 to 12)	33.8 (20.3 to 53)	272 (174 to 410)	1202.9 (793.8 to 1800.4)
	Male	0 (0 to 0)	0.3 (0.2 to 0.6)	0 (0 to 0)	1.8 (1.2 to 2.8)	0 (0 to 0)	0.3 (0.2 to 0.5)	1 (0 to 1)	6.7 (4.2 to 11.7)	0 (0 to 0)	0.2 (0.1 to 0.4)	1 (0 to 1)	6.9 (4.4 to 12)	0 (0 to 0)	0.4 (0.3 to 0.6)	0 (0 to 1)	2.3 (1.5 to 3.4)	0 (0 to 0)	0.3 (0.2 to 0.5)	2 (1 to 3)	8 (5 to 12.6)	0 (0 to 0)	0.3 (0.1 to 0.4)	2 (1 to 3)	8.3 (5.2 to 13)
Mauritania	Both	119 (87 to 159)	11.2 (8.3 to 14.9)	768 (588 to 983)	71.6 (55.3 to 90.9)	94 (70 to 125)	9.3 (7 to 12.3)	2858 (2066 to 3863)	256.6 (187.2 to 346)	66 (43 to 97)	6.1 (4 to 8.8)	2924 (2109 to 3941)	262.7 (191.8 to 353.3)	303 (213 to 406)	13.3 (9.4 to 17.8)	2186 (1584 to 2835)	93.9 (69.5 to 120.8)	193 (137 to 255)	9.1 (6.6 to 11.9)	5718 (3933 to 7678)	240.2 (165.6 to 320.8)	179 (111 to 266)	7.7 (4.8 to 11.2)	5897 (4075 to 7919)	247.9 (171.8 to 332.3)
	Female	118 (86 to 158)	21.4 (15.7 to 28.5)	764 (584 to 979)	136.9 (105.5 to 174.3)	94 (69 to 125)	17.5 (13.1 to 23.2)	2841 (2051 to 3841)	493.9 (360.2 to 668.8)	65 (42 to 96)	11.7 (7.6 to 17)	2906 (2098 to 3925)	505.5 (369 to 682.5)	302 (212 to 404)	26.4 (18.6 to 35.1)	2177 (1577 to 2827)	185.9 (137.6 to 238.9)	192 (136 to 254)	18.1 (13.1 to 23.6)	5689 (3919 to 7648)	473 (325.7 to 634.1)	178 (110 to 264)	15.2 (9.4 to 22.1)	5868 (4056 to 7886)	488.2 (338.6 to 654)
	Male	1 (0 to 1)	0.1 (0.1 to 0.2)	4 (3 to 5)	0.7 (0.5 to 1)	1 (0 to 1)	0.1 (0.1 to 0.2)	17 (11 to 25)	3.2 (2.1 to 4.8)	0 (0 to 1)	0.1 (0 to 0.1)	17 (12 to 26)	3.2 (2.2 to 4.9)	1 (1 to 2)	0.1 (0.1 to 0.2)	9 (5 to 14)	0.8 (0.5 to 1.2)	1 (1 to 2)	0.1 (0.1 to 0.2)	29 (17 to 46)	2.5 (1.5 to 4.1)	1 (0 to 2)	0.1 (0 to 0.1)	29 (17 to 47)	2.6 (1.5 to 4.2)
Mauritius	Both	98 (89 to 108)	11.7 (10.6 to 12.8)	883 (796 to 980)	105.6 (95.1 to 118.1)	51 (46 to 55)	6.7 (6.1 to 7.2)	1622 (1488 to 1770)	188.2 (173 to 205.3)	65 (44 to 89)	7.6 (5.2 to 10.4)	1687 (1553 to 1843)	195.9 (180.3 to 214.5)	482 (382 to 601)	27.9 (22.1 to 34.6)	4220 (3459 to 5137)	242.5 (198.3 to 294.1)	205 (165 to 252)	11.9 (9.5 to 14.5)	5821 (4631 to 7248)	334.4 (266 to 414.4)	313 (211 to 444)	18.1 (12.2 to 25.7)	6133 (4868 to 7615)	352.5 (279.7 to 437.3)
	Female	98 (89 to 108)	21.9 (20 to 24.1)	881 (793 to 978)	198.5 (178.7 to 220.9)	50 (46 to 55)	12.1 (11.1 to 13.1)	1615 (1481 to 1763)	357.3 (328.1 to 390.7)	65 (44 to 89)	14.4 (9.7 to 19.6)	1680 (1546 to 1836)	371.8 (341.7 to 407.8)	481 (380 to 599)	53.5 (42.3 to 66.4)	4208 (3449 to 5121)	465.9 (379.6 to 568)	204 (164 to 251)	22 (17.7 to 27.1)	5795 (4610 to 7213)	643.5 (511 to 799.5)	311 (210 to 442)	34.8 (23.4 to 49.5)	6106 (4847 to 7583)	678.2 (537 to 843.7)
	Male	0 (0 to 0)	0.1 (0.1 to 0.1)	3 (2 to 3)	0.6 (0.5 to 0.7)	0 (0 to 0)	0.1 (0.1 to 0.1)	7 (6 to 7)	1.7 (1.6 to 1.9)	0 (0 to 0)	0.1 (0 to 0.1)	7 (6 to 8)	1.8 (1.6 to 2)	2 (1 to 2)	0.2 (0.2 to 0.3)	13 (9 to 16)	1.5 (1.2 to 2)	1 (1 to 1)	0.1 (0.1 to 0.2)	26 (20 to 33)	3.2 (2.5 to 4)	1 (1 to 2)	0.2 (0.1 to 0.2)	27 (21 to 34)	3.3 (2.6 to 4.2)
Mexico	Both	5864 (5683 to 6032)	11.8 (11.4 to 12.2)	47821 (45777 to 49962)	96.2 (91.5 to 101.6)	2837 (2745 to 2912)	6.1 (5.9 to 6.3)	92488 (90131 to 94884)	177.4 (172.5 to 181.8)	3447 (2476 to 4597)	6.9 (4.9 to 9.1)	95935 (93264 to 98734)	184.2 (178.9 to 189.5)	24442 (19918 to 29949)	19.6 (16 to 24)	208252 (172807 to 249729)	166.9 (139 to 199.2)	8097 (6718 to 9852)	6.7 (5.6 to 8.1)	241807 (197500 to 296971)	191.3 (156.9 to 234.6)	14666 (9987 to 20413)	11.8 (8 to 16.3)	256474 (212636 to 312818)	203.1 (168.6 to 247.3)
	Female	5833 (5653 to 5999)	22.8 (22 to 23.5)	47579 (45535 to 49683)	185.2 (176.2 to 195.4)	2815 (2724 to 2889)	11.9 (11.4 to 12.2)	91849 (89503 to 94236)	341.5 (332 to 350.1)	3426 (2462 to 4570)	13.2 (9.5 to 17.5)	95275 (92615 to 98076)	354.7 (344.3 to 364.9)	24312 (19777 to 29810)	36.8 (30 to 45)	207280 (171791 to 248701)	313.4 (260.5 to 374.7)	8024 (6649 to 9777)	12.5 (10.3 to 15.2)	239911 (195552 to 295036)	359.1 (293.7 to 441.1)	14578 (9914 to 20277)	22.1 (15 to 30.6)	254489 (210687 to 310666)	381.1 (315.7 to 464.6)
	Male	31 (30 to 32)	0.1 (0.1 to 0.1)	242 (213 to 280)	1 (0.9 to 1.2)	22 (21 to 23)	0.1 (0.1 to 0.1)	639 (617 to 660)	2.6 (2.5 to 2.7)	21 (15 to 28)	0.1 (0.1 to 0.1)	660 (637 to 682)	2.7 (2.6 to 2.8)	130 (104 to 161)	0.2 (0.2 to 0.3)	972 (794 to 1193)	1.7 (1.4 to 2.1)	73 (60 to 90)	0.1 (0.1 to 0.2)	1896 (1529 to 2338)	3.3 (2.7 to 4.1)	88 (59 to 125)	0.2 (0.1 to 0.2)	1984 (1614 to 2427)	3.5 (2.8 to 4.2)
Micronesia (Federated States of)	Both	10 (7 to 13)	18.7 (14 to 24.8)	76 (60 to 97)	145.2 (116.6 to 180.2)	7 (5 to 9)	14.5 (10.7 to 18.9)	233 (174 to 316)	420.1 (314.9 to 561)	6 (4 to 9)	11 (7.2 to 15.8)	239 (178 to 323)	431.1 (322.7 to 573.6)	28 (16 to 46)	34.7 (21.1 to 56.1)	212 (130 to 335)	257.9 (167.3 to 392.7)	16 (10 to 26)	22.8 (14.7 to 35.7)	523 (290 to 867)	621.4 (366.6 to 1018.1)	17 (9 to 29)	20.4 (11.3 to 34.7)	540 (299 to 901)	641.8 (379.6 to 1051.1)
	Female	10 (7 to 13)	37.1 (27.8 to 49.5)	76 (60 to 97)	288.9 (230.8 to 361.2)	7 (5 to 9)	28.2 (20.9 to 37)	232 (173 to 314)	845.7 (633 to 1136)	6 (4 to 9)	21.9 (14.2 to 31.8)	237 (177 to 321)	867.6 (646.6 to 1161.4)	27 (16 to 46)	66.1 (39.5 to 107.4)	211 (130 to 333)	495.8 (316.3 to 765.2)	16 (10 to 26)	42.1 (26.9 to 66.5)	520 (289 to 862)	1199.1 (693.3 to 1979.4)	17 (9 to 29)	39.2 (21.3 to 67.2)	536 (297 to 896)	1238.2 (715 to 2045.4)
	Male	0 (0 to 0)	0.3 (0.2 to 0.5)	0 (0 to 1)	2 (1.5 to 3)	0 (0 to 0)	0.3 (0.2 to 0.5)	2 (1 to 3)	6.8 (4.4 to 11.3)	0 (0 to 0)	0.2 (0.1 to 0.3)	2 (1 to 3)	7.1 (4.5 to 11.6)	0 (0 to 0)	0.5 (0.3 to 0.8)	1 (1 to 2)	3 (1.9 to 4.5)	0 (0 to 0)	0.4 (0.2 to 0.6)	3 (2 to 5)	9 (5 to 14)	0 (0 to 0)	0.3 (0.2 to 0.5)	4 (2 to 5)	9.4 (5.2 to 14.5)
Monaco	Both	31 (24 to 40)	55.6 (42.4 to 71.4)	340 (277 to 414)	582.1 (472 to 712.6)	11 (8 to 14)	16.9 (12.9 to 21.4)	232 (177 to 297)	426.4 (324.4 to 550.5)	24 (16 to 34)	42 (27.3 to 60.7)	256 (196 to 327)	468.3 (356.2 to 605.3)	58 (42 to 73)	78.5 (57.3 to 101.6)	592 (474 to 719)	788.5 (623.5 to 978.4)	16 (12 to 19)	17.4 (12.9 to 21.7)	311 (229 to 393)	427 (313.1 to 549.3)	43 (28 to 62)	58.7 (38.2 to 85.7)	354 (263 to 447)	485.7 (357.5 to 631.2)
	Female	31 (23 to 40)	102.1 (77.7 to 131.6)	337 (274 to 411)	1065.2 (859.8 to 1309.4)	11 (8 to 14)	29.8 (22.7 to 38)	229 (174 to 294)	787 (597.4 to 1021.9)	24 (16 to 34)	77 (50.5 to 111.6)	253 (193 to 324)	863.9 (654.6 to 1124.3)	57 (41 to 72)	149.6 (108.7 to 193.8)	586 (469 to 713)	1505 (1188.4 to 1875.1)	16 (12 to 19)	32.1 (23.7 to 40.4)	307 (226 to 387)	813.4 (594.5 to 1046.9)	43 (28 to 62)	111.8 (72.5 to 164)	350 (259 to 443)	925.2 (678.7 to 1199.5)

		1990												2019											
		Incidence		Prevalence		Deaths		YLLs (Years of Life Lost)		YLDs (Years Lived with Disability)		DALYs (Disability-Adjusted Life Years)		Incidence		Prevalence		Deaths		YLLs (Years of Life Lost)		YLDs (Years Lived with Disability)		DALYs (Disability-Adjusted Life Years)	
Location	Sex	Number	Rate	Number	Rate	Number	Rate	Number	Rate	Number	Rate	Number	Rate	Number	Rate	Number	Rate	Number	Rate	Number	Rate	Number	Rate	Number	Rate
	Male	0 (0 to 1)	1.3 (1 to 1.9)	3 (2 to 4)	11.4 (8.5 to 15.5)	0 (0 to 0)	0.5 (0.4 to 0.7)	3 (2 to 4)	11.1 (8.1 to 14.9)	0 (0 to 0)	1.1 (0.6 to 1.6)	3 (2 to 5)	12.2 (8.9 to 16.3)	1 (1 to 1)	1.8 (1.3 to 2.5)	6 (4 to 8)	14.8 (11 to 19.8)	0 (0 to 0)	0.5 (0.4 to 0.7)	4 (3 to 6)	10.8 (7.9 to 14.6)	1 (0 to 1)	1.4 (0.9 to 2.1)	5 (3 to 6)	12.2 (9.1 to 16.3)
Mongolia	Both	75 (56 to 97)	6.4 (4.9 to 8.3)	706 (574 to 860)	63.1 (51.9 to 76)	50 (38 to 65)	4.6 (3.5 to 5.8)	1650 (1231 to 2167)	136.7 (102.3 to 179.5)	50 (32 to 73)	4.3 (2.8 to 6.3)	1701 (1267 to 2231)	141.1 (105.8 to 184)	283 (201 to 387)	9.9 (7.2 to 13.2)	2424 (1828 to 3149)	84.8 (66.2 to 107.4)	141 (101 to 191)	5.7 (4.3 to 7.6)	4640 (3288 to 6343)	154.2 (111.7 to 210)	185 (113 to 280)	6.4 (4.1 to 9.4)	4825 (3414 to 6614)	160.5 (116.2 to 217.6)
	Female	74 (55 to 97)	12.2 (9.2 to 15.9)	703 (571 to 857)	118.1 (96.3 to 143)	50 (38 to 65)	8.5 (6.5 to 10.9)	1643 (1224 to 2159)	264.8 (197.4 to 349.7)	50 (32 to 72)	8.2 (5.3 to 12)	1692 (1259 to 2221)	273 (203.6 to 359.3)	282 (200 to 385)	17.9 (12.9 to 24)	2415 (1820 to 3138)	153.2 (118.7 to 195.3)	140 (101 to 190)	9.9 (7.4 to 13.2)	4619 (3271 to 6319)	281.2 (202.7 to 383.1)	184 (112 to 278)	11.5 (7.3 to 17.1)	4804 (3397 to 6592)	292.7 (210.5 to 397.5)
	Male	0 (0 to 0)	0.1 (0 to 0.1)	3 (2 to 4)	0.5 (0.4 to 0.7)	0 (0 to 0)	0.1 (0 to 0.1)	8 (6 to 10)	1.3 (0.9 to 1.6)	0 (0 to 0)	0 (0 to 0.1)	8 (6 to 10)	1.3 (1 to 1.7)	1 (1 to 2)	0.1 (0.1 to 0.2)	9 (7 to 13)	0.8 (0.6 to 1.1)	1 (0 to 1)	0.1 (0.1 to 0.1)	20 (15 to 28)	1.7 (1.3 to 2.4)	1 (1 to 1)	0.1 (0.1 to 0.1)	21 (15 to 29)	1.8 (1.3 to 2.5)
Montenegro	Both	211 (173 to 264)	33.1 (27.3 to 41.3)	2016 (1689 to 2438)	317.4 (266.8 to 383.7)	83 (68 to 101)	13.3 (11.1 to 16.3)	2309 (1890 to 2862)	359.2 (295.2 to 445.4)	145 (97 to 211)	22.8 (15.1 to 33.1)	2454 (2012 to 3038)	382 (314.5 to 472.1)	413 (329 to 508)	45.7 (36.3 to 56.2)	3976 (3306 to 4749)	434.8 (361.3 to 519.1)	136 (111 to 164)	14.6 (11.9 to 17.6)	3427 (2734 to 4218)	379 (302.3 to 465.8)	284 (190 to 400)	31.4 (20.9 to 44.4)	3711 (2965 to 4557)	410.4 (327.9 to 505.2)
	Female	209 (172 to 262)	60.9 (50.1 to 76.3)	2001 (1675 to 2423)	581.6 (487.9 to 703.9)	82 (68 to 100)	23.8 (19.7 to 29.1)	2290 (1872 to 2838)	665 (545 to 826.3)	144 (96 to 208)	41.9 (27.9 to 61)	2434 (1993 to 3006)	706.8 (579 to 873.9)	409 (326 to 503)	86.1 (68.2 to 106.1)	3941 (3280 to 4716)	818.6 (676.2 to 981.6)	134 (109 to 163)	26.4 (21.4 to 31.9)	3390 (2704 to 4174)	714.3 (567.8 to 881.2)	281 (188 to 396)	59 (39.2 to 83.6)	3671 (2935 to 4511)	773.3 (615.6 to 954.5)
	Male	2 (1 to 3)	0.7 (0.5 to 1)	15 (12 to 22)	5.5 (4.2 to 7.5)	1 (1 to 1)	0.3 (0.2 to 0.5)	19 (14 to 28)	6.8 (4.9 to 9.7)	1 (1 to 2)	0.5 (0.3 to 0.8)	21 (15 to 30)	7.3 (5.3 to 10.3)	4 (3 to 6)	1 (0.7 to 1.4)	35 (25 to 50)	8.1 (5.9 to 11.3)	2 (1 to 2)	0.4 (0.3 to 0.6)	37 (24 to 52)	8.4 (5.7 to 11.7)	3 (2 to 5)	0.8 (0.4 to 1.2)	40 (27 to 57)	9.2 (6.3 to 12.7)
Morocco	Both	2392 (1937 to 2883)	14.7 (11.9 to 17.6)	18899 (15798 to 22450)	119 (100.2 to 140)	1508 (1222 to 1803)	9.7 (7.9 to 11.5)	54762 (44026 to 66422)	327.5 (264.1 to 394.8)	1461 (959 to 2072)	9 (5.9 to 12.8)	56223 (45172 to 68274)	336.5 (271.6 to 405.8)	9893 (7152 to 13669)	26.9 (19.5 to 36.8)	83324 (62901 to 111307)	228 (174.2 to 300.2)	4464 (3256 to 6065)	12.6 (9.3 to 16.9)	154451 (111558 to 214911)	413 (299.9 to 567.6)	6324 (3988 to 9489)	17.2 (11 to 25.4)	160775 (116654 to 222518)	430.2 (312.9 to 589.4)
	Female	2348 (1891 to 2838)	28.2 (22.6 to 33.9)	18627 (15550 to 22171)	229.6 (192.8 to 271.1)	1472 (1187 to 1766)	18.4 (14.9 to 22)	53860 (43121 to 65579)	631 (506.9 to 762.1)	1433 (933 to 2047)	17.3 (11.3 to 24.5)	55293 (44320 to 67421)	648.3 (519.8 to 783.8)	9755 (7043 to 13518)	52.5 (38.2 to 72)	82405 (62167 to 109991)	447.6 (341.1 to 590.6)	4372 (3195 to 5942)	24.4 (18.1 to 32.8)	152269 (109531 to 212953)	808.9 (585.5 to 1117.5)	6234 (3944 to 9348)	33.6 (21.6 to 49.8)	158502 (114796 to 219730)	842.5 (612.4 to 1157.9)
	Male	44 (29 to 63)	0.7 (0.4 to 1)	272 (195 to 363)	4 (2.9 to 5.3)	36 (24 to 51)	0.6 (0.4 to 0.8)	902 (598 to 1270)	12.8 (8.4 to 17.9)	27 (17 to 41)	0.4 (0.2 to 0.6)	930 (615 to 1309)	13.2 (8.6 to 18.5)	137 (84 to 205)	0.9 (0.6 to 1.4)	919 (604 to 1324)	5.9 (4 to 8.4)	92 (58 to 139)	0.7 (0.4 to 1)	2182 (1370 to 3231)	13.8 (8.5 to 20.5)	90 (51 to 142)	0.6 (0.3 to 0.9)	2272 (1425 to 3354)	14.4 (9 to 21.4)
Mozambique	Both	596 (450 to 760)	9 (7.1 to 11.2)	3845 (3067 to 4724)	55.8 (45.6 to 67)	493 (381 to 622)	8.1 (6.5 to 9.9)	16178 (12116 to 20996)	219 (167.8 to 278.7)	325 (213 to 462)	4.7 (3.2 to 6.5)	16503 (12395 to 21432)	223.7 (172.4 to 284)	1904 (1272 to 2680)	15.4 (10.9 to 21.3)	12285 (8430 to 16992)	92.4 (67 to 124.2)	1374 (955 to 1911)	12.5 (9.1 to 17)	43917 (28650 to 62920)	317.2 (215.8 to 445.8)	1061 (649 to 1607)	8.1 (5.1 to 12)	44978 (29266 to 64027)	325.3 (222 to 457.5)
	Female	594 (448 to 757)	16.7 (13.2 to 20.9)	3823 (3049 to 4701)	104.2 (84.9 to 125.2)	491 (379 to 619)	14.9 (11.9 to 18.3)	16119 (12067 to 20941)	410.8 (314.2 to 523.8)	324 (211 to 460)	8.8 (5.9 to 12.3)	16443 (12345 to 21376)	419.6 (323 to 533.1)	1898 (1267 to 2672)	27.2 (19.1 to 37.6)	12231 (8385 to 16935)	166.1 (119 to 224.2)	1368 (951 to 1904)	21.5 (15.5 to 29.4)	43751 (28507 to 62713)	572.9 (385.6 to 808.3)	1056 (644 to 1600)	14.4 (9.1 to 21.6)	44807 (29109 to 63861)	587.4 (397.4 to 827.9)
	Male	2 (2 to 3)	0.1 (0.1 to 0.1)	23 (18 to 29)	1 (0.7 to 1.3)	2 (1 to 3)	0.1 (0.1 to 0.1)	58 (43 to 79)	1.8 (1.3 to 2.5)	2 (1 to 3)	0.1 (0 to 0.1)	60 (44 to 82)	1.9 (1.4 to 2.5)	7 (5 to 9)	0.1 (0.1 to 0.2)	54 (42 to 69)	1.2 (0.9 to 1.5)	5 (4 to 7)	0.1 (0.1 to 0.2)	166 (119 to 225)	2.7 (1.9 to 3.7)	5 (3 to 7)	0.1 (0.1 to 0.1)	171 (122 to 231)	2.8 (2 to 3.8)
Myanmar	Both	6877 (4670 to 9633)	24.8 (17.6 to 34.1)	46024 (32129 to 62613)	165.4 (119.8 to 221.5)	5006 (3579 to 6838)	18.9 (13.9 to 25.4)	183742 (122718 to 255000)	636.6 (439.6 to 873)	3897 (2319 to 5922)	13.8 (8.5 to 20.6)	187639 (125842 to 259629)	650.4 (450.8 to 891)	7623 (6052 to 9658)	14.9 (12 to 18.7)	64194 (52445 to 78502)	125.2 (104.2 to 151.6)	4617 (3781 to 5787)	9.6 (8 to 11.9)	142976 (112990 to 182080)	271.8 (217.4 to 342.9)	4776 (3195 to 6862)	9.3 (6.3 to 13.2)	147752 (116978 to 188773)	281.1 (225.3 to 356.1)
	Female	6846 (4648 to 9595)	47.3 (33.3 to 65.1)	45869 (31966 to 62450)	315.8 (227.8 to 425)	4978 (3554 to 6800)	35.7 (26.1 to 48.2)	182967 (122258 to 254048)	1223.6 (839.5 to 1679.6)	3878 (2307 to 5891)	26.5 (16.1 to 39.5)	186845 (125388 to 258800)	1250 (858.5 to 1716.9)	7540 (5992 to 9563)	26.6 (21.4 to 33.5)	63731 (52085 to 77869)	224.6 (185.9 to 272.6)	4556 (3731 to 5721)	16.8 (13.9 to 20.8)	141330 (111799 to 179715)	489.1 (390.7 to 617.4)	4724 (3170 to 6785)	16.6 (11.1 to 23.7)	146054 (115899 to 186264)	505.7 (404.8 to 641)

		1990												2019											
		Incidence		Prevalence		Deaths		YLLs (Years of Life Lost)		YLDs (Years Lived with Disability)		DALYs (Disability-Adjusted Life Years)		Incidence		Prevalence		Deaths		YLLs (Years of Life Lost)		YLDs (Years Lived with Disability)		DALYs (Disability-Adjusted Life Years)	
Location	Sex	Number	Rate	Number	Rate	Number	Rate	Number	Rate	Number	Rate	Number	Rate	Number	Rate	Number	Rate	Number	Rate	Number	Rate	Number	Rate	Number	Rate
	Male	32 (17 to 51)	0.3 (0.2 to 0.5)	155 (88 to 245)	1.3 (0.7 to 2)	27 (15 to 43)	0.3 (0.2 to 0.4)	775 (421 to 1235)	6.5 (3.5 to 10.4)	18 (9 to 31)	0.2 (0.1 to 0.3)	793 (431 to 1258)	6.7 (3.6 to 10.6)	83 (52 to 122)	0.4 (0.3 to 0.6)	464 (301 to 685)	2.1 (1.4 to 3.1)	61 (40 to 88)	0.3 (0.2 to 0.5)	1646 (1043 to 2434)	7.5 (4.9 to 10.9)	52 (29 to 82)	0.3 (0.1 to 0.4)	1698 (1079 to 2519)	7.8 (5.1 to 11.3)
Namibia	Both	92 (68 to 116)	12.1 (9.2 to 15)	629 (487 to 764)	82.3 (64.6 to 98.9)	71 (54 to 88)	9.7 (7.5 to 12)	2181 (1579 to 2798)	274.9 (200 to 347.7)	52 (33 to 72)	6.7 (4.4 to 9.3)	2233 (1623 to 2859)	281.6 (204 to 356.1)	436 (299 to 638)	28 (19.6 to 40.2)	3130 (2193 to 4504)	196.8 (141.6 to 277.1)	259 (181 to 371)	17.8 (12.8 to 24.8)	7806 (5186 to 11633)	480.9 (328.7 to 704.3)	255 (152 to 401)	16 (9.7 to 24.7)	8061 (5341 to 11992)	496.9 (339 to 729)
	Female	87 (64 to 111)	21.3 (16 to 26.8)	606 (465 to 735)	147.5 (115.1 to 177.9)	67 (50 to 84)	16.7 (12.7 to 20.9)	2087 (1492 to 2685)	495.6 (356.5 to 634.9)	50 (32 to 69)	12 (7.6 to 16.6)	2136 (1533 to 2757)	507.6 (366.9 to 650.3)	423 (287 to 624)	48.2 (33.1 to 70.1)	3057 (2133 to 4410)	343.8 (244.4 to 489.3)	250 (173 to 358)	29.7 (20.9 to 41.7)	7570 (4971 to 11292)	838.7 (563.5 to 1243.1)	247 (146 to 388)	27.8 (16.6 to 43.1)	7817 (5129 to 11680)	866.4 (581.9 to 1283.1)
	Male	4 (3 to 7)	1.6 (1.1 to 2.3)	23 (16 to 32)	7.4 (5.3 to 10.2)	4 (3 to 6)	1.6 (1.1 to 2.4)	94 (60 to 139)	30 (19.9 to 43.7)	3 (2 to 4)	0.8 (0.5 to 1.3)	97 (62 to 142)	30.9 (20.4 to 44.9)	13 (9 to 18)	2.4 (1.7 to 3.3)	73 (53 to 101)	12.4 (9 to 16.6)	10 (7 to 13)	2.1 (1.4 to 2.8)	236 (165 to 327)	39.5 (28.1 to 53.7)	8 (5 to 12)	1.3 (0.8 to 2)	244 (171 to 338)	40.9 (29.1 to 55.6)
Nauru	Both	1 (1 to 2)	22.7 (14.5 to 31.2)	10 (7 to 14)	192.5 (141.4 to 249.4)	1 (0 to 1)	14.8 (9.3 to 20)	25 (16 to 35)	427.3 (275.4 to 586.8)	1 (0 to 1)	14.3 (8.6 to 21.4)	25 (17 to 36)	441.6 (286.3 to 606.9)	2 (2 to 4)	39.9 (26.6 to 54.7)	21 (14 to 28)	334.8 (244.2 to 436)	1 (1 to 2)	21.7 (15.2 to 29)	41 (26 to 59)	629 (416.3 to 872.3)	2 (1 to 3)	25.6 (15.5 to 38.2)	43 (27 to 61)	654.6 (434.4 to 904.4)
	Female	1 (1 to 2)	47.6 (30 to 65.6)	10 (7 to 14)	402.5 (293.2 to 523.5)	1 (0 to 1)	30.8 (19.2 to 41.9)	24 (16 to 34)	899.4 (578.7 to 1242.7)	1 (0 to 1)	30 (17.8 to 44.9)	25 (16 to 36)	929.4 (600.4 to 1281.2)	2 (2 to 3)	75.5 (50.3 to 104)	20 (14 to 28)	626.1 (451.4 to 818.9)	1 (1 to 2)	41.4 (28.9 to 55.2)	41 (25 to 58)	1187 (781 to 1646.6)	2 (1 to 3)	48.1 (28.9 to 72.3)	43 (26 to 61)	1235 (815.2 to 1707.5)
	Male	0 (0 to 0)	0.4 (0.2 to 0.6)	0 (0 to 0)	2.6 (1.9 to 3.8)	0 (0 to 0)	0.3 (0.2 to 0.5)	0 (0 to 0)	7.3 (4.4 to 11.6)	0 (0 to 0)	0.3 (0.2 to 0.4)	0 (0 to 0)	7.6 (4.6 to 11.9)	0 (0 to 0)	0.6 (0.3 to 0.9)	0 (0 to 0)	3.8 (2.5 to 5.7)	0 (0 to 0)	0.4 (0.3 to 0.6)	0 (0 to 0)	9.8 (5.8 to 15.5)	0 (0 to 0)	0.4 (0.2 to 0.7)	0 (0 to 0)	10.2 (6 to 16.1)
Nepal	Both	942 (631 to 1338)	8.4 (5.6 to 11.7)	6245 (4607 to 8383)	56.7 (42.2 to 74.7)	730 (485 to 1023)	6.7 (4.4 to 9.3)	24769 (16712 to 35417)	210.2 (139.9 to 295.5)	521 (327 to 781)	4.6 (2.9 to 6.9)	25290 (17021 to 36223)	214.9 (143.4 to 302.2)	3716 (2723 to 4973)	15.3 (11.3 to 20.4)	27395 (20758 to 35734)	112.6 (86 to 146)	2254 (1673 to 3002)	9.6 (7.1 to 12.7)	70200 (50970 to 94515)	283.9 (206.4 to 379.9)	2169 (1408 to 3199)	8.9 (5.8 to 13)	72369 (52531 to 97254)	292.7 (213.9 to 392.3)
	Female	929 (619 to 1325)	16.9 (11.1 to 23.7)	6183 (4546 to 8313)	115 (85.1 to 152.6)	718 (473 to 1010)	13.6 (8.8 to 18.8)	24465 (16386 to 35114)	426.5 (281.8 to 599.7)	513 (321 to 774)	9.3 (5.9 to 13.9)	24979 (16690 to 35911)	435.9 (288.3 to 613.2)	3687 (2699 to 4941)	28.8 (21.2 to 38.4)	27236 (20665 to 35584)	212.2 (162.1 to 276.3)	2231 (1654 to 2973)	18.1 (13.4 to 23.8)	69649 (50635 to 93870)	533.2 (387.8 to 712.8)	2151 (1396 to 3161)	16.7 (10.9 to 24.4)	71800 (52128 to 96448)	549.9 (401.8 to 738)
	Male	13 (8 to 19)	0.3 (0.2 to 0.5)	61 (41 to 89)	1.2 (0.8 to 1.8)	12 (8 to 18)	0.3 (0.2 to 0.5)	304 (197 to 447)	6.3 (4 to 9.3)	7 (4 to 11)	0.2 (0.1 to 0.3)	311 (201 to 456)	6.5 (4.2 to 9.5)	29 (17 to 48)	0.3 (0.2 to 0.5)	159 (98 to 256)	1.4 (0.9 to 2.3)	23 (13 to 37)	0.2 (0.1 to 0.4)	551 (325 to 908)	5.1 (3 to 8.3)	18 (10 to 33)	0.2 (0.1 to 0.3)	569 (335 to 935)	5.2 (3.1 to 8.5)
Netherlands	Both	10692 (9994 to 11397)	57.2 (53.6 to 61)	112129 (102089 to 124123)	593.2 (542.1 to 653.5)	3617 (3403 to 3770)	18.7 (17.7 to 19.5)	87136 (83861 to 90105)	471.5 (454.7 to 488)	8015 (5627 to 10807)	42.7 (29.9 to 57.6)	95151 (90977 to 99292)	514.2 (492.3 to 536.2)	16707 (12927 to 21329)	57.8 (44.5 to 74.3)	182747 (152619 to 221249)	617.5 (507.7 to 759)	4350 (3894 to 4765)	13.2 (11.9 to 14.3)	87538 (80648 to 94476)	303.2 (281.3 to 326)	12756 (8522 to 18257)	43.9 (29.2 to 63.2)	100294 (91017 to 110124)	347.1 (316.8 to 380.8)
	Female	10653 (9957 to 11359)	107.1 (100.1 to 114.3)	111747 (101746 to 123739)	1100.5 (1006.4 to 1206.8)	3601 (3388 to 3753)	33.3 (31.6 to 34.6)	86797 (83516 to 89740)	890.8 (859.5 to 920.9)	7981 (5602 to 10764)	79.7 (55.8 to 107.7)	94778 (90607 to 98923)	970.5 (929.3 to 1013)	16608 (12844 to 21207)	111.5 (85.9 to 143.9)	181807 (151682 to 220100)	1193.6 (977.4 to 1473.2)	4320 (3865 to 4729)	24.1 (22 to 26.1)	86986 (80081 to 93914)	584.8 (542.6 to 628.2)	12671 (8466 to 18141)	84.9 (56.3 to 122.9)	99656 (90341 to 109479)	669.7 (611.5 to 734.7)
	Male	39 (32 to 48)	0.5 (0.4 to 0.6)	383 (320 to 455)	4.6 (3.9 to 5.5)	15 (13 to 18)	0.2 (0.2 to 0.2)	339 (295 to 384)	4 (3.5 to 4.5)	34 (22 to 48)	0.4 (0.3 to 0.6)	373 (326 to 425)	4.4 (3.9 to 5)	99 (67 to 140)	0.6 (0.4 to 0.9)	940 (701 to 1266)	6.2 (4.6 to 8.1)	30 (24 to 37)	0.2 (0.2 to 0.2)	553 (455 to 680)	3.7 (3.1 to 4.5)	85 (51 to 131)	0.6 (0.3 to 0.8)	638 (519 to 789)	4.3 (3.5 to 5.2)
New Zealand	Both	2187 (2039 to 2351)	58.8 (54.8 to 63.2)	22908 (20894 to 25439)	609.6 (558.3 to 673.3)	657 (623 to 688)	17.5 (16.6 to 18.4)	17363 (16576 to 18136)	472.1 (450.1 to 492.8)	1637 (1140 to 2222)	43.7 (30.5 to 59.4)	18999 (18020 to 20002)	515.8 (489.6 to 543.9)	3613 (2795 to 4659)	53.5 (41.1 to 69.1)	40468 (33710 to 49889)	581.2 (477.1 to 718.4)	769 (696 to 832)	10.5 (9.6 to 11.3)	18108 (16806 to 19444)	276.9 (257.7 to 297.3)	2794 (1795 to 4042)	41 (25.9 to 59.1)	20902 (19103 to 22926)	317.8 (291.2 to 347.1)
	Female	2183 (2035 to 2346)	111.7 (104.3 to 120.3)	22877 (20863 to 25406)	1146.4 (1051.2 to 1264.6)	653 (619 to 684)	32.1 (30.5 to 33.6)	17265 (16481 to 18044)	906 (863.4 to 946)	1634 (1138 to 2218)	82.8 (57.6 to 112)	18899 (17921 to 19907)	988.8 (937.6 to 1043.5)	3605 (2787 to 4651)	101.2 (77.6 to 130.9)	40411 (33657 to 49833)	1099.2 (901.8 to 1358.8)	762 (689 to 824)	19.4 (17.9 to 20.9)	17949 (16639 to 19290)	521.4 (485.5 to 560.1)	2789 (1791 to 4035)	77.6 (49 to 111.9)	20738 (18942 to 22756)	599 (548.5 to 654.6)

		1990												2019											
		Incidence		Prevalence		Deaths		YLLs (Years of Life Lost)		YLDs (Years Lived with Disability)		DALYs (Disability-Adjusted Life Years)		Incidence		Prevalence		Deaths		YLLs (Years of Life Lost)		YLDs (Years Lived with Disability)		DALYs (Disability-Adjusted Life Years)	
Location	Sex	Number	Rate	Number	Rate	Number	Rate	Number	Rate	Number	Rate	Number	Rate	Number	Rate	Number	Rate	Number	Rate	Number	Rate	Number	Rate	Number	Rate
	Male	4 (4 to 5)	0.2 (0.2 to 0.3)	31 (25 to 38)	1.8 (1.5 to 2.2)	4 (4 to 5)	0.3 (0.2 to 0.3)	98 (84 to 114)	5.6 (4.8 to 6.5)	3 (2 to 4)	0.2 (0.1 to 0.2)	100 (86 to 117)	5.8 (5 to 6.7)	8 (6 to 10)	0.2 (0.2 to 0.3)	57 (44 to 73)	1.7 (1.3 to 2.2)	8 (6 to 9)	0.2 (0.2 to 0.3)	159 (132 to 188)	4.8 (4 to 5.6)	5 (3 to 7)	0.1 (0.1 to 0.2)	164 (136 to 194)	4.9 (4.2 to 5.8)
Nicaragua	Both	135 (112 to 156)	7.3 (6.1 to 8.5)	1139 (974 to 1305)	63 (53.8 to 72.3)	69 (57 to 79)	4 (3.3 to 4.6)	2382 (1987 to 2733)	122.6 (101.6 to 140.9)	82 (56 to 114)	4.4 (3 to 6.2)	2464 (2055 to 2825)	127 (105.5 to 146.2)	875 (698 to 1106)	18 (14.6 to 22.5)	7248 (5830 to 9065)	147.8 (120.6 to 183)	302 (248 to 371)	6.8 (5.6 to 8.1)	8685 (6910 to 10987)	173.2 (138.6 to 217.5)	520 (339 to 729)	10.6 (7 to 14.8)	9204 (7295 to 11671)	183.8 (147.4 to 230.6)
	Female	134 (111 to 154)	13.8 (11.4 to 16)	1130 (963 to 1296)	118.9 (101.5 to 136.4)	68 (56 to 78)	7.3 (6.1 to 8.5)	2349 (1956 to 2700)	231.8 (191.7 to 267)	81 (55 to 113)	8.3 (5.7 to 11.6)	2430 (2023 to 2792)	240.1 (198.9 to 277)	869 (693 to 1099)	32.8 (26.5 to 41.1)	7208 (5802 to 9019)	270.3 (219.7 to 335.8)	299 (246 to 367)	12.1 (10 to 14.6)	8600 (6831 to 10885)	316.3 (252.5 to 397.8)	516 (336 to 723)	19.4 (12.7 to 27.1)	9116 (7221 to 11567)	335.7 (268.6 to 422.4)
	Male	2 (1 to 2)	0.2 (0.2 to 0.3)	10 (7 to 12)	1.2 (0.9 to 1.6)	1 (1 to 2)	0.2 (0.1 to 0.2)	33 (26 to 42)	4 (3.1 to 5.2)	1 (1 to 1)	0.1 (0.1 to 0.2)	34 (27 to 43)	4.2 (3.2 to 5.4)	6 (4 to 8)	0.3 (0.2 to 0.4)	40 (29 to 56)	1.9 (1.3 to 2.6)	3 (2 to 5)	0.2 (0.1 to 0.2)	85 (61 to 115)	4 (2.9 to 5.4)	4 (2 to 6)	0.2 (0.1 to 0.3)	89 (64 to 120)	4.2 (3 to 5.7)
Niger	Both	191 (137 to 262)	5.8 (4.1 to 7.9)	1284 (985 to 1663)	39 (30.9 to 49)	153 (110 to 210)	5 (3.6 to 6.9)	5215 (3736 to 7207)	141.6 (101.7 to 196)	108 (69 to 157)	3.2 (2 to 4.6)	5323 (3807 to 7352)	144.8 (104 to 200.4)	671 (446 to 1012)	7.5 (5.1 to 11.1)	4627 (3262 to 6698)	50.3 (36.4 to 70.5)	479 (324 to 713)	5.8 (4 to 8.5)	15573 (10342 to 23626)	160.1 (108.2 to 239.2)	387 (219 to 647)	4.2 (2.5 to 6.8)	15959 (10594 to 24086)	164.3 (111.1 to 245.5)
	Female	190 (135 to 260)	11.8 (8.4 to 16.2)	1274 (977 to 1653)	80.1 (62.9 to 101.4)	151 (108 to 209)	10.1 (7.2 to 13.8)	5169 (3688 to 7166)	291.8 (208.8 to 403.6)	107 (68 to 156)	6.6 (4.2 to 9.5)	5276 (3763 to 7292)	298.4 (213.4 to 412.6)	666 (442 to 1006)	14.4 (9.8 to 21.4)	4597 (3235 to 6665)	96.8 (70.1 to 135.7)	475 (321 to 708)	11.2 (7.7 to 16.3)	15450 (10237 to 23447)	307.1 (207.8 to 459.4)	384 (217 to 643)	8 (4.7 to 13.1)	15833 (10538 to 23953)	315.2 (212.8 to 472.6)
	Male	2 (1 to 3)	0.1 (0.1 to 0.2)	10 (7 to 14)	0.5 (0.4 to 0.8)	2 (1 to 2)	0.1 (0.1 to 0.2)	46 (31 to 69)	2.6 (1.7 to 3.8)	1 (1 to 2)	0.1 (0 to 0.1)	47 (31 to 70)	2.7 (1.7 to 3.9)	5 (3 to 8)	0.1 (0.1 to 0.2)	30 (20 to 44)	0.7 (0.4 to 1)	4 (3 to 6)	0.1 (0.1 to 0.2)	123 (78 to 188)	2.7 (1.6 to 4.2)	3 (2 to 5)	0.1 (0 to 0.1)	126 (80 to 192)	2.8 (1.7 to 4.3)
Nigeria	Both	5361 (3797 to 7480)	11.4 (8.2 to 15.6)	36169 (27166 to 48013)	76.1 (58 to 99)	4142 (2960 to 5893)	9.3 (6.8 to 13.1)	124185 (86572 to 180408)	246.8 (174.6 to 355.9)	2975 (1851 to 4575)	6.2 (3.9 to 9.4)	127159 (89327 to 183731)	253 (179.9 to 364.2)	21190 (14416 to 29569)	20.6 (14.4 to 28.2)	149536 (105086 to 207459)	142.1 (103 to 190.8)	13385 (9417 to 18494)	14.3 (10.3 to 19.2)	424625 (293684 to 611824)	386.8 (270.6 to 537.6)	12294 (7458 to 18513)	11.6 (7.3 to 17.1)	436920 (303424 to 621718)	398.5 (281.2 to 550.4)
	Female	5330 (3772 to 7445)	24.3 (17.3 to 33.7)	35942 (26959 to 47769)	163 (123.3 to 214.9)	4116 (2936 to 5866)	19.3 (13.8 to 27.4)	123430 (86014 to 179711)	545 (381.9 to 790.8)	2954 (1837 to 4544)	13.3 (8.4 to 20.5)	126384 (88635 to 183043)	558.3 (392 to 806.8)	21121 (14363 to 29478)	38.8 (27 to 52.9)	149039 (104533 to 206888)	266.7 (194 to 357.6)	13331 (9350 to 18430)	27 (19.5 to 36.3)	423152 (292216 to 610423)	724.2 (506.4 to 1006.2)	12248 (7421 to 18455)	21.8 (13.7 to 32)	435400 (302154 to 619864)	746.1 (527.3 to 1029.7)
	Male	31 (21 to 45)	0.1 (0.1 to 0.2)	228 (165 to 308)	0.9 (0.7 to 1.2)	26 (17 to 38)	0.1 (0.1 to 0.2)	755 (510 to 1092)	2.9 (1.9 to 4.1)	21 (13 to 32)	0.1 (0.1 to 0.1)	775 (525 to 1120)	2.9 (2 to 4.3)	70 (49 to 98)	0.2 (0.1 to 0.2)	497 (361 to 670)	1.1 (0.8 to 1.4)	54 (39 to 76)	0.1 (0.1 to 0.2)	1473 (1021 to 2133)	3.1 (2.2 to 4.5)	47 (29 to 73)	0.1 (0.1 to 0.2)	1520 (1068 to 2180)	3.3 (2.3 to 4.6)
Niue	Both	0 (0 to 1)	22.5 (16.2 to 30.1)	4 (3 to 5)	193 (150.7 to 243.3)	0 (0 to 0)	14 (10.4 to 18.4)	8 (6 to 10)	376.9 (269.7 to 502.8)	0 (0 to 0)	14.1 (8.9 to 20.5)	8 (6 to 11)	391 (281.6 to 523.4)	1 (0 to 1)	35.5 (23.2 to 48.8)	6 (4 to 8)	291.7 (208 to 384.2)	0 (0 to 0)	16.5 (11 to 21.9)	9 (6 to 12)	431.5 (281.9 to 592.7)	0 (0 to 1)	22.7 (13.9 to 33.8)	9 (6 to 13)	454.2 (297.5 to 623.5)
	Female	0 (0 to 1)	41.4 (29.5 to 55.9)	4 (3 to 5)	358.5 (277.5 to 454.5)	0 (0 to 0)	24.6 (18.2 to 32.7)	8 (6 to 10)	705.6 (498.4 to 948.3)	0 (0 to 0)	26.3 (16.4 to 38.6)	8 (6 to 11)	731.9 (520.7 to 986.4)	1 (0 to 1)	67.6 (43.8 to 93.3)	6 (4 to 8)	560.6 (395.6 to 744.5)	0 (0 to 0)	30.1 (20.1 to 40.3)	9 (6 to 12)	828.8 (538.4 to 1145.3)	0 (0 to 1)	43.5 (26.4 to 65)	9 (6 to 13)	872.2 (567.4 to 1201)
	Male	0 (0 to 0)	0.4 (0.2 to 0.5)	0 (0 to 0)	2.4 (1.8 to 3.3)	0 (0 to 0)	0.3 (0.2 to 0.4)	0 (0 to 0)	5.9 (3.8 to 8.6)	0 (0 to 0)	0.2 (0.1 to 0.4)	0 (0 to 0)	6.2 (4 to 9)	0 (0 to 0)	0.6 (0.4 to 0.8)	0 (0 to 0)	3.7 (2.6 to 5.1)	0 (0 to 0)	0.3 (0.2 to 0.5)	0 (0 to 0)	7.4 (5 to 10.6)	0 (0 to 0)	0.4 (0.2 to 0.6)	0 (0 to 0)	7.8 (5.2 to 11.2)
North Macedonia	Both	498 (442 to 558)	24.8 (22 to 27.8)	4803 (4284 to 5403)	242.6 (215.6 to 274.5)	240 (214 to 268)	12.4 (11.1 to 14)	7624 (6811 to 8500)	373.8 (334.4 to 416.6)	338 (228 to 465)	16.9 (11.4 to 23.1)	7962 (7128 to 8862)	390.6 (350.5 to 435.6)	1168 (882 to 1513)	37.5 (28.3 to 48.8)	10952 (8753 to 13676)	350.6 (281 to 437.6)	430 (335 to 542)	14.1 (11 to 17.6)	11139 (8461 to 14233)	357.5 (271.8 to 456.6)	787 (503 to 1162)	25.2 (16.1 to 37.4)	11925 (9034 to 15302)	382.7 (291.1 to 488)
	Female	490 (434 to 551)	47.4 (42.1 to 53.3)	4738 (4216 to 5336)	463.2 (411.4 to 524.4)	236 (210 to 264)	23.5 (21 to 26.3)	7510 (6708 to 8377)	718.6 (643.1 to 801.9)	332 (224 to 458)	32.2 (21.8 to 44.2)	7842 (7012 to 8739)	750.8 (673 to 836.5)	1152 (869 to 1493)	72.9 (54.8 to 95.1)	10816 (8639 to 13501)	681.1 (542.7 to 854.5)	423 (330 to 533)	26.5 (20.6 to 33.2)	10983 (8336 to 14053)	695.5 (526.2 to 891.4)	774 (493 to 1145)	49 (31.2 to 72.7)	11757 (8898 to 15071)	744.5 (563.7 to 953.4)
	Male	8 (7 to 9)	0.8 (0.7 to 1)	65 (55 to 77)	7.1 (6 to 8.3)	4 (4 to 5)	0.5 (0.4 to 0.6)	115 (99 to 133)	12 (10.4 to 14)	6 (4 to 8)	0.6 (0.4 to 0.9)	120 (104 to 141)	12.6 (11 to 14.7)	16 (11 to 22)	1.1 (0.8 to 1.4)	135 (101 to 183)	9 (6.9 to 11.9)	6 (5 to 8)	0.5 (0.3 to 0.6)	156 (115 to 208)	10.3 (7.7 to 13.7)	12 (8 to 18)	0.8 (0.5 to 1.2)	168 (124 to 224)	11.1 (8.4 to 14.7)

		1990												2019											
		Incidence		Prevalence		Deaths		YLLs (Years of Life Lost)		YLDs (Years Lived with Disability)		DALYs (Disability-Adjusted Life Years)		Incidence		Prevalence		Deaths		YLLs (Years of Life Lost)		YLDs (Years Lived with Disability)		DALYs (Disability-Adjusted Life Years)	
Location	Sex	Number	Rate	Number	Rate	Number	Rate	Number	Rate	Number	Rate	Number	Rate	Number	Rate	Number	Rate	Number	Rate	Number	Rate	Number	Rate	Number	Rate
Northern Mariana Islands	Both	7 (5 to 9)	23.3 (17.9 to 29.7)	61 (46 to 76)	205.3 (164.3 to 251.3)	3 (2 to 3)	12.5 (9.7 to 15.6)	102 (74 to 131)	325.4 (247.4 to 411.6)	5 (3 to 7)	15 (9.7 to 21)	106 (78 to 137)	340.5 (259 to 431.3)	18 (14 to 23)	32.1 (25.2 to 40.4)	171 (140 to 209)	298.9 (246.7 to 361.8)	7 (5 to 9)	13.9 (11.3 to 17.2)	203 (158 to 261)	355.2 (282.3 to 449.7)	12 (8 to 18)	21.3 (14.1 to 30.7)	215 (167 to 275)	376.5 (299 to 475)
	Female	7 (5 to 9)	56.8 (43.2 to 72.6)	60 (46 to 76)	503.9 (400.5 to 619.9)	3 (2 to 3)	29.1 (22.6 to 36.4)	101 (74 to 131)	803.8 (607.3 to 1021.1)	5 (3 to 7)	36.9 (23.7 to 52)	106 (78 to 137)	840.6 (636.9 to 1064.9)	18 (14 to 23)	65.6 (51.6 to 82.7)	170 (139 to 208)	613.7 (505.4 to 744.2)	7 (5 to 9)	27.5 (22.2 to 34.3)	202 (157 to 260)	729.2 (577.1 to 925.7)	12 (8 to 18)	43.8 (28.7 to 63.4)	214 (166 to 273)	773 (612.7 to 979.8)
	Male	0 (0 to 0)	0.1 (0.1 to 0.2)	0 (0 to 0)	1.6 (1.3 to 2.2)	0 (0 to 0)	0.1 (0.1 to 0.1)	0 (0 to 0)	2.2 (1.5 to 3.2)	0 (0 to 0)	0.1 (0.1 to 0.2)	0 (0 to 0)	2.3 (1.6 to 3.4)	0 (0 to 0)	0.3 (0.2 to 0.4)	1 (1 to 1)	2.7 (2.1 to 3.4)	0 (0 to 0)	0.2 (0.1 to 0.2)	1 (1 to 1)	3.6 (2.6 to 4.9)	0 (0 to 0)	0.2 (0.1 to 0.3)	1 (1 to 2)	3.8 (2.8 to 5.2)
Norway	Both	2077 (1958 to 2194)	35.1 (33.1 to 37.1)	23287 (20823 to 26232)	381.7 (347.4 to 423.8)	875 (821 to 910)	13.7 (13 to 14.2)	19701 (18904 to 20324)	348.8 (336.2 to 359.2)	1585 (1107 to 2154)	26.5 (18.5 to 36.1)	21286 (20351 to 22151)	375.3 (359.5 to 389.8)	2927 (2351 to 3688)	35 (27.9 to 44.2)	34227 (29035 to 40158)	397.4 (335.1 to 470.5)	797 (706 to 871)	8.3 (7.5 to 9)	16050 (14808 to 17364)	191.9 (178 to 207.1)	2291 (1526 to 3299)	27.2 (18.1 to 39.7)	18341 (16769 to 20127)	219.1 (201.5 to 240.6)
	Female	2017 (1900 to 2130)	64.6 (61 to 68.3)	22774 (20369 to 25700)	698.1 (636.9 to 772.3)	850 (796 to 885)	24.3 (23.1 to 25.1)	19175 (18390 to 19800)	651.5 (629 to 671.6)	1539 (1072 to 2091)	48.6 (33.8 to 66.1)	20713 (19771 to 21577)	700.1 (671.9 to 727.5)	2860 (2282 to 3605)	67.7 (53.6 to 85.8)	33510 (28411 to 39431)	766.8 (645.2 to 913.1)	777 (688 to 851)	15.3 (13.9 to 16.6)	15663 (14437 to 16940)	370.6 (344.2 to 400.2)	2233 (1482 to 3227)	52.6 (34.6 to 76.9)	17896 (16351 to 19669)	423.2 (389.1 to 465.2)
	Male	60 (52 to 69)	2.2 (1.9 to 2.5)	514 (443 to 598)	18.6 (16.1 to 21.5)	24 (23 to 26)	0.9 (0.8 to 0.9)	526 (503 to 549)	19.7 (18.9 to 20.6)	47 (32 to 64)	1.7 (1.2 to 2.3)	573 (544 to 603)	21.4 (20.3 to 22.5)	68 (51 to 89)	1.6 (1.2 to 2.1)	717 (574 to 888)	16.6 (13.3 to 20.4)	20 (18 to 22)	0.5 (0.4 to 0.5)	387 (348 to 428)	9.2 (8.3 to 10.2)	58 (39 to 84)	1.3 (0.9 to 2)	445 (396 to 496)	10.6 (9.4 to 11.8)
Oman	Both	75 (52 to 107)	9.3 (6.6 to 13.3)	707 (535 to 939)	91.9 (71.2 to 118.6)	40 (28 to 57)	5.8 (4.1 to 8.2)	1273 (887 to 1815)	147.5 (104.6 to 210.7)	51 (31 to 79)	6.3 (4 to 9.7)	1324 (924 to 1885)	153.8 (109.1 to 220.3)	448 (365 to 534)	19.5 (16.3 to 22.9)	4075 (3399 to 4798)	178.7 (154 to 205.2)	133 (111 to 158)	7.7 (6.5 to 9)	4199 (3449 to 5001)	175 (146.4 to 207)	305 (205 to 434)	13 (8.8 to 17.9)	4504 (3688 to 5378)	188 (157.8 to 222.1)
	Female	68 (46 to 99)	19.3 (13.2 to 28.4)	655 (489 to 882)	193.9 (147.5 to 257)	35 (24 to 52)	10.9 (7.5 to 16.2)	1134 (765 to 1655)	312.9 (211.5 to 462.9)	46 (28 to 73)	13.2 (8.1 to 20.7)	1180 (795 to 1718)	326 (221.6 to 479.8)	427 (346 to 511)	44.7 (36.8 to 52.9)	3894 (3232 to 4600)	413.8 (351.7 to 479)	124 (102 to 147)	15.9 (13.3 to 18.9)	3946 (3223 to 4725)	404.9 (335.3 to 480.9)	289 (193 to 412)	29.9 (20.2 to 41.7)	4235 (3446 to 5087)	434.8 (359.1 to 519)
	Male	7 (5 to 11)	2.3 (1.5 to 3.4)	52 (35 to 73)	13.6 (9.4 to 18.8)	5 (3 to 7)	1.8 (1.2 to 2.7)	139 (88 to 207)	35.5 (22.8 to 50.8)	5 (3 to 8)	1.4 (0.7 to 2.1)	144 (92 to 212)	36.9 (23.7 to 52.8)	21 (14 to 31)	2.5 (1.7 to 3.7)	181 (126 to 254)	18 (12.9 to 25.1)	9 (6 to 13)	1.4 (1 to 2.1)	253 (166 to 369)	24.6 (16.7 to 34.4)	16 (9 to 25)	1.7 (1 to 2.6)	269 (178 to 393)	26.3 (17.7 to 37)
Pakistan	Both	12821 (9291 to 17372)	20.3 (14.6 to 27.7)	86028 (66842 to 111841)	135.1 (105.5 to 174.6)	9807 (7126 to 13283)	16.2 (11.7 to 22.1)	318724 (234369 to 424814)	481.1 (352.4 to 646.2)	7092 (4533 to 10762)	11 (7.1 to 16.8)	325816 (239525 to 433840)	492.2 (360.7 to 660.9)	51438 (37937 to 69293)	38.3 (28.8 to 51)	370695 (282410 to 487082)	269.5 (210 to 347.2)	32118 (24388 to 43374)	26.3 (20.2 to 34.9)	1089396 (818685 to 1490050)	761.6 (577.4 to 1028.9)	29982 (19495 to 45430)	21.7 (14.3 to 32.2)	1119378 (847412 to 1520925)	783.3 (597.3 to 1054.5)
	Female	12284 (8753 to 16839)	41.8 (29.5 to 58.2)	83434 (64416 to 109224)	283.6 (219.1 to 370.3)	9290 (6592 to 12742)	33.2 (23.3 to 46.3)	307813 (224089 to 413383)	994.5 (715.6 to 1349.8)	6808 (4321 to 10454)	22.9 (14.5 to 35)	314621 (228696 to 422261)	1017.4 (733.9 to 1381.6)	50293 (36575 to 68299)	76.5 (56.1 to 102.5)	364325 (275411 to 480500)	542.8 (418 to 704.5)	31177 (23333 to 42602)	51.9 (39 to 69.8)	1066965 (793526 to 1468573)	1526.7 (1141 to 2089.1)	29324 (18998 to 44191)	43.4 (28.3 to 64.7)	1096288 (818710 to 1501821)	1570.1 (1177.2 to 2135.5)
	Male	537 (413 to 697)	1.9 (1.4 to 2.4)	2594 (2091 to 3266)	8.4 (6.8 to 10.6)	517 (391 to 668)	1.9 (1.4 to 2.5)	10911 (8647 to 13973)	35.4 (27.7 to 45.3)	283 (187 to 403)	0.9 (0.6 to 1.3)	11194 (8856 to 14337)	36.3 (28.4 to 46.5)	1144 (798 to 1587)	2.3 (1.6 to 3.2)	6370 (4561 to 8752)	11.8 (8.7 to 15.9)	941 (665 to 1299)	2.1 (1.5 to 2.9)	22431 (15671 to 31116)	40 (28.4 to 54.9)	658 (408 to 971)	1.3 (0.8 to 1.8)	23090 (16281 to 31864)	41.3 (29.4 to 56.5)
Palau	Both	3 (2 to 4)	27.6 (19.8 to 36.5)	26 (20 to 33)	230.9 (179.1 to 290.8)	2 (1 to 2)	15.9 (11.5 to 20.8)	50 (35 to 67)	433.5 (308.5 to 578.3)	2 (1 to 3)	17.4 (11 to 25.3)	52 (37 to 70)	450.9 (321.9 to 603.6)	8 (6 to 11)	36.2 (26.9 to 47.3)	73 (57 to 93)	307.1 (243.1 to 383.4)	3 (3 to 5)	17.1 (12.9 to 21.5)	105 (76 to 138)	441.1 (323.2 to 572.9)	5 (3 to 8)	23.4 (15.1 to 33.4)	110 (80 to 145)	464.4 (341.8 to 598.9)
	Female	3 (2 to 4)	54.6 (39 to 72.6)	26 (20 to 33)	458.8 (354.6 to 578.8)	2 (1 to 2)	30.8 (22.1 to 40.3)	49 (35 to 66)	862.5 (612.6 to 1154)	2 (1 to 3)	34.6 (21.9 to 50.5)	51 (37 to 69)	897.1 (638 to 1202.2)	8 (6 to 11)	75.3 (55.3 to 99)	73 (57 to 93)	642 (502.5 to 808.3)	3 (3 to 4)	34 (25.5 to 43.1)	104 (76 to 138)	923.8 (675.2 to 1208)	5 (3 to 8)	48.9 (31.3 to 70.4)	110 (79 to 145)	972.7 (713.6 to 1264.4)
	Male	0 (0 to 0)	0.2 (0.2 to 0.4)	0 (0 to 0)	1.8 (1.3 to 2.7)	0 (0 to 0)	0.2 (0.1 to 0.3)	0 (0 to 0)	3.9 (2.5 to 6.4)	0 (0 to 0)	0.2 (0.1 to 0.3)	0 (0 to 0)	4.1 (2.6 to 6.6)	0 (0 to 0)	0.3 (0.2 to 0.5)	0 (0 to 0)	2.2 (1.5 to 3.3)	0 (0 to 0)	0.2 (0.1 to 0.3)	0 (0 to 1)	4.3 (2.7 to 6.5)	0 (0 to 0)	0.2 (0.1 to 0.4)	1 (0 to 1)	4.5 (2.9 to 6.8)

		1990												2019											
		Incidence		Prevalence		Deaths		YLLs (Years of Life Lost)		YLDs (Years Lived with Disability)		DALYs (Disability-Adjusted Life Years)		Incidence		Prevalence		Deaths		YLLs (Years of Life Lost)		YLDs (Years Lived with Disability)		DALYs (Disability-Adjusted Life Years)	
Location	Sex	Number	Rate	Number	Rate	Number	Rate	Number	Rate	Number	Rate	Number	Rate	Number	Rate	Number	Rate	Number	Rate	Number	Rate	Number	Rate	Number	Rate
Palestine	Both	177 (125 to 253)	18.4 (12.9 to 26.2)	1492 (1126 to 2021)	155.4 (117.9 to 209)	97 (69 to 138)	10.7 (7.6 to 15.3)	3114 (2201 to 4422)	315.7 (220.5 to 447.1)	113 (69 to 178)	11.6 (7.1 to 18.3)	3227 (2282 to 4578)	327.2 (229.9 to 465.9)	845 (693 to 1019)	29.4 (24.1 to 35.3)	7064 (5892 to 8412)	241.4 (203.1 to 285.3)	339 (280 to 405)	13.5 (11.2 to 16.1)	10684 (8802 to 12718)	357.8 (295.9 to 427.1)	541 (366 to 763)	18.4 (12.7 to 25.7)	11225 (9233 to 13356)	376.2 (310.7 to 447.3)
	Female	176 (124 to 252)	33.5 (23.4 to 47.8)	1483 (1117 to 2010)	282.7 (214.4 to 381.2)	96 (68 to 137)	19.2 (13.6 to 27.6)	3092 (2184 to 4404)	574.5 (400.6 to 816.8)	112 (68 to 177)	21.1 (13 to 33.2)	3203 (2251 to 4558)	595.6 (417.5 to 845.3)	840 (688 to 1012)	57.1 (46.9 to 68.8)	7020 (5848 to 8365)	472.5 (396.9 to 558.4)	336 (278 to 402)	25.5 (21.2 to 30.5)	10601 (8731 to 12616)	702.9 (581.1 to 839.2)	537 (363 to 757)	36 (24.7 to 50.3)	11138 (9150 to 13244)	738.9 (609.5 to 879)
	Male	1 (1 to 2)	0.3 (0.2 to 0.4)	10 (7 to 13)	2.3 (1.7 to 3.2)	1 (1 to 1)	0.2 (0.2 to 0.4)	23 (15 to 33)	5.2 (3.4 to 7.8)	1 (1 to 1)	0.2 (0.1 to 0.3)	24 (16 to 34)	5.4 (3.6 to 8.1)	5 (4 to 7)	0.5 (0.4 to 0.6)	43 (33 to 57)	3.5 (2.7 to 4.5)	3 (2 to 4)	0.3 (0.2 to 0.4)	83 (61 to 109)	6.4 (4.8 to 8.4)	4 (2 to 6)	0.3 (0.2 to 0.5)	87 (64 to 114)	6.7 (5.1 to 8.9)
Panama	Both	239 (218 to 263)	14.6 (13.4 to 16)	1953 (1780 to 2142)	119.3 (108.7 to 130.4)	105 (97 to 114)	6.7 (6.2 to 7.3)	3234 (2987 to 3518)	192.1 (177.8 to 208.6)	141 (101 to 188)	8.6 (6.1 to 11.5)	3375 (3109 to 3671)	200.7 (185.6 to 218.2)	925 (699 to 1183)	22.1 (16.7 to 28.2)	7861 (6062 to 9848)	188.1 (145.3 to 235.9)	297 (228 to 376)	7.1 (5.4 to 9)	8083 (6089 to 10447)	192.4 (144.9 to 248.8)	554 (359 to 820)	13.3 (8.6 to 19.6)	8637 (6503 to 11131)	205.7 (155 to 264.8)
	Female	233 (212 to 257)	28.9 (26.4 to 31.7)	1918 (1745 to 2109)	237.6 (215.9 to 260.3)	101 (93 to 110)	13 (11.9 to 14.1)	3134 (2887 to 3416)	377.3 (348 to 410.5)	137 (98 to 184)	16.9 (12.1 to 22.7)	3271 (3008 to 3564)	394.2 (363.1 to 430.2)	916 (692 to 1171)	43.1 (32.6 to 55.2)	7798 (6014 to 9776)	368.5 (284.3 to 462.4)	291 (224 to 370)	13.5 (10.4 to 17.1)	7966 (5990 to 10301)	375 (282.1 to 485.1)	548 (354 to 812)	25.9 (16.7 to 38.4)	8514 (6405 to 10975)	400.9 (301.4 to 517)
	Male	6 (5 to 7)	0.8 (0.7 to 0.9)	35 (29 to 42)	4.5 (3.8 to 5.4)	4 (4 to 5)	0.6 (0.5 to 0.7)	100 (87 to 115)	12.8 (11.1 to 14.7)	4 (2 to 5)	0.5 (0.3 to 0.6)	104 (90 to 118)	13.3 (11.5 to 15.3)	9 (6 to 13)	0.5 (0.3 to 0.6)	63 (44 to 87)	3.1 (2.2 to 4.3)	5 (4 to 7)	0.3 (0.2 to 0.3)	117 (82 to 160)	5.7 (4 to 7.8)	6 (4 to 9)	0.3 (0.2 to 0.5)	123 (86 to 169)	6 (4.2 to 8.3)
Papua New Guinea	Both	585 (433 to 769)	23.8 (17.8 to 30.8)	4023 (3112 to 5070)	165.8 (132.2 to 206.2)	405 (301 to 528)	17.6 (13.3 to 22.6)	15189 (11170 to 19858)	587.2 (434.2 to 762.1)	340 (215 to 502)	13.7 (8.8 to 19.7)	15529 (11428 to 20394)	600.9 (444.5 to 781.8)	2078 (1508 to 2827)	30.9 (22.9 to 41.5)	14907 (11311 to 19751)	222.1 (173.2 to 286.8)	1287 (944 to 1725)	20.8 (15.6 to 27.7)	48651 (35406 to 65966)	686.1 (503.5 to 918.2)	1237 (779 to 1819)	18.1 (11.8 to 26.3)	49888 (36209 to 67731)	704.2 (515.7 to 938.7)
	Female	583 (430 to 766)	49.3 (37 to 63.9)	4011 (3095 to 5053)	343.3 (272.6 to 427.9)	404 (301 to 526)	36.4 (27.4 to 46.8)	15138 (11115 to 19806)	1222.3 (904.7 to 1590.4)	339 (214 to 501)	28.4 (18.2 to 41.1)	15477 (11345 to 20339)	1250.7 (923.8 to 1627.9)	2071 (1503 to 2815)	64.5 (47.7 to 86.7)	14863 (11272 to 19688)	464 (361.7 to 597)	1281 (937 to 1718)	43.5 (32.5 to 57.7)	48475 (35196 to 65770)	1430.2 (1049.2 to 1912.6)	1232 (776 to 1814)	37.8 (24.6 to 54.9)	49707 (35973 to 67466)	1468 (1077.2 to 1956.4)
	Male	2 (1 to 4)	0.2 (0.1 to 0.4)	12 (7 to 21)	1.2 (0.7 to 2)	2 (1 to 3)	0.2 (0.1 to 0.4)	51 (27 to 95)	4.8 (2.4 to 9.1)	1 (1 to 3)	0.1 (0.1 to 0.3)	53 (28 to 98)	4.9 (2.5 to 9.3)	8 (4 to 14)	0.3 (0.2 to 0.6)	44 (26 to 76)	1.6 (1 to 2.8)	6 (3 to 12)	0.3 (0.1 to 0.5)	176 (94 to 328)	6.2 (3.3 to 11.9)	5 (2 to 9)	0.2 (0.1 to 0.4)	181 (97 to 338)	6.4 (3.4 to 12.3)
Paraguay	Both	287 (245 to 336)	11.8 (10 to 13.7)	2297 (1998 to 2642)	94 (81.4 to 107.6)	158 (135 to 182)	6.8 (5.8 to 7.8)	4876 (4195 to 5650)	192.5 (165.2 to 222.4)	166 (115 to 232)	6.8 (4.6 to 9.3)	5042 (4336 to 5853)	199.3 (170.8 to 231.2)	1348 (987 to 1737)	22.8 (16.9 to 29.3)	10992 (8259 to 14018)	184.9 (140.2 to 234.1)	540 (409 to 686)	9.5 (7.2 to 12.1)	15512 (11367 to 20090)	257 (190.6 to 332.2)	794 (508 to 1169)	13.4 (8.6 to 19.6)	16306 (12042 to 21214)	270.3 (201 to 350.3)
	Female	285 (243 to 333)	22.8 (19.4 to 26.5)	2285 (1987 to 2630)	182.9 (158.4 to 209.2)	156 (133 to 181)	12.9 (11.1 to 14.9)	4837 (4158 to 5607)	376.3 (322.6 to 436.4)	165 (114 to 230)	13.1 (9 to 18.1)	5002 (4299 to 5811)	389.4 (332.6 to 452.6)	1341 (982 to 1726)	44.3 (32.7 to 57)	10945 (8221 to 13956)	361.5 (272.8 to 459.3)	536 (406 to 682)	18 (13.7 to 23)	15399 (11264 to 19958)	502.3 (370.8 to 650)	790 (505 to 1162)	26.1 (16.7 to 38.3)	16189 (11950 to 21080)	528.4 (392.2 to 686.1)
	Male	2 (2 to 2)	0.2 (0.1 to 0.2)	12 (9 to 15)	1 (0.8 to 1.3)	1 (1 to 2)	0.1 (0.1 to 0.2)	39 (31 to 50)	3.4 (2.6 to 4.3)	1 (1 to 2)	0.1 (0.1 to 0.2)	41 (31 to 51)	3.5 (2.7 to 4.4)	7 (5 to 10)	0.3 (0.2 to 0.4)	47 (32 to 68)	1.7 (1.1 to 2.4)	4 (3 to 6)	0.2 (0.1 to 0.2)	113 (77 to 162)	3.9 (2.7 to 5.6)	5 (3 to 7)	0.2 (0.1 to 0.3)	117 (80 to 168)	4.1 (2.8 to 5.9)
Peru	Both	1398 (1182 to 1633)	10.4 (8.8 to 12.1)	10508 (8976 to 12149)	77.8 (67 to 89.4)	851 (729 to 984)	6.7 (5.7 to 7.7)	27344 (23192 to 31773)	195.2 (165.7 to 226.4)	787 (533 to 1110)	5.8 (3.9 to 8.1)	28131 (23828 to 32749)	201 (171.2 to 233.4)	4795 (3539 to 6383)	14.4 (10.7 to 19.2)	40273 (30474 to 52270)	121.4 (92.2 to 157.5)	1872 (1420 to 2441)	5.7 (4.3 to 7.4)	52837 (38615 to 69964)	157.8 (115.8 to 209)	2868 (1810 to 4243)	8.6 (5.4 to 12.8)	55704 (40920 to 73973)	166.5 (122.7 to 220.4)
	Female	1378 (1163 to 1611)	20.2 (17 to 23.5)	10399 (8879 to 12039)	151.6 (130.5 to 174.3)	836 (716 to 967)	12.8 (11 to 14.9)	26925 (22843 to 31289)	379 (322.1 to 440.5)	776 (525 to 1096)	11.2 (7.6 to 15.8)	27701 (23461 to 32208)	390.2 (331.3 to 453.6)	4743 (3502 to 6319)	27.6 (20.4 to 36.9)	39928 (30152 to 51853)	233.4 (177 to 302.6)	1840 (1391 to 2398)	10.8 (8.1 to 14.1)	52093 (38164 to 69066)	301.7 (221.1 to 399.3)	2835 (1786 to 4195)	16.5 (10.4 to 24.6)	54927 (40318 to 72923)	318.3 (234.5 to 421.2)
	Male	19 (15 to 25)	0.3 (0.2 to 0.4)	109 (83 to 140)	1.7 (1.3 to 2.2)	16 (12 to 20)	0.3 (0.2 to 0.4)	419 (323 to 541)	6.5 (5 to 8.5)	11 (7 to 16)	0.2 (0.1 to 0.3)	430 (332 to 556)	6.7 (5.2 to 8.7)	52 (34 to 78)	0.3 (0.2 to 0.5)	345 (229 to 512)	2.2 (1.5 to 3.2)	32 (20 to 47)	0.2 (0.1 to 0.3)	744 (490 to 1119)	4.7 (3.1 to 7)	33 (19 to 54)	0.2 (0.1 to 0.3)	777 (507 to 1171)	4.9 (3.2 to 7.4)
Philippines	Both	6434 (5567 to 7312)	18.3 (16 to 20.8)	50171 (44073 to 56754)	139.5 (122.6 to 157.5)	4006 (3508 to 4518)	12.8 (11.1 to 14.3)	130677 (114304 to 147748)	353.3 (309.4 to 398.9)	3904 (2724 to 5344)	10.8 (7.6 to 14.6)	134581 (118028 to 151679)	364.1 (319.1 to 409.1)	19247 (14387 to 25103)	21.3 (16.1 to 27.6)	154998 (119738 to 196987)	170.3 (133.6 to 214.3)	10255 (7741 to 13310)	12.3 (9.4 to 15.8)	331827 (247322 to 435843)	356.2 (266.9 to 465.1)	12025 (7773 to 17618)	13.1 (8.6 to 18.9)	343852 (257829 to 449078)	369.3 (279.1 to 479.3)

		1990												2019											
		Incidence		Prevalence		Deaths		YLLs (Years of Life Lost)		YLDs (Years Lived with Disability)		DALYs (Disability-Adjusted Life Years)		Incidence		Prevalence		Deaths		YLLs (Years of Life Lost)		YLDs (Years Lived with Disability)		DALYs (Disability-Adjusted Life Years)	
Location	Sex	Number	Rate	Number	Rate	Number	Rate	Number	Rate	Number	Rate	Number	Rate	Number	Rate	Number	Rate	Number	Rate	Number	Rate	Number	Rate	Number	Rate
	Female	6372 (5498 to 7250)	35.4 (30.8 to 40.2)	49755 (43711 to 56335)	270.4 (237.1 to 306.1)	3961 (3465 to 4470)	24.5 (21.4 to 27.4)	129342 (112870 to 146408)	685.8 (599.4 to 775)	3863 (2694 to 5288)	20.8 (14.6 to 28.3)	133205 (116653 to 150296)	706.6 (617.9 to 794.6)	19032 (14207 to 24890)	40.6 (30.5 to 53)	153564 (118321 to 195677)	326.5 (254.3 to 413.1)	10110 (7604 to 13135)	22.8 (17.4 to 29.5)	327666 (243031 to 430896)	686.6 (511.4 to 899.1)	11879 (7654 to 17379)	25.1 (16.3 to 36.4)	339545 (253445 to 443335)	711.7 (534.1 to 924.4)
	Male	61 (52 to 72)	0.4 (0.3 to 0.5)	417 (354 to 490)	2.5 (2.2 to 3)	45 (39 to 52)	0.3 (0.3 to 0.4)	1335 (1156 to 1533)	7.9 (6.8 to 9)	41 (28 to 56)	0.3 (0.2 to 0.4)	1375 (1197 to 1570)	8.2 (7.1 to 9.3)	216 (163 to 283)	0.6 (0.4 to 0.7)	1434 (1093 to 1859)	3.6 (2.8 to 4.6)	145 (110 to 190)	0.4 (0.3 to 0.5)	4161 (3136 to 5455)	10.1 (7.7 to 13.2)	146 (93 to 218)	0.4 (0.2 to 0.6)	4307 (3277 to 5616)	10.5 (8 to 13.7)
Poland	Both	9667 (9265 to 10093)	22.7 (21.7 to 23.8)	89589 (81825 to 98633)	209.1 (191.4 to 230.1)	5295 (5100 to 5438)	12.6 (12 to 12.9)	143006 (139315 to 146707)	334.6 (325.7 to 343.2)	6343 (4512 to 8529)	14.8 (10.5 to 19.9)	149350 (145274 to 153729)	349.4 (339.7 to 359.5)	18756 (14673 to 23572)	30.1 (23.6 to 38.3)	178260 (147165 to 216703)	283.5 (231.8 to 347)	7572 (6063 to 9372)	11.1 (8.9 to 13.8)	166094 (132296 to 208995)	265.2 (210.2 to 335.4)	12661 (8386 to 18102)	20.4 (13.4 to 29.2)	178755 (144376 to 222351)	285.6 (229.8 to 356.7)
	Female	9597 (9194 to 10022)	40.9 (39 to 42.8)	88991 (81283 to 97888)	374.2 (344.2 to 408.4)	5258 (5065 to 5401)	21.6 (20.8 to 22.2)	142078 (138405 to 145759)	608.3 (593 to 623.9)	6289 (4473 to 8447)	26.7 (18.9 to 35.9)	148367 (144301 to 152750)	635.1 (618.3 to 653.5)	18544 (14430 to 23330)	55.5 (43.2 to 70.9)	176495 (145516 to 214993)	523.1 (426.2 to 643.8)	7491 (5993 to 9292)	19.2 (15.3 to 24)	164318 (130565 to 207131)	487.6 (384.3 to 620.2)	12497 (8285 to 17879)	37.7 (24.7 to 54.1)	176815 (142437 to 220291)	525.3 (419.9 to 658.1)
	Male	69 (61 to 79)	0.4 (0.3 to 0.4)	598 (509 to 697)	3.3 (2.8 to 3.9)	37 (35 to 40)	0.2 (0.2 to 0.2)	929 (881 to 983)	5 (4.8 to 5.3)	54 (37 to 76)	0.3 (0.2 to 0.4)	983 (927 to 1041)	5.3 (5 to 5.6)	213 (158 to 281)	0.7 (0.6 to 1)	1765 (1351 to 2277)	6.2 (4.8 to 7.8)	81 (63 to 102)	0.3 (0.2 to 0.4)	1776 (1371 to 2261)	6.2 (4.8 to 7.9)	164 (100 to 239)	0.6 (0.4 to 0.8)	1940 (1515 to 2440)	6.8 (5.3 to 8.5)
Portugal	Both	4421 (4152 to 4696)	35.5 (33.4 to 37.7)	49575 (44368 to 55085)	385.4 (347.8 to 424.3)	1818 (1736 to 1899)	14.3 (13.7 to 15)	47492 (45719 to 49314)	383 (368.3 to 397.8)	3358 (2329 to 4610)	26.6 (18.4 to 36.5)	50850 (48684 to 53014)	409.6 (392.6 to 426.6)	7505 (5793 to 9736)	39.7 (30.3 to 52)	86203 (72465 to 104364)	441.8 (365 to 544)	2145 (1918 to 2358)	9.3 (8.5 to 10.2)	43660 (40040 to 47443)	231.2 (213.3 to 251.5)	5814 (3829 to 8442)	30.6 (19.7 to 45.1)	49474 (45014 to 54631)	261.8 (238.9 to 290)
	Female	4380 (4113 to 4652)	64.5 (60.7 to 68.7)	49229 (44030 to 54727)	696.9 (630.5 to 766.6)	1796 (1713 to 1876)	24.9 (23.8 to 26)	46985 (45196 to 48804)	701 (673.8 to 728.5)	3326 (2302 to 4570)	48.3 (33.4 to 66.5)	50311 (48164 to 52446)	749.3 (718 to 781)	7438 (5736 to 9649)	73.3 (55.8 to 96.1)	85566 (71981 to 103441)	814.3 (670.8 to 1006.1)	2120 (1894 to 2336)	16.4 (15 to 17.9)	43209 (39635 to 47007)	425.9 (392.8 to 463.2)	5757 (3790 to 8368)	56.4 (36.2 to 83.4)	48966 (44556 to 54122)	482.3 (439.7 to 535.7)
	Male	41 (35 to 49)	0.7 (0.6 to 0.9)	345 (294 to 410)	6 (5.1 to 7)	23 (20 to 26)	0.4 (0.4 to 0.5)	506 (441 to 577)	8.9 (7.8 to 10)	32 (22 to 46)	0.6 (0.4 to 0.8)	539 (468 to 612)	9.4 (8.2 to 10.6)	67 (47 to 94)	0.7 (0.5 to 1)	637 (478 to 829)	6.6 (4.9 to 8.5)	25 (20 to 31)	0.2 (0.2 to 0.3)	451 (373 to 544)	4.9 (4.1 to 5.8)	57 (35 to 90)	0.6 (0.4 to 0.9)	508 (418 to 620)	5.5 (4.5 to 6.6)
Puerto Rico	Both	1029 (952 to 1110)	28.8 (26.7 to 31.1)	8969 (8258 to 9660)	250.4 (230.9 to 269.9)	370 (348 to 392)	10.5 (9.8 to 11.1)	10634 (10052 to 11264)	298.7 (282.1 to 316.6)	634 (446 to 862)	17.7 (12.4 to 24.1)	11268 (10630 to 11968)	316.4 (298.7 to 336.1)	2222 (1709 to 2826)	38.7 (29.5 to 50.2)	20137 (16059 to 25001)	349.9 (276.6 to 442.5)	626 (491 to 787)	9.5 (7.4 to 12.2)	14367 (11097 to 18401)	259 (196.4 to 335.1)	1384 (921 to 1976)	24.2 (16.2 to 35)	15750 (12189 to 20164)	283.2 (215.6 to 364.9)
	Female	1014 (939 to 1095)	53.1 (49.2 to 57.3)	8875 (8166 to 9561)	463.4 (426.7 to 499.9)	361 (340 to 383)	18.9 (17.8 to 20.1)	10433 (9855 to 11075)	548.6 (517.8 to 582.4)	624 (440 to 851)	32.6 (22.9 to 44.6)	11057 (10422 to 11756)	581.3 (547.9 to 617.5)	2189 (1681 to 2788)	70.8 (53.9 to 91.9)	19895 (15871 to 24735)	641.2 (504.9 to 812)	610 (478 to 767)	16.9 (13.1 to 21.6)	14037 (10842 to 17911)	471.5 (356.6 to 611)	1361 (908 to 1950)	44.2 (29.5 to 63.9)	15398 (11913 to 19743)	515.6 (391.8 to 665.9)
	Male	14 (12 to 17)	0.9 (0.7 to 1)	94 (77 to 113)	5.6 (4.6 to 6.7)	9 (7 to 10)	0.5 (0.5 to 0.6)	202 (172 to 234)	12.2 (10.4 to 14)	9 (6 to 13)	0.6 (0.4 to 0.8)	211 (181 to 244)	12.7 (10.9 to 14.7)	34 (23 to 48)	1.1 (0.8 to 1.6)	242 (169 to 343)	8.4 (5.9 to 12)	16 (12 to 22)	0.5 (0.4 to 0.7)	329 (234 to 454)	11.7 (8.3 to 16)	22 (14 to 35)	0.8 (0.5 to 1.2)	352 (252 to 483)	12.5 (8.9 to 17.2)
Qatar	Both	30 (24 to 39)	16.8 (12.6 to 22.5)	260 (208 to 322)	142.3 (116.4 to 175.2)	14 (11 to 18)	10.8 (7.9 to 14.7)	517 (401 to 660)	247.7 (187.1 to 328.8)	20 (13 to 29)	10.4 (6.9 to 14.9)	537 (417 to 684)	258.2 (195.6 to 341.4)	388 (287 to 509)	25.5 (19.8 to 32.1)	3468 (2633 to 4489)	216.8 (172.6 to 269.5)	88 (66 to 114)	9 (7.1 to 11.2)	3139 (2344 to 4107)	193.4 (150.1 to 243.2)	260 (164 to 388)	16.3 (10.7 to 23.1)	3399 (2551 to 4461)	209.7 (162.5 to 262.5)
	Female	30 (23 to 38)	48.9 (37 to 64.6)	255 (202 to 317)	420.4 (341.4 to 522)	14 (11 to 17)	28.2 (20.9 to 38)	503 (388 to 645)	766.6 (582.2 to 1005.6)	19 (13 to 28)	30.8 (20.2 to 44.2)	522 (405 to 667)	797.4 (605.2 to 1045.6)	382 (283 to 501)	103.7 (80.2 to 131.2)	3410 (2589 to 4419)	880.3 (701.3 to 1098.3)	86 (64 to 111)	36.9 (28.9 to 45.8)	3070 (2292 to 4009)	790.6 (610.1 to 996.8)	255 (160 to 379)	65.8 (43.5 to 92.9)	3325 (2493 to 4364)	856.4 (662.5 to 1074.6)
	Male	1 (0 to 1)	0.8 (0.5 to 1.2)	6 (4 to 8)	5.5 (3.7 to 7.9)	0 (0 to 1)	0.5 (0.3 to 0.8)	14 (8 to 21)	12.5 (7.6 to 18.5)	0 (0 to 1)	0.5 (0.3 to 0.8)	14 (8 to 21)	13 (8 to 19.3)	6 (4 to 10)	0.9 (0.5 to 1.3)	59 (39 to 85)	7.2 (4.9 to 10.2)	2 (1 to 3)	0.4 (0.3 to 0.6)	69 (42 to 105)	7.7 (4.8 to 11.2)	5 (3 to 8)	0.6 (0.3 to 1.1)	74 (44 to 112)	8.4 (5.2 to 12)
Republic of Korea	Both	3460 (3239 to 3716)	9.5 (8.9 to 10.2)	44949 (39714 to 51208)	135.3 (117.8 to 156.1)	1429 (1353 to 1512)	4.3 (4.1 to 4.6)	48680 (46135 to 51532)	128.3 (121.6 to 135.7)	2898 (1939 to 4027)	8.3 (5.5 to 11.6)	51578 (48815 to 54682)	136.5 (129.1 to 144.8)	20328 (16423 to 24533)	23.8 (19.3 to 28.7)	218097 (184776 to 253670)	252.3 (214.7 to 294.3)	4090 (3603 to 4578)	4.7 (4.1 to 5.2)	107403 (95353 to 119372)	125.4 (111.6 to 138.9)	15556 (10234 to 22523)	18.2 (12 to 26.3)	122960 (108643 to 138700)	143.6 (127 to 161.6)

		1990												2019											
		Incidence		Prevalence		Deaths		YLLs (Years of Life Lost)		YLDs (Years Lived with Disability)		DALYs (Disability-Adjusted Life Years)		Incidence		Prevalence		Deaths		YLLs (Years of Life Lost)		YLDs (Years Lived with Disability)		DALYs (Disability-Adjusted Life Years)	
Location	Sex	Number	Rate	Number	Rate	Number	Rate	Number	Rate	Number	Rate	Number	Rate	Number	Rate	Number	Rate	Number	Rate	Number	Rate	Number	Rate	Number	Rate
	Female	3444 (3223 to 3702)	17.5 (16.4 to 18.8)	44796 (39551 to 51041)	239.7 (209.7 to 275.4)	1419 (1344 to 1502)	7.6 (7.2 to 8.1)	48393 (45835 to 51259)	240.2 (227.9 to 254)	2884 (1929 to 4007)	14.9 (9.9 to 20.8)	51278 (48509 to 54396)	255.1 (241.1 to 270.7)	20251 (16357 to 24449)	46.4 (37.6 to 56.1)	217371 (184216 to 252723)	488.5 (414.5 to 571.8)	4063 (3582 to 4548)	8.7 (7.7 to 9.7)	106795 (94795 to 118833)	245.9 (218.9 to 272.7)	15490 (10177 to 22417)	35.3 (23.1 to 51.3)	122285 (107936 to 138057)	281.2 (247.8 to 316.6)
	Male	16 (13 to 19)	0.1 (0.1 to 0.2)	153 (126 to 187)	1.1 (0.9 to 1.4)	9 (8 to 11)	0.1 (0.1 to 0.1)	287 (247 to 336)	1.9 (1.7 to 2.3)	13 (9 to 18)	0.1 (0.1 to 0.1)	300 (259 to 349)	2 (1.8 to 2.4)	77 (55 to 105)	0.2 (0.2 to 0.3)	725 (550 to 961)	1.9 (1.5 to 2.4)	28 (21 to 35)	0.1 (0.1 to 0.1)	608 (467 to 766)	1.6 (1.3 to 2)	66 (42 to 105)	0.2 (0.1 to 0.3)	674 (520 to 857)	1.8 (1.4 to 2.2)
Republic of Moldova	Both	1074 (1007 to 1146)	23.5 (22.1 to 25.1)	9900 (9021 to 10830)	218.5 (198.3 to 239.6)	529 (501 to 558)	11.8 (11.2 to 12.4)	16765 (15907 to 17670)	364 (345.5 to 383.8)	717 (502 to 967)	15.7 (11 to 21.2)	17482 (16564 to 18463)	379.7 (360 to 400.8)	1245 (1052 to 1469)	22.2 (18.9 to 26.5)	12000 (10514 to 13784)	213.4 (187.5 to 246.8)	512 (437 to 602)	9 (7.6 to 10.5)	13554 (11539 to 16039)	244.2 (207.7 to 288.9)	854 (575 to 1193)	15.3 (10.4 to 21.4)	14408 (12300 to 17075)	259.4 (220.8 to 307.8)
	Female	1071 (1004 to 1143)	41.8 (39.2 to 44.6)	9878 (8995 to 10805)	383.8 (348.6 to 419.4)	528 (500 to 556)	20.5 (19.4 to 21.6)	16721 (15864 to 17623)	653.3 (619.8 to 688.4)	715 (501 to 964)	27.8 (19.5 to 37.5)	17436 (16518 to 18415)	681.2 (644.5 to 719.3)	1226 (1036 to 1448)	39.2 (33.2 to 46.8)	11868 (10391 to 13651)	374.4 (327.6 to 434.3)	504 (430 to 593)	15.3 (13 to 18)	13328 (11350 to 15777)	433.1 (367.8 to 513.3)	841 (564 to 1173)	26.8 (18.1 to 37.5)	14169 (12088 to 16799)	459.9 (391 to 546.7)
	Male	3 (2 to 3)	0.1 (0.1 to 0.2)	22 (19 to 26)	1.2 (1 to 1.4)	2 (1 to 2)	0.1 (0.1 to 0.1)	44 (39 to 49)	2.3 (2 to 2.5)	2 (1 to 3)	0.1 (0.1 to 0.2)	46 (41 to 51)	2.4 (2.1 to 2.6)	19 (14 to 24)	0.8 (0.6 to 1)	132 (102 to 168)	5.4 (4.2 to 6.8)	8 (7 to 10)	0.4 (0.3 to 0.4)	226 (181 to 272)	9.2 (7.4 to 11.1)	13 (9 to 20)	0.6 (0.4 to 0.8)	239 (192 to 289)	9.8 (7.9 to 11.8)
Romania	Both	5009 (4753 to 5298)	18.3 (17.3 to 19.3)	49190 (44566 to 54358)	178.7 (162.6 to 197.4)	2619 (2508 to 2740)	9.6 (9.2 to 10)	77597 (74409 to 81268)	282.3 (270.7 to 295.3)	3425 (2366 to 4707)	12.5 (8.6 to 17.1)	81022 (77540 to 85082)	294.7 (282.6 to 309.1)	8751 (7089 to 10635)	27.3 (22.1 to 33.4)	82199 (68867 to 96308)	254.4 (211.8 to 301.1)	3661 (3007 to 4417)	10.4 (8.5 to 12.6)	86207 (69836 to 105031)	270.6 (218.2 to 331.9)	5918 (4016 to 8266)	18.5 (12.4 to 26.3)	92126 (74533 to 112708)	289.1 (233.2 to 355.4)
	Female	4947 (4685 to 5243)	34.1 (32.4 to 36.2)	48680 (44091 to 53808)	331.1 (302.2 to 364.3)	2584 (2475 to 2704)	17.4 (16.7 to 18.3)	76707 (73475 to 80286)	531.5 (509.3 to 556.3)	3378 (2333 to 4640)	23.2 (16.1 to 32)	80085 (76593 to 83994)	554.8 (531.4 to 581.6)	8592 (6966 to 10464)	50.9 (41.1 to 62.5)	80939 (67860 to 94937)	473.4 (394.3 to 562.7)	3595 (2951 to 4326)	18.5 (15.1 to 22.6)	84701 (68612 to 103207)	504.4 (406.2 to 622)	5798 (3929 to 8101)	34.5 (23 to 49.1)	90499 (73158 to 110758)	538.9 (432.9 to 665.4)
	Male	62 (52 to 72)	0.5 (0.4 to 0.6)	511 (434 to 595)	4.1 (3.5 to 4.7)	35 (30 to 39)	0.3 (0.3 to 0.3)	890 (780 to 1022)	6.9 (6.1 to 7.8)	47 (32 to 65)	0.4 (0.3 to 0.5)	937 (823 to 1076)	7.3 (6.4 to 8.3)	160 (120 to 208)	1 (0.8 to 1.4)	1260 (963 to 1620)	8.3 (6.4 to 10.7)	66 (51 to 83)	0.4 (0.3 to 0.5)	1506 (1167 to 1875)	10 (7.8 to 12.5)	120 (74 to 178)	0.8 (0.5 to 1.2)	1626 (1252 to 2026)	10.8 (8.3 to 13.4)
Russian Federation	Both	37484 (36515 to 39077)	21.2 (20.6 to 22.1)	382312 (351083 to 422264)	216.1 (198.9 to 238.5)	16692 (16236 to 17297)	9.5 (9.2 to 9.8)	481894 (469381 to 501524)	270.6 (263.3 to 281.9)	26160 (18379 to 35287)	14.8 (10.4 to 20)	508054 (493379 to 530549)	285.4 (276.8 to 298.5)	68411 (55984 to 83181)	31.3 (25.4 to 38.3)	668142 (564736 to 785557)	301.8 (253.9 to 357.2)	23778 (19601 to 28319)	10.4 (8.6 to 12.4)	596647 (483946 to 723409)	272 (219.8 to 330.8)	47346 (31430 to 68619)	21.6 (14.3 to 31.4)	643993 (535844 to 773926)	293.6 (243.3 to 354.3)
	Female	36949 (35973 to 38510)	36.1 (35.1 to 37.7)	378238 (347047 to 417949)	359.4 (334.1 to 390.7)	16422 (15960 to 17015)	15.1 (14.7 to 15.7)	474529 (462130 to 494032)	463.8 (451.6 to 484.1)	25765 (18081 to 34761)	24.9 (17.6 to 33.7)	500294 (485472 to 522525)	488.7 (473.8 to 512.3)	67693 (55292 to 82510)	54.4 (44 to 66.9)	662018 (559054 to 779062)	521.7 (436.9 to 620.7)	23486 (19329 to 27969)	17 (13.9 to 20.5)	589540 (476530 to 715933)	471.8 (379.7 to 577)	46779 (30993 to 67755)	37.5 (24.8 to 54.8)	636320 (527775 to 766309)	509.3 (419.5 to 618.3)
	Male	535 (491 to 570)	0.8 (0.8 to 0.9)	4074 (3744 to 4395)	6.1 (5.6 to 6.6)	270 (247 to 286)	0.5 (0.4 to 0.5)	7364 (6758 to 7805)	10.9 (10 to 11.5)	395 (284 to 529)	0.6 (0.4 to 0.8)	7760 (7122 to 8231)	11.5 (10.5 to 12.1)	718 (586 to 866)	0.8 (0.7 to 1)	6124 (5152 to 7198)	6.9 (5.8 to 8.1)	292 (240 to 353)	0.3 (0.3 to 0.4)	7107 (5780 to 8648)	7.9 (6.5 to 9.6)	566 (377 to 806)	0.6 (0.4 to 0.9)	7673 (6318 to 9250)	8.6 (7.1 to 10.3)
Rwanda	Both	495 (375 to 640)	15.5 (12 to 19.5)	2904 (2242 to 3700)	87.1 (69.3 to 109)	405 (310 to 517)	13.5 (10.7 to 16.9)	13332 (9757 to 17480)	382.9 (290.7 to 494.3)	266 (169 to 375)	8.1 (5.2 to 11.2)	13598 (9990 to 17829)	390.9 (298.1 to 503.9)	1118 (816 to 1500)	16.8 (12.8 to 21.9)	7699 (5794 to 10166)	109 (85.2 to 140.4)	760 (572 to 998)	12.8 (9.9 to 16.3)	23355 (16893 to 32215)	320.3 (238.4 to 424.8)	643 (393 to 978)	9.2 (5.8 to 13.5)	23999 (17398 to 32941)	329.5 (245.8 to 436.3)
	Female	453 (339 to 599)	25.1 (18.8 to 32.6)	2727 (2077 to 3515)	146.9 (114.8 to 186.2)	365 (273 to 483)	21.4 (16.2 to 27.6)	12373 (9027 to 16531)	638.4 (473 to 848.1)	245 (154 to 354)	13.2 (8.5 to 18.8)	12618 (9242 to 16855)	651.7 (481.6 to 864.8)	1058 (774 to 1426)	27.3 (20.5 to 36)	7376 (5563 to 9771)	182.5 (141.7 to 236.3)	710 (531 to 941)	20 (15.4 to 25.6)	22153 (16022 to 30494)	532.8 (393.8 to 715.3)	610 (371 to 929)	15.2 (9.5 to 22.6)	22763 (16390 to 31284)	547.9 (405 to 733.6)
	Male	42 (24 to 63)	3.7 (2.2 to 5.6)	177 (109 to 266)	14.4 (9 to 21.1)	40 (23 to 60)	3.8 (2.3 to 5.8)	959 (553 to 1453)	76.1 (45 to 114.2)	21 (11 to 35)	1.8 (0.9 to 2.9)	980 (564 to 1490)	77.9 (46.5 to 116.8)	60 (36 to 94)	2.9 (1.7 to 4.4)	323 (201 to 504)	13.7 (8.9 to 20.5)	50 (31 to 80)	2.7 (1.6 to 4.1)	1202 (712 to 1926)	49.4 (30.2 to 76.9)	34 (18 to 59)	1.5 (0.8 to 2.5)	1236 (736 to 1973)	50.9 (31.4 to 79.1)
Saint Kitts and Nevis	Both	18 (16 to 20)	54.6 (47.9 to 61.2)	139 (122 to 155)	414.3 (366.7 to 463.4)	9 (8 to 10)	25.7 (22.9 to 28.3)	229 (204 to 255)	700.2 (619.7 to 780.4)	11 (8 to 14)	31.7 (22.3 to 41.5)	240 (213 to 268)	731.9 (648.2 to 816)	31 (23 to 40)	44.3 (32.8 to 56.9)	252 (189 to 324)	348.9 (263.7 to 443.9)	11 (8 to 13)	16.7 (13.4 to 20.8)	286 (204 to 373)	396.6 (287.3 to 518.1)	19 (12 to 28)	26.8 (17.1 to 38.4)	305 (218 to 400)	423.3 (306.5 to 550.7)

		1990												2019											
		Incidence		Prevalence		Deaths		YLLs (Years of Life Lost)		YLDs (Years Lived with Disability)		DALYs (Disability-Adjusted Life Years)		Incidence		Prevalence		Deaths		YLLs (Years of Life Lost)		YLDs (Years Lived with Disability)		DALYs (Disability-Adjusted Life Years)	
Location	Sex	Number	Rate	Number	Rate	Number	Rate	Number	Rate	Number	Rate	Number	Rate	Number	Rate	Number	Rate	Number	Rate	Number	Rate	Number	Rate	Number	Rate
	Female	18 (16 to 20)	98.8 (86.4 to 111)	137 (120 to 153)	758.5 (670.1 to 850.3)	8 (8 to 9)	44.7 (39.8 to 49.5)	223 (198 to 249)	1273.9 (1121.2 to 1425.3)	10 (7 to 14)	57.6 (40.4 to 75.4)	234 (207 to 261)	1331.5 (1173.6 to 1489.9)	30 (22 to 40)	84.1 (61.5 to 108.9)	247 (185 to 318)	673.1 (504.5 to 861.6)	10 (8 to 13)	30 (23.6 to 37.5)	276 (196 to 362)	751.7 (534.8 to 985.8)	19 (12 to 27)	51 (32.4 to 73.9)	295 (211 to 387)	802.8 (571.1 to 1054.3)
	Male	0 (0 to 0)	2.4 (2.1 to 2.7)	2 (2 to 2)	13 (11 to 15.3)	0 (0 to 0)	1.8 (1.6 to 2.1)	6 (5 to 6)	37.3 (32.5 to 42.6)	0 (0 to 0)	1.3 (0.9 to 1.9)	6 (5 to 7)	38.6 (33.7 to 44.3)	1 (1 to 1)	2.4 (1.9 to 2.9)	5 (4 to 6)	14 (10.9 to 17.7)	0 (0 to 0)	1.4 (1.2 to 1.7)	10 (7 to 12)	28.9 (23 to 35.2)	0 (0 to 1)	1.4 (0.9 to 2.1)	10 (8 to 13)	30.3 (24 to 37.2)
Saint Lucia	Both	26 (23 to 29)	29.7 (26.6 to 32.8)	201 (181 to 223)	227.4 (204 to 252.4)	13 (12 to 15)	16.1 (14.7 to 17.7)	385 (349 to 424)	436.9 (395 to 482)	15 (11 to 20)	16.9 (12 to 22.8)	400 (362 to 441)	453.8 (409.7 to 501.1)	64 (53 to 77)	29.2 (24.4 to 35)	524 (440 to 624)	237.9 (201.2 to 282.4)	26 (21 to 30)	12 (10.1 to 14.1)	702 (581 to 842)	318.6 (264.7 to 380.9)	38 (26 to 52)	17.2 (11.9 to 23.7)	739 (612 to 884)	335.8 (278.8 to 400.9)
	Female	25 (23 to 28)	53.2 (47.6 to 59)	198 (178 to 220)	413.3 (371.3 to 459.9)	13 (12 to 14)	27.8 (25.2 to 30.6)	377 (341 to 415)	791.7 (711.1 to 873.7)	15 (10 to 20)	30.5 (21.5 to 41.3)	392 (353 to 432)	822.2 (740 to 909.9)	63 (52 to 75)	55.3 (46.2 to 66.4)	516 (433 to 615)	455.8 (384.5 to 542.5)	25 (21 to 29)	21.7 (18.2 to 25.7)	684 (567 to 822)	604.7 (501 to 725.6)	37 (25 to 51)	32.7 (22.4 to 45.4)	721 (596 to 862)	637.4 (527.7 to 764.3)
	Male	0 (0 to 1)	1.3 (1.2 to 1.5)	3 (2 to 3)	7.1 (6.2 to 8.1)	0 (0 to 0)	1.1 (1 to 1.3)	8 (7 to 9)	22 (19.6 to 24.7)	0 (0 to 0)	0.7 (0.5 to 1)	9 (8 to 10)	22.7 (20.2 to 25.5)	1 (1 to 1)	1.2 (1 to 1.5)	8 (6 to 10)	7.8 (6.3 to 9.7)	1 (1 to 1)	0.9 (0.7 to 1)	17 (14 to 21)	17 (13.8 to 21)	1 (1 to 1)	0.7 (0.5 to 1.1)	18 (15 to 22)	17.7 (14.4 to 21.8)
Saint Vincent and the Grenadines	Both	22 (19 to 24)	30.9 (27.6 to 34.3)	169 (152 to 187)	238.3 (213.8 to 264.2)	11 (10 to 13)	16.6 (15.1 to 18.3)	312 (281 to 346)	442 (396.8 to 491.2)	13 (9 to 17)	17.7 (12.5 to 23.5)	325 (293 to 360)	459.7 (413.5 to 511.3)	45 (38 to 53)	33.7 (28.7 to 39.7)	358 (308 to 420)	264.3 (227.8 to 309.1)	20 (18 to 24)	15.6 (13.5 to 18)	567 (483 to 665)	422 (359.4 to 494.5)	26 (18 to 36)	19.6 (13.6 to 27)	593 (505 to 698)	441.6 (376.8 to 519.6)
	Female	21 (19 to 24)	56.5 (50.4 to 63)	168 (151 to 185)	441.5 (395.8 to 489.7)	11 (10 to 12)	29.3 (26.5 to 32.3)	308 (277 to 341)	820.5 (735.4 to 914)	12 (9 to 17)	32.6 (23.1 to 43.5)	320 (288 to 355)	853.1 (766.3 to 949.1)	45 (38 to 53)	68.1 (57.9 to 80.4)	354 (305 to 416)	538.7 (464.5 to 631.5)	20 (17 to 23)	30.7 (26.5 to 35.5)	557 (474 to 653)	852.3 (726.2 to 999.4)	26 (18 to 36)	39.6 (27.5 to 54.7)	583 (496 to 687)	891.9 (759.8 to 1050.9)
	Male	0 (0 to 0)	0.9 (0.7 to 1)	2 (1 to 2)	4.8 (4.1 to 5.5)	0 (0 to 0)	0.7 (0.6 to 0.8)	5 (4 to 5)	14.2 (12.6 to 16.1)	0 (0 to 0)	0.5 (0.3 to 0.7)	5 (4 to 5)	14.7 (13 to 16.6)	1 (1 to 1)	0.9 (0.8 to 1.1)	4 (3 to 5)	5.4 (4.5 to 6.6)	0 (0 to 1)	0.7 (0.6 to 0.8)	10 (8 to 12)	14.1 (11.7 to 16.8)	0 (0 to 1)	0.5 (0.4 to 0.8)	10 (8 to 12)	14.7 (12.2 to 17.4)
Samoa	Both	15 (11 to 20)	15.9 (11.9 to 21.5)	127 (100 to 163)	135.9 (108.9 to 173.2)	10 (7 to 13)	10.7 (8.2 to 14.2)	297 (220 to 404)	308.4 (230.2 to 417.5)	9 (6 to 14)	9.9 (6.4 to 14.4)	307 (227 to 417)	318.2 (237.5 to 429.4)	35 (23 to 57)	22 (14.6 to 35)	294 (209 to 439)	183.6 (132.6 to 269.9)	19 (13 to 30)	12.5 (8.5 to 19.7)	582 (378 to 928)	355.3 (235 to 565.3)	22 (13 to 37)	13.7 (8.2 to 22.9)	604 (395 to 960)	369 (243.8 to 585.3)
	Female	15 (11 to 20)	31.1 (23.1 to 42.5)	126 (99 to 162)	266.4 (213.1 to 341.8)	9 (7 to 13)	20.5 (15.5 to 27.4)	294 (217 to 401)	609.9 (452.5 to 827.9)	9 (6 to 14)	19.3 (12.5 to 28.5)	304 (224 to 414)	629.2 (465.7 to 852.5)	35 (23 to 56)	44 (29.2 to 70.4)	292 (207 to 436)	368.5 (263.4 to 545.9)	19 (13 to 30)	24.5 (16.6 to 38.8)	577 (372 to 923)	716.9 (468.9 to 1146)	22 (13 to 37)	27.4 (16.3 to 46)	599 (389 to 955)	744.3 (488.4 to 1185.4)
	Male	0 (0 to 0)	0.4 (0.2 to 0.5)	1 (1 to 1)	2.3 (1.7 to 3.1)	0 (0 to 0)	0.3 (0.2 to 0.4)	3 (2 to 4)	6.5 (4.3 to 9.3)	0 (0 to 0)	0.2 (0.1 to 0.4)	3 (2 to 4)	6.7 (4.5 to 9.6)	0 (0 to 0)	0.4 (0.3 to 0.6)	2 (1 to 3)	2.9 (2.1 to 4.1)	0 (0 to 0)	0.3 (0.2 to 0.5)	5 (4 to 8)	7.1 (4.7 to 10.5)	0 (0 to 0)	0.3 (0.2 to 0.5)	6 (4 to 8)	7.4 (4.9 to 11)
San Marino	Both	10 (8 to 12)	32.9 (27.5 to 39.7)	116 (99 to 136)	369.5 (317.1 to 428.9)	4 (3 to 4)	11.1 (9.2 to 13.4)	78 (64 to 95)	257.6 (209.5 to 314.3)	8 (5 to 11)	25.3 (16.9 to 35.9)	86 (72 to 105)	283 (232 to 345)	24 (18 to 33)	44.3 (32.7 to 61.2)	252 (203 to 325)	465 (368.7 to 606.6)	7 (4 to 10)	10.5 (6.8 to 15.8)	138 (88 to 210)	259.5 (160.9 to 406.8)	18 (11 to 26)	33.4 (20.9 to 50.1)	155 (101 to 235)	292.9 (189.4 to 452.9)
	Female	10 (8 to 12)	62.6 (52.3 to 75.6)	116 (99 to 135)	699.1 (597.1 to 813.2)	4 (3 to 4)	20.1 (16.6 to 24.3)	78 (64 to 95)	493.3 (398.3 to 602.4)	8 (5 to 11)	48.2 (32.1 to 68.3)	86 (71 to 104)	541.5 (442 to 659.7)	24 (18 to 32)	84.2 (61.8 to 116.4)	251 (202 to 324)	885.9 (701.9 to 1158.3)	7 (4 to 10)	19.5 (12.6 to 29.6)	137 (87 to 209)	493.1 (305.1 to 773.9)	18 (11 to 26)	63.5 (39.8 to 95.5)	155 (101 to 234)	556.6 (359.1 to 864.3)
	Male	0 (0 to 0)	0.3 (0.2 to 0.4)	0 (0 to 1)	3 (2.3 to 3.8)	0 (0 to 0)	0.1 (0.1 to 0.2)	0 (0 to 0)	2.4 (1.8 to 3.2)	0 (0 to 0)	0.3 (0.2 to 0.4)	0 (0 to 1)	2.7 (2 to 3.6)	0 (0 to 0)	0.4 (0.2 to 0.5)	1 (1 to 1)	3.6 (2.7 to 4.8)	0 (0 to 0)	0.1 (0.1 to 0.2)	1 (0 to 1)	2.3 (1.4 to 3.5)	0 (0 to 0)	0.3 (0.2 to 0.5)	1 (0 to 1)	2.6 (1.6 to 3.9)
Sao Tome and Principe	Both	5 (4 to 7)	8.3 (6.4 to 10.2)	39 (31 to 47)	60 (47.5 to 72.2)	4 (3 to 5)	6.6 (5.2 to 8.1)	123 (90 to 155)	184.1 (137 to 230.5)	3 (2 to 4)	4.7 (3 to 6.6)	126 (92 to 159)	188.8 (139.9 to 235.5)	21 (13 to 31)	16.5 (10.4 to 24.3)	150 (101 to 219)	116.9 (79.4 to 166.6)	12 (8 to 18)	10.8 (6.8 to 15.9)	387 (244 to 577)	291.3 (183.4 to 432.2)	12 (7 to 20)	9.5 (5.4 to 15.3)	399 (251 to 595)	300.8 (189.9 to 446.2)
	Female	5 (4 to 7)	15.6 (11.9 to 19.3)	39 (30 to 47)	113.8 (89.8 to 136.6)	4 (3 to 5)	12.3 (9.7 to 15.2)	123 (90 to 155)	351 (260.3 to 440.4)	3 (2 to 4)	9 (5.6 to 12.5)	126 (92 to 158)	359.9 (266.4 to 449.7)	20 (13 to 31)	32.1 (20.2 to 47.7)	150 (100 to 219)	229.4 (155.2 to 329.1)	12 (8 to 18)	20.8 (13.1 to 30.7)	385 (243 to 576)	573.3 (360.5 to 852.3)	12 (7 to 20)	18.7 (10.5 to 30)	398 (250 to 594)	592 (372.7 to 880.2)

		1990												2019											
		Incidence		Prevalence		Deaths		YLLs (Years of Life Lost)		YLDs (Years Lived with Disability)		DALYs (Disability-Adjusted Life Years)		Incidence		Prevalence		Deaths		YLLs (Years of Life Lost)		YLDs (Years Lived with Disability)		DALYs (Disability-Adjusted Life Years)	
Location	Sex	Number	Rate	Number	Rate	Number	Rate	Number	Rate	Number	Rate	Number	Rate	Number	Rate	Number	Rate	Number	Rate	Number	Rate	Number	Rate	Number	Rate
	Male	0 (0 to 0)	0.1 (0 to 0.1)	0 (0 to 0)	0.3 (0.2 to 0.4)	0 (0 to 0)	0 (0 to 0.1)	0 (0 to 1)	1.2 (0.8 to 1.6)	0 (0 to 0)	0 (0 to 0)	0 (0 to 1)	1.2 (0.8 to 1.6)	0 (0 to 0)	0.1 (0.1 to 0.1)	0 (0 to 0)	0.5 (0.4 to 0.7)	0 (0 to 0)	0.1 (0 to 0.1)	1 (1 to 2)	1.7 (1.2 to 2.4)	0 (0 to 0)	0.1 (0 to 0.1)	1 (1 to 2)	1.7 (1.2 to 2.4)
Saudi Arabia	Both	475 (344 to 638)	6.1 (4.5 to 8.3)	4618 (3606 to 5835)	63.9 (51.4 to 78.3)	316 (225 to 432)	4.5 (3.3 to 6.2)	11074 (7848 to 15224)	130.7 (92.9 to 178.8)	327 (207 to 492)	4.2 (2.7 to 6.3)	11400 (8121 to 15558)	134.9 (97.2 to 185)	5371 (3870 to 7305)	17.2 (12.8 to 22.7)	47484 (35066 to 63347)	157 (122.8 to 200)	1428 (1049 to 1890)	5.8 (4.5 to 7.6)	52977 (38260 to 71206)	166.3 (123.5 to 218.5)	3603 (2267 to 5384)	11.5 (7.4 to 16.8)	56580 (40898 to 76008)	177.8 (132.7 to 235.1)
	Female	464 (331 to 626)	14.9 (10.8 to 20.1)	4529 (3522 to 5739)	154.1 (123 to 191)	307 (219 to 422)	10.7 (7.7 to 14.8)	10830 (7622 to 14931)	326.1 (230.7 to 447.4)	319 (201 to 481)	10.3 (6.6 to 15.4)	11149 (7894 to 15281)	336.4 (241.5 to 461.4)	5330 (3833 to 7262)	43.1 (31.9 to 57.1)	47120 (34765 to 62900)	394.2 (306.8 to 503.5)	1410 (1032 to 1868)	14.3 (10.9 to 18.7)	52462 (37864 to 70600)	417.4 (308.5 to 549.5)	3571 (2240 to 5339)	28.8 (18.4 to 42.2)	56033 (40441 to 75439)	446.1 (332.2 to 589.5)
	Male	11 (7 to 16)	0.3 (0.2 to 0.5)	89 (66 to 119)	2.4 (1.8 to 3.2)	9 (6 to 12)	0.3 (0.2 to 0.4)	243 (161 to 364)	6.1 (4 to 8.8)	8 (5 to 12)	0.2 (0.1 to 0.3)	251 (166 to 375)	6.3 (4.2 to 9.1)	41 (27 to 59)	0.4 (0.3 to 0.5)	364 (259 to 516)	3.2 (2.3 to 4.3)	17 (12 to 25)	0.2 (0.1 to 0.3)	515 (346 to 748)	4.1 (2.8 to 5.8)	32 (19 to 51)	0.3 (0.2 to 0.5)	547 (370 to 795)	4.4 (3 to 6.2)
Senegal	Both	374 (283 to 465)	10.3 (7.9 to 12.7)	2491 (1994 to 3011)	67.9 (54.8 to 81.4)	288 (221 to 354)	8.5 (6.6 to 10.3)	9391 (7155 to 11713)	242.7 (184.8 to 303.2)	210 (139 to 300)	5.7 (3.8 to 8.1)	9600 (7294 to 11991)	248.3 (189 to 309.3)	1281 (894 to 1678)	15.2 (10.9 to 19.9)	8919 (6602 to 11384)	103.2 (77.7 to 130.2)	875 (622 to 1146)	11.2 (8.1 to 14.6)	27319 (18838 to 36309)	305.7 (213.2 to 402.8)	734 (451 to 1105)	8.5 (5.4 to 12.5)	28053 (19361 to 37362)	314.2 (218.8 to 413.5)
	Female	372 (281 to 462)	20.4 (15.7 to 25.2)	2478 (1983 to 2998)	134.6 (108.7 to 161.3)	286 (219 to 352)	16.7 (13 to 20.4)	9334 (7104 to 11626)	478.6 (362.5 to 596.7)	208 (138 to 297)	11.2 (7.5 to 16)	9542 (7246 to 11940)	489.8 (371.8 to 609.5)	1275 (889 to 1671)	29.1 (20.8 to 38)	8883 (6567 to 11352)	198.2 (149.3 to 250.2)	870 (617 to 1141)	21.3 (15.4 to 27.8)	27179 (18738 to 36160)	586.4 (407.6 to 773.6)	730 (448 to 1099)	16.3 (10.2 to 24.1)	27909 (19258 to 37198)	602.6 (418.6 to 794.2)
	Male	2 (2 to 3)	0.1 (0.1 to 0.2)	13 (9 to 19)	0.7 (0.5 to 1)	2 (1 to 3)	0.1 (0.1 to 0.2)	57 (39 to 83)	3 (2.1 to 4.6)	1 (1 to 2)	0.1 (0 to 0.1)	58 (40 to 85)	3.1 (2.1 to 4.7)	6 (4 to 9)	0.2 (0.1 to 0.3)	37 (25 to 53)	0.9 (0.6 to 1.3)	5 (3 to 8)	0.1 (0.1 to 0.2)	139 (94 to 206)	3.4 (2.2 to 5)	4 (2 to 6)	0.1 (0.1 to 0.2)	143 (97 to 212)	3.5 (2.3 to 5.2)
Serbia	Both	3256 (2866 to 3720)	28.7 (25.4 to 32.5)	30903 (27241 to 34884)	269.8 (238.4 to 304)	1607 (1413 to 1834)	14.7 (12.9 to 16.7)	45724 (40495 to 51630)	399.4 (356.1 to 448.7)	2193 (1479 to 3018)	19.2 (13.2 to 26.5)	47917 (42372 to 54256)	418.7 (372.8 to 472)	6253 (4901 to 8029)	44.4 (34.6 to 57)	57381 (46903 to 70661)	404.4 (327.2 to 501.5)	2394 (1920 to 2978)	16 (12.8 to 19.9)	54466 (43076 to 68937)	389.4 (304.4 to 497.6)	4211 (2783 to 6069)	29.9 (19.4 to 43.3)	58677 (46385 to 74089)	419.3 (330.5 to 531.4)
	Female	3215 (2819 to 3678)	53.8 (47.5 to 60.9)	30583 (26906 to 34538)	504.7 (446.6 to 569.2)	1585 (1391 to 1811)	26.8 (23.6 to 30.5)	45170 (39957 to 51035)	753.4 (671.7 to 846)	2163 (1458 to 2975)	36 (24.7 to 49.4)	47333 (41840 to 53642)	789.4 (703.6 to 887.4)	6148 (4815 to 7880)	83.5 (64.8 to 107.2)	56568 (46219 to 69575)	762.3 (615 to 949.9)	2350 (1886 to 2926)	29.1 (23.2 to 36.2)	53545 (42325 to 67600)	731.7 (570.2 to 938)	4134 (2731 to 5980)	56.1 (36.3 to 81.7)	57679 (45709 to 72992)	787.8 (619.8 to 1006.3)
	Male	41 (28 to 55)	0.8 (0.6 to 1)	320 (242 to 419)	6 (4.6 to 7.8)	22 (16 to 30)	0.5 (0.3 to 0.6)	554 (397 to 737)	10.2 (7.3 to 13.3)	30 (19 to 46)	0.6 (0.4 to 0.9)	584 (417 to 781)	10.8 (7.8 to 14.1)	105 (67 to 154)	1.5 (1 to 2.2)	813 (556 to 1141)	11.7 (8.1 to 16.4)	44 (30 to 59)	0.6 (0.4 to 0.9)	922 (619 to 1293)	13.5 (9.2 to 18.6)	76 (45 to 120)	1.1 (0.7 to 1.7)	998 (670 to 1393)	14.6 (10 to 20.2)
Seychelles	Both	7 (7 to 9)	13.3 (11.8 to 15.2)	65 (58 to 74)	115.9 (102.5 to 132)	5 (4 to 5)	8.4 (7.4 to 9.4)	140 (124 to 160)	249.7 (220.4 to 285.5)	5 (3 to 7)	8.5 (5.9 to 11.7)	145 (128 to 166)	258.2 (227.9 to 294.8)	33 (27 to 39)	27.4 (22.5 to 32.5)	282 (238 to 329)	232.7 (197.2 to 269.9)	15 (12 to 18)	13.1 (10.9 to 15.4)	436 (359 to 526)	356.7 (294.5 to 427.9)	21 (14 to 30)	17.5 (11.9 to 24.4)	457 (376 to 553)	374.2 (309.6 to 448.5)
	Female	7 (7 to 8)	25.4 (22.4 to 29)	65 (57 to 74)	220.3 (194.8 to 250.6)	5 (4 to 5)	15.4 (13.7 to 17.4)	140 (123 to 160)	483.4 (425.2 to 553.2)	5 (3 to 7)	16.3 (11.3 to 22.8)	144 (128 to 165)	499.6 (440.1 to 571.4)	33 (27 to 39)	55.2 (45.3 to 65.7)	281 (237 to 328)	472.9 (399.2 to 551.5)	15 (12 to 17)	25 (20.7 to 29.7)	433 (356 to 524)	726 (599.8 to 874.6)	21 (14 to 30)	35.6 (24.1 to 50)	454 (373 to 549)	761.6 (627.2 to 915.7)
	Male	0 (0 to 0)	0.2 (0.1 to 0.2)	0 (0 to 0)	1 (0.8 to 1.3)	0 (0 to 0)	0.1 (0.1 to 0.2)	1 (1 to 1)	2.9 (2.2 to 3.8)	0 (0 to 0)	0.1 (0.1 to 0.1)	1 (1 to 1)	3 (2.3 to 4)	0 (0 to 0)	0.4 (0.3 to 0.5)	1 (1 to 2)	2.4 (1.8 to 3.1)	0 (0 to 0)	0.2 (0.2 to 0.3)	3 (2 to 4)	5.3 (4 to 7)	0 (0 to 0)	0.3 (0.2 to 0.4)	3 (2 to 4)	5.6 (4.2 to 7.3)
Sierra Leone	Both	148 (106 to 196)	7.3 (5.3 to 9.6)	1003 (763 to 1284)	49 (37.7 to 62.2)	119 (86 to 157)	6.1 (4.5 to 7.9)	3654 (2565 to 4901)	170.7 (121.7 to 227.8)	83 (52 to 124)	4 (2.5 to 5.9)	3737 (2628 to 5025)	174.8 (124.5 to 233)	502 (353 to 724)	12 (8.6 to 17.2)	3389 (2479 to 4695)	78.3 (58.3 to 107.8)	345 (247 to 497)	9 (6.6 to 12.9)	11178 (7727 to 16438)	247.7 (173.8 to 359.7)	290 (174 to 450)	6.7 (4.1 to 10.3)	11467 (7919 to 16837)	254.4 (178.8 to 368.5)
	Female	147 (105 to 194)	14.6 (10.6 to 19.2)	996 (756 to 1276)	99 (76.3 to 125.4)	118 (85 to 155)	12.3 (9 to 16)	3624 (2543 to 4861)	342.7 (244.3 to 458)	82 (51 to 123)	8.1 (5.1 to 12)	3706 (2603 to 4983)	350.8 (250 to 468.7)	499 (351 to 721)	24 (17.1 to 34.7)	3372 (2464 to 4676)	157.9 (117.3 to 218.3)	343 (244 to 494)	17.8 (12.9 to 25.7)	11112 (7667 to 16364)	499.1 (348.9 to 728.1)	288 (172 to 448)	13.5 (8.3 to 20.7)	11400 (7855 to 16755)	512.6 (358.5 to 743.4)
	Male	1 (1 to 2)	0.1 (0.1 to 0.2)	7 (5 to 10)	0.7 (0.4 to 0.9)	1 (1 to 2)	0.1 (0.1 to 0.2)	31 (20 to 45)	2.9 (1.9 to 4.3)	1 (0 to 1)	0.1 (0 to 0.1)	32 (21 to 46)	3 (2 to 4.4)	3 (2 to 4)	0.2 (0.1 to 0.2)	17 (12 to 24)	0.8 (0.5 to 1.1)	2 (2 to 3)	0.1 (0.1 to 0.2)	65 (43 to 96)	3.1 (2.1 to 4.7)	2 (1 to 3)	0.1 (0.1 to 0.1)	67 (45 to 98)	3.2 (2.1 to 4.8)
Singapore	Both	638 (588 to 694)	23.6 (21.8 to 25.6)	6795 (6181 to 7522)	262.7 (237.9 to 291.5)	205 (194 to 216)	8.4 (8 to 8.9)	6729 (6368 to 7109)	241.8 (229.3 to 254.8)	489 (335 to 678)	18.3 (12.6 to 25.4)	7217 (6789 to 7631)	260.1 (245.2 to 275.5)	2524 (1994 to 3142)	31 (24.5 to 38.5)	27184 (22591 to 32420)	335.1 (279.5 to 398.9)	515 (465 to 560)	6.5 (5.8 to 7.1)	13700 (12620 to 14887)	166.4 (153.2 to 180.6)	1937 (1274 to 2830)	23.8 (15.7 to 34.8)	15636 (14178 to 17350)	190.2 (172.2 to 211.1)

		1990												2019											
		Incidence		Prevalence		Deaths		YLLs (Years of Life Lost)		YLDs (Years Lived with Disability)		DALYs (Disability-Adjusted Life Years)		Incidence		Prevalence		Deaths		YLLs (Years of Life Lost)		YLDs (Years Lived with Disability)		DALYs (Disability-Adjusted Life Years)	
**Location**	**Sex**	**Number**	**Rate**	**Number**	**Rate**	**Number**	**Rate**	**Number**	**Rate**	**Number**	**Rate**	**Number**	**Rate**	**Number**	**Rate**	**Number**	**Rate**	**Number**	**Rate**	**Number**	**Rate**	**Number**	**Rate**	**Number**	**Rate**
	Female	636 (586 to 691)	44.7 (41.2 to 48.7)	6772 (6160 to 7500)	493.8 (447.3 to 548.6)	204 (193 to 215)	15.5 (14.6 to 16.4)	6695 (6337 to 7077)	460.2 (435.9 to 485.9)	487 (333 to 675)	34.5 (23.8 to 48)	7182 (6756 to 7594)	494.8 (465.9 to 523.9)	2516 (1986 to 3132)	62.7 (49.6 to 78.1)	27102 (22515 to 32327)	674.3 (558.3 to 807.1)	512 (462 to 557)	12.7 (11.4 to 13.8)	13637 (12562 to 14821)	338.7 (312.8 to 368)	1930 (1268 to 2819)	48.1 (31.6 to 70.6)	15567 (14118 to 17283)	386.9 (351.2 to 429.7)
	Male	2 (2 to 3)	0.2 (0.2 to 0.3)	23 (19 to 27)	2.1 (1.8 to 2.5)	1 (1 to 1)	0.1 (0.1 to 0.2)	33 (29 to 38)	3 (2.7 to 3.5)	2 (1 to 3)	0.2 (0.1 to 0.3)	35 (31 to 40)	3.2 (2.8 to 3.7)	8 (6 to 11)	0.2 (0.2 to 0.3)	82 (62 to 107)	2.3 (1.7 to 2.9)	3 (2 to 3)	0.1 (0.1 to 0.1)	63 (52 to 76)	1.7 (1.4 to 2)	7 (4 to 11)	0.2 (0.1 to 0.3)	70 (58 to 84)	1.9 (1.6 to 2.3)
Slovakia	Both	1467 (1369 to 1575)	25.3 (23.6 to 27.2)	14109 (12917 to 15591)	241.6 (221.3 to 266.4)	685 (643 to 728)	11.8 (11.1 to 12.5)	18922 (17742 to 20141)	327.1 (306.2 to 347.9)	1004 (709 to 1357)	17.2 (12.2 to 23.4)	19926 (18652 to 21261)	344.3 (321.6 to 367.3)	2901 (2161 to 3825)	33.4 (24.7 to 44.1)	27135 (21299 to 34222)	310.1 (241.8 to 392.5)	974 (733 to 1258)	10.9 (8.2 to 14)	22053 (16458 to 29161)	255.7 (190.7 to 338.3)	1977 (1311 to 2899)	22.7 (15.2 to 33.3)	24030 (17935 to 31778)	278.4 (207.6 to 368.5)
	Female	1451 (1353 to 1560)	45.5 (42.4 to 49.1)	13976 (12800 to 15446)	433.2 (396.8 to 474.9)	677 (635 to 720)	20.7 (19.4 to 22)	18730 (17548 to 19937)	596.9 (557.6 to 635.8)	991 (700 to 1342)	30.9 (21.7 to 42)	19721 (18444 to 21071)	627.8 (586.1 to 670.1)	2872 (2133 to 3785)	60.6 (44.4 to 80.3)	26885 (21114 to 33951)	564.1 (436.3 to 721.1)	963 (727 to 1246)	18.7 (14.1 to 24.4)	21814 (16274 to 28858)	468.7 (349.2 to 623.3)	1954 (1295 to 2874)	41.3 (27.2 to 60.4)	23769 (17741 to 31414)	509.9 (379.2 to 677.9)
	Male	17 (14 to 20)	0.7 (0.6 to 0.8)	133 (110 to 159)	5.3 (4.4 to 6.3)	8 (7 to 10)	0.4 (0.3 to 0.4)	193 (162 to 226)	7.6 (6.5 to 9)	13 (9 to 18)	0.5 (0.3 to 0.7)	205 (174 to 242)	8.1 (6.9 to 9.6)	29 (20 to 43)	0.8 (0.5 to 1.1)	250 (178 to 350)	6.6 (4.8 to 9.1)	11 (7 to 15)	0.3 (0.2 to 0.4)	238 (162 to 338)	6.3 (4.3 to 8.8)	23 (14 to 36)	0.6 (0.4 to 1)	261 (180 to 368)	6.9 (4.8 to 9.6)
Slovenia	Both	870 (644 to 1129)	36.4 (27.1 to 47.3)	8169 (6399 to 10293)	340.8 (266.4 to 429.8)	361 (277 to 463)	15 (11.6 to 19.3)	9204 (6904 to 12102)	384.3 (288.2 to 505.2)	583 (376 to 846)	24.4 (15.8 to 35.4)	9787 (7375 to 12831)	408.7 (307.7 to 537.4)	1233 (947 to 1638)	33.7 (25.7 to 45.1)	12643 (10341 to 15704)	340.3 (275.4 to 430)	438 (339 to 570)	10 (7.8 to 13.1)	8482 (6532 to 11227)	224.9 (172.6 to 300.4)	857 (550 to 1240)	23.5 (15.1 to 34.4)	9338 (7225 to 12238)	248.4 (191.7 to 328.1)
	Female	862 (638 to 1120)	65.4 (48.1 to 85.4)	8104 (6347 to 10218)	609.4 (472.9 to 775.3)	357 (274 to 459)	25.4 (19.4 to 32.9)	9127 (6844 to 12017)	690.9 (515.8 to 914.2)	577 (372 to 839)	43.9 (28.2 to 64.1)	9704 (7313 to 12731)	734.8 (549.5 to 974.9)	1217 (933 to 1619)	64.7 (49.4 to 86.8)	12504 (10230 to 15522)	653.2 (524.9 to 830.4)	433 (335 to 562)	17.7 (13.7 to 23.3)	8381 (6458 to 11106)	427.3 (326.9 to 573.1)	844 (543 to 1221)	45.2 (28.9 to 66.7)	9226 (7134 to 12098)	472.6 (363.6 to 627.6)
	Male	8 (6 to 11)	0.9 (0.6 to 1.1)	64 (49 to 84)	6.6 (5 to 8.5)	4 (3 to 5)	0.4 (0.3 to 0.6)	77 (58 to 103)	8.1 (6.1 to 10.7)	6 (4 to 9)	0.6 (0.4 to 0.9)	83 (62 to 111)	8.7 (6.5 to 11.5)	16 (11 to 22)	0.9 (0.6 to 1.2)	139 (101 to 185)	7.8 (5.7 to 10.2)	5 (4 to 7)	0.3 (0.2 to 0.4)	100 (70 to 137)	5.7 (4.1 to 7.8)	12 (8 to 19)	0.7 (0.4 to 1.1)	112 (80 to 152)	6.4 (4.6 to 8.6)
Solomon Islands	Both	18 (11 to 25)	10.2 (6.3 to 14.1)	146 (105 to 188)	88.6 (65.3 to 112.6)	12 (7 to 17)	7.4 (4.6 to 10.3)	433 (272 to 605)	238.3 (149.8 to 331.1)	11 (7 to 17)	6.4 (3.9 to 9.8)	444 (279 to 620)	244.7 (154.3 to 339)	281 (215 to 363)	62.6 (48.3 to 80.3)	2039 (1583 to 2626)	449.1 (355 to 567.8)	153 (117 to 196)	37.1 (29.2 to 46.8)	5910 (4483 to 7689)	1269.5 (976.3 to 1629.7)	169 (107 to 245)	36.9 (24.1 to 52.9)	6079 (4597 to 7916)	1306.5 (1004.8 to 1673.3)
	Female	17 (11 to 24)	22.1 (13.4 to 30.6)	145 (103 to 187)	194.5 (143.6 to 247.1)	12 (7 to 17)	16.3 (9.8 to 22.7)	427 (268 to 599)	512 (317.9 to 710.9)	11 (6 to 17)	14 (8.3 to 21.3)	438 (275 to 614)	526 (326 to 731.3)	280 (214 to 362)	126.5 (97.5 to 162.4)	2034 (1579 to 2619)	907.3 (717.2 to 1146.7)	152 (117 to 195)	75 (59 to 94.5)	5893 (4463 to 7672)	2561.3 (1967.3 to 3291.1)	168 (107 to 244)	74.5 (48.8 to 106.8)	6061 (4579 to 7893)	2635.7 (2026.6 to 3377.1)
	Male	0 (0 to 0)	0.3 (0.2 to 0.5)	1 (1 to 3)	1.9 (1.2 to 3)	0 (0 to 0)	0.3 (0.1 to 0.5)	6 (3 to 11)	6.4 (3.6 to 12)	0 (0 to 0)	0.2 (0.1 to 0.3)	6 (3 to 11)	6.6 (3.7 to 12.3)	1 (0 to 1)	0.5 (0.3 to 0.9)	5 (3 to 9)	2.9 (1.8 to 4.9)	1 (0 to 1)	0.4 (0.2 to 0.7)	17 (9 to 32)	9.3 (4.9 to 17)	1 (0 to 1)	0.3 (0.1 to 0.6)	18 (9 to 33)	9.6 (5 to 17.5)
Somalia	Both	257 (177 to 373)	8.4 (5.9 to 12.1)	1609 (1168 to 2213)	51.1 (39 to 67.5)	212 (147 to 305)	7.5 (5.3 to 10.7)	7329 (4956 to 10756)	208.8 (145.6 to 301.5)	142 (84 to 225)	4.5 (2.7 to 6.9)	7471 (5051 to 10940)	213.3 (149 to 307.5)	701 (428 to 1102)	9.3 (5.7 to 14.6)	4501 (3002 to 6688)	57.1 (40.2 to 82)	571 (355 to 889)	8.3 (5.2 to 12.9)	19022 (11923 to 29517)	219.6 (135.6 to 343.6)	390 (221 to 669)	5 (2.9 to 8.3)	19412 (12170 to 30091)	224.5 (138.8 to 351.5)
	Female	233 (154 to 341)	13.7 (9.3 to 20.3)	1494 (1079 to 2103)	87.7 (65.7 to 118.6)	188 (126 to 274)	12 (8.2 to 17.5)	6727 (4414 to 10106)	354 (237.8 to 518.6)	129 (76 to 208)	7.4 (4.4 to 11.7)	6856 (4519 to 10290)	361.5 (243 to 529.6)	651 (393 to 1033)	14.9 (9 to 23.9)	4244 (2796 to 6355)	94.5 (64.4 to 138.3)	524 (325 to 831)	12.9 (8 to 20.6)	17789 (11016 to 27970)	367 (227 to 580.4)	362 (199 to 632)	8 (4.5 to 13.7)	18152 (11275 to 28563)	375.1 (232.4 to 592.3)
	Male	25 (15 to 39)	2.6 (1.6 to 4.1)	115 (75 to 179)	10.7 (7.4 to 15.9)	23 (15 to 37)	2.7 (1.6 to 4.1)	602 (363 to 974)	52.9 (33.3 to 83.3)	13 (7 to 22)	1.3 (0.7 to 2.1)	616 (371 to 994)	54.2 (34.1 to 85.3)	51 (29 to 82)	2.3 (1.4 to 3.7)	257 (158 to 396)	10.2 (6.8 to 15.2)	46 (27 to 75)	2.3 (1.4 to 3.7)	1232 (701 to 1988)	45 (27.2 to 72.7)	28 (14 to 48)	1.1 (0.6 to 2)	1261 (715 to 2027)	46.2 (27.8 to 74.4)
South Africa	Both	3503 (3121 to 3969)	15.1 (13.3 to 17.5)	26395 (23712 to 29311)	112.7 (100.2 to 127)	2370 (2078 to 2744)	11.1 (9.6 to 13.1)	73519 (65651 to 81830)	298.1 (263.4 to 338.7)	2087 (1480 to 2836)	8.8 (6.2 to 12.1)	75606 (67462 to 84203)	306.9 (270.6 to 348.4)	8682 (7506 to 9966)	18.7 (16.4 to 21.3)	66279 (58405 to 74873)	138.5 (122.9 to 156.3)	5309 (4671 to 6033)	12.4 (11.1 to 14)	142083 (123201 to 163575)	292.4 (254.9 to 334.9)	5170 (3610 to 7084)	10.8 (7.6 to 14.7)	147253 (128115 to 169348)	303.2 (264.9 to 347.3)

		1990												2019											
		Incidence		Prevalence		Deaths		YLLs (Years of Life Lost)		YLDs (Years Lived with Disability)		DALYs (Disability-Adjusted Life Years)		Incidence		Prevalence		Deaths		YLLs (Years of Life Lost)		YLDs (Years Lived with Disability)		DALYs (Disability-Adjusted Life Years)	
Location	Sex	Number	Rate	Number	Rate	Number	Rate	Number	Rate	Number	Rate	Number	Rate	Number	Rate	Number	Rate	Number	Rate	Number	Rate	Number	Rate	Number	Rate
	Female	3439 (3063 to 3901)	26.5 (23.4 to 30.4)	25965 (23315 to 28830)	198.8 (176.9 to 223.2)	2321 (2031 to 2688)	18.8 (16.3 to 22.1)	72103 (64341 to 80331)	532.9 (471.4 to 601.8)	2045 (1449 to 2774)	15.5 (11 to 21.2)	74148 (66121 to 82726)	548.4 (484.3 to 619.5)	8540 (7370 to 9820)	32.1 (27.8 to 37)	65305 (57470 to 73827)	241.6 (212.9 to 272.6)	5209 (4576 to 5915)	20.5 (18.1 to 23.2)	139381 (120671 to 160760)	511.1 (442.7 to 587.9)	5074 (3536 to 6957)	18.8 (13.1 to 25.7)	144455 (125484 to 166351)	529.9 (461.8 to 609.3)
	Male	63 (53 to 81)	0.7 (0.6 to 0.9)	430 (362 to 523)	4.5 (3.8 to 5.6)	49 (40 to 64)	0.6 (0.5 to 0.8)	1416 (1195 to 1744)	13.6 (11.3 to 17.5)	42 (29 to 61)	0.4 (0.3 to 0.7)	1458 (1232 to 1793)	14.1 (11.7 to 18.1)	142 (120 to 169)	0.8 (0.7 to 0.9)	974 (839 to 1135)	4.9 (4.3 to 5.8)	100 (86 to 120)	0.6 (0.5 to 0.7)	2702 (2302 to 3226)	13.1 (11.2 to 15.6)	96 (66 to 137)	0.5 (0.3 to 0.7)	2799 (2385 to 3328)	13.6 (11.7 to 16.2)
South Sudan	Both	195 (143 to 254)	7.7 (5.8 to 9.9)	1239 (957 to 1577)	46.9 (36.9 to 58.4)	164 (122 to 211)	6.9 (5.1 to 8.7)	4975 (3523 to 6618)	177.6 (129.8 to 232.5)	107 (69 to 155)	4 (2.7 to 5.8)	5082 (3599 to 6744)	181.6 (132.7 to 237.7)	380 (252 to 539)	8.6 (6 to 11.9)	2499 (1768 to 3435)	53.9 (39.7 to 71.8)	291 (192 to 416)	7.3 (5 to 10)	9262 (5849 to 13765)	188.3 (122.5 to 271.5)	212 (125 to 332)	4.6 (2.8 to 7.1)	9474 (6002 to 14071)	192.9 (126.3 to 277.8)
	Female	171 (125 to 227)	14.6 (10.9 to 19)	1129 (876 to 1442)	95 (74.4 to 118.9)	140 (104 to 183)	12.6 (9.4 to 16.3)	4454 (3199 to 5939)	351.6 (256.5 to 467)	94 (60 to 137)	7.8 (5.1 to 11.4)	4548 (3268 to 6069)	359.5 (261.5 to 477.7)	349 (227 to 496)	15.9 (10.8 to 22.1)	2349 (1657 to 3239)	103 (76.3 to 136.8)	262 (169 to 375)	13.1 (8.9 to 18.1)	8623 (5353 to 12735)	349.9 (224.3 to 500.3)	195 (114 to 310)	8.5 (5.2 to 13.1)	8819 (5494 to 13011)	358.5 (229.7 to 513.4)
	Male	24 (13 to 39)	2.1 (1.2 to 3.4)	110 (66 to 170)	8.9 (5.8 to 13.4)	23 (13 to 37)	2.2 (1.2 to 3.5)	521 (290 to 851)	41.3 (23.4 to 65.7)	13 (7 to 21)	1.1 (0.6 to 1.7)	534 (297 to 870)	42.3 (24 to 67.2)	31 (16 to 53)	1.8 (1 to 3.1)	151 (86 to 243)	8.2 (4.9 to 13.1)	29 (16 to 49)	1.9 (1 to 3.1)	639 (328 to 1101)	33.5 (18 to 57.6)	17 (8 to 30)	0.9 (0.5 to 1.6)	655 (337 to 1127)	34.4 (18.5 to 59.1)
Spain	Both	17091 (16196 to 17938)	35.2 (33.3 to 36.9)	196217 (177960 to 219643)	391.1 (359.1 to 434)	6524 (6128 to 6798)	12.9 (12.1 to 13.4)	167792 (161143 to 173551)	351.5 (338.3 to 363.4)	13253 (9169 to 18346)	26.9 (18.7 to 37.2)	181045 (173298 to 188883)	378.4 (362.4 to 394.3)	29815 (23127 to 38294)	37 (28.5 to 47.8)	335529 (280250 to 406999)	408.6 (337.1 to 501.7)	8075 (7100 to 8851)	8.4 (7.6 to 9.1)	163798 (150241 to 175267)	205.3 (190 to 219.3)	22934 (14901 to 33244)	28.4 (18.2 to 41.7)	186731 (169266 to 204238)	233.7 (213.4 to 255.8)
	Female	16962 (16058 to 17819)	65.4 (61.9 to 68.7)	194924 (176969 to 218183)	718.1 (660.1 to 792.2)	6483 (6089 to 6754)	23.1 (21.9 to 24)	166851 (160160 to 172612)	661.7 (638.4 to 683.6)	13141 (9091 to 18201)	49.9 (34.3 to 68.9)	179992 (172219 to 187811)	711.6 (682.7 to 741.5)	29411 (22819 to 37841)	69.9 (53.6 to 90.5)	331702 (277321 to 402072)	771.4 (632.8 to 948.3)	7981 (7002 to 8763)	15.2 (13.8 to 16.3)	161975 (148544 to 173454)	390.1 (361 to 417.6)	22594 (14715 to 32790)	53.7 (34.3 to 78.9)	184569 (167164 to 202058)	443.8 (405.1 to 484.9)
	Male	129 (106 to 157)	0.5 (0.5 to 0.7)	1293 (1095 to 1555)	5.6 (4.8 to 6.6)	41 (36 to 46)	0.2 (0.2 to 0.2)	942 (838 to 1057)	4 (3.6 to 4.5)	112 (74 to 162)	0.5 (0.3 to 0.7)	1053 (935 to 1190)	4.5 (4 to 5.1)	404 (287 to 550)	1 (0.7 to 1.4)	3827 (2932 to 4979)	9.6 (7.4 to 12.7)	94 (77 to 114)	0.2 (0.2 to 0.3)	1822 (1526 to 2148)	4.7 (4 to 5.5)	340 (214 to 519)	0.9 (0.5 to 1.3)	2162 (1780 to 2615)	5.6 (4.6 to 6.7)
Sri Lanka	Both	967 (836 to 1102)	7.6 (6.6 to 8.7)	8767 (7714 to 9887)	69.6 (61.1 to 78.5)	554 (481 to 629)	4.8 (4.2 to 5.5)	18247 (15827 to 20820)	138.5 (120.3 to 157.8)	641 (440 to 890)	5 (3.4 to 6.9)	18888 (16392 to 21562)	143.5 (124.7 to 163.4)	4168 (3060 to 5609)	16.3 (12 to 21.9)	36770 (28384 to 47681)	142.1 (110.1 to 184.1)	1692 (1248 to 2246)	6.9 (5.1 to 9.1)	45872 (33445 to 61242)	177.8 (130 to 238.1)	2713 (1753 to 4071)	10.5 (6.7 to 15.8)	48585 (35693 to 65099)	188.3 (138.1 to 251.9)
	Female	956 (825 to 1091)	15.1 (13.1 to 17.2)	8697 (7640 to 9809)	138.4 (121.4 to 156.4)	546 (474 to 621)	9.5 (8.2 to 10.8)	18024 (15624 to 20583)	274.2 (238.2 to 312.9)	634 (434 to 879)	9.9 (6.8 to 13.6)	18658 (16156 to 21312)	284.1 (246.4 to 324.3)	4124 (3028 to 5557)	29.8 (21.9 to 40)	36463 (28145 to 47379)	261.1 (201.4 to 339.8)	1670 (1233 to 2222)	12.1 (9 to 16.1)	45298 (33070 to 60458)	325.7 (238.1 to 436.2)	2682 (1728 to 4030)	19.3 (12.4 to 29.1)	47980 (35214 to 64550)	345 (252.2 to 463.1)
	Male	11 (9 to 14)	0.2 (0.1 to 0.3)	70 (53 to 90)	1.2 (0.9 to 1.5)	8 (6 to 10)	0.1 (0.1 to 0.2)	223 (173 to 282)	3.6 (2.8 to 4.6)	7 (5 to 11)	0.1 (0.1 to 0.2)	230 (179 to 292)	3.7 (2.9 to 4.8)	44 (27 to 67)	0.4 (0.2 to 0.6)	308 (194 to 475)	2.6 (1.6 to 3.9)	22 (14 to 33)	0.2 (0.1 to 0.3)	573 (360 to 864)	4.8 (3.1 to 7.2)	31 (17 to 53)	0.3 (0.1 to 0.4)	605 (377 to 909)	5.1 (3.2 to 7.6)
Sudan	Both	756 (547 to 1054)	7 (5 to 9.8)	6228 (4885 to 8054)	59.3 (47.1 to 76)	524 (379 to 729)	5.1 (3.7 to 7.3)	18345 (13338 to 25518)	162 (117.4 to 225.2)	473 (302 to 748)	4.4 (2.8 to 6.9)	18818 (13734 to 26054)	166.3 (120.8 to 231.4)	2902 (1865 to 4062)	11.8 (8 to 16.2)	23436 (16080 to 32066)	95.5 (69 to 126.3)	1414 (970 to 1954)	6.4 (4.6 to 8.6)	49994 (31708 to 72122)	197.3 (132.3 to 275.8)	1839 (1004 to 2875)	7.4 (4.3 to 11.3)	51833 (33183 to 74982)	204.7 (138.1 to 285.6)
	Female	730 (522 to 1021)	13.7 (9.7 to 19.5)	6071 (4715 to 7936)	118.7 (93.8 to 153.7)	501 (358 to 706)	9.9 (7 to 14.4)	17734 (12723 to 24697)	318.5 (228.7 to 446.8)	457 (293 to 732)	8.6 (5.6 to 13.8)	18190 (13030 to 25216)	327.1 (234.7 to 460)	2846 (1827 to 4005)	24 (16.2 to 33)	23052 (15851 to 31616)	196.4 (143.4 to 259.2)	1375 (931 to 1914)	13.1 (9.4 to 17.7)	48936 (31076 to 70606)	401.4 (270.6 to 559.8)	1802 (980 to 2825)	15 (8.8 to 22.9)	50738 (32468 to 73398)	416.4 (282.4 to 577.1)
	Male	27 (14 to 44)	0.6 (0.3 to 0.9)	157 (96 to 244)	3.2 (2 to 4.8)	23 (12 to 38)	0.5 (0.3 to 0.9)	611 (321 to 1024)	11.9 (6.3 to 19.6)	16 (8 to 28)	0.3 (0.2 to 0.6)	628 (331 to 1050)	12.2 (6.5 to 20.2)	56 (32 to 91)	0.5 (0.3 to 0.9)	384 (239 to 587)	3.6 (2.3 to 5.4)	39 (23 to 61)	0.4 (0.2 to 0.6)	1058 (612 to 1685)	9.4 (5.5 to 14.8)	38 (20 to 63)	0.4 (0.2 to 0.6)	1096 (638 to 1741)	9.8 (5.7 to 15.4)
Suriname	Both	41 (35 to 47)	14.6 (12.6 to 16.7)	318 (275 to 367)	114.1 (98.9 to 130.8)	23 (20 to 27)	8.8 (7.7 to 9.9)	694 (592 to 801)	242.4 (209.3 to 279.3)	24 (16 to 33)	8.4 (5.6 to 11.7)	718 (612 to 830)	250.9 (217 to 288.7)	131 (104 to 162)	21.2 (16.9 to 25.8)	1015 (816 to 1239)	162.6 (131.8 to 197.1)	63 (51 to 77)	10.5 (8.5 to 12.7)	1789 (1424 to 2222)	285.1 (227.3 to 350.4)	75 (52 to 104)	12.1 (8.3 to 16.6)	1864 (1481 to 2316)	297.2 (237.5 to 365.4)

		1990												2019											
		Incidence		Prevalence		Deaths		YLLs (Years of Life Lost)		YLDs (Years Lived with Disability)		DALYs (Disability-Adjusted Life Years)		Incidence		Prevalence		Deaths		YLLs (Years of Life Lost)		YLDs (Years Lived with Disability)		DALYs (Disability-Adjusted Life Years)	
Location	Sex	Number	Rate	Number	Rate	Number	Rate	Number	Rate	Number	Rate	Number	Rate	Number	Rate	Number	Rate	Number	Rate	Number	Rate	Number	Rate	Number	Rate
	Female	40 (34 to 46)	27.7 (23.9 to 31.8)	314 (271 to 362)	218.2 (188.6 to 250.8)	23 (20 to 26)	16.3 (14.2 to 18.5)	680 (579 to 787)	461.4 (397.3 to 532)	23 (15 to 32)	16 (10.6 to 22.3)	703 (599 to 816)	477.4 (411.9 to 551)	129 (102 to 159)	39.5 (31.4 to 48.4)	1003 (807 to 1224)	305.7 (247 to 372.8)	62 (50 to 75)	19 (15.4 to 23)	1755 (1391 to 2175)	534.3 (424.5 to 657.5)	74 (51 to 102)	22.5 (15.5 to 31.2)	1829 (1448 to 2267)	556.8 (443 to 686.7)
	Male	1 (1 to 1)	0.6 (0.5 to 0.7)	4 (3 to 5)	3.1 (2.6 to 3.8)	1 (0 to 1)	0.5 (0.4 to 0.6)	14 (11 to 17)	10.8 (8.8 to 13)	0 (0 to 1)	0.3 (0.2 to 0.5)	14 (12 to 17)	11.1 (9.1 to 13.4)	2 (1 to 3)	0.7 (0.5 to 1)	12 (9 to 17)	4.3 (3.1 to 5.8)	1 (1 to 2)	0.6 (0.4 to 0.8)	35 (25 to 48)	12.1 (8.6 to 16.5)	1 (1 to 2)	0.4 (0.3 to 0.7)	36 (25 to 49)	12.5 (8.9 to 17.1)
Sweden	Both	5134 (4812 to 5452)	40.5 (38 to 43.1)	58792 (52990 to 65807)	445.6 (407.3 to 492)	1663 (1553 to 1741)	12 (11.3 to 12.5)	38110 (36472 to 39490)	311.4 (299.2 to 322)	4011 (2756 to 5498)	31.1 (21.4 to 42.7)	42121 (39948 to 44128)	342.5 (326.7 to 358.2)	7320 (5846 to 9046)	42.2 (33.4 to 52.4)	83446 (70285 to 98575)	464.2 (386.3 to 549.9)	1947 (1724 to 2110)	9.4 (8.6 to 10.2)	37205 (34370 to 39910)	217.7 (203.1 to 232.3)	5694 (3781 to 8194)	32.5 (21.3 to 47.1)	42899 (39125 to 46568)	250.3 (229.8 to 270.6)
	Female	5110 (4787 to 5429)	77.1 (72.5 to 82.3)	58534 (52776 to 65510)	839 (769 to 924.1)	1654 (1544 to 1732)	21.9 (20.8 to 22.8)	37909 (36285 to 39282)	598 (575.3 to 618.2)	3990 (2742 to 5471)	59 (40.4 to 81)	41899 (39733 to 43906)	657 (626.9 to 687.3)	7279 (5807 to 9006)	82.5 (65.4 to 102.7)	82999 (69913 to 98024)	905.1 (751.7 to 1071.4)	1934 (1712 to 2098)	17.8 (16.2 to 19.1)	36961 (34136 to 39669)	425.9 (398.1 to 454.2)	5657 (3754 to 8150)	63.5 (41.4 to 92.1)	42618 (38855 to 46295)	489.5 (449.7 to 529.4)
	Male	24 (20 to 28)	0.4 (0.3 to 0.4)	257 (217 to 304)	4.1 (3.5 to 4.8)	9 (8 to 11)	0.1 (0.1 to 0.2)	200 (177 to 224)	3.4 (3 to 3.7)	22 (14 to 30)	0.3 (0.2 to 0.5)	222 (196 to 250)	3.7 (3.3 to 4.1)	41 (31 to 53)	0.5 (0.4 to 0.6)	446 (359 to 547)	5 (4 to 6.1)	12 (10 to 14)	0.1 (0.1 to 0.1)	244 (210 to 280)	2.9 (2.6 to 3.4)	37 (25 to 54)	0.4 (0.3 to 0.6)	281 (240 to 325)	3.4 (2.9 to 3.9)
Switzerland	Both	5155 (4819 to 5478)	54.1 (50.6 to 57.6)	53203 (48556 to 58424)	552.4 (505.7 to 601.5)	1953 (1822 to 2044)	19.4 (18.2 to 20.2)	44576 (42589 to 46323)	477.8 (457 to 496.2)	3780 (2618 to 5150)	39.6 (27.5 to 53.9)	48355 (46004 to 50671)	517.3 (492.9 to 541.1)	6160 (4734 to 7936)	40.2 (30.7 to 52.1)	70274 (59193 to 83949)	449.1 (373.3 to 545)	1697 (1472 to 1859)	9.6 (8.5 to 10.5)	33018 (29714 to 35684)	218.2 (199.6 to 235.1)	4756 (3077 to 6851)	30.9 (19.8 to 45.4)	37774 (33790 to 41646)	249.1 (224.8 to 275)
	Female	5131 (4795 to 5451)	100.1 (93.6 to 106.5)	52982 (48344 to 58223)	1016.2 (933.6 to 1102.9)	1944 (1813 to 2034)	34.1 (32.2 to 35.5)	44368 (42373 to 46118)	893.5 (856.9 to 928.4)	3759 (2604 to 5124)	73.1 (50.7 to 99.7)	48128 (45791 to 50431)	966.6 (922.3 to 1010.8)	6134 (4718 to 7906)	77.5 (58.8 to 100.4)	69981 (58940 to 83594)	861.9 (713.3 to 1050.1)	1689 (1466 to 1852)	17.8 (15.9 to 19.4)	32863 (29569 to 35525)	421.8 (385.7 to 454.8)	4732 (3062 to 6819)	59.5 (38.2 to 87.6)	37595 (33616 to 41445)	481.3 (435.1 to 531.5)
	Male	24 (20 to 30)	0.6 (0.5 to 0.7)	221 (184 to 264)	5.2 (4.3 to 6.1)	10 (8 to 11)	0.2 (0.2 to 0.3)	207 (182 to 234)	4.9 (4.3 to 5.5)	20 (13 to 29)	0.5 (0.3 to 0.7)	228 (199 to 257)	5.4 (4.7 to 6.1)	26 (18 to 37)	0.4 (0.3 to 0.5)	292 (227 to 377)	3.9 (3.1 to 5)	8 (6 to 10)	0.1 (0.1 to 0.1)	155 (126 to 186)	2.2 (1.8 to 2.6)	24 (15 to 37)	0.3 (0.2 to 0.5)	179 (146 to 218)	2.5 (2.1 to 3)
Syrian Arab Republic	Both	425 (306 to 570)	6.6 (4.8 to 8.8)	4240 (3333 to 5313)	69.8 (55.5 to 86.8)	237 (171 to 314)	4 (2.9 to 5.3)	8300 (5972 to 11071)	125.2 (90.1 to 165.6)	295 (183 to 429)	4.7 (2.9 to 6.8)	8595 (6203 to 11485)	129.8 (93.6 to 172.2)	1882 (1311 to 2638)	13.4 (9.4 to 18.7)	17125 (12587 to 23029)	122.2 (91.6 to 163.1)	715 (504 to 1010)	5.4 (3.9 to 7.5)	22882 (15934 to 32655)	159.1 (111.8 to 224.2)	1255 (753 to 1924)	8.9 (5.5 to 13.6)	24137 (16868 to 34521)	168 (119.2 to 237.2)
	Female	422 (304 to 567)	13.7 (9.8 to 18.1)	4217 (3310 to 5288)	144.5 (114.7 to 179.7)	236 (169 to 312)	8.3 (6 to 11)	8245 (5916 to 11022)	256.7 (184.2 to 340.4)	293 (182 to 427)	9.6 (6 to 14.1)	8539 (6149 to 11423)	266.3 (191.5 to 353.6)	1873 (1303 to 2628)	26.9 (18.9 to 37.3)	17051 (12521 to 22955)	244.8 (184.6 to 324.5)	711 (500 to 1004)	11.3 (8.1 to 15.5)	22758 (15848 to 32520)	316.2 (222.8 to 446.3)	1248 (749 to 1916)	17.8 (10.9 to 27.1)	24006 (16758 to 34377)	334 (237.3 to 472.1)
	Male	3 (2 to 3)	0.1 (0.1 to 0.1)	23 (18 to 29)	0.8 (0.6 to 1)	2 (1 to 2)	0.1 (0 to 0.1)	55 (41 to 70)	1.6 (1.2 to 2)	2 (1 to 3)	0.1 (0 to 0.1)	57 (43 to 72)	1.6 (1.2 to 2.1)	9 (6 to 12)	0.1 (0.1 to 0.2)	74 (55 to 97)	1.1 (0.9 to 1.5)	4 (3 to 6)	0.1 (0.1 to 0.1)	124 (86 to 170)	1.8 (1.3 to 2.4)	7 (4 to 10)	0.1 (0.1 to 0.1)	130 (91 to 178)	1.9 (1.3 to 2.6)
Taiwan (Province of China)	Both	1981 (1877 to 2093)	10.8 (10.3 to 11.4)	19509 (17999 to 21066)	109.2 (100.8 to 119)	742 (712 to 772)	4.4 (4.2 to 4.6)	25590 (24545 to 26689)	138.9 (133.3 to 144.7)	1398 (949 to 1928)	7.7 (5.2 to 10.6)	26988 (25868 to 28196)	146.6 (140.5 to 153.2)	9330 (7069 to 12459)	25.5 (19.3 to 34)	96121 (77577 to 121000)	259 (207.3 to 330.2)	2333 (1828 to 3015)	6.1 (4.8 to 7.9)	66900 (51867 to 88146)	181.5 (140.3 to 240.1)	6695 (4260 to 9840)	18.3 (11.6 to 27.1)	73595 (56993 to 96000)	199.8 (154.7 to 261.9)
	Female	1968 (1863 to 2082)	22.4 (21.3 to 23.6)	19418 (17907 to 20974)	227.1 (209.1 to 248.3)	735 (705 to 765)	9 (8.6 to 9.4)	25406 (24361 to 26497)	287.4 (275.9 to 299.3)	1388 (942 to 1918)	15.9 (10.8 to 21.9)	26794 (25677 to 28007)	303.3 (290.6 to 316.8)	9289 (7038 to 12405)	49.4 (37.2 to 65.9)	95823 (77317 to 120631)	499.9 (399.1 to 638.3)	2317 (1816 to 2989)	11.6 (9 to 15)	66552 (51510 to 87735)	350.6 (270.3 to 464.2)	6664 (4241 to 9791)	35.3 (22.5 to 52.6)	73217 (56668 to 95519)	386 (298.6 to 506.2)
	Male	13 (11 to 15)	0.2 (0.1 to 0.2)	92 (77 to 108)	1.1 (0.9 to 1.2)	7 (6 to 8)	0.1 (0.1 to 0.1)	184 (165 to 206)	2.2 (2 to 2.4)	10 (6 to 13)	0.1 (0.1 to 0.2)	194 (173 to 216)	2.3 (2.1 to 2.6)	41 (29 to 57)	0.2 (0.2 to 0.3)	298 (208 to 416)	1.7 (1.2 to 2.4)	16 (12 to 22)	0.1 (0.1 to 0.1)	348 (253 to 473)	2 (1.5 to 2.6)	31 (19 to 48)	0.2 (0.1 to 0.3)	379 (276 to 513)	2.2 (1.6 to 2.9)
Tajikistan	Both	389 (344 to 437)	12.9 (11.4 to 14.5)	3458 (3045 to 3868)	116 (102.1 to 130.1)	236 (210 to 262)	8.1 (7.2 to 9)	7818 (6948 to 8771)	252.8 (225 to 284.1)	254 (174 to 348)	8.4 (5.7 to 11.4)	8072 (7188 to 9083)	261.1 (232.3 to 293.6)	862 (666 to 1107)	13.5 (10.6 to 17.2)	7237 (5807 to 8959)	113.5 (94 to 136.8)	445 (345 to 567)	8 (6.4 to 10.1)	15149 (11600 to 19507)	223.9 (174.4 to 284.7)	553 (355 to 806)	8.5 (5.6 to 12.2)	15702 (12018 to 20223)	232.4 (181.4 to 294.8)
	Female	388 (343 to 436)	24.4 (21.6 to 27.5)	3450 (3038 to 3859)	217.2 (191 to 242.8)	235 (209 to 262)	15 (13.3 to 16.7)	7800 (6932 to 8753)	485.8 (431.5 to 547.4)	253 (174 to 347)	15.8 (10.9 to 21.6)	8053 (7170 to 9065)	501.6 (445.9 to 565.1)	859 (663 to 1104)	26 (20.3 to 33)	7214 (5788 to 8931)	218.4 (180.1 to 264)	443 (344 to 565)	15.2 (12 to 19.1)	15094 (11559 to 19440)	433.8 (337.5 to 552.1)	551 (354 to 802)	16.4 (10.7 to 23.6)	15645 (11972 to 20153)	450.2 (350.6 to 571.6)
	Male	1 (1 to 1)	0.1 (0 to 0.1)	8 (6 to 10)	0.5 (0.4 to 0.6)	1 (0 to 1)	0 (0 to 0.1)	18 (15 to 22)	1.1 (0.9 to 1.4)	1 (0 to 1)	0 (0 to 0.1)	19 (16 to 23)	1.2 (1 to 1.4)	3 (2 to 4)	0.1 (0.1 to 0.2)	24 (17 to 31)	0.9 (0.6 to 1.1)	2 (1 to 3)	0.1 (0.1 to 0.1)	54 (40 to 73)	2 (1.4 to 2.7)	2 (1 to 3)	0.1 (0.1 to 0.1)	57 (42 to 76)	2.1 (1.5 to 2.8)

		1990												2019											
		Incidence		Prevalence		Deaths		YLLs (Years of Life Lost)		YLDs (Years Lived with Disability)		DALYs (Disability-Adjusted Life Years)		Incidence		Prevalence		Deaths		YLLs (Years of Life Lost)		YLDs (Years Lived with Disability)		DALYs (Disability-Adjusted Life Years)	
Location	Sex	Number	Rate	Number	Rate	Number	Rate	Number	Rate	Number	Rate	Number	Rate	Number	Rate	Number	Rate	Number	Rate	Number	Rate	Number	Rate	Number	Rate
Thailand	Both	4398 (3774 to 5109)	10.2 (8.8 to 11.7)	38435 (33719 to 43843)	90.7 (79.3 to 103.3)	2502 (2174 to 2866)	6.3 (5.5 to 7.2)	85210 (73182 to 98400)	190.3 (164.7 to 218.7)	2866 (1905 to 4017)	6.6 (4.4 to 9.2)	88076 (75497 to 101347)	196.9 (170.3 to 225.9)	17739 (12784 to 23841)	17.6 (12.7 to 23.5)	172494 (133214 to 219868)	169.9 (131.8 to 217)	6834 (5022 to 8995)	6.7 (4.9 to 8.8)	198558 (142209 to 266157)	195.8 (140 to 262.9)	12213 (7627 to 18371)	12.1 (7.5 to 18.2)	210771 (152046 to 282030)	207.9 (149.9 to 278.2)
	Female	4327 (3714 to 5042)	19 (16.4 to 22)	38015 (33322 to 43350)	170.2 (148.4 to 193.7)	2451 (2122 to 2816)	11.5 (10 to 13.3)	83805 (71925 to 97031)	358.1 (309.5 to 412.7)	2821 (1868 to 3958)	12.3 (8.2 to 17.2)	86625 (74254 to 99940)	370.4 (320.1 to 426.1)	17657 (12731 to 23719)	33.3 (23.9 to 44.6)	171882 (132818 to 219234)	321.6 (248.4 to 411.9)	6790 (4986 to 8937)	12.4 (9.1 to 16.4)	197518 (141439 to 264810)	371.6 (266 to 499.7)	12153 (7577 to 18290)	23 (14.3 to 34.7)	209671 (151315 to 280747)	394.6 (284 to 529)
	Male	71 (53 to 94)	0.4 (0.3 to 0.6)	420 (319 to 546)	2.3 (1.8 to 3.1)	51 (39 to 69)	0.4 (0.3 to 0.5)	1406 (1085 to 1820)	7.7 (5.9 to 10.3)	45 (29 to 67)	0.3 (0.2 to 0.4)	1451 (1116 to 1882)	8 (6.1 to 10.6)	83 (52 to 119)	0.2 (0.1 to 0.3)	612 (408 to 870)	1.3 (0.9 to 1.9)	44 (29 to 63)	0.1 (0.1 to 0.1)	1040 (669 to 1502)	2.3 (1.5 to 3.3)	60 (34 to 98)	0.1 (0.1 to 0.2)	1100 (705 to 1583)	2.4 (1.6 to 3.4)
Timor-Leste	Both	31 (22 to 42)	7.9 (5.7 to 10.6)	223 (170 to 293)	58.6 (46.4 to 74.3)	23 (16 to 31)	6.4 (4.7 to 8.4)	822 (585 to 1130)	194.4 (140.3 to 262.6)	18 (11 to 27)	4.5 (2.8 to 6.7)	840 (595 to 1156)	199 (143.6 to 267.9)	109 (73 to 150)	12.4 (8.7 to 17.1)	804 (563 to 1092)	90.7 (65.3 to 121.8)	68 (48 to 94)	8.2 (5.9 to 11.2)	2110 (1370 to 2957)	237 (157.8 to 331.7)	64 (38 to 98)	7.3 (4.4 to 10.9)	2174 (1411 to 3052)	244.2 (162.6 to 342.9)
	Female	30 (22 to 42)	16 (11.5 to 21.6)	221 (169 to 292)	119 (93.9 to 150.8)	23 (16 to 31)	13 (9.3 to 17)	815 (580 to 1122)	395.4 (283.9 to 535.5)	18 (11 to 26)	9.2 (5.8 to 13.6)	833 (589 to 1146)	404.7 (290.2 to 546.8)	107 (73 to 149)	24.9 (17.3 to 34.5)	798 (558 to 1083)	182.5 (131 to 245.7)	67 (47 to 93)	16.3 (11.6 to 22.4)	2087 (1357 to 2932)	476.7 (316.9 to 669.8)	64 (38 to 97)	14.5 (8.8 to 21.8)	2150 (1398 to 3025)	491.2 (326.6 to 691)
	Male	0 (0 to 0)	0.2 (0.1 to 0.3)	1 (1 to 2)	0.8 (0.5 to 1.2)	0 (0 to 0)	0.2 (0.1 to 0.3)	7 (4 to 10)	3.8 (2.4 to 5.7)	0 (0 to 0)	0.1 (0.1 to 0.2)	7 (4 to 10)	3.9 (2.5 to 5.9)	1 (1 to 2)	0.3 (0.2 to 0.5)	6 (4 to 10)	1.5 (0.9 to 2.3)	1 (1 to 1)	0.2 (0.2 to 0.4)	24 (14 to 36)	5.4 (3.4 to 8.4)	1 (0 to 1)	0.2 (0.1 to 0.3)	24 (15 to 38)	5.6 (3.4 to 8.6)
Togo	Both	152 (121 to 188)	10.1 (8.2 to 12.3)	1027 (848 to 1244)	67.5 (57 to 80.1)	111 (89 to 136)	8.1 (6.6 to 9.7)	3771 (2983 to 4660)	229.9 (183.5 to 281.5)	87 (58 to 123)	5.6 (3.8 to 7.9)	3858 (3054 to 4778)	235.6 (188.5 to 289)	596 (438 to 811)	13.9 (10.6 to 18.5)	4158 (3159 to 5536)	92.9 (73.1 to 120.3)	392 (295 to 523)	10.3 (8 to 13.5)	12737 (9260 to 17327)	270.6 (202.1 to 361.7)	347 (218 to 521)	7.8 (5 to 11.5)	13084 (9505 to 17798)	278.3 (208 to 373.1)
	Female	151 (120 to 187)	18.9 (15.3 to 23)	1021 (843 to 1238)	126.4 (106.7 to 149.8)	110 (88 to 135)	15 (12.2 to 18)	3749 (2963 to 4636)	429.4 (342.4 to 525.4)	86 (57 to 122)	10.5 (7.1 to 14.9)	3835 (3034 to 4751)	439.9 (351.8 to 538.7)	593 (435 to 808)	24.4 (18.5 to 32.6)	4140 (3143 to 5511)	165.2 (129.3 to 215.7)	390 (292 to 520)	17.6 (13.6 to 23.2)	12666 (9209 to 17253)	484.9 (358.8 to 651.1)	345 (217 to 519)	13.8 (8.8 to 20.4)	13011 (9434 to 17722)	498.7 (369.6 to 667.9)
	Male	1 (1 to 1)	0.1 (0.1 to 0.2)	5 (4 to 7)	0.7 (0.5 to 1.1)	1 (1 to 1)	0.1 (0.1 to 0.2)	23 (16 to 32)	3.1 (2.2 to 4.5)	1 (0 to 1)	0.1 (0.1 to 0.1)	23 (17 to 33)	3.2 (2.3 to 4.6)	3 (2 to 5)	0.2 (0.1 to 0.3)	19 (13 to 27)	1 (0.7 to 1.4)	2 (2 to 3)	0.2 (0.1 to 0.2)	71 (48 to 105)	3.8 (2.5 to 5.6)	2 (1 to 3)	0.1 (0.1 to 0.2)	73 (49 to 107)	3.9 (2.6 to 5.7)
Tokelau	Both	0 (0 to 0)	21.1 (12.3 to 30.4)	2 (1 to 3)	170.4 (117.3 to 227.7)	0 (0 to 0)	14.6 (8.6 to 20.9)	5 (3 to 8)	440.7 (257.1 to 642.1)	0 (0 to 0)	12.9 (7.1 to 20.1)	6 (3 to 8)	453.5 (266.8 to 659)	0 (0 to 1)	31.8 (21.8 to 44.5)	3 (3 to 5)	254.4 (185.9 to 344.7)	0 (0 to 0)	16.9 (11.7 to 22.9)	6 (4 to 9)	479.5 (325.9 to 674.8)	0 (0 to 0)	19.8 (12.3 to 29.4)	7 (5 to 9)	499.3 (339.5 to 702)
	Female	0 (0 to 0)	38.2 (22.3 to 55.3)	2 (1 to 3)	308.8 (213 to 413.1)	0 (0 to 0)	26.4 (15.5 to 37.9)	5 (3 to 8)	798.1 (464.4 to 1166.3)	0 (0 to 0)	23.3 (12.8 to 36.4)	6 (3 to 8)	821.4 (481.9 to 1193)	0 (0 to 1)	64 (44 to 89.6)	3 (3 to 5)	514.2 (375.4 to 699.3)	0 (0 to 0)	33.7 (23.4 to 46)	6 (4 to 9)	967.1 (658.3 to 1362.6)	0 (0 to 0)	39.9 (24.9 to 59.4)	7 (5 to 9)	1007 (685.6 to 1414.4)
	Male	0 (0 to 0)	0.2 (0.1 to 0.4)	0 (0 to 0)	1.5 (1 to 2.1)	0 (0 to 0)	0.2 (0.1 to 0.3)	0 (0 to 0)	4.4 (2.5 to 7.1)	0 (0 to 0)	0.1 (0.1 to 0.3)	0 (0 to 0)	4.5 (2.6 to 7.3)	0 (0 to 0)	0.3 (0.2 to 0.6)	0 (0 to 0)	2.2 (1.4 to 3.3)	0 (0 to 0)	0.2 (0.1 to 0.4)	0 (0 to 0)	5.2 (3.2 to 8.5)	0 (0 to 0)	0.2 (0.1 to 0.4)	0 (0 to 0)	5.4 (3.3 to 8.9)
Tonga	Both	14 (11 to 17)	22.7 (17.9 to 28.3)	110 (89 to 133)	180.7 (147.3 to 217.2)	9 (7 to 11)	15.3 (12.1 to 18.8)	278 (215 to 353)	448.6 (349.3 to 566.3)	8 (5 to 12)	13.7 (9 to 18.8)	287 (221 to 363)	462.3 (359.9 to 581.1)	24 (18 to 32)	29.4 (21.7 to 38.5)	197 (154 to 251)	238.7 (187.7 to 300.7)	14 (11 to 18)	17.5 (13.2 to 22.6)	401 (292 to 537)	481.2 (351.8 to 640.9)	15 (10 to 22)	18 (11.7 to 26.6)	416 (305 to 555)	499.2 (366.9 to 662.3)
	Female	14 (11 to 17)	43.2 (34 to 54.1)	110 (89 to 133)	345.8 (281.5 to 415.7)	9 (7 to 11)	28.8 (22.8 to 35.6)	278 (214 to 353)	856.8 (666.6 to 1081.5)	8 (5 to 12)	26.1 (17.2 to 35.8)	286 (221 to 362)	882.9 (687.5 to 1109.7)	24 (18 to 32)	55.6 (40.9 to 73.1)	197 (153 to 251)	455.4 (357.1 to 575.4)	14 (11 to 18)	32.3 (24.3 to 42)	401 (291 to 536)	919.7 (669.7 to 1228.5)	15 (10 to 22)	34.2 (22.2 to 50.8)	416 (304 to 554)	954 (699.3 to 1271.8)
	Male	0 (0 to 0)	0.1 (0 to 0.1)	0 (0 to 0)	0.5 (0.4 to 0.7)	0 (0 to 0)	0.1 (0 to 0.1)	0 (0 to 1)	1.3 (0.9 to 1.8)	0 (0 to 0)	0 (0 to 0.1)	0 (0 to 1)	1.3 (0.9 to 1.9)	0 (0 to 0)	0.1 (0.1 to 0.1)	0 (0 to 0)	0.6 (0.5 to 0.9)	0 (0 to 0)	0.1 (0 to 0.1)	1 (0 to 1)	1.5 (1 to 2.1)	0 (0 to 0)	0.1 (0 to 0.1)	1 (0 to 1)	1.5 (1.1 to 2.2)
Trinidad and Tobago	Both	242 (223 to 263)	27.6 (25.6 to 30)	1878 (1736 to 2037)	210.9 (194.5 to 229.3)	122 (114 to 130)	14.9 (13.9 to 15.9)	3606 (3345 to 3865)	400.4 (372.3 to 428.7)	141 (102 to 188)	15.8 (11.5 to 21.2)	3746 (3481 to 4005)	416.3 (386.6 to 444.4)	545 (401 to 732)	29.9 (21.9 to 39.9)	4478 (3374 to 5842)	244.2 (184.2 to 317.4)	230 (174 to 299)	12.7 (9.6 to 16.4)	6134 (4508 to 8149)	337.2 (247.4 to 449.1)	323 (206 to 469)	17.7 (11.4 to 25.9)	6457 (4763 to 8578)	354.9 (261.1 to 472.5)

		1990												2019											
		Incidence		Prevalence		Deaths		YLLs (Years of Life Lost)		YLDs (Years Lived with Disability)		DALYs (Disability-Adjusted Life Years)		Incidence		Prevalence		Deaths		YLLs (Years of Life Lost)		YLDs (Years Lived with Disability)		DALYs (Disability-Adjusted Life Years)	
Location	Sex	Number	Rate	Number	Rate	Number	Rate	Number	Rate	Number	Rate	Number	Rate	Number	Rate	Number	Rate	Number	Rate	Number	Rate	Number	Rate	Number	Rate
	Female	241 (222 to 262)	52.9 (48.9 to 57.6)	1870 (1728 to 2029)	407.3 (375.3 to 443.1)	121 (113 to 130)	27.7 (25.8 to 29.6)	3585 (3325 to 3843)	774.2 (719.3 to 829.7)	140 (101 to 188)	30.5 (22.1 to 40.9)	3725 (3461 to 3985)	804.6 (746.9 to 860.1)	537 (395 to 720)	57.7 (42.3 to 77.5)	4429 (3340 to 5784)	475.6 (357.6 to 620.3)	225 (171 to 293)	23.7 (17.9 to 30.9)	6014 (4405 to 8000)	653 (476.4 to 874.5)	318 (203 to 462)	34.3 (21.9 to 50.2)	6332 (4668 to 8403)	687.3 (503.7 to 917.4)
	Male	1 (1 to 1)	0.3 (0.2 to 0.3)	7 (6 to 9)	1.8 (1.6 to 2.1)	1 (1 to 1)	0.2 (0.2 to 0.2)	21 (18 to 23)	5 (4.4 to 5.5)	1 (0 to 1)	0.2 (0.1 to 0.2)	21 (19 to 24)	5.1 (4.6 to 5.7)	8 (5 to 10)	0.9 (0.6 to 1.2)	50 (35 to 68)	5.5 (3.9 to 7.5)	5 (3 to 6)	0.6 (0.4 to 0.8)	120 (84 to 165)	13.4 (9.5 to 18.2)	5 (3 to 7)	0.5 (0.3 to 0.8)	125 (88 to 171)	13.9 (9.8 to 18.9)
Tunisia	Both	695 (569 to 855)	12.7 (10.4 to 15.6)	6327 (5296 to 7511)	115.7 (97.5 to 136.6)	365 (300 to 446)	7.2 (5.9 to 8.8)	11620 (9585 to 14242)	205.5 (169.2 to 250.8)	461 (312 to 666)	8.3 (5.7 to 12.1)	12081 (9951 to 14821)	213.9 (176.3 to 261.6)	3172 (2272 to 4293)	23.9 (17.2 to 32.2)	29197 (22255 to 37786)	219.3 (168.1 to 282)	1061 (771 to 1416)	8.3 (6.1 to 11)	31544 (22596 to 42620)	235 (168.2 to 316)	2144 (1329 to 3280)	16.1 (10.1 to 24.6)	33687 (24084 to 45533)	251.1 (179.7 to 337.4)
	Female	680 (557 to 840)	25 (20.4 to 30.9)	6221 (5208 to 7373)	230.5 (194.1 to 271.9)	354 (291 to 436)	14 (11.5 to 17.3)	11368 (9354 to 13948)	405.7 (333.5 to 497.9)	451 (302 to 653)	16.5 (11.2 to 23.9)	11818 (9707 to 14518)	422.1 (347.5 to 518.3)	3129 (2241 to 4237)	46 (33 to 62.2)	28854 (21971 to 37331)	424 (323.8 to 546)	1039 (753 to 1386)	15.7 (11.5 to 20.9)	31032 (22110 to 42022)	452.5 (323.5 to 610.4)	2112 (1314 to 3227)	31 (19.3 to 47.4)	33143 (23657 to 44761)	483.5 (345.2 to 650.3)
	Male	15 (10 to 20)	0.6 (0.4 to 0.8)	106 (77 to 143)	4.1 (3 to 5.4)	10 (7 to 14)	0.5 (0.3 to 0.6)	253 (171 to 354)	9.6 (6.6 to 13.3)	10 (6 to 16)	0.4 (0.2 to 0.6)	263 (178 to 368)	10 (6.8 to 13.8)	43 (26 to 69)	0.7 (0.4 to 1.1)	343 (226 to 520)	5.6 (3.7 to 8.3)	22 (13 to 34)	0.4 (0.2 to 0.6)	512 (301 to 812)	8.2 (4.8 to 12.8)	32 (17 to 55)	0.5 (0.3 to 0.9)	544 (317 to 860)	8.7 (5.2 to 13.5)
Turkey	Both	4133 (3279 to 5244)	10.2 (8.1 to 12.9)	36174 (30246 to 44134)	90.6 (76.4 to 109.9)	2516 (2001 to 3203)	6.5 (5.2 to 8.3)	81484 (64427 to 104083)	193.7 (153.9 to 246)	2654 (1764 to 3840)	6.5 (4.3 to 9.4)	84138 (66672 to 107245)	200.2 (159.2 to 252.8)	17380 (13655 to 21843)	18.9 (14.9 to 23.8)	157687 (128440 to 192668)	171.1 (139.8 to 208.9)	6049 (4828 to 7484)	6.8 (5.5 to 8.4)	167844 (131471 to 211433)	180.6 (142.1 to 227.2)	11566 (7814 to 16767)	12.5 (8.5 to 18.2)	179410 (141036 to 225876)	193.1 (152.4 to 242.2)
	Female	4047 (3201 to 5151)	19.4 (15.4 to 24.6)	35606 (29744 to 43550)	173.4 (145.5 to 210.6)	2450 (1949 to 3126)	12.3 (9.8 to 15.6)	79721 (63118 to 102190)	372.9 (295.4 to 475.9)	2598 (1722 to 3757)	12.4 (8.3 to 18)	82319 (65124 to 105081)	385.3 (306 to 488.2)	17130 (13440 to 21566)	36.1 (28.3 to 45.5)	155738 (127069 to 190476)	327.7 (267.8 to 400.7)	5926 (4729 to 7337)	12.6 (10.1 to 15.7)	164911 (129220 to 207565)	345.9 (271.3 to 435.3)	11381 (7681 to 16496)	24 (16.1 to 34.7)	176292 (138548 to 222264)	369.8 (291 to 464.6)
	Male	86 (56 to 131)	0.5 (0.3 to 0.8)	568 (390 to 815)	3.2 (2.2 to 4.6)	66 (43 to 100)	0.4 (0.3 to 0.6)	1763 (1103 to 2692)	9.4 (6.1 to 14.5)	56 (32 to 88)	0.3 (0.2 to 0.5)	1819 (1149 to 2772)	9.8 (6.4 to 14.9)	250 (177 to 351)	0.6 (0.4 to 0.8)	1950 (1424 to 2688)	4.6 (3.4 to 6.3)	123 (87 to 165)	0.3 (0.2 to 0.4)	2933 (2100 to 3987)	6.8 (4.9 to 9.2)	185 (115 to 288)	0.4 (0.3 to 0.7)	3118 (2237 to 4234)	7.3 (5.2 to 9.8)
Turkmenistan	Both	272 (253 to 292)	12.5 (11.6 to 13.4)	2396 (2192 to 2612)	112.5 (102 to 123.5)	155 (145 to 164)	7.5 (7.1 to 8)	5212 (4882 to 5549)	230.8 (216.2 to 245.9)	179 (121 to 244)	8.2 (5.6 to 11.1)	5391 (5054 to 5749)	239 (224.2 to 254.7)	716 (560 to 931)	15.2 (11.9 to 19.6)	6298 (5113 to 7867)	135.2 (111 to 167.2)	301 (237 to 383)	6.9 (5.4 to 8.6)	10276 (8034 to 13271)	214.1 (168.5 to 275.1)	476 (310 to 703)	10.1 (6.7 to 14.8)	10752 (8441 to 13933)	224.3 (177 to 288.5)
	Female	272 (252 to 291)	22.8 (21.1 to 24.4)	2388 (2184 to 2603)	202.4 (184.2 to 221.8)	154 (145 to 164)	13.3 (12.5 to 14.1)	5197 (4866 to 5533)	428 (400.6 to 456.2)	178 (121 to 244)	14.9 (10.1 to 20.1)	5375 (5038 to 5734)	442.8 (414.4 to 471.8)	707 (553 to 920)	28.8 (22.6 to 37.3)	6237 (5055 to 7788)	255.4 (208.4 to 317.3)	296 (233 to 378)	12.5 (9.9 to 15.9)	10148 (7924 to 13113)	408.3 (320.1 to 526)	470 (306 to 695)	19.1 (12.5 to 28.1)	10618 (8334 to 13794)	427.4 (336.8 to 553.5)
	Male	1 (1 to 1)	0.1 (0.1 to 0.1)	8 (7 to 10)	0.9 (0.7 to 1)	0 (0 to 1)	0.1 (0.1 to 0.1)	15 (14 to 17)	1.5 (1.4 to 1.7)	1 (0 to 1)	0.1 (0 to 0.1)	16 (15 to 18)	1.6 (1.4 to 1.7)	9 (6 to 12)	0.5 (0.4 to 0.6)	62 (46 to 81)	3.2 (2.5 to 4.2)	5 (3 to 6)	0.3 (0.2 to 0.4)	128 (97 to 168)	6.6 (5.1 to 8.6)	6 (4 to 9)	0.3 (0.2 to 0.5)	134 (102 to 175)	7 (5.3 to 9.1)
Tuvalu	Both	2 (1 to 2)	22.7 (17.4 to 29.6)	13 (10 to 16)	171.9 (137.3 to 211.9)	1 (1 to 1)	16.7 (12.9 to 21.3)	37 (28 to 49)	497 (373.5 to 649)	1 (1 to 1)	13.5 (9.1 to 19.4)	38 (29 to 50)	510.5 (383.1 to 662.7)	3 (2 to 4)	29.7 (20.6 to 42.4)	24 (18 to 33)	223.9 (167.1 to 305.6)	2 (1 to 3)	18.5 (13 to 25.9)	55 (38 to 79)	512 (352.5 to 734.4)	2 (1 to 3)	17.8 (10.9 to 27.5)	57 (39 to 82)	529.8 (367.3 to 761.6)
	Female	2 (1 to 2)	39.2 (29.7 to 51.5)	13 (10 to 16)	298.9 (236.6 to 370.6)	1 (1 to 1)	28.4 (21.8 to 36.4)	37 (28 to 49)	868 (649.5 to 1137.1)	1 (1 to 1)	23.5 (15.7 to 33.8)	38 (28 to 50)	891.5 (665.8 to 1162.6)	3 (2 to 4)	57.9 (39.9 to 83.2)	24 (18 to 33)	438.5 (324.5 to 602.1)	2 (1 to 3)	35.1 (24.5 to 49.4)	55 (38 to 79)	1007.7 (691 to 1456.2)	2 (1 to 3)	34.9 (21.2 to 54.2)	57 (39 to 82)	1042.6 (714.4 to 1505)
	Male	0 (0 to 0)	0.3 (0.2 to 0.5)	0 (0 to 0)	1.8 (1.3 to 2.8)	0 (0 to 0)	0.3 (0.2 to 0.5)	0 (0 to 0)	6.2 (3.9 to 10.4)	0 (0 to 0)	0.2 (0.1 to 0.3)	0 (0 to 0)	6.4 (4 to 10.7)	0 (0 to 0)	0.4 (0.2 to 0.6)	0 (0 to 0)	2.4 (1.6 to 3.4)	0 (0 to 0)	0.3 (0.2 to 0.5)	0 (0 to 1)	7 (4.3 to 10.8)	0 (0 to 0)	0.3 (0.1 to 0.4)	0 (0 to 1)	7.3 (4.5 to 11.2)
Uganda	Both	824 (611 to 1053)	12.1 (9.2 to 15.2)	5055 (3893 to 6293)	71 (56.2 to 86.8)	671 (506 to 849)	10.6 (8.2 to 13.1)	20064 (14472 to 26103)	269.3 (199.3 to 345.3)	457 (303 to 651)	6.5 (4.3 to 9)	20521 (14826 to 26748)	275.7 (203.8 to 353.5)	3145 (2342 to 4053)	19.1 (14.5 to 24)	21833 (16714 to 27635)	127.4 (100 to 156.7)	2112 (1596 to 2696)	14.2 (11 to 17.7)	67932 (50255 to 88670)	378.5 (284.2 to 488.4)	1814 (1185 to 2604)	10.6 (7.1 to 14.9)	69746 (51636 to 91567)	389.1 (292.7 to 502.8)
	Female	654 (461 to 866)	17.6 (12.8 to 22.9)	4307 (3239 to 5445)	114.1 (87.6 to 141.5)	509 (368 to 665)	14.5 (10.7 to 18.6)	16508 (11394 to 21992)	413.9 (291.9 to 547.6)	370 (235 to 543)	9.7 (6.2 to 14.1)	16879 (11684 to 22422)	423.6 (298.9 to 560.5)	2937 (2158 to 3839)	31.4 (23.5 to 40.1)	20719 (15763 to 26313)	216.7 (167.6 to 270.3)	1935 (1444 to 2499)	22.4 (17.1 to 28.4)	63785 (46596 to 83229)	638.4 (473.1 to 832.7)	1698 (1101 to 2453)	17.7 (11.6 to 25.2)	65483 (47967 to 85665)	656.1 (486 to 859.3)

		1990												2019											
		Incidence		Prevalence		Deaths		YLLs (Years of Life Lost)		YLDs (Years Lived with Disability)		DALYs (Disability-Adjusted Life Years)		Incidence		Prevalence		Deaths		YLLs (Years of Life Lost)		YLDs (Years Lived with Disability)		DALYs (Disability-Adjusted Life Years)	
Location	Sex	Number	Rate	Number	Rate	Number	Rate	Number	Rate	Number	Rate	Number	Rate	Number	Rate	Number	Rate	Number	Rate	Number	Rate	Number	Rate	Number	Rate
	Male	171 (124 to 227)	6.7 (4.9 to 8.7)	747 (556 to 985)	26.2 (19.8 to 34.3)	162 (118 to 213)	6.7 (4.9 to 8.8)	3556 (2565 to 4701)	122.1 (90 to 160.2)	86 (55 to 127)	3.1 (2.1 to 4.6)	3642 (2627 to 4825)	125.2 (92.2 to 164.1)	208 (143 to 299)	4.2 (2.9 to 5.9)	1114 (801 to 1568)	20 (14.6 to 27.1)	177 (122 to 254)	3.9 (2.7 to 5.6)	4147 (2831 to 5974)	71.7 (49.9 to 102.2)	116 (71 to 177)	2.2 (1.3 to 3.2)	4263 (2911 to 6159)	73.9 (51.4 to 105.3)
Ukraine	Both	18346 (17333 to 19472)	27.2 (25.6 to 28.8)	173184 (158562 to 191238)	254.6 (233.7 to 279.7)	10203 (9752 to 10695)	14.8 (14.1 to 15.5)	295910 (282043 to 309610)	440 (420.6 to 460.8)	12155 (8451 to 16477)	18 (12.5 to 24.5)	308066 (293432 to 322833)	458 (437.3 to 479.6)	17869 (13990 to 22466)	26 (20.3 to 32.8)	174785 (143842 to 211757)	249.8 (204.9 to 303.4)	8561 (6796 to 10688)	11.8 (9.4 to 14.8)	229886 (179746 to 291255)	337.4 (261.9 to 430.8)	12239 (8126 to 17287)	17.7 (11.8 to 25.4)	242124 (192649 to 303818)	355.2 (281.5 to 447.1)
	Female	18328 (17315 to 19452)	47 (44.3 to 49.9)	172986 (158374 to 191032)	432.3 (398.4 to 470.6)	10193 (9743 to 10684)	24.6 (23.5 to 25.8)	295638 (281770 to 309318)	773.2 (739.1 to 811)	12140 (8439 to 16453)	31 (21.6 to 42.2)	307778 (293149 to 322524)	804.2 (767.2 to 843.9)	17667 (13772 to 22279)	45 (35 to 57.2)	173360 (142495 to 210314)	428.8 (348.7 to 523.2)	8473 (6708 to 10581)	19.8 (15.6 to 24.9)	227587 (177107 to 288924)	589.5 (455.6 to 756.2)	12090 (8005 to 17149)	30.6 (20.2 to 43.9)	239676 (190349 to 301212)	620.1 (486.2 to 785)
	Male	18 (16 to 22)	0.1 (0.1 to 0.1)	197 (163 to 240)	0.8 (0.6 to 0.9)	9 (8 to 11)	0 (0 to 0)	272 (240 to 309)	1 (0.9 to 1.1)	16 (10 to 22)	0.1 (0 to 0.1)	288 (254 to 327)	1.1 (1 to 1.2)	202 (145 to 278)	0.7 (0.5 to 1)	1425 (1031 to 1930)	5 (3.7 to 6.7)	88 (66 to 115)	0.3 (0.2 to 0.4)	2299 (1705 to 3027)	8 (6 to 10.5)	149 (92 to 229)	0.5 (0.3 to 0.8)	2448 (1836 to 3211)	8.5 (6.5 to 11.2)
United Arab Emirates	Both	91 (67 to 123)	14 (10 to 19.6)	775 (608 to 999)	123.8 (97.2 to 157.8)	48 (35 to 65)	9.4 (6.8 to 13.3)	1741 (1285 to 2336)	236.5 (169.9 to 328.2)	59 (38 to 89)	8.9 (5.7 to 13.4)	1800 (1326 to 2414)	245.4 (176.6 to 341.3)	1271 (906 to 1738)	15 (11.5 to 19.4)	10882 (8046 to 14563)	136.5 (110 to 166.8)	455 (332 to 609)	7.3 (5.6 to 9.3)	17772 (12806 to 24064)	194.5 (148.3 to 253.1)	834 (519 to 1265)	9.9 (6.5 to 14.2)	18606 (13399 to 25270)	204.4 (156.2 to 266.2)
	Female	86 (63 to 119)	40.8 (28.7 to 57.8)	742 (577 to 967)	359 (280.7 to 461.4)	45 (32 to 62)	25.5 (17.9 to 36.3)	1644 (1197 to 2243)	718 (506.1 to 1008.3)	56 (36 to 86)	25.8 (16.3 to 39.5)	1699 (1239 to 2336)	743.8 (526.3 to 1041.7)	1220 (872 to 1648)	57.5 (43.3 to 73.7)	10461 (7740 to 13923)	519.3 (412.8 to 644.5)	430 (313 to 571)	26.2 (20 to 33.6)	16825 (12024 to 22574)	753.5 (565.4 to 977.7)	796 (495 to 1212)	37.4 (24.2 to 54.5)	17621 (12543 to 23549)	791 (594 to 1020.9)
	Male	4 (2 to 7)	1.2 (0.5 to 2.1)	33 (18 to 52)	7.8 (4.3 to 12.1)	3 (1 to 5)	1 (0.4 to 1.7)	98 (45 to 171)	21.1 (9.5 to 36.4)	3 (1 to 5)	0.8 (0.3 to 1.3)	101 (47 to 176)	21.9 (9.8 to 37.7)	51 (22 to 103)	1.1 (0.5 to 2)	421 (208 to 797)	8.5 (4.9 to 14.1)	25 (11 to 50)	0.7 (0.3 to 1.2)	947 (397 to 1935)	16.3 (7.6 to 29.3)	38 (16 to 80)	0.8 (0.4 to 1.5)	985 (415 to 2013)	17.1 (8.1 to 30.7)
United Kingdom	Both	42065 (40630 to 43112)	53 (51.5 to 54.3)	435227 (399818 to 480467)	537.1 (499.4 to 584.3)	17431 (16531 to 17901)	20.6 (19.6 to 21.1)	396898 (384809 to 404168)	510.1 (496.3 to 519.3)	30854 (21758 to 42178)	38.6 (27.2 to 52.7)	427752 (413199 to 441698)	548.8 (531.6 to 566.1)	53010 (42241 to 65883)	49.2 (38.9 to 61.7)	573393 (481491 to 685227)	522.1 (433.8 to 633.6)	15256 (13900 to 16106)	12.3 (11.4 to 12.9)	300854 (284468 to 313473)	282.9 (270.3 to 294.1)	39983 (26635 to 57704)	37 (24.4 to 54)	340837 (319900 to 362996)	319.9 (302.3 to 340.9)
	Female	41846 (40409 to 42896)	98.2 (95.4 to 100.5)	433247 (397995 to 478254)	987.7 (921.9 to 1069.6)	17339 (16441 to 17806)	36 (34.6 to 36.8)	394955 (382888 to 402198)	953.8 (931.2 to 970.9)	30670 (21633 to 41952)	71.4 (50.1 to 97.3)	425626 (411144 to 439501)	1025.2 (994.6 to 1056.8)	52730 (41949 to 65672)	94.3 (74.3 to 118.8)	570689 (479014 to 682198)	1000.3 (829.8 to 1217.1)	15162 (13811 to 16011)	22.6 (21 to 23.6)	299110 (282746 to 311743)	542.8 (519.4 to 564.3)	39743 (26491 to 57420)	71 (46.8 to 103.9)	338853 (317922 to 360908)	613.8 (579.9 to 654)
	Male	220 (208 to 232)	0.6 (0.6 to 0.6)	1979 (1790 to 2206)	5.3 (4.8 to 5.9)	93 (89 to 96)	0.3 (0.2 to 0.3)	1943 (1883 to 2000)	5.3 (5.1 to 5.4)	183 (131 to 246)	0.5 (0.3 to 0.7)	2126 (2040 to 2214)	5.8 (5.6 to 6)	280 (219 to 354)	0.5 (0.4 to 0.6)	2704 (2210 to 3279)	4.9 (4 to 6)	94 (86 to 100)	0.2 (0.2 to 0.2)	1745 (1641 to 1841)	3.3 (3.1 to 3.5)	240 (162 to 337)	0.4 (0.3 to 0.6)	1984 (1850 to 2136)	3.7 (3.5 to 4)
United Republic of Tanzania	Both	1081 (874 to 1300)	9.6 (7.9 to 11.4)	7067 (5886 to 8392)	59.2 (50 to 69.7)	863 (705 to 1033)	8.4 (7 to 9.9)	25475 (20354 to 31056)	205.2 (166.1 to 246.3)	608 (421 to 833)	5.2 (3.6 to 7)	26083 (20827 to 31845)	210.4 (170.2 to 253.3)	3728 (2886 to 4627)	13.8 (11.1 to 16.8)	24976 (19663 to 30749)	87.6 (71.1 to 105.1)	2602 (2073 to 3165)	10.7 (8.7 to 12.7)	76933 (59179 to 96781)	261.8 (205.7 to 322.4)	2126 (1399 to 3019)	7.5 (5.1 to 10.5)	79060 (60868 to 99360)	269.4 (211.3 to 331.2)
	Female	942 (749 to 1153)	15.6 (12.5 to 18.7)	6396 (5267 to 7599)	101.8 (84.7 to 119.7)	734 (589 to 887)	13.2 (10.6 to 15.7)	22503 (17738 to 27892)	341.9 (272.8 to 418.8)	534 (365 to 736)	8.5 (5.9 to 11.6)	23038 (18177 to 28516)	350.5 (279.4 to 429.1)	3452 (2638 to 4324)	23.9 (18.7 to 29.2)	23501 (18336 to 29187)	155.8 (124.8 to 188.6)	2363 (1862 to 2904)	17.8 (14.2 to 21.4)	71548 (54115 to 90431)	458.7 (352.3 to 570.3)	1971 (1290 to 2831)	13.1 (8.8 to 18.4)	73519 (55516 to 92832)	471.8 (362.8 to 588.2)
	Male	139 (90 to 209)	3.1 (2 to 4.6)	671 (453 to 997)	13.5 (9.4 to 19.3)	129 (83 to 192)	3.1 (2 to 4.6)	2972 (1936 to 4565)	58.3 (38.1 to 87.2)	74 (41 to 123)	1.5 (0.9 to 2.4)	3046 (1983 to 4671)	59.8 (39.2 to 89.8)	276 (167 to 440)	2.7 (1.7 to 4.3)	1475 (924 to 2268)	13.5 (8.7 to 20.3)	238 (142 to 373)	2.6 (1.6 to 4)	5386 (3194 to 8683)	47.8 (28.7 to 74.9)	155 (82 to 267)	1.5 (0.8 to 2.5)	5541 (3281 to 8949)	49.2 (29.6 to 77.1)
United States of America	Both	190935 (184205 to 195770)	63.6 (61.5 to 65.2)	1973740 (1849231 to 2135418)	648.7 (611.8 to 697.2)	48212 (45756 to 49506)	15.6 (14.9 to 16)	1199579 (1165785 to 1222052)	411.4 (400.5 to 418.6)	139405 (98758 to 187772)	46.1 (32.7 to 62.5)	1338984 (1287599 to 1397544)	457.5 (440.9 to 477.5)	254486 (210821 to 308184)	50.2 (41.2 to 61.1)	2817911 (2398717 to 3278681)	543.2 (460.5 to 634.4)	55021 (51008 to 57900)	10.1 (9.5 to 10.5)	1212430 (1157030 to 1261823)	246.6 (236.1 to 256.3)	190962 (128770 to 267492)	37.5 (25.1 to 52.6)	1403392 (1314386 to 1499020)	284.1 (267.1 to 303.3)

		1990												2019											
		Incidence		Prevalence		Deaths		YLLs (Years of Life Lost)		YLDs (Years Lived with Disability)		DALYs (Disability-Adjusted Life Years)		Incidence		Prevalence		Deaths		YLLs (Years of Life Lost)		YLDs (Years Lived with Disability)		DALYs (Disability-Adjusted Life Years)	
Location	Sex	Number	Rate	Number	Rate	Number	Rate	Number	Rate	Number	Rate	Number	Rate	Number	Rate	Number	Rate	Number	Rate	Number	Rate	Number	Rate	Number	Rate
	Female	189273 (182651 to 194086)	115.8 (112.3 to 118.5)	1956809 (1833565 to 2117207)	1168.1 (1105 to 1247.8)	47836 (45387 to 49122)	27.7 (26.5 to 28.3)	1190741 (1156956 to 1213211)	761.8 (743.9 to 775.1)	137983 (97735 to 185806)	83.5 (59.1 to 112.8)	1328724 (1277529 to 1387097)	845.3 (816 to 881.2)	251531 (207355 to 305346)	94.2 (77.4 to 115.1)	2787997 (2370169 to 3247109)	1013.7 (857.9 to 1188.9)	54402 (50420 to 57271)	18.4 (17.3 to 19.2)	1199215 (1144039 to 1248457)	466.3 (446.5 to 484.8)	188455 (126777 to 264364)	70.1 (46.8 to 98.5)	1387670 (1300006 to 1483612)	536.4 (504.9 to 572.8)
	Male	1663 (1584 to 1748)	1.2 (1.2 to 1.3)	16931 (15592 to 18672)	12.7 (11.7 to 14)	376 (361 to 392)	0.3 (0.3 to 0.3)	8837 (8527 to 9195)	6.6 (6.4 to 6.9)	1422 (1023 to 1893)	1.1 (0.8 to 1.4)	10259 (9666 to 10880)	7.7 (7.2 to 8.2)	2956 (2375 to 3634)	1.2 (1 to 1.5)	29913 (25215 to 35846)	12.2 (10.3 to 14.6)	619 (574 to 662)	0.2 (0.2 to 0.3)	13215 (12364 to 14024)	5.4 (5.1 to 5.7)	2507 (1689 to 3484)	1 (0.7 to 1.4)	15722 (14495 to 17073)	6.4 (5.9 to 7)
United States Virgin Islands	Both	33 (27 to 39)	35.3 (29.4 to 41.5)	269 (225 to 317)	280.3 (237.7 to 328.1)	15 (12 to 17)	17 (14.4 to 19.8)	433 (358 to 512)	442.1 (366.9 to 520.1)	20 (13 to 28)	20.7 (14.1 to 28.7)	453 (374 to 536)	462.9 (384.5 to 543.6)	70 (56 to 85)	41.9 (33.2 to 52.1)	587 (483 to 706)	347.4 (281 to 425.3)	28 (23 to 33)	16.5 (13.4 to 19.9)	652 (520 to 800)	400.3 (313.9 to 496.5)	42 (28 to 59)	25.2 (16.8 to 35.8)	694 (550 to 849)	425.4 (332.3 to 527.9)
	Female	33 (27 to 39)	64.7 (53.7 to 76.2)	266 (223 to 314)	519.6 (440.3 to 609.8)	14 (12 to 17)	30.2 (25.6 to 35.2)	424 (349 to 503)	812.7 (673.5 to 958)	19 (13 to 27)	38.2 (25.8 to 53.2)	444 (367 to 525)	850.9 (706 to 1003.2)	68 (55 to 84)	74.5 (58.7 to 93.1)	576 (472 to 697)	624.8 (503 to 770)	27 (22 to 32)	28.1 (22.7 to 34.1)	629 (500 to 772)	710.1 (551.1 to 882.7)	41 (28 to 58)	44.8 (29.6 to 64.2)	671 (531 to 824)	754.9 (586.2 to 941.9)
	Male	1 (0 to 1)	1.4 (1 to 1.8)	3 (2 to 4)	7.7 (5.9 to 10.2)	0 (0 to 0)	1 (0.8 to 1.4)	9 (7 to 12)	21.4 (15.9 to 28.1)	0 (0 to 0)	0.8 (0.5 to 1.2)	9 (7 to 12)	22.2 (16.5 to 29.1)	2 (1 to 2)	2.2 (1.6 to 3)	11 (7 to 15)	13.3 (9.3 to 18.2)	1 (1 to 1)	1.4 (1 to 1.8)	22 (16 to 30)	28.6 (20.7 to 37.8)	1 (1 to 2)	1.3 (0.8 to 2)	23 (17 to 32)	29.9 (21.6 to 39.5)
Uruguay	Both	1418 (1325 to 1510)	38.9 (36.3 to 41.4)	12591 (11552 to 13844)	343 (316.5 to 374.4)	807 (764 to 842)	21.6 (20.5 to 22.6)	20425 (19548 to 21238)	569.9 (545.7 to 592.7)	952 (668 to 1298)	26.1 (18.3 to 35.7)	21376 (20437 to 22303)	596 (569.2 to 621.9)	1959 (1519 to 2470)	40.3 (31.1 to 51.2)	18858 (15795 to 22687)	387.3 (318.3 to 470.9)	936 (849 to 1018)	17.3 (15.8 to 18.7)	19767 (18265 to 21296)	417.7 (388.1 to 451.5)	1374 (925 to 1984)	28.5 (18.8 to 41.3)	21141 (19489 to 22931)	446.2 (411.6 to 486.6)
	Female	1408 (1316 to 1500)	71.1 (66.3 to 75.9)	12516 (11484 to 13770)	627.5 (579.2 to 682.7)	800 (757 to 836)	38.5 (36.6 to 40.1)	20268 (19396 to 21077)	1055.3 (1010.9 to 1097.9)	944 (662 to 1290)	47.8 (33.4 to 65.6)	21212 (20268 to 22138)	1103.1 (1053.7 to 1150.8)	1942 (1505 to 2450)	72.6 (55.8 to 92.6)	18730 (15694 to 22546)	700 (572.5 to 854.7)	926 (840 to 1008)	30 (27.5 to 32.3)	19567 (18088 to 21081)	759 (704.4 to 821.7)	1361 (916 to 1963)	51.5 (33.9 to 75)	20928 (19284 to 22720)	810.5 (746.2 to 884.6)
	Male	10 (9 to 12)	0.6 (0.5 to 0.7)	75 (64 to 89)	4.5 (3.9 to 5.3)	7 (6 to 8)	0.4 (0.4 to 0.5)	157 (134 to 180)	9.4 (8 to 10.7)	7 (5 to 10)	0.4 (0.3 to 0.6)	164 (139 to 189)	9.8 (8.4 to 11.2)	17 (12 to 22)	0.8 (0.5 to 1)	129 (98 to 169)	5.9 (4.5 to 7.6)	10 (8 to 12)	0.4 (0.3 to 0.5)	200 (163 to 242)	9.3 (7.6 to 11.1)	13 (8 to 19)	0.6 (0.4 to 0.9)	213 (173 to 258)	9.8 (8 to 11.8)
Uzbekistan	Both	1651 (1539 to 1769)	13.4 (12.5 to 14.3)	16116 (14609 to 17770)	133.9 (120.6 to 148.6)	914 (860 to 969)	7.8 (7.3 to 8.2)	29491 (27620 to 31344)	232.8 (218.6 to 247)	1129 (782 to 1545)	9.2 (6.4 to 12.6)	30620 (28699 to 32538)	242 (227.4 to 256.6)	5480 (4449 to 6676)	20.2 (16.6 to 24.2)	48418 (40270 to 58105)	182 (155.3 to 214.4)	2253 (1845 to 2704)	9.7 (8.2 to 11.5)	77488 (63018 to 93686)	275.4 (226.2 to 328.6)	3645 (2418 to 5257)	13.4 (9 to 19)	81133 (66329 to 98398)	288.8 (238.3 to 345.8)
	Female	1648 (1536 to 1765)	24.5 (22.9 to 26.2)	16077 (14569 to 17731)	240.8 (217.6 to 266.2)	913 (858 to 968)	13.8 (13 to 14.7)	29432 (27563 to 31286)	433 (406.6 to 459.9)	1126 (780 to 1541)	16.7 (11.6 to 22.9)	30558 (28640 to 32476)	449.7 (422 to 477.9)	5460 (4434 to 6653)	37.3 (30.6 to 44.8)	48253 (40106 to 57940)	334.9 (284.1 to 396)	2244 (1838 to 2693)	17.4 (14.6 to 20.6)	77193 (62744 to 93342)	511.2 (419.5 to 611.2)	3630 (2407 to 5235)	24.7 (16.6 to 35.3)	80823 (66069 to 98034)	536 (441.4 to 643.2)
	Male	3 (3 to 4)	0.1 (0 to 0.1)	39 (32 to 48)	0.7 (0.6 to 0.9)	2 (2 to 2)	0 (0 to 0)	59 (55 to 64)	0.9 (0.8 to 1)	3 (2 to 4)	0 (0 to 0.1)	62 (58 to 67)	1 (0.9 to 1.1)	20 (16 to 25)	0.2 (0.2 to 0.3)	165 (131 to 203)	1.6 (1.3 to 1.9)	10 (8 to 12)	0.1 (0.1 to 0.1)	295 (235 to 358)	2.8 (2.2 to 3.3)	15 (10 to 22)	0.2 (0.1 to 0.2)	310 (248 to 379)	2.9 (2.4 to 3.5)
Vanuatu	Both	8 (5 to 11)	9.9 (6.5 to 13.7)	63 (47 to 83)	81.1 (61.9 to 103.7)	5 (4 to 8)	7.7 (5 to 10.6)	189 (122 to 270)	223.9 (144.6 to 314.4)	5 (3 to 7)	6 (3.6 to 9.1)	193 (126 to 277)	229.9 (149.6 to 322.3)	37 (24 to 55)	18.6 (12.1 to 27.5)	264 (183 to 378)	130.1 (91.9 to 182.4)	24 (16 to 35)	13.2 (8.6 to 19)	804 (511 to 1213)	388.2 (252.4 to 579.5)	21 (12 to 34)	10.5 (6.1 to 16.4)	825 (523 to 1242)	398.7 (259.3 to 592.7)
	Female	8 (5 to 11)	21.4 (13.8 to 29.9)	63 (46 to 83)	177.1 (134.8 to 226)	5 (3 to 8)	16.8 (10.9 to 23.3)	187 (120 to 268)	482.9 (309 to 680.9)	5 (3 to 7)	13 (7.8 to 19.8)	191 (124 to 276)	495.9 (319.5 to 696)	36 (24 to 55)	38.2 (25 to 56.7)	262 (182 to 376)	267.7 (188.6 to 375.3)	24 (15 to 35)	27.2 (17.8 to 39.3)	797 (506 to 1205)	796 (514.9 to 1188.3)	21 (12 to 33)	21.6 (12.5 to 33.7)	819 (516 to 1235)	817.6 (527.9 to 1215.3)
	Male	0 (0 to 0)	0.2 (0.1 to 0.4)	1 (0 to 1)	1.4 (0.9 to 2.1)	0 (0 to 0)	0.2 (0.1 to 0.4)	2 (1 to 3)	4.9 (2.7 to 8.6)	0 (0 to 0)	0.1 (0.1 to 0.3)	2 (1 to 3)	5 (2.8 to 8.8)	0 (0 to 1)	0.3 (0.2 to 0.6)	2 (1 to 3)	1.9 (1.3 to 3)	0 (0 to 0)	0.3 (0.2 to 0.5)	7 (4 to 11)	6.9 (4 to 11.3)	0 (0 to 0)	0.2 (0.1 to 0.4)	7 (4 to 11)	7.1 (4.2 to 11.7)
Venezuela (Bolivarian Republic of)	Both	1685 (1583 to 1801)	15 (14.1 to 16)	13367 (12479 to 14363)	118.4 (110.4 to 127.2)	806 (764 to 846)	7.7 (7.3 to 8.1)	26028 (24720 to 27451)	220.5 (209.3 to 232.1)	984 (697 to 1324)	8.7 (6.2 to 11.7)	27012 (25687 to 28443)	229.2 (217.3 to 241.3)	8475 (6289 to 11298)	27.9 (20.8 to 37)	71150 (54208 to 93200)	233.4 (179.2 to 303.9)	2811 (2128 to 3671)	9.5 (7.2 to 12.4)	81498 (60603 to 109596)	265.3 (197.8 to 355.4)	5073 (3283 to 7511)	16.7 (10.8 to 24.6)	86571 (64614 to 116284)	282 (211.6 to 377.8)

		1990												2019											
		Incidence		Prevalence		Deaths		YLLs (Years of Life Lost)		YLDs (Years Lived with Disability)		DALYs (Disability-Adjusted Life Years)		Incidence		Prevalence		Deaths		YLLs (Years of Life Lost)		YLDs (Years Lived with Disability)		DALYs (Disability-Adjusted Life Years)	
Location	Sex	Number	Rate	Number	Rate	Number	Rate	Number	Rate	Number	Rate	Number	Rate	Number	Rate	Number	Rate	Number	Rate	Number	Rate	Number	Rate	Number	Rate
	Female	1661 (1560 to 1775)	28.4 (26.7 to 30.4)	13235 (12349 to 14217)	225.7 (210.5 to 242.8)	787 (747 to 829)	14.3 (13.5 to 15)	25597 (24280 to 26992)	419.1 (397.8 to 441.7)	970 (685 to 1309)	16.4 (11.7 to 22.1)	26567 (25239 to 27987)	435.5 (412.9 to 459)	8428 (6255 to 11241)	53 (39.4 to 70.5)	70847 (54004 to 92831)	445.2 (341.1 to 580.6)	2783 (2109 to 3641)	17.7 (13.4 to 23.1)	80863 (60097 to 108795)	506 (376.5 to 679.3)	5043 (3262 to 7464)	31.7 (20.5 to 46.9)	85906 (64107 to 115429)	537.7 (402.1 to 721.9)
	Male	24 (21 to 27)	0.5 (0.5 to 0.6)	132 (114 to 151)	2.8 (2.4 to 3.2)	18 (16 to 20)	0.4 (0.4 to 0.5)	431 (389 to 472)	9 (8.1 to 9.9)	14 (10 to 19)	0.3 (0.2 to 0.4)	445 (401 to 488)	9.3 (8.4 to 10.3)	46 (34 to 64)	0.3 (0.2 to 0.5)	303 (215 to 417)	2.2 (1.5 to 3)	27 (20 to 37)	0.2 (0.2 to 0.3)	635 (457 to 859)	4.6 (3.3 to 6.2)	30 (19 to 45)	0.2 (0.1 to 0.3)	665 (482 to 902)	4.8 (3.5 to 6.4)
Viet Nam	Both	6300 (4898 to 7874)	15.1 (11.8 to 18.7)	49666 (40005 to 60034)	118.4 (95.9 to 142.6)	4308 (3426 to 5297)	10.7 (8.6 to 13.1)	131958 (102470 to 166066)	309.6 (241.9 to 388)	3820 (2492 to 5380)	9.1 (5.9 to 12.6)	135778 (105984 to 171010)	318.7 (248.6 to 398.8)	27821 (20840 to 36275)	26.7 (20.2 to 34.4)	236079 (184934 to 302165)	225.2 (177.8 to 283.5)	11969 (9140 to 15527)	12.4 (9.4 to 16)	359492 (269517 to 468131)	339.1 (256.5 to 439.2)	17975 (11372 to 26612)	17.1 (11.1 to 25)	377467 (282930 to 491607)	356.2 (268.5 to 462)
	Female	6088 (4706 to 7640)	25.8 (20.1 to 32.3)	48643 (39065 to 58944)	206 (165.2 to 249.3)	4125 (3267 to 5098)	17.7 (14.1 to 21.9)	127977 (98682 to 161215)	539 (418.5 to 678.5)	3701 (2408 to 5242)	15.6 (10.1 to 22)	131678 (102226 to 165862)	554.6 (431.6 to 696.8)	27432 (20526 to 35854)	48.6 (36.6 to 63)	233624 (182907 to 298745)	413.6 (323.8 to 523.8)	11732 (8927 to 15223)	21.5 (16.4 to 27.8)	354024 (265202 to 461930)	622.1 (468.1 to 806.1)	17719 (11182 to 26176)	31.3 (20.1 to 46.1)	371742 (279096 to 485149)	653.4 (492.2 to 848.6)
	Male	212 (150 to 289)	1.5 (1 to 2.1)	1023 (734 to 1405)	6.2 (4.5 to 8.3)	183 (129 to 249)	1.4 (1 to 2)	3981 (2856 to 5400)	25.1 (18.1 to 33.9)	119 (74 to 178)	0.8 (0.5 to 1.2)	4100 (2940 to 5563)	25.9 (18.7 to 34.9)	389 (272 to 561)	1.1 (0.8 to 1.6)	2455 (1741 to 3592)	6.2 (4.4 to 9)	237 (168 to 334)	0.8 (0.5 to 1.1)	5469 (3880 to 7796)	13.9 (9.9 to 19.6)	256 (156 to 405)	0.7 (0.4 to 1.1)	5725 (4069 to 8203)	14.6 (10.4 to 20.6)
Yemen	Both	391 (242 to 637)	6.6 (4.1 to 10.9)	3248 (2343 to 4744)	57.8 (42.9 to 81.2)	271 (168 to 442)	4.9 (3 to 8.1)	9366 (5802 to 15040)	148.9 (93 to 243.5)	244 (141 to 410)	4.2 (2.4 to 7)	9609 (5967 to 15450)	153.1 (95.2 to 249.6)	2032 (1446 to 2836)	11.7 (8.6 to 16)	16022 (11761 to 21908)	92.3 (70.8 to 122.1)	1107 (810 to 1539)	7 (5.2 to 9.5)	39272 (27942 to 55702)	215.8 (157.2 to 302.2)	1251 (755 to 1946)	7.1 (4.4 to 10.8)	40523 (28761 to 57471)	222.9 (162.6 to 311.8)
	Female	378 (229 to 620)	12.6 (7.6 to 21)	3168 (2271 to 4651)	110 (80.1 to 158.4)	260 (157 to 428)	9.1 (5.4 to 15.2)	9050 (5495 to 14723)	289.6 (176.5 to 476.3)	235 (133 to 401)	7.9 (4.5 to 13.6)	9285 (5633 to 15062)	297.5 (181.5 to 490.7)	2001 (1418 to 2805)	22.7 (16.6 to 31.3)	15809 (11583 to 21685)	180.2 (137.6 to 239.9)	1085 (790 to 1506)	13.4 (10 to 18.4)	38639 (27428 to 54742)	420.3 (306.3 to 587.2)	1230 (742 to 1918)	13.8 (8.6 to 21.1)	39870 (28352 to 56502)	434.1 (314.8 to 605.8)
	Male	13 (6 to 23)	0.6 (0.3 to 1)	79 (45 to 131)	3.2 (1.9 to 5)	11 (5 to 19)	0.5 (0.3 to 0.9)	316 (158 to 571)	11.8 (6 to 21)	8 (4 to 15)	0.3 (0.2 to 0.6)	324 (163 to 584)	12.2 (6.2 to 21.6)	31 (19 to 50)	0.5 (0.3 to 0.7)	212 (142 to 327)	3 (2.1 to 4.5)	22 (14 to 35)	0.4 (0.2 to 0.6)	632 (389 to 1023)	8.4 (5.2 to 13.5)	21 (12 to 35)	0.3 (0.2 to 0.5)	653 (400 to 1054)	8.7 (5.4 to 14)
Zambia	Both	399 (316 to 503)	12.1 (9.8 to 14.9)	2468 (2003 to 3064)	71.1 (59.2 to 86.7)	313 (250 to 391)	10.4 (8.4 to 12.7)	10535 (8193 to 13420)	288.1 (228.2 to 361.6)	222 (147 to 313)	6.5 (4.4 to 9)	10756 (8348 to 13712)	294.5 (233 to 369.9)	1256 (915 to 1673)	15.6 (11.7 to 20.6)	8682 (6457 to 11335)	100.8 (77.8 to 130.3)	812 (600 to 1074)	11.6 (9 to 14.9)	26894 (19120 to 36160)	300.1 (219.7 to 398)	734 (454 to 1106)	8.6 (5.6 to 12.6)	27629 (19665 to 37182)	308.8 (226.8 to 410.4)
	Female	359 (278 to 457)	21.4 (17.1 to 26.8)	2286 (1831 to 2855)	131.4 (109 to 160.3)	275 (215 to 347)	17.9 (14.4 to 22.1)	9651 (7353 to 12507)	517.7 (405.3 to 654.1)	201 (131 to 285)	11.5 (7.7 to 16.3)	9851 (7509 to 12699)	529.2 (413.7 to 667.3)	1171 (843 to 1561)	27.3 (20.3 to 36.7)	8231 (6083 to 10742)	183.3 (139.1 to 237)	741 (543 to 988)	19.5 (14.8 to 25.7)	25174 (17615 to 33964)	538.1 (390.3 to 721.4)	686 (427 to 1037)	15.3 (9.8 to 22.7)	25860 (18147 to 34863)	553.4 (400.8 to 743.5)
	Male	40 (27 to 60)	3.2 (2 to 4.6)	182 (127 to 263)	13.1 (9.3 to 18.6)	38 (25 to 58)	3.3 (2.1 to 4.8)	884 (580 to 1318)	61.9 (40.5 to 93.2)	21 (12 to 35)	1.5 (0.8 to 2.5)	905 (594 to 1350)	63.5 (41.5 to 95.3)	85 (51 to 133)	3.2 (1.9 to 5.1)	451 (285 to 693)	15.1 (9.7 to 23)	71 (42 to 113)	2.9 (1.8 to 4.7)	1720 (1030 to 2653)	55.9 (33.2 to 88.4)	49 (26 to 81)	1.7 (0.9 to 2.8)	1769 (1063 to 2736)	57.6 (34.4 to 90.8)
Zimbabwe	Both	591 (484 to 706)	13.1 (10.8 to 15.6)	4201 (3516 to 4910)	91.4 (77.6 to 106.3)	394 (324 to 471)	9.6 (8 to 11.4)	12356 (10039 to 15044)	255.5 (209.3 to 308)	345 (231 to 477)	7.4 (5.1 to 10.2)	12700 (10352 to 15459)	262.9 (215.5 to 316.1)	1754 (1203 to 2432)	21.6 (15.2 to 29.6)	11886 (8599 to 16025)	142.5 (105.4 to 187.5)	1161 (803 to 1614)	15.8 (11.1 to 21.8)	37527 (25457 to 53306)	432.1 (296.3 to 604.7)	993 (618 to 1513)	11.9 (7.4 to 17.7)	38520 (26126 to 54815)	444 (304.2 to 622.1)
	Female	583 (476 to 698)	24.8 (20.4 to 29.4)	4151 (3472 to 4855)	174 (146.5 to 203.3)	388 (318 to 466)	17.7 (14.7 to 21.1)	12179 (9883 to 14859)	489.5 (398 to 592.7)	340 (227 to 471)	14.1 (9.6 to 19.5)	12518 (10192 to 15266)	503.6 (409.5 to 609.4)	1740 (1191 to 2413)	37.6 (26.3 to 51.7)	11794 (8521 to 15933)	249.3 (182.8 to 330.7)	1150 (793 to 1600)	26.9 (18.7 to 37.2)	37161 (25154 to 52942)	761.5 (520.2 to 1074.1)	983 (611 to 1497)	20.8 (12.9 to 31)	38145 (25829 to 54398)	782.3 (533.1 to 1101.4)
	Male	8 (5 to 10)	0.4 (0.3 to 0.5)	50 (38 to 64)	2.4 (1.9 to 3)	6 (4 to 8)	0.3 (0.2 to 0.4)	177 (128 to 236)	7.6 (5.5 to 10.1)	5 (3 to 8)	0.2 (0.1 to 0.4)	182 (131 to 242)	7.9 (5.7 to 10.4)	15 (10 to 21)	0.4 (0.3 to 0.6)	92 (64 to 128)	2.7 (2 to 3.5)	11 (8 to 16)	0.4 (0.3 to 0.5)	365 (243 to 538)	9.7 (6.6 to 13.7)	9 (6 to 15)	0.3 (0.2 to 0.4)	375 (249 to 551)	10 (6.8 to 14.1)

Supplementary table 2. The regional, socio-demographic index (SDI) quintiles, and national trend of age-standardized rates of epidemiologic indices attributed to various breast cancer risk factors for females, in 1990 and 2019.

Location	Risk factor	1990				2019			
		Deaths	YLLs (Years of Life Lost)	YLDs (Years Lived with Disability)	DALYs (Disability-Adjusted Life Years)	Deaths	YLLs (Years of Life Lost)	YLDs (Years Lived with Disability)	DALYs (Disability-Adjusted Life Years)
WHO regions									
African Region	All risk factors	2.6 (1.7 to 3.7)	64.5 (43.6 to 89.1)	1.7 (1.1 to 2.6)	66.2 (44.8 to 91.7)	3.8 (2.5 to 5.5)	87.3 (55.8 to 126)	2.8 (1.7 to 4.4)	90.1 (57.7 to 129.7)
African Region	Behavioral risks	1.7 (1.2 to 2.1)	47.4 (35.6 to 59.7)	1.2 (0.8 to 1.7)	48.6 (36.4 to 61.4)	2 (1.5 to 2.5)	54.5 (40.9 to 69.5)	1.7 (1.1 to 2.4)	56.2 (42 to 71.5)
African Region	Alcohol use	0.8 (0.6 to 1)	23.1 (17.5 to 29)	0.5 (0.4 to 0.8)	23.6 (17.9 to 29.7)	1 (0.7 to 1.2)	28.1 (21.1 to 35.9)	0.8 (0.5 to 1.2)	29 (21.6 to 36.9)
African Region	Diet high in red meat	0.3 (0.1 to 0.4)	7.7 (2.2 to 10.8)	0.2 (0.1 to 0.3)	7.9 (2.3 to 11.1)	0.3 (0.1 to 0.5)	9.5 (2.6 to 13.5)	0.3 (0.1 to 0.5)	9.8 (2.7 to 13.9)
African Region	Low physical activity	0.2 (0.1 to 0.3)	3.6 (2 to 6.3)	0.1 (0 to 0.2)	3.7 (2 to 6.4)	0.2 (0.1 to 0.4)	4.5 (2.4 to 7.9)	0.2 (0.1 to 0.3)	4.7 (2.5 to 8.1)
African Region	Smoking	0.2 (0.1 to 0.3)	5.1 (3 to 7.4)	0.1 (0.1 to 0.2)	5.3 (3.1 to 7.6)	0.2 (0.1 to 0.3)	4.3 (2.4 to 6.4)	0.1 (0.1 to 0.2)	4.5 (2.5 to 6.6)
African Region	Secondhand smoke	0.3 (0.1 to 0.6)	9.9 (2.4 to 17.4)	0.2 (0.1 to 0.5)	10.2 (2.4 to 17.9)	0.3 (0.1 to 0.6)	10.1 (2.4 to 17.7)	0.3 (0.1 to 0.6)	10.4 (2.4 to 18.4)
African Region	Metabolic risks	1.1 (0.3 to 2.2)	19.1 (2.2 to 42.8)	0.6 (0.1 to 1.3)	19.7 (2.4 to 43.8)	2 (0.7 to 3.8)	36.5 (7.8 to 77)	1.3 (0.3 to 2.7)	37.8 (8.2 to 79.9)
African Region	High body-mass index	0.3 (0 to 0.8)	3.7 (-4.6 to 13.1)	0.2 (-0.1 to 0.4)	3.8 (-4.6 to 13.6)	0.8 (0.1 to 1.6)	10.5 (-4.9 to 28)	0.4 (-0.1 to 1)	10.9 (-5 to 28.8)
African Region	High fasting plasma glucose	0.7 (0.1 to 1.7)	16 (2.9 to 36.8)	0.4 (0.1 to 1.1)	16.4 (3 to 37.8)	1.3 (0.2 to 2.9)	27.6 (5.1 to 63)	0.9 (0.2 to 2.2)	28.5 (5.2 to 65.3)
Eastern Mediterranean Region	All risk factors	2.6 (1.4 to 4.3)	66.3 (35.4 to 109.7)	2 (1 to 3.4)	68.3 (36.5 to 112.8)	4.6 (2.5 to 7.5)	110.9 (57 to 186.9)	4.6 (2.2 to 8)	115.5 (59.3 to 193.7)
Eastern Mediterranean Region	Behavioral risks	1.5 (0.9 to 2.2)	43.8 (25.4 to 64.1)	1.2 (0.7 to 2)	45 (26.2 to 66.1)	1.9 (1.2 to 2.6)	55.7 (34.1 to 77.7)	2.3 (1.3 to 3.5)	58 (35.6 to 80.5)
Eastern Mediterranean Region	Alcohol use	0 (0 to 0.1)	1.5 (1.1 to 2.1)	0 (0 to 0.1)	1.6 (1.1 to 2.2)	0.1 (0 to 0.1)	2.2 (1.6 to 3)	0.1 (0.1 to 0.1)	2.3 (1.7 to 3.1)
Eastern Mediterranean Region	Diet high in red meat	0.3 (0.1 to 0.5)	10.6 (2.3 to 16.1)	0.3 (0.1 to 0.5)	10.9 (2.4 to 16.6)	0.5 (0.1 to 0.7)	14.5 (3.3 to 21.2)	0.6 (0.1 to 0.9)	15.1 (3.4 to 22.1)
Eastern Mediterranean Region	Low physical activity	0.3 (0.1 to 0.5)	6.6 (3 to 11.6)	0.2 (0.1 to 0.4)	6.8 (3.1 to 12)	0.4 (0.2 to 0.7)	9.8 (4.4 to 16.7)	0.4 (0.2 to 0.8)	10.2 (4.6 to 17.5)
Eastern Mediterranean Region	Smoking	0.3 (0.1 to 0.4)	6.9 (3.9 to 10.8)	0.2 (0.1 to 0.3)	7.1 (4 to 11.1)	0.3 (0.2 to 0.4)	7.7 (4.8 to 11)	0.3 (0.2 to 0.5)	8 (5 to 11.4)
Eastern Mediterranean Region	Secondhand smoke	0.6 (0.1 to 1.1)	19.4 (4.7 to 34.9)	0.5 (0.1 to 1)	19.9 (4.9 to 35.7)	0.7 (0.2 to 1.3)	23.1 (5.6 to 41.4)	0.9 (0.2 to 1.7)	24 (5.8 to 42.9)
Eastern Mediterranean Region	Metabolic risks	1.2 (0.3 to 2.7)	24.6 (0.5 to 61.1)	0.8 (0.1 to 2)	25.4 (0.5 to 63.1)	2.9 (0.9 to 5.8)	60.1 (9 to 133.5)	2.5 (0.4 to 5.8)	62.6 (9.4 to 139.9)
Eastern Mediterranean Region	High body-mass index	0.3 (-0.1 to 0.9)	2 (-9.5 to 14.1)	0.1 (-0.2 to 0.5)	2.2 (-9.7 to 14.6)	0.8 (0 to 1.8)	7.9 (-15.2 to 30)	0.4 (-0.7 to 1.4)	8.3 (-15.8 to 31.4)
Eastern Mediterranean Region	High fasting plasma glucose	0.9 (0.2 to 2.3)	23.2 (4 to 57.6)	0.7 (0.1 to 1.8)	23.9 (4.1 to 59.4)	2.2 (0.4 to 5.1)	54.8 (10.8 to 127.4)	2.3 (0.4 to 5.5)	57.1 (11.3 to 132.1)
European Region	All risk factors	7.3 (5.8 to 8.8)	193.4 (155.7 to 228.8)	13.2 (8.8 to 18.3)	206.7 (166.4 to 244.5)	6 (4.6 to 7.8)	144.1 (111.2 to 181.1)	15.5 (9.9 to 22.2)	159.6 (123.4 to 200.4)
European Region	Behavioral risks	5.6 (4.8 to 6.4)	163.7 (140.3 to 187)	11 (7.6 to 15.3)	174.8 (149.3 to 198.8)	4.1 (3.4 to 4.8)	110 (92.8 to 128.1)	11.8 (7.8 to 16.8)	121.8 (101.8 to 142.2)
European Region	Alcohol use	2.8 (2.3 to 3.3)	82.5 (68.3 to 97.6)	5.6 (3.7 to 8)	88.2 (72.8 to 104.3)	2 (1.6 to 2.4)	54.5 (44.5 to 65.7)	5.9 (3.9 to 8.5)	60.4 (49.2 to 73)
European Region	Diet high in red meat	1.1 (0.6 to 1.5)	32.8 (16.3 to 43.5)	2.2 (1 to 3.4)	35 (17.4 to 46.5)	0.8 (0.4 to 1.1)	22.2 (10.7 to 29.9)	2.4 (1 to 3.8)	24.6 (11.7 to 33.1)
European Region	Low physical activity	0.3 (0.1 to 0.5)	6.8 (3.1 to 12.6)	0.5 (0.2 to 1.1)	7.3 (3.4 to 13.5)	0.3 (0.1 to 0.5)	5.6 (2.5 to 10)	0.6 (0.3 to 1.3)	6.2 (2.7 to 11.2)
European Region	Smoking	1.4 (1 to 1.8)	41 (29.3 to 53.2)	2.8 (1.7 to 4.3)	43.8 (31.3 to 57.1)	1 (0.7 to 1.3)	27.1 (19.3 to 35.2)	3 (1.8 to 4.6)	30.1 (21.4 to 39.4)
European Region	Secondhand smoke	0.5 (0.1 to 0.9)	17.6 (4.3 to 30.3)	1.1 (0.2 to 1.9)	18.7 (4.5 to 32.1)	0.4 (0.1 to 0.6)	11 (2.7 to 18.8)	1 (0.2 to 1.9)	12 (3 to 20.7)
European Region	Metabolic risks	2.1 (0.8 to 3.9)	38.7 (6.8 to 78.9)	2.9 (0.7 to 5.8)	41.6 (7.4 to 84.1)	2.4 (1 to 4.4)	43.9 (10.7 to 85.6)	4.7 (1.3 to 9.6)	48.6 (12.1 to 93.6)
European Region	High body-mass index	1 (0.2 to 2.1)	14.2 (-6.6 to 36.8)	1.1 (-0.2 to 2.6)	15.3 (-6.7 to 39.5)	1.1 (0.3 to 2.3)	16.8 (-3 to 40.1)	1.8 (-0.1 to 4.1)	18.6 (-3 to 44.2)
European Region	High fasting plasma glucose	1.2 (0.2 to 2.7)	26 (4.9 to 58.8)	1.9 (0.3 to 4.6)	27.8 (5.2 to 63)	1.4 (0.3 to 3.1)	29.2 (5.7 to 65.2)	3.2 (0.6 to 7.7)	32.4 (6.3 to 72.3)
Region of the Americas	All risk factors	7.1 (5.5 to 8.9)	183.4 (143.6 to 226.3)	16.7 (11.1 to 23.3)	200.1 (156.8 to 245.9)	5.2 (3.8 to 7)	124.1 (91.7 to 163.9)	14.1 (8.9 to 20.8)	138.2 (101 to 180.7)
Region of the Americas	Behavioral risks	5.1 (4.3 to 5.9)	145.5 (123.1 to 164.9)	12.6 (8.7 to 17.4)	158.1 (134.5 to 180)	3.3 (2.7 to 3.8)	88.4 (74.3 to 102)	9.5 (6.4 to 13.4)	97.9 (82 to 113)
Region of the Americas	Alcohol use	2 (1.7 to 2.4)	60.2 (49.8 to 71.6)	5.2 (3.5 to 7.3)	65.4 (54 to 77.7)	1.4 (1.1 to 1.7)	39.7 (32.4 to 47.5)	4.4 (2.9 to 6.3)	44.1 (35.9 to 52.5)
Region of the Americas	Diet high in red meat	1.1 (0.5 to 1.5)	30.7 (15.4 to 40.7)	2.7 (1.3 to 4.1)	33.3 (16.7 to 44.7)	0.8 (0.4 to 1.1)	21.6 (11.1 to 29.2)	2.2 (1 to 3.5)	23.8 (12 to 32.3)
Region of the Americas	Low physical activity	0.3 (0.1 to 0.6)	7.5 (3.1 to 14.6)	0.7 (0.3 to 1.5)	8.2 (3.3 to 15.8)	0.2 (0.1 to 0.4)	5.6 (2.3 to 10.2)	0.6 (0.2 to 1.2)	6.2 (2.5 to 11.3)

Location	Risk factor	1990				2019			
		Deaths	YLLs (Years of Life Lost)	YLDs (Years Lived with Disability)	DALYs (Disability-Adjusted Life Years)	Deaths	YLLs (Years of Life Lost)	YLDs (Years Lived with Disability)	DALYs (Disability-Adjusted Life Years)
Region of the Americas	Smoking	1.8 (1.3 to 2.3)	49.1 (35.6 to 63.4)	4.4 (2.7 to 6.6)	53.5 (38.8 to 69.4)	0.9 (0.6 to 1.1)	21.8 (15.4 to 28.8)	2.5 (1.5 to 3.8)	24.4 (17.3 to 32.1)
Region of the Americas	Secondhand smoke	0.4 (0.1 to 0.7)	12.5 (3 to 21.7)	0.9 (0.2 to 1.6)	13.3 (3.1 to 23.3)	0.2 (0.1 to 0.4)	7.1 (1.8 to 12.4)	0.6 (0.1 to 1.1)	7.7 (1.9 to 13.5)
Region of the Americas	Metabolic risks	2.5 (0.9 to 4.7)	48.7 (10.4 to 97.8)	5.2 (1.6 to 10.3)	54 (12.3 to 107.1)	2.4 (0.9 to 4.4)	44.7 (9.6 to 88.6)	5.8 (1.8 to 11.3)	50.5 (11.4 to 98.6)
Region of the Americas	High body-mass index	1 (0.2 to 2.2)	15.1 (-5.3 to 38.8)	2 (0.1 to 4.4)	17.2 (-5.3 to 43.3)	1 (0.2 to 2)	13 (-5.6 to 33.2)	2.2 (0.1 to 5)	15.3 (-5.6 to 38.3)
Region of the Americas	High fasting plasma glucose	1.6 (0.3 to 3.5)	35.7 (6.8 to 79.7)	3.4 (0.6 to 8.1)	39.1 (7.4 to 86.8)	1.6 (0.3 to 3.4)	34.1 (6.6 to 75)	3.9 (0.8 to 9)	38 (7.4 to 83.8)
South-East Asia Region	All risk factors	1.7 (1 to 2.6)	46.7 (26.5 to 71.6)	1.3 (0.7 to 2.1)	47.9 (27.4 to 73.2)	2.6 (1.5 to 4.1)	68.4 (39.8 to 106.3)	2.6 (1.4 to 4.3)	71.1 (41.4 to 109.9)
South-East Asia Region	Behavioral risks	0.9 (0.5 to 1.3)	27.4 (15.2 to 39.6)	0.7 (0.4 to 1.1)	28.1 (15.6 to 40.7)	1 (0.6 to 1.3)	28.4 (16.3 to 40.3)	1 (0.6 to 1.6)	29.5 (16.9 to 41.7)
South-East Asia Region	Alcohol use	0.1 (0.1 to 0.1)	2.3 (1.6 to 3)	0.1 (0 to 0.1)	2.3 (1.7 to 3.1)	0.1 (0.1 to 0.2)	3.8 (2.6 to 5.2)	0.2 (0.1 to 0.2)	4 (2.8 to 5.4)
South-East Asia Region	Diet high in red meat	0.1 (0 to 0.1)	3.1 (1.1 to 4.2)	0.1 (0 to 0.1)	3.1 (1.1 to 4.3)	0.1 (0 to 0.2)	3.6 (1.3 to 5.1)	0.1 (0 to 0.2)	3.7 (1.3 to 5.3)
South-East Asia Region	Low physical activity	0.1 (0.1 to 0.2)	3.2 (1.7 to 5.7)	0.1 (0 to 0.2)	3.3 (1.8 to 5.9)	0.1 (0.1 to 0.3)	3.3 (1.8 to 5.7)	0.1 (0.1 to 0.2)	3.4 (1.9 to 6)
South-East Asia Region	Smoking	0.2 (0.1 to 0.2)	4.1 (2.5 to 6)	0.1 (0.1 to 0.2)	4.2 (2.5 to 6.2)	0.1 (0.1 to 0.2)	3.2 (1.9 to 4.8)	0.1 (0.1 to 0.2)	3.3 (2 to 4.9)
South-East Asia Region	Secondhand smoke	0.5 (0.1 to 0.8)	15.3 (3.6 to 27.1)	0.4 (0.1 to 0.7)	15.7 (3.7 to 27.8)	0.5 (0.1 to 0.8)	15.1 (3.7 to 26.2)	0.5 (0.1 to 1)	15.6 (3.8 to 27)
South-East Asia Region	Metabolic risks	0.8 (0.3 to 1.7)	20.9 (6.3 to 43.3)	0.6 (0.2 to 1.2)	21.5 (6.5 to 44.4)	1.8 (0.7 to 3.3)	43 (16.9 to 80.2)	1.7 (0.7 to 3.2)	44.7 (17.8 to 84.2)
South-East Asia Region	High body-mass index	0.3 (0.1 to 0.6)	6.1 (1.1 to 15.1)	0.2 (0 to 0.5)	6.3 (1.2 to 15.5)	0.8 (0.3 to 1.4)	17.9 (6.2 to 33.6)	0.8 (0.3 to 1.4)	18.7 (6.5 to 34.6)
South-East Asia Region	High fasting plasma glucose	0.6 (0.1 to 1.4)	15.1 (2.7 to 35.5)	0.4 (0.1 to 1)	15.6 (2.8 to 36.5)	1.1 (0.2 to 2.5)	26.7 (5 to 61.9)	1 (0.2 to 2.4)	27.7 (5.2 to 64.2)
Western Pacific Region	All risk factors	2.2 (1.5 to 3.1)	62 (42 to 85.5)	3.4 (2.1 to 4.9)	65.4 (44.6 to 90.2)	2.9 (1.8 to 4)	76.3 (50.2 to 105.9)	7.2 (4.3 to 10.7)	83.4 (55.2 to 114.8)
Western Pacific Region	Behavioral risks	1.4 (1 to 1.7)	40.8 (28.7 to 52.1)	2.4 (1.5 to 3.4)	43.1 (30.5 to 55)	1.4 (1 to 1.8)	40.5 (29.2 to 52)	4 (2.5 to 5.7)	44.5 (32.1 to 56.5)
Western Pacific Region	Alcohol use	0.4 (0.3 to 0.5)	13.3 (10.7 to 16)	1 (0.6 to 1.4)	14.3 (11.4 to 17.3)	0.4 (0.3 to 0.5)	12.1 (9.4 to 14.9)	1.3 (0.8 to 1.9)	13.4 (10.4 to 16.6)
Western Pacific Region	Diet high in red meat	0.3 (0.1 to 0.4)	9.6 (3.5 to 13.4)	0.5 (0.2 to 0.8)	10.1 (3.7 to 13.9)	0.4 (0.2 to 0.6)	13 (5.9 to 18.3)	1.2 (0.5 to 2)	14.2 (6.5 to 19.9)
Western Pacific Region	Low physical activity	0.1 (0 to 0.2)	2.2 (1.2 to 4.1)	0.1 (0.1 to 0.3)	2.4 (1.2 to 4.4)	0.1 (0 to 0.2)	2.2 (1.2 to 4.1)	0.2 (0.1 to 0.5)	2.4 (1.3 to 4.5)
Western Pacific Region	Smoking	0.2 (0.1 to 0.2)	4.7 (3.1 to 6.4)	0.4 (0.2 to 0.5)	5.1 (3.4 to 6.8)	0.2 (0.1 to 0.2)	3.9 (2.6 to 5.3)	0.4 (0.2 to 0.7)	4.3 (2.9 to 5.9)
Western Pacific Region	Secondhand smoke	0.4 (0.1 to 0.7)	13.2 (3.2 to 22.7)	0.6 (0.1 to 1.1)	13.8 (3.3 to 23.7)	0.4 (0.1 to 0.6)	11.5 (2.9 to 19.9)	1 (0.2 to 1.8)	12.5 (3.1 to 21.7)
Western Pacific Region	Metabolic risks	1 (0.3 to 1.9)	24.2 (8.2 to 46.9)	1.2 (0.4 to 2.4)	25.4 (8.6 to 49.2)	1.7 (0.7 to 2.9)	41 (16.5 to 71.6)	3.7 (1.4 to 6.9)	44.6 (18.1 to 77.8)
Western Pacific Region	High body-mass index	0.5 (0.1 to 1.2)	12.9 (3 to 29.6)	0.6 (0.1 to 1.4)	13.5 (3.2 to 30.9)	1.1 (0.4 to 2.2)	28 (8.8 to 54.9)	2.4 (0.7 to 5)	30.4 (9.5 to 59.3)
Western Pacific Region	High fasting plasma glucose	0.5 (0.1 to 1.1)	12 (2.3 to 27.8)	0.6 (0.1 to 1.6)	12.7 (2.4 to 29.2)	0.6 (0.1 to 1.4)	14.8 (2.7 to 34.2)	1.4 (0.2 to 3.4)	16.2 (3 to 37.2)
SDI Quintiles									
High SDI	All risk factors	7.8 (6.3 to 9.4)	205.3 (167.3 to 243.5)	18.7 (12.5 to 25.8)	224 (183 to 264.4)	5.6 (4.3 to 7)	133.5 (104.8 to 166)	18.9 (12.2 to 27.2)	152.4 (118.2 to 189)
High SDI	Behavioral risks	6.1 (5.2 to 6.9)	172.5 (148.4 to 194.2)	15.2 (10.4 to 21)	187.7 (161.3 to 211.7)	3.8 (3.2 to 4.4)	101.5 (86.7 to 115.7)	14.1 (9.4 to 19.7)	115.6 (98.1 to 132.4)
High SDI	Alcohol use	3 (2.5 to 3.6)	86.9 (71.8 to 102.8)	7.5 (5 to 10.6)	94.4 (78.2 to 111.8)	2 (1.6 to 2.4)	53.5 (43.6 to 63.5)	7.4 (4.8 to 10.7)	61 (49.6 to 72.4)
High SDI	Diet high in red meat	1.2 (0.6 to 1.6)	32.7 (16.8 to 43.6)	2.9 (1.4 to 4.6)	35.6 (18.2 to 47.6)	0.8 (0.4 to 1)	20.6 (10.3 to 27.6)	2.8 (1.3 to 4.6)	23.4 (11.4 to 31.8)
High SDI	Low physical activity	0.3 (0.1 to 0.6)	7.4 (2.9 to 14.6)	0.7 (0.3 to 1.6)	8.1 (3.1 to 16.1)	0.2 (0.1 to 0.5)	5.4 (2 to 10.2)	0.7 (0.3 to 1.6)	6.1 (2.3 to 11.7)
High SDI	Smoking	1.8 (1.3 to 2.3)	51.3 (37.3 to 65.8)	4.6 (2.8 to 6.9)	55.9 (40.9 to 71.7)	0.9 (0.7 to 1.2)	24.6 (17.8 to 31.7)	3.5 (2 to 5.2)	28.1 (20.1 to 36.5)
High SDI	Secondhand smoke	0.4 (0.1 to 0.7)	13.2 (3.2 to 22.9)	1.1 (0.2 to 2)	14.3 (3.4 to 24.7)	0.2 (0.1 to 0.4)	7.1 (1.7 to 12.2)	0.9 (0.2 to 1.7)	8 (1.9 to 13.8)
High SDI	Metabolic risks	2.3 (0.9 to 4.3)	43.9 (10.3 to 87.4)	4.6 (1.3 to 9.2)	48.5 (11.7 to 95.2)	2.2 (0.9 to 4)	41.8 (11.8 to 79.8)	6.3 (2 to 12.4)	48.1 (14.2 to 91.6)
High SDI	High body-mass index	1 (0.2 to 2.1)	15.7 (-3.4 to 38.1)	1.8 (0 to 3.9)	17.5 (-3.6 to 42.4)	1 (0.3 to 1.9)	14.8 (-1 to 33.6)	2.4 (0.2 to 5.3)	17.2 (-0.8 to 38.8)
High SDI	High fasting plasma glucose	1.4 (0.3 to 3)	29.9 (5.7 to 67.5)	3 (0.6 to 7.3)	33 (6.2 to 73.9)	1.4 (0.3 to 3)	29.1 (5.7 to 63.4)	4.2 (0.8 to 10)	33.4 (6.5 to 73.3)
High-middle SDI	All risk factors	4.6 (3.6 to 5.8)	124.6 (97 to 154.1)	7 (4.6 to 9.8)	131.6 (102.8 to 162.4)	4.2 (3.1 to 5.6)	104.7 (77.2 to 137.6)	9.2 (5.9 to 13.3)	113.9 (84.2 to 148.7)
High-middle SDI	Behavioral risks	3.2 (2.7 to 3.8)	96.2 (79.1 to 112.2)	5.3 (3.6 to 7.4)	101.5 (83.3 to 118.2)	2.4 (2 to 2.9)	67.2 (54.1 to 80.4)	5.9 (3.9 to 8.3)	73.1 (58.6 to 87.3)
High-middle SDI	Alcohol use	1.3 (1.1 to 1.6)	40.7 (33.5 to 48.5)	2.3 (1.6 to 3.3)	43.1 (35.3 to 51)	0.9 (0.7 to 1.1)	25.3 (20.5 to 31.1)	2.2 (1.4 to 3.2)	27.5 (22.3 to 33.9)

Location	Risk factor	1990				2019			
		Deaths	YLLs (Years of Life Lost)	YLDs (Years Lived with Disability)	DALYs (Disability-Adjusted Life Years)	Deaths	YLLs (Years of Life Lost)	YLDs (Years Lived with Disability)	DALYs (Disability-Adjusted Life Years)
High-middle SDI	Diet high in red meat	0.7 (0.4 to 1)	21.7 (10.5 to 28.8)	1.2 (0.5 to 1.9)	22.9 (11.1 to 30.4)	0.6 (0.3 to 0.8)	16.8 (8.4 to 23)	1.5 (0.7 to 2.4)	18.4 (9.1 to 24.9)
High-middle SDI	Low physical activity	0.2 (0.1 to 0.3)	4.8 (2.4 to 8.3)	0.3 (0.1 to 0.6)	5 (2.5 to 8.7)	0.2 (0.1 to 0.3)	4.1 (2 to 7)	0.4 (0.2 to 0.7)	4.5 (2.2 to 7.7)
High-middle SDI	Smoking	0.7 (0.5 to 0.9)	20.7 (14.2 to 27.5)	1.2 (0.7 to 1.8)	21.8 (15.1 to 29.1)	0.5 (0.4 to 0.7)	14.1 (9.8 to 18.6)	1.3 (0.7 to 1.9)	15.4 (10.7 to 20.4)
High-middle SDI	Secondhand smoke	0.5 (0.1 to 0.9)	16.6 (4 to 28.4)	0.8 (0.2 to 1.5)	17.4 (4.2 to 29.7)	0.4 (0.1 to 0.7)	11.9 (2.9 to 20.6)	1 (0.2 to 1.8)	12.9 (3.2 to 22.4)
High-middle SDI	Metabolic risks	1.7 (0.7 to 3)	34.3 (11.4 to 65.5)	2 (0.7 to 3.8)	36.3 (12.1 to 68.7)	2.1 (0.9 to 3.7)	44.4 (17.9 to 78.5)	3.9 (1.6 to 7.2)	48.3 (19.7 to 84.6)
High-middle SDI	High body-mass index	0.8 (0.3 to 1.7)	15.1 (1.4 to 33.4)	0.9 (0.1 to 2)	16 (1.5 to 35.2)	1.2 (0.5 to 2.2)	23.8 (8.2 to 45.4)	2.1 (0.7 to 4.1)	25.9 (9 to 48.9)
High-middle SDI	High fasting plasma glucose	0.9 (0.2 to 2)	20.4 (3.8 to 46.7)	1.2 (0.2 to 2.9)	21.6 (4 to 49.1)	1 (0.2 to 2.3)	22.7 (4.4 to 50.7)	2 (0.4 to 4.8)	24.7 (4.7 to 55.3)
Low SDI	All risk factors	1.9 (1.2 to 2.8)	50.9 (32.7 to 74.7)	1.2 (0.7 to 1.9)	52.1 (33.7 to 76.3)	2.9 (1.8 to 4.5)	73.4 (45.4 to 112.3)	2.1 (1.2 to 3.4)	75.5 (46.8 to 115.8)
Low SDI	Behavioral risks	1.2 (0.8 to 1.6)	35.4 (25 to 46.3)	0.8 (0.5 to 1.2)	36.2 (25.6 to 47.4)	1.5 (1.1 to 1.9)	43.3 (32 to 55.3)	1.2 (0.8 to 1.7)	44.5 (32.8 to 56.9)
Low SDI	Alcohol use	0.4 (0.3 to 0.5)	12.9 (9.4 to 16.5)	0.3 (0.2 to 0.4)	13.2 (9.6 to 16.9)	0.5 (0.4 to 0.7)	16.2 (11.9 to 20.8)	0.4 (0.3 to 0.6)	16.6 (12.2 to 21.3)
Low SDI	Diet high in red meat	0.2 (0.1 to 0.3)	6 (1.6 to 8.6)	0.1 (0 to 0.2)	6.1 (1.7 to 8.8)	0.3 (0.1 to 0.4)	7.7 (2.1 to 10.8)	0.2 (0.1 to 0.3)	7.9 (2.2 to 11.1)
Low SDI	Low physical activity	0.1 (0.1 to 0.2)	3.2 (1.7 to 5.5)	0.1 (0 to 0.2)	3.2 (1.8 to 5.7)	0.2 (0.1 to 0.3)	3.9 (2.2 to 6.7)	0.1 (0.1 to 0.2)	4 (2.3 to 6.9)
Low SDI	Smoking	0.2 (0.1 to 0.3)	4.4 (2.4 to 6.7)	0.1 (0.1 to 0.2)	4.6 (2.5 to 6.8)	0.2 (0.1 to 0.3)	5.4 (3 to 7.8)	0.2 (0.1 to 0.3)	5.6 (3.1 to 8.1)
Low SDI	Secondhand smoke	0.3 (0.1 to 0.6)	10 (2.4 to 17.6)	0.2 (0.1 to 0.4)	10.2 (2.5 to 18)	0.4 (0.1 to 0.6)	11.5 (2.6 to 20.3)	0.3 (0.1 to 0.6)	11.8 (2.7 to 20.9)
Low SDI	Metabolic risks	0.8 (0.2 to 1.6)	17 (3.6 to 38.8)	0.5 (0.1 to 1)	17.5 (3.7 to 39.6)	1.5 (0.5 to 3.1)	32.8 (7.3 to 72.2)	1 (0.3 to 2.2)	33.8 (7.6 to 73.8)
Low SDI	High body-mass index	0.2 (0 to 0.4)	2.5 (-1.6 to 7.9)	0.1 (0 to 0.2)	2.5 (-1.6 to 8.1)	0.4 (0 to 0.9)	5.3 (-3.3 to 15.9)	0.2 (0 to 0.5)	5.5 (-3.3 to 16.4)
Low SDI	High fasting plasma glucose	0.6 (0.1 to 1.4)	14.8 (2.6 to 35.3)	0.4 (0.1 to 0.9)	15.2 (2.7 to 36.4)	1.2 (0.2 to 2.7)	28.4 (5.4 to 66.2)	0.8 (0.2 to 2)	29.2 (5.6 to 67.9)
Low-middle SDI	All risk factors	2 (1.2 to 2.9)	53.4 (32.5 to 79)	1.5 (0.9 to 2.4)	54.8 (33.4 to 81)	2.9 (1.8 to 4.5)	75.1 (44.8 to 114.9)	2.9 (1.6 to 4.6)	77.9 (46.5 to 119.5)
Low-middle SDI	Behavioral risks	1.2 (0.8 to 1.6)	34.8 (22.5 to 46.7)	0.9 (0.6 to 1.4)	35.7 (23.2 to 48)	1.4 (1 to 1.8)	39.8 (27.5 to 52.2)	1.5 (0.9 to 2.1)	41.3 (28.6 to 54.3)
Low-middle SDI	Alcohol use	0.2 (0.1 to 0.2)	5.4 (4.2 to 6.7)	0.1 (0.1 to 0.2)	5.6 (4.3 to 6.9)	0.3 (0.2 to 0.3)	8.5 (6.6 to 10.6)	0.3 (0.2 to 0.4)	8.8 (6.8 to 10.9)
Low-middle SDI	Diet high in red meat	0.2 (0.1 to 0.3)	5.9 (2 to 8)	0.2 (0.1 to 0.3)	6 (2.1 to 8.2)	0.3 (0.1 to 0.4)	8.3 (3.1 to 11.5)	0.3 (0.1 to 0.5)	8.6 (3.3 to 11.9)
Low-middle SDI	Low physical activity	0.2 (0.1 to 0.3)	3.7 (2 to 6.4)	0.1 (0.1 to 0.2)	3.8 (2 to 6.6)	0.2 (0.1 to 0.3)	4.4 (2.4 to 7.6)	0.2 (0.1 to 0.3)	4.6 (2.5 to 7.8)
Low-middle SDI	Smoking	0.2 (0.2 to 0.3)	6.2 (3.8 to 8.8)	0.2 (0.1 to 0.3)	6.4 (3.9 to 9.1)	0.2 (0.1 to 0.3)	4.9 (2.9 to 7)	0.2 (0.1 to 0.3)	5.1 (3.1 to 7.2)
Low-middle SDI	Secondhand smoke	0.5 (0.1 to 0.8)	14.7 (3.5 to 25.5)	0.4 (0.1 to 0.7)	15.1 (3.6 to 26.1)	0.5 (0.1 to 0.8)	15 (3.3 to 25.9)	0.5 (0.1 to 1)	15.5 (3.5 to 26.8)
Low-middle SDI	Metabolic risks	0.9 (0.3 to 1.9)	20.4 (5.5 to 44.5)	0.6 (0.2 to 1.3)	21 (5.7 to 45.8)	1.7 (0.6 to 3.4)	38.5 (11.7 to 80.1)	1.5 (0.5 to 3.1)	40 (12.3 to 82.8)
Low-middle SDI	High body-mass index	0.2 (0.1 to 0.6)	4.4 (0.3 to 11.9)	0.2 (0 to 0.4)	4.5 (0.4 to 12.3)	0.6 (0.2 to 1.1)	9.3 (-0.2 to 22.4)	0.4 (0.1 to 1)	9.8 (-0.2 to 23.3)
Low-middle SDI	High fasting plasma glucose	0.7 (0.1 to 1.5)	16.5 (3 to 38.7)	0.5 (0.1 to 1.2)	16.9 (3.1 to 40.1)	1.2 (0.2 to 2.8)	30.5 (5.7 to 71)	1.1 (0.2 to 2.7)	31.6 (5.9 to 73.6)
Middle SDI	All risk factors	2.3 (1.5 to 3.3)	62.3 (40 to 87.2)	2.3 (1.3 to 3.4)	64.5 (41.6 to 90.5)	3.2 (2 to 4.5)	81.5 (52.7 to 114.6)	5.1 (3 to 7.8)	86.6 (56.2 to 120.8)
Middle SDI	Behavioral risks	1.3 (0.9 to 1.7)	39.2 (26.7 to 51.2)	1.4 (0.8 to 2)	40.6 (27.4 to 53.1)	1.4 (1 to 1.8)	40.9 (28.7 to 52.6)	2.5 (1.5 to 3.6)	43.4 (30.3 to 55.8)
Middle SDI	Alcohol use	0.3 (0.2 to 0.3)	8.8 (6.9 to 10.8)	0.3 (0.2 to 0.4)	9.1 (7.1 to 11.2)	0.3 (0.3 to 0.4)	10.2 (8 to 12.6)	0.6 (0.4 to 0.8)	10.8 (8.4 to 13.3)
Middle SDI	Diet high in red meat	0.3 (0.1 to 0.4)	8.7 (3 to 11.9)	0.3 (0.1 to 0.5)	9 (3.1 to 12.3)	0.4 (0.2 to 0.5)	11.2 (5.2 to 15.4)	0.8 (0.3 to 1.2)	12 (5.5 to 16.3)
Middle SDI	Low physical activity	0.1 (0.1 to 0.2)	3 (1.7 to 5.3)	0.1 (0.1 to 0.2)	3.1 (1.7 to 5.6)	0.1 (0.1 to 0.2)	3.3 (1.8 to 5.9)	0.2 (0.1 to 0.4)	3.5 (1.9 to 6.3)
Middle SDI	Smoking	0.2 (0.1 to 0.3)	5.3 (3.4 to 7.2)	0.2 (0.1 to 0.3)	5.5 (3.5 to 7.5)	0.2 (0.1 to 0.2)	4.2 (2.7 to 5.8)	0.3 (0.1 to 0.4)	4.4 (2.9 to 6.1)
Middle SDI	Secondhand smoke	0.5 (0.1 to 0.8)	15.2 (3.6 to 26.2)	0.5 (0.1 to 0.9)	15.7 (3.7 to 27)	0.4 (0.1 to 0.8)	13.8 (3.4 to 23.7)	0.8 (0.2 to 1.5)	14.6 (3.6 to 25)
Middle SDI	Metabolic risks	1.1 (0.4 to 2.1)	25.9 (8.5 to 50.4)	1 (0.3 to 2)	26.9 (8.8 to 52.2)	2 (0.9 to 3.5)	45.5 (18.4 to 81.6)	2.9 (1.1 to 5.3)	48.4 (20.2 to 86.2)
Middle SDI	High body-mass index	0.5 (0.1 to 1.1)	10.7 (2 to 25)	0.4 (0.1 to 1)	11.2 (2.1 to 25.9)	1.1 (0.4 to 2.1)	24.5 (7.3 to 48.5)	1.6 (0.5 to 3.3)	26.2 (7.9 to 51.3)
Middle SDI	High fasting plasma glucose	0.7 (0.1 to 1.5)	15.9 (3 to 36.4)	0.6 (0.1 to 1.4)	16.5 (3.1 to 37.8)	1 (0.2 to 2.2)	23.1 (4.4 to 51.7)	1.4 (0.3 to 3.3)	24.5 (4.7 to 54.7)
**Countries**									
Afghanistan	All risk factors	2 (1.1 to 3.3)	51.7 (26.6 to 87.9)	1.3 (0.6 to 2.3)	53 (27.3 to 90.2)	3.3 (1.6 to 6)	80.1 (37.8 to 149.8)	2.1 (1 to 4)	82.3 (38.7 to 153.3)
Afghanistan	Behavioral risks	1 (0.6 to 1.5)	31.4 (17.6 to 46)	0.7 (0.4 to 1.1)	32.1 (18 to 46.8)	1.2 (0.7 to 1.8)	34.8 (19.3 to 52.4)	0.9 (0.5 to 1.4)	35.6 (19.8 to 53.6)
Afghanistan	Alcohol use	0 (0 to 0)	0 (0 to 0)	0 (0 to 0)	0 (0 to 0)	0 (0 to 0)	0.5 (0.2 to 0.8)	0 (0 to 0)	0.5 (0.2 to 0.8)

Location	Risk factor	1990				2019			
		Deaths	YLLs (Years of Life Lost)	YLDs (Years Lived with Disability)	DALYs (Disability-Adjusted Life Years)	Deaths	YLLs (Years of Life Lost)	YLDs (Years Lived with Disability)	DALYs (Disability-Adjusted Life Years)
Afghanistan	Diet high in red meat	0.4 (0.1 to 0.6)	13.1 (3.9 to 20)	0.3 (0.1 to 0.5)	13.4 (4 to 20.5)	0.3 (0.1 to 0.5)	10.3 (2.2 to 16.4)	0.3 (0.1 to 0.4)	10.6 (2.3 to 16.8)
Afghanistan	Low physical activity	0.2 (0.1 to 0.4)	5.7 (2.4 to 10.4)	0.1 (0.1 to 0.3)	5.9 (2.5 to 10.7)	0.3 (0.1 to 0.6)	7.3 (2.9 to 13.6)	0.2 (0.1 to 0.4)	7.5 (3 to 14)
Afghanistan	Smoking	0.1 (0 to 0.1)	1.6 (0.9 to 2.4)	0 (0 to 0.1)	1.6 (0.9 to 2.5)	0.1 (0.1 to 0.2)	3.3 (1.9 to 5.3)	0.1 (0 to 0.1)	3.4 (1.9 to 5.4)
Afghanistan	Secondhand smoke	0.4 (0.1 to 0.7)	11.7 (2.7 to 21.6)	0.3 (0.1 to 0.5)	12 (2.8 to 22.1)	0.5 (0.1 to 0.9)	14.3 (3.6 to 26.6)	0.4 (0.1 to 0.7)	14.7 (3.6 to 27.2)
Afghanistan	Metabolic risks	1 (0.3 to 2.3)	22 (0.5 to 54)	0.6 (0.1 to 1.4)	22.6 (0.7 to 55.4)	2.3 (0.7 to 4.8)	49.2 (8.6 to 114.4)	1.4 (0.3 to 3.1)	50.5 (9 to 116.9)
Afghanistan	High body-mass index	0.2 (-0.1 to 0.6)	0.5 (-9.1 to 8.7)	0.1 (-0.1 to 0.3)	0.6 (-9.2 to 8.9)	0.5 (0 to 1.1)	3.9 (-10.8 to 18.3)	0.2 (-0.2 to 0.6)	4 (-10.9 to 18.9)
Afghanistan	High fasting plasma glucose	0.9 (0.2 to 2)	22 (3.9 to 51.7)	0.5 (0.1 to 1.4)	22.5 (4 to 53)	1.9 (0.4 to 4.3)	47 (9.5 to 108.2)	1.3 (0.2 to 3.1)	48.3 (9.8 to 110.8)
Albania	All risk factors	1.5 (1 to 2)	38.8 (24.4 to 52.7)	2 (1.1 to 2.9)	40.8 (25.7 to 55)	2.6 (1.6 to 4)	65.9 (40.9 to 99.9)	5.2 (2.9 to 8.4)	71 (43.9 to 108.3)
Albania	Behavioral risks	1 (0.7 to 1.3)	31 (20.9 to 40.8)	1.4 (0.9 to 2.1)	32.4 (21.8 to 42.3)	1.7 (1.1 to 2.4)	51.3 (33.6 to 74.1)	4 (2.3 to 6.3)	55.3 (36.1 to 79.5)
Albania	Alcohol use	0.2 (0.1 to 0.3)	8 (5.2 to 11)	0.3 (0.2 to 0.6)	8.3 (5.4 to 11.4)	0.5 (0.3 to 0.7)	16.9 (10.9 to 25.3)	1.3 (0.8 to 2.1)	18.2 (11.7 to 27.2)
Albania	Diet high in red meat	0.2 (0.1 to 0.3)	7.9 (2.1 to 10.7)	0.4 (0.1 to 0.6)	8.3 (2.2 to 11.2)	0.5 (0.2 to 0.8)	16 (6.8 to 25.6)	1.3 (0.5 to 2.1)	17.3 (7.3 to 27.6)
Albania	Low physical activity	0.1 (0 to 0.1)	2 (1.2 to 3.6)	0.1 (0.1 to 0.2)	2.1 (1.3 to 3.8)	0.1 (0.1 to 0.2)	2.7 (1.4 to 4.7)	0.2 (0.1 to 0.4)	2.9 (1.5 to 5.1)
Albania	Smoking	0.2 (0.1 to 0.2)	3.8 (2.4 to 5.7)	0.2 (0.1 to 0.3)	4 (2.5 to 6)	0.3 (0.2 to 0.4)	7 (4.1 to 10.7)	0.6 (0.3 to 0.9)	7.5 (4.4 to 11.7)
Albania	Secondhand smoke	0.3 (0.1 to 0.6)	10.6 (2.7 to 18)	0.5 (0.1 to 0.9)	11 (2.8 to 18.9)	0.4 (0.1 to 0.7)	11.9 (3 to 22.6)	0.9 (0.2 to 1.8)	12.8 (3.2 to 24.4)
Albania	Metabolic risks	0.6 (0.1 to 1.1)	8.7 (-2 to 20.6)	0.6 (0.1 to 1.3)	9.3 (-1.9 to 21.7)	1 (0.3 to 2.1)	16.8 (-0.4 to 41.1)	1.3 (-0.1 to 3.2)	18.1 (-0.5 to 44.7)
Albania	High body-mass index	0.3 (0 to 0.6)	2.4 (-5.6 to 9.9)	0.2 (-0.1 to 0.7)	2.7 (-5.7 to 10.4)	0.5 (0 to 1.2)	5.2 (-8.2 to 19.4)	0.4 (-0.7 to 1.5)	5.5 (-8.8 to 21.1)
Albania	High fasting plasma glucose	0.3 (0.1 to 0.7)	6.6 (1.2 to 15.7)	0.3 (0.1 to 0.9)	6.9 (1.2 to 16.4)	0.5 (0.1 to 1.3)	12.3 (2.2 to 30.3)	1 (0.2 to 2.6)	13.3 (2.3 to 32.9)
Algeria	All risk factors	2 (1.1 to 3.2)	45.3 (21.7 to 75.7)	1.8 (0.9 to 3.2)	47.1 (22.9 to 78.7)	3 (1.5 to 4.9)	65.1 (29.9 to 112.1)	3.7 (1.7 to 6.7)	68.9 (31.3 to 118.6)
Algeria	Behavioral risks	1.1 (0.6 to 1.6)	31.2 (17.9 to 46.3)	1.2 (0.6 to 1.9)	32.4 (18.8 to 48.1)	1.2 (0.7 to 1.8)	33.7 (19.8 to 50.9)	1.9 (1 to 3.2)	35.7 (20.9 to 53.7)
Algeria	Alcohol use	0 (0 to 0.1)	1.5 (0.9 to 2.4)	0.1 (0 to 0.1)	1.6 (0.9 to 2.5)	0.1 (0.1 to 0.1)	2.7 (1.6 to 4)	0.1 (0.1 to 0.2)	2.8 (1.7 to 4.2)
Algeria	Diet high in red meat	0.2 (0 to 0.3)	6.6 (1.3 to 10.5)	0.2 (0 to 0.4)	6.9 (1.3 to 10.9)	0.3 (0 to 0.4)	7.6 (1.5 to 11.9)	0.4 (0.1 to 0.8)	8 (1.6 to 12.6)
Algeria	Low physical activity	0.3 (0.1 to 0.5)	5.8 (2.3 to 11)	0.2 (0.1 to 0.5)	6.1 (2.4 to 11.5)	0.3 (0.1 to 0.5)	6.9 (2.8 to 12.8)	0.4 (0.2 to 0.8)	7.3 (2.9 to 13.5)
Algeria	Smoking	0.1 (0.1 to 0.1)	2.4 (1.4 to 3.9)	0.1 (0 to 0.2)	2.5 (1.5 to 4)	0.1 (0.1 to 0.1)	2.1 (1.2 to 3.3)	0.1 (0.1 to 0.2)	2.2 (1.3 to 3.5)
Algeria	Secondhand smoke	0.5 (0.1 to 0.9)	15.7 (3.6 to 27.3)	0.6 (0.1 to 1.1)	16.2 (3.7 to 28.4)	0.5 (0.1 to 0.9)	15.5 (3.9 to 28.5)	0.9 (0.2 to 1.7)	16.4 (4.1 to 30.1)
Algeria	Metabolic risks	1 (0.2 to 2.2)	15.5 (-5.6 to 42.9)	0.8 (0 to 1.9)	16.2 (-5.7 to 44.6)	2 (0.6 to 3.9)	34.7 (0.7 to 78.8)	2 (0 to 4.8)	36.7 (0.6 to 83.6)
Algeria	High body-mass index	0.3 (-0.1 to 0.8)	-1.5 (-15.1 to 10.3)	0.1 (-0.4 to 0.5)	-1.4 (-15.6 to 10.8)	0.6 (-0.1 to 1.5)	2.9 (-15.5 to 21.8)	0.2 (-0.9 to 1.2)	3.1 (-16.6 to 23)
Algeria	High fasting plasma glucose	0.8 (0.1 to 1.8)	17.7 (3.3 to 43.1)	0.7 (0.1 to 1.8)	18.4 (3.5 to 44.7)	1.5 (0.3 to 3.3)	33.9 (6.5 to 77.6)	2 (0.4 to 4.7)	35.9 (6.9 to 82.6)
American Samoa	All risk factors	10.2 (6.1 to 14.5)	250.2 (151.6 to 353.9)	8.8 (4.9 to 13.5)	258.9 (157.2 to 367.3)	15.4 (8.9 to 22.9)	381.1 (226.2 to 561.3)	15.1 (8.4 to 23.7)	396.1 (235.6 to 580)
American Samoa	Behavioral risks	3 (1.9 to 4)	81.1 (52.1 to 110.4)	2.8 (1.6 to 4.2)	83.9 (54 to 113.6)	3.9 (2.6 to 5.3)	108.4 (71.1 to 148.6)	4.3 (2.5 to 6.5)	112.6 (73.7 to 155)
American Samoa	Alcohol use	0.1 (0 to 0.2)	3.7 (1 to 7.2)	0.1 (0 to 0.3)	3.8 (1.1 to 7.5)	0.1 (0 to 0.3)	4.6 (1.2 to 9.5)	0.2 (0 to 0.4)	4.8 (1.2 to 9.9)
American Samoa	Diet high in red meat	0.7 (0.2 to 1.1)	20.4 (6.7 to 31.2)	0.7 (0.2 to 1.1)	21.1 (7 to 32.3)	0.9 (0.3 to 1.4)	26.7 (8.6 to 41.2)	1.1 (0.3 to 1.8)	27.8 (8.8 to 42.7)
American Samoa	Low physical activity	0.5 (0.2 to 0.9)	10.6 (3.9 to 20.1)	0.4 (0.1 to 0.7)	10.9 (4 to 20.8)	0.7 (0.3 to 1.3)	16 (5.5 to 30.3)	0.6 (0.2 to 1.3)	16.6 (5.7 to 31.5)
American Samoa	Smoking	1 (0.6 to 1.5)	27.7 (14.9 to 42)	1 (0.5 to 1.6)	28.6 (15.6 to 43.4)	1.3 (0.8 to 1.9)	35.4 (19.9 to 53.6)	1.4 (0.7 to 2.3)	36.8 (20.6 to 55.6)
American Samoa	Secondhand smoke	0.8 (0.2 to 1.4)	23.2 (5.1 to 41.1)	0.8 (0.2 to 1.5)	24 (5.2 to 42.3)	1.1 (0.3 to 1.9)	31.1 (7.7 to 56.8)	1.2 (0.3 to 2.3)	32.3 (8 to 59.5)
American Samoa	Metabolic risks	8.4 (4 to 13.1)	196.4 (94.3 to 305.2)	6.9 (3.3 to 11.6)	203.3 (98.1 to 315.5)	13.2 (6.4 to 20.9)	314.1 (151.5 to 505.5)	12.4 (6 to 20.4)	326.6 (157.4 to 523.1)
American Samoa	High body-mass index	6.3 (2.4 to 10.7)	148.5 (61.8 to 246.3)	5.2 (1.9 to 9.3)	153.7 (64.1 to 254)	8.9 (3.3 to 15.2)	210.2 (78.3 to 354.4)	8.3 (3.1 to 14.1)	218.4 (81.3 to 367.4)
American Samoa	High fasting plasma glucose	3 (0.6 to 6.7)	68.8 (13.3 to 154)	2.4 (0.5 to 5.8)	71.3 (13.7 to 159.4)	6.1 (1.4 to 13)	148.2 (34.2 to 319.7)	5.9 (1.3 to 13.5)	154.1 (35.4 to 330.2)
Andorra	All risk factors	6.6 (4.6 to 9.6)	168 (118.7 to 242.7)	15.7 (9.6 to 24)	183.7 (129.7 to 264.2)	5.8 (3.9 to 8.2)	142.4 (96.4 to 203.1)	20 (11.7 to 31.1)	162.4 (109.9 to 230)
Andorra	Behavioral risks	5.3 (3.8 to 7.6)	148.6 (104.5 to 210)	13.7 (8.2 to 20.7)	162.3 (114.6 to 231.2)	4.3 (2.9 to 6)	116.6 (80 to 163.1)	16.3 (9.6 to 25.1)	132.9 (91.8 to 187.7)

Location	Risk factor	1990				2019			
		Deaths	YLLs (Years of Life Lost)	YLDs (Years Lived with Disability)	DALYs (Disability-Adjusted Life Years)	Deaths	YLLs (Years of Life Lost)	YLDs (Years Lived with Disability)	DALYs (Disability-Adjusted Life Years)
Andorra	Alcohol use	2.8 (1.9 to 4.1)	78.4 (53 to 114.8)	7.2 (4.1 to 11.3)	85.7 (58.3 to 124.7)	2.2 (1.4 to 3.1)	61.1 (39.8 to 84.9)	8.5 (4.8 to 13.2)	69.6 (46.1 to 98.1)
Andorra	Diet high in red meat	1.1 (0.5 to 1.7)	28.8 (13.3 to 46.2)	2.7 (1.1 to 4.6)	31.5 (14.4 to 51.1)	0.9 (0.4 to 1.5)	24.2 (10.9 to 38.8)	3.4 (1.4 to 6)	27.6 (12.6 to 44.5)
Andorra	Low physical activity	0.3 (0.1 to 0.7)	7.2 (2.2 to 15.8)	0.7 (0.2 to 1.6)	7.9 (2.5 to 17.1)	0.3 (0.1 to 0.6)	7 (2.2 to 14.3)	1 (0.3 to 2.1)	8 (2.6 to 16.6)
Andorra	Smoking	1.4 (0.8 to 2.1)	39.8 (24 to 60.9)	3.6 (2 to 6)	43.4 (26.1 to 66.9)	1 (0.6 to 1.6)	28.6 (16.5 to 45.4)	4 (2 to 6.8)	32.6 (19 to 51.8)
Andorra	Secondhand smoke	0.4 (0.1 to 0.7)	11.9 (2.6 to 22.7)	1.1 (0.2 to 2.1)	13 (2.8 to 24.7)	0.3 (0.1 to 0.5)	8.2 (1.9 to 15.5)	1.1 (0.3 to 2.2)	9.4 (2.1 to 17.5)
Andorra	Metabolic risks	1.6 (0.6 to 3.3)	26.5 (3.1 to 60.7)	2.8 (0.5 to 6)	29.2 (3.6 to 66.2)	1.9 (0.6 to 3.9)	34.7 (7.1 to 74.7)	5 (1 to 11)	39.7 (8 to 86)
Andorra	High body-mass index	0.9 (0.2 to 2)	11.9 (-4.8 to 33.1)	1.3 (-0.3 to 3.3)	13.2 (-5.5 to 36.1)	0.9 (0.2 to 2)	13 (-2.8 to 33.7)	1.9 (-0.4 to 4.9)	14.9 (-3.3 to 38.6)
Andorra	High fasting plasma glucose	0.8 (0.1 to 1.9)	15.4 (2.7 to 38.6)	1.5 (0.3 to 4.1)	17 (2.9 to 43.3)	1.1 (0.2 to 2.8)	23.1 (4 to 57.8)	3.3 (0.6 to 8.9)	26.4 (4.6 to 66.7)
Angola	All risk factors	2 (1.1 to 3.1)	54.3 (31.6 to 85.5)	1.2 (0.7 to 2)	55.6 (32.2 to 87.3)	4.2 (2.7 to 6.3)	108.5 (69.2 to 161.8)	2.9 (1.8 to 4.7)	111.4 (71.4 to 166.1)
Angola	Behavioral risks	1.3 (0.8 to 1.9)	39.9 (25 to 59)	0.8 (0.5 to 1.3)	40.8 (25.5 to 60.5)	2.9 (2 to 4)	81.3 (55.6 to 114.8)	2.1 (1.3 to 3.3)	83.4 (57.1 to 118.2)
Angola	Alcohol use	0.6 (0.4 to 1)	20.2 (12.2 to 29.8)	0.4 (0.2 to 0.7)	20.6 (12.5 to 30.4)	1.9 (1.3 to 2.6)	53.7 (35.6 to 76.1)	1.4 (0.8 to 2.2)	55.1 (36.7 to 78.5)
Angola	Diet high in red meat	0.2 (0 to 0.4)	6.7 (1.2 to 11.2)	0.1 (0 to 0.2)	6.8 (1.2 to 11.4)	0.4 (0.1 to 0.6)	10.9 (2.2 to 18)	0.3 (0.1 to 0.5)	11.2 (2.3 to 18.5)
Angola	Low physical activity	0.2 (0.1 to 0.3)	3.7 (1.7 to 7.3)	0.1 (0 to 0.2)	3.8 (1.7 to 7.5)	0.3 (0.1 to 0.5)	5.5 (2.4 to 10.8)	0.2 (0.1 to 0.3)	5.6 (2.5 to 11.1)
Angola	Smoking	0.1 (0.1 to 0.2)	3.5 (1.8 to 5.6)	0.1 (0 to 0.1)	3.6 (1.8 to 5.8)	0.2 (0.1 to 0.3)	4.4 (2.4 to 7.3)	0.1 (0.1 to 0.2)	4.6 (2.4 to 7.5)
Angola	Secondhand smoke	0.2 (0 to 0.4)	7.3 (1.7 to 14.2)	0.1 (0 to 0.3)	7.5 (1.7 to 14.5)	0.3 (0.1 to 0.6)	10.4 (2.3 to 19.3)	0.3 (0.1 to 0.5)	10.6 (2.3 to 19.8)
Angola	Metabolic risks	0.7 (0.2 to 1.7)	15.9 (2.5 to 38.6)	0.4 (0.1 to 1)	16.3 (2.6 to 39.7)	1.6 (0.5 to 3.3)	31.8 (5.9 to 72.8)	0.9 (0.2 to 2.1)	32.7 (6.1 to 74.9)
Angola	High body-mass index	0.1 (0 to 0.3)	1.1 (-1.8 to 5.5)	0 (0 to 0.2)	1.2 (-1.9 to 5.6)	0.4 (0 to 1)	6 (-5 to 19.6)	0.2 (-0.1 to 0.6)	6.3 (-5.1 to 20.1)
Angola	High fasting plasma glucose	0.6 (0.1 to 1.6)	15 (2.6 to 37.8)	0.4 (0.1 to 0.9)	15.3 (2.6 to 38.8)	1.2 (0.2 to 2.7)	26.5 (4.8 to 63.5)	0.8 (0.1 to 1.9)	27.2 (4.9 to 65.2)
Antigua and Barbuda	All risk factors	4.3 (2.6 to 6.6)	104.7 (62.5 to 158.9)	5.1 (2.8 to 8.3)	109.9 (65.6 to 166.2)	7.8 (4.7 to 12.4)	177.7 (108.7 to 279.2)	11.4 (6.4 to 19)	189.1 (115.9 to 298.1)
Antigua and Barbuda	Behavioral risks	2.1 (1.5 to 2.7)	61 (43.8 to 79)	2.8 (1.8 to 4.1)	63.8 (46.1 to 82.1)	3.4 (2.6 to 4.3)	91.5 (69.2 to 117.9)	5.7 (3.8 to 8.2)	97.1 (73.8 to 124.6)
Antigua and Barbuda	Alcohol use	0.6 (0.4 to 0.8)	20.3 (12.6 to 28.6)	0.9 (0.5 to 1.4)	21.2 (13.1 to 30)	1.4 (1 to 1.8)	41.7 (30.4 to 56.1)	2.5 (1.6 to 3.7)	44.2 (32.1 to 59.3)
Antigua and Barbuda	Diet high in red meat	0.5 (0.1 to 0.7)	15.3 (3.3 to 21.4)	0.7 (0.2 to 1.1)	16 (3.5 to 22.4)	0.6 (0.1 to 0.9)	17 (3.9 to 24.9)	1.1 (0.2 to 1.7)	18.1 (4.1 to 26.5)
Antigua and Barbuda	Low physical activity	0.4 (0.1 to 0.7)	8.1 (3 to 15.7)	0.4 (0.1 to 0.9)	8.5 (3.1 to 16.7)	0.6 (0.2 to 1.1)	12.1 (3.9 to 23.2)	0.8 (0.3 to 1.6)	12.9 (4.1 to 24.5)
Antigua and Barbuda	Smoking	0.4 (0.3 to 0.6)	11 (7 to 16)	0.5 (0.3 to 0.8)	11.5 (7.4 to 16.8)	0.6 (0.4 to 0.9)	15.5 (9.8 to 22.7)	1 (0.6 to 1.6)	16.4 (10.5 to 24.4)
Antigua and Barbuda	Secondhand smoke	0.3 (0.1 to 0.5)	8.7 (2 to 15.3)	0.4 (0.1 to 0.7)	9 (2 to 15.9)	0.3 (0.1 to 0.6)	9.4 (2.3 to 17.1)	0.6 (0.1 to 1.1)	10 (2.4 to 18.2)
Antigua and Barbuda	Metabolic risks	2.4 (0.7 to 4.9)	48.1 (9.1 to 105.7)	2.6 (0.6 to 5.5)	50.7 (9.7 to 111.2)	4.9 (1.8 to 9.6)	97.4 (27.7 to 203)	6.5 (2 to 13.6)	103.9 (30 to 214)
Antigua and Barbuda	High body-mass index	0.6 (0 to 1.5)	7.9 (-9.3 to 26.8)	0.5 (-0.3 to 1.5)	8.5 (-9.6 to 28.1)	1.5 (0.3 to 3.3)	25.3 (-4.1 to 61.4)	1.8 (0.1 to 4.1)	27.1 (-4.3 to 65.7)
Antigua and Barbuda	High fasting plasma glucose	1.8 (0.4 to 4.2)	41.8 (7.9 to 96)	2.1 (0.4 to 5)	43.9 (8.3 to 100.3)	3.6 (0.7 to 8.1)	77 (15.9 to 174.1)	5 (1 to 11.9)	82.1 (16.9 to 186.3)
Argentina	All risk factors	10.5 (8.5 to 12.6)	274.2 (226.2 to 324.3)	11.4 (7.6 to 15.8)	285.6 (236.7 to 338.2)	9.5 (7.3 to 12.1)	226.6 (176.5 to 285.1)	14.1 (8.8 to 21.5)	240.7 (187.6 to 302)
Argentina	Behavioral risks	8.6 (7.3 to 9.9)	240 (204.1 to 276.3)	9.7 (6.5 to 13.5)	249.7 (212.6 to 286.9)	6.8 (5.6 to 8)	179.2 (150.1 to 209.9)	11 (6.8 to 16.2)	190.2 (158.7 to 224.1)
Argentina	Alcohol use	4.2 (3.4 to 5.1)	122.6 (100.2 to 147.6)	4.9 (3.2 to 7)	127.5 (104.1 to 153.4)	3.2 (2.5 to 3.9)	87.4 (68.6 to 107.6)	5.3 (3.2 to 8)	92.7 (72.4 to 114.3)
Argentina	Diet high in red meat	1.9 (0.9 to 2.8)	52.8 (25.5 to 77.1)	2.1 (1 to 3.6)	54.9 (26.3 to 80.7)	1.7 (0.9 to 2.5)	44.6 (21.9 to 65.2)	2.7 (1.3 to 4.8)	47.3 (23.1 to 69.7)
Argentina	Low physical activity	0.1 (0.1 to 0.3)	3.1 (2.2 to 8.7)	0.1 (0.1 to 0.4)	3.2 (2.3 to 9)	0.1 (0.1 to 0.3)	2.9 (1.9 to 7.7)	0.2 (0.1 to 0.5)	3 (2 to 8.1)
Argentina	Smoking	2.5 (1.7 to 3.3)	67.7 (46.6 to 91.4)	2.8 (1.6 to 4.2)	70.5 (48.5 to 95.3)	1.9 (1.3 to 2.4)	47.3 (32.8 to 62.7)	2.9 (1.7 to 4.6)	50.2 (34.6 to 66.9)
Argentina	Secondhand smoke	0.8 (0.2 to 1.4)	22.3 (5.4 to 38.8)	0.9 (0.2 to 1.7)	23.2 (5.6 to 40.4)	0.6 (0.1 to 1)	15.4 (3.5 to 27.2)	0.9 (0.2 to 1.8)	16.4 (3.8 to 29)
Argentina	Metabolic risks	2.6 (0.9 to 5)	48.5 (11.4 to 99)	2.3 (0.6 to 4.7)	50.8 (12 to 103)	3.5 (1.3 to 6.5)	64 (16.9 to 126.6)	4.2 (1.2 to 8.8)	68.2 (18.2 to 134.5)
Argentina	High body-mass index	1.1 (0.2 to 2.3)	15.8 (-4.3 to 40)	0.8 (0 to 2)	16.6 (-4.2 to 42.2)	1.5 (0.4 to 3.1)	22.1 (-3 to 53.5)	1.5 (-0.1 to 3.6)	23.6 (-3 to 56.9)
Argentina	High fasting plasma glucose	1.6 (0.3 to 3.7)	34.3 (6.5 to 78.7)	1.5 (0.3 to 3.7)	35.9 (6.8 to 82.5)	2.2 (0.4 to 4.8)	44.9 (8.4 to 101)	2.9 (0.5 to 7.3)	47.8 (9 to 106.9)

Location	Risk factor	1990				2019			
		Deaths	YLLs (Years of Life Lost)	YLDs (Years Lived with Disability)	DALYs (Disability-Adjusted Life Years)	Deaths	YLLs (Years of Life Lost)	YLDs (Years Lived with Disability)	DALYs (Disability-Adjusted Life Years)
Armenia	All risk factors	5.7 (3.9 to 7.8)	165.9 (112.3 to 222.9)	6.7 (4.1 to 10)	172.6 (116.9 to 231.1)	6 (3.8 to 8.6)	153 (97.3 to 221.3)	9.7 (5.6 to 15)	162.6 (103.5 to 235.5)
Armenia	Behavioral risks	3.7 (2.7 to 4.7)	125.1 (88.6 to 158.1)	4.9 (3 to 7.2)	130 (92.8 to 164.3)	3.1 (2.2 to 4.2)	95 (67.2 to 125.9)	5.9 (3.6 to 8.8)	100.9 (71.2 to 133.5)
Armenia	Alcohol use	1.5 (1.1 to 1.9)	54.9 (41.7 to 68.8)	2.1 (1.4 to 3.1)	57 (43.3 to 71.6)	1.3 (0.9 to 1.7)	42.6 (30.6 to 57)	2.6 (1.7 to 4)	45.3 (32.3 to 60.7)
Armenia	Diet high in red meat	0.8 (0.2 to 1)	25.1 (6.5 to 33.2)	1 (0.3 to 1.6)	26.1 (6.7 to 34.6)	0.8 (0.3 to 1.2)	24.2 (8.8 to 35.2)	1.5 (0.5 to 2.4)	25.8 (9.3 to 37.3)
Armenia	Low physical activity	0.2 (0.1 to 0.3)	4.8 (3.1 to 9.2)	0.2 (0.1 to 0.4)	5 (3.2 to 9.6)	0.2 (0.1 to 0.3)	4.2 (2.5 to 8)	0.3 (0.1 to 0.5)	4.5 (2.6 to 8.5)
Armenia	Smoking	0.3 (0.2 to 0.4)	7.7 (4.4 to 11.8)	0.3 (0.2 to 0.5)	8 (4.6 to 12.3)	0.2 (0.1 to 0.3)	4.8 (2.9 to 7.4)	0.3 (0.2 to 0.5)	5.2 (3 to 7.8)
Armenia	Secondhand smoke	1.2 (0.3 to 2)	38.9 (9.7 to 66.4)	1.5 (0.4 to 2.8)	40.4 (10.2 to 69)	0.8 (0.2 to 1.4)	24.2 (5.5 to 43.1)	1.5 (0.3 to 2.8)	25.8 (5.9 to 45.8)
Armenia	Metabolic risks	2.2 (0.6 to 4.3)	45.9 (1.7 to 100.4)	2 (0.2 to 4.3)	47.9 (2.1 to 104.4)	3.2 (1.1 to 6)	66.1 (15.2 to 131.2)	4.2 (1 to 8.7)	70.3 (16.2 to 139.4)
Armenia	High body-mass index	0.9 (-0.1 to 2.2)	12.3 (-16.9 to 40.3)	0.6 (-0.5 to 1.7)	12.9 (-17.4 to 42)	1.5 (0.3 to 2.9)	24 (-4.5 to 56.1)	1.5 (-0.3 to 3.7)	25.5 (-4.8 to 59.6)
Armenia	High fasting plasma glucose	1.3 (0.3 to 3)	35.2 (6.6 to 80.5)	1.5 (0.3 to 3.6)	36.7 (6.9 to 84.1)	1.9 (0.4 to 4.3)	45.6 (8.7 to 104)	2.9 (0.5 to 6.9)	48.5 (9.2 to 111.4)
Australia	All risk factors	8.5 (7 to 10)	226.8 (190.2 to 261.2)	19.8 (13.3 to 27.8)	246.6 (205.9 to 285.5)	5.9 (4.6 to 7.4)	140 (111.4 to 171.7)	20.6 (12.6 to 30.9)	160.6 (127.1 to 199.6)
Australia	Behavioral risks	7.1 (6.2 to 8.1)	202.6 (175.3 to 231.3)	17.3 (11.5 to 24.2)	220 (188.2 to 251.9)	4.3 (3.6 to 5)	114.4 (96.1 to 133.5)	16.5 (10.4 to 24.4)	130.8 (108.9 to 154.7)
Australia	Alcohol use	3.6 (2.9 to 4.3)	104.1 (85.9 to 124)	8.9 (5.8 to 12.5)	113 (92.8 to 134.7)	2.3 (1.8 to 2.8)	61 (48.4 to 74.4)	8.7 (5.3 to 13.5)	69.7 (55.1 to 86.6)
Australia	Diet high in red meat	1.6 (0.8 to 2.3)	44.4 (21.6 to 65.4)	3.8 (1.8 to 6.3)	48.3 (23.2 to 71.5)	1.1 (0.5 to 1.5)	27.2 (13.3 to 40.6)	3.9 (1.8 to 6.8)	31.2 (15.3 to 46.6)
Australia	Low physical activity	0.4 (0.1 to 0.7)	8 (2.9 to 15.7)	0.8 (0.3 to 1.7)	8.7 (3.2 to 17.4)	0.3 (0.1 to 0.6)	7 (2.2 to 13.4)	1.1 (0.3 to 2.2)	8 (2.6 to 15.3)
Australia	Smoking	2 (1.4 to 2.5)	57.1 (41.3 to 74)	4.8 (2.8 to 7.3)	61.9 (44.8 to 80.8)	0.9 (0.6 to 1.2)	25.1 (17.7 to 33.1)	3.6 (2 to 5.7)	28.7 (20 to 37.7)
Australia	Secondhand smoke	0.4 (0.1 to 0.7)	13.3 (3.2 to 22.9)	1.1 (0.2 to 2)	14.4 (3.5 to 24.8)	0.2 (0.1 to 0.4)	6.3 (1.5 to 11.1)	0.9 (0.2 to 1.7)	7.2 (1.7 to 12.6)
Australia	Metabolic risks	1.9 (0.7 to 3.5)	33.6 (3.2 to 68.9)	3.5 (0.7 to 7)	37 (4.1 to 74.9)	2 (0.8 to 3.7)	34.5 (6.5 to 67.7)	5.6 (1.3 to 11.5)	40.1 (8.5 to 78.3)
Australia	High body-mass index	1.1 (0.2 to 2.2)	16.1 (-6 to 40.2)	1.7 (-0.3 to 4.3)	17.9 (-6.1 to 43.8)	1.1 (0.3 to 2.2)	15.9 (-3.3 to 38.9)	2.7 (-0.1 to 6.2)	18.6 (-3.4 to 44)
Australia	High fasting plasma glucose	0.9 (0.2 to 2)	18.4 (3.4 to 42.5)	1.8 (0.3 to 4.6)	20.2 (3.7 to 46.9)	1 (0.2 to 2.3)	20.2 (3.8 to 45.9)	3.1 (0.6 to 7.9)	23.3 (4.5 to 53.2)
Austria	All risk factors	8.1 (6.7 to 9.6)	217 (180.6 to 252.4)	18.3 (12.3 to 25.4)	235.3 (196.7 to 274.3)	6 (4.7 to 7.6)	139.4 (112.7 to 170.6)	17.6 (11.2 to 26.2)	157 (126.7 to 192.3)
Austria	Behavioral risks	6.7 (5.7 to 7.6)	190.2 (162.7 to 216.4)	15.9 (10.8 to 22.1)	206 (175.4 to 234.7)	4.5 (3.8 to 5.2)	112.9 (95.9 to 131.5)	14.3 (9.2 to 21.1)	127.2 (106.8 to 148.8)
Austria	Alcohol use	3.6 (3 to 4.3)	104.1 (85.8 to 123.8)	8.7 (5.8 to 12.6)	112.8 (92.8 to 134.6)	2.3 (1.8 to 2.8)	57.7 (45.9 to 70.9)	7.3 (4.7 to 11.2)	65 (51.3 to 80.4)
Austria	Diet high in red meat	1.5 (0.7 to 2)	41.2 (20.4 to 56.5)	3.5 (1.6 to 5.5)	44.7 (22.1 to 61.5)	0.9 (0.4 to 1.3)	22.7 (11.1 to 31.4)	2.9 (1.2 to 4.7)	25.5 (12.3 to 35.6)
Austria	Low physical activity	0.4 (0.1 to 0.8)	9.3 (3.1 to 18.4)	0.8 (0.3 to 1.8)	10.1 (3.4 to 20.1)	0.3 (0.1 to 0.6)	6.8 (2.2 to 12.8)	0.9 (0.3 to 1.8)	7.7 (2.5 to 14.7)
Austria	Smoking	1.4 (1 to 1.8)	42 (30.2 to 55.1)	3.5 (2 to 5.3)	45.5 (32.6 to 59.9)	1.1 (0.8 to 1.5)	30.6 (22 to 39.8)	3.9 (2.3 to 6.1)	34.5 (24.7 to 45)
Austria	Secondhand smoke	0.4 (0.1 to 0.8)	14.5 (3.4 to 25.3)	1.2 (0.3 to 2.2)	15.6 (3.7 to 27.3)	0.3 (0.1 to 0.5)	7.6 (1.9 to 13.2)	0.9 (0.2 to 1.8)	8.5 (2.1 to 14.9)
Austria	Metabolic risks	1.9 (0.7 to 3.6)	36.2 (9.5 to 72.2)	3.2 (0.9 to 6.4)	39.4 (10.4 to 77.9)	2.1 (0.8 to 3.8)	36 (10.5 to 70.3)	4.5 (1.3 to 9.4)	40.6 (11.8 to 78.8)
Austria	High body-mass index	1 (0.2 to 2.2)	17.4 (-1 to 40.9)	1.5 (0 to 3.5)	18.9 (-1.2 to 44.5)	1 (0.3 to 2)	14.5 (0.2 to 33.2)	1.8 (-0.1 to 4.3)	16.3 (0.2 to 38.1)
Austria	High fasting plasma glucose	1 (0.2 to 2.2)	19.9 (3.6 to 45.8)	1.8 (0.3 to 4.5)	21.7 (3.9 to 49.9)	1.2 (0.2 to 2.7)	23.1 (4.3 to 52.2)	3 (0.6 to 7.5)	26.1 (4.9 to 58.5)
Azerbaijan	All risk factors	3.1 (2 to 4.3)	86.7 (55 to 121.9)	3.1 (1.8 to 4.8)	89.9 (56.7 to 125.8)	3.9 (2.3 to 6)	99.3 (55.6 to 154.9)	4.8 (2.5 to 8)	104.1 (58.6 to 162.2)
Azerbaijan	Behavioral risks	2.2 (1.6 to 2.9)	72.8 (52.6 to 96)	2.5 (1.5 to 3.7)	75.2 (54.2 to 99.2)	2.1 (1.4 to 3)	67 (44 to 93.4)	3.2 (1.8 to 4.9)	70.1 (45.8 to 97.8)
Azerbaijan	Alcohol use	1 (0.7 to 1.4)	36.6 (25.4 to 49.8)	1.2 (0.7 to 1.9)	37.8 (26.3 to 51.2)	0.9 (0.6 to 1.3)	30.1 (18.7 to 44.2)	1.4 (0.8 to 2.3)	31.5 (19.6 to 46.5)
Azerbaijan	Diet high in red meat	0.4 (0.1 to 0.5)	12.4 (2.6 to 17.1)	0.4 (0.1 to 0.7)	12.8 (2.7 to 17.6)	0.5 (0.1 to 0.7)	15.4 (4.4 to 23.3)	0.7 (0.2 to 1.2)	16.1 (4.6 to 24.5)
Azerbaijan	Low physical activity	0.1 (0.1 to 0.2)	3 (1.9 to 5.9)	0.1 (0.1 to 0.2)	3.1 (2 to 6.1)	0.1 (0.1 to 0.2)	3.2 (1.8 to 6.1)	0.2 (0.1 to 0.3)	3.4 (2 to 6.4)
Azerbaijan	Smoking	0.1 (0 to 0.1)	2 (1 to 3.2)	0.1 (0 to 0.1)	2 (1 to 3.4)	0.1 (0 to 0.1)	2.3 (1 to 3.9)	0.1 (0 to 0.2)	2.4 (1.1 to 4.1)
Azerbaijan	Secondhand smoke	0.7 (0.2 to 1.2)	22 (5.4 to 38.8)	0.7 (0.2 to 1.4)	22.8 (5.6 to 39.9)	0.6 (0.2 to 1.1)	19.2 (4.9 to 33.8)	0.9 (0.2 to 1.7)	20.1 (5.2 to 35.5)
Azerbaijan	Metabolic risks	1 (0.1 to 2.1)	15.4 (-12.5 to 46.1)	0.7 (-0.3 to 1.9)	16.1 (-12.7 to 47.6)	2 (0.5 to 4)	36.3 (-2.7 to 89)	1.8 (0 to 4.4)	38.2 (-2.6 to 93.6)

Location	Risk factor	1990				2019			
		Deaths	YLLs (Years of Life Lost)	YLDs (Years Lived with Disability)	DALYs (Disability-Adjusted Life Years)	Deaths	YLLs (Years of Life Lost)	YLDs (Years Lived with Disability)	DALYs (Disability-Adjusted Life Years)
Azerbaijan	High body-mass index	0.4 (-0.2 to 1.1)	-2.4 (-22.9 to 14.9)	0.1 (-0.7 to 0.8)	-2.3 (-23.5 to 15.4)	0.8 (-0.1 to 1.8)	5.3 (-19.2 to 30.4)	0.3 (-0.9 to 1.5)	5.6 (-19.9 to 31.7)
Azerbaijan	High fasting plasma glucose	0.7 (0.1 to 1.6)	18.3 (3.4 to 43.1)	0.7 (0.1 to 1.6)	19 (3.5 to 44.6)	1.3 (0.2 to 3)	33 (5.8 to 77.1)	1.6 (0.3 to 4)	34.6 (6.1 to 81)
Bahamas	All risk factors	8.2 (5.6 to 11.5)	221.6 (148.3 to 305.6)	9.8 (5.9 to 15)	231.4 (154.7 to 320.3)	8.6 (5.1 to 13.3)	208.5 (116.5 to 330)	11.8 (6.2 to 19.2)	220.2 (123.2 to 349.2)
Bahamas	Behavioral risks	5.2 (4 to 6.4)	166 (128.2 to 204.4)	6.9 (4.5 to 9.8)	173 (133.7 to 212.8)	4.4 (3 to 5.9)	131.4 (88.5 to 183.5)	7 (4.1 to 10.6)	138.4 (92.9 to 192.6)
Bahamas	Alcohol use	2.4 (1.8 to 3.2)	84.8 (63.1 to 111.5)	3.5 (2.1 to 5.1)	88.3 (65.4 to 116.5)	1.6 (0.7 to 2.5)	53 (24.8 to 85.7)	2.7 (1.1 to 4.7)	55.8 (25.9 to 89.6)
Bahamas	Diet high in red meat	1.3 (0.5 to 1.8)	41.4 (16 to 56.7)	1.7 (0.6 to 2.7)	43.1 (16.7 to 58.9)	1.3 (0.5 to 1.9)	39.6 (15.3 to 58.7)	2.1 (0.8 to 3.5)	41.7 (16.1 to 62)
Bahamas	Low physical activity	0.6 (0.2 to 1.1)	14 (4.8 to 27.8)	0.6 (0.2 to 1.4)	14.7 (5 to 29.1)	0.7 (0.2 to 1.3)	15.7 (5.1 to 31.1)	0.9 (0.3 to 1.9)	16.6 (5.4 to 33)
Bahamas	Smoking	0.6 (0.4 to 0.9)	16.9 (10.7 to 24.3)	0.7 (0.4 to 1.2)	17.7 (11.1 to 25.5)	0.6 (0.3 to 0.8)	14.2 (8.6 to 20.9)	0.8 (0.4 to 1.3)	15 (9.1 to 22)
Bahamas	Secondhand smoke	0.6 (0.1 to 1)	18.4 (4.2 to 33.3)	0.8 (0.2 to 1.5)	19.2 (4.4 to 34.6)	0.5 (0.1 to 0.9)	15.4 (3.3 to 28.4)	0.8 (0.2 to 1.6)	16.2 (3.5 to 29.9)
Bahamas	Metabolic risks	3.5 (1 to 6.9)	64.2 (-2.6 to 151.5)	3.3 (0.3 to 7.5)	67.5 (-2.4 to 160)	4.7 (1.5 to 9.5)	87.3 (5.9 to 199.9)	5.4 (0.7 to 12.1)	92.7 (7 to 209.7)
Bahamas	High body-mass index	1.1 (-0.2 to 2.7)	6.1 (-32.6 to 44.2)	0.7 (-1 to 2.5)	6.8 (-33.8 to 46.3)	1.5 (-0.1 to 3.4)	8.7 (-35.8 to 52)	0.9 (-1.4 to 3.4)	9.7 (-36.7 to 55.2)
Bahamas	High fasting plasma glucose	2.5 (0.5 to 5.7)	61.1 (11.7 to 140.8)	2.8 (0.5 to 6.9)	63.9 (12.1 to 147.1)	3.5 (0.7 to 7.9)	83.2 (16 to 189.6)	4.7 (0.9 to 11.4)	87.9 (16.8 to 201.1)
Bahrain	All risk factors	6.6 (3.9 to 10.1)	150.9 (86.1 to 233.6)	5.8 (3.1 to 9.6)	156.7 (89.6 to 242.3)	7.7 (4.2 to 12.6)	150.1 (73.9 to 255.5)	10.4 (4.8 to 18.4)	160.5 (79.3 to 271.3)
Bahrain	Behavioral risks	2.9 (2.1 to 3.7)	78.8 (55.4 to 100.9)	2.9 (1.8 to 4.4)	81.7 (58 to 104.2)	2.2 (1.5 to 3.1)	53.9 (35.9 to 74.2)	3.8 (2.3 to 5.8)	57.6 (38.2 to 79.5)
Bahrain	Alcohol use	0.5 (0.3 to 0.6)	14.2 (10.1 to 19.6)	0.5 (0.3 to 0.8)	14.7 (10.5 to 20.2)	0.2 (0.1 to 0.3)	5.1 (3.1 to 7.4)	0.4 (0.2 to 0.6)	5.5 (3.4 to 7.9)
Bahrain	Diet high in red meat	0.7 (0.2 to 1)	19 (5.1 to 28.8)	0.7 (0.2 to 1.2)	19.7 (5.3 to 30)	0.6 (0.1 to 0.9)	15.3 (3.7 to 23.7)	1.1 (0.3 to 1.8)	16.4 (4 to 25.4)
Bahrain	Low physical activity	0.6 (0.2 to 1.1)	14.1 (5.4 to 25)	0.6 (0.2 to 1)	14.7 (5.6 to 26.1)	0.7 (0.3 to 1.2)	13.9 (5.4 to 24.7)	1 (0.4 to 1.9)	14.9 (5.7 to 26.5)
Bahrain	Smoking	0.6 (0.4 to 0.9)	16.2 (10.3 to 23.9)	0.6 (0.3 to 1)	16.8 (10.7 to 24.7)	0.3 (0.2 to 0.5)	7.8 (4.9 to 12)	0.5 (0.3 to 0.9)	8.4 (5.2 to 12.9)
Bahrain	Secondhand smoke	0.7 (0.1 to 1.1)	18.8 (3.8 to 33.2)	0.7 (0.2 to 1.3)	19.4 (4 to 34.1)	0.5 (0.1 to 0.9)	13.6 (3 to 24.3)	0.9 (0.2 to 1.8)	14.5 (3.2 to 25.9)
Bahrain	Metabolic risks	4.2 (1.4 to 7.7)	81.7 (17.2 to 164.7)	3.3 (0.8 to 6.7)	85 (18.1 to 171.3)	6 (2.3 to 11.1)	106.4 (28 to 210.8)	7.3 (1.8 to 15)	113.7 (29.4 to 224.7)
Bahrain	High body-mass index	1.6 (0.2 to 3.4)	22.6 (-13.7 to 64.8)	0.9 (-0.4 to 2.6)	23.5 (-14.2 to 67.1)	1.8 (0.3 to 3.6)	19.8 (-14.9 to 56)	1.2 (-1.3 to 3.7)	21 (-16.1 to 60)
Bahrain	High fasting plasma glucose	2.9 (0.6 to 6.4)	64.5 (12.5 to 143)	2.5 (0.5 to 6)	67 (13.1 to 148.3)	4.8 (1.1 to 9.9)	96.2 (21 to 200.9)	6.8 (1.5 to 14.8)	103 (22.5 to 215.6)
Bangladesh	All risk factors	1.4 (0.7 to 2.5)	41.9 (18.6 to 71.1)	1 (0.4 to 1.8)	42.9 (19 to 72.8)	1.8 (0.9 to 3.1)	47.7 (22.1 to 82.3)	1.7 (0.8 to 3.1)	49.4 (22.8 to 84.9)
Bangladesh	Behavioral risks	0.9 (0.4 to 1.4)	27.1 (11.2 to 45)	0.6 (0.3 to 1)	27.7 (11.5 to 46)	0.9 (0.4 to 1.4)	25.1 (12.3 to 40)	0.9 (0.4 to 1.5)	25.9 (12.7 to 41.4)
Bangladesh	Alcohol use	0 (0 to 0)	0.4 (0 to 0.8)	0 (0 to 0)	0.4 (0 to 0.8)	0 (0 to 0.1)	1.2 (0.3 to 2.1)	0 (0 to 0.1)	1.2 (0.3 to 2.2)
Bangladesh	Diet high in red meat	0.1 (0 to 0.1)	2.5 (1 to 3.8)	0.1 (0 to 0.1)	2.5 (1 to 3.9)	0.1 (0 to 0.1)	2.6 (0.9 to 4.1)	0.1 (0 to 0.2)	2.7 (1 to 4.3)
Bangladesh	Low physical activity	0.1 (0.1 to 0.2)	2.5 (1.5 to 4.7)	0.1 (0 to 0.1)	2.6 (1.5 to 4.8)	0.1 (0.1 to 0.2)	2.7 (1.5 to 5)	0.1 (0.1 to 0.2)	2.8 (1.6 to 5.2)
Bangladesh	Smoking	0.1 (0.1 to 0.2)	2.7 (1.3 to 4.5)	0.1 (0 to 0.1)	2.8 (1.3 to 4.7)	0.1 (0 to 0.1)	1.9 (0.9 to 3.2)	0.1 (0 to 0.1)	2 (0.9 to 3.3)
Bangladesh	Secondhand smoke	0.6 (0.1 to 1.1)	19.4 (4.2 to 36.3)	0.4 (0.1 to 0.8)	19.8 (4.3 to 37.2)	0.5 (0.1 to 1)	17.1 (3.9 to 31.2)	0.6 (0.1 to 1.1)	17.7 (4.1 to 32.2)
Bangladesh	Metabolic risks	0.6 (0.1 to 1.5)	15.8 (2.3 to 39.1)	0.4 (0.1 to 1)	16.2 (2.4 to 39.9)	1 (0.3 to 2.3)	24 (4.5 to 56.2)	0.9 (0.2 to 2.1)	24.9 (4.7 to 58.1)
Bangladesh	High body-mass index	0 (0 to 0.2)	0.2 (-2.1 to 2.6)	0 (0 to 0.1)	0.2 (-2.1 to 2.7)	0.2 (0 to 0.4)	1.9 (-3.4 to 8.6)	0.1 (-0.1 to 0.3)	2 (-3.5 to 8.8)
Bangladesh	High fasting plasma glucose	0.6 (0.1 to 1.4)	15.7 (2.6 to 38.6)	0.4 (0.1 to 1)	16.1 (2.7 to 39.3)	0.9 (0.2 to 2.1)	22.5 (4.1 to 54.5)	0.8 (0.1 to 2.1)	23.3 (4.2 to 56.3)
Barbados	All risk factors	6.9 (4.4 to 10.2)	164.9 (102.9 to 239.1)	8.4 (4.7 to 13.1)	173.3 (108.3 to 251.5)	9.4 (5.6 to 14.6)	213.5 (128.3 to 333)	14.5 (8.1 to 23.9)	228 (135.9 to 354.9)
Barbados	Behavioral risks	3.6 (2.7 to 4.4)	107.2 (81.1 to 130.3)	5.1 (3.3 to 7.2)	112.3 (84.4 to 136.8)	4.2 (3 to 5.4)	117 (84.1 to 152.4)	7.5 (4.9 to 11.1)	124.5 (89.4 to 162.9)
Barbados	Alcohol use	1.3 (0.9 to 1.7)	42.3 (30.8 to 54.5)	2 (1.2 to 2.9)	44.3 (31.9 to 57.1)	1.8 (1.3 to 2.5)	56.7 (39.1 to 77.4)	3.6 (2.2 to 5.5)	60.3 (41.7 to 81.8)
Barbados	Diet high in red meat	1.1 (0.4 to 1.5)	32.4 (12.7 to 43.6)	1.5 (0.6 to 2.4)	33.9 (13.3 to 45.6)	1 (0.3 to 1.4)	26.2 (7.5 to 38.7)	1.7 (0.5 to 2.8)	27.9 (7.9 to 41)
Barbados	Low physical activity	0.6 (0.2 to 1.1)	13.7 (4.5 to 25.9)	0.7 (0.2 to 1.4)	14.4 (4.8 to 27.2)	0.8 (0.3 to 1.5)	17.7 (5.6 to 33)	1.2 (0.4 to 2.4)	18.9 (6.1 to 35)
Barbados	Smoking	0.4 (0.3 to 0.6)	12.4 (7.7 to 18.3)	0.6 (0.3 to 0.9)	13 (8.1 to 19.1)	0.4 (0.2 to 0.6)	11.1 (6.8 to 17)	0.7 (0.4 to 1.2)	11.8 (7.2 to 18.1)
Barbados	Secondhand smoke	0.4 (0.1 to 0.7)	11.7 (2.6 to 21.1)	0.5 (0.1 to 1.1)	12.3 (2.7 to 22.1)	0.4 (0.1 to 0.7)	10.7 (2.6 to 19.8)	0.7 (0.1 to 1.3)	11.4 (2.8 to 21.2)

Location	Risk factor	1990				2019			
		Deaths	YLLs (Years of Life Lost)	YLDs (Years Lived with Disability)	DALYs (Disability-Adjusted Life Years)	Deaths	YLLs (Years of Life Lost)	YLDs (Years Lived with Disability)	DALYs (Disability-Adjusted Life Years)
Barbados	Metabolic risks	3.7 (1.2 to 7.1)	65 (5.9 to 141)	3.7 (0.7 to 8)	68.7 (6.5 to 147.6)	5.8 (2.1 to 11.1)	108.9 (24.2 to 230.9)	7.8 (1.9 to 16.3)	116.7 (26 to 247.6)
Barbados	High body-mass index	1.2 (0 to 2.9)	10.1 (-22.3 to 44.1)	0.9 (-0.8 to 2.8)	11 (-23 to 46.3)	2.1 (0.4 to 4.4)	27.9 (-15.6 to 79.1)	2.2 (-0.7 to 6)	30.2 (-16.5 to 84.4)
Barbados	High fasting plasma glucose	2.6 (0.5 to 5.9)	58.2 (11.3 to 131.2)	3 (0.6 to 7.3)	61.2 (11.9 to 138.3)	4.1 (0.8 to 9)	87.5 (17.4 to 200.4)	6 (1.2 to 14.3)	93.5 (18.6 to 214.3)
Belarus	All risk factors	4.8 (3.7 to 5.8)	146.2 (113.6 to 177.5)	7.7 (4.9 to 10.8)	153.8 (120.2 to 186.9)	4.1 (2.8 to 5.7)	113.5 (78.2 to 161.2)	9.6 (5.7 to 14.4)	123.1 (84.8 to 172.7)
Belarus	Behavioral risks	3.8 (3.1 to 4.4)	128.8 (105.3 to 150.6)	6.6 (4.3 to 9.1)	135.4 (110.1 to 158.2)	2.9 (2.1 to 3.9)	91.4 (64.9 to 122.7)	7.6 (4.6 to 11.4)	99 (69.9 to 132.2)
Belarus	Alcohol use	1.9 (1.5 to 2.3)	65.9 (52.1 to 80.1)	3.3 (2.2 to 4.8)	69.3 (54.7 to 84.3)	1.5 (1.1 to 2.1)	48.9 (33.9 to 68.4)	4.1 (2.4 to 6.2)	52.9 (36.9 to 74.4)
Belarus	Diet high in red meat	1 (0.5 to 1.3)	32.3 (16.4 to 43.7)	1.7 (0.8 to 2.7)	34 (17.1 to 46)	0.8 (0.3 to 1.2)	22.3 (10.1 to 35)	1.9 (0.8 to 3.2)	24.1 (11.2 to 37.4)
Belarus	Low physical activity	0.1 (0.1 to 0.2)	3.3 (2.1 to 6.2)	0.2 (0.1 to 0.4)	3.5 (2.2 to 6.5)	0.1 (0.1 to 0.2)	2.7 (1.5 to 5.1)	0.2 (0.1 to 0.5)	2.9 (1.6 to 5.5)
Belarus	Smoking	0.6 (0.4 to 0.9)	21.4 (12.2 to 32)	1.1 (0.5 to 1.8)	22.5 (12.9 to 33.7)	0.4 (0.3 to 0.7)	14.5 (8.3 to 22.5)	1.2 (0.6 to 2)	15.7 (8.9 to 24.4)
Belarus	Secondhand smoke	0.5 (0.1 to 0.9)	17.2 (4.2 to 29.7)	0.9 (0.2 to 1.6)	18.1 (4.4 to 31.3)	0.4 (0.1 to 0.7)	11.3 (2.5 to 20.8)	0.9 (0.2 to 1.8)	12.3 (2.8 to 22.5)
Belarus	Metabolic risks	1.2 (0.3 to 2.3)	21.3 (-4.2 to 50.3)	1.3 (-0.1 to 3.1)	22.6 (-4.1 to 53)	1.4 (0.5 to 2.6)	27.7 (5.1 to 57.5)	2.5 (0.5 to 5.1)	30.2 (5.8 to 62.2)
Belarus	High body-mass index	0.6 (-0.1 to 1.4)	6.5 (-13.7 to 25.5)	0.5 (-0.5 to 1.7)	7.1 (-14.1 to 27.3)	0.9 (0.2 to 1.7)	15.2 (-2.2 to 35.7)	1.4 (-0.1 to 3.3)	16.5 (-2.3 to 38.7)
Belarus	High fasting plasma glucose	0.6 (0.1 to 1.3)	15.4 (2.7 to 35.6)	0.9 (0.1 to 2.1)	16.3 (2.9 to 37.7)	0.6 (0.1 to 1.4)	13.5 (2.4 to 33.9)	1.2 (0.2 to 3.1)	14.7 (2.6 to 36.6)
Belgium	All risk factors	12.1 (9.9 to 14.3)	316.9 (264.5 to 369)	24.8 (16.4 to 34.4)	341.6 (285.4 to 396.1)	8.3 (6.6 to 10.4)	194.1 (157.9 to 236.7)	25.2 (16 to 37.9)	219.3 (177.5 to 269.1)
Belgium	Behavioral risks	9.9 (8.5 to 11.3)	278.4 (240 to 316.4)	21.5 (14.5 to 29.9)	299.9 (257.2 to 341.3)	6.3 (5.3 to 7.3)	158.5 (135.7 to 185.1)	20.5 (12.9 to 30.4)	179 (151.7 to 209.6)
Belgium	Alcohol use	5.7 (4.6 to 6.8)	158.3 (129.4 to 188.9)	12.2 (8.1 to 17.3)	170.5 (139.2 to 203.3)	3.5 (2.8 to 4.3)	88.6 (70.6 to 108.5)	11.4 (7.1 to 17.5)	100 (79.5 to 123.6)
Belgium	Diet high in red meat	1.6 (0.7 to 2.1)	44.9 (20.9 to 59.4)	3.5 (1.4 to 5.4)	48.3 (22.3 to 64.2)	1.2 (0.6 to 1.7)	31 (15.5 to 42.6)	4 (1.8 to 6.7)	35 (17.3 to 48.5)
Belgium	Low physical activity	0.6 (0.2 to 1.1)	13 (4.3 to 25.6)	1.1 (0.3 to 2.3)	14.1 (4.8 to 28)	0.5 (0.2 to 0.8)	9.1 (2.9 to 17)	1.2 (0.4 to 2.5)	10.3 (3.2 to 19.2)
Belgium	Smoking	2.5 (1.8 to 3.3)	74.9 (51.3 to 98.6)	5.7 (3.2 to 8.7)	80.6 (55.1 to 106.7)	1.5 (1.1 to 1.9)	39.4 (28.6 to 51.2)	5.1 (2.9 to 8)	44.5 (32 to 58.2)
Belgium	Secondhand smoke	0.6 (0.1 to 1.1)	19.2 (4.5 to 33.4)	1.5 (0.3 to 2.8)	20.7 (4.9 to 36.1)	0.3 (0.1 to 0.5)	8.2 (1.9 to 14.2)	1 (0.2 to 2)	9.3 (2.2 to 16.1)
Belgium	Metabolic risks	2.9 (1.1 to 5.5)	53.3 (12.6 to 106.6)	4.5 (1.1 to 9)	57.8 (13.9 to 115.1)	2.8 (1.1 to 5.1)	49.8 (15.1 to 97.9)	6.6 (1.9 to 13.6)	56.4 (17.4 to 109.9)
Belgium	High body-mass index	1.4 (0.3 to 2.9)	21.1 (-3.6 to 51.1)	1.8 (-0.1 to 4.4)	22.9 (-3.6 to 55.1)	1.2 (0.3 to 2.5)	19.3 (0.4 to 43.8)	2.6 (0 to 6)	21.9 (0.4 to 49.4)
Belgium	High fasting plasma glucose	1.6 (0.3 to 3.7)	33.9 (6.5 to 77.9)	2.8 (0.5 to 7.1)	36.8 (7 to 84.4)	1.7 (0.3 to 3.8)	32.6 (6.4 to 73.7)	4.3 (0.8 to 10.9)	36.9 (7.2 to 83.5)
Belize	All risk factors	1.8 (1.1 to 2.7)	42.5 (25.7 to 63.6)	1.8 (1 to 2.9)	44.4 (27 to 65.9)	2.5 (1.5 to 4.1)	59.6 (31.6 to 96.6)	3 (1.4 to 5.1)	62.6 (33 to 100.9)
Belize	Behavioral risks	0.9 (0.7 to 1.2)	25.9 (19.3 to 33.3)	1 (0.7 to 1.5)	26.9 (20.1 to 34.4)	1.1 (0.8 to 1.4)	31.6 (22.9 to 41.7)	1.5 (0.9 to 2.2)	33.1 (24 to 43.3)
Belize	Alcohol use	0.2 (0.2 to 0.3)	7.9 (5.8 to 10.5)	0.3 (0.2 to 0.4)	8.2 (6 to 10.9)	0.3 (0.2 to 0.4)	10.7 (7.5 to 14.8)	0.5 (0.3 to 0.7)	11.2 (7.8 to 15.6)
Belize	Diet high in red meat	0.2 (0 to 0.3)	5.4 (1.1 to 7.7)	0.2 (0 to 0.3)	5.6 (1.1 to 7.9)	0.2 (0 to 0.3)	5.8 (1.1 to 8.7)	0.3 (0.1 to 0.5)	6.1 (1.2 to 9.1)
Belize	Low physical activity	0.2 (0.1 to 0.3)	3.7 (1.3 to 7.1)	0.2 (0.1 to 0.3)	3.8 (1.4 to 7.3)	0.2 (0.1 to 0.4)	5.2 (1.6 to 9.9)	0.3 (0.1 to 0.5)	5.5 (1.7 to 10.4)
Belize	Smoking	0.2 (0.1 to 0.3)	4.4 (2.8 to 6.5)	0.2 (0.1 to 0.3)	4.6 (3 to 6.8)	0.2 (0.1 to 0.3)	4.5 (2.7 to 6.8)	0.2 (0.1 to 0.4)	4.8 (2.9 to 7.1)
Belize	Secondhand smoke	0.2 (0 to 0.3)	5.4 (1.2 to 9.7)	0.2 (0 to 0.4)	5.6 (1.3 to 10)	0.2 (0.1 to 0.4)	6.6 (1.6 to 11.8)	0.3 (0.1 to 0.6)	6.9 (1.6 to 12.3)
Belize	Metabolic risks	1 (0.3 to 1.9)	18.2 (1.8 to 39.4)	0.9 (0.2 to 1.9)	19.1 (1.9 to 40.9)	1.6 (0.5 to 3.2)	30.5 (2.1 to 68)	1.7 (0.2 to 3.6)	32.2 (2.4 to 71.7)
Belize	High body-mass index	0.3 (0 to 0.8)	4.1 (-4.8 to 13.9)	0.3 (-0.1 to 0.7)	4.3 (-4.9 to 14.7)	0.6 (0 to 1.3)	5.2 (-11.5 to 21.7)	0.4 (-0.4 to 1.2)	5.5 (-11.6 to 23.1)
Belize	High fasting plasma glucose	0.7 (0.1 to 1.5)	14.8 (2.8 to 34.2)	0.6 (0.1 to 1.6)	15.4 (3 to 35.7)	1.1 (0.2 to 2.5)	27.2 (5.2 to 62.1)	1.4 (0.3 to 3.3)	28.6 (5.5 to 65.3)
Benin	All risk factors	1.7 (1 to 2.6)	43.9 (25.6 to 66.9)	1.1 (0.6 to 1.8)	45 (26.2 to 68.7)	2.7 (1.5 to 4.4)	64.6 (33.9 to 105.6)	1.9 (0.9 to 3.3)	66.5 (34.8 to 108.8)
Benin	Behavioral risks	1 (0.6 to 1.4)	29.3 (19.1 to 41.5)	0.7 (0.4 to 1)	30 (19.5 to 42.3)	1.2 (0.8 to 1.7)	36.2 (23.4 to 53)	1 (0.6 to 1.5)	37.2 (24.1 to 54.4)
Benin	Alcohol use	0.4 (0.3 to 0.7)	13.9 (8.4 to 20.9)	0.3 (0.2 to 0.5)	14.2 (8.5 to 21.3)	0.6 (0.4 to 1)	20.3 (11.8 to 31.2)	0.5 (0.3 to 0.9)	20.8 (12.1 to 31.9)
Benin	Diet high in red meat	0.1 (0 to 0.2)	4.2 (0.9 to 6.8)	0.1 (0 to 0.2)	4.3 (0.9 to 6.9)	0.2 (0 to 0.3)	4.4 (1 to 7.6)	0.1 (0 to 0.2)	4.5 (1 to 7.8)
Benin	Low physical activity	0.1 (0.1 to 0.2)	2.5 (1.3 to 4.6)	0.1 (0 to 0.1)	2.6 (1.3 to 4.7)	0.1 (0.1 to 0.3)	3.3 (1.6 to 6.3)	0.1 (0 to 0.2)	3.4 (1.7 to 6.5)

Location	Risk factor	1990				2019			
		Deaths	YLLs (Years of Life Lost)	YLDs (Years Lived with Disability)	DALYs (Disability-Adjusted Life Years)	Deaths	YLLs (Years of Life Lost)	YLDs (Years Lived with Disability)	DALYs (Disability-Adjusted Life Years)
Benin	Smoking	0.1 (0 to 0.2)	2.6 (1.1 to 4.4)	0.1 (0 to 0.1)	2.6 (1.1 to 4.5)	0.1 (0 to 0.1)	2.2 (1 to 3.8)	0.1 (0 to 0.1)	2.3 (1.1 to 3.9)
Benin	Secondhand smoke	0.2 (0 to 0.4)	6.9 (1.5 to 12.7)	0.2 (0 to 0.3)	7.1 (1.6 to 13)	0.2 (0 to 0.4)	7 (1.6 to 13.2)	0.2 (0 to 0.4)	7.2 (1.6 to 13.5)
Benin	Metabolic risks	0.8 (0.2 to 1.6)	15.8 (2.4 to 34.7)	0.4 (0.1 to 1)	16.2 (2.5 to 35.6)	1.6 (0.5 to 3.2)	30.6 (5.5 to 66.7)	1 (0.2 to 2.1)	31.6 (5.6 to 68.7)
Benin	High body-mass index	0.3 (0 to 0.7)	4.6 (-2.8 to 14.4)	0.1 (0 to 0.4)	4.7 (-2.8 to 14.7)	0.6 (0.1 to 1.4)	8.7 (-6.8 to 26.3)	0.3 (-0.1 to 0.9)	9 (-7 to 27.1)
Benin	High fasting plasma glucose	0.5 (0.1 to 1.2)	11.5 (2 to 27.1)	0.3 (0.1 to 0.8)	11.8 (2.1 to 27.9)	1.1 (0.2 to 2.5)	23.2 (4.5 to 55.6)	0.7 (0.1 to 1.8)	23.9 (4.6 to 57.5)
Bermuda	All risk factors	9.5 (7 to 12.5)	224.2 (163.9 to 293.7)	13.8 (8.8 to 19.7)	238 (175.7 to 310.6)	5.3 (3.7 to 7.9)	114.7 (77.7 to 167.4)	12.7 (7.8 to 19.8)	127.4 (86.5 to 183.3)
Bermuda	Behavioral risks	6.2 (5 to 7.5)	168.3 (133.4 to 203.7)	10 (6.7 to 14.1)	178.2 (140.2 to 216.1)	3.1 (2.3 to 4.2)	81.1 (59.7 to 108.6)	8.7 (5.6 to 13)	89.8 (66.5 to 120.8)
Bermuda	Alcohol use	2.5 (1.9 to 3.3)	77.4 (58.4 to 100.7)	4.5 (2.9 to 6.6)	81.8 (61.4 to 106.5)	1.1 (0.8 to 1.6)	34.3 (23.4 to 49.1)	3.6 (2.2 to 5.7)	37.9 (25.8 to 54)
Bermuda	Diet high in red meat	1.6 (0.7 to 2.2)	43 (19.5 to 58.7)	2.5 (1.1 to 4)	45.6 (20.6 to 62.4)	0.9 (0.4 to 1.3)	22.5 (10.2 to 33.8)	2.4 (1 to 4)	24.9 (11.4 to 37.4)
Bermuda	Low physical activity	0.7 (0.3 to 1.3)	15.4 (5.4 to 28.6)	1 (0.3 to 2)	16.4 (5.7 to 30.4)	0.4 (0.1 to 0.8)	8.5 (2.7 to 15.8)	1 (0.3 to 1.9)	9.4 (2.9 to 17.7)
Bermuda	Smoking	1.2 (0.8 to 1.7)	29.3 (17.6 to 42.5)	1.8 (1 to 2.8)	31.1 (18.5 to 45.2)	0.6 (0.4 to 0.9)	14.4 (9.3 to 21.7)	1.6 (0.9 to 2.6)	16 (10.3 to 24.3)
Bermuda	Secondhand smoke	0.5 (0.1 to 0.9)	15.3 (3.6 to 26.6)	0.9 (0.2 to 1.7)	16.2 (3.8 to 28.2)	0.3 (0.1 to 0.5)	7.1 (1.6 to 13.2)	0.7 (0.2 to 1.5)	7.8 (1.8 to 14.5)
Bermuda	Metabolic risks	3.9 (1.4 to 7.2)	67.1 (10.9 to 137.1)	4.6 (1.1 to 9)	71.7 (12.3 to 146.2)	2.6 (0.9 to 5)	40 (5.8 to 86)	4.8 (0.9 to 10.1)	44.8 (7 to 95.4)
Bermuda	High body-mass index	2.1 (0.5 to 4.3)	31 (-8.1 to 75.2)	2.3 (-0.1 to 5)	33.3 (-8.3 to 79.6)	1.3 (0.3 to 2.7)	15.2 (-7.5 to 39.9)	2 (-0.5 to 4.9)	17.2 (-7.9 to 44.3)
Bermuda	High fasting plasma glucose	1.9 (0.4 to 4.4)	38.9 (7.2 to 91.8)	2.5 (0.4 to 6.1)	41.4 (7.5 to 96.8)	1.4 (0.3 to 3.4)	27 (5.1 to 65.4)	3.1 (0.6 to 7.7)	30.1 (5.8 to 73.7)
Bhutan	All risk factors	1.3 (0.7 to 2.2)	35.3 (17.7 to 61.4)	0.9 (0.4 to 1.6)	36.1 (18.1 to 63)	1.8 (0.9 to 3.1)	39.9 (18.5 to 74.4)	1.6 (0.7 to 3)	41.4 (19.4 to 76.8)
Bhutan	Behavioral risks	0.7 (0.4 to 1.1)	22.4 (12.5 to 35.8)	0.5 (0.3 to 0.8)	22.9 (12.8 to 36.4)	0.7 (0.4 to 1)	17.4 (10.6 to 27.4)	0.7 (0.4 to 1.1)	18 (10.9 to 28.2)
Bhutan	Alcohol use	0.1 (0.1 to 0.2)	4.9 (2.7 to 7.8)	0.1 (0.1 to 0.2)	5 (2.7 to 7.9)	0.1 (0 to 0.1)	2 (1 to 3.4)	0.1 (0 to 0.1)	2 (1 to 3.5)
Bhutan	Diet high in red meat	0.1 (0 to 0.2)	3.6 (0.8 to 6.3)	0.1 (0 to 0.1)	3.7 (0.9 to 6.4)	0.1 (0 to 0.2)	3.6 (0.8 to 6.2)	0.1 (0 to 0.3)	3.7 (0.8 to 6.5)
Bhutan	Low physical activity	0.2 (0.1 to 0.3)	3.7 (1.7 to 7.1)	0.1 (0 to 0.2)	3.8 (1.8 to 7.2)	0.2 (0.1 to 0.4)	4 (1.9 to 7.5)	0.2 (0.1 to 0.3)	4.2 (1.9 to 7.8)
Bhutan	Smoking	0.1 (0.1 to 0.2)	4 (2.1 to 7.1)	0.1 (0 to 0.2)	4.1 (2.2 to 7.3)	0.1 (0.1 to 0.2)	3 (1.6 to 5)	0.1 (0.1 to 0.2)	3.1 (1.6 to 5.1)
Bhutan	Secondhand smoke	0.2 (0 to 0.4)	6.7 (1.4 to 13.4)	0.1 (0 to 0.3)	6.8 (1.4 to 13.7)	0.2 (0 to 0.3)	5.2 (1.2 to 10)	0.2 (0 to 0.4)	5.4 (1.2 to 10.3)
Bhutan	Metabolic risks	0.6 (0.1 to 1.4)	13.8 (0.9 to 35.2)	0.4 (0.1 to 0.9)	14.1 (1 to 36.2)	1.2 (0.3 to 2.5)	23.8 (4.2 to 55.6)	1 (0.2 to 2.2)	24.8 (4.4 to 57.9)
Bhutan	High body-mass index	0.1 (0 to 0.4)	1.1 (-5.1 to 6.8)	0.1 (-0.1 to 0.2)	1.2 (-5.2 to 7.1)	0.4 (0 to 0.9)	4.7 (-5.8 to 16.5)	0.2 (-0.2 to 0.7)	4.9 (-6 to 17.1)
Bhutan	High fasting plasma glucose	0.5 (0.1 to 1.2)	12.8 (2.2 to 33.1)	0.3 (0.1 to 0.9)	13.2 (2.2 to 33.8)	0.9 (0.2 to 2.1)	19.9 (3.8 to 50.7)	0.8 (0.1 to 2.1)	20.7 (3.9 to 52.7)
Bolivia (Plurinational State of)	All risk factors	2.9 (1.8 to 4.6)	79.5 (48.8 to 121.8)	1.9 (1.1 to 3.1)	81.5 (50.1 to 124.7)	4.1 (2.4 to 6.5)	99.4 (58.1 to 159.3)	3.8 (2 to 6.6)	103.2 (60.5 to 164.9)
Bolivia (Plurinational State of)	Behavioral risks	1.8 (1.2 to 2.6)	58 (38.1 to 83.3)	1.3 (0.8 to 2)	59.3 (39 to 85.3)	2 (1.4 to 2.8)	60.3 (39.7 to 84.7)	2.2 (1.3 to 3.4)	62.5 (41.1 to 87.5)
Bolivia (Plurinational State of)	Alcohol use	0.7 (0.5 to 1.1)	24.7 (15.5 to 36.6)	0.5 (0.3 to 0.9)	25.2 (15.8 to 37.3)	0.9 (0.6 to 1.3)	28.6 (18.1 to 42.8)	1 (0.6 to 1.6)	29.6 (18.8 to 44.3)
Bolivia (Plurinational State of)	Diet high in red meat	0.6 (0.2 to 0.9)	18.7 (6.6 to 28.8)	0.4 (0.1 to 0.7)	19.1 (6.8 to 29.3)	0.7 (0.2 to 1.1)	20 (7.3 to 31)	0.7 (0.3 to 1.3)	20.8 (7.6 to 32.3)
Bolivia (Plurinational State of)	Low physical activity	0.1 (0 to 0.2)	1.8 (1.1 to 5.1)	0 (0 to 0.1)	1.9 (1.2 to 5.2)	0.1 (0.1 to 0.2)	2.2 (1.3 to 5.3)	0.1 (0 to 0.2)	2.3 (1.3 to 5.5)
Bolivia (Plurinational State of)	Smoking	0.3 (0.1 to 0.5)	7.4 (3.3 to 13.3)	0.2 (0.1 to 0.4)	7.6 (3.3 to 13.6)	0.2 (0.1 to 0.4)	5 (2.2 to 8.6)	0.2 (0.1 to 0.3)	5.2 (2.3 to 8.9)
Bolivia (Plurinational State of)	Secondhand smoke	0.2 (0 to 0.5)	8 (1.5 to 14.8)	0.2 (0 to 0.4)	8.2 (1.6 to 15.1)	0.2 (0.1 to 0.4)	7 (1.6 to 13.8)	0.3 (0.1 to 0.5)	7.3 (1.6 to 14.2)
Bolivia (Plurinational State of)	Metabolic risks	1.2 (0.3 to 2.5)	24 (0.6 to 55.8)	0.7 (0.1 to 1.5)	24.7 (0.7 to 57.3)	2.3 (0.8 to 4.4)	43.3 (6.6 to 93.2)	1.8 (0.4 to 3.9)	45.1 (7 to 97)
Bolivia (Plurinational State of)	High body-mass index	0.4 (0 to 1.1)	5.6 (-7.7 to 21.3)	0.2 (-0.1 to 0.6)	5.8 (-7.9 to 21.9)	1 (0.1 to 2.1)	14.5 (-8.4 to 40)	0.7 (-0.2 to 1.7)	15.1 (-8.7 to 41.7)
Bolivia (Plurinational State of)	High fasting plasma glucose	0.8 (0.1 to 1.9)	19.2 (3.4 to 46)	0.5 (0.1 to 1.3)	19.7 (3.5 to 47.3)	1.4 (0.3 to 3.3)	31.1 (6 to 75.9)	1.2 (0.2 to 3.2)	32.3 (6.2 to 78.6)
Bosnia and Herzegovina	All risk factors	2.7 (1.9 to 3.8)	73.1 (50.5 to 98)	3.4 (2.1 to 5.2)	76.5 (52.4 to 102.7)	6 (3.8 to 9.1)	142.5 (92.3 to 214)	9.6 (5.5 to 15.7)	152.1 (98.2 to 227.8)
Bosnia and Herzegovina	Behavioral risks	1.7 (1.3 to 2.1)	54.3 (41.1 to 67.5)	2.4 (1.5 to 3.5)	56.7 (42.6 to 70.4)	3.2 (2.3 to 4.3)	89.8 (63.4 to 122.1)	6.1 (3.7 to 9.4)	95.9 (67.7 to 130.5)
Bosnia and Herzegovina	Alcohol use	0.5 (0.3 to 0.6)	14.7 (9.7 to 20.4)	0.6 (0.4 to 1)	15.4 (10.1 to 21.3)	0.8 (0.6 to 1.2)	24.2 (16.2 to 35.2)	1.7 (1 to 2.6)	25.9 (17.3 to 37.5)
Bosnia and Herzegovina	Diet high in red meat	0.2 (0 to 0.3)	6.3 (1.2 to 8.7)	0.3 (0.1 to 0.5)	6.6 (1.3 to 9)	0.4 (0.1 to 0.7)	11.7 (2.4 to 18.5)	0.8 (0.2 to 1.4)	12.5 (2.6 to 19.8)
Bosnia and Herzegovina	Low physical activity	0.1 (0.1 to 0.2)	2.1 (1.4 to 4.1)	0.1 (0.1 to 0.2)	2.2 (1.4 to 4.3)	0.2 (0.1 to 0.3)	3.4 (2 to 6.1)	0.2 (0.1 to 0.4)	3.6 (2.1 to 6.5)

Location	Risk factor	1990				2019			
		Deaths	YLLs (Years of Life Lost)	YLDs (Years Lived with Disability)	DALYs (Disability-Adjusted Life Years)	Deaths	YLLs (Years of Life Lost)	YLDs (Years Lived with Disability)	DALYs (Disability-Adjusted Life Years)
Bosnia and Herzegovina	Smoking	0.7 (0.5 to 1)	23.2 (15.4 to 32)	1 (0.6 to 1.6)	24.3 (16.1 to 33.4)	1.4 (1 to 2)	41.2 (27 to 59)	2.8 (1.6 to 4.5)	43.9 (29 to 63.3)
Bosnia and Herzegovina	Secondhand smoke	0.3 (0.1 to 0.6)	10.8 (2.6 to 18.7)	0.5 (0.1 to 0.9)	11.3 (2.7 to 19.5)	0.6 (0.1 to 1.1)	15.2 (3.8 to 28.4)	1 (0.2 to 2)	16.3 (4 to 30.3)
Bosnia and Herzegovina	Metabolic risks	1.1 (0.4 to 2.2)	21.5 (2 to 46.2)	1.2 (0.2 to 2.5)	22.7 (2.3 to 48.1)	3.3 (1.2 to 6.5)	63.2 (18.7 to 133.2)	4.2 (1.3 to 8.9)	67.4 (20.1 to 139.9)
Bosnia and Herzegovina	High body-mass index	0.5 (0 to 1)	6 (-6.3 to 18.3)	0.4 (-0.2 to 1.1)	6.4 (-6.6 to 19.3)	1.3 (0.3 to 2.7)	21.1 (-1.1 to 51.2)	1.3 (-0.2 to 3.3)	22.4 (-1.2 to 54.4)
Bosnia and Herzegovina	High fasting plasma glucose	0.7 (0.1 to 1.6)	16.4 (3.1 to 37.8)	0.8 (0.1 to 2)	17.2 (3.2 to 39.4)	2.1 (0.4 to 5)	45.7 (8.8 to 107.9)	3.1 (0.6 to 7.6)	48.8 (9.6 to 115.9)
Botswana	All risk factors	3.7 (2.2 to 5.8)	89.7 (52.1 to 142.2)	2.6 (1.4 to 4.2)	92.2 (53.8 to 145.3)	6.9 (3.8 to 11.4)	154.7 (81.8 to 256.1)	6 (3 to 10.3)	160.6 (84.6 to 266.8)
Botswana	Behavioral risks	2.3 (1.4 to 3.2)	62.4 (38.6 to 91.2)	1.7 (0.9 to 2.7)	64.1 (39.6 to 93.6)	3.3 (2 to 5.2)	90.8 (52 to 146.3)	3.4 (1.8 to 5.6)	94.2 (53.8 to 151.4)
Botswana	Alcohol use	0.6 (0.4 to 0.9)	18.2 (11.5 to 27.9)	0.5 (0.3 to 0.8)	18.7 (11.8 to 28.5)	0.9 (0.5 to 1.5)	28 (15.3 to 46.9)	1 (0.5 to 1.8)	29.1 (15.9 to 48.4)
Botswana	Diet high in red meat	0.5 (0.2 to 0.9)	15.8 (4.5 to 25.9)	0.4 (0.1 to 0.7)	16.3 (4.6 to 26.6)	0.8 (0.2 to 1.4)	23.2 (6.2 to 40.4)	0.9 (0.2 to 1.6)	24 (6.4 to 41.6)
Botswana	Low physical activity	0.2 (0.1 to 0.3)	3.9 (1.9 to 7.5)	0.1 (0.1 to 0.2)	4 (2 to 7.8)	0.3 (0.1 to 0.6)	6.4 (2.9 to 12.4)	0.2 (0.1 to 0.5)	6.6 (3.1 to 12.9)
Botswana	Smoking	0.4 (0.2 to 0.7)	9.1 (4.4 to 15.2)	0.3 (0.1 to 0.5)	9.4 (4.6 to 15.6)	0.5 (0.3 to 0.9)	11.7 (6.1 to 19.7)	0.5 (0.2 to 0.8)	12.2 (6.3 to 20.5)
Botswana	Secondhand smoke	0.6 (0.2 to 1.2)	18.3 (4.4 to 33.9)	0.5 (0.1 to 1)	18.8 (4.5 to 35)	0.9 (0.2 to 1.8)	25.8 (5.8 to 51.1)	1 (0.2 to 2)	26.8 (6 to 52.8)
Botswana	Metabolic risks	1.6 (0.5 to 3.4)	30.9 (5.3 to 68.7)	1 (0.2 to 2.1)	31.9 (5.6 to 70.8)	4 (1.3 to 8.2)	72.2 (14.2 to 162.7)	2.9 (0.7 to 6.3)	75.1 (14.8 to 169)
Botswana	High body-mass index	0.6 (0 to 1.3)	8.8 (-6 to 26.1)	0.3 (-0.1 to 0.8)	9.1 (-6 to 27)	1.6 (0.3 to 3.4)	20.7 (-13.9 to 58.9)	0.9 (-0.4 to 2.4)	21.6 (-14.3 to 60.9)
Botswana	High fasting plasma glucose	1.1 (0.2 to 2.7)	23 (4.2 to 56.8)	0.7 (0.1 to 1.8)	23.7 (4.4 to 58.5)	2.6 (0.5 to 6.2)	55.9 (10.2 to 132.9)	2.2 (0.4 to 5.4)	58.1 (10.6 to 139.3)
Brazil	All risk factors	4.8 (3.6 to 6.1)	125.5 (94.9 to 158.5)	4.6 (3 to 6.4)	130 (98.5 to 164.4)	4.2 (3.1 to 5.5)	105.8 (77.8 to 137.5)	6.1 (3.9 to 8.8)	111.9 (82.5 to 145.5)
Brazil	Behavioral risks	3.4 (2.8 to 4)	99.1 (80 to 116.4)	3.5 (2.4 to 4.8)	102.6 (82.4 to 121.4)	2.8 (2.3 to 3.4)	81.3 (66.2 to 97)	4.5 (3.1 to 6.3)	85.8 (69.6 to 102.1)
Brazil	Alcohol use	0.7 (0.5 to 0.8)	22.4 (17.1 to 28.1)	0.8 (0.5 to 1.1)	23.1 (17.7 to 29.1)	0.7 (0.6 to 0.9)	24.8 (19.3 to 30.9)	1.3 (0.9 to 1.9)	26.2 (20.3 to 32.5)
Brazil	Diet high in red meat	0.7 (0.3 to 0.9)	20.5 (8.8 to 27)	0.7 (0.3 to 1.1)	21.2 (9.1 to 27.9)	0.8 (0.4 to 1.2)	24.3 (12.3 to 34)	1.3 (0.7 to 2.1)	25.7 (12.9 to 35.9)
Brazil	Low physical activity	0.5 (0.2 to 0.8)	12.1 (4 to 21.6)	0.4 (0.2 to 0.8)	12.6 (4.2 to 22.4)	0.5 (0.2 to 0.8)	11 (3.9 to 18.9)	0.6 (0.2 to 1.2)	11.7 (4.1 to 20)
Brazil	Smoking	1.4 (1 to 1.8)	39.1 (28.1 to 51.5)	1.4 (0.9 to 2.1)	40.6 (29.3 to 53.5)	0.7 (0.5 to 1)	20 (13.9 to 26.8)	1.2 (0.7 to 1.7)	21.1 (14.6 to 28.3)
Brazil	Secondhand smoke	0.5 (0.1 to 0.8)	13.3 (3.1 to 22.9)	0.5 (0.1 to 0.9)	13.7 (3.2 to 23.8)	0.3 (0.1 to 0.5)	7.7 (1.7 to 13.6)	0.4 (0.1 to 0.8)	8.2 (1.8 to 14.3)
Brazil	Metabolic risks	1.7 (0.6 to 3.3)	33 (5.8 to 70.3)	1.3 (0.3 to 2.8)	34.3 (6.1 to 73.1)	1.7 (0.6 to 3.2)	30.5 (3.6 to 65.2)	2 (0.4 to 4.1)	32.4 (4.1 to 69.1)
Brazil	High body-mass index	0.6 (0 to 1.2)	7 (-6.1 to 21.3)	0.4 (-0.1 to 0.9)	7.4 (-6.2 to 22.1)	0.7 (0.1 to 1.5)	7.3 (-8.4 to 22.7)	0.6 (-0.3 to 1.6)	7.9 (-8.7 to 24.2)
Brazil	High fasting plasma glucose	1.2 (0.2 to 2.6)	27.1 (5.2 to 61.1)	1 (0.2 to 2.5)	28.1 (5.4 to 63.5)	1.1 (0.2 to 2.4)	24.7 (4.6 to 55.5)	1.5 (0.3 to 3.5)	26.2 (4.9 to 59)
Brunei Darussalam	All risk factors	4.4 (2.6 to 6.9)	129.8 (78.6 to 200.5)	6.9 (3.7 to 11.5)	136.6 (83.2 to 211.1)	4.9 (2.5 to 8.2)	127 (65.9 to 211.9)	9.4 (4.6 to 16.8)	136.4 (71 to 227.8)
Brunei Darussalam	Behavioral risks	2.5 (1.7 to 3.3)	82.6 (57.9 to 110.7)	4.1 (2.6 to 6.1)	86.7 (60.8 to 116.4)	1.8 (1.1 to 2.4)	53.1 (33.6 to 72.8)	3.8 (2.2 to 5.7)	56.9 (36.2 to 78.1)
Brunei Darussalam	Alcohol use	0.7 (0.5 to 1.1)	26.5 (16.7 to 39.8)	1.3 (0.7 to 2.1)	27.8 (17.6 to 41.6)	0.2 (0 to 0.4)	6.9 (0 to 15.1)	0.5 (0 to 1.1)	7.4 (0 to 16.1)
Brunei Darussalam	Diet high in red meat	0.6 (0.2 to 0.9)	22 (7.4 to 32.1)	1.1 (0.4 to 1.8)	23.1 (7.8 to 33.7)	0.5 (0.1 to 0.8)	16.4 (4 to 25)	1.2 (0.3 to 2)	17.6 (4.3 to 26.6)
Brunei Darussalam	Low physical activity	0.2 (0.1 to 0.4)	5.3 (2.1 to 11.2)	0.3 (0.1 to 0.7)	5.6 (2.2 to 11.8)	0.3 (0.1 to 0.5)	6.7 (2.5 to 14.6)	0.5 (0.2 to 1.1)	7.2 (2.7 to 15.7)
Brunei Darussalam	Smoking	0.5 (0.4 to 0.8)	16.3 (10.3 to 24.3)	0.9 (0.5 to 1.4)	17.2 (10.9 to 25.6)	0.4 (0.3 to 0.6)	11.3 (7.4 to 16.5)	0.8 (0.5 to 1.3)	12.2 (7.9 to 17.7)
Brunei Darussalam	Secondhand smoke	0.5 (0.1 to 0.9)	16.9 (3.5 to 31)	0.8 (0.2 to 1.5)	17.7 (3.7 to 32.5)	0.4 (0.1 to 0.7)	13.4 (2.8 to 24.5)	0.9 (0.2 to 1.8)	14.4 (3.1 to 26.3)
Brunei Darussalam	Metabolic risks	2.2 (0.5 to 4.6)	53.9 (10.1 to 119.7)	3.2 (0.7 to 7.2)	57.1 (10.7 to 126.9)	3.4 (1.1 to 6.8)	80.2 (22 to 167.6)	6.1 (1.6 to 13.1)	86.2 (23.9 to 179.7)
Brunei Darussalam	High body-mass index	0.2 (-0.1 to 0.6)	1 (-8.4 to 9.8)	0.2 (-0.3 to 0.7)	1.1 (-8.6 to 10.6)	0.6 (0.1 to 1.3)	8.7 (-4.9 to 25.7)	0.7 (-0.3 to 1.8)	9.3 (-5.1 to 27.4)
Brunei Darussalam	High fasting plasma glucose	2.1 (0.4 to 4.5)	53.9 (11.2 to 120.7)	3.1 (0.6 to 7.1)	57 (11.9 to 127.2)	3 (0.6 to 6.4)	74.1 (15.4 to 161.1)	5.6 (1.1 to 12.7)	79.7 (16.6 to 174.5)
Bulgaria	All risk factors	6.1 (4.7 to 7.5)	179.5 (140.7 to 219)	11.3 (7.3 to 16.1)	190.8 (150.6 to 234.2)	7.5 (5.3 to 10.5)	205.8 (146.8 to 287.2)	17.1 (10.6 to 26.3)	222.8 (157.7 to 311.4)
Bulgaria	Behavioral risks	4.7 (3.9 to 5.5)	155.3 (128.9 to 181.2)	9.5 (6.3 to 13.3)	164.8 (136.5 to 193.1)	5.4 (4 to 7.1)	163.8 (119.1 to 220)	13.3 (8.3 to 20)	177.2 (129.2 to 237.3)
Bulgaria	Alcohol use	1.9 (1.5 to 2.3)	63.5 (49.8 to 77.8)	3.9 (2.5 to 5.6)	67.4 (52.8 to 82.5)	2.3 (1.6 to 3.2)	72.3 (50.1 to 100.5)	5.9 (3.5 to 9)	78.2 (54.5 to 108.7)
Bulgaria	Diet high in red meat	1 (0.5 to 1.3)	31.2 (15.4 to 42.4)	1.9 (0.8 to 3)	33.1 (16.2 to 44.9)	1.2 (0.6 to 1.8)	34.6 (16.9 to 52.7)	2.8 (1.2 to 4.8)	37.5 (18.1 to 57.1)
Bulgaria	Low physical activity	0.2 (0.1 to 0.3)	4.7 (2.8 to 8.4)	0.3 (0.2 to 0.6)	5 (2.9 to 8.9)	0.2 (0.1 to 0.4)	5.4 (2.8 to 9.3)	0.5 (0.2 to 0.9)	5.9 (3.1 to 10.1)

Location	Risk factor	1990				2019			
		Deaths	YLLs (Years of Life Lost)	YLDs (Years Lived with Disability)	DALYs (Disability-Adjusted Life Years)	Deaths	YLLs (Years of Life Lost)	YLDs (Years Lived with Disability)	DALYs (Disability-Adjusted Life Years)
Bulgaria	Smoking	1.6 (1.1 to 2.2)	55 (36.8 to 75)	3.3 (1.9 to 5.2)	58.3 (38.8 to 79.1)	1.6 (1.1 to 2.3)	52.3 (33.9 to 76.7)	4.2 (2.3 to 6.8)	56.5 (36.5 to 82.6)
Bulgaria	Secondhand smoke	0.6 (0.1 to 1)	17.8 (4.3 to 31)	1.1 (0.3 to 2)	18.9 (4.6 to 33.2)	0.6 (0.1 to 1.1)	17.1 (4.3 to 31.3)	1.4 (0.3 to 2.7)	18.5 (4.5 to 34)
Bulgaria	Metabolic risks	1.7 (0.5 to 3.3)	31.5 (-1 to 70.5)	2.3 (0.2 to 5)	33.8 (-0.7 to 74.8)	2.7 (1 to 5.2)	55 (13.5 to 114)	4.9 (1.3 to 10.3)	59.9 (14.7 to 123.2)
Bulgaria	High body-mass index	0.8 (0 to 1.8)	9.9 (-13.6 to 32.4)	0.8 (-0.6 to 2.4)	10.7 (-14.2 to 34.7)	1.3 (0.3 to 2.7)	20.9 (-5.4 to 51.7)	1.9 (-0.3 to 4.6)	22.8 (-5.6 to 55.8)
Bulgaria	High fasting plasma glucose	0.9 (0.2 to 2.2)	23.1 (4.2 to 54)	1.5 (0.3 to 3.8)	24.6 (4.5 to 57.3)	1.6 (0.3 to 3.8)	36.7 (6.9 to 88.2)	3.2 (0.6 to 8.1)	39.9 (7.6 to 96.2)
Burkina Faso	All risk factors	3.5 (2.3 to 5.1)	90.4 (59 to 126.2)	2.1 (1.3 to 3.3)	92.5 (60.3 to 129.5)	4.9 (3.4 to 6.9)	120.9 (81.3 to 168.7)	3.4 (2 to 5.3)	124.3 (84 to 173.3)
Burkina Faso	Behavioral risks	2.6 (1.8 to 3.5)	73 (51 to 99.1)	1.7 (1 to 2.5)	74.6 (52.1 to 101)	3.3 (2.3 to 4.4)	91.5 (63.9 to 124.4)	2.5 (1.5 to 3.7)	94 (65.5 to 127.8)
Burkina Faso	Alcohol use	1.8 (1.3 to 2.5)	52.3 (36 to 71.6)	1.2 (0.7 to 1.8)	53.5 (36.8 to 73.2)	2.4 (1.6 to 3.3)	67.6 (44.6 to 94.4)	1.8 (1.1 to 2.9)	69.4 (45.8 to 96.9)
Burkina Faso	Diet high in red meat	0.3 (0.1 to 0.4)	8.3 (1.5 to 12.9)	0.2 (0 to 0.3)	8.4 (1.6 to 13.2)	0.4 (0.1 to 0.6)	11.6 (2.3 to 18.2)	0.3 (0.1 to 0.5)	11.9 (2.4 to 18.7)
Burkina Faso	Low physical activity	0.1 (0.1 to 0.2)	2.7 (1.6 to 5.4)	0.1 (0 to 0.1)	2.8 (1.6 to 5.5)	0.2 (0.1 to 0.3)	3.3 (1.9 to 6.3)	0.1 (0 to 0.2)	3.4 (1.9 to 6.5)
Burkina Faso	Smoking	0 (0 to 0.1)	1.1 (0.4 to 1.8)	0 (0 to 0)	1.1 (0.4 to 1.9)	0 (0 to 0.1)	1.1 (0.5 to 2)	0 (0 to 0.1)	1.1 (0.5 to 2)
Burkina Faso	Secondhand smoke	0.4 (0.1 to 0.7)	11.4 (2.7 to 21.2)	0.3 (0.1 to 0.5)	11.7 (2.7 to 21.7)	0.4 (0.1 to 0.7)	11.8 (2.7 to 21.2)	0.3 (0.1 to 0.6)	12.1 (2.7 to 21.9)
Burkina Faso	Metabolic risks	1.1 (0.3 to 2.3)	20.3 (4.4 to 45.2)	0.6 (0.1 to 1.3)	20.9 (4.5 to 46.3)	1.9 (0.6 to 3.8)	35 (5.6 to 77.3)	1.1 (0.3 to 2.4)	36 (5.9 to 79.6)
Burkina Faso	High body-mass index	0.2 (0 to 0.6)	3.2 (-2.1 to 10.2)	0.1 (0 to 0.3)	3.3 (-2.2 to 10.5)	0.7 (0.1 to 1.6)	8.9 (-6.3 to 27.6)	0.3 (-0.1 to 0.8)	9.2 (-6.3 to 28.6)
Burkina Faso	High fasting plasma glucose	0.9 (0.2 to 2)	17.4 (3.1 to 41.4)	0.5 (0.1 to 1.2)	17.9 (3.2 to 42.6)	1.3 (0.2 to 3)	27.3 (5.1 to 63.5)	0.8 (0.1 to 2)	28.1 (5.2 to 65.2)
Burundi	All risk factors	2.3 (1.4 to 3.6)	64.5 (39.7 to 99.5)	1.4 (0.8 to 2.3)	65.9 (40.5 to 101.6)	1.9 (1 to 3.4)	48.6 (26.7 to 85.2)	1.3 (0.7 to 2.4)	49.9 (27.3 to 87.6)
Burundi	Behavioral risks	1.6 (1.1 to 2.3)	50.8 (32.3 to 74)	1.1 (0.6 to 1.7)	51.8 (32.9 to 75.5)	1 (0.7 to 1.6)	31.2 (19.6 to 48.3)	0.8 (0.4 to 1.3)	32 (20.2 to 49.4)
Burundi	Alcohol use	1.1 (0.7 to 1.6)	34.5 (21.4 to 51.1)	0.7 (0.4 to 1.1)	35.2 (21.8 to 52.1)	0.6 (0.3 to 0.9)	17.8 (10.8 to 28.7)	0.4 (0.2 to 0.7)	18.3 (11 to 29.4)
Burundi	Diet high in red meat	0.1 (0 to 0.2)	3.4 (1.1 to 5.6)	0.1 (0 to 0.1)	3.5 (1.2 to 5.7)	0.1 (0 to 0.2)	2.6 (1 to 4.4)	0.1 (0 to 0.1)	2.7 (1 to 4.5)
Burundi	Low physical activity	0.1 (0 to 0.2)	2 (1.2 to 5.1)	0 (0 to 0.1)	2.1 (1.2 to 5.1)	0.1 (0 to 0.2)	1.8 (1 to 4.3)	0 (0 to 0.1)	1.9 (1.1 to 4.4)
Burundi	Smoking	0.2 (0.1 to 0.3)	4.4 (1.8 to 8.1)	0.1 (0 to 0.2)	4.5 (1.8 to 8.2)	0.1 (0.1 to 0.2)	3.3 (1.3 to 6.1)	0.1 (0 to 0.2)	3.4 (1.3 to 6.2)
Burundi	Secondhand smoke	0.2 (0 to 0.4)	7.8 (1.5 to 14.7)	0.2 (0 to 0.3)	7.9 (1.5 to 15)	0.2 (0 to 0.4)	6.3 (1.5 to 12.6)	0.2 (0 to 0.3)	6.4 (1.5 to 12.9)
Burundi	Metabolic risks	0.7 (0.2 to 1.7)	15.2 (1.3 to 38.4)	0.4 (0.1 to 1)	15.6 (1.3 to 39.5)	0.9 (0.2 to 2.1)	18.6 (2.8 to 45.4)	0.5 (0.1 to 1.3)	19.1 (2.9 to 46.7)
Burundi	High body-mass index	0.1 (0 to 0.4)	1.2 (-4 to 7.7)	0.1 (0 to 0.2)	1.2 (-4.1 to 7.9)	0.2 (0 to 0.6)	2.7 (-3.1 to 10.3)	0.1 (0 to 0.3)	2.8 (-3.1 to 10.7)
Burundi	High fasting plasma glucose	0.6 (0.1 to 1.5)	14.1 (2.3 to 36.4)	0.3 (0.1 to 0.9)	14.5 (2.4 to 37.3)	0.7 (0.1 to 1.8)	16.1 (2.7 to 42.5)	0.4 (0.1 to 1.2)	16.6 (2.8 to 43.5)
Cabo Verde	All risk factors	2.2 (1.5 to 3.1)	55.8 (37.2 to 77.9)	1.7 (1 to 2.6)	57.5 (38.3 to 80.1)	3.4 (2 to 5.4)	73 (44.3 to 114.5)	3.2 (1.8 to 5.4)	76.2 (46.4 to 119.5)
Cabo Verde	Behavioral risks	1.4 (1.1 to 1.9)	43.5 (32.3 to 56.4)	1.2 (0.8 to 1.8)	44.7 (33.1 to 58.2)	1.6 (1.2 to 2.2)	43.7 (30.9 to 58.3)	1.9 (1.2 to 2.8)	45.6 (32.5 to 60.4)
Cabo Verde	Alcohol use	0.8 (0.6 to 1)	24.2 (17.3 to 32.6)	0.7 (0.4 to 1)	24.9 (17.8 to 33.6)	0.9 (0.6 to 1.3)	25.4 (17.8 to 35.4)	1.1 (0.7 to 1.7)	26.5 (18.6 to 37)
Cabo Verde	Diet high in red meat	0.2 (0 to 0.3)	7 (1.3 to 10.4)	0.2 (0 to 0.3)	7.2 (1.4 to 10.7)	0.3 (0.1 to 0.4)	7.1 (1.4 to 10.9)	0.3 (0.1 to 0.5)	7.4 (1.5 to 11.4)
Cabo Verde	Low physical activity	0.1 (0.1 to 0.2)	2.6 (1.5 to 4.7)	0.1 (0 to 0.2)	2.7 (1.5 to 4.8)	0.2 (0.1 to 0.3)	3.1 (1.6 to 5.5)	0.1 (0.1 to 0.3)	3.2 (1.7 to 5.7)
Cabo Verde	Smoking	0.1 (0.1 to 0.2)	3.5 (1.6 to 5.9)	0.1 (0 to 0.2)	3.6 (1.7 to 6)	0.1 (0.1 to 0.2)	2.9 (1.4 to 4.7)	0.1 (0.1 to 0.2)	3 (1.5 to 4.9)
Cabo Verde	Secondhand smoke	0.2 (0.1 to 0.4)	7.7 (1.8 to 13.9)	0.2 (0 to 0.4)	7.9 (1.8 to 14.4)	0.2 (0.1 to 0.4)	6.9 (1.5 to 12.2)	0.3 (0.1 to 0.6)	7.2 (1.6 to 12.8)
Cabo Verde	Metabolic risks	0.8 (0.2 to 1.7)	13.8 (-0.6 to 33.2)	0.5 (0.1 to 1.1)	14.3 (-0.4 to 34.1)	1.9 (0.6 to 3.9)	32.9 (5.6 to 71.8)	1.5 (0.3 to 3.3)	34.4 (5.9 to 74.8)
Cabo Verde	High body-mass index	0.2 (0 to 0.6)	1.5 (-7.2 to 9.2)	0.1 (-0.1 to 0.4)	1.6 (-7.4 to 9.4)	0.6 (0.1 to 1.4)	7.6 (-5.2 to 23.3)	0.4 (-0.2 to 1.1)	8 (-5.4 to 24.4)
Cabo Verde	High fasting plasma glucose	0.6 (0.1 to 1.4)	12.7 (2.4 to 29.1)	0.4 (0.1 to 1)	13.2 (2.4 to 30.2)	1.4 (0.3 to 3.2)	26.8 (5.2 to 62.5)	1.2 (0.2 to 2.9)	28 (5.4 to 65.4)
Cambodia	All risk factors	1.5 (0.9 to 2.5)	47.1 (26.6 to 74.9)	1.2 (0.6 to 2)	48.2 (27.3 to 76.6)	3.4 (2.1 to 5.1)	96.6 (58.7 to 142.1)	3.3 (1.8 to 5.2)	99.9 (61.2 to 146.6)
Cambodia	Behavioral risks	0.9 (0.5 to 1.4)	29.7 (16.8 to 45.4)	0.7 (0.4 to 1.1)	30.4 (17.2 to 46.5)	1.6 (1.1 to 2.2)	52.3 (34.2 to 73)	1.7 (1 to 2.7)	54.1 (35.4 to 75.4)
Cambodia	Alcohol use	0.1 (0 to 0.2)	3.8 (1.6 to 6.7)	0.1 (0 to 0.2)	3.9 (1.7 to 6.9)	0.7 (0.4 to 0.9)	23.2 (14.8 to 33.7)	0.8 (0.4 to 1.2)	24 (15.2 to 34.9)
Cambodia	Diet high in red meat	0.2 (0 to 0.3)	6.1 (1.1 to 9.8)	0.1 (0 to 0.2)	6.2 (1.1 to 10)	0.3 (0.1 to 0.4)	8.1 (1.6 to 12.8)	0.3 (0.1 to 0.5)	8.4 (1.7 to 13.3)
Cambodia	Low physical activity	0.1 (0 to 0.1)	1.6 (0.9 to 3.9)	0 (0 to 0.1)	1.6 (0.9 to 4)	0.1 (0 to 0.2)	2 (1.2 to 4.3)	0.1 (0 to 0.1)	2.1 (1.2 to 4.5)
Cambodia	Smoking	0.2 (0.1 to 0.3)	4.4 (2.2 to 7)	0.1 (0.1 to 0.2)	4.5 (2.3 to 7.2)	0.2 (0.1 to 0.3)	4.7 (2.5 to 7.5)	0.2 (0.1 to 0.3)	4.9 (2.6 to 7.7)

Location	Risk factor	1990				2019			
		Deaths	YLLs (Years of Life Lost)	YLDs (Years Lived with Disability)	DALYs (Disability-Adjusted Life Years)	Deaths	YLLs (Years of Life Lost)	YLDs (Years Lived with Disability)	DALYs (Disability-Adjusted Life Years)
Cambodia	Secondhand smoke	0.4 (0.1 to 0.8)	14.7 (3.4 to 26.9)	0.3 (0.1 to 0.7)	15 (3.5 to 27.5)	0.5 (0.1 to 0.9)	16.6 (3.8 to 30.1)	0.6 (0.1 to 1.1)	17.2 (4 to 31.1)
Cambodia	Metabolic risks	0.7 (0.2 to 1.5)	18.9 (5.5 to 40)	0.5 (0.1 to 1.1)	19.4 (5.6 to 40.9)	2 (0.8 to 3.6)	50.1 (18.9 to 91.4)	1.7 (0.6 to 3.2)	51.8 (19.6 to 94.4)
Cambodia	High body-mass index	0.4 (0.1 to 1)	10.7 (2 to 28.1)	0.3 (0 to 0.7)	11 (2.1 to 28.9)	1.1 (0.3 to 2.3)	28.6 (7.6 to 60.4)	1 (0.2 to 2.2)	29.6 (7.8 to 62.2)
Cambodia	High fasting plasma glucose	0.3 (0.1 to 0.8)	8.6 (1.4 to 20.6)	0.2 (0 to 0.6)	8.8 (1.4 to 21.1)	1 (0.2 to 2.2)	23.7 (4.3 to 55.2)	0.8 (0.2 to 2.1)	24.5 (4.4 to 57.2)
Cameroon	All risk factors	3.1 (2 to 4.4)	77.1 (48.6 to 111.2)	1.9 (1.1 to 3)	79 (50 to 113.9)	4.5 (2.6 to 7.2)	105.9 (60.3 to 169.8)	3.2 (1.7 to 5.4)	109.1 (61.8 to 174.9)
Cameroon	Behavioral risks	1.9 (1.4 to 2.6)	57.5 (39.9 to 77.4)	1.3 (0.8 to 1.9)	58.8 (41.1 to 79.3)	2.4 (1.6 to 3.5)	69.9 (44.5 to 105)	2 (1.1 to 3.1)	71.9 (45.6 to 107.2)
Cameroon	Alcohol use	1.2 (0.8 to 1.6)	35.6 (24.3 to 48.8)	0.8 (0.5 to 1.2)	36.4 (24.9 to 50)	1.5 (0.9 to 2.3)	45.4 (27.5 to 69.2)	1.3 (0.7 to 2.1)	46.7 (28.2 to 71.5)
Cameroon	Diet high in red meat	0.3 (0.1 to 0.5)	9.6 (1.8 to 14.8)	0.2 (0 to 0.4)	9.8 (1.8 to 15.1)	0.4 (0.1 to 0.6)	10.1 (1.9 to 18.1)	0.3 (0 to 0.5)	10.4 (1.9 to 18.7)
Cameroon	Low physical activity	0.2 (0.1 to 0.3)	3.6 (1.9 to 6.6)	0.1 (0 to 0.2)	3.7 (2 to 6.7)	0.2 (0.1 to 0.4)	4.9 (2.3 to 9.5)	0.2 (0.1 to 0.3)	5.1 (2.4 to 9.7)
Cameroon	Smoking	0.1 (0 to 0.1)	1.5 (0.7 to 2.6)	0 (0 to 0.1)	1.5 (0.7 to 2.6)	0.1 (0 to 0.1)	1.5 (0.7 to 2.8)	0 (0 to 0.1)	1.6 (0.7 to 2.9)
Cameroon	Secondhand smoke	0.3 (0.1 to 0.5)	9.3 (2 to 17.1)	0.2 (0 to 0.4)	9.6 (2 to 17.4)	0.3 (0.1 to 0.6)	10.2 (2.2 to 19.7)	0.3 (0.1 to 0.6)	10.5 (2.3 to 20.3)
Cameroon	Metabolic risks	1.3 (0.4 to 2.5)	21.9 (0.7 to 50.5)	0.7 (0.1 to 1.4)	22.6 (0.8 to 51.8)	2.3 (0.8 to 4.6)	40.3 (4.5 to 89.5)	1.4 (0.3 to 2.9)	41.6 (4.8 to 92.3)
Cameroon	High body-mass index	0.7 (0 to 1.5)	9.2 (-7.3 to 30.1)	0.3 (-0.1 to 0.8)	9.5 (-7.3 to 30.9)	1.1 (0.1 to 2.4)	13.4 (-11 to 41.1)	0.5 (-0.2 to 1.4)	13.9 (-11.2 to 42.7)
Cameroon	High fasting plasma glucose	0.6 (0.1 to 1.4)	13.5 (2.5 to 32.1)	0.4 (0.1 to 0.9)	13.8 (2.5 to 32.8)	1.3 (0.3 to 3.3)	28.9 (5.6 to 70.5)	0.9 (0.2 to 2.3)	29.8 (5.7 to 72.4)
Canada	All risk factors	8.4 (6.9 to 9.9)	220.1 (184.3 to 255.7)	22.9 (15.5 to 32.6)	243.1 (203.1 to 283.4)	5.8 (4.5 to 7.3)	135.8 (107.3 to 166.8)	21.1 (13.2 to 31.9)	156.9 (123.4 to 193.8)
Canada	Behavioral risks	6.9 (5.9 to 7.8)	193.1 (166.4 to 219)	19.7 (13.3 to 27.7)	212.8 (181.9 to 241.6)	4.1 (3.4 to 4.8)	107.6 (90.2 to 124.6)	16.2 (10.3 to 23.8)	123.8 (104 to 144.8)
Canada	Alcohol use	3.1 (2.5 to 3.8)	90.3 (73 to 108.9)	9.1 (6 to 13.3)	99.4 (79.7 to 119.7)	2.1 (1.6 to 2.5)	57.3 (45.8 to 69.6)	8.5 (5.3 to 12.8)	65.8 (52 to 80.7)
Canada	Diet high in red meat	1.3 (0.7 to 1.8)	37.5 (19 to 50.1)	3.8 (1.7 to 6.1)	41.3 (21.1 to 55.7)	0.8 (0.4 to 1.2)	21.7 (10.6 to 29.8)	3.3 (1.4 to 5.4)	25 (11.9 to 34.7)
Canada	Low physical activity	0.3 (0.1 to 0.7)	7.6 (2.9 to 15.5)	0.8 (0.3 to 1.9)	8.4 (3.2 to 17.3)	0.3 (0.1 to 0.5)	5.7 (2 to 11.2)	0.9 (0.3 to 2)	6.6 (2.3 to 13.1)
Canada	Smoking	2.4 (1.7 to 3.2)	67.8 (50.1 to 86.8)	7 (4.2 to 10.4)	74.8 (54.6 to 96)	1.1 (0.8 to 1.4)	27.5 (19.8 to 36)	4.2 (2.4 to 6.7)	31.8 (22.4 to 41.8)
Canada	Secondhand smoke	0.4 (0.1 to 0.7)	11.8 (2.7 to 20.9)	1.2 (0.3 to 2.2)	12.9 (2.9 to 22.9)	0.2 (0 to 0.3)	5.4 (1.2 to 9.6)	0.8 (0.2 to 1.5)	6.2 (1.4 to 11.1)
Canada	Metabolic risks	2.1 (0.8 to 3.8)	36.5 (5.8 to 74.2)	4.4 (1.1 to 8.8)	40.9 (6.9 to 81.3)	2.1 (0.8 to 3.8)	37 (9.4 to 71.6)	6.3 (1.8 to 12.5)	43.3 (11 to 83.3)
Canada	High body-mass index	1.2 (0.2 to 2.5)	18.8 (-4.2 to 46.7)	2.3 (-0.1 to 5.4)	21.1 (-4.4 to 51.5)	1.2 (0.3 to 2.3)	17.2 (-2 to 39.5)	3.1 (0.1 to 6.8)	20.3 (-1.7 to 45.9)
Canada	High fasting plasma glucose	0.9 (0.2 to 2.1)	18.9 (3.4 to 43.4)	2.1 (0.4 to 5.3)	21.1 (3.8 to 48)	1.1 (0.2 to 2.4)	21.4 (4 to 49.1)	3.5 (0.6 to 8.8)	24.9 (4.6 to 57.4)
Central African Republic	All risk factors	3.2 (2 to 4.9)	89.9 (57.5 to 134.6)	1.9 (1.1 to 3.1)	91.8 (59.2 to 137.5)	3.6 (1.9 to 6.5)	96.9 (50.9 to 172.7)	2.1 (1.1 to 3.9)	99 (52.3 to 176.5)
Central African Republic	Behavioral risks	2.3 (1.6 to 3.2)	70.2 (48.8 to 98)	1.4 (0.9 to 2.2)	71.6 (49.8 to 99.7)	2.3 (1.3 to 3.8)	66.7 (37.6 to 114.3)	1.4 (0.7 to 2.5)	68.1 (38.5 to 116.4)
Central African Republic	Alcohol use	1.2 (0.8 to 1.7)	37.9 (24.6 to 54.1)	0.8 (0.4 to 1.2)	38.6 (25.1 to 55.3)	1 (0.5 to 1.7)	30.3 (15.6 to 54.1)	0.6 (0.3 to 1.1)	30.9 (15.9 to 54.9)
Central African Republic	Diet high in red meat	0.6 (0.2 to 0.9)	17.8 (5.8 to 27.2)	0.4 (0.1 to 0.6)	18.2 (5.9 to 27.7)	0.7 (0.3 to 1.4)	22.2 (8 to 41.8)	0.5 (0.2 to 0.9)	22.6 (8.1 to 42.6)
Central African Republic	Low physical activity	0.2 (0.1 to 0.4)	5.3 (2.5 to 10.2)	0.1 (0.1 to 0.2)	5.4 (2.5 to 10.5)	0.2 (0.1 to 0.5)	5.6 (2.3 to 11.3)	0.1 (0.1 to 0.3)	5.7 (2.4 to 11.5)
Central African Republic	Smoking	0.1 (0.1 to 0.2)	3.1 (1.5 to 5.2)	0.1 (0 to 0.1)	3.2 (1.5 to 5.3)	0.1 (0.1 to 0.2)	2.6 (1.2 to 5.1)	0.1 (0 to 0.1)	2.7 (1.3 to 5.2)
Central African Republic	Secondhand smoke	0.3 (0.1 to 0.5)	9.1 (1.9 to 17.7)	0.2 (0 to 0.4)	9.3 (1.9 to 18.1)	0.3 (0.1 to 0.6)	8.8 (1.7 to 19.4)	0.2 (0 to 0.4)	9 (1.8 to 19.8)
Central African Republic	Metabolic risks	1 (0.2 to 2.3)	22.4 (3.5 to 53.5)	0.5 (0.1 to 1.3)	23 (3.6 to 54.7)	1.5 (0.4 to 3.7)	34 (6.3 to 88.8)	0.8 (0.2 to 2.1)	34.8 (6.5 to 90.8)
Central African Republic	High body-mass index	0.2 (0 to 0.5)	2.1 (-2.9 to 8.6)	0.1 (0 to 0.2)	2.1 (-3 to 8.9)	0.3 (0 to 0.7)	3.5 (-3.9 to 12.5)	0.1 (0 to 0.4)	3.6 (-3.9 to 12.8)
Central African Republic	High fasting plasma glucose	0.9 (0.2 to 2.1)	20.6 (3.7 to 52.4)	0.5 (0.1 to 1.2)	21.1 (3.7 to 53.7)	1.3 (0.2 to 3.5)	30.9 (5.8 to 85.1)	0.7 (0.1 to 1.9)	31.7 (6 to 86.8)
Chad	All risk factors	1.5 (0.8 to 2.3)	39.9 (22.7 to 63.6)	0.9 (0.5 to 1.6)	40.8 (23.2 to 64.8)	2.5 (1.4 to 4)	63.8 (35.6 to 102.1)	1.6 (0.8 to 2.8)	65.4 (36.6 to 104.5)
Chad	Behavioral risks	1 (0.6 to 1.5)	29.7 (17 to 45.3)	0.7 (0.4 to 1.1)	30.4 (17.4 to 46.3)	1.4 (0.8 to 2.1)	41.6 (24.2 to 63.5)	1 (0.5 to 1.7)	42.7 (24.8 to 64.9)
Chad	Alcohol use	0.4 (0.2 to 0.7)	13.2 (5.4 to 23.7)	0.3 (0.1 to 0.5)	13.5 (5.4 to 24.1)	0.7 (0.3 to 1.2)	22.2 (10.5 to 36.4)	0.5 (0.2 to 1)	22.8 (10.7 to 37.2)
Chad	Diet high in red meat	0.2 (0.1 to 0.4)	7.2 (1.5 to 11.6)	0.2 (0 to 0.3)	7.4 (1.6 to 11.8)	0.3 (0.1 to 0.5)	8.2 (1.6 to 13.8)	0.2 (0 to 0.4)	8.4 (1.7 to 14.1)
Chad	Low physical activity	0.1 (0 to 0.1)	1.9 (1 to 3.6)	0 (0 to 0.1)	1.9 (1 to 3.7)	0.1 (0.1 to 0.2)	2.5 (1.3 to 4.8)	0.1 (0 to 0.1)	2.6 (1.3 to 4.9)
Chad	Smoking	0.1 (0 to 0.1)	1.7 (0.7 to 3.1)	0 (0 to 0.1)	1.7 (0.7 to 3.2)	0.1 (0 to 0.1)	1.9 (0.9 to 3.4)	0.1 (0 to 0.1)	2 (0.9 to 3.5)

Location	Risk factor	1990				2019			
		Deaths	YLLs (Years of Life Lost)	YLDs (Years Lived with Disability)	DALYs (Disability-Adjusted Life Years)	Deaths	YLLs (Years of Life Lost)	YLDs (Years Lived with Disability)	DALYs (Disability-Adjusted Life Years)
Chad	Secondhand smoke	0.2 (0.1 to 0.4)	6.8 (1.6 to 12.8)	0.1 (0 to 0.3)	6.9 (1.7 to 13)	0.3 (0.1 to 0.5)	8.3 (2.1 to 15.7)	0.2 (0 to 0.4)	8.5 (2.1 to 16.1)
Chad	Metabolic risks	0.5 (0.1 to 1.2)	11.1 (2.4 to 26.1)	0.3 (0.1 to 0.7)	11.4 (2.4 to 26.7)	1.2 (0.3 to 2.5)	24.5 (5.6 to 55)	0.7 (0.2 to 1.6)	25.2 (5.8 to 56.5)
Chad	High body-mass index	0.1 (0 to 0.3)	1.6 (-0.8 to 5.7)	0.1 (0 to 0.2)	1.7 (-0.8 to 5.9)	0.3 (0 to 0.7)	4.9 (-1.6 to 14)	0.2 (0 to 0.4)	5.1 (-1.6 to 14.4)
Chad	High fasting plasma glucose	0.4 (0.1 to 1.1)	9.6 (1.6 to 24.1)	0.3 (0 to 0.7)	9.8 (1.6 to 24.8)	0.9 (0.2 to 2.1)	20.1 (3.4 to 48.5)	0.5 (0.1 to 1.4)	20.7 (3.5 to 49.8)
Chile	All risk factors	5.6 (4.4 to 6.9)	142.6 (112.9 to 173.6)	6.6 (4.4 to 9.4)	149.2 (118.3 to 181.8)	4.9 (3.6 to 6.4)	114.2 (85.4 to 146.3)	9.3 (5.8 to 14.1)	123.5 (92.7 to 157.8)
Chile	Behavioral risks	4.3 (3.5 to 5)	120.4 (99.6 to 141.3)	5.4 (3.6 to 7.7)	125.8 (104.1 to 148)	3.3 (2.7 to 4)	88.7 (73 to 106.8)	7.1 (4.4 to 10.7)	95.9 (77.9 to 114.9)
Chile	Alcohol use	2 (1.6 to 2.4)	57.8 (47.1 to 69.9)	2.6 (1.7 to 3.7)	60.4 (49 to 73)	1.5 (1.2 to 1.9)	42.4 (33 to 52.8)	3.4 (2.1 to 5.2)	45.8 (35.5 to 57.2)
Chile	Diet high in red meat	0.6 (0.2 to 0.8)	17.2 (6.4 to 22.8)	0.8 (0.3 to 1.2)	17.9 (6.6 to 23.8)	0.7 (0.3 to 0.9)	17.7 (8.5 to 24.5)	1.4 (0.6 to 2.4)	19.1 (9.2 to 26.5)
Chile	Low physical activity	0.2 (0.1 to 0.4)	4.2 (1.7 to 8.7)	0.2 (0.1 to 0.5)	4.4 (1.8 to 9.3)	0.2 (0.1 to 0.4)	4.1 (1.4 to 8.2)	0.3 (0.1 to 0.8)	4.4 (1.5 to 8.9)
Chile	Smoking	1.3 (0.9 to 1.8)	39.6 (24.5 to 55.1)	1.8 (1 to 2.8)	41.4 (25.7 to 57.7)	0.9 (0.6 to 1.3)	25.1 (15.5 to 36)	2 (1 to 3.3)	27.1 (16.5 to 38.9)
Chile	Secondhand smoke	0.5 (0.1 to 0.9)	14.3 (3.5 to 24.6)	0.7 (0.1 to 1.2)	15 (3.7 to 25.8)	0.4 (0.1 to 0.6)	8.9 (2.2 to 15.5)	0.7 (0.2 to 1.4)	9.6 (2.3 to 16.8)
Chile	Metabolic risks	1.7 (0.6 to 3.2)	29.9 (5.6 to 63.4)	1.6 (0.4 to 3.4)	31.5 (6 to 66.5)	2 (0.8 to 3.8)	34.3 (7.2 to 70.2)	2.9 (0.7 to 6.2)	37.2 (8.2 to 75.6)
Chile	High body-mass index	0.7 (0.1 to 1.6)	9.4 (-5.6 to 25.8)	0.6 (-0.1 to 1.5)	10 (-5.6 to 27.2)	0.8 (0.2 to 1.7)	10.1 (-4.5 to 27.1)	0.9 (-0.3 to 2.3)	11 (-4.9 to 29.4)
Chile	High fasting plasma glucose	1.1 (0.2 to 2.4)	21.8 (4.2 to 49.6)	1.1 (0.2 to 2.6)	22.9 (4.4 to 52)	1.3 (0.3 to 2.8)	26.3 (5.2 to 58.1)	2.2 (0.4 to 5.4)	28.5 (5.6 to 62.3)
China	All risk factors	1.8 (1.1 to 2.8)	51.6 (31.1 to 77.7)	2.2 (1.2 to 3.5)	53.8 (32.6 to 81.3)	2.5 (1.5 to 3.7)	64.7 (38.9 to 96.4)	6.3 (3.5 to 10)	71 (43 to 105)
China	Behavioral risks	1 (0.6 to 1.4)	30.2 (18.5 to 41.8)	1.2 (0.7 to 1.9)	31.4 (19.3 to 43.5)	1.1 (0.8 to 1.5)	31.4 (21.2 to 43.2)	3.1 (1.8 to 4.6)	34.4 (23.4 to 46.4)
China	Alcohol use	0.2 (0.1 to 0.3)	5.8 (4.1 to 7.9)	0.2 (0.1 to 0.4)	6 (4.3 to 8.2)	0.2 (0.1 to 0.3)	6.1 (4.1 to 8.3)	0.6 (0.4 to 0.9)	6.7 (4.5 to 9.1)
China	Diet high in red meat	0.3 (0.1 to 0.4)	9 (3.2 to 13.3)	0.4 (0.1 to 0.6)	9.4 (3.3 to 13.7)	0.4 (0.2 to 0.6)	12.2 (5.6 to 17.9)	1.2 (0.5 to 1.9)	13.4 (6.2 to 19.4)
China	Low physical activity	0.1 (0 to 0.1)	1.9 (1 to 3.5)	0.1 (0 to 0.2)	2 (1.1 to 3.7)	0.1 (0 to 0.2)	1.8 (1 to 3.4)	0.2 (0.1 to 0.4)	2 (1.1 to 3.8)
China	Smoking	0.1 (0.1 to 0.1)	1.9 (1.1 to 3)	0.1 (0 to 0.2)	2 (1.2 to 3.1)	0.1 (0.1 to 0.2)	2.5 (1.6 to 3.6)	0.2 (0.1 to 0.4)	2.7 (1.8 to 3.9)
China	Secondhand smoke	0.4 (0.1 to 0.7)	12.8 (3.1 to 22.6)	0.5 (0.1 to 1)	13.3 (3.2 to 23.5)	0.3 (0.1 to 0.6)	10.2 (2.4 to 18.2)	1 (0.2 to 1.8)	11.1 (2.6 to 19.6)
China	Metabolic risks	0.9 (0.3 to 1.9)	24 (7.4 to 48.3)	1.1 (0.3 to 2.2)	25.1 (7.7 to 50.4)	1.6 (0.6 to 2.8)	38.1 (14.2 to 68.2)	3.7 (1.3 to 7)	41.8 (15.7 to 74.8)
China	High body-mass index	0.5 (0.1 to 1.2)	13.2 (2.4 to 31.9)	0.6 (0.1 to 1.5)	13.8 (2.5 to 33.2)	1.1 (0.3 to 2.3)	27.4 (7.6 to 56)	2.6 (0.7 to 5.4)	30 (8.4 to 60.7)
China	High fasting plasma glucose	0.5 (0.1 to 1)	11.6 (2.1 to 27.2)	0.5 (0.1 to 1.3)	12.1 (2.2 to 28.1)	0.5 (0.1 to 1.2)	12.5 (2.3 to 29.8)	1.2 (0.2 to 3)	13.7 (2.5 to 32.4)
Colombia	All risk factors	3.4 (2.3 to 4.7)	86.2 (59.3 to 117.6)	3.6 (2.1 to 5.4)	89.8 (61.3 to 122)	2.5 (1.5 to 4.1)	62.8 (36.2 to 102.7)	4.7 (2.5 to 7.8)	67.4 (38.7 to 110.1)
Colombia	Behavioral risks	2 (1.6 to 2.4)	59.4 (46.6 to 70.7)	2.3 (1.6 to 3.2)	61.7 (48.4 to 73.3)	1.3 (0.9 to 1.8)	38.8 (26.4 to 53.8)	2.7 (1.6 to 4.2)	41.5 (28.3 to 57.4)
Colombia	Alcohol use	0.6 (0.5 to 0.8)	21.4 (16.4 to 26.8)	0.8 (0.5 to 1.2)	22.2 (17 to 27.9)	0.4 (0.3 to 0.6)	13.5 (9 to 19.9)	0.9 (0.5 to 1.4)	14.4 (9.6 to 21.1)
Colombia	Diet high in red meat	0.5 (0.1 to 0.6)	13.6 (4.6 to 17.9)	0.5 (0.2 to 0.8)	14.1 (4.7 to 18.6)	0.4 (0.1 to 0.6)	11.5 (3.8 to 18)	0.8 (0.3 to 1.4)	12.3 (4.2 to 19.1)
Colombia	Low physical activity	0.1 (0.1 to 0.2)	2.8 (1.3 to 5.8)	0.1 (0.1 to 0.3)	2.9 (1.4 to 6.1)	0.1 (0 to 0.2)	2.7 (1.1 to 5.8)	0.2 (0.1 to 0.5)	2.9 (1.2 to 6.3)
Colombia	Smoking	0.6 (0.4 to 0.9)	15.7 (9.8 to 22.7)	0.7 (0.4 to 1.1)	16.4 (10.2 to 23.7)	0.3 (0.2 to 0.4)	6.9 (3.9 to 10.9)	0.5 (0.3 to 0.9)	7.4 (4.2 to 11.6)
Colombia	Secondhand smoke	0.3 (0.1 to 0.5)	9.1 (2.2 to 15.9)	0.4 (0.1 to 0.7)	9.5 (2.3 to 16.5)	0.2 (0 to 0.4)	6 (1.4 to 11.5)	0.4 (0.1 to 0.8)	6.4 (1.5 to 12.2)
Colombia	Metabolic risks	1.6 (0.5 to 3)	31.1 (6.9 to 64.1)	1.4 (0.4 to 3)	32.5 (7.2 to 66.9)	1.4 (0.4 to 2.8)	27 (4 to 61.7)	2.2 (0.4 to 4.9)	29.1 (4.5 to 66.5)
Colombia	High body-mass index	0.5 (0 to 1)	6.3 (-3.9 to 17.6)	0.3 (-0.1 to 0.9)	6.7 (-4 to 18.4)	0.5 (0 to 1.1)	5.1 (-7.3 to 18.3)	0.5 (-0.4 to 1.6)	5.6 (-7.6 to 20)
Colombia	High fasting plasma glucose	1.2 (0.2 to 2.6)	25.9 (5 to 57.9)	1.1 (0.2 to 2.7)	27 (5.2 to 60.3)	1 (0.2 to 2.4)	23.1 (4.3 to 56.9)	1.8 (0.3 to 4.4)	24.9 (4.6 to 60.8)
Comoros	All risk factors	1.4 (0.7 to 2.4)	33.3 (15 to 58.4)	0.8 (0.4 to 1.5)	34.1 (15.4 to 59.7)	2.2 (1.2 to 3.6)	48.8 (24 to 82.2)	1.4 (0.7 to 2.5)	50.2 (24.9 to 84.3)
Comoros	Behavioral risks	0.8 (0.3 to 1.3)	22.4 (8.7 to 38.7)	0.5 (0.2 to 0.9)	22.9 (8.9 to 39.5)	1 (0.6 to 1.6)	29.8 (16.4 to 46.8)	0.8 (0.4 to 1.3)	30.6 (16.8 to 48)
Comoros	Alcohol use	0.1 (0 to 0.2)	2.6 (0.8 to 5.3)	0.1 (0 to 0.1)	2.7 (0.8 to 5.4)	0.2 (0.1 to 0.3)	5.7 (2.2 to 10.3)	0.2 (0.1 to 0.3)	5.8 (2.3 to 10.6)
Comoros	Diet high in red meat	0.2 (0 to 0.3)	4.8 (1 to 8.8)	0.1 (0 to 0.2)	4.9 (1 to 9)	0.2 (0 to 0.3)	5.8 (1.3 to 9.7)	0.2 (0 to 0.3)	5.9 (1.3 to 10)
Comoros	Low physical activity	0.1 (0 to 0.1)	1.5 (0.6 to 3.9)	0 (0 to 0.1)	1.5 (0.7 to 4)	0.1 (0.1 to 0.2)	2 (1.2 to 4.9)	0.1 (0 to 0.1)	2.1 (1.3 to 5)
Comoros	Smoking	0.1 (0 to 0.2)	2.2 (0.9 to 4.2)	0.1 (0 to 0.1)	2.3 (0.9 to 4.3)	0.1 (0.1 to 0.2)	2.8 (1.3 to 4.8)	0.1 (0 to 0.1)	2.9 (1.3 to 4.9)

Location	Risk factor	1990				2019			
		Deaths	YLLs (Years of Life Lost)	YLDs (Years Lived with Disability)	DALYs (Disability-Adjusted Life Years)	Deaths	YLLs (Years of Life Lost)	YLDs (Years Lived with Disability)	DALYs (Disability-Adjusted Life Years)
Comoros	Secondhand smoke	0.4 (0.1 to 0.8)	11.7 (2.2 to 24.2)	0.3 (0 to 0.5)	12 (2.2 to 24.7)	0.5 (0.1 to 0.9)	14.2 (3.1 to 26.7)	0.4 (0.1 to 0.8)	14.6 (3.2 to 27.5)
Comoros	Metabolic risks	0.7 (0.2 to 1.5)	11.4 (0.2 to 29.3)	0.3 (0.1 to 0.8)	11.8 (0.3 to 30)	1.2 (0.3 to 2.5)	19.9 (-0.1 to 47.9)	0.6 (0.1 to 1.5)	20.6 (0.1 to 49.4)
Comoros	High body-mass index	0.2 (0 to 0.6)	1.6 (-5.4 to 9.8)	0.1 (-0.1 to 0.3)	1.7 (-5.5 to 10.1)	0.4 (0 to 1)	3.3 (-9 to 17.5)	0.2 (-0.2 to 0.6)	3.5 (-9.1 to 18)
Comoros	High fasting plasma glucose	0.5 (0.1 to 1.2)	10 (1.6 to 26.3)	0.3 (0 to 0.7)	10.3 (1.7 to 26.8)	0.8 (0.1 to 1.9)	17.1 (3.1 to 42.9)	0.5 (0.1 to 1.3)	17.6 (3.1 to 44.2)
Congo	All risk factors	4.5 (2.7 to 6.9)	118.7 (70.9 to 182.9)	2.7 (1.6 to 4.3)	121.4 (72.8 to 186.6)	6.6 (3.4 to 10.8)	162.4 (83.6 to 267.9)	4.6 (2.4 to 8)	167 (86.5 to 275.9)
Congo	Behavioral risks	3 (2 to 4.3)	87.7 (55.3 to 130.5)	1.9 (1.1 to 3)	89.6 (56.6 to 132.7)	3.9 (2.2 to 6.1)	110.4 (59.9 to 176)	3 (1.6 to 5.1)	113.4 (61.8 to 181.1)
Congo	Alcohol use	1.9 (1.2 to 2.9)	57.9 (35.2 to 88.6)	1.3 (0.7 to 2)	59.2 (35.9 to 90.3)	2.6 (1.3 to 4.2)	73.4 (37.5 to 121.2)	2 (1 to 3.5)	75.4 (38.7 to 124.3)
Congo	Diet high in red meat	0.4 (0.1 to 0.7)	11.9 (2.1 to 20.3)	0.3 (0 to 0.5)	12.1 (2.1 to 20.8)	0.5 (0.1 to 1)	16 (3.1 to 28.6)	0.4 (0.1 to 0.8)	16.4 (3.2 to 29.4)
Congo	Low physical activity	0.3 (0.1 to 0.6)	6.9 (3.1 to 14.1)	0.2 (0.1 to 0.3)	7.1 (3.2 to 14.6)	0.4 (0.2 to 0.8)	8.7 (3.5 to 17.4)	0.3 (0.1 to 0.5)	8.9 (3.7 to 17.8)
Congo	Smoking	0.1 (0.1 to 0.2)	3.1 (1.4 to 5.7)	0.1 (0 to 0.1)	3.2 (1.5 to 5.8)	0.1 (0.1 to 0.2)	3.3 (1.5 to 6.3)	0.1 (0 to 0.2)	3.4 (1.5 to 6.5)
Congo	Secondhand smoke	0.3 (0.1 to 0.7)	11 (2.3 to 21.8)	0.2 (0.1 to 0.5)	11.2 (2.3 to 22.3)	0.4 (0.1 to 0.9)	13.4 (2.6 to 27.5)	0.4 (0.1 to 0.7)	13.7 (2.7 to 28.3)
Congo	Metabolic risks	1.7 (0.5 to 3.7)	35 (5.1 to 80.9)	0.9 (0.2 to 2.1)	35.9 (5.3 to 83)	3 (0.9 to 6.6)	59.6 (10.2 to 141.3)	1.8 (0.4 to 4.2)	61.4 (10.6 to 145.3)
Congo	High body-mass index	0.5 (0 to 1.3)	6.9 (-7.1 to 23.4)	0.2 (-0.1 to 0.6)	7.2 (-7.2 to 24.1)	1.1 (0.1 to 2.5)	14.9 (-9.3 to 45.7)	0.5 (-0.1 to 1.5)	15.4 (-9.4 to 47)
Congo	High fasting plasma glucose	1.2 (0.2 to 3.1)	28.8 (5.3 to 73.5)	0.7 (0.1 to 1.8)	29.5 (5.5 to 75)	2 (0.4 to 5.1)	46.9 (8.4 to 119.2)	1.4 (0.3 to 3.5)	48.2 (8.6 to 122.7)
Cook Islands	All risk factors	15.9 (8.8 to 24.1)	397.9 (230.1 to 594.7)	16.6 (8.8 to 26.7)	414.4 (240.5 to 613.7)	17.8 (10.6 to 26)	418.8 (251.9 to 612.7)	24.6 (13.8 to 38.5)	443.4 (268.3 to 651)
Cook Islands	Behavioral risks	5.2 (3.2 to 7.5)	146.6 (88.4 to 210.5)	6.1 (3.5 to 9.6)	152.7 (92 to 219)	5.6 (3.8 to 7.8)	152.9 (97.9 to 213.5)	9.1 (5.3 to 13.9)	162.1 (103.9 to 226.2)
Cook Islands	Alcohol use	0.2 (0 to 0.6)	8.6 (0 to 21)	0.4 (0 to 0.9)	9 (0 to 21.9)	1.2 (0.6 to 1.9)	39.9 (20.5 to 63)	2.4 (1.1 to 4.1)	42.3 (21.6 to 66.8)
Cook Islands	Diet high in red meat	1.2 (0.4 to 2)	36.9 (11.9 to 59.6)	1.5 (0.5 to 2.6)	38.5 (12.4 to 62.2)	1.2 (0.4 to 1.9)	33.4 (11.7 to 52.8)	2 (0.7 to 3.4)	35.4 (12.4 to 55.8)
Cook Islands	Low physical activity	0.7 (0.3 to 1.3)	15.9 (6.1 to 31.1)	0.7 (0.3 to 1.4)	16.5 (6.3 to 32.6)	0.8 (0.3 to 1.5)	17.3 (6.1 to 32.3)	1 (0.3 to 2.1)	18.3 (6.4 to 34.2)
Cook Islands	Smoking	1.6 (0.9 to 2.5)	42.6 (21.9 to 70.5)	1.8 (0.8 to 3.1)	44.4 (23.1 to 73.6)	1.3 (0.8 to 2)	33.1 (17.8 to 51.9)	2 (1 to 3.4)	35 (19.1 to 54.9)
Cook Islands	Secondhand smoke	1.7 (0.4 to 3.1)	50 (10.8 to 92.4)	2.1 (0.5 to 4)	52.1 (11.2 to 96.2)	1.4 (0.3 to 2.6)	39.6 (9.5 to 72.8)	2.4 (0.6 to 4.5)	41.9 (10.1 to 76.8)
Cook Islands	Metabolic risks	12.3 (5.2 to 20.5)	289.7 (125.1 to 481.1)	12 (4.9 to 21.2)	301.7 (130.6 to 499.7)	14.3 (6.7 to 22.5)	314.9 (149.6 to 509.7)	18.3 (8.4 to 31.3)	333.2 (158.9 to 536.1)
Cook Islands	High body-mass index	9.3 (3 to 17.2)	219.5 (75.1 to 399.2)	9.1 (2.9 to 17.5)	228.5 (77.9 to 414)	10.3 (3.5 to 17.5)	225.4 (80.3 to 377.3)	13 (4.6 to 23.1)	238.4 (85.7 to 400.4)
Cook Islands	High fasting plasma glucose	4.1 (0.8 to 9.2)	93.1 (17.7 to 212.1)	3.9 (0.7 to 9.4)	97.1 (18.6 to 220.3)	5.7 (1.2 to 12.5)	125.3 (26.4 to 282.4)	7.4 (1.6 to 18.1)	132.7 (27.9 to 298.7)
Costa Rica	All risk factors	3.2 (2.2 to 4.5)	82.6 (57.9 to 112.1)	4.8 (2.8 to 7.1)	87.3 (60.6 to 118.6)	3.5 (2.1 to 5.7)	79.9 (47.7 to 126.9)	6.8 (3.7 to 11.8)	86.7 (52.1 to 137.2)
Costa Rica	Behavioral risks	2 (1.5 to 2.4)	60.1 (47.2 to 72.2)	3.3 (2.1 to 4.7)	63.4 (49.9 to 76.2)	1.7 (1.1 to 2.2)	46 (31.6 to 62.9)	3.7 (2.2 to 5.7)	49.7 (34.5 to 68.2)
Costa Rica	Alcohol use	0.7 (0.5 to 0.9)	23.3 (17.8 to 29.1)	1.2 (0.8 to 1.8)	24.5 (18.6 to 30.6)	0.6 (0.4 to 0.8)	18 (11.7 to 25.4)	1.4 (0.8 to 2.2)	19.4 (12.7 to 27.4)
Costa Rica	Diet high in red meat	0.4 (0.1 to 0.6)	13.2 (4.5 to 17.6)	0.7 (0.2 to 1.1)	14 (4.7 to 18.5)	0.4 (0.1 to 0.6)	11.4 (3.2 to 17.7)	0.9 (0.3 to 1.6)	12.3 (3.5 to 19.1)
Costa Rica	Low physical activity	0.1 (0 to 0.2)	2 (1.1 to 4.1)	0.1 (0.1 to 0.2)	2.1 (1.2 to 4.4)	0.1 (0 to 0.2)	2.2 (1.1 to 4.5)	0.2 (0.1 to 0.4)	2.4 (1.2 to 4.9)
Costa Rica	Smoking	0.5 (0.3 to 0.7)	13.8 (8.6 to 20.1)	0.8 (0.4 to 1.3)	14.6 (9 to 21.2)	0.4 (0.2 to 0.6)	8.8 (5.3 to 13.6)	0.7 (0.4 to 1.2)	9.5 (5.7 to 14.8)
Costa Rica	Secondhand smoke	0.4 (0.1 to 0.7)	11.4 (2.7 to 19.8)	0.6 (0.1 to 1.2)	12 (2.8 to 20.8)	0.3 (0.1 to 0.5)	7.8 (1.9 to 14.3)	0.6 (0.1 to 1.2)	8.4 (2 to 15.4)
Costa Rica	Metabolic risks	1.4 (0.5 to 2.8)	25.8 (3.1 to 55.8)	1.7 (0.3 to 3.6)	27.5 (3.4 to 58.7)	2.1 (0.7 to 4.2)	37.7 (7.1 to 84.5)	3.5 (0.9 to 7.6)	41.2 (7.9 to 91.3)
Costa Rica	High body-mass index	0.5 (0 to 1.1)	5.1 (-7 to 18.3)	0.4 (-0.3 to 1.3)	5.5 (-7.3 to 19.5)	0.7 (0.1 to 1.7)	9.1 (-6 to 27.1)	1 (-0.3 to 2.6)	10.1 (-6.3 to 29.5)
Costa Rica	High fasting plasma glucose	1 (0.2 to 2.3)	21.7 (4.4 to 49.2)	1.3 (0.3 to 3.1)	23.1 (4.6 to 52.3)	1.4 (0.3 to 3.4)	30.5 (5.9 to 73.7)	2.7 (0.5 to 6.7)	33.1 (6.4 to 79.3)
Croatia	All risk factors	7.7 (6 to 9.7)	196.8 (155.4 to 241.9)	12.9 (8.4 to 18.2)	209.7 (165.7 to 257.2)	6.5 (4.5 to 9.5)	147.1 (102.2 to 209.3)	14.3 (8.8 to 22.4)	161.4 (112.1 to 229)
Croatia	Behavioral risks	5.7 (4.7 to 6.8)	161.6 (131.9 to 192.5)	10.5 (7.1 to 14.9)	172.2 (140.4 to 205.3)	4.1 (3 to 5.4)	104.6 (76 to 141.2)	10.3 (6.2 to 15.1)	114.9 (83.5 to 154.6)
Croatia	Alcohol use	2.5 (1.9 to 3.2)	70.1 (53.4 to 88.7)	4.6 (2.9 to 6.7)	74.7 (57 to 95.4)	1.7 (1.1 to 2.3)	43.2 (29.3 to 61.3)	4.3 (2.5 to 6.5)	47.5 (32.2 to 67.2)

Location	Risk factor	1990				2019			
		Deaths	YLLs (Years of Life Lost)	YLDs (Years Lived with Disability)	DALYs (Disability-Adjusted Life Years)	Deaths	YLLs (Years of Life Lost)	YLDs (Years Lived with Disability)	DALYs (Disability-Adjusted Life Years)
Croatia	Diet high in red meat	0.6 (0.2 to 0.9)	17.7 (4.7 to 24)	1.2 (0.3 to 1.9)	18.9 (5 to 25.6)	0.7 (0.2 to 1)	17.2 (6.1 to 26.1)	1.7 (0.6 to 2.9)	18.9 (6.7 to 28.8)
Croatia	Low physical activity	0.2 (0.1 to 0.4)	5 (3 to 8.7)	0.3 (0.2 to 0.6)	5.3 (3.2 to 9.3)	0.2 (0.1 to 0.4)	4.3 (2.2 to 7.3)	0.4 (0.2 to 0.8)	4.7 (2.5 to 8)
Croatia	Smoking	2.2 (1.5 to 2.8)	62.8 (44.5 to 82.8)	4.1 (2.4 to 6.3)	66.9 (47.3 to 88.7)	1.4 (0.9 to 1.9)	36.3 (24.3 to 53)	3.6 (2 to 5.7)	39.8 (26.6 to 58.3)
Croatia	Secondhand smoke	0.8 (0.2 to 1.4)	22.2 (5.5 to 38.9)	1.4 (0.3 to 2.7)	23.6 (5.8 to 41.7)	0.5 (0.1 to 1)	13.3 (3.2 to 25)	1.3 (0.3 to 2.5)	14.6 (3.5 to 27)
Croatia	Metabolic risks	2.6 (1 to 4.7)	46.7 (11.1 to 90.6)	3.1 (0.7 to 6.2)	49.8 (12 to 96.2)	3 (1.2 to 5.7)	54.5 (17.8 to 108.2)	5.2 (1.6 to 10.5)	59.7 (19.5 to 117.3)
Croatia	High body-mass index	1.2 (0.3 to 2.5)	18.4 (-3.9 to 44.1)	1.2 (-0.2 to 3)	19.6 (-4.1 to 47.2)	1.5 (0.4 to 2.9)	23.5 (2.3 to 51.1)	2.2 (0.1 to 4.7)	25.7 (2.5 to 55.4)
Croatia	High fasting plasma glucose	1.5 (0.3 to 3.3)	30.2 (5.8 to 69.3)	2 (0.4 to 5)	32.3 (6.1 to 74)	1.8 (0.3 to 4.1)	33.9 (6.4 to 80.8)	3.3 (0.6 to 8.1)	37.2 (7.1 to 88.6)
Cuba	All risk factors	4.7 (3.3 to 6.4)	119 (84.5 to 158)	7 (4.4 to 10.3)	126 (89.3 to 167)	4.7 (3.2 to 6.9)	108.4 (72.4 to 157.6)	9.2 (5.4 to 14.1)	117.6 (78.9 to 170.7)
Cuba	Behavioral risks	3.1 (2.4 to 3.6)	87.7 (70.5 to 104.3)	4.9 (3.3 to 7)	92.6 (74.1 to 110.3)	2.7 (2 to 3.5)	71.9 (52.6 to 94)	5.9 (3.7 to 8.6)	77.8 (57.3 to 101.9)
Cuba	Alcohol use	0.8 (0.6 to 1)	27.2 (20.8 to 34.1)	1.5 (1 to 2.2)	28.7 (21.9 to 36)	0.8 (0.5 to 1)	23.7 (16.5 to 32)	1.9 (1.2 to 2.9)	25.6 (17.8 to 34.6)
Cuba	Diet high in red meat	0.5 (0.1 to 0.7)	15.4 (4.6 to 20.3)	0.9 (0.3 to 1.3)	16.3 (4.9 to 21.5)	0.5 (0.1 to 0.7)	13.7 (4.2 to 20.7)	1.1 (0.3 to 1.9)	14.8 (4.6 to 22.3)
Cuba	Low physical activity	0.3 (0.1 to 0.6)	7.3 (2.6 to 14)	0.4 (0.2 to 0.9)	7.8 (2.7 to 14.8)	0.4 (0.1 to 0.7)	7.7 (2.7 to 14.4)	0.7 (0.2 to 1.4)	8.4 (2.9 to 15.8)
Cuba	Smoking	1.1 (0.8 to 1.5)	29.9 (20.6 to 41)	1.7 (1 to 2.7)	31.7 (21.8 to 43.4)	0.9 (0.6 to 1.3)	23.2 (14.9 to 33.5)	2 (1.1 to 3.1)	25.1 (16.1 to 36.3)
Cuba	Secondhand smoke	0.5 (0.1 to 0.8)	13.7 (3.2 to 24)	0.8 (0.2 to 1.4)	14.5 (3.5 to 25.3)	0.3 (0.1 to 0.5)	8.4 (1.9 to 15.1)	0.7 (0.1 to 1.3)	9.1 (2.1 to 16.3)
Cuba	Metabolic risks	2 (0.6 to 3.8)	37.7 (5.9 to 80.9)	2.4 (0.6 to 5.2)	40.2 (6.5 to 85.6)	2.4 (0.8 to 4.7)	43.8 (8.9 to 93.6)	3.9 (1 to 8.3)	47.7 (9.8 to 101.9)
Cuba	High body-mass index	0.7 (0.1 to 1.5)	8.5 (-8 to 25.6)	0.7 (-0.3 to 1.9)	9.2 (-8.3 to 28)	1 (0.2 to 2)	13.2 (-6.5 to 34.5)	1.3 (-0.4 to 3.3)	14.5 (-6.8 to 37.7)
Cuba	High fasting plasma glucose	1.4 (0.3 to 3.1)	30.7 (5.9 to 69.1)	1.9 (0.3 to 4.4)	32.5 (6.2 to 73.8)	1.6 (0.3 to 3.5)	32.7 (6.4 to 75)	2.8 (0.5 to 7)	35.5 (6.9 to 81.6)
Cyprus	All risk factors	7.4 (5.2 to 10.3)	178.7 (129.6 to 237.3)	13.5 (8.5 to 20.1)	192.3 (139.8 to 255.4)	6.8 (4.7 to 9.4)	151.5 (108.9 to 205.8)	22.1 (13.3 to 34)	173.7 (126.3 to 235.4)
Cyprus	Behavioral risks	4.9 (3.9 to 6.1)	135.1 (105.9 to 170.3)	9.9 (6.3 to 14.2)	145 (113.9 to 182.7)	4.1 (3.2 to 5.1)	105.1 (81.4 to 130.9)	15.3 (9.8 to 22.8)	120.5 (93.3 to 151.2)
Cyprus	Alcohol use	2 (1.5 to 2.5)	55.5 (42 to 72.3)	4 (2.5 to 5.9)	59.6 (45 to 77.1)	1.7 (1.2 to 2.2)	45.2 (32.8 to 58.7)	6.6 (4 to 10)	51.8 (37.2 to 66.9)
Cyprus	Diet high in red meat	1.1 (0.5 to 1.6)	30.5 (14.8 to 43.2)	2.2 (0.9 to 3.6)	32.7 (16 to 46.3)	0.9 (0.3 to 1.2)	21.3 (8.5 to 30.2)	3.1 (1.2 to 5.2)	24.4 (9.8 to 34.9)
Cyprus	Low physical activity	0.3 (0.1 to 0.6)	6.1 (2.6 to 12.7)	0.5 (0.2 to 1.1)	6.6 (2.8 to 13.8)	0.3 (0.1 to 0.6)	5.8 (2.2 to 11.8)	0.8 (0.3 to 1.9)	6.6 (2.5 to 13.6)
Cyprus	Smoking	1.3 (0.9 to 1.8)	37.9 (24.6 to 54.6)	2.8 (1.6 to 4.4)	40.6 (26.5 to 59.1)	1.1 (0.7 to 1.4)	29 (19.9 to 38.9)	4.2 (2.4 to 6.6)	33.3 (22.5 to 45)
Cyprus	Secondhand smoke	0.6 (0.1 to 1.1)	17.4 (4 to 30.5)	1.3 (0.3 to 2.3)	18.6 (4.4 to 32.8)	0.5 (0.1 to 0.8)	12.8 (3.2 to 22.8)	1.9 (0.4 to 3.6)	14.7 (3.7 to 26.1)
Cyprus	Metabolic risks	3.1 (1.1 to 5.9)	53.9 (15.8 to 107.8)	4.4 (1.4 to 9.4)	58.3 (17.4 to 116.8)	3.2 (1.2 to 6.1)	57.4 (19.3 to 113.8)	8.4 (2.6 to 16.9)	65.8 (22.2 to 129.3)
Cyprus	High body-mass index	0.8 (0.2 to 1.8)	14 (0.2 to 33.2)	1.1 (0.1 to 2.6)	15.2 (0.3 to 35.4)	1 (0.3 to 2.1)	17.4 (1.8 to 38.7)	2.5 (0.2 to 5.7)	19.9 (2 to 44.4)
Cyprus	High fasting plasma glucose	2.3 (0.5 to 5.1)	41.7 (8.7 to 93.7)	3.4 (0.7 to 8)	45.1 (9.4 to 101.1)	2.3 (0.5 to 5)	42.4 (8.3 to 92.3)	6.3 (1.2 to 14.8)	48.7 (9.5 to 106.1)
Czechia	All risk factors	8.6 (6.9 to 10.5)	219.8 (177.6 to 264.6)	13.9 (9.2 to 19.3)	233.7 (188.2 to 280.4)	6.3 (4.5 to 8.9)	142.5 (103.2 to 197.8)	14.9 (9.3 to 22.4)	157.4 (114.1 to 218.1)
Czechia	Behavioral risks	6.4 (5.4 to 7.3)	178.1 (151.6 to 202.9)	11.2 (7.5 to 15.5)	189.4 (160.7 to 215.4)	4 (3.1 to 5.1)	100.4 (77.7 to 130.1)	10.6 (6.9 to 15.4)	111 (86.1 to 144.2)
Czechia	Alcohol use	3.3 (2.6 to 4)	94.8 (76.8 to 113.8)	6 (3.9 to 8.5)	100.7 (81.2 to 121)	2.1 (1.6 to 2.8)	55.6 (40.8 to 74.4)	5.9 (3.7 to 8.8)	61.5 (45.1 to 82)
Czechia	Diet high in red meat	1.2 (0.6 to 1.6)	33.1 (16.4 to 44.1)	2.1 (0.9 to 3.3)	35.2 (17.4 to 47)	0.7 (0.3 to 1)	17.9 (8 to 26.4)	1.9 (0.8 to 3.1)	19.8 (8.9 to 29.1)
Czechia	Low physical activity	0.3 (0.1 to 0.4)	5.7 (3.2 to 9.7)	0.4 (0.2 to 0.7)	6.1 (3.4 to 10.4)	0.2 (0.1 to 0.4)	4.3 (2.1 to 7.6)	0.4 (0.2 to 0.9)	4.8 (2.4 to 8.4)
Czechia	Smoking	1.8 (1.3 to 2.3)	48.7 (33.9 to 65.2)	3.1 (1.8 to 4.8)	51.8 (35.8 to 69.6)	1 (0.7 to 1.4)	25.1 (17.5 to 35.6)	2.7 (1.6 to 4.2)	27.8 (19.3 to 39.3)
Czechia	Secondhand smoke	0.5 (0.1 to 0.9)	15.8 (3.7 to 27.7)	1 (0.2 to 1.8)	16.8 (3.8 to 29.4)	0.3 (0.1 to 0.5)	7.6 (1.9 to 13.8)	0.8 (0.2 to 1.6)	8.4 (2.1 to 15.3)
Czechia	Metabolic risks	3 (1.2 to 5.4)	57.1 (16.8 to 108.7)	3.6 (1.1 to 7.4)	60.7 (18.1 to 114.9)	3.1 (1.3 to 5.7)	56.7 (20.5 to 111.5)	5.8 (1.9 to 11.7)	62.5 (22.6 to 122.1)
Czechia	High body-mass index	1.4 (0.3 to 2.6)	22.8 (-0.6 to 49.3)	1.4 (0 to 3.3)	24.2 (-0.7 to 52.9)	1.3 (0.4 to 2.6)	21.4 (3 to 46)	2.1 (0.2 to 4.7)	23.5 (3.2 to 50.9)
Czechia	High fasting plasma glucose	1.7 (0.3 to 3.8)	36.9 (7 to 82.1)	2.4 (0.4 to 5.7)	39.2 (7.5 to 87.1)	2 (0.4 to 4.5)	38.9 (7.7 to 89.3)	4 (0.8 to 9.5)	43 (8.6 to 99.3)
Côte d'Ivoire	All risk factors	2.1 (1.3 to 3.1)	53.9 (33.3 to 78.5)	1.4 (0.8 to 2.2)	55.2 (34.2 to 80.5)	3.3 (2.1 to 5)	79.6 (49.4 to 121.9)	2.3 (1.3 to 3.7)	81.8 (50.6 to 125.2)
Côte d'Ivoire	Behavioral risks	1.4 (1 to 1.9)	41.5 (27.3 to 57.2)	1 (0.6 to 1.4)	42.4 (27.9 to 58.3)	2 (1.3 to 2.8)	55.1 (36 to 79.4)	1.5 (0.9 to 2.3)	56.6 (36.9 to 81.4)

Location	Risk factor	1990				2019			
		Deaths	YLLs (Years of Life Lost)	YLDs (Years Lived with Disability)	DALYs (Disability-Adjusted Life Years)	Deaths	YLLs (Years of Life Lost)	YLDs (Years Lived with Disability)	DALYs (Disability-Adjusted Life Years)
Côte d'Ivoire	Alcohol use	0.7 (0.4 to 1.1)	21.8 (13.3 to 31.9)	0.5 (0.3 to 0.8)	22.3 (13.7 to 32.6)	1.1 (0.7 to 1.6)	31.4 (18.6 to 46.8)	0.8 (0.5 to 1.4)	32.2 (19.2 to 48)
Côte d'Ivoire	Diet high in red meat	0.3 (0.1 to 0.4)	7.7 (1.5 to 11.8)	0.2 (0 to 0.3)	7.9 (1.5 to 12.1)	0.3 (0.1 to 0.5)	8.4 (1.6 to 13.9)	0.2 (0 to 0.4)	8.6 (1.7 to 14.2)
Côte d'Ivoire	Low physical activity	0.1 (0.1 to 0.2)	2.5 (1.4 to 4.5)	0.1 (0 to 0.1)	2.6 (1.4 to 4.7)	0.2 (0.1 to 0.3)	3.3 (1.7 to 6.2)	0.1 (0 to 0.2)	3.4 (1.7 to 6.4)
Côte d'Ivoire	Smoking	0.1 (0.1 to 0.2)	2.8 (1.5 to 4.5)	0.1 (0 to 0.1)	2.8 (1.5 to 4.6)	0.2 (0.1 to 0.3)	3.8 (2 to 6.2)	0.1 (0.1 to 0.2)	3.9 (2.1 to 6.4)
Côte d'Ivoire	Secondhand smoke	0.3 (0.1 to 0.5)	8.3 (1.9 to 15.4)	0.2 (0 to 0.4)	8.5 (2 to 15.8)	0.4 (0.1 to 0.7)	10.6 (2.4 to 20.2)	0.3 (0.1 to 0.5)	10.9 (2.4 to 20.6)
Côte d'Ivoire	Metabolic risks	0.8 (0.2 to 1.6)	14 (1.1 to 33.6)	0.4 (0.1 to 0.9)	14.4 (1.2 to 34.4)	1.5 (0.5 to 3.2)	28 (3.4 to 64.5)	0.9 (0.2 to 2)	28.9 (3.7 to 66.6)
Côte d'Ivoire	High body-mass index	0.3 (0 to 0.7)	2.7 (-5.1 to 11.9)	0.1 (-0.1 to 0.4)	2.9 (-5.2 to 12.2)	0.5 (0 to 1.1)	5 (-7.7 to 19.3)	0.2 (-0.1 to 0.7)	5.2 (-7.8 to 19.8)
Côte d'Ivoire	High fasting plasma glucose	0.5 (0.1 to 1.3)	11.6 (2.1 to 27.5)	0.3 (0.1 to 0.8)	11.9 (2.1 to 28)	1.1 (0.2 to 2.6)	24 (4.4 to 56.2)	0.7 (0.1 to 1.7)	24.7 (4.5 to 57.8)
Democratic People's Republic of Korea	All risk factors	1.8 (0.9 to 2.9)	51.6 (27.5 to 86.5)	2 (1 to 3.5)	53.6 (28.5 to 90)	2.3 (1.2 to 3.7)	65.9 (34.8 to 108.9)	2.8 (1.4 to 4.9)	68.7 (36.1 to 113.4)
Democratic People's Republic of Korea	Behavioral risks	1 (0.5 to 1.5)	31 (16.8 to 49.1)	1.2 (0.6 to 2)	32.2 (17.3 to 50.8)	1.1 (0.6 to 1.7)	35.2 (19.1 to 56.2)	1.5 (0.7 to 2.5)	36.7 (19.9 to 58)
Democratic People's Republic of Korea	Alcohol use	0.2 (0.1 to 0.3)	6.6 (4.1 to 10.4)	0.3 (0.1 to 0.4)	6.9 (4.3 to 10.8)	0.2 (0.1 to 0.3)	6.8 (4 to 10.3)	0.3 (0.2 to 0.5)	7.1 (4.1 to 10.7)
Democratic People's Republic of Korea	Diet high in red meat	0.2 (0 to 0.3)	5.7 (1 to 9.6)	0.2 (0 to 0.4)	5.9 (1.1 to 10)	0.2 (0 to 0.3)	6.2 (1.1 to 10.7)	0.3 (0 to 0.5)	6.5 (1.1 to 11.1)
Democratic People's Republic of Korea	Low physical activity	0.1 (0 to 0.2)	2 (1 to 4.3)	0.1 (0 to 0.2)	2.1 (1.1 to 4.5)	0.1 (0 to 0.2)	2.4 (1.2 to 4.9)	0.1 (0.1 to 0.2)	2.5 (1.3 to 5.1)
Democratic People's Republic of Korea	Smoking	0.1 (0 to 0.1)	2.3 (1.1 to 3.9)	0.1 (0 to 0.2)	2.4 (1.2 to 4.1)	0.1 (0.1 to 0.2)	4 (2.2 to 6.7)	0.2 (0.1 to 0.3)	4.2 (2.3 to 7)
Democratic People's Republic of Korea	Secondhand smoke	0.5 (0.1 to 0.9)	15.4 (3.6 to 29.2)	0.6 (0.1 to 1.1)	16 (3.7 to 30.1)	0.5 (0.1 to 1)	16.9 (3.5 to 32.4)	0.7 (0.2 to 1.4)	17.6 (3.7 to 33.5)
Democratic People's Republic of Korea	Metabolic risks	0.9 (0.2 to 1.9)	22.6 (5.6 to 50.9)	0.9 (0.2 to 2.1)	23.5 (5.8 to 52.9)	1.3 (0.4 to 2.6)	33.6 (9.1 to 69.6)	1.4 (0.4 to 3)	35.1 (9.4 to 72.8)
Democratic People's Republic of Korea	High body-mass index	0.5 (0.1 to 1.3)	12.8 (1.8 to 35.1)	0.5 (0.1 to 1.4)	13.3 (1.8 to 36.4)	0.6 (0.1 to 1.6)	16.4 (2.4 to 43.4)	0.7 (0.1 to 1.9)	17.1 (2.5 to 45.3)
Democratic People's Republic of Korea	High fasting plasma glucose	0.4 (0.1 to 1)	10.3 (1.7 to 26.7)	0.4 (0.1 to 1.1)	10.7 (1.8 to 27.8)	0.7 (0.1 to 1.6)	18.3 (3.4 to 44.1)	0.8 (0.1 to 2)	19.1 (3.6 to 45.8)
Democratic Republic of the Congo	All risk factors	2.5 (1.6 to 3.7)	63.3 (39.9 to 93.6)	1.5 (0.9 to 2.3)	64.8 (40.9 to 95.5)	3.2 (1.7 to 5.5)	78.8 (43.4 to 132.7)	2.1 (1.1 to 3.6)	80.9 (44.6 to 136.2)
Democratic Republic of the Congo	Behavioral risks	1.5 (1.1 to 2)	44.2 (30.6 to 59.7)	1 (0.6 to 1.5)	45.2 (31.4 to 60.9)	1.7 (1 to 2.4)	46.5 (28.8 to 66.4)	1.2 (0.6 to 1.9)	47.6 (29.4 to 68.2)
Democratic Republic of the Congo	Alcohol use	0.9 (0.6 to 1.3)	27.8 (17.8 to 39.6)	0.6 (0.4 to 0.9)	28.4 (18.3 to 40.4)	1 (0.5 to 1.5)	28 (15.7 to 42.1)	0.7 (0.3 to 1.2)	28.7 (16.1 to 43.2)
Democratic Republic of the Congo	Diet high in red meat	0.1 (0 to 0.2)	4.1 (1.2 to 6.4)	0.1 (0 to 0.1)	4.2 (1.2 to 6.5)	0.1 (0.1 to 0.2)	3.9 (1.4 to 6.4)	0.1 (0 to 0.2)	4 (1.5 to 6.6)
Democratic Republic of the Congo	Low physical activity	0.2 (0.1 to 0.4)	4.9 (2.3 to 9.1)	0.1 (0.1 to 0.2)	5 (2.3 to 9.3)	0.3 (0.1 to 0.5)	6.2 (2.9 to 11.7)	0.2 (0.1 to 0.4)	6.4 (3 to 12)
Democratic Republic of the Congo	Smoking	0.1 (0.1 to 0.2)	2.7 (1.3 to 4.5)	0.1 (0 to 0.1)	2.8 (1.3 to 4.6)	0.1 (0 to 0.2)	2.5 (1.1 to 4.3)	0.1 (0 to 0.1)	2.5 (1.2 to 4.4)
Democratic Republic of the Congo	Secondhand smoke	0.2 (0 to 0.4)	6 (1.4 to 11.4)	0.1 (0 to 0.2)	6.1 (1.4 to 11.7)	0.2 (0.1 to 0.4)	7 (1.5 to 13.4)	0.2 (0 to 0.3)	7.1 (1.6 to 13.6)
Democratic Republic of the Congo	Metabolic risks	1.1 (0.3 to 2.2)	20.9 (3.3 to 47.3)	0.6 (0.1 to 1.2)	21.4 (3.4 to 48.2)	1.7 (0.4 to 3.8)	35 (6.7 to 82.4)	1 (0.2 to 2.3)	35.9 (6.9 to 84.8)
Democratic Republic of the Congo	High body-mass index	0.3 (0 to 0.7)	3.6 (-3.8 to 11.8)	0.1 (0 to 0.3)	3.7 (-3.9 to 12.2)	0.4 (0 to 0.9)	5 (-4 to 16.9)	0.2 (-0.1 to 0.5)	5.1 (-4.1 to 17.4)
Democratic Republic of the Congo	High fasting plasma glucose	0.8 (0.2 to 1.9)	17.6 (3.4 to 41.8)	0.4 (0.1 to 1.1)	18.1 (3.5 to 42.9)	1.4 (0.3 to 3.3)	30.6 (5.5 to 75.2)	0.8 (0.1 to 2.1)	31.5 (5.7 to 77)
Denmark	All risk factors	14.9 (12.8 to 17)	395.6 (340.7 to 447.7)	24.9 (17.1 to 34.4)	420.6 (361.9 to 475.2)	8.4 (6.7 to 10.4)	185.7 (150.9 to 226.6)	22.1 (14.1 to 33)	207.8 (167 to 256)
Denmark	Behavioral risks	13.2 (11.4 to 14.9)	362.9 (315.5 to 408.5)	22.9 (15.6 to 32)	385.8 (334.9 to 434.1)	6.5 (5.5 to 7.7)	153.4 (129.2 to 179.6)	18.4 (11.7 to 27.4)	171.8 (143.3 to 202)
Denmark	Alcohol use	6.9 (5.7 to 8.2)	192 (158.1 to 226.4)	12.1 (8.1 to 17.1)	204.1 (168.2 to 241.5)	3.6 (2.8 to 4.4)	85.8 (68 to 104.9)	10.3 (6.4 to 15.4)	96.1 (75.9 to 117.1)
Denmark	Diet high in red meat	2.2 (1.1 to 2.9)	61.1 (30.2 to 82.6)	3.8 (1.8 to 6.1)	64.9 (32 to 87.8)	1.2 (0.6 to 1.6)	28.3 (14.2 to 39.1)	3.4 (1.5 to 5.6)	31.6 (15.7 to 44.3)
Denmark	Low physical activity	0.5 (0.2 to 1)	12 (4.4 to 24.6)	0.8 (0.3 to 1.8)	12.8 (4.7 to 26.1)	0.4 (0.1 to 0.7)	7.2 (2.5 to 14.2)	0.9 (0.3 to 1.9)	8 (2.8 to 15.9)
Denmark	Smoking	4.7 (3.4 to 5.9)	126 (91.7 to 160.5)	7.9 (4.7 to 11.6)	133.9 (97.9 to 170.6)	1.9 (1.4 to 2.4)	42.7 (31.3 to 55.3)	5.1 (3 to 8.1)	47.8 (34.9 to 62.3)
Denmark	Secondhand smoke	0.7 (0.2 to 1.3)	22.6 (5.3 to 40)	1.4 (0.3 to 2.6)	24 (5.6 to 42.7)	0.2 (0.1 to 0.4)	6.9 (1.5 to 12.3)	0.8 (0.2 to 1.6)	7.7 (1.7 to 13.9)
Denmark	Metabolic risks	2.6 (0.9 to 4.8)	50.9 (13.1 to 101.5)	3.2 (0.7 to 6.3)	54.1 (14 to 107.1)	2.6 (1 to 4.8)	46.3 (15.4 to 89.3)	5.4 (1.6 to 11.4)	51.7 (16.9 to 99.1)
Denmark	High body-mass index	1.4 (0.3 to 2.9)	24.7 (-1 to 58.7)	1.4 (-0.1 to 3.4)	26.1 (-1 to 62.2)	1.2 (0.3 to 2.4)	19.3 (1.8 to 42.1)	2.2 (0.1 to 5.2)	21.5 (1.8 to 46.4)
Denmark	High fasting plasma glucose	1.2 (0.2 to 2.9)	27.4 (5 to 64.3)	1.8 (0.3 to 4.4)	29.2 (5.3 to 68.2)	1.5 (0.3 to 3.4)	28.7 (5.5 to 66.7)	3.4 (0.6 to 8.8)	32.1 (6 to 74.4)
Djibouti	All risk factors	1.7 (0.9 to 2.8)	45.3 (24.4 to 71.6)	1.1 (0.6 to 1.9)	46.4 (25 to 73.5)	2.9 (1.6 to 4.7)	68.5 (36 to 111.5)	2.1 (1 to 3.6)	70.6 (36.9 to 115)

Location	Risk factor	1990				2019			
		Deaths	YLLs (Years of Life Lost)	YLDs (Years Lived with Disability)	DALYs (Disability-Adjusted Life Years)	Deaths	YLLs (Years of Life Lost)	YLDs (Years Lived with Disability)	DALYs (Disability-Adjusted Life Years)
Djibouti	Behavioral risks	1.2 (0.7 to 1.8)	34 (19.1 to 51.7)	0.8 (0.4 to 1.3)	34.8 (19.5 to 53.1)	1.6 (0.9 to 2.4)	43.1 (22.8 to 68.1)	1.3 (0.6 to 2.1)	44.4 (23.4 to 69.9)
Djibouti	Alcohol use	0.3 (0.2 to 0.5)	9 (4.7 to 14.5)	0.2 (0.1 to 0.4)	9.2 (4.8 to 14.9)	0.2 (0.1 to 0.3)	4.7 (1.8 to 8.6)	0.1 (0 to 0.3)	4.8 (1.9 to 8.8)
Djibouti	Diet high in red meat	0.3 (0.1 to 0.5)	8.6 (1.7 to 14.4)	0.2 (0 to 0.4)	8.8 (1.8 to 14.7)	0.5 (0.1 to 0.8)	13 (3.2 to 22.2)	0.4 (0.1 to 0.7)	13.4 (3.3 to 22.8)
Djibouti	Low physical activity	0.1 (0 to 0.2)	1.6 (0.8 to 3.7)	0 (0 to 0.1)	1.6 (0.9 to 3.8)	0.1 (0.1 to 0.2)	2.3 (1.3 to 5.4)	0.1 (0 to 0.2)	2.4 (1.3 to 5.6)
Djibouti	Smoking	0.1 (0.1 to 0.2)	2.8 (1.2 to 5)	0.1 (0 to 0.1)	2.9 (1.3 to 5.1)	0.2 (0.1 to 0.4)	4.6 (2.2 to 7.8)	0.1 (0.1 to 0.3)	4.7 (2.2 to 8)
Djibouti	Secondhand smoke	0.4 (0.1 to 0.8)	13.1 (3 to 24.6)	0.3 (0.1 to 0.6)	13.4 (3 to 25.1)	0.7 (0.1 to 1.3)	19.8 (3.7 to 36.3)	0.6 (0.1 to 1.1)	20.4 (3.8 to 37.3)
Djibouti	Metabolic risks	0.6 (0.1 to 1.4)	12.3 (2.1 to 29.8)	0.3 (0.1 to 0.8)	12.6 (2.2 to 30.6)	1.4 (0.5 to 3)	27.6 (5.9 to 61.6)	0.9 (0.2 to 1.9)	28.5 (6.2 to 63.6)
Djibouti	High body-mass index	0.1 (0 to 0.3)	1.3 (-1.6 to 5.8)	0 (0 to 0.2)	1.3 (-1.6 to 6)	0.5 (0 to 1.1)	7.1 (-3.6 to 20.7)	0.3 (-0.1 to 0.7)	7.4 (-3.6 to 21.4)
Djibouti	High fasting plasma glucose	0.5 (0.1 to 1.3)	11.1 (1.8 to 28)	0.3 (0 to 0.8)	11.4 (1.9 to 28.8)	1 (0.2 to 2.4)	21.2 (3.8 to 53)	0.7 (0.1 to 1.7)	21.9 (3.9 to 54.4)
Dominica	All risk factors	6 (3.9 to 9)	148.4 (95.5 to 219)	6.6 (3.8 to 10.2)	155 (99.8 to 229.1)	7.8 (4.5 to 12.6)	175.7 (100.6 to 277.1)	8.6 (4.6 to 14.5)	184.3 (105.9 to 291)
Dominica	Behavioral risks	3.4 (2.6 to 4.3)	101.3 (76 to 129.8)	4.2 (2.7 to 6.1)	105.5 (79.4 to 135.5)	3.7 (2.7 to 4.9)	105.3 (75.1 to 142.1)	4.8 (3 to 7.1)	110.1 (78.1 to 148.9)
Dominica	Alcohol use	1.5 (1.1 to 2)	51.3 (37.1 to 68.1)	2 (1.3 to 3.1)	53.3 (38.6 to 70.7)	1.6 (1.1 to 2.3)	53.3 (36.4 to 76.2)	2.4 (1.4 to 3.7)	55.7 (38.1 to 79.2)
Dominica	Diet high in red meat	0.6 (0.1 to 0.9)	19 (4.5 to 27.3)	0.8 (0.2 to 1.3)	19.8 (4.7 to 28.4)	0.8 (0.2 to 1.2)	22 (5.7 to 33.9)	1 (0.2 to 1.7)	23 (5.9 to 35.6)
Dominica	Low physical activity	0.4 (0.2 to 0.8)	9.7 (3.6 to 19.5)	0.4 (0.2 to 0.9)	10.1 (3.8 to 20.5)	0.6 (0.2 to 1.1)	12.5 (4 to 24.2)	0.6 (0.2 to 1.2)	13.1 (4.2 to 25.5)
Dominica	Smoking	0.5 (0.3 to 0.8)	14 (8.3 to 21.5)	0.6 (0.3 to 1)	14.7 (8.7 to 22.4)	0.4 (0.3 to 0.7)	10.8 (6.4 to 16.3)	0.5 (0.3 to 0.8)	11.3 (6.7 to 17.1)
Dominica	Secondhand smoke	0.4 (0.1 to 0.7)	12.2 (2.9 to 21.8)	0.5 (0.1 to 1)	12.7 (3 to 22.7)	0.4 (0.1 to 0.7)	11.6 (2.7 to 21.5)	0.5 (0.1 to 1)	12.1 (2.8 to 22.2)
Dominica	Metabolic risks	3 (0.9 to 6.1)	53.1 (2.1 to 123.3)	2.7 (0.4 to 5.9)	55.8 (2.8 to 129.1)	4.7 (1.4 to 9.3)	79.3 (3.1 to 185.5)	4.3 (0.6 to 9.8)	83.6 (3.9 to 193.5)
Dominica	High body-mass index	0.9 (-0.1 to 2.1)	5.3 (-20.7 to 30.6)	0.5 (-0.7 to 1.8)	5.8 (-21.3 to 32.2)	1.4 (-0.1 to 3.3)	5.9 (-34.2 to 41.2)	0.7 (-1.1 to 2.5)	6.6 (-35.7 to 43.2)
Dominica	High fasting plasma glucose	2.2 (0.4 to 5)	50.1 (9.8 to 113.4)	2.3 (0.4 to 5.4)	52.4 (10.2 to 118.4)	3.6 (0.8 to 8.1)	78.3 (15.9 to 180.1)	3.8 (0.8 to 9.3)	82.1 (16.8 to 188.7)
Dominican Republic	All risk factors	1.9 (1.3 to 2.5)	48.7 (34.7 to 65.1)	1.7 (1.1 to 2.6)	50.4 (35.7 to 67.4)	3.3 (2.1 to 5.1)	81.9 (50 to 128.3)	4 (2.2 to 6.7)	85.9 (52.3 to 135.1)
Dominican Republic	Behavioral risks	1.4 (1.1 to 1.7)	40.7 (31.5 to 50.8)	1.4 (0.9 to 1.9)	42.1 (32.6 to 52.6)	2.1 (1.4 to 2.9)	62.1 (41.1 to 86.9)	2.9 (1.7 to 4.4)	65 (43.1 to 90.1)
Dominican Republic	Alcohol use	0.5 (0.3 to 0.6)	17.5 (12.8 to 22.8)	0.5 (0.3 to 0.8)	18 (13.2 to 23.5)	0.7 (0.5 to 1.1)	27.3 (17.4 to 39.8)	1.2 (0.7 to 1.9)	28.5 (18.2 to 41.3)
Dominican Republic	Diet high in red meat	0.2 (0 to 0.3)	5.9 (1.2 to 8.5)	0.2 (0 to 0.3)	6.1 (1.2 to 8.7)	0.4 (0.1 to 0.6)	11 (2.4 to 17.7)	0.5 (0.1 to 0.9)	11.5 (2.5 to 18.5)
Dominican Republic	Low physical activity	0.1 (0.1 to 0.3)	2.7 (1.2 to 5.7)	0.1 (0 to 0.2)	2.9 (1.3 to 6)	0.2 (0.1 to 0.4)	4.9 (1.8 to 10.3)	0.2 (0.1 to 0.5)	5.1 (1.9 to 10.7)
Dominican Republic	Smoking	0.5 (0.3 to 0.7)	11.4 (7.3 to 16.3)	0.4 (0.2 to 0.7)	11.9 (7.6 to 16.9)	0.7 (0.5 to 1)	16.2 (10.2 to 24.9)	0.8 (0.5 to 1.3)	17 (10.6 to 25.9)
Dominican Republic	Secondhand smoke	0.2 (0 to 0.3)	5 (1.2 to 9)	0.2 (0 to 0.3)	5.2 (1.2 to 9.3)	0.2 (0 to 0.4)	5.9 (1.2 to 11.7)	0.3 (0.1 to 0.5)	6.2 (1.3 to 12.1)
Dominican Republic	Metabolic risks	0.6 (0.1 to 1.2)	9.1 (-2.2 to 23.6)	0.4 (0 to 1)	9.5 (-2.1 to 24.6)	1.3 (0.4 to 2.9)	22.8 (-3 to 58.8)	1.3 (0 to 3.1)	24.1 (-3 to 61.5)
Dominican Republic	High body-mass index	0.2 (0 to 0.5)	0.5 (-6.9 to 7)	0.1 (-0.1 to 0.4)	0.6 (-7 to 7.3)	0.4 (-0.1 to 1.2)	1.6 (-14.1 to 17.8)	0.2 (-0.5 to 1.1)	1.9 (-14.5 to 18.6)
Dominican Republic	High fasting plasma glucose	0.4 (0.1 to 0.9)	8.8 (1.6 to 21.1)	0.3 (0.1 to 0.8)	9.1 (1.6 to 21.9)	0.9 (0.2 to 2.3)	22.1 (4.1 to 54.9)	1.1 (0.2 to 2.8)	23.2 (4.3 to 57.8)
Ecuador	All risk factors	1.5 (0.9 to 2.3)	34.3 (18.4 to 52.2)	1.1 (0.6 to 1.9)	35.4 (19.2 to 53.7)	2.7 (1.5 to 4.2)	58.4 (30.1 to 96.2)	3.1 (1.5 to 5.3)	61.5 (31.7 to 101.6)
Ecuador	Behavioral risks	0.8 (0.6 to 1)	23.3 (16 to 29.6)	0.7 (0.4 to 1)	24 (16.5 to 30.4)	1.2 (0.8 to 1.6)	33.8 (22.6 to 46.8)	1.7 (1 to 2.6)	35.5 (23.9 to 49.5)
Ecuador	Alcohol use	0.2 (0.1 to 0.2)	5.8 (4 to 7.9)	0.2 (0.1 to 0.2)	5.9 (4.1 to 8.1)	0.3 (0.2 to 0.5)	10.7 (7 to 15.5)	0.5 (0.3 to 0.8)	11.2 (7.4 to 16.3)
Ecuador	Diet high in red meat	0.2 (0.1 to 0.3)	7.1 (1.6 to 9.5)	0.2 (0 to 0.3)	7.3 (1.7 to 9.7)	0.4 (0.1 to 0.7)	12.7 (4.6 to 19.9)	0.6 (0.2 to 1.1)	13.4 (4.8 to 20.9)
Ecuador	Low physical activity	0 (0 to 0.1)	1.2 (0.8 to 3)	0 (0 to 0.1)	1.2 (0.8 to 3.1)	0.1 (0 to 0.1)	1.6 (0.9 to 3.8)	0.1 (0 to 0.2)	1.7 (1 to 4)
Ecuador	Smoking	0.2 (0.1 to 0.3)	5.1 (2.9 to 7.7)	0.2 (0.1 to 0.3)	5.2 (2.9 to 7.9)	0.2 (0.1 to 0.3)	4.9 (2.5 to 7.5)	0.3 (0.1 to 0.4)	5.2 (2.6 to 7.9)
Ecuador	Secondhand smoke	0.2 (0 to 0.3)	4.9 (1.1 to 8.8)	0.1 (0 to 0.3)	5.1 (1.1 to 9.1)	0.2 (0 to 0.3)	5.1 (1.2 to 9.3)	0.2 (0.1 to 0.5)	5.3 (1.2 to 9.8)
Ecuador	Metabolic risks	0.8 (0.2 to 1.6)	11.9 (-3.1 to 30.3)	0.5 (0 to 1.1)	12.4 (-3.1 to 31.3)	1.6 (0.5 to 3.2)	26.7 (-1.1 to 61.3)	1.5 (0 to 3.5)	28.2 (-1 to 64.8)
Ecuador	High body-mass index	0.3 (-0.1 to 0.7)	1.2 (-8.9 to 11.1)	0.1 (-0.2 to 0.5)	1.4 (-9.1 to 11.5)	0.6 (0 to 1.4)	4.7 (-13.5 to 22.1)	0.3 (-0.6 to 1.3)	5 (-14.2 to 23.2)
Ecuador	High fasting plasma glucose	0.5 (0.1 to 1.2)	11.2 (2.1 to 25.5)	0.4 (0.1 to 0.9)	11.6 (2.2 to 26.3)	1.1 (0.2 to 2.5)	23.8 (4.7 to 55.1)	1.3 (0.2 to 3.2)	25.1 (4.9 to 58.2)
Egypt	All risk factors	1.3 (0.8 to 2)	31.6 (14.5 to 49.9)	1.2 (0.5 to 2)	32.8 (15.1 to 51.9)	2.9 (1.4 to 5.3)	67.6 (28.9 to 126.7)	3.2 (1.3 to 5.9)	70.8 (30.5 to 132)

Location	Risk factor	1990				2019			
		Deaths	YLLs (Years of Life Lost)	YLDs (Years Lived with Disability)	DALYs (Disability-Adjusted Life Years)	Deaths	YLLs (Years of Life Lost)	YLDs (Years Lived with Disability)	DALYs (Disability-Adjusted Life Years)
Egypt	Behavioral risks	0.7 (0.4 to 1)	22.9 (13.1 to 33.2)	0.7 (0.4 to 1.1)	23.6 (13.5 to 34.1)	1.2 (0.6 to 1.8)	34.3 (17.1 to 55.3)	1.6 (0.7 to 2.7)	35.8 (17.8 to 57.3)
Egypt	Alcohol use	0 (0 to 0)	0.7 (0.4 to 1.1)	0 (0 to 0)	0.7 (0.4 to 1.2)	0 (0 to 0.1)	1.1 (0.5 to 1.9)	0 (0 to 0.1)	1.1 (0.5 to 1.9)
Egypt	Diet high in red meat	0.2 (0 to 0.2)	5.3 (1 to 7.3)	0.2 (0 to 0.3)	5.4 (1 to 7.6)	0.3 (0.1 to 0.5)	9.2 (2 to 14.9)	0.4 (0.1 to 0.7)	9.6 (2.1 to 15.5)
Egypt	Low physical activity	0.2 (0.1 to 0.3)	4.5 (1.9 to 8.3)	0.2 (0.1 to 0.3)	4.7 (1.9 to 8.6)	0.3 (0.1 to 0.6)	7.5 (2.8 to 14.7)	0.4 (0.1 to 0.7)	7.9 (2.9 to 15.4)
Egypt	Smoking	0 (0 to 0)	0.6 (0.4 to 0.9)	0 (0 to 0)	0.6 (0.4 to 0.9)	0 (0 to 0.1)	1 (0.5 to 1.7)	0 (0 to 0.1)	1.1 (0.6 to 1.8)
Egypt	Secondhand smoke	0.4 (0.1 to 0.6)	12.3 (3.1 to 21.6)	0.4 (0.1 to 0.7)	12.7 (3.1 to 22.1)	0.5 (0.1 to 1)	16.4 (3.5 to 32.5)	0.7 (0.2 to 1.4)	17.2 (3.7 to 33.7)
Egypt	Metabolic risks	0.6 (0.1 to 1.3)	9.5 (-4.8 to 26.9)	0.5 (0 to 1.1)	10 (-4.8 to 27.9)	1.9 (0.6 to 4.1)	36.3 (0.3 to 87.9)	1.8 (0.1 to 4.1)	38.1 (0.4 to 92.8)
Egypt	High body-mass index	0.3 (-0.1 to 0.7)	0.4 (-11.5 to 11.6)	0.1 (-0.2 to 0.6)	0.5 (-11.8 to 12)	0.9 (0 to 2)	9.8 (-14.1 to 37)	0.5 (-0.6 to 1.8)	10.2 (-14.9 to 38.7)
Egypt	High fasting plasma glucose	0.4 (0.1 to 0.9)	9.6 (1.8 to 22.8)	0.4 (0.1 to 0.9)	10 (1.9 to 23.5)	1.2 (0.2 to 3)	29.2 (5.6 to 74.3)	1.4 (0.3 to 3.6)	30.6 (5.9 to 77.6)
El Salvador	All risk factors	1 (0.6 to 1.6)	26.5 (15.4 to 41.5)	1 (0.5 to 1.6)	27.4 (15.9 to 42.9)	2 (1 to 3.5)	47.9 (23.7 to 87.8)	3 (1.4 to 5.5)	50.9 (25.1 to 92.6)
El Salvador	Behavioral risks	0.5 (0.4 to 0.7)	17.5 (13.3 to 22.4)	0.6 (0.4 to 0.8)	18.1 (13.8 to 23.1)	0.8 (0.5 to 1.1)	24.5 (15.7 to 35.2)	1.4 (0.8 to 2.2)	25.9 (16.7 to 37.4)
El Salvador	Alcohol use	0.2 (0.2 to 0.3)	8 (5.7 to 10.8)	0.3 (0.2 to 0.4)	8.2 (5.8 to 11.1)	0.3 (0.2 to 0.5)	11.6 (7.4 to 17.1)	0.6 (0.4 to 1)	12.3 (7.9 to 18.1)
El Salvador	Diet high in red meat	0.1 (0 to 0.1)	2.6 (0.6 to 3.6)	0.1 (0 to 0.1)	2.7 (0.6 to 3.8)	0.1 (0 to 0.2)	4.3 (0.9 to 7.4)	0.2 (0 to 0.5)	4.6 (0.9 to 7.8)
El Salvador	Low physical activity	0 (0 to 0.1)	1.1 (0.6 to 2.4)	0 (0 to 0.1)	1.1 (0.6 to 2.5)	0.1 (0 to 0.1)	1.5 (0.8 to 3.1)	0.1 (0 to 0.2)	1.6 (0.8 to 3.3)
El Salvador	Smoking	0.1 (0.1 to 0.2)	3.5 (2.2 to 5.2)	0.1 (0.1 to 0.2)	3.7 (2.3 to 5.4)	0.1 (0.1 to 0.2)	3.9 (2.3 to 6.2)	0.2 (0.1 to 0.4)	4.1 (2.4 to 6.5)
El Salvador	Secondhand smoke	0.1 (0 to 0.1)	2.8 (0.7 to 5)	0.1 (0 to 0.2)	2.9 (0.7 to 5.2)	0.1 (0 to 0.2)	3.8 (0.8 to 7.6)	0.2 (0 to 0.4)	4 (0.9 to 8)
El Salvador	Metabolic risks	0.5 (0.1 to 1.1)	9.6 (-1 to 23.4)	0.4 (0 to 1)	10 (-1 to 24.2)	1.3 (0.4 to 2.8)	25.2 (1.5 to 59.7)	1.7 (0.2 to 3.9)	26.9 (1.7 to 64)
El Salvador	High body-mass index	0.2 (0 to 0.4)	0.8 (-5.7 to 7)	0.1 (-0.1 to 0.3)	0.9 (-5.7 to 7.2)	0.4 (0 to 1)	3.3 (-9.3 to 15.8)	0.3 (-0.4 to 1.2)	3.6 (-9.6 to 16.9)
El Salvador	High fasting plasma glucose	0.4 (0.1 to 0.9)	9 (1.7 to 21.2)	0.3 (0.1 to 0.8)	9.4 (1.8 to 22)	1 (0.2 to 2.3)	23.2 (4.4 to 57.8)	1.4 (0.3 to 3.7)	24.6 (4.8 to 61.1)
Equatorial Guinea	All risk factors	2.1 (1.1 to 3.4)	56.5 (31.7 to 89.7)	1.2 (0.6 to 2.1)	57.7 (32.3 to 91.5)	6.5 (3.5 to 11.1)	145.8 (77.1 to 252.8)	4.8 (2.5 to 8.6)	150.6 (79.7 to 260.7)
Equatorial Guinea	Behavioral risks	1.4 (0.8 to 2.1)	42.1 (25.3 to 64.3)	0.9 (0.5 to 1.4)	42.9 (26 to 65.8)	3.8 (2.2 to 6.2)	99.6 (56 to 166.1)	3.2 (1.6 to 5.7)	102.8 (58 to 172.4)
Equatorial Guinea	Alcohol use	0.7 (0.4 to 1.1)	22.2 (12.4 to 34.3)	0.5 (0.2 to 0.8)	22.7 (12.6 to 34.8)	2.3 (1.3 to 3.9)	62.8 (33.2 to 107.2)	2 (1 to 3.6)	64.8 (34.4 to 110)
Equatorial Guinea	Diet high in red meat	0.3 (0.1 to 0.4)	7.8 (1.5 to 13.9)	0.2 (0 to 0.3)	7.9 (1.6 to 14.2)	0.7 (0.2 to 1.3)	17.9 (4.6 to 34.4)	0.6 (0.1 to 1.1)	18.5 (4.8 to 35.6)
Equatorial Guinea	Low physical activity	0.2 (0.1 to 0.3)	3.8 (1.6 to 7.3)	0.1 (0 to 0.2)	3.9 (1.6 to 7.5)	0.4 (0.2 to 0.9)	8.6 (3.1 to 18.2)	0.3 (0.1 to 0.6)	8.9 (3.2 to 18.8)
Equatorial Guinea	Smoking	0.1 (0 to 0.1)	1.6 (0.6 to 3.1)	0 (0 to 0.1)	1.7 (0.7 to 3.1)	0.1 (0.1 to 0.2)	2.8 (1.1 to 5.5)	0.1 (0 to 0.2)	2.9 (1.1 to 5.8)
Equatorial Guinea	Secondhand smoke	0.3 (0.1 to 0.5)	8.2 (1.9 to 15.7)	0.2 (0 to 0.3)	8.3 (2 to 16)	0.4 (0.1 to 0.9)	12.3 (2.5 to 25.5)	0.4 (0.1 to 0.8)	12.6 (2.6 to 26.3)
Equatorial Guinea	Metabolic risks	0.7 (0.2 to 1.7)	16 (2.4 to 39.7)	0.4 (0.1 to 1)	16.4 (2.4 to 40.8)	3.1 (1 to 6.8)	53.7 (10.5 to 127.2)	1.9 (0.4 to 4.5)	55.6 (11 to 131.4)
Equatorial Guinea	High body-mass index	0.1 (0 to 0.4)	1.4 (-2.6 to 6.1)	0.1 (0 to 0.2)	1.4 (-2.6 to 6.2)	1.3 (0.2 to 3)	16.2 (-10.1 to 48.7)	0.6 (-0.2 to 1.7)	16.9 (-10.4 to 50.3)
Equatorial Guinea	High fasting plasma glucose	0.6 (0.1 to 1.6)	14.8 (2.4 to 37.8)	0.3 (0.1 to 0.9)	15.2 (2.5 to 38.7)	1.9 (0.4 to 5)	39.9 (7.1 to 104.4)	1.3 (0.2 to 3.7)	41.2 (7.3 to 107.5)
Eritrea	All risk factors	1.3 (0.6 to 2.4)	35.7 (16.5 to 63.5)	0.8 (0.3 to 1.5)	36.5 (16.9 to 64.9)	2.7 (1.5 to 4.6)	67.6 (37.4 to 114.4)	1.7 (0.9 to 3.1)	69.4 (38.3 to 117.3)
Eritrea	Behavioral risks	0.7 (0.4 to 1.1)	22.6 (11.5 to 36.3)	0.5 (0.2 to 0.8)	23.1 (11.8 to 36.9)	1.2 (0.7 to 1.7)	34.6 (20.8 to 52)	0.8 (0.5 to 1.3)	35.4 (21.3 to 53.1)
Eritrea	Alcohol use	0.3 (0.1 to 0.5)	10 (4.6 to 17)	0.2 (0.1 to 0.4)	10.2 (4.7 to 17.4)	0.5 (0.3 to 0.8)	15.4 (8.2 to 25.2)	0.4 (0.2 to 0.7)	15.8 (8.4 to 26)
Eritrea	Diet high in red meat	0.2 (0 to 0.3)	5.5 (1 to 10.4)	0.1 (0 to 0.2)	5.7 (1 to 10.6)	0.3 (0.1 to 0.5)	8.8 (1.7 to 15.2)	0.2 (0 to 0.4)	9 (1.8 to 15.5)
Eritrea	Low physical activity	0.1 (0 to 0.1)	1.7 (0.9 to 3.9)	0 (0 to 0.1)	1.8 (0.9 to 4)	0.1 (0.1 to 0.2)	2.7 (1.6 to 5.8)	0.1 (0 to 0.2)	2.8 (1.6 to 5.9)
Eritrea	Smoking	0 (0 to 0)	0.5 (0.2 to 0.9)	0 (0 to 0)	0.5 (0.2 to 1)	0 (0 to 0)	0.6 (0.2 to 1.1)	0 (0 to 0)	0.6 (0.2 to 1.1)
Eritrea	Secondhand smoke	0.2 (0 to 0.3)	5.3 (1 to 10.9)	0.1 (0 to 0.2)	5.4 (1 to 11.1)	0.2 (0.1 to 0.5)	7.7 (1.7 to 14.9)	0.2 (0 to 0.4)	7.9 (1.7 to 15.3)
Eritrea	Metabolic risks	0.6 (0.1 to 1.5)	13.8 (2.5 to 34.7)	0.3 (0.1 to 0.8)	14.1 (2.5 to 35.5)	1.6 (0.5 to 3.3)	34.9 (10.1 to 74.5)	1 (0.3 to 2.1)	35.8 (10.4 to 76.6)
Eritrea	High body-mass index	0.1 (0 to 0.3)	2.1 (-0.7 to 6.9)	0.1 (0 to 0.2)	2.2 (-0.7 to 7.1)	0.6 (0.1 to 1.2)	10.7 (-0.2 to 25.7)	0.3 (0 to 0.7)	11 (-0.1 to 26.2)
Eritrea	High fasting plasma glucose	0.5 (0.1 to 1.3)	11.8 (1.8 to 32.1)	0.3 (0 to 0.7)	12.1 (1.8 to 32.9)	1.1 (0.2 to 2.6)	25 (4.4 to 60.7)	0.7 (0.1 to 1.7)	25.6 (4.5 to 62.1)
Estonia	All risk factors	5.6 (4.3 to 7)	159.4 (122 to 194.1)	9 (5.8 to 12.5)	168.4 (129.1 to 204.6)	5.5 (3.9 to 7.6)	140.7 (99.2 to 193.3)	14.1 (8.8 to 21.5)	154.7 (109.5 to 211.4)

Location	Risk factor	1990				2019			
		Deaths	YLLs (Years of Life Lost)	YLDs (Years Lived with Disability)	DALYs (Disability-Adjusted Life Years)	Deaths	YLLs (Years of Life Lost)	YLDs (Years Lived with Disability)	DALYs (Disability-Adjusted Life Years)
Estonia	Behavioral risks	4.3 (3.5 to 5.1)	134.7 (110.5 to 159.2)	7.4 (4.9 to 10.3)	142.1 (115.8 to 167.8)	3.8 (2.8 to 5.1)	109 (78.8 to 145.8)	10.8 (6.7 to 16.3)	119.9 (87.3 to 160.9)
Estonia	Alcohol use	2 (1.5 to 2.5)	65 (50.3 to 80.8)	3.5 (2.3 to 5.1)	68.5 (52.3 to 85.2)	2.1 (1.4 to 2.8)	61.1 (41.7 to 84.2)	6.1 (3.6 to 9.2)	67.2 (46 to 92.3)
Estonia	Diet high in red meat	0.8 (0.3 to 1.1)	26 (10.3 to 34.6)	1.4 (0.6 to 2.2)	27.5 (10.9 to 36.5)	0.6 (0.2 to 1)	17.9 (6.5 to 27.6)	1.8 (0.6 to 3.1)	19.7 (7.1 to 30.5)
Estonia	Low physical activity	0.2 (0.1 to 0.3)	3.7 (2.3 to 6.9)	0.2 (0.1 to 0.4)	3.9 (2.5 to 7.3)	0.2 (0.1 to 0.3)	3.3 (1.9 to 6.1)	0.3 (0.2 to 0.7)	3.6 (2.1 to 6.7)
Estonia	Smoking	1.1 (0.7 to 1.6)	34.7 (22 to 48.8)	1.9 (1 to 3)	36.6 (23.3 to 51.3)	1 (0.6 to 1.4)	27.3 (16.6 to 41.1)	2.7 (1.4 to 4.4)	30 (18.3 to 45)
Estonia	Secondhand smoke	0.5 (0.1 to 0.9)	16.8 (4 to 28.8)	0.9 (0.2 to 1.7)	17.7 (4.2 to 30.4)	0.3 (0.1 to 0.6)	9.3 (2.1 to 17)	0.9 (0.2 to 1.8)	10.2 (2.3 to 18.7)
Estonia	Metabolic risks	1.6 (0.5 to 3.1)	30.6 (1.1 to 65.5)	1.9 (0.2 to 4.1)	32.5 (1.6 to 69.3)	2.1 (0.8 to 4)	40.9 (11.5 to 82.3)	4.1 (1.1 to 8.4)	45 (12.9 to 91.5)
Estonia	High body-mass index	0.9 (0.1 to 2)	12.6 (-10.4 to 37.5)	0.8 (-0.5 to 2.3)	13.4 (-10.7 to 40)	1.3 (0.3 to 2.5)	21.5 (-1 to 48.1)	2.1 (-0.1 to 5.1)	23.6 (-1 to 52.9)
Estonia	High fasting plasma glucose	0.8 (0.1 to 1.8)	19 (3.3 to 44.4)	1.1 (0.2 to 2.8)	20.1 (3.5 to 46.6)	0.9 (0.2 to 2.2)	21.2 (3.7 to 50.5)	2.2 (0.4 to 5.6)	23.3 (4.1 to 56)
Eswatini	All risk factors	3.6 (2.1 to 5.5)	80.8 (47.1 to 124.4)	2.3 (1.3 to 3.6)	83.1 (48.6 to 127.2)	5.6 (3 to 9.3)	117.5 (59.2 to 197.9)	3.7 (1.8 to 6.3)	121.2 (61.5 to 203.6)
Eswatini	Behavioral risks	2 (1.3 to 2.7)	52.3 (33.8 to 73.2)	1.4 (0.8 to 2.1)	53.7 (34.7 to 75.3)	2.5 (1.5 to 3.8)	62.7 (35.5 to 100.7)	1.8 (1 to 3)	64.6 (36.4 to 103.5)
Eswatini	Alcohol use	0.8 (0.5 to 1.2)	21.6 (13.9 to 31.4)	0.6 (0.3 to 0.9)	22.2 (14.3 to 32.1)	1 (0.6 to 1.7)	27.2 (14.9 to 45.9)	0.8 (0.4 to 1.3)	28 (15.4 to 47.2)
Eswatini	Diet high in red meat	0.6 (0.2 to 0.9)	15.8 (5 to 24.6)	0.4 (0.1 to 0.7)	16.2 (5.1 to 25.2)	0.7 (0.2 to 1.1)	18.1 (5.4 to 32.4)	0.5 (0.1 to 1)	18.6 (5.6 to 33.2)
Eswatini	Low physical activity	0.1 (0.1 to 0.3)	3.2 (1.6 to 6)	0.1 (0 to 0.2)	3.3 (1.7 to 6.2)	0.2 (0.1 to 0.5)	5 (2.2 to 10.2)	0.2 (0.1 to 0.3)	5.1 (2.3 to 10.5)
Eswatini	Smoking	0.3 (0.2 to 0.4)	4.5 (2.5 to 7.1)	0.1 (0.1 to 0.2)	4.7 (2.6 to 7.3)	0.3 (0.2 to 0.5)	4.5 (2.3 to 7.5)	0.1 (0.1 to 0.3)	4.6 (2.4 to 7.7)
Eswatini	Secondhand smoke	0.3 (0.1 to 0.6)	9.4 (2 to 18.1)	0.2 (0.1 to 0.5)	9.7 (2.1 to 18.5)	0.4 (0.1 to 0.7)	10.4 (2.2 to 20.8)	0.3 (0.1 to 0.6)	10.7 (2.2 to 21.4)
Eswatini	Metabolic risks	1.9 (0.6 to 3.5)	32.9 (4.5 to 68)	1 (0.2 to 2.2)	33.9 (4.7 to 70.4)	3.5 (1.3 to 6.9)	62 (14.9 to 133.5)	2.1 (0.5 to 4.4)	64 (15.5 to 137.7)
Eswatini	High body-mass index	0.9 (0.2 to 1.9)	12.7 (-6 to 32.9)	0.5 (0 to 1.1)	13.2 (-5.9 to 34)	1.6 (0.4 to 3.3)	21.3 (-9.3 to 54.8)	0.8 (-0.1 to 1.9)	22 (-9.3 to 56.7)
Eswatini	High fasting plasma glucose	1 (0.2 to 2.4)	21.7 (3.8 to 50.9)	0.6 (0.1 to 1.6)	22.4 (3.9 to 52.2)	2.1 (0.4 to 5.2)	44.8 (8.2 to 113.2)	1.4 (0.2 to 3.6)	46.2 (8.5 to 115.8)
Ethiopia	All risk factors	1.9 (1 to 3.2)	52.8 (29.6 to 84)	1.1 (0.6 to 2)	53.9 (30.4 to 85.7)	2.1 (1.2 to 3.3)	50.4 (29.9 to 77)	1.5 (0.8 to 2.3)	51.9 (30.9 to 78.8)
Ethiopia	Behavioral risks	1.2 (0.7 to 1.7)	37 (22.2 to 54.5)	0.8 (0.4 to 1.2)	37.8 (22.6 to 55.8)	1.2 (0.8 to 1.7)	34.1 (22.7 to 48.4)	1 (0.6 to 1.5)	35.1 (23.4 to 49.6)
Ethiopia	Alcohol use	0.7 (0.4 to 1.1)	22.6 (12.5 to 35.2)	0.5 (0.2 to 0.8)	23.1 (12.7 to 35.9)	0.8 (0.5 to 1.1)	22.1 (13.4 to 33.3)	0.6 (0.3 to 1)	22.7 (13.8 to 34.2)
Ethiopia	Diet high in red meat	0.2 (0 to 0.3)	6.5 (1.3 to 10.2)	0.1 (0 to 0.2)	6.6 (1.3 to 10.4)	0.2 (0 to 0.3)	6 (1.2 to 9.4)	0.2 (0 to 0.3)	6.2 (1.3 to 9.6)
Ethiopia	Low physical activity	0.1 (0 to 0.2)	2.2 (1.3 to 4.9)	0 (0 to 0.1)	2.2 (1.3 to 5)	0.1 (0.1 to 0.2)	1.9 (1.2 to 4.2)	0.1 (0 to 0.1)	1.9 (1.2 to 4.3)
Ethiopia	Smoking	0 (0 to 0.1)	0.9 (0.4 to 1.7)	0 (0 to 0)	0.9 (0.4 to 1.8)	0 (0 to 0)	0.7 (0.3 to 1.2)	0 (0 to 0)	0.7 (0.3 to 1.3)
Ethiopia	Secondhand smoke	0.2 (0 to 0.3)	5.7 (1.2 to 11.1)	0.1 (0 to 0.2)	5.8 (1.2 to 11.3)	0.1 (0 to 0.2)	4.2 (1 to 7.7)	0.1 (0 to 0.2)	4.3 (1 to 7.8)
Ethiopia	Metabolic risks	0.8 (0.2 to 1.8)	16.8 (1.5 to 41.8)	0.4 (0.1 to 1)	17.2 (1.5 to 42.6)	1 (0.3 to 2.1)	17.4 (2.5 to 40.6)	0.6 (0.1 to 1.2)	18 (2.6 to 42.2)
Ethiopia	High body-mass index	0.1 (0 to 0.3)	0.2 (-4.2 to 5)	0 (0 to 0.1)	0.2 (-4.2 to 5.1)	0.3 (0 to 0.7)	2.7 (-4.2 to 11)	0.1 (-0.1 to 0.4)	2.8 (-4.3 to 11.4)
Ethiopia	High fasting plasma glucose	0.7 (0.1 to 1.7)	16.7 (2.8 to 41.4)	0.4 (0.1 to 1)	17.1 (2.9 to 42.4)	0.7 (0.1 to 1.7)	15 (2.7 to 36.6)	0.5 (0.1 to 1.1)	15.5 (2.7 to 37.7)
Fiji	All risk factors	11.5 (6.5 to 17)	306 (178.5 to 452.3)	9.1 (5 to 14.3)	315.2 (183.7 to 464.8)	16.4 (8.9 to 24.9)	410.2 (224.2 to 626.3)	13.9 (7.3 to 22.1)	424.1 (231.6 to 643.7)
Fiji	Behavioral risks	3.9 (2.5 to 5.4)	114.3 (73.5 to 163)	3.3 (1.9 to 5.2)	117.7 (75.6 to 167.5)	4.1 (2.6 to 5.9)	115 (72.4 to 166.5)	3.8 (2.2 to 6)	118.8 (74.8 to 171.8)
Fiji	Alcohol use	0.2 (0.1 to 0.3)	6.6 (4.3 to 9.3)	0.2 (0.1 to 0.3)	6.8 (4.4 to 9.5)	0.3 (0.2 to 0.4)	10 (6.2 to 15.4)	0.3 (0.2 to 0.5)	10.3 (6.4 to 15.8)
Fiji	Diet high in red meat	1.2 (0.5 to 1.7)	36.1 (14 to 54.3)	1 (0.4 to 1.7)	37.1 (14.3 to 55.7)	1.3 (0.5 to 1.9)	37.1 (13.4 to 58.5)	1.2 (0.4 to 2.1)	38.3 (13.9 to 60.2)
Fiji	Low physical activity	0.5 (0.2 to 0.9)	11 (4 to 22)	0.3 (0.1 to 0.7)	11.3 (4.1 to 22.7)	0.7 (0.3 to 1.2)	15.4 (5.5 to 30.1)	0.5 (0.2 to 1.1)	15.9 (5.8 to 31.6)
Fiji	Smoking	1.2 (0.6 to 1.8)	32.7 (16.3 to 52.5)	1 (0.5 to 1.6)	33.7 (16.8 to 53.9)	0.8 (0.4 to 1.3)	21.5 (11.1 to 34.8)	0.7 (0.3 to 1.3)	22.2 (11.4 to 35.9)
Fiji	Secondhand smoke	1.1 (0.3 to 2)	33.9 (7.8 to 61.3)	1 (0.2 to 1.9)	34.9 (8 to 62.9)	1.2 (0.3 to 2.3)	36.2 (8.5 to 68.3)	1.2 (0.3 to 2.3)	37.4 (8.8 to 70)
Fiji	Metabolic risks	8.8 (3.8 to 14.5)	221.6 (95.9 to 372.4)	6.7 (2.8 to 11.6)	228.3 (99.1 to 383)	13.8 (6.2 to 22.8)	333 (150.3 to 550.3)	11.3 (5.1 to 19.1)	344.3 (155.4 to 568.5)
Fiji	High body-mass index	6.3 (2 to 11.4)	157.9 (50.1 to 287.5)	4.8 (1.5 to 8.8)	162.7 (51.7 to 295.8)	9.4 (3 to 16.8)	220.3 (72.9 to 396.5)	7.5 (2.6 to 13.8)	227.8 (75.1 to 408.8)
Fiji	High fasting plasma glucose	3.3 (0.7 to 7.4)	83 (16.9 to 189.7)	2.5 (0.5 to 6)	85.6 (17.3 to 195.1)	6.3 (1.4 to 13.9)	154.7 (33.6 to 348.4)	5.3 (1.1 to 12.3)	160 (34.7 to 359)

Location	Risk factor	1990				2019			
		Deaths	YLLs (Years of Life Lost)	YLDs (Years Lived with Disability)	DALYs (Disability-Adjusted Life Years)	Deaths	YLLs (Years of Life Lost)	YLDs (Years Lived with Disability)	DALYs (Disability-Adjusted Life Years)
Finland	All risk factors	6.6 (5.2 to 8.2)	180.4 (144.7 to 217.7)	16.3 (10.5 to 22.9)	196.7 (157.2 to 237.6)	5.4 (4 to 7.2)	128 (96.9 to 164.9)	21 (13 to 32.6)	149 (112.7 to 194.3)
Finland	Behavioral risks	5.1 (4.3 to 5.8)	151.1 (129.5 to 173.6)	13.3 (8.9 to 18.7)	164.4 (140.2 to 189.9)	3.6 (2.9 to 4.2)	94.6 (78.7 to 111.4)	15.3 (9.5 to 22.7)	109.9 (90.5 to 131.1)
Finland	Alcohol use	2.5 (2 to 3)	75.9 (61.4 to 90.7)	6.6 (4.3 to 9.7)	82.5 (66.2 to 98.5)	1.8 (1.4 to 2.3)	50.6 (39.1 to 63.2)	8.1 (5 to 12.4)	58.7 (44.9 to 74.4)
Finland	Diet high in red meat	1.1 (0.6 to 1.5)	31.4 (15.7 to 42.4)	2.8 (1.3 to 4.4)	34.2 (17 to 46.4)	0.8 (0.4 to 1.1)	19.9 (9.8 to 27.5)	3.2 (1.4 to 5.4)	23.2 (11.3 to 32.3)
Finland	Low physical activity	0.3 (0.1 to 0.5)	6.1 (2.3 to 12.5)	0.6 (0.2 to 1.3)	6.6 (2.5 to 13.7)	0.2 (0.1 to 0.5)	5 (1.7 to 9.9)	0.8 (0.3 to 1.8)	5.8 (2 to 11.5)
Finland	Smoking	1.4 (1 to 1.9)	43.2 (30.3 to 58.3)	3.8 (2.2 to 5.8)	47 (32.8 to 63.8)	0.9 (0.6 to 1.1)	22.7 (16.1 to 29.9)	3.7 (2 to 6)	26.4 (18.5 to 35.2)
Finland	Secondhand smoke	0.3 (0.1 to 0.6)	10.5 (2.5 to 18.5)	0.9 (0.2 to 1.7)	11.4 (2.6 to 20.2)	0.2 (0 to 0.3)	5.1 (1.2 to 9)	0.8 (0.2 to 1.6)	5.9 (1.4 to 10.4)
Finland	Metabolic risks	2 (0.7 to 3.9)	38.5 (8.5 to 79.5)	3.9 (1 to 8)	42.4 (9.7 to 86.8)	2.3 (0.9 to 4.4)	43.3 (13.4 to 86.5)	7.4 (2.2 to 15.3)	50.7 (15.6 to 101.6)
Finland	High body-mass index	0.8 (0.1 to 1.7)	11.5 (-4.5 to 31)	1.2 (-0.3 to 3.1)	12.7 (-4.6 to 33.5)	0.9 (0.2 to 1.7)	13.6 (-0.1 to 31.3)	2.4 (0.2 to 5.8)	16.1 (0.1 to 36.1)
Finland	High fasting plasma glucose	1.3 (0.3 to 2.9)	28.4 (5.3 to 64.3)	2.8 (0.5 to 6.7)	31.1 (5.8 to 70.6)	1.5 (0.3 to 3.5)	31.6 (6.2 to 70.8)	5.3 (1 to 13.2)	36.9 (7.4 to 83.9)
France	All risk factors	9 (7.5 to 10.5)	237.6 (200.4 to 273)	18.3 (12.2 to 25.5)	255.9 (217 to 294.7)	6.7 (5.4 to 8.2)	162.7 (131.3 to 195)	21.9 (13.6 to 33.3)	184.6 (148.6 to 223.3)
France	Behavioral risks	7.8 (6.6 to 8.9)	215.8 (185 to 246.8)	16.5 (11.1 to 23)	232.2 (198.4 to 265.9)	5.4 (4.5 to 6.3)	140.8 (118.9 to 165.5)	18.8 (11.5 to 28.5)	159.6 (132.4 to 189.6)
France	Alcohol use	4.4 (3.6 to 5.3)	123.1 (100.7 to 147.5)	9.4 (6.1 to 13.4)	132.4 (108.3 to 158.9)	2.9 (2.3 to 3.6)	77.5 (62.1 to 95.2)	10.4 (6.2 to 16.2)	87.9 (69.6 to 108.9)
France	Diet high in red meat	1.7 (0.8 to 2.3)	45.4 (22.3 to 64)	3.5 (1.6 to 5.7)	48.9 (23.9 to 69)	1.1 (0.6 to 1.6)	29.3 (14.4 to 41.4)	3.9 (1.7 to 6.6)	33.2 (16.1 to 46.7)
France	Low physical activity	0.5 (0.2 to 1)	12.2 (3.8 to 23.2)	1 (0.3 to 2)	13.2 (4.1 to 25)	0.4 (0.1 to 0.8)	9.3 (3 to 16.9)	1.3 (0.4 to 2.6)	10.5 (3.3 to 19.4)
France	Smoking	1.5 (1.1 to 2)	43.2 (29.5 to 57.9)	3.3 (1.9 to 5)	46.5 (32.1 to 62.4)	1.2 (0.8 to 1.6)	33 (22.1 to 44.6)	4.4 (2.4 to 7.2)	37.4 (25 to 50.3)
France	Secondhand smoke	0.5 (0.1 to 0.9)	17.2 (4.3 to 29.6)	1.3 (0.3 to 2.3)	18.5 (4.5 to 32)	0.2 (0.1 to 0.4)	7.6 (1.9 to 13.2)	1 (0.2 to 1.9)	8.6 (2.1 to 15.1)
France	Metabolic risks	1.7 (0.6 to 3.1)	30.4 (7.1 to 59.4)	2.6 (0.6 to 5.3)	33 (7.7 to 64.5)	1.8 (0.7 to 3.3)	30.6 (7.3 to 61.2)	4.3 (1 to 8.9)	34.9 (8.3 to 69.3)
France	High body-mass index	1 (0.2 to 2)	15.7 (-2 to 36.4)	1.4 (-0.1 to 3.2)	17.1 (-2 to 39.1)	1 (0.2 to 2)	13.8 (-3 to 33.6)	1.9 (-0.2 to 5)	15.7 (-3.1 to 38.1)
France	High fasting plasma glucose	0.8 (0.1 to 1.8)	15.4 (2.8 to 35.7)	1.3 (0.2 to 3.2)	16.7 (3.1 to 39.1)	0.9 (0.2 to 2.1)	17.9 (3.4 to 42.1)	2.5 (0.4 to 6.5)	20.4 (3.9 to 48)
Gabon	All risk factors	5.5 (3.7 to 7.7)	143.3 (96.8 to 196.8)	3.6 (2.2 to 5.4)	146.9 (99.7 to 201.5)	7.4 (4.2 to 11.6)	170.8 (98.9 to 266.6)	5.6 (3.1 to 9.3)	176.4 (102.2 to 274.1)
Gabon	Behavioral risks	4.2 (2.9 to 5.7)	119 (81.5 to 162.6)	2.9 (1.8 to 4.2)	121.9 (83.6 to 166.3)	4.6 (2.8 to 6.9)	122.4 (73.7 to 182)	3.9 (2.2 to 6.1)	126.2 (76.2 to 187.3)
Gabon	Alcohol use	2.5 (1.7 to 3.5)	72 (47.9 to 100)	1.8 (1.1 to 2.6)	73.8 (48.8 to 102.8)	2.7 (1.6 to 4.1)	73.6 (42.6 to 113.6)	2.3 (1.3 to 3.8)	75.9 (44.1 to 117.3)
Gabon	Diet high in red meat	1.2 (0.6 to 1.8)	33.3 (15.9 to 52)	0.8 (0.3 to 1.3)	34.1 (16.3 to 53)	1.2 (0.5 to 1.9)	30.8 (13.1 to 52.2)	1 (0.4 to 1.7)	31.8 (13.5 to 53.9)
Gabon	Low physical activity	0.3 (0.1 to 0.5)	5.8 (2.8 to 10.8)	0.2 (0.1 to 0.3)	6 (2.9 to 11.1)	0.4 (0.2 to 0.8)	8 (3.3 to 15.4)	0.3 (0.1 to 0.6)	8.2 (3.4 to 15.9)
Gabon	Smoking	0.1 (0.1 to 0.2)	3.2 (1.5 to 5.3)	0.1 (0 to 0.1)	3.3 (1.5 to 5.4)	0.2 (0.1 to 0.3)	3.7 (1.6 to 6.4)	0.1 (0.1 to 0.2)	3.8 (1.7 to 6.6)
Gabon	Secondhand smoke	0.4 (0.1 to 0.7)	12.2 (2.6 to 22.5)	0.3 (0.1 to 0.6)	12.5 (2.7 to 23.1)	0.5 (0.1 to 0.9)	13.4 (2.8 to 26.3)	0.4 (0.1 to 0.8)	13.8 (2.9 to 27.1)
Gabon	Metabolic risks	1.6 (0.4 to 3.3)	29.6 (2 to 66.1)	0.9 (0.1 to 1.9)	30.5 (2.4 to 67.9)	3.3 (1 to 6.9)	57.8 (8.7 to 133.3)	2.1 (0.4 to 4.6)	59.9 (9 to 137.3)
Gabon	High body-mass index	0.5 (0 to 1.3)	6.1 (-8.2 to 23.4)	0.2 (-0.1 to 0.7)	6.3 (-8.4 to 23.9)	1.4 (0.1 to 3.3)	17.8 (-14 to 55)	0.7 (-0.3 to 2)	18.5 (-14.1 to 57)
Gabon	High fasting plasma glucose	1.1 (0.2 to 2.6)	24.3 (4.4 to 56.7)	0.7 (0.1 to 1.6)	25 (4.6 to 58.3)	2 (0.4 to 5)	42.9 (7.9 to 107.1)	1.4 (0.3 to 3.7)	44.3 (8.2 to 111)
Gambia	All risk factors	1 (0.5 to 1.5)	24.7 (14.4 to 38.3)	0.7 (0.4 to 1.1)	25.3 (14.7 to 39.2)	2.1 (1.1 to 3.6)	53.2 (27.6 to 90.4)	1.6 (0.8 to 2.7)	54.8 (28.8 to 93)
Gambia	Behavioral risks	0.6 (0.4 to 0.9)	17.2 (10.6 to 26)	0.4 (0.2 to 0.7)	17.6 (10.8 to 26.7)	1.1 (0.6 to 1.7)	31.7 (18.1 to 50.6)	0.9 (0.5 to 1.5)	32.6 (18.6 to 51.7)
Gambia	Alcohol use	0.2 (0.1 to 0.3)	6.7 (3.8 to 10.8)	0.2 (0.1 to 0.3)	6.9 (3.9 to 11.1)	0.5 (0.3 to 0.9)	16.1 (8.8 to 26.2)	0.4 (0.2 to 0.8)	16.6 (9 to 27)
Gambia	Diet high in red meat	0.1 (0 to 0.1)	2.4 (0.5 to 4.1)	0.1 (0 to 0.1)	2.5 (0.5 to 4.2)	0.1 (0 to 0.2)	3.4 (0.8 to 6.1)	0.1 (0 to 0.2)	3.5 (0.8 to 6.3)
Gambia	Low physical activity	0.1 (0 to 0.1)	1.4 (0.7 to 2.7)	0 (0 to 0.1)	1.4 (0.7 to 2.8)	0.1 (0 to 0.2)	2.5 (1.2 to 4.7)	0.1 (0 to 0.1)	2.6 (1.2 to 4.9)
Gambia	Smoking	0 (0 to 0.1)	0.7 (0.3 to 1.3)	0 (0 to 0)	0.8 (0.3 to 1.3)	0 (0 to 0.1)	0.9 (0.4 to 1.7)	0 (0 to 0.1)	0.9 (0.4 to 1.8)
Gambia	Secondhand smoke	0.2 (0 to 0.4)	6.4 (1.5 to 12)	0.2 (0 to 0.3)	6.6 (1.5 to 12.3)	0.3 (0.1 to 0.6)	9.7 (2 to 19.3)	0.3 (0.1 to 0.6)	10 (2.1 to 19.8)

Location	Risk factor	1990				2019			
		Deaths	YLLs (Years of Life Lost)	YLDs (Years Lived with Disability)	DALYs (Disability-Adjusted Life Years)	Deaths	YLLs (Years of Life Lost)	YLDs (Years Lived with Disability)	DALYs (Disability-Adjusted Life Years)
Gambia	Metabolic risks	0.4 (0.1 to 0.8)	8.2 (1.4 to 18.2)	0.3 (0.1 to 0.5)	8.4 (1.5 to 18.8)	1.1 (0.4 to 2.5)	23.7 (5.5 to 55)	0.7 (0.2 to 1.7)	24.4 (5.7 to 56.4)
Gambia	High body-mass index	0.2 (0 to 0.4)	2.6 (-1.1 to 7.7)	0.1 (0 to 0.3)	2.7 (-1.1 to 7.9)	0.4 (0.1 to 1.1)	7.9 (-2.5 to 23.9)	0.3 (0 to 0.7)	8.1 (-2.6 to 24.5)
Gambia	High fasting plasma glucose	0.3 (0 to 0.6)	5.7 (1 to 14.3)	0.2 (0 to 0.4)	5.9 (1 to 14.8)	0.7 (0.1 to 1.9)	16.7 (3.1 to 42.7)	0.5 (0.1 to 1.3)	17.2 (3.2 to 44.1)
Georgia	All risk factors	4.9 (3.1 to 7.1)	132.3 (77 to 191.8)	6.1 (3.3 to 9.6)	138.4 (80.4 to 200.1)	6 (3.6 to 9.2)	156.2 (91.9 to 239.7)	8.5 (4.6 to 13.9)	164.7 (96.6 to 252.3)
Georgia	Behavioral risks	3 (2 to 3.9)	98.1 (64.1 to 130.3)	4.4 (2.6 to 6.6)	102.4 (66.7 to 135.8)	3 (2 to 4)	91.7 (60.9 to 124)	4.9 (2.8 to 7.3)	96.6 (64 to 130.5)
Georgia	Alcohol use	0.6 (0.3 to 0.9)	22 (11.8 to 34.4)	1 (0.5 to 1.7)	22.9 (12.4 to 35.7)	0.9 (0.5 to 1.3)	29.5 (17.6 to 44.1)	1.6 (0.8 to 2.5)	31.1 (18.6 to 46.3)
Georgia	Diet high in red meat	0.9 (0.2 to 1.2)	28.1 (8.2 to 38.2)	1.3 (0.4 to 2)	29.4 (8.5 to 40.1)	0.7 (0.2 to 1)	20.4 (4.9 to 30.4)	1.1 (0.3 to 1.8)	21.5 (5.1 to 31.9)
Georgia	Low physical activity	0.2 (0.1 to 0.4)	5.2 (3.2 to 10.2)	0.2 (0.1 to 0.5)	5.4 (3.4 to 10.8)	0.2 (0.1 to 0.4)	4.9 (2.9 to 9.4)	0.3 (0.1 to 0.6)	5.2 (3.1 to 9.9)
Georgia	Smoking	0.4 (0.3 to 0.6)	13.6 (8 to 20.7)	0.6 (0.3 to 1)	14.3 (8.3 to 21.6)	0.4 (0.3 to 0.6)	12.1 (7 to 18.1)	0.7 (0.3 to 1)	12.8 (7.4 to 19.1)
Georgia	Secondhand smoke	1 (0.2 to 1.7)	33.1 (7.7 to 56.6)	1.5 (0.4 to 2.7)	34.6 (8.1 to 59.1)	0.9 (0.2 to 1.7)	28.9 (6.5 to 51.6)	1.5 (0.4 to 2.8)	30.4 (6.9 to 54.6)
Georgia	Metabolic risks	2.1 (0.5 to 4.1)	37.4 (-7.9 to 92.1)	1.9 (-0.2 to 4.4)	39.3 (-8 to 96.5)	3.4 (1.1 to 6.5)	71.6 (12.8 to 152.2)	4 (0.8 to 8.8)	75.6 (13.8 to 160.5)
Georgia	High body-mass index	1 (-0.1 to 2.3)	7 (-26.3 to 38.5)	0.4 (-1.1 to 2)	7.5 (-27.3 to 40.3)	1.3 (0.1 to 2.7)	17.7 (-12.1 to 50.7)	1 (-0.6 to 2.9)	18.7 (-12.5 to 53)
Georgia	High fasting plasma glucose	1.2 (0.2 to 2.8)	31.8 (6 to 74.7)	1.5 (0.3 to 3.7)	33.3 (6.3 to 78.9)	2.3 (0.4 to 5.1)	57.3 (10.6 to 131.2)	3.2 (0.6 to 7.6)	60.5 (11.1 to 138.4)
Germany	All risk factors	10.1 (8.2 to 12.2)	266.8 (219.5 to 315.3)	19.6 (13 to 27.5)	286.4 (236.5 to 339.7)	8.2 (6.3 to 10.4)	189.6 (149.6 to 234.8)	23.7 (14.3 to 35.4)	213.3 (168.4 to 266)
Germany	Behavioral risks	8 (6.9 to 9.1)	228.3 (195.9 to 261.1)	16.3 (10.9 to 22.6)	244.6 (208.8 to 279.5)	5.8 (4.9 to 6.7)	147.9 (125.7 to 171.2)	18.3 (11.6 to 26.9)	166.3 (140.4 to 193.8)
Germany	Alcohol use	4.8 (3.8 to 5.8)	135.3 (108.8 to 164.5)	9.7 (6.2 to 14)	145 (116.4 to 175.9)	3.5 (2.7 to 4.3)	88.5 (69.8 to 109.4)	11 (6.9 to 16.3)	99.5 (78.3 to 123.1)
Germany	Diet high in red meat	1.5 (0.8 to 2)	42.1 (20.7 to 57)	3 (1.4 to 4.7)	45.1 (22.3 to 61.2)	1.1 (0.5 to 1.5)	27.5 (13.8 to 37.6)	3.4 (1.4 to 5.6)	30.9 (15.3 to 42.6)
Germany	Low physical activity	0.3 (0.1 to 0.6)	6.3 (2.7 to 13)	0.5 (0.2 to 1.2)	6.8 (2.9 to 14.2)	0.3 (0.1 to 0.5)	5.5 (2.1 to 11.1)	0.7 (0.3 to 1.5)	6.2 (2.4 to 12.5)
Germany	Smoking	2 (1.4 to 2.6)	58.6 (40.5 to 76.9)	4.1 (2.4 to 6.2)	62.7 (43.2 to 82.2)	1.3 (0.9 to 1.7)	35.1 (24.8 to 45.7)	4.3 (2.4 to 6.7)	39.4 (27.8 to 51.6)
Germany	Secondhand smoke	0.4 (0.1 to 0.7)	13.8 (3.3 to 23.8)	0.9 (0.2 to 1.7)	14.7 (3.5 to 25.3)	0.2 (0.1 to 0.4)	7.6 (1.8 to 13.5)	0.9 (0.2 to 1.8)	8.6 (2.1 to 15.1)
Germany	Metabolic risks	3 (1.1 to 5.6)	55.3 (12.9 to 111)	4.7 (1.4 to 9.7)	60.1 (14.3 to 119.7)	3.3 (1.3 to 6)	58.8 (18.4 to 113.6)	7.5 (2.2 to 15.7)	66.4 (20.9 to 127.6)
Germany	High body-mass index	1.1 (0.2 to 2.3)	17.1 (-4.4 to 42.2)	1.6 (0 to 3.7)	18.7 (-4.3 to 46)	1.2 (0.3 to 2.4)	18.4 (-0.7 to 41.3)	2.3 (0 to 5.3)	20.7 (-0.7 to 46.1)
Germany	High fasting plasma glucose	1.9 (0.4 to 4.3)	40.5 (7.8 to 91.8)	3.4 (0.6 to 7.9)	43.9 (8.5 to 98.9)	2.2 (0.5 to 4.8)	43.5 (8.9 to 94.3)	5.6 (1.1 to 13.5)	49 (10 to 107)
Ghana	All risk factors	3.5 (2.1 to 5.3)	89.5 (54.5 to 135.1)	2.3 (1.3 to 3.6)	91.8 (55.9 to 139)	5.7 (3.4 to 8.9)	129.9 (72.1 to 205.1)	4.2 (2.3 to 7.1)	134.2 (74.7 to 211.3)
Ghana	Behavioral risks	2.1 (1.5 to 2.9)	63.9 (43.5 to 89.7)	1.5 (0.9 to 2.3)	65.4 (44.5 to 91.7)	2.6 (1.9 to 3.7)	75.4 (51 to 106.2)	2.3 (1.4 to 3.5)	77.7 (52.6 to 109.7)
Ghana	Alcohol use	1.3 (0.8 to 1.9)	40.5 (25.8 to 59.7)	1 (0.6 to 1.5)	41.5 (26.4 to 61.2)	1.6 (1 to 2.4)	47.4 (30.2 to 69.4)	1.4 (0.8 to 2.3)	48.9 (31.1 to 71.5)
Ghana	Diet high in red meat	0.3 (0.1 to 0.4)	8 (1.6 to 12.6)	0.2 (0 to 0.3)	8.2 (1.7 to 12.9)	0.4 (0.1 to 0.6)	10.4 (2.1 to 16.7)	0.3 (0.1 to 0.6)	10.7 (2.1 to 17.1)
Ghana	Low physical activity	0.2 (0.1 to 0.4)	4.8 (2.6 to 9)	0.1 (0.1 to 0.3)	4.9 (2.6 to 9.2)	0.3 (0.1 to 0.5)	6.4 (3.4 to 11.8)	0.2 (0.1 to 0.4)	6.6 (3.5 to 12.1)
Ghana	Smoking	0.2 (0.1 to 0.2)	3.8 (2.1 to 6.2)	0.1 (0.1 to 0.2)	3.9 (2.2 to 6.4)	0.1 (0.1 to 0.2)	3.5 (1.9 to 5.8)	0.1 (0.1 to 0.2)	3.6 (2 to 6)
Ghana	Secondhand smoke	0.2 (0.1 to 0.5)	8.4 (2 to 15.9)	0.2 (0 to 0.4)	8.6 (2 to 16.2)	0.3 (0.1 to 0.6)	9.7 (2.2 to 18.5)	0.3 (0.1 to 0.6)	10 (2.2 to 19)
Ghana	Metabolic risks	1.4 (0.4 to 3.1)	28 (2.5 to 66)	0.8 (0.2 to 1.9)	28.8 (2.6 to 67.9)	3.3 (1.1 to 6.7)	59.7 (7 to 131)	2.1 (0.3 to 4.6)	61.8 (7.3 to 134.5)
Ghana	High body-mass index	0.3 (0 to 0.9)	3.2 (-8 to 14.6)	0.1 (-0.1 to 0.5)	3.3 (-8.1 to 15.1)	1.4 (0.1 to 3.1)	17.3 (-17.2 to 54.2)	0.7 (-0.4 to 1.9)	18 (-17.5 to 56.1)
Ghana	High fasting plasma glucose	1.1 (0.2 to 2.8)	25.4 (4.4 to 60.9)	0.7 (0.1 to 1.7)	26.1 (4.6 to 62.4)	2.1 (0.4 to 4.9)	45.4 (8.5 to 108.2)	1.5 (0.3 to 3.7)	46.9 (8.8 to 111.7)
Greece	All risk factors	6.6 (5.2 to 8)	175 (139.9 to 208.3)	15.5 (10.1 to 21.8)	190.5 (151.8 to 226.2)	6.7 (5 to 8.7)	157.1 (122 to 199)	19.6 (12.2 to 29.6)	176.6 (135.8 to 224.3)
Greece	Behavioral risks	5 (4.1 to 5.8)	147.4 (123.5 to 170.3)	12.7 (8.4 to 18)	160.2 (134 to 184.5)	4.6 (3.8 to 5.4)	122.9 (101.8 to 145)	15.2 (9.3 to 22.9)	138.1 (113.5 to 163.7)
Greece	Alcohol use	2.2 (1.7 to 2.6)	67.5 (54 to 81)	5.8 (3.7 to 8.4)	73.3 (59 to 87.9)	1.5 (1.1 to 2)	43.2 (32.6 to 55.3)	5.3 (3.2 to 8.4)	48.5 (36.6 to 62.8)
Greece	Diet high in red meat	1.2 (0.6 to 1.6)	33.4 (16.9 to 44.9)	2.9 (1.3 to 4.7)	36.3 (18.2 to 49.1)	1.2 (0.6 to 1.6)	28.9 (14.7 to 40.2)	3.6 (1.6 to 6.1)	32.5 (16.7 to 44.9)
Greece	Low physical activity	0.2 (0.1 to 0.4)	4.7 (2.2 to 9.8)	0.4 (0.2 to 1)	5.2 (2.4 to 10.9)	0.3 (0.1 to 0.5)	4.8 (2 to 9.6)	0.6 (0.2 to 1.4)	5.4 (2.3 to 10.9)

Location	Risk factor	1990				2019			
		Deaths	YLLs (Years of Life Lost)	YLDs (Years Lived with Disability)	DALYs (Disability-Adjusted Life Years)	Deaths	YLLs (Years of Life Lost)	YLDs (Years Lived with Disability)	DALYs (Disability-Adjusted Life Years)
Greece	Smoking	1.2 (0.9 to 1.6)	37.8 (25.9 to 49.8)	3.3 (1.9 to 5)	41 (28.1 to 54.3)	1.6 (1.2 to 2.1)	46.2 (33.7 to 59.5)	5.7 (3.2 to 9)	51.9 (37.7 to 67.5)
Greece	Secondhand smoke	0.6 (0.1 to 1.1)	18.5 (4.4 to 31.8)	1.6 (0.4 to 2.9)	20.1 (4.8 to 34.9)	0.5 (0.1 to 0.8)	12.4 (2.9 to 21.9)	1.5 (0.3 to 3)	13.9 (3.3 to 25)
Greece	Metabolic risks	1.9 (0.7 to 3.6)	34.7 (6.6 to 70.6)	3.4 (0.8 to 6.8)	38.1 (7.9 to 77.2)	2.6 (1 to 4.7)	44.1 (13 to 87.9)	5.6 (1.6 to 11.6)	49.7 (14.2 to 98.8)
Greece	High body-mass index	1 (0.2 to 2.1)	15 (-4.1 to 38.9)	1.5 (-0.2 to 3.7)	16.5 (-4.3 to 42.1)	1.2 (0.3 to 2.4)	17 (-1.4 to 39.7)	2.2 (-0.1 to 5.5)	19.2 (-1.6 to 44.4)
Greece	High fasting plasma glucose	1 (0.2 to 2.3)	20.9 (3.8 to 47.3)	2 (0.4 to 4.9)	22.9 (4.2 to 52.3)	1.5 (0.3 to 3.4)	29 (5.5 to 66.7)	3.7 (0.7 to 9.2)	32.7 (6.3 to 74.6)
Greenland	All risk factors	8.5 (6.5 to 10.9)	243.2 (183.4 to 311.8)	10.6 (6.8 to 15.2)	253.8 (190.5 to 325.3)	6.4 (4.7 to 8.7)	164.7 (119 to 224)	10.4 (6.6 to 15.4)	175.1 (126.2 to 239.1)
Greenland	Behavioral risks	7.3 (5.7 to 9.2)	221.2 (169.2 to 283.7)	9.4 (6.1 to 13.6)	230.6 (176.1 to 294.6)	5 (3.7 to 6.5)	139.1 (101.8 to 185.5)	8.5 (5.5 to 12.5)	147.7 (108.4 to 197.2)
Greenland	Alcohol use	2.5 (1.6 to 3.5)	78.4 (51 to 112.2)	3.2 (1.8 to 5)	81.6 (53.1 to 116.8)	1.7 (1.1 to 2.5)	52.1 (32.8 to 75.5)	3.1 (1.8 to 4.8)	55.2 (35 to 79.6)
Greenland	Diet high in red meat	1.4 (0.7 to 2)	42.8 (20.5 to 62.2)	1.8 (0.8 to 2.9)	44.6 (21.3 to 64.7)	1 (0.5 to 1.5)	28.1 (13.7 to 42.8)	1.7 (0.8 to 2.9)	29.8 (14.6 to 45.5)
Greenland	Low physical activity	0.3 (0.1 to 0.6)	7.5 (3 to 16)	0.4 (0.1 to 0.8)	7.8 (3.1 to 16.7)	0.3 (0.1 to 0.5)	5.7 (2 to 11.8)	0.4 (0.1 to 0.8)	6.1 (2.1 to 12.6)
Greenland	Smoking	3.2 (2.2 to 4.3)	92.8 (61.7 to 128.3)	4 (2.2 to 6.3)	96.8 (64.2 to 133.6)	2.1 (1.5 to 3)	56.7 (37.1 to 81.2)	3.6 (2.1 to 5.6)	60.2 (39.7 to 85.8)
Greenland	Secondhand smoke	0.8 (0.2 to 1.4)	25.3 (6.1 to 44.6)	1 (0.2 to 1.9)	26.3 (6.3 to 46.3)	0.4 (0.1 to 0.7)	12.1 (2.6 to 22.2)	0.7 (0.1 to 1.3)	12.8 (2.8 to 23.6)
Greenland	Metabolic risks	1.7 (0.5 to 3.4)	30.5 (-0.3 to 70.1)	1.7 (0.3 to 3.5)	32.2 (0.1 to 73.2)	2 (0.7 to 3.8)	35.6 (5.1 to 78.3)	2.6 (0.5 to 5.2)	38.2 (6 to 84)
Greenland	High body-mass index	1.2 (0.1 to 2.6)	18.2 (-9.1 to 51.8)	1.1 (-0.1 to 2.7)	19.4 (-9.3 to 54.1)	1.1 (0.2 to 2.4)	15.8 (-5.8 to 45.4)	1.3 (-0.2 to 3.2)	17.1 (-6 to 48.5)
Greenland	High fasting plasma glucose	0.5 (0.1 to 1.3)	13 (2.2 to 31.5)	0.6 (0.1 to 1.6)	13.6 (2.3 to 33.2)	1 (0.2 to 2.3)	21.3 (3.8 to 51.2)	1.4 (0.3 to 3.6)	22.7 (4.1 to 54.7)
Grenada	All risk factors	5.4 (3.5 to 8)	142.5 (94.1 to 210.3)	5.6 (3.3 to 8.8)	148.1 (97.6 to 218.1)	7.8 (4.8 to 11.8)	186.1 (114.5 to 280.7)	9.9 (5.5 to 16)	196 (120.2 to 295)
Grenada	Behavioral risks	3 (2.4 to 3.7)	92 (72.9 to 114.9)	3.4 (2.2 to 4.8)	95.4 (75.7 to 118.7)	3.6 (2.8 to 4.5)	105.2 (81 to 131.6)	5.3 (3.4 to 7.5)	110.4 (85.3 to 137.8)
Grenada	Alcohol use	1.5 (1.2 to 2)	53 (39.6 to 68.6)	1.9 (1.2 to 2.7)	54.9 (41.3 to 71.1)	1.8 (1.4 to 2.3)	59.3 (44.1 to 76.8)	2.9 (1.8 to 4.3)	62.2 (46.4 to 80.6)
Grenada	Diet high in red meat	0.3 (0.1 to 0.4)	9 (2 to 12.9)	0.3 (0.1 to 0.5)	9.3 (2 to 13.3)	0.5 (0.1 to 0.7)	13.2 (2.6 to 19)	0.7 (0.1 to 1.1)	13.8 (2.8 to 19.9)
Grenada	Low physical activity	0.4 (0.1 to 0.7)	8.6 (3.3 to 17.2)	0.4 (0.1 to 0.7)	9 (3.4 to 17.9)	0.5 (0.2 to 1)	11.5 (4.1 to 22.4)	0.6 (0.2 to 1.3)	12.1 (4.3 to 23.7)
Grenada	Smoking	0.5 (0.3 to 0.7)	12.7 (7.7 to 18.6)	0.5 (0.3 to 0.8)	13.2 (8 to 19.3)	0.5 (0.3 to 0.8)	13 (8 to 19.4)	0.7 (0.4 to 1.1)	13.7 (8.3 to 20.3)
Grenada	Secondhand smoke	0.4 (0.1 to 0.7)	12.4 (2.7 to 22.3)	0.5 (0.1 to 0.9)	12.9 (2.8 to 23.1)	0.4 (0.1 to 0.8)	12.7 (3 to 23)	0.6 (0.1 to 1.2)	13.4 (3.1 to 24.1)
Grenada	Metabolic risks	2.7 (0.8 to 5.5)	56.8 (11.1 to 126.2)	2.5 (0.6 to 5.4)	59.2 (11.7 to 132.2)	4.7 (1.6 to 9.1)	91.9 (15.8 to 195)	5.2 (1.2 to 11.2)	97.1 (16.8 to 204.2)
Grenada	High body-mass index	0.6 (-0.1 to 1.5)	6.9 (-11.5 to 27.4)	0.4 (-0.3 to 1.3)	7.3 (-11.8 to 28.7)	1.3 (0.1 to 3.1)	15.3 (-18.9 to 51.9)	1.1 (-0.7 to 3.2)	16.4 (-19.8 to 55.4)
Grenada	High fasting plasma glucose	2.2 (0.4 to 4.9)	51.7 (9.7 to 118.3)	2.1 (0.4 to 5)	53.8 (10.1 to 123.4)	3.6 (0.8 to 7.8)	81.6 (17 to 178)	4.4 (0.9 to 10.2)	86 (17.8 to 187.5)
Guam	All risk factors	6.4 (3.9 to 9.3)	146.6 (91 to 207)	6.7 (3.7 to 10.4)	153.2 (94.9 to 215.6)	7.1 (4.1 to 10.2)	179 (105.9 to 255.4)	9.2 (5.2 to 14.2)	188.2 (111.3 to 266)
Guam	Behavioral risks	2.2 (1.5 to 2.9)	55.4 (37.3 to 75.5)	2.5 (1.5 to 3.8)	57.9 (39.3 to 79)	2.2 (1.5 to 3)	65 (42.6 to 89.1)	3.3 (1.9 to 5.1)	68.3 (44.8 to 93.4)
Guam	Alcohol use	0.1 (0 to 0.3)	4.2 (0 to 10.9)	0.2 (0 to 0.5)	4.3 (0 to 11.2)	0.2 (0 to 0.6)	9.1 (0 to 20.1)	0.5 (0 to 1.1)	9.5 (0 to 21.2)
Guam	Diet high in red meat	0.6 (0.2 to 0.9)	15.8 (6.3 to 22.8)	0.7 (0.3 to 1.1)	16.5 (6.6 to 23.9)	0.6 (0.3 to 0.9)	18.5 (7.5 to 28.1)	0.9 (0.4 to 1.5)	19.5 (7.9 to 29.4)
Guam	Low physical activity	0.3 (0.1 to 0.5)	5.5 (2.2 to 10.3)	0.3 (0.1 to 0.5)	5.7 (2.3 to 10.8)	0.3 (0.1 to 0.5)	6.1 (2.3 to 11.9)	0.3 (0.1 to 0.7)	6.4 (2.4 to 12.6)
Guam	Smoking	0.6 (0.3 to 0.9)	16 (8.7 to 25.4)	0.7 (0.4 to 1.2)	16.7 (9.1 to 26.5)	0.6 (0.4 to 0.9)	17.3 (9.3 to 26.5)	0.9 (0.4 to 1.5)	18.2 (9.7 to 27.9)
Guam	Secondhand smoke	0.7 (0.1 to 1.2)	17 (4 to 30.5)	0.8 (0.2 to 1.4)	17.8 (4.2 to 31.9)	0.6 (0.2 to 1)	17.5 (4.5 to 31.7)	0.9 (0.2 to 1.6)	18.4 (4.7 to 33.1)
Guam	Metabolic risks	4.9 (2.2 to 8)	105.3 (46.7 to 170)	4.8 (2 to 8.3)	110.1 (48.7 to 178.1)	5.6 (2.4 to 8.8)	131.7 (56.7 to 209.4)	6.7 (2.9 to 11.3)	138.5 (60 to 219.8)
Guam	High body-mass index	3.9 (1.3 to 6.8)	84.3 (27.5 to 147.3)	3.8 (1.2 to 7.1)	88.1 (29.5 to 152.2)	4.4 (1.6 to 7.5)	103.2 (36.9 to 175.6)	5.3 (1.9 to 9.4)	108.5 (39 to 184.1)
Guam	High fasting plasma glucose	1.4 (0.3 to 3.1)	27.7 (5.5 to 63.2)	1.3 (0.2 to 3.1)	29 (5.7 to 66.4)	1.6 (0.3 to 3.8)	38.9 (7.9 to 89.6)	2 (0.4 to 4.8)	40.9 (8.3 to 94.1)
Guatemala	All risk factors	1.2 (0.7 to 1.9)	31.2 (19.1 to 48.1)	0.9 (0.5 to 1.5)	32.1 (19.7 to 49.6)	2.1 (1.1 to 3.8)	50 (24.5 to 91.3)	2.3 (1 to 4.2)	52.3 (25.6 to 95.5)
Guatemala	Behavioral risks	0.6 (0.4 to 0.8)	19.7 (14.1 to 25.5)	0.5 (0.3 to 0.8)	20.2 (14.5 to 26.3)	0.6 (0.4 to 0.9)	18.9 (12.4 to 26.8)	0.8 (0.5 to 1.2)	19.7 (12.8 to 27.9)
Guatemala	Alcohol use	0.3 (0.2 to 0.3)	9.1 (6.5 to 12.2)	0.2 (0.1 to 0.4)	9.3 (6.7 to 12.5)	0.2 (0.1 to 0.3)	6.6 (4.1 to 9.8)	0.3 (0.2 to 0.4)	6.8 (4.3 to 10.1)
Guatemala	Diet high in red meat	0.1 (0 to 0.1)	2.7 (0.6 to 4)	0.1 (0 to 0.1)	2.8 (0.6 to 4.1)	0.1 (0 to 0.2)	3.8 (0.8 to 6.2)	0.2 (0 to 0.3)	4 (0.8 to 6.5)

Location	Risk factor	1990				2019			
		Deaths	YLLs (Years of Life Lost)	YLDs (Years Lived with Disability)	DALYs (Disability-Adjusted Life Years)	Deaths	YLLs (Years of Life Lost)	YLDs (Years Lived with Disability)	DALYs (Disability-Adjusted Life Years)
Guatemala	Low physical activity	0 (0 to 0.1)	0.9 (0.6 to 2.7)	0 (0 to 0.1)	0.9 (0.6 to 2.8)	0 (0 to 0.1)	1 (0.6 to 3)	0 (0 to 0.1)	1.1 (0.7 to 3.1)
Guatemala	Smoking	0.1 (0.1 to 0.2)	3.3 (1.7 to 5.3)	0.1 (0 to 0.2)	3.4 (1.7 to 5.5)	0.1 (0.1 to 0.2)	3.2 (1.6 to 5.2)	0.1 (0.1 to 0.2)	3.3 (1.6 to 5.5)
Guatemala	Secondhand smoke	0.1 (0 to 0.2)	4.3 (1.1 to 7.7)	0.1 (0 to 0.2)	4.4 (1.1 to 7.9)	0.2 (0 to 0.3)	4.8 (1.1 to 8.9)	0.2 (0 to 0.4)	5 (1.2 to 9.2)
Guatemala	Metabolic risks	0.6 (0.2 to 1.3)	12.3 (1.2 to 28.4)	0.4 (0.1 to 1)	12.7 (1.3 to 29.3)	1.5 (0.5 to 3.2)	33.1 (7.6 to 72)	1.6 (0.4 to 3.5)	34.7 (8.1 to 74.7)
Guatemala	High body-mass index	0.1 (0 to 0.3)	0.9 (-4 to 5.1)	0.1 (-0.1 to 0.2)	1 (-4 to 5.4)	0.4 (0.1 to 0.9)	5.6 (-3.3 to 16.2)	0.3 (-0.1 to 0.8)	5.9 (-3.3 to 16.9)
Guatemala	High fasting plasma glucose	0.5 (0.1 to 1.2)	11.6 (2.2 to 27.2)	0.4 (0.1 to 0.9)	12 (2.3 to 27.9)	1.2 (0.2 to 2.8)	28.9 (5.6 to 67.7)	1.3 (0.3 to 3.3)	30.2 (5.8 to 70.7)
Guinea	All risk factors	1.6 (0.9 to 2.5)	39.8 (22.1 to 62.3)	1 (0.5 to 1.6)	40.8 (22.7 to 64.2)	2.7 (1.5 to 4.6)	63.9 (34.5 to 108.7)	1.7 (0.8 to 3.1)	65.6 (35.3 to 111.5)
Guinea	Behavioral risks	0.8 (0.5 to 1.2)	24.1 (15.2 to 34.8)	0.5 (0.3 to 0.8)	24.6 (15.6 to 35.7)	1.1 (0.7 to 1.7)	32.7 (20.1 to 48.7)	0.8 (0.4 to 1.3)	33.5 (20.6 to 49.9)
Guinea	Alcohol use	0.2 (0.1 to 0.3)	6.4 (3.6 to 9.9)	0.1 (0.1 to 0.2)	6.5 (3.7 to 10.1)	0.3 (0.2 to 0.5)	9.5 (5.2 to 15.4)	0.2 (0.1 to 0.4)	9.7 (5.3 to 15.8)
Guinea	Diet high in red meat	0.1 (0 to 0.2)	3.9 (1 to 5.8)	0.1 (0 to 0.1)	4 (1.1 to 6)	0.2 (0 to 0.4)	6.9 (1.4 to 11.5)	0.2 (0 to 0.3)	7 (1.4 to 11.8)
Guinea	Low physical activity	0.1 (0.1 to 0.2)	2.9 (1.6 to 5.3)	0.1 (0 to 0.1)	2.9 (1.6 to 5.5)	0.2 (0.1 to 0.3)	3.6 (1.9 to 6.8)	0.1 (0 to 0.2)	3.7 (1.9 to 7)
Guinea	Smoking	0.1 (0 to 0.1)	1.8 (0.9 to 2.9)	0 (0 to 0.1)	1.8 (0.9 to 3)	0.1 (0.1 to 0.2)	2.6 (1.3 to 4.6)	0.1 (0 to 0.1)	2.7 (1.3 to 4.7)
Guinea	Secondhand smoke	0.3 (0.1 to 0.6)	9.7 (2.2 to 17.5)	0.2 (0 to 0.4)	9.9 (2.2 to 17.8)	0.4 (0.1 to 0.7)	10.9 (2.4 to 20.8)	0.3 (0.1 to 0.5)	11.2 (2.5 to 21.3)
Guinea	Metabolic risks	0.8 (0.2 to 1.7)	16.7 (2.7 to 35.7)	0.5 (0.1 to 0.9)	17.1 (2.9 to 36.6)	1.6 (0.5 to 3.4)	33.2 (6.9 to 74.6)	1 (0.2 to 2.1)	34.2 (7.3 to 76.2)
Guinea	High body-mass index	0.3 (0 to 0.7)	4.9 (-2.5 to 14.7)	0.2 (0 to 0.4)	5 (-2.5 to 15.2)	0.6 (0.1 to 1.3)	9.2 (-4.6 to 26.1)	0.3 (0 to 0.8)	9.5 (-4.5 to 26.7)
Guinea	High fasting plasma glucose	0.5 (0.1 to 1.3)	12.1 (2.2 to 29.3)	0.3 (0.1 to 0.8)	12.4 (2.2 to 30)	1.1 (0.2 to 2.6)	25 (4.6 to 60.1)	0.7 (0.1 to 1.8)	25.7 (4.7 to 61.8)
Guinea-Bissau	All risk factors	2.4 (1.4 to 3.7)	67 (39.9 to 104.2)	1.5 (0.8 to 2.5)	68.5 (40.9 to 106.2)	3.7 (2 to 6.5)	96.2 (52.5 to 170.3)	2.5 (1.3 to 4.4)	98.7 (53.9 to 175)
Guinea-Bissau	Behavioral risks	1.6 (1 to 2.3)	50.7 (31.7 to 73.6)	1.1 (0.6 to 1.6)	51.7 (32.5 to 75.1)	2 (1.2 to 3.2)	62.2 (37.4 to 99.3)	1.5 (0.8 to 2.5)	63.7 (38.2 to 101.7)
Guinea-Bissau	Alcohol use	0.8 (0.5 to 1.2)	26.2 (15.7 to 39.8)	0.5 (0.3 to 0.9)	26.7 (16.1 to 40.7)	1 (0.6 to 1.6)	31.7 (17.6 to 52.7)	0.8 (0.4 to 1.3)	32.5 (18.1 to 54.1)
Guinea-Bissau	Diet high in red meat	0.3 (0.1 to 0.5)	10.3 (1.9 to 16.8)	0.2 (0 to 0.4)	10.5 (1.9 to 17.1)	0.4 (0.1 to 0.8)	13.5 (2.7 to 23.7)	0.3 (0.1 to 0.6)	13.8 (2.8 to 24.3)
Guinea-Bissau	Low physical activity	0.1 (0.1 to 0.2)	3.4 (1.7 to 6.5)	0.1 (0 to 0.2)	3.4 (1.8 to 6.7)	0.2 (0.1 to 0.4)	4.5 (2.3 to 8.6)	0.1 (0.1 to 0.2)	4.6 (2.4 to 8.8)
Guinea-Bissau	Smoking	0.1 (0 to 0.1)	1.7 (0.8 to 3.1)	0 (0 to 0.1)	1.8 (0.8 to 3.2)	0.1 (0 to 0.1)	1.7 (0.8 to 3.2)	0 (0 to 0.1)	1.8 (0.8 to 3.2)
Guinea-Bissau	Secondhand smoke	0.3 (0.1 to 0.6)	10.8 (2.3 to 20.6)	0.2 (0 to 0.4)	11.1 (2.3 to 21.1)	0.4 (0.1 to 0.8)	12.8 (2.8 to 26.8)	0.3 (0.1 to 0.6)	13.1 (2.8 to 27.4)
Guinea-Bissau	Metabolic risks	0.9 (0.2 to 1.9)	17.9 (1.8 to 43.9)	0.5 (0.1 to 1.1)	18.4 (1.9 to 44.9)	1.8 (0.5 to 4.1)	37.1 (6 to 92.3)	1.1 (0.3 to 2.5)	38.2 (6.2 to 94.5)
Guinea-Bissau	High body-mass index	0.2 (0 to 0.6)	2.5 (-4.7 to 11.4)	0.1 (0 to 0.3)	2.6 (-4.8 to 11.8)	0.5 (0 to 1.3)	6 (-8.4 to 23.7)	0.2 (-0.1 to 0.7)	6.2 (-8.5 to 24.5)
Guinea-Bissau	High fasting plasma glucose	0.7 (0.1 to 1.6)	15.8 (2.9 to 38.7)	0.4 (0.1 to 1)	16.2 (2.9 to 39.6)	1.4 (0.3 to 3.5)	32.2 (5.7 to 84.6)	0.9 (0.2 to 2.3)	33.1 (5.9 to 86.6)
Guyana	All risk factors	5 (3 to 7.8)	124.5 (74.2 to 196.8)	4.2 (2.3 to 7.1)	128.7 (76.9 to 202.9)	5.8 (3 to 10.1)	143 (69.7 to 256.4)	5.9 (2.8 to 11)	148.9 (72.7 to 268.1)
Guyana	Behavioral risks	2.4 (1.8 to 3)	69.3 (50.9 to 88.8)	2.2 (1.4 to 3.2)	71.5 (52.6 to 91.7)	2.2 (1.5 to 3)	64.7 (43.1 to 90.4)	2.5 (1.5 to 3.8)	67.2 (45.1 to 93)
Guyana	Alcohol use	0.9 (0.7 to 1.3)	30.5 (21.6 to 41.1)	0.9 (0.6 to 1.4)	31.5 (22.2 to 42.5)	0.7 (0.5 to 1.1)	25.3 (16.1 to 38.7)	0.9 (0.5 to 1.5)	26.2 (16.6 to 40)
Guyana	Diet high in red meat	0.2 (0.1 to 0.3)	6.2 (1.6 to 9.2)	0.2 (0 to 0.3)	6.4 (1.6 to 9.5)	0.2 (0.1 to 0.4)	6.8 (1.7 to 11.1)	0.3 (0.1 to 0.5)	7.1 (1.7 to 11.5)
Guyana	Low physical activity	0.4 (0.1 to 0.7)	8.5 (3.2 to 16.5)	0.3 (0.1 to 0.6)	8.7 (3.3 to 17.1)	0.4 (0.1 to 0.8)	10.5 (3.6 to 21.1)	0.4 (0.1 to 0.9)	10.9 (3.7 to 21.9)
Guyana	Smoking	0.4 (0.3 to 0.6)	11.5 (6.7 to 17.2)	0.4 (0.2 to 0.6)	11.9 (6.9 to 17.7)	0.4 (0.2 to 0.6)	9.9 (5.7 to 15.8)	0.4 (0.2 to 0.7)	10.3 (6 to 16.5)
Guyana	Secondhand smoke	0.5 (0.1 to 0.9)	15.4 (3.2 to 27.4)	0.5 (0.1 to 0.9)	15.8 (3.3 to 28.2)	0.5 (0.1 to 0.8)	14.4 (3.4 to 26.8)	0.5 (0.1 to 1)	14.9 (3.6 to 27.8)
Guyana	Metabolic risks	2.9 (0.9 to 5.9)	61.6 (12.9 to 135.4)	2.2 (0.6 to 4.8)	63.8 (13.5 to 140.6)	4 (1.2 to 8.4)	85.8 (15.1 to 196.2)	3.7 (0.9 to 8.5)	89.6 (15.9 to 204.9)
Guyana	High body-mass index	0.7 (0 to 1.5)	8.5 (-8.3 to 28.4)	0.4 (-0.1 to 1.1)	8.9 (-8.4 to 29.3)	0.9 (0 to 2.2)	8.2 (-19.7 to 36.8)	0.6 (-0.5 to 1.8)	8.8 (-20.2 to 38.8)
Guyana	High fasting plasma glucose	2.3 (0.5 to 5.2)	55.2 (11 to 127.2)	1.9 (0.4 to 4.6)	57.1 (11.4 to 132.9)	3.3 (0.7 to 7.4)	81.7 (17 to 191.1)	3.3 (0.7 to 8.1)	85.1 (17.9 to 198.3)
Haiti	All risk factors	4.4 (2.5 to 7.4)	122.1 (70.8 to 198.9)	2.9 (1.5 to 5)	125.1 (72.7 to 203.4)	5.8 (2.9 to 10.5)	154.2 (76 to 280.6)	4.4 (2.1 to 8.3)	158.5 (78.7 to 288.3)
Haiti	Behavioral risks	2.4 (1.7 to 3.1)	73.8 (51.5 to 100.8)	1.7 (1 to 2.5)	75.5 (52.8 to 103.3)	2.5 (1.6 to 3.7)	75.4 (46.7 to 114.3)	2 (1.1 to 3.2)	77.4 (48 to 117.4)
Haiti	Alcohol use	1 (0.7 to 1.4)	34.3 (23.1 to 48.9)	0.7 (0.4 to 1.1)	35.1 (23.6 to 50)	1.1 (0.7 to 1.7)	37.5 (22.6 to 58.3)	1 (0.5 to 1.6)	38.4 (23.2 to 59.7)
Haiti	Diet high in red meat	0.3 (0.1 to 0.5)	10.6 (2 to 17.1)	0.2 (0 to 0.4)	10.8 (2 to 17.5)	0.4 (0.1 to 0.7)	12 (2.3 to 21.5)	0.3 (0.1 to 0.6)	12.3 (2.3 to 22.1)
Haiti	Low physical activity	0.4 (0.1 to 0.7)	8.7 (3.4 to 18.4)	0.2 (0.1 to 0.5)	8.9 (3.5 to 18.9)	0.4 (0.1 to 0.8)	9.8 (3.3 to 21.4)	0.3 (0.1 to 0.6)	10.1 (3.5 to 22)

Location	Risk factor	1990				2019			
		Deaths	YLLs (Years of Life Lost)	YLDs (Years Lived with Disability)	DALYs (Disability-Adjusted Life Years)	Deaths	YLLs (Years of Life Lost)	YLDs (Years Lived with Disability)	DALYs (Disability-Adjusted Life Years)
Haiti	Smoking	0.4 (0.2 to 0.6)	10.5 (5.1 to 17.2)	0.3 (0.1 to 0.4)	10.8 (5.2 to 17.6)	0.3 (0.2 to 0.6)	8.6 (4 to 15.3)	0.2 (0.1 to 0.5)	8.8 (4.1 to 15.7)
Haiti	Secondhand smoke	0.4 (0.1 to 0.7)	12.1 (2.6 to 23.7)	0.3 (0.1 to 0.5)	12.4 (2.7 to 24.1)	0.3 (0.1 to 0.6)	9.9 (2.2 to 19.6)	0.3 (0.1 to 0.5)	10.2 (2.3 to 20.1)
Haiti	Metabolic risks	2.2 (0.5 to 5.1)	53.3 (6.4 to 126)	1.4 (0.3 to 3.3)	54.7 (6.8 to 129.2)	3.7 (0.9 to 8.1)	86.7 (16 to 203.6)	2.6 (0.6 to 6.2)	89.2 (16.6 to 210.1)
Haiti	High body-mass index	0.2 (-0.1 to 0.7)	0 (-12.9 to 12)	0.1 (-0.2 to 0.4)	0.1 (-13.1 to 12.4)	0.4 (-0.1 to 1.1)	3.4 (-12.3 to 18.8)	0.2 (-0.2 to 0.7)	3.6 (-12.4 to 19.4)
Haiti	High fasting plasma glucose	2 (0.4 to 4.9)	53.9 (9.8 to 125.7)	1.3 (0.2 to 3.2)	55.3 (10 to 129.3)	3.3 (0.6 to 7.8)	84.9 (16.4 to 202.5)	2.4 (0.5 to 6.1)	87.3 (16.9 to 209.1)
Honduras	All risk factors	1.4 (0.8 to 2.3)	36 (21 to 58.3)	1.2 (0.6 to 2)	37.1 (21.7 to 60.3)	2.7 (1.4 to 4.7)	63.1 (31.5 to 112.5)	2.8 (1.3 to 5.4)	66 (33.1 to 117.8)
Honduras	Behavioral risks	0.7 (0.4 to 0.9)	21 (13.5 to 29.2)	0.6 (0.4 to 0.9)	21.6 (13.8 to 30.1)	0.9 (0.6 to 1.4)	26.3 (15.6 to 40.4)	1.1 (0.6 to 1.8)	27.4 (16.4 to 42)
Honduras	Alcohol use	0.1 (0.1 to 0.2)	4.5 (3.1 to 6.3)	0.1 (0.1 to 0.2)	4.6 (3.2 to 6.5)	0.2 (0.1 to 0.3)	6.2 (3.6 to 9.7)	0.3 (0.1 to 0.4)	6.5 (3.8 to 10.1)
Honduras	Diet high in red meat	0.1 (0 to 0.2)	4.3 (0.8 to 6.7)	0.1 (0 to 0.2)	4.4 (0.8 to 6.8)	0.2 (0 to 0.4)	6.1 (1.1 to 10.5)	0.3 (0 to 0.5)	6.3 (1.1 to 11)
Honduras	Low physical activity	0 (0 to 0.1)	1.2 (0.7 to 2.8)	0 (0 to 0.1)	1.2 (0.7 to 2.9)	0.1 (0 to 0.1)	1.7 (0.9 to 3.6)	0.1 (0 to 0.2)	1.8 (0.9 to 3.8)
Honduras	Smoking	0.2 (0.1 to 0.3)	4.9 (2.7 to 7.6)	0.2 (0.1 to 0.3)	5.1 (2.8 to 7.9)	0.2 (0.1 to 0.3)	5.2 (2.8 to 8.8)	0.2 (0.1 to 0.4)	5.4 (2.9 to 9.2)
Honduras	Secondhand smoke	0.2 (0 to 0.4)	6.7 (1.5 to 12.1)	0.2 (0 to 0.4)	6.9 (1.5 to 12.4)	0.3 (0.1 to 0.5)	7.8 (1.9 to 14.6)	0.3 (0.1 to 0.7)	8.2 (2 to 15.2)
Honduras	Metabolic risks	0.8 (0.2 to 1.6)	16.3 (2.5 to 37.7)	0.6 (0.1 to 1.3)	16.8 (2.6 to 39)	1.9 (0.6 to 3.8)	39.8 (10.2 to 84.5)	1.9 (0.5 to 4.1)	41.6 (10.7 to 88.7)
Honduras	High body-mass index	0.2 (0 to 0.5)	2.6 (-4.3 to 8.7)	0.1 (-0.1 to 0.4)	2.7 (-4.4 to 9)	0.6 (0.1 to 1.3)	9.8 (-3.6 to 26.4)	0.5 (0 to 1.3)	10.3 (-3.8 to 27.5)
Honduras	High fasting plasma glucose	0.6 (0.1 to 1.4)	14.1 (2.8 to 34.2)	0.5 (0.1 to 1.2)	14.6 (2.9 to 35.1)	1.4 (0.3 to 3.3)	31.7 (6 to 78.6)	1.4 (0.3 to 3.7)	33.1 (6.3 to 81.7)
Hungary	All risk factors	8.3 (6.5 to 10.3)	217.9 (171.8 to 263.8)	11.9 (7.6 to 16.8)	229.8 (181 to 278.1)	6.4 (4.5 to 8.9)	154.8 (109.7 to 212.2)	13.3 (8.3 to 20.2)	168.1 (120.5 to 231.5)
Hungary	Behavioral risks	6.2 (5.1 to 7.1)	180.7 (151.8 to 207.4)	9.7 (6.5 to 13.8)	190.4 (159.4 to 220.5)	4.1 (3.1 to 5.2)	111.6 (85.1 to 145.2)	9.7 (6.2 to 14.4)	121.3 (92.3 to 157)
Hungary	Alcohol use	2.7 (2.2 to 3.3)	80.9 (64.2 to 97.8)	4.4 (2.9 to 6.3)	85.3 (67.3 to 103.5)	1.6 (1.1 to 2.1)	42.8 (30.3 to 58)	3.7 (2.2 to 5.8)	46.5 (32.8 to 62.6)
Hungary	Diet high in red meat	1.1 (0.5 to 1.5)	32.3 (13.8 to 42.2)	1.7 (0.7 to 2.7)	34 (14.5 to 44.6)	0.8 (0.3 to 1.2)	21.2 (8.4 to 31.7)	1.8 (0.7 to 3)	23.1 (9 to 34.3)
Hungary	Low physical activity	0.2 (0.1 to 0.4)	5.3 (3.2 to 9.2)	0.3 (0.2 to 0.6)	5.6 (3.3 to 9.8)	0.2 (0.1 to 0.4)	4.2 (2.4 to 7.3)	0.4 (0.2 to 0.7)	4.6 (2.6 to 8)
Hungary	Smoking	1.9 (1.4 to 2.5)	59.2 (41.7 to 77.8)	3.2 (1.9 to 4.8)	62.4 (43.9 to 81.9)	1.4 (1 to 2)	40.6 (28.6 to 56.9)	3.5 (2.1 to 5.5)	44.1 (30.8 to 61.8)
Hungary	Secondhand smoke	0.7 (0.2 to 1.3)	21.5 (5 to 37)	1.2 (0.3 to 2.1)	22.7 (5.3 to 38.9)	0.5 (0.1 to 0.9)	13.1 (3.2 to 23.7)	1.1 (0.2 to 2.1)	14.2 (3.5 to 25.6)
Hungary	Metabolic risks	2.7 (1 to 5.1)	49.1 (7.1 to 101.9)	2.8 (0.5 to 5.9)	51.9 (7.5 to 107.5)	2.9 (1.1 to 5.3)	55.4 (15.7 to 108.6)	4.7 (1.2 to 9.6)	60.1 (17.3 to 117.8)
Hungary	High body-mass index	1.4 (0.3 to 2.8)	18.7 (-8.8 to 50.1)	1.1 (-0.4 to 2.9)	19.8 (-9.3 to 52.5)	1.4 (0.4 to 2.9)	23.9 (0.3 to 54.1)	1.9 (-0.1 to 4.5)	25.8 (0.2 to 58.9)
Hungary	High fasting plasma glucose	1.5 (0.3 to 3.4)	32.6 (6.1 to 74.7)	1.8 (0.3 to 4.5)	34.5 (6.5 to 79)	1.6 (0.3 to 3.8)	34.5 (6.7 to 81.9)	3 (0.6 to 7.3)	37.5 (7.3 to 89)
Iceland	All risk factors	7.5 (6 to 9.1)	196.4 (156.5 to 235.1)	19.2 (12.3 to 27.7)	215.5 (171.3 to 259.4)	4.5 (3.4 to 5.8)	108.6 (82.8 to 138.6)	16.2 (10.4 to 23.3)	124.8 (94.7 to 159.2)
Iceland	Behavioral risks	5.9 (4.9 to 6.9)	167.5 (137.8 to 197.3)	16 (10.4 to 23.2)	183.6 (151.4 to 217.4)	3.3 (2.7 to 4)	88.2 (72 to 105.4)	12.8 (8.3 to 18.3)	101.1 (81.8 to 122.5)
Iceland	Alcohol use	2.2 (1.7 to 2.8)	64.8 (48.7 to 81.2)	6.1 (3.8 to 9.2)	70.9 (53.7 to 89)	1.6 (1.2 to 2)	45.1 (34.8 to 55.6)	6.5 (4.1 to 9.4)	51.6 (39.7 to 63.6)
Iceland	Diet high in red meat	1.5 (0.8 to 2.1)	42.2 (20.4 to 59.5)	4.1 (1.9 to 6.7)	46.3 (22.2 to 65.6)	0.8 (0.4 to 1.1)	21.2 (10.6 to 30.2)	3.1 (1.4 to 5)	24.3 (12 to 34.8)
Iceland	Low physical activity	0.4 (0.1 to 0.8)	9.5 (3 to 18.5)	1 (0.3 to 2.1)	10.5 (3.4 to 20.3)	0.3 (0.1 to 0.5)	5.8 (1.8 to 10.9)	0.9 (0.3 to 1.8)	6.7 (2 to 12.7)
Iceland	Smoking	1.9 (1.3 to 2.5)	53.5 (38.1 to 70.9)	5.1 (2.9 to 8)	58.6 (41.7 to 77.4)	0.7 (0.5 to 1)	19.5 (13.4 to 26.1)	2.9 (1.7 to 4.4)	22.3 (15.7 to 30.3)
Iceland	Secondhand smoke	0.5 (0.1 to 0.9)	15.3 (3.6 to 27.3)	1.4 (0.3 to 2.7)	16.7 (3.8 to 29.7)	0.2 (0 to 0.3)	5.3 (1.2 to 9.6)	0.7 (0.2 to 1.4)	6.1 (1.4 to 10.9)
Iceland	Metabolic risks	2.1 (0.7 to 3.8)	37.8 (7.7 to 76.5)	4 (1 to 8.2)	41.9 (8.7 to 83.4)	1.6 (0.6 to 3)	26.8 (4.4 to 57.2)	4.4 (1 to 8.9)	31.2 (5.7 to 65.7)
Iceland	High body-mass index	1.2 (0.3 to 2.5)	19.1 (-4.2 to 47.1)	2.1 (-0.2 to 5)	21.2 (-4.3 to 51.6)	0.7 (0.1 to 1.6)	9.2 (-4.9 to 25.7)	1.7 (-0.3 to 4.3)	10.9 (-5.1 to 29.5)
Iceland	High fasting plasma glucose	0.9 (0.2 to 2.2)	19.9 (3.5 to 47.1)	2.1 (0.4 to 5.2)	22 (4 to 52.1)	0.9 (0.2 to 2.1)	18.8 (3.5 to 43.8)	2.9 (0.5 to 7.1)	21.7 (4.1 to 49.9)
India	All risk factors	1.3 (0.7 to 2.1)	33.1 (17.3 to 54.1)	0.9 (0.4 to 1.5)	34 (17.8 to 55.6)	2.1 (1.1 to 3.6)	51.8 (24.5 to 91.4)	1.8 (0.9 to 3.4)	53.6 (25.7 to 94.6)
India	Behavioral risks	0.7 (0.4 to 1)	19.9 (11 to 29)	0.5 (0.3 to 0.8)	20.4 (11.4 to 29.7)	0.8 (0.5 to 1.2)	22.9 (12.7 to 34)	0.8 (0.4 to 1.2)	23.7 (13.2 to 35)
India	Alcohol use	0.1 (0 to 0.1)	1.7 (1.1 to 2.4)	0 (0 to 0.1)	1.7 (1.1 to 2.5)	0.1 (0.1 to 0.2)	3.3 (2 to 4.9)	0.1 (0.1 to 0.2)	3.4 (2.1 to 5)
India	Diet high in red meat	0.1 (0 to 0.1)	1.9 (0.9 to 2.6)	0 (0 to 0.1)	1.9 (0.9 to 2.6)	0.1 (0 to 0.1)	2.3 (1.1 to 3.5)	0.1 (0 to 0.1)	2.4 (1.1 to 3.6)

Location	Risk factor	1990				2019			
		Deaths	YLLs (Years of Life Lost)	YLDs (Years Lived with Disability)	DALYs (Disability-Adjusted Life Years)	Deaths	YLLs (Years of Life Lost)	YLDs (Years Lived with Disability)	DALYs (Disability-Adjusted Life Years)
India	Low physical activity	0.1 (0.1 to 0.3)	3.4 (1.7 to 5.9)	0.1 (0 to 0.2)	3.5 (1.7 to 6.1)	0.2 (0.1 to 0.3)	3.5 (1.8 to 5.9)	0.1 (0.1 to 0.2)	3.6 (1.9 to 6.2)
India	Smoking	0.1 (0.1 to 0.1)	2.4 (1.4 to 3.7)	0.1 (0 to 0.1)	2.5 (1.4 to 3.8)	0.1 (0.1 to 0.2)	2.6 (1.5 to 4)	0.1 (0 to 0.1)	2.7 (1.5 to 4.1)
India	Secondhand smoke	0.4 (0.1 to 0.6)	10.9 (2.6 to 19.4)	0.3 (0.1 to 0.5)	11.2 (2.7 to 19.9)	0.4 (0.1 to 0.7)	11.7 (2.8 to 21.2)	0.4 (0.1 to 0.7)	12.1 (2.9 to 21.9)
India	Metabolic risks	0.6 (0.2 to 1.4)	14.3 (2.3 to 33.3)	0.4 (0.1 to 1)	14.7 (2.4 to 34.2)	1.4 (0.4 to 2.9)	30.7 (6.5 to 69.7)	1.1 (0.3 to 2.6)	31.8 (6.8 to 72)
India	High body-mass index	0.1 (0 to 0.2)	0.5 (-2.2 to 3.6)	0 (0 to 0.1)	0.5 (-2.2 to 3.7)	0.3 (0 to 0.7)	4.2 (-3.1 to 12.8)	0.2 (-0.1 to 0.5)	4.3 (-3.1 to 13.3)
India	High fasting plasma glucose	0.6 (0.1 to 1.4)	13.9 (2.5 to 32.9)	0.4 (0.1 to 0.9)	14.3 (2.5 to 33.9)	1.1 (0.2 to 2.6)	27.4 (5.1 to 64.6)	1 (0.2 to 2.4)	28.3 (5.3 to 67.2)
Indonesia	All risk factors	2.2 (1.2 to 3.5)	68.9 (37.2 to 106.4)	1.8 (0.9 to 3)	70.7 (38.1 to 109.7)	4.4 (2.4 to 6.9)	130.1 (70.1 to 202.3)	4.7 (2.4 to 7.7)	134.8 (73.4 to 209.1)
Indonesia	Behavioral risks	1.1 (0.5 to 1.7)	38.7 (16.5 to 60.9)	1 (0.4 to 1.6)	39.7 (16.9 to 62.7)	1.4 (0.7 to 2.2)	46.4 (21 to 74.5)	1.6 (0.7 to 2.8)	48.1 (21.8 to 76.7)
Indonesia	Alcohol use	0 (0 to 0.1)	1.1 (0.3 to 2.1)	0 (0 to 0.1)	1.1 (0.3 to 2.1)	0 (0 to 0.1)	1.3 (0.3 to 2.6)	0 (0 to 0.1)	1.3 (0.3 to 2.7)
Indonesia	Diet high in red meat	0.1 (0 to 0.2)	5.2 (1.4 to 7.7)	0.1 (0 to 0.2)	5.3 (1.5 to 7.9)	0.2 (0.1 to 0.3)	6.5 (1.6 to 10.4)	0.2 (0.1 to 0.4)	6.8 (1.7 to 10.7)
Indonesia	Low physical activity	0.1 (0.1 to 0.2)	3.2 (1.9 to 6.3)	0.1 (0 to 0.2)	3.3 (1.9 to 6.5)	0.1 (0.1 to 0.3)	3.8 (2 to 7.3)	0.1 (0.1 to 0.3)	4 (2.1 to 7.6)
Indonesia	Smoking	0.1 (0.1 to 0.2)	2.8 (1.4 to 4.7)	0.1 (0 to 0.1)	2.9 (1.4 to 4.8)	0.2 (0.1 to 0.3)	4.2 (2.2 to 6.7)	0.2 (0.1 to 0.3)	4.3 (2.3 to 6.9)
Indonesia	Secondhand smoke	0.7 (0.2 to 1.3)	27 (6.3 to 48.1)	0.7 (0.2 to 1.2)	27.7 (6.5 to 49.2)	0.9 (0.2 to 1.6)	31.4 (7.5 to 56.9)	1.1 (0.3 to 2.1)	32.5 (7.8 to 58.1)
Indonesia	Metabolic risks	1.2 (0.3 to 2.4)	32.3 (9.4 to 64.6)	0.9 (0.3 to 1.9)	33.2 (9.6 to 66.3)	3.3 (1.4 to 5.7)	89.9 (36.7 to 156.1)	3.3 (1.3 to 5.8)	93.2 (38.4 to 160.8)
Indonesia	High body-mass index	0.7 (0.1 to 1.6)	18 (3.3 to 44.2)	0.5 (0.1 to 1.3)	18.5 (3.4 to 45.3)	2.4 (0.7 to 4.6)	66.7 (20.9 to 127.8)	2.4 (0.8 to 4.7)	69.1 (21.6 to 132.3)
Indonesia	High fasting plasma glucose	0.5 (0.1 to 1.3)	15 (2.7 to 36.3)	0.4 (0.1 to 1)	15.4 (2.8 to 37.1)	1 (0.2 to 2.4)	27 (4.7 to 64.7)	1 (0.2 to 2.5)	27.9 (4.9 to 66.8)
Iran (Islamic Republic of)	All risk factors	1.5 (0.8 to 2.6)	36.5 (18.6 to 61.6)	1.6 (0.8 to 2.8)	38 (19.4 to 63.7)	2.3 (1.3 to 3.7)	52.1 (25.8 to 85.9)	3.4 (1.6 to 5.9)	55.4 (27.5 to 91)
Iran (Islamic Republic of)	Behavioral risks	0.8 (0.5 to 1.2)	23.6 (14.1 to 34.7)	1 (0.5 to 1.5)	24.6 (14.8 to 36.2)	0.9 (0.6 to 1.3)	26.6 (17.1 to 36.3)	1.7 (1 to 2.7)	28.4 (18.1 to 39)
Iran (Islamic Republic of)	Alcohol use	0 (0 to 0)	0.3 (0.2 to 0.5)	0 (0 to 0)	0.3 (0.2 to 0.5)	0 (0 to 0.1)	1.2 (0.9 to 1.7)	0.1 (0 to 0.1)	1.3 (0.9 to 1.8)
Iran (Islamic Republic of)	Diet high in red meat	0.2 (0 to 0.3)	6.7 (1.5 to 10.2)	0.3 (0.1 to 0.5)	6.9 (1.6 to 10.6)	0.2 (0 to 0.3)	6.3 (1.4 to 8.9)	0.4 (0.1 to 0.7)	6.7 (1.5 to 9.5)
Iran (Islamic Republic of)	Low physical activity	0.2 (0.1 to 0.4)	4.4 (1.7 to 8.5)	0.2 (0.1 to 0.4)	4.6 (1.7 to 8.8)	0.2 (0.1 to 0.4)	5.4 (2.1 to 10)	0.4 (0.1 to 0.7)	5.8 (2.3 to 10.7)
Iran (Islamic Republic of)	Smoking	0.1 (0.1 to 0.2)	3.8 (2 to 6)	0.2 (0.1 to 0.3)	3.9 (2.1 to 6.3)	0.1 (0.1 to 0.2)	3.9 (2.4 to 5.4)	0.2 (0.1 to 0.4)	4.1 (2.6 to 5.8)
Iran (Islamic Republic of)	Secondhand smoke	0.3 (0.1 to 0.5)	9.2 (2.2 to 16.2)	0.4 (0.1 to 0.7)	9.5 (2.3 to 16.9)	0.3 (0.1 to 0.6)	10.6 (2.5 to 18.6)	0.7 (0.2 to 1.3)	11.3 (2.6 to 19.7)
Iran (Islamic Republic of)	Metabolic risks	0.8 (0.2 to 1.7)	14.1 (-1.2 to 36.4)	0.7 (0 to 1.6)	14.7 (-1.1 to 37.7)	1.5 (0.5 to 2.9)	27.8 (2.1 to 61.3)	1.8 (0.1 to 4.2)	29.6 (2.2 to 65.7)
Iran (Islamic Republic of)	High body-mass index	0.3 (0 to 0.7)	2.2 (-6.8 to 11.9)	0.1 (-0.2 to 0.6)	2.3 (-7.1 to 12.3)	0.5 (0 to 1)	3.1 (-10.6 to 15.8)	0.1 (-0.8 to 1)	3.2 (-11.3 to 16.5)
Iran (Islamic Republic of)	High fasting plasma glucose	0.5 (0.1 to 1.3)	12.4 (2.1 to 30.7)	0.5 (0.1 to 1.4)	12.9 (2.2 to 32.2)	1.1 (0.2 to 2.4)	26.3 (5.2 to 58.1)	1.8 (0.3 to 4.2)	28.1 (5.5 to 61)
Iraq	All risk factors	3 (1.4 to 5.1)	76.3 (32.9 to 137.4)	2.6 (1.1 to 4.7)	78.9 (34 to 142.3)	4.4 (2.2 to 7.6)	108.9 (46.4 to 202.2)	5.3 (2.2 to 9.9)	114.2 (49.7 to 211.6)
Iraq	Behavioral risks	1.5 (0.9 to 2.3)	47.9 (28 to 72.9)	1.5 (0.8 to 2.6)	49.5 (29.2 to 75.1)	1.8 (1.1 to 2.6)	53.3 (30.3 to 82.1)	2.6 (1.4 to 4.3)	55.9 (31.8 to 85.6)
Iraq	Alcohol use	0.2 (0.1 to 0.2)	5.4 (3.3 to 8.5)	0.2 (0.1 to 0.3)	5.6 (3.4 to 8.8)	0.1 (0.1 to 0.2)	3.3 (1.8 to 5.3)	0.2 (0.1 to 0.3)	3.4 (1.9 to 5.6)
Iraq	Diet high in red meat	0.2 (0 to 0.4)	7.4 (1.5 to 12.1)	0.2 (0 to 0.4)	7.6 (1.5 to 12.5)	0.2 (0 to 0.3)	6.5 (1.5 to 10.9)	0.3 (0.1 to 0.6)	6.9 (1.6 to 11.4)
Iraq	Low physical activity	0.3 (0.1 to 0.6)	9 (3.1 to 17.3)	0.3 (0.1 to 0.6)	9.3 (3.2 to 18)	0.5 (0.2 to 0.8)	12.1 (4.5 to 22.4)	0.6 (0.2 to 1.3)	12.8 (4.8 to 23.4)
Iraq	Smoking	0.3 (0.2 to 0.5)	7.7 (4.3 to 12.5)	0.3 (0.1 to 0.4)	7.9 (4.5 to 12.9)	0.3 (0.2 to 0.5)	8.6 (5.4 to 13.4)	0.4 (0.2 to 0.7)	9.1 (5.7 to 14)
Iraq	Secondhand smoke	0.6 (0.1 to 1.1)	20.1 (4.7 to 37.6)	0.6 (0.1 to 1.2)	20.7 (4.8 to 38.5)	0.7 (0.2 to 1.4)	24.3 (6 to 46.2)	1.2 (0.3 to 2.3)	25.4 (6.3 to 48.3)
Iraq	Metabolic risks	1.6 (0.3 to 3.5)	31.7 (-7.8 to 87.2)	1.2 (-0.1 to 3)	32.9 (-7.8 to 89.9)	2.9 (0.8 to 6)	61.4 (1.9 to 147.8)	3 (0 to 7.3)	64.4 (1.9 to 155)
Iraq	High body-mass index	0.4 (-0.3 to 1.3)	-0.9 (-24.7 to 21.2)	0.1 (-0.7 to 0.8)	-0.8 (-25.2 to 21.9)	0.7 (-0.2 to 1.9)	2.9 (-26.4 to 30.4)	0.1 (-1.3 to 1.4)	3 (-27.6 to 31.8)
Iraq	High fasting plasma glucose	1.2 (0.2 to 3)	34 (6.2 to 83.6)	1.1 (0.2 to 2.9)	35.2 (6.4 to 86.3)	2.3 (0.5 to 5.4)	61.8 (12.1 to 145.1)	3.1 (0.6 to 7.6)	64.8 (12.8 to 153.5)
Ireland	All risk factors	10.9 (9.2 to 12.7)	296.7 (250.9 to 344.6)	22.5 (15.3 to 31.1)	319.2 (269.2 to 372.1)	7.2 (5.7 to 9.1)	170.7 (136.4 to 210.5)	24.5 (15.6 to 37.3)	195.2 (155.1 to 243.2)
Ireland	Behavioral risks	9.5 (8.1 to 10.8)	271.3 (232.9 to 308.4)	20.4 (13.9 to 28.5)	291.7 (249.3 to 332.7)	5.4 (4.5 to 6.5)	139.3 (116.2 to 164.7)	19.9 (12.5 to 30)	159.2 (131 to 188.8)
Ireland	Alcohol use	4.2 (3.4 to 5)	126.8 (103.1 to 151.5)	9.4 (6.3 to 13.3)	136.3 (110.5 to 162.9)	2.5 (1.9 to 3.1)	67.1 (52.4 to 82.9)	9.5 (5.8 to 15)	76.6 (59.1 to 95.3)

Location	Risk factor	1990				2019			
		Deaths	YLLs (Years of Life Lost)	YLDs (Years Lived with Disability)	DALYs (Disability-Adjusted Life Years)	Deaths	YLLs (Years of Life Lost)	YLDs (Years Lived with Disability)	DALYs (Disability-Adjusted Life Years)
Ireland	Diet high in red meat	1.9 (1 to 2.6)	53.9 (26.6 to 73.9)	4.1 (1.9 to 6.4)	58 (28.8 to 80.1)	1.3 (0.6 to 1.8)	32 (15.7 to 47.5)	4.6 (2.1 to 8.2)	36.6 (18.1 to 54.6)
Ireland	Low physical activity	0.8 (0.3 to 1.4)	19.4 (6.2 to 34.5)	1.5 (0.5 to 2.9)	20.9 (6.7 to 37)	0.6 (0.2 to 1)	12.2 (4 to 21.1)	1.8 (0.6 to 3.4)	13.9 (4.6 to 24.4)
Ireland	Smoking	3.1 (2.2 to 4)	85.1 (61.5 to 109)	6.4 (3.9 to 9.5)	91.6 (65.9 to 117.4)	1.4 (1 to 1.9)	36.4 (26.2 to 47.4)	5.2 (3 to 8.3)	41.6 (29.8 to 54.8)
Ireland	Secondhand smoke	0.7 (0.2 to 1.2)	21 (5 to 36.1)	1.5 (0.3 to 2.8)	22.5 (5.2 to 39.2)	0.3 (0.1 to 0.5)	7.9 (1.9 to 13.7)	1.1 (0.2 to 2.1)	9 (2.2 to 16)
Ireland	Metabolic risks	2 (0.7 to 3.8)	35.6 (3 to 75.7)	2.9 (0.3 to 6)	38.5 (3.5 to 81.6)	2.4 (1 to 4.5)	42.9 (12.6 to 84.3)	6.3 (1.6 to 12.7)	49.1 (14.2 to 95.1)
Ireland	High body-mass index	1.2 (0.2 to 2.6)	18.4 (-7.9 to 48)	1.6 (-0.5 to 4)	20 (-8.5 to 51.5)	1.1 (0.3 to 2.3)	17.2 (-1.6 to 40.2)	2.5 (-0.2 to 6)	19.7 (-1.9 to 46.6)
Ireland	High fasting plasma glucose	0.8 (0.1 to 1.9)	18 (3.2 to 42.2)	1.4 (0.2 to 3.6)	19.4 (3.5 to 45.8)	1.4 (0.3 to 3.2)	27.5 (5.3 to 63.5)	4 (0.7 to 10.2)	31.5 (6.1 to 72.8)
Israel	All risk factors	7.3 (5.2 to 9.7)	182.3 (131.1 to 234)	12.2 (7.6 to 18.1)	194.5 (139.7 to 249.2)	5.5 (3.8 to 7.7)	123 (87 to 165.8)	14 (8.5 to 22.3)	137.1 (96.5 to 184.5)
Israel	Behavioral risks	4.8 (3.8 to 5.8)	140.4 (109.4 to 168.9)	9 (5.7 to 13)	149.4 (115.7 to 180)	3.1 (2.4 to 3.7)	82.1 (64.2 to 98.7)	9.2 (5.5 to 13.7)	91.3 (71 to 110.3)
Israel	Alcohol use	0.9 (0.6 to 1.3)	29.8 (19.6 to 40.8)	1.9 (1.1 to 3)	31.7 (20.7 to 43.3)	0.8 (0.6 to 1.1)	24.2 (16.8 to 32.4)	2.7 (1.5 to 4.3)	26.9 (18.6 to 36)
Israel	Diet high in red meat	0.9 (0.2 to 1.2)	25 (7.4 to 32.7)	1.6 (0.5 to 2.5)	26.6 (7.9 to 35)	0.8 (0.3 to 1.1)	20.3 (7.9 to 27.4)	2.3 (0.8 to 3.8)	22.6 (8.7 to 30.5)
Israel	Low physical activity	0.5 (0.2 to 1)	11.7 (3.8 to 23.3)	0.8 (0.3 to 1.7)	12.5 (4.1 to 24.8)	0.4 (0.1 to 0.8)	8.3 (2.7 to 15.6)	1 (0.3 to 2)	9.3 (3 to 17.6)
Israel	Smoking	2.2 (1.5 to 2.9)	63.6 (44.1 to 84.9)	4.1 (2.4 to 6.3)	67.7 (47.5 to 90.3)	1 (0.7 to 1.3)	25.1 (18 to 33.2)	2.9 (1.6 to 4.6)	27.9 (19.9 to 37.1)
Israel	Secondhand smoke	0.6 (0.1 to 1)	19.6 (4.5 to 33.9)	1.2 (0.2 to 2.3)	20.8 (4.9 to 36.1)	0.3 (0.1 to 0.6)	9.6 (2.3 to 17)	1.1 (0.2 to 2.1)	10.7 (2.6 to 18.8)
Israel	Metabolic risks	2.9 (1 to 5.5)	49.5 (8.4 to 103)	3.7 (0.8 to 7.7)	53.2 (9.3 to 110.5)	2.8 (1.1 to 5.2)	48 (12.6 to 93.4)	5.6 (1.5 to 11.7)	53.7 (14 to 104.5)
Israel	High body-mass index	1.3 (0.2 to 2.8)	17.1 (-8.6 to 45.8)	1.4 (-0.4 to 3.4)	18.5 (-8.9 to 48.9)	1.2 (0.3 to 2.5)	16.6 (-2.8 to 39.8)	2 (-0.2 to 4.8)	18.6 (-3.1 to 44.6)
Israel	High fasting plasma glucose	1.7 (0.3 to 3.8)	34.4 (6.4 to 77.9)	2.5 (0.5 to 6)	36.8 (6.9 to 84.2)	1.8 (0.3 to 3.9)	33.8 (6.6 to 75.3)	4 (0.7 to 9.7)	37.7 (7.4 to 83.9)
Italy	All risk factors	8.6 (6.9 to 10.5)	230.8 (189.5 to 274.1)	21.4 (14.1 to 30.1)	252.2 (206.1 to 301.7)	5.9 (4.5 to 7.7)	140.8 (108.2 to 177.1)	21.1 (13.1 to 32)	161.8 (124.8 to 204.3)
Italy	Behavioral risks	6.7 (5.7 to 7.7)	194.3 (165.4 to 223.8)	17.9 (12 to 25.2)	212.1 (179.5 to 243.3)	4 (3.3 to 4.7)	106.7 (88.6 to 124)	15.9 (9.9 to 23.8)	122.7 (101.8 to 143.4)
Italy	Alcohol use	3.4 (2.7 to 4.2)	100.1 (79.3 to 123.8)	9.2 (5.9 to 13.5)	109.3 (86.5 to 135.2)	1.7 (1.3 to 2.2)	47.3 (37.1 to 59.6)	7.1 (4.3 to 10.9)	54.4 (42.1 to 68.8)
Italy	Diet high in red meat	1.3 (0.7 to 1.8)	38.5 (19.8 to 51.8)	3.6 (1.7 to 5.6)	42.1 (21.3 to 56.5)	0.9 (0.4 to 1.2)	23.2 (11.5 to 31.5)	3.5 (1.5 to 5.7)	26.7 (13.3 to 36.4)
Italy	Low physical activity	0.5 (0.2 to 0.9)	12.3 (3.8 to 23.2)	1.2 (0.4 to 2.5)	13.5 (4.3 to 25.1)	0.4 (0.1 to 0.7)	8 (2.6 to 14.9)	1.2 (0.4 to 2.6)	9.2 (3 to 17)
Italy	Smoking	1.6 (1.1 to 2)	46.4 (33.1 to 60.1)	4.3 (2.5 to 6.4)	50.7 (36 to 66.3)	1.1 (0.8 to 1.4)	29.2 (21.3 to 37.3)	4.4 (2.5 to 6.7)	33.5 (24.4 to 43.4)
Italy	Secondhand smoke	0.6 (0.1 to 1)	18.7 (4.3 to 32.8)	1.7 (0.4 to 3.1)	20.4 (4.7 to 35.6)	0.3 (0.1 to 0.5)	9.5 (2.3 to 16.5)	1.4 (0.3 to 2.7)	10.9 (2.6 to 18.8)
Italy	Metabolic risks	2.5 (0.9 to 4.7)	48.9 (12.8 to 97.8)	4.7 (1.3 to 9.5)	53.6 (14.4 to 105.5)	2.4 (0.9 to 4.4)	43.6 (13.4 to 85.2)	6.6 (1.8 to 13.7)	50.2 (15.5 to 97.9)
Italy	High body-mass index	1 (0.2 to 2.1)	15.6 (-1.7 to 37.9)	1.5 (-0.1 to 3.7)	17.2 (-1.8 to 41.6)	0.9 (0.2 to 1.8)	13.9 (0.2 to 31.5)	2.1 (0 to 4.8)	16 (0.2 to 35.6)
Italy	High fasting plasma glucose	1.6 (0.3 to 3.6)	35 (6.6 to 78.2)	3.4 (0.6 to 8)	38.4 (7.2 to 86)	1.6 (0.3 to 3.5)	31.6 (6.2 to 69.5)	4.8 (0.9 to 11.7)	36.3 (7.1 to 80)
Jamaica	All risk factors	4.3 (2.8 to 6.2)	106.7 (68.6 to 153.4)	5.2 (3 to 8.3)	111.9 (72.1 to 161.6)	7 (4.1 to 11.2)	169.4 (92.6 to 278)	9.9 (5 to 16.9)	179.3 (98.3 to 292.5)
Jamaica	Behavioral risks	2.3 (1.8 to 2.9)	68.6 (52.5 to 84.3)	3.1 (2.1 to 4.4)	71.7 (54.5 to 88.7)	3.4 (2.4 to 4.5)	101.7 (70.9 to 137.9)	5.6 (3.4 to 8.4)	107.3 (74.8 to 144.5)
Jamaica	Alcohol use	0.7 (0.5 to 1)	24.3 (17.9 to 31.6)	1.1 (0.7 to 1.6)	25.4 (18.7 to 33)	1.2 (0.8 to 1.7)	41.9 (27.7 to 59)	2.2 (1.3 to 3.5)	44.2 (29.4 to 62.1)
Jamaica	Diet high in red meat	0.4 (0.1 to 0.5)	10.9 (2.1 to 14.8)	0.5 (0.1 to 0.8)	11.4 (2.2 to 15.4)	0.5 (0.1 to 0.7)	14.6 (2.7 to 23.1)	0.8 (0.1 to 1.4)	15.4 (2.9 to 24.5)
Jamaica	Low physical activity	0.4 (0.1 to 0.7)	9.3 (3 to 17.3)	0.5 (0.1 to 0.9)	9.7 (3.2 to 18.2)	0.6 (0.2 to 1.2)	15.7 (4.9 to 30)	0.9 (0.3 to 1.9)	16.6 (5.2 to 31.9)
Jamaica	Smoking	0.6 (0.4 to 0.8)	15.5 (10.1 to 21.9)	0.7 (0.4 to 1.1)	16.2 (10.5 to 22.9)	0.7 (0.4 to 1)	18.1 (11.5 to 27.1)	1 (0.6 to 1.7)	19.1 (12.1 to 28.7)
Jamaica	Secondhand smoke	0.4 (0.1 to 0.7)	11.9 (2.8 to 20.6)	0.5 (0.1 to 1)	12.5 (2.9 to 21.5)	0.5 (0.1 to 0.9)	16.1 (3.8 to 30.1)	0.9 (0.2 to 1.7)	17 (4 to 31.7)
Jamaica	Metabolic risks	2.2 (0.7 to 4.3)	43.3 (7 to 92.4)	2.3 (0.5 to 5)	45.7 (7.6 to 97.6)	4.1 (1.1 to 8.5)	76.5 (-1.1 to 184.4)	4.9 (0.4 to 11.6)	81.4 (-0.7 to 194.4)
Jamaica	High body-mass index	0.7 (0 to 1.7)	9.1 (-9.5 to 29.8)	0.6 (-0.3 to 1.8)	9.7 (-10 to 30.9)	1.1 (-0.3 to 2.8)	2.6 (-39.9 to 38.4)	0.6 (-1.6 to 2.7)	3.1 (-41.8 to 40.9)
Jamaica	High fasting plasma glucose	1.6 (0.3 to 3.4)	36.1 (6.8 to 80.5)	1.8 (0.3 to 4.4)	37.9 (7.2 to 83.9)	3.2 (0.7 to 7.3)	78.5 (15.6 to 180.4)	4.6 (0.9 to 11.2)	83 (16.5 to 189.6)
Japan	All risk factors	2.2 (1.7 to 2.6)	66.4 (53.8 to 78.2)	7.2 (4.6 to 10.2)	73.5 (59.7 to 86.6)	2.4 (1.9 to 3.1)	70.1 (55.9 to 85.6)	11.3 (7 to 17.1)	81.4 (64.2 to 99.6)
Japan	Behavioral risks	1.8 (1.5 to 2.1)	59.8 (50.2 to 69.5)	6.3 (4.2 to 9.1)	66.1 (55.5 to 76.8)	1.9 (1.6 to 2.3)	59.6 (49.7 to 70.1)	9.5 (5.9 to 14.3)	69.1 (57 to 81.8)

Location	Risk factor	1990				2019			
		Deaths	YLLs (Years of Life Lost)	YLDs (Years Lived with Disability)	DALYs (Disability-Adjusted Life Years)	Deaths	YLLs (Years of Life Lost)	YLDs (Years Lived with Disability)	DALYs (Disability-Adjusted Life Years)
Japan	Alcohol use	1.1 (0.9 to 1.3)	36.8 (29.9 to 43.6)	3.8 (2.4 to 5.6)	40.7 (33 to 48.4)	1.1 (0.9 to 1.4)	36.5 (28.5 to 44.5)	5.8 (3.6 to 8.9)	42.3 (32.9 to 52.2)
Japan	Diet high in red meat	0.2 (0.1 to 0.3)	7 (2 to 9.1)	0.7 (0.2 to 1.2)	7.7 (2.3 to 10.1)	0.3 (0.1 to 0.3)	7.8 (2.4 to 10.5)	1.2 (0.4 to 2.1)	9 (2.9 to 12.3)
Japan	Low physical activity	0.1 (0 to 0.2)	2.5 (1 to 5.3)	0.3 (0.1 to 0.7)	2.8 (1.1 to 6)	0.1 (0 to 0.2)	2.9 (1.1 to 6.1)	0.5 (0.2 to 1.2)	3.4 (1.3 to 7.4)
Japan	Smoking	0.3 (0.2 to 0.4)	9.8 (6.8 to 13)	1.1 (0.6 to 1.6)	10.9 (7.5 to 14.4)	0.3 (0.2 to 0.4)	9.8 (6.8 to 13)	1.6 (0.9 to 2.5)	11.4 (7.9 to 15.2)
Japan	Secondhand smoke	0.2 (0.1 to 0.4)	8.3 (1.9 to 14.2)	0.8 (0.2 to 1.6)	9.1 (2.1 to 15.8)	0.2 (0.1 to 0.4)	6.9 (1.7 to 11.9)	1.1 (0.2 to 2.1)	8 (1.9 to 13.6)
Japan	Metabolic risks	0.4 (0.1 to 0.9)	8.2 (0.6 to 19.1)	1.1 (0.2 to 2.4)	9.2 (0.7 to 21.1)	0.6 (0.2 to 1.2)	12.9 (2.9 to 27.8)	2.2 (0.5 to 5)	15.1 (3.5 to 32.1)
Japan	High body-mass index	0.1 (0 to 0.3)	1.1 (-2.8 to 4.9)	0.2 (-0.2 to 0.6)	1.3 (-3 to 5.5)	0.2 (0 to 0.5)	3.3 (-0.9 to 9.1)	0.6 (-0.1 to 1.6)	3.8 (-1 to 10.5)
Japan	High fasting plasma glucose	0.3 (0.1 to 0.7)	7.2 (1.3 to 16.8)	0.9 (0.2 to 2.2)	8.1 (1.5 to 19.1)	0.4 (0.1 to 1)	9.8 (1.8 to 22.9)	1.7 (0.3 to 4.3)	11.5 (2.1 to 27.1)
Jordan	All risk factors	4.1 (2.3 to 6.7)	100.1 (51.6 to 163.3)	3.7 (1.8 to 6.5)	103.8 (53.4 to 170.2)	4.8 (2.7 to 7.5)	103.2 (54.6 to 167.7)	6.5 (3 to 11.4)	109.7 (57.5 to 177.8)
Jordan	Behavioral risks	2.1 (1.3 to 3)	62.4 (37.8 to 89.6)	2.2 (1.2 to 3.5)	64.7 (39.2 to 92.6)	2 (1.3 to 2.8)	53.3 (33.3 to 76.4)	3.4 (1.9 to 5.3)	56.7 (35.4 to 81.9)
Jordan	Alcohol use	0.1 (0 to 0.1)	2.5 (1.4 to 4)	0.1 (0 to 0.2)	2.6 (1.4 to 4.2)	0.1 (0 to 0.1)	2.7 (1.4 to 4.3)	0.2 (0.1 to 0.3)	2.8 (1.5 to 4.6)
Jordan	Diet high in red meat	0.4 (0.1 to 0.6)	12.8 (2.5 to 19.5)	0.4 (0.1 to 0.8)	13.2 (2.6 to 20.2)	0.4 (0.1 to 0.6)	10.7 (2.2 to 16.9)	0.7 (0.1 to 1.2)	11.4 (2.3 to 18)
Jordan	Low physical activity	0.3 (0.1 to 0.6)	7.5 (3.2 to 14.3)	0.3 (0.1 to 0.6)	7.8 (3.4 to 15)	0.4 (0.2 to 0.7)	8.6 (3.3 to 16.1)	0.6 (0.2 to 1.1)	9.2 (3.6 to 17.2)
Jordan	Smoking	0.6 (0.3 to 0.8)	15.8 (9.2 to 24.4)	0.6 (0.3 to 0.9)	16.3 (9.6 to 25.2)	0.5 (0.3 to 0.7)	12.6 (7.4 to 18.5)	0.8 (0.4 to 1.2)	13.3 (7.9 to 19.6)
Jordan	Secondhand smoke	0.8 (0.2 to 1.4)	26.2 (6.2 to 46.2)	0.9 (0.2 to 1.7)	27.1 (6.3 to 47.9)	0.7 (0.2 to 1.2)	20.8 (4.6 to 37.6)	1.3 (0.3 to 2.5)	22.1 (5 to 39.9)
Jordan	Metabolic risks	2.3 (0.6 to 4.6)	42.4 (-1.1 to 99.4)	1.7 (0.1 to 3.9)	44.1 (-1.1 to 103.3)	3.1 (1.1 to 5.8)	56 (8 to 116.3)	3.5 (0.4 to 7.5)	59.5 (8.4 to 122.7)
Jordan	High body-mass index	0.9 (-0.1 to 2.1)	8.5 (-19.1 to 38.8)	0.4 (-0.6 to 1.5)	8.9 (-19.8 to 40.3)	1.4 (0.1 to 2.8)	16.3 (-15.3 to 46.2)	0.9 (-1.1 to 2.9)	17.2 (-16.2 to 49.2)
Jordan	High fasting plasma glucose	1.5 (0.3 to 3.5)	36.5 (6.6 to 85.9)	1.4 (0.3 to 3.4)	37.9 (6.9 to 89.4)	2 (0.4 to 4.4)	44 (8.7 to 99.7)	2.9 (0.5 to 6.7)	46.8 (9.2 to 105.7)
Kazakhstan	All risk factors	4.9 (3.5 to 6.3)	139.6 (100 to 179.7)	5.6 (3.5 to 8.1)	145.2 (103.9 to 185.8)	4.8 (3.1 to 6.8)	123.4 (80.4 to 175.5)	7.1 (4.2 to 10.9)	130.6 (85.5 to 184.9)
Kazakhstan	Behavioral risks	3.4 (2.6 to 4.2)	110.1 (85.8 to 135.5)	4.3 (2.7 to 6.2)	114.4 (89.5 to 140.3)	2.7 (2 to 3.4)	79.9 (59.1 to 101.8)	4.6 (2.9 to 6.8)	84.4 (62.5 to 107.9)
Kazakhstan	Alcohol use	1.6 (1.1 to 2.1)	53.9 (38.9 to 71.2)	2.1 (1.2 to 3.2)	56 (40.3 to 74.3)	1.1 (0.8 to 1.4)	34.4 (24.3 to 46.7)	2 (1.2 to 3.1)	36.4 (25.3 to 49.5)
Kazakhstan	Diet high in red meat	1 (0.5 to 1.4)	32.1 (15.6 to 44.1)	1.3 (0.6 to 2)	33.3 (16.4 to 45.9)	0.9 (0.4 to 1.3)	25.7 (12.8 to 37.5)	1.5 (0.7 to 2.4)	27.2 (13.5 to 39.6)
Kazakhstan	Low physical activity	0.2 (0.1 to 0.3)	4 (2.4 to 7)	0.2 (0.1 to 0.3)	4.2 (2.5 to 7.3)	0.2 (0.1 to 0.3)	3.9 (2.2 to 6.7)	0.2 (0.1 to 0.4)	4.1 (2.3 to 7.1)
Kazakhstan	Smoking	0.3 (0.1 to 0.4)	8.1 (4.1 to 13.3)	0.3 (0.1 to 0.5)	8.4 (4.3 to 13.8)	0.2 (0.1 to 0.4)	7 (3.6 to 10.7)	0.4 (0.2 to 0.7)	7.4 (3.8 to 11.4)
Kazakhstan	Secondhand smoke	0.6 (0.2 to 1.1)	20.4 (5 to 35.5)	0.8 (0.2 to 1.4)	21.1 (5.2 to 36.8)	0.5 (0.1 to 0.8)	14.5 (3.5 to 25.3)	0.8 (0.2 to 1.5)	15.3 (3.6 to 26.6)
Kazakhstan	Metabolic risks	1.8 (0.6 to 3.3)	35.2 (3 to 75.2)	1.6 (0.2 to 3.4)	36.7 (3.3 to 77.9)	2.5 (0.9 to 4.6)	51.6 (10.7 to 103.7)	3 (0.7 to 6.2)	54.6 (11.3 to 110.9)
Kazakhstan	High body-mass index	0.8 (0 to 1.8)	10.8 (-12.3 to 33.7)	0.6 (-0.3 to 1.5)	11.4 (-12.8 to 35.4)	1.1 (0.2 to 2.2)	16.9 (-5 to 41.7)	1 (-0.3 to 2.5)	17.9 (-5.2 to 43.5)
Kazakhstan	High fasting plasma glucose	1 (0.2 to 2.3)	25.9 (4.8 to 59.2)	1.1 (0.2 to 2.6)	26.9 (5 to 61.4)	1.5 (0.3 to 3.5)	37.6 (7.2 to 85.8)	2.2 (0.4 to 5.4)	39.9 (7.7 to 90.8)
Kenya	All risk factors	1.4 (0.8 to 2.4)	35.8 (19.8 to 61)	1 (0.5 to 1.7)	36.8 (20.4 to 62.7)	2.3 (1.3 to 3.7)	54.5 (30.8 to 87.5)	1.6 (0.9 to 2.5)	56 (31.7 to 89.2)
Kenya	Behavioral risks	1 (0.6 to 1.6)	27.9 (16.4 to 46.3)	0.7 (0.4 to 1.2)	28.7 (16.9 to 47.4)	1.3 (0.9 to 1.9)	37.2 (23.7 to 54.9)	1.1 (0.6 to 1.7)	38.2 (24.2 to 56.3)
Kenya	Alcohol use	0.4 (0.3 to 0.7)	12.9 (7.5 to 21.4)	0.3 (0.2 to 0.6)	13.3 (7.8 to 21.9)	0.6 (0.4 to 0.9)	16 (10.1 to 24.9)	0.5 (0.3 to 0.7)	16.5 (10.5 to 25.5)
Kenya	Diet high in red meat	0.2 (0 to 0.4)	6.3 (1.4 to 11.4)	0.2 (0 to 0.3)	6.5 (1.5 to 11.7)	0.3 (0.1 to 0.5)	9.7 (2.4 to 15.6)	0.3 (0.1 to 0.5)	10 (2.4 to 16.1)
Kenya	Low physical activity	0 (0 to 0.1)	1.2 (0.6 to 3)	0 (0 to 0.1)	1.2 (0.7 to 3)	0.1 (0 to 0.2)	1.8 (1.1 to 4.5)	0.1 (0 to 0.1)	1.8 (1.1 to 4.6)
Kenya	Smoking	0.1 (0 to 0.2)	1.8 (0.8 to 3.3)	0.1 (0 to 0.1)	1.8 (0.8 to 3.3)	0.1 (0.1 to 0.2)	2.8 (1.4 to 4.9)	0.1 (0 to 0.2)	2.9 (1.4 to 5.1)
Kenya	Secondhand smoke	0.2 (0 to 0.4)	6.7 (1.4 to 14.2)	0.2 (0 to 0.4)	6.8 (1.5 to 14.6)	0.3 (0.1 to 0.5)	7.9 (1.8 to 15.4)	0.2 (0 to 0.4)	8.1 (1.8 to 15.9)
Kenya	Metabolic risks	0.5 (0.1 to 1.1)	8.7 (-0.1 to 22)	0.3 (0 to 0.6)	9 (-0.1 to 22.7)	1.1 (0.3 to 2.2)	18.9 (-0.1 to 44.9)	0.6 (0 to 1.3)	19.5 (0 to 46.2)
Kenya	High body-mass index	0.2 (0 to 0.5)	1.4 (-4 to 7.2)	0.1 (-0.1 to 0.2)	1.4 (-4 to 7.4)	0.5 (0 to 1.1)	4.9 (-8.3 to 18.6)	0.2 (-0.2 to 0.6)	5 (-8.6 to 19.1)
Kenya	High fasting plasma glucose	0.3 (0.1 to 0.9)	7.5 (1.2 to 19.6)	0.2 (0 to 0.6)	7.7 (1.2 to 20.2)	0.6 (0.1 to 1.6)	14.6 (2.6 to 36.6)	0.4 (0.1 to 1.1)	15.1 (2.7 to 37.7)
Kiribati	All risk factors	9.8 (5.8 to 14.4)	277.5 (168.9 to 410.6)	6.6 (3.7 to 10.3)	284.1 (172.7 to 420.7)	12.7 (7.5 to 19.8)	341.1 (201.3 to 540)	8.9 (4.9 to 14.8)	350 (206.9 to 552.2)
Kiribati	Behavioral risks	4.1 (2.7 to 5.6)	120.6 (76.8 to 167.5)	2.8 (1.6 to 4.3)	123.5 (78.7 to 171.4)	5.1 (3.2 to 7.7)	139.1 (85.2 to 215.2)	3.6 (2.1 to 5.8)	142.7 (87.6 to 219.9)

Location	Risk factor	1990				2019			
		Deaths	YLLs (Years of Life Lost)	YLDs (Years Lived with Disability)	DALYs (Disability-Adjusted Life Years)	Deaths	YLLs (Years of Life Lost)	YLDs (Years Lived with Disability)	DALYs (Disability-Adjusted Life Years)
Kiribati	Alcohol use	0.1 (0 to 0.2)	3.4 (0.9 to 6.8)	0.1 (0 to 0.1)	3.5 (0.9 to 6.9)	0.1 (0 to 0.2)	3.2 (0.7 to 6.7)	0.1 (0 to 0.2)	3.3 (0.7 to 6.9)
Kiribati	Diet high in red meat	0.5 (0.1 to 0.8)	17 (3.3 to 25.7)	0.4 (0.1 to 0.6)	17.4 (3.4 to 26.3)	0.6 (0.1 to 1)	18.2 (3.4 to 29.9)	0.5 (0.1 to 0.8)	18.7 (3.5 to 30.7)
Kiribati	Low physical activity	0.4 (0.2 to 0.9)	11.2 (4.2 to 23.2)	0.3 (0.1 to 0.6)	11.5 (4.3 to 23.8)	0.6 (0.2 to 1.1)	13.5 (4.9 to 27.8)	0.4 (0.1 to 0.8)	13.9 (5.1 to 28.6)
Kiribati	Smoking	2 (1.2 to 3)	54.9 (30.8 to 81.9)	1.4 (0.7 to 2.1)	56.3 (31.5 to 83.9)	2.7 (1.5 to 4.2)	66.6 (35.9 to 110)	1.8 (0.9 to 3)	68.4 (36.9 to 112.6)
Kiribati	Secondhand smoke	1.2 (0.3 to 2.2)	39.8 (9.6 to 72.2)	0.9 (0.2 to 1.7)	40.7 (9.8 to 73.8)	1.4 (0.3 to 2.7)	44.4 (11 to 85)	1.1 (0.3 to 2.2)	45.5 (11.3 to 87.1)
Kiribati	Metabolic risks	6.6 (2.7 to 11.2)	183.9 (76.2 to 313.6)	4.5 (1.8 to 7.9)	188.4 (78.2 to 321.1)	9.1 (4 to 16.1)	238.8 (104.4 to 427.3)	6.3 (2.5 to 11.8)	245.1 (107.7 to 436.9)
Kiribati	High body-mass index	4.7 (1.6 to 8.8)	129.8 (44.8 to 242.3)	3.2 (1 to 6)	133 (46.1 to 247.4)	5.8 (1.8 to 11.2)	151.4 (47.7 to 296)	4 (1.3 to 7.9)	155.4 (48.9 to 303.3)
Kiribati	High fasting plasma glucose	2.3 (0.4 to 5.6)	65.5 (12.4 to 153.6)	1.6 (0.3 to 3.9)	67.1 (12.7 to 157.8)	4.1 (0.8 to 9.7)	107.1 (21.9 to 258.7)	2.8 (0.5 to 6.9)	109.9 (22.4 to 265.3)
Kuwait	All risk factors	4.2 (2.5 to 6.3)	98.4 (55.8 to 150.3)	5.4 (2.8 to 8.9)	103.8 (58.6 to 158.3)	3.9 (2.2 to 6)	81.6 (44.1 to 131)	7.2 (3.4 to 12.1)	88.8 (48.2 to 141.5)
Kuwait	Behavioral risks	1.9 (1.3 to 2.5)	53.8 (35.7 to 71)	3 (1.7 to 4.4)	56.7 (37.6 to 75)	1.4 (0.9 to 2.1)	35.2 (21.8 to 51.9)	3.1 (1.8 to 4.9)	38.4 (23.9 to 56.1)
Kuwait	Alcohol use	0 (0 to 0)	0 (0 to 0)	0 (0 to 0)	0 (0 to 0)	0 (0 to 0)	0.2 (0 to 0.5)	0 (0 to 0)	0.3 (0 to 0.5)
Kuwait	Diet high in red meat	0.7 (0.3 to 0.9)	20.2 (7.8 to 27.3)	1.1 (0.4 to 1.7)	21.3 (8.3 to 29)	0.5 (0.2 to 0.7)	12.6 (4.6 to 19.9)	1.1 (0.4 to 1.9)	13.7 (5.2 to 21.4)
Kuwait	Low physical activity	0.5 (0.2 to 0.8)	11.9 (4.5 to 20.8)	0.7 (0.2 to 1.3)	12.6 (4.8 to 22.1)	0.4 (0.2 to 0.7)	9.3 (3.3 to 16.5)	0.9 (0.3 to 1.6)	10.2 (3.6 to 18.1)
Kuwait	Smoking	0.2 (0.1 to 0.4)	5.9 (3.5 to 9)	0.3 (0.2 to 0.6)	6.3 (3.7 to 9.5)	0.1 (0.1 to 0.2)	3.2 (1.9 to 4.8)	0.3 (0.2 to 0.5)	3.5 (2.1 to 5.2)
Kuwait	Secondhand smoke	0.6 (0.1 to 1)	17.8 (4.2 to 30.8)	1 (0.2 to 1.8)	18.8 (4.4 to 32.5)	0.4 (0.1 to 0.7)	11.3 (2.6 to 20.4)	1 (0.2 to 1.9)	12.3 (2.8 to 22.1)
Kuwait	Metabolic risks	2.6 (0.9 to 4.7)	50.4 (7.7 to 102.3)	2.8 (0.5 to 5.8)	53.2 (8 to 107.9)	2.8 (1.1 to 5)	52.2 (14 to 100.8)	4.5 (1.1 to 9.2)	56.7 (15.3 to 109)
Kuwait	High body-mass index	1.1 (0.1 to 2.2)	15.3 (-10 to 42.7)	0.8 (-0.6 to 2.2)	16.1 (-10.6 to 44.3)	1.2 (0.3 to 2.4)	19 (-3.9 to 42.4)	1.5 (-0.4 to 3.6)	20.5 (-4.3 to 46.1)
Kuwait	High fasting plasma glucose	1.6 (0.3 to 3.6)	38.3 (7.7 to 85.6)	2.2 (0.4 to 5.1)	40.5 (8.2 to 90.3)	1.7 (0.4 to 3.8)	37.4 (7.7 to 83.7)	3.4 (0.7 to 7.9)	40.8 (8.3 to 91.2)
Kyrgyzstan	All risk factors	3.2 (2.1 to 4.3)	91 (61.4 to 121.5)	3.3 (1.9 to 4.8)	94.3 (64 to 126.2)	2.2 (1.4 to 3)	55.3 (35.8 to 78.7)	2.7 (1.6 to 4.2)	58 (37.4 to 82.5)
Kyrgyzstan	Behavioral risks	2.2 (1.6 to 2.8)	73.1 (51.7 to 93.3)	2.5 (1.6 to 3.6)	75.7 (53.2 to 96.6)	1.3 (0.9 to 1.7)	39 (27 to 50.9)	1.9 (1.1 to 2.8)	40.9 (28.5 to 53.5)
Kyrgyzstan	Alcohol use	0.5 (0.4 to 0.7)	18.5 (13.6 to 24.4)	0.6 (0.4 to 0.9)	19.1 (14.1 to 25.2)	0.3 (0.2 to 0.5)	12.1 (8.4 to 16.7)	0.6 (0.3 to 0.9)	12.6 (8.7 to 17.5)
Kyrgyzstan	Diet high in red meat	0.9 (0.5 to 1.2)	29.6 (14.5 to 40.2)	1 (0.5 to 1.6)	30.6 (14.9 to 41.5)	0.4 (0.2 to 0.6)	12.5 (5.1 to 18)	0.6 (0.2 to 1)	13.1 (5.4 to 18.9)
Kyrgyzstan	Low physical activity	0.1 (0.1 to 0.2)	3 (1.9 to 5.7)	0.1 (0.1 to 0.2)	3.1 (2 to 5.9)	0.1 (0.1 to 0.1)	1.8 (1.1 to 3.4)	0.1 (0.1 to 0.2)	1.9 (1.2 to 3.6)
Kyrgyzstan	Smoking	0.2 (0.1 to 0.3)	5.9 (3.7 to 8.6)	0.2 (0.1 to 0.3)	6.1 (3.8 to 8.9)	0.1 (0.1 to 0.2)	3.9 (2.4 to 5.4)	0.2 (0.1 to 0.3)	4 (2.5 to 5.7)
Kyrgyzstan	Secondhand smoke	0.6 (0.1 to 1.1)	19.9 (4.8 to 35.1)	0.7 (0.2 to 1.3)	20.6 (4.9 to 36.1)	0.4 (0.1 to 0.6)	10.8 (2.5 to 18.9)	0.5 (0.1 to 1)	11.3 (2.6 to 19.7)
Kyrgyzstan	Metabolic risks	1.1 (0.3 to 2.2)	19.8 (-3.2 to 48.2)	0.8 (0 to 1.9)	20.7 (-3.3 to 49.9)	1 (0.3 to 1.8)	18.3 (2.2 to 39.8)	0.9 (0.1 to 2)	19.2 (2.3 to 41.6)
Kyrgyzstan	High body-mass index	0.5 (0 to 1.3)	5.5 (-11.4 to 23.1)	0.3 (-0.3 to 1)	5.8 (-11.7 to 23.9)	0.5 (0 to 1)	6.2 (-5.3 to 19)	0.3 (-0.2 to 1)	6.6 (-5.5 to 19.9)
Kyrgyzstan	High fasting plasma glucose	0.6 (0.1 to 1.3)	14.9 (2.7 to 34.9)	0.6 (0.1 to 1.4)	15.5 (2.8 to 36.1)	0.5 (0.1 to 1.3)	12.7 (2.3 to 31.6)	0.6 (0.1 to 1.6)	13.4 (2.4 to 33.1)
Lao People's Democratic Republic	All risk factors	4.2 (2.4 to 6.6)	130 (73 to 209.6)	2.8 (1.5 to 4.6)	132.8 (74.6 to 214.2)	6.3 (3.7 to 10)	179.8 (106.3 to 286.3)	5.3 (2.9 to 8.9)	185.1 (109.7 to 292.2)
Lao People's Democratic Republic	Behavioral risks	2.6 (1.5 to 4)	86 (48.5 to 140)	1.8 (0.9 to 3)	87.8 (49.6 to 142.6)	2.9 (1.8 to 4.2)	92.3 (56.8 to 138.1)	2.6 (1.5 to 4.4)	94.9 (58.7 to 141.5)
Lao People's Democratic Republic	Alcohol use	1 (0.6 to 1.6)	37 (20.4 to 61.2)	0.7 (0.4 to 1.3)	37.8 (20.7 to 62.5)	1.3 (0.8 to 2)	45.4 (27.6 to 71.5)	1.3 (0.7 to 2.2)	46.7 (28.4 to 73.4)
Lao People's Democratic Republic	Diet high in red meat	0.3 (0.1 to 0.5)	10.2 (1.8 to 18)	0.2 (0 to 0.4)	10.4 (1.8 to 18.4)	0.5 (0.1 to 0.8)	14.8 (3.3 to 25.5)	0.4 (0.1 to 0.8)	15.3 (3.4 to 26.2)
Lao People's Democratic Republic	Low physical activity	0.1 (0.1 to 0.2)	2.8 (1.5 to 6.6)	0.1 (0 to 0.1)	2.9 (1.5 to 6.8)	0.1 (0.1 to 0.2)	2.9 (1.6 to 6.5)	0.1 (0 to 0.2)	3 (1.6 to 6.7)
Lao People's Democratic Republic	Smoking	0.4 (0.2 to 0.6)	10 (5.1 to 17.1)	0.2 (0.1 to 0.4)	10.2 (5.2 to 17.5)	0.3 (0.2 to 0.5)	7.2 (3.8 to 12.4)	0.2 (0.1 to 0.4)	7.5 (3.9 to 12.7)
Lao People's Democratic Republic	Secondhand smoke	0.9 (0.2 to 1.8)	30.1 (6 to 60.3)	0.6 (0.1 to 1.3)	30.7 (6.1 to 61.7)	0.9 (0.2 to 1.7)	26.9 (5.8 to 54.2)	0.8 (0.2 to 1.6)	27.6 (6 to 55.8)
Lao People's Democratic Republic	Metabolic risks	1.9 (0.5 to 3.8)	50.2 (14.1 to 106.5)	1.2 (0.3 to 2.5)	51.4 (14.4 to 108.5)	3.9 (1.6 to 7.2)	101.5 (38.3 to 188.3)	3 (1.1 to 5.8)	104.6 (39.4 to 193.3)
Lao People's Democratic Republic	High body-mass index	0.9 (0.2 to 2.2)	25.1 (4.6 to 62.4)	0.6 (0.1 to 1.5)	25.7 (4.7 to 63.7)	2.4 (0.6 to 5.1)	63.9 (17 to 135.6)	1.9 (0.5 to 4.1)	65.7 (17.6 to 139)
Lao People's Democratic Republic	High fasting plasma glucose	1 (0.2 to 2.5)	26.4 (4.7 to 66.7)	0.6 (0.1 to 1.6)	27 (4.7 to 68.2)	1.8 (0.4 to 4.3)	43.3 (8.2 to 107.4)	1.3 (0.3 to 3.3)	44.7 (8.5 to 110.2)
Latvia	All risk factors	5.3 (4.1 to 6.7)	153.7 (115.8 to 191)	7.8 (5 to 11.3)	161.6 (121.9 to 199.5)	5.7 (3.8 to 7.9)	147 (98.7 to 207.1)	11.3 (6.8 to 17.2)	158.3 (106.5 to 221.6)

Location	Risk factor	1990				2019			
		Deaths	YLLs (Years of Life Lost)	YLDs (Years Lived with Disability)	DALYs (Disability-Adjusted Life Years)	Deaths	YLLs (Years of Life Lost)	YLDs (Years Lived with Disability)	DALYs (Disability-Adjusted Life Years)
Latvia	Behavioral risks	4 (3.2 to 4.8)	130.2 (103.2 to 156.4)	6.3 (4.1 to 8.9)	136.5 (108.2 to 162.7)	3.8 (2.7 to 5.2)	111.7 (78.6 to 154.8)	8.3 (5.1 to 12.6)	120 (84.7 to 165.8)
Latvia	Alcohol use	1.7 (1.2 to 2.1)	58.2 (42.8 to 72.5)	2.7 (1.7 to 4.1)	60.9 (44.8 to 75.6)	2 (1.3 to 2.7)	59.8 (40.7 to 84.5)	4.4 (2.6 to 6.8)	64.2 (43.3 to 91)
Latvia	Diet high in red meat	0.9 (0.4 to 1.2)	29.4 (12.9 to 38.9)	1.4 (0.6 to 2.2)	30.8 (13.6 to 40.8)	0.7 (0.2 to 1.1)	19.7 (7.1 to 31)	1.5 (0.5 to 2.4)	21.1 (7.6 to 33.2)
Latvia	Low physical activity	0.2 (0.1 to 0.4)	5.3 (3 to 9.1)	0.3 (0.1 to 0.5)	5.6 (3.2 to 9.6)	0.3 (0.1 to 0.4)	5.2 (2.5 to 9.3)	0.4 (0.2 to 0.8)	5.7 (2.7 to 10.1)
Latvia	Smoking	0.9 (0.5 to 1.3)	29 (16.6 to 42.4)	1.4 (0.7 to 2.3)	30.4 (17.4 to 44.8)	0.8 (0.5 to 1.2)	22.5 (12.6 to 34.9)	1.7 (0.9 to 2.9)	24.2 (13.5 to 37.3)
Latvia	Secondhand smoke	0.6 (0.1 to 1)	19 (4.7 to 32.9)	0.9 (0.2 to 1.7)	20 (5 to 34.4)	0.5 (0.1 to 0.9)	14.1 (3.1 to 26.2)	1 (0.2 to 2)	15.2 (3.3 to 28.4)
Latvia	Metabolic risks	1.6 (0.4 to 3)	28.3 (-2.7 to 65.2)	1.9 (0.2 to 3.9)	30.1 (-2.4 to 69.2)	2.3 (0.9 to 4.2)	44.3 (11.6 to 90.3)	3.8 (1.1 to 7.4)	48.1 (12.6 to 96.8)
Latvia	High body-mass index	0.9 (0.1 to 2)	11.3 (-13.8 to 37)	0.9 (-0.3 to 2.5)	12.3 (-13.8 to 39.8)	1.3 (0.3 to 2.7)	22.5 (-2.4 to 51.9)	2 (0.1 to 4.4)	24.5 (-2.4 to 55.2)
Latvia	High fasting plasma glucose	0.7 (0.1 to 1.6)	18 (3.3 to 42.2)	1 (0.2 to 2.4)	18.9 (3.5 to 44)	1 (0.2 to 2.5)	23.9 (4.3 to 58.6)	1.9 (0.3 to 4.9)	25.8 (4.7 to 63.6)
Lebanon	All risk factors	5.2 (3.2 to 7.8)	129 (73.8 to 197.2)	5.2 (2.9 to 8.5)	134.2 (77.1 to 204.1)	9.9 (6 to 15.1)	231.9 (137.1 to 360.7)	18.5 (9.5 to 30.9)	250.3 (150.3 to 390.9)
Lebanon	Behavioral risks	3.2 (2.2 to 4.4)	94.9 (63.7 to 129.8)	3.7 (2.2 to 5.5)	98.7 (66.3 to 135.2)	5.4 (3.7 to 7.6)	145.1 (98.3 to 208.2)	11.8 (6.9 to 18.3)	156.8 (105.3 to 227)
Lebanon	Alcohol use	0.3 (0.2 to 0.5)	10.8 (7.4 to 15.8)	0.4 (0.2 to 0.6)	11.3 (7.7 to 16.3)	0.3 (0.2 to 0.4)	8.1 (4.8 to 12.5)	0.7 (0.4 to 1.1)	8.8 (5.3 to 13.6)
Lebanon	Diet high in red meat	0.6 (0.1 to 1)	20.2 (4.8 to 30.3)	0.8 (0.2 to 1.3)	20.9 (5 to 31.5)	1 (0.3 to 1.6)	29.5 (8.9 to 45.5)	2.4 (0.7 to 4.1)	32 (9.6 to 49.6)
Lebanon	Low physical activity	0.5 (0.2 to 0.8)	11 (4.5 to 21.1)	0.5 (0.2 to 0.9)	11.5 (4.7 to 22)	0.8 (0.3 to 1.4)	17.7 (6.4 to 33.5)	1.5 (0.5 to 2.9)	19.2 (6.9 to 36.3)
Lebanon	Smoking	1.2 (0.8 to 1.8)	33.8 (20.6 to 50.1)	1.3 (0.7 to 2.2)	35.1 (21.4 to 52)	2.5 (1.6 to 3.7)	66.3 (41.4 to 97.9)	5.3 (2.9 to 8.7)	71.6 (44.6 to 106.7)
Lebanon	Secondhand smoke	0.7 (0.2 to 1.4)	23.8 (5.4 to 43.2)	0.9 (0.2 to 1.8)	24.7 (5.6 to 44.8)	1.1 (0.2 to 1.9)	31.7 (6.9 to 58.6)	2.6 (0.6 to 4.9)	34.3 (7.5 to 63.1)
Lebanon	Metabolic risks	2.3 (0.5 to 4.8)	40.4 (-6.8 to 102.3)	1.8 (-0.1 to 4.4)	42.2 (-6.8 to 106.1)	5.4 (1.9 to 10.6)	105.6 (20.6 to 231.5)	8.2 (1.1 to 18.8)	113.7 (21.1 to 249.7)
Lebanon	High body-mass index	0.8 (-0.2 to 2.1)	4.2 (-25.1 to 31.9)	0.3 (-0.9 to 1.5)	4.5 (-26 to 33.1)	2.2 (0.3 to 4.7)	29.7 (-20.4 to 86.5)	1.9 (-2.1 to 6.6)	31.6 (-22.1 to 93.2)
Lebanon	High fasting plasma glucose	1.6 (0.3 to 3.7)	38.1 (7 to 90.2)	1.6 (0.3 to 4)	39.7 (7.2 to 93.5)	3.6 (0.7 to 8.2)	82.4 (15.4 to 191.6)	6.8 (1.2 to 17.1)	89.1 (16.7 to 208.3)
Lesotho	All risk factors	2.3 (1.4 to 3.5)	55.7 (33.1 to 85.5)	1.5 (0.8 to 2.4)	57.2 (34 to 87.8)	6.3 (3.3 to 10.8)	148.9 (76.1 to 263.2)	4 (2 to 7.2)	152.9 (78.2 to 270.2)
Lesotho	Behavioral risks	1.3 (0.9 to 1.9)	38.3 (23.8 to 56.7)	0.9 (0.5 to 1.5)	39.3 (24.5 to 58)	3.1 (1.7 to 4.9)	88.4 (47 to 142.5)	2.2 (1.2 to 3.9)	90.6 (48.1 to 145.8)
Lesotho	Alcohol use	0.5 (0.3 to 0.8)	16.3 (9.7 to 25.2)	0.4 (0.2 to 0.7)	16.7 (10 to 25.8)	1.2 (0.6 to 2)	38.8 (18.7 to 63.6)	1 (0.5 to 1.7)	39.8 (19.1 to 64.9)
Lesotho	Diet high in red meat	0.3 (0.1 to 0.5)	8.5 (1.7 to 14)	0.2 (0 to 0.4)	8.7 (1.8 to 14.3)	0.7 (0.1 to 1.1)	18.6 (4 to 33.4)	0.5 (0.1 to 0.9)	19.1 (4.1 to 34.2)
Lesotho	Low physical activity	0.1 (0.1 to 0.2)	2.2 (1.2 to 4.2)	0.1 (0 to 0.1)	2.2 (1.2 to 4.3)	0.2 (0.1 to 0.4)	5 (2.4 to 10)	0.1 (0.1 to 0.3)	5.2 (2.5 to 10.3)
Lesotho	Smoking	0.1 (0.1 to 0.2)	2.2 (1.3 to 3.4)	0.1 (0 to 0.1)	2.3 (1.3 to 3.5)	0.3 (0.1 to 0.4)	4.6 (2.5 to 7.5)	0.1 (0.1 to 0.2)	4.7 (2.6 to 7.8)
Lesotho	Secondhand smoke	0.4 (0.1 to 0.7)	10.6 (2.4 to 20.6)	0.3 (0.1 to 0.5)	10.9 (2.5 to 21)	0.8 (0.2 to 1.7)	25.3 (5.6 to 50.9)	0.6 (0.1 to 1.3)	25.9 (5.8 to 52.2)
Lesotho	Metabolic risks	1.1 (0.3 to 2.2)	19.2 (3.2 to 41.9)	0.6 (0.1 to 1.3)	19.8 (3.3 to 43)	3.6 (1.1 to 7.5)	67.3 (10.9 to 153.2)	2 (0.4 to 4.4)	69.3 (11.2 to 157)
Lesotho	High body-mass index	0.4 (0.1 to 0.9)	5.9 (-3.9 to 17.9)	0.2 (0 to 0.5)	6.1 (-3.9 to 18.4)	1.5 (0.1 to 3.4)	21.1 (-14.6 to 60.7)	0.7 (-0.3 to 1.9)	21.8 (-14.8 to 62.2)
Lesotho	High fasting plasma glucose	0.7 (0.1 to 1.6)	13.9 (2.4 to 34.6)	0.4 (0.1 to 1)	14.3 (2.5 to 35.6)	2.2 (0.4 to 5.6)	49.9 (8.9 to 129.7)	1.4 (0.3 to 3.6)	51.3 (9.2 to 132.9)
Liberia	All risk factors	2.6 (1.6 to 3.8)	66.3 (41.1 to 96.3)	1.6 (0.9 to 2.5)	67.9 (42 to 98.5)	3.6 (2 to 6.4)	86 (46.4 to 150.8)	2.5 (1.3 to 4.4)	88.5 (47.6 to 155.4)
Liberia	Behavioral risks	1.5 (1.1 to 2)	44.8 (32.2 to 60.9)	1 (0.6 to 1.5)	45.8 (32.8 to 62.3)	1.7 (1.1 to 2.6)	48.5 (30.5 to 75.8)	1.3 (0.7 to 2.2)	49.8 (31.4 to 78.1)
Liberia	Alcohol use	0.9 (0.6 to 1.2)	27.8 (19.1 to 38.5)	0.6 (0.4 to 0.9)	28.4 (19.5 to 39.5)	1 (0.6 to 1.5)	29.6 (17.1 to 47.7)	0.8 (0.4 to 1.3)	30.4 (17.6 to 48.9)
Liberia	Diet high in red meat	0.2 (0 to 0.2)	4.5 (1 to 7.1)	0.1 (0 to 0.2)	4.6 (1 to 7.2)	0.2 (0 to 0.3)	4.8 (1.1 to 8.9)	0.1 (0 to 0.2)	4.9 (1.1 to 9.1)
Liberia	Low physical activity	0.2 (0.1 to 0.3)	3.5 (1.8 to 6.6)	0.1 (0 to 0.2)	3.6 (1.8 to 6.8)	0.2 (0.1 to 0.4)	4.6 (2.2 to 8.9)	0.1 (0.1 to 0.3)	4.7 (2.2 to 9.1)
Liberia	Smoking	0.1 (0.1 to 0.2)	2.7 (1.2 to 4.6)	0.1 (0 to 0.1)	2.7 (1.2 to 4.7)	0.1 (0 to 0.2)	2.7 (1.1 to 5)	0.1 (0 to 0.2)	2.8 (1.2 to 5.2)
Liberia	Secondhand smoke	0.3 (0.1 to 0.5)	7.8 (1.8 to 14.2)	0.2 (0 to 0.3)	8 (1.9 to 14.5)	0.3 (0.1 to 0.6)	8.3 (1.7 to 16.8)	0.2 (0 to 0.5)	8.5 (1.7 to 17.3)
Liberia	Metabolic risks	1.2 (0.4 to 2.4)	23.8 (4.3 to 50.5)	0.6 (0.2 to 1.4)	24.5 (4.5 to 51.9)	2.1 (0.7 to 4.5)	40.9 (7.8 to 93.1)	1.3 (0.3 to 2.9)	42.2 (8 to 96)
Liberia	High body-mass index	0.5 (0 to 1)	7.7 (-4.7 to 21.1)	0.2 (0 to 0.6)	8 (-4.7 to 21.6)	0.7 (0.1 to 1.8)	11.4 (-7.8 to 34.7)	0.4 (-0.1 to 1.1)	11.8 (-8 to 35.6)
Liberia	High fasting plasma glucose	0.8 (0.1 to 1.8)	16.8 (3 to 41.5)	0.4 (0.1 to 1.1)	17.2 (3 to 42.5)	1.4 (0.3 to 3.6)	31.4 (6 to 80)	0.9 (0.2 to 2.4)	32.3 (6.2 to 82)

Location	Risk factor	1990				2019			
		Deaths	YLLs (Years of Life Lost)	YLDs (Years Lived with Disability)	DALYs (Disability-Adjusted Life Years)	Deaths	YLLs (Years of Life Lost)	YLDs (Years Lived with Disability)	DALYs (Disability-Adjusted Life Years)
Libya	All risk factors	2 (1 to 3.3)	49.2 (23 to 83.2)	2 (0.9 to 3.6)	51.2 (24.1 to 86.2)	3.8 (1.9 to 6.7)	92.2 (40.7 to 170.2)	4.7 (2 to 8.9)	96.9 (42.6 to 178.7)
Libya	Behavioral risks	1 (0.6 to 1.5)	30.5 (17 to 46.5)	1.2 (0.6 to 1.9)	31.7 (17.6 to 48.2)	1.4 (0.8 to 2.1)	41.9 (22.2 to 65.9)	2.1 (1 to 3.5)	44 (23.3 to 69.7)
Libya	Alcohol use	0 (0 to 0)	0.2 (0.1 to 0.3)	0 (0 to 0)	0.2 (0.1 to 0.3)	0 (0 to 0)	1 (0.4 to 1.5)	0 (0 to 0.1)	1 (0.4 to 1.6)
Libya	Diet high in red meat	0.3 (0.1 to 0.5)	10.8 (3 to 16.8)	0.4 (0.1 to 0.7)	11.2 (3.2 to 17.4)	0.3 (0.1 to 0.5)	10.6 (2.1 to 17.1)	0.5 (0.1 to 1)	11.1 (2.2 to 18.1)
Libya	Low physical activity	0.2 (0.1 to 0.4)	6 (2.3 to 11.3)	0.2 (0.1 to 0.5)	6.3 (2.4 to 11.7)	0.4 (0.1 to 0.7)	9.7 (3.5 to 18.8)	0.5 (0.2 to 1)	10.2 (3.7 to 19.7)
Libya	Smoking	0 (0 to 0.1)	1 (0.6 to 1.8)	0 (0 to 0.1)	1.1 (0.6 to 1.9)	0 (0 to 0.1)	1.1 (0.6 to 1.9)	0.1 (0 to 0.1)	1.2 (0.6 to 2)
Libya	Secondhand smoke	0.4 (0.1 to 0.7)	13.3 (3.2 to 24.4)	0.5 (0.1 to 0.9)	13.8 (3.3 to 25.2)	0.6 (0.1 to 1.2)	20.7 (4.4 to 38.2)	1 (0.2 to 2)	21.7 (4.7 to 40.1)
Libya	Metabolic risks	1.1 (0.3 to 2.3)	20.5 (-3.2 to 51.8)	0.9 (0 to 2.2)	21.4 (-3.2 to 54)	2.7 (0.8 to 5.5)	55 (4.3 to 127.6)	2.8 (0.2 to 6.7)	57.8 (4.2 to 134.2)
Libya	High body-mass index	0.4 (-0.1 to 1.1)	2.7 (-12.5 to 18.2)	0.2 (-0.4 to 0.9)	2.9 (-12.8 to 18.9)	0.9 (-0.1 to 1.9)	7.4 (-20.6 to 34.8)	0.3 (-1.1 to 1.8)	7.7 (-21.9 to 36.2)
Libya	High fasting plasma glucose	0.7 (0.1 to 1.8)	18.9 (3.3 to 46)	0.8 (0.1 to 2)	19.7 (3.5 to 47.5)	2 (0.4 to 4.7)	51.4 (10.6 to 123)	2.7 (0.5 to 6.6)	54.1 (11.1 to 129)
Lithuania	All risk factors	5 (3.9 to 6.2)	147.2 (113.2 to 177.9)	8.3 (5.5 to 11.7)	155.6 (118.8 to 188)	5.2 (3.8 to 6.8)	138.5 (102.6 to 182.4)	10.5 (6.8 to 15.7)	149 (110.1 to 197)
Lithuania	Behavioral risks	3.9 (3.2 to 4.6)	128.8 (106 to 150.7)	7.1 (4.8 to 10)	136 (111.4 to 159.2)	3.8 (2.9 to 5)	113.8 (85.3 to 150.3)	8.6 (5.6 to 12.9)	122.4 (91.7 to 162.5)
Lithuania	Alcohol use	2 (1.5 to 2.4)	67.2 (51.8 to 81.6)	3.7 (2.4 to 5.3)	70.9 (54.8 to 86.4)	2.2 (1.6 to 2.9)	68.2 (49.1 to 91.5)	5.1 (3.2 to 7.9)	73.4 (52.9 to 99)
Lithuania	Diet high in red meat	0.9 (0.5 to 1.3)	30.1 (14.9 to 40.2)	1.7 (0.7 to 2.6)	31.8 (15.6 to 42.6)	0.8 (0.4 to 1.2)	22.5 (10.4 to 33.4)	1.7 (0.7 to 2.8)	24.2 (11.2 to 35.9)
Lithuania	Low physical activity	0.2 (0.1 to 0.3)	4.8 (2.7 to 8.3)	0.3 (0.2 to 0.5)	5.1 (2.9 to 8.8)	0.2 (0.1 to 0.4)	4.6 (2.4 to 8.2)	0.4 (0.2 to 0.7)	5 (2.6 to 8.8)
Lithuania	Smoking	0.6 (0.4 to 0.9)	21.3 (12.3 to 31.4)	1.2 (0.6 to 1.9)	22.5 (12.9 to 33.2)	0.6 (0.4 to 0.9)	17.7 (10.4 to 26.9)	1.3 (0.7 to 2.2)	19.1 (11.1 to 28.9)
Lithuania	Secondhand smoke	0.5 (0.1 to 0.8)	16.5 (4 to 28.4)	0.9 (0.2 to 1.7)	17.4 (4.2 to 29.9)	0.4 (0.1 to 0.6)	11.1 (2.6 to 19.9)	0.8 (0.2 to 1.6)	11.9 (2.8 to 21.5)
Lithuania	Metabolic risks	1.3 (0.4 to 2.5)	22.4 (-3.4 to 52.6)	1.4 (-0.1 to 3.3)	23.8 (-3.3 to 55.6)	1.7 (0.6 to 3.1)	31.6 (6.8 to 62.7)	2.5 (0.5 to 5)	34.1 (7.3 to 68.1)
Lithuania	High body-mass index	0.8 (0 to 1.7)	8.4 (-13.2 to 30)	0.6 (-0.7 to 2)	9.1 (-13.9 to 31.8)	1.1 (0.3 to 2.1)	16.8 (-2.5 to 39)	1.3 (-0.2 to 3.2)	18.1 (-2.7 to 41.5)
Lithuania	High fasting plasma glucose	0.6 (0.1 to 1.3)	14.7 (2.7 to 34.7)	0.9 (0.2 to 2.1)	15.6 (2.8 to 36.5)	0.7 (0.1 to 1.6)	16 (2.8 to 38.5)	1.2 (0.2 to 3.2)	17.2 (3.1 to 41.9)
Luxembourg	All risk factors	11 (9.1 to 13.2)	289.3 (242.2 to 339.5)	22.1 (15.1 to 30.8)	311.4 (261.2 to 363.4)	7 (5.2 to 9.4)	160.3 (120.5 to 207.7)	21.2 (12.9 to 32)	181.6 (136.8 to 235.6)
Luxembourg	Behavioral risks	9.3 (7.9 to 10.7)	258.1 (219.2 to 297.8)	19.5 (13.4 to 27.5)	277.7 (236.2 to 319.1)	4.9 (4 to 6.1)	124.2 (99.8 to 150.7)	16.3 (10.3 to 23.5)	140.6 (111.9 to 171.9)
Luxembourg	Alcohol use	5 (4.1 to 6.1)	141.3 (115.1 to 169.1)	10.7 (7.1 to 15.3)	152 (124.1 to 182.1)	2.6 (2 to 3.4)	67.3 (51.6 to 87.4)	8.8 (5.6 to 12.9)	76.2 (57.6 to 98.7)
Luxembourg	Diet high in red meat	1.8 (0.9 to 2.5)	50.1 (25 to 68.8)	3.8 (1.8 to 6.1)	53.9 (26.8 to 74.2)	1.1 (0.5 to 1.5)	27.1 (13.4 to 39)	3.6 (1.6 to 5.9)	30.7 (14.9 to 44.4)
Luxembourg	Low physical activity	0.5 (0.2 to 0.9)	9.7 (3.7 to 19.4)	0.8 (0.3 to 1.7)	10.5 (4 to 21.1)	0.3 (0.1 to 0.6)	6 (2.1 to 11.7)	0.8 (0.3 to 1.8)	6.8 (2.4 to 13.4)
Luxembourg	Smoking	2.4 (1.7 to 3.2)	69 (47.9 to 92)	5.2 (3.1 to 7.9)	74.2 (51.3 to 99.6)	1.2 (0.8 to 1.6)	28.9 (19.9 to 39.5)	3.8 (2.2 to 6)	32.7 (22.5 to 44.6)
Luxembourg	Secondhand smoke	0.6 (0.1 to 1.1)	19.2 (4.4 to 33.6)	1.4 (0.3 to 2.6)	20.6 (4.8 to 35.7)	0.3 (0.1 to 0.5)	8.1 (2 to 14.4)	1.1 (0.2 to 2)	9.2 (2.2 to 16.2)
Luxembourg	Metabolic risks	2.4 (0.9 to 4.4)	44.4 (10.5 to 87.5)	3.7 (1 to 7.2)	48 (11.2 to 94.4)	2.8 (1 to 5.3)	49.4 (14.5 to 98.1)	6.7 (2 to 13.8)	56.1 (16.6 to 111.1)
Luxembourg	High body-mass index	1.4 (0.3 to 2.9)	23.5 (-2.1 to 55.2)	2 (0 to 4.6)	25.5 (-2.1 to 59.4)	1 (0.3 to 2)	14.8 (-0.7 to 34.5)	2 (-0.1 to 4.8)	16.8 (-0.6 to 39.3)
Luxembourg	High fasting plasma glucose	1.1 (0.2 to 2.5)	22 (4 to 51.4)	1.8 (0.3 to 4.5)	23.8 (4.3 to 56.8)	1.9 (0.4 to 4.3)	37 (7.4 to 83.3)	5 (1 to 12.2)	42 (8.3 to 94.1)
Madagascar	All risk factors	1.8 (1.2 to 2.6)	52.3 (33 to 74.1)	1.2 (0.7 to 1.9)	53.5 (33.8 to 75.6)	2.2 (1.2 to 3.6)	56.8 (31.8 to 93.4)	1.5 (0.8 to 2.6)	58.4 (32.5 to 95.9)
Madagascar	Behavioral risks	1.3 (0.9 to 1.8)	42.1 (27.3 to 58.5)	0.9 (0.5 to 1.4)	43 (27.9 to 59.7)	1.2 (0.7 to 1.8)	36 (21.2 to 55.6)	0.9 (0.5 to 1.4)	36.9 (21.8 to 56.8)
Madagascar	Alcohol use	0.5 (0.3 to 0.7)	15.5 (9.9 to 22.4)	0.3 (0.2 to 0.5)	15.8 (10.1 to 22.9)	0.4 (0.2 to 0.7)	13.8 (7.4 to 21.9)	0.3 (0.2 to 0.6)	14.1 (7.5 to 22.4)
Madagascar	Diet high in red meat	0.3 (0.1 to 0.5)	10.8 (2.6 to 16.1)	0.2 (0.1 to 0.4)	11.1 (2.7 to 16.5)	0.3 (0.1 to 0.5)	8.4 (1.6 to 14.3)	0.2 (0 to 0.4)	8.6 (1.6 to 14.8)
Madagascar	Low physical activity	0.1 (0 to 0.2)	1.7 (1.1 to 4.3)	0 (0 to 0.1)	1.7 (1.1 to 4.4)	0.1 (0 to 0.2)	1.9 (1.2 to 4.6)	0.1 (0 to 0.1)	2 (1.2 to 4.7)
Madagascar	Smoking	0.1 (0 to 0.2)	2.7 (1.2 to 4.4)	0.1 (0 to 0.1)	2.8 (1.2 to 4.6)	0.1 (0 to 0.1)	1.9 (0.9 to 3.2)	0.1 (0 to 0.1)	1.9 (0.9 to 3.3)
Madagascar	Secondhand smoke	0.4 (0.1 to 0.7)	12.9 (2.9 to 24.2)	0.3 (0.1 to 0.5)	13.2 (3 to 24.7)	0.3 (0.1 to 0.7)	11 (2.4 to 22)	0.3 (0.1 to 0.5)	11.3 (2.5 to 22.5)
Madagascar	Metabolic risks	0.6 (0.1 to 1.2)	11.2 (0.9 to 27)	0.3 (0.1 to 0.7)	11.5 (1 to 27.6)	1.1 (0.3 to 2.3)	22.5 (4.9 to 50.5)	0.7 (0.2 to 1.5)	23.1 (5.1 to 51.8)
Madagascar	High body-mass index	0.1 (0 to 0.3)	0.9 (-3.9 to 5.6)	0.1 (0 to 0.2)	0.9 (-3.9 to 5.7)	0.4 (0.1 to 0.9)	6.3 (-2.1 to 17.1)	0.2 (0 to 0.5)	6.6 (-2.1 to 17.6)

Location	Risk factor	1990				2019			
		Deaths	YLLs (Years of Life Lost)	YLDs (Years Lived with Disability)	DALYs (Disability-Adjusted Life Years)	Deaths	YLLs (Years of Life Lost)	YLDs (Years Lived with Disability)	DALYs (Disability-Adjusted Life Years)
Madagascar	High fasting plasma glucose	0.4 (0.1 to 1)	10.5 (1.9 to 24.9)	0.3 (0 to 0.7)	10.7 (1.9 to 25.6)	0.7 (0.1 to 1.8)	16.6 (3.1 to 43.1)	0.5 (0.1 to 1.2)	17.1 (3.1 to 43.9)
Malawi	All risk factors	1.5 (0.8 to 2.4)	38.5 (22 to 61.2)	0.9 (0.5 to 1.6)	39.5 (22.6 to 62.7)	2.6 (1.5 to 4.1)	59.1 (33.3 to 94.6)	1.6 (0.9 to 2.8)	60.8 (34.5 to 97.3)
Malawi	Behavioral risks	0.9 (0.6 to 1.3)	27.7 (18.3 to 38.7)	0.6 (0.4 to 0.9)	28.3 (18.6 to 39.5)	1.3 (0.9 to 1.8)	36.3 (22.9 to 51.5)	1 (0.5 to 1.5)	37.2 (23.4 to 52.9)
Malawi	Alcohol use	0.4 (0.3 to 0.6)	13.3 (8.2 to 19.5)	0.3 (0.2 to 0.5)	13.6 (8.4 to 20)	0.7 (0.4 to 1)	19.1 (11.4 to 28.7)	0.5 (0.3 to 0.8)	19.6 (11.7 to 29.4)
Malawi	Diet high in red meat	0.1 (0 to 0.1)	2.7 (0.9 to 4.3)	0.1 (0 to 0.1)	2.8 (0.9 to 4.4)	0.2 (0 to 0.3)	4.7 (1.1 to 7.7)	0.1 (0 to 0.2)	4.8 (1.1 to 7.9)
Malawi	Low physical activity	0.1 (0 to 0.1)	1.8 (1.1 to 3.7)	0 (0 to 0.1)	1.8 (1.1 to 3.8)	0.1 (0.1 to 0.2)	2.3 (1.3 to 4.9)	0.1 (0 to 0.1)	2.3 (1.3 to 5)
Malawi	Smoking	0.1 (0 to 0.2)	2.5 (1.1 to 4.3)	0.1 (0 to 0.1)	2.5 (1.1 to 4.4)	0.2 (0.1 to 0.3)	3.3 (1.3 to 5.5)	0.1 (0 to 0.2)	3.4 (1.3 to 5.7)
Malawi	Secondhand smoke	0.2 (0.1 to 0.5)	8.2 (1.8 to 15.3)	0.2 (0 to 0.3)	8.3 (1.9 to 15.6)	0.3 (0.1 to 0.5)	7.9 (2 to 14.8)	0.2 (0 to 0.4)	8.1 (2 to 15.1)
Malawi	Metabolic risks	0.6 (0.1 to 1.4)	11.6 (-0.8 to 31.3)	0.3 (0 to 0.8)	11.9 (-0.7 to 31.9)	1.4 (0.4 to 2.8)	24.7 (4 to 56.1)	0.7 (0.2 to 1.6)	25.5 (4.2 to 57.6)
Malawi	High body-mass index	0.1 (0 to 0.3)	-0.6 (-6 to 4.6)	0 (-0.1 to 0.2)	-0.6 (-6 to 4.8)	0.5 (0 to 1.1)	5.9 (-5.2 to 18.4)	0.2 (-0.1 to 0.6)	6.1 (-5.2 to 19)
Malawi	High fasting plasma glucose	0.5 (0.1 to 1.3)	12.4 (2.1 to 30.9)	0.3 (0.1 to 0.8)	12.7 (2.2 to 31.6)	0.9 (0.2 to 2.2)	19.5 (3.5 to 48.1)	0.6 (0.1 to 1.4)	20.1 (3.6 to 49.5)
Malaysia	All risk factors	5.2 (3 to 7.8)	142.6 (84.1 to 208.7)	4.6 (2.5 to 7.5)	147.2 (86 to 215.4)	8.3 (4.5 to 13)	206.7 (111.7 to 324.8)	10.9 (5.5 to 18)	217.6 (119.1 to 342.3)
Malaysia	Behavioral risks	1.9 (1.2 to 2.6)	55.6 (33.5 to 78.3)	1.8 (0.9 to 2.7)	57.4 (34.6 to 80.8)	2 (1.2 to 3)	56.5 (30.5 to 84.8)	3 (1.5 to 4.9)	59.5 (32 to 89.3)
Malaysia	Alcohol use	0.3 (0.2 to 0.4)	8.3 (5 to 12)	0.3 (0.1 to 0.4)	8.5 (5.2 to 12.4)	0.2 (0.1 to 0.4)	6.8 (3.6 to 10.7)	0.4 (0.2 to 0.6)	7.1 (3.8 to 11.3)
Malaysia	Diet high in red meat	0.4 (0.1 to 0.6)	12.6 (2.4 to 17.8)	0.4 (0.1 to 0.7)	13 (2.5 to 18.3)	0.4 (0.1 to 0.6)	12.1 (2.2 to 19)	0.6 (0.1 to 1.1)	12.7 (2.3 to 20.2)
Malaysia	Low physical activity	0.2 (0.1 to 0.4)	4.6 (2.4 to 8.7)	0.2 (0.1 to 0.3)	4.7 (2.5 to 9.1)	0.3 (0.1 to 0.6)	6.2 (2.9 to 11.6)	0.3 (0.1 to 0.7)	6.5 (3.1 to 12.2)
Malaysia	Smoking	0.2 (0.1 to 0.3)	4.3 (2.2 to 7)	0.1 (0.1 to 0.3)	4.5 (2.2 to 7.3)	0.2 (0.1 to 0.3)	4.1 (2.3 to 6.5)	0.2 (0.1 to 0.4)	4.4 (2.4 to 6.9)
Malaysia	Secondhand smoke	0.9 (0.2 to 1.5)	27.5 (6.8 to 48)	0.9 (0.2 to 1.6)	28.4 (7 to 49.5)	1 (0.2 to 1.8)	28.9 (7.3 to 52.9)	1.5 (0.3 to 2.9)	30.4 (7.6 to 55.7)
Malaysia	Metabolic risks	3.7 (1.5 to 6.5)	95.2 (39.5 to 164)	3.1 (1.2 to 5.7)	98.3 (40.8 to 169.5)	6.8 (3 to 11.8)	163.2 (70.1 to 282.4)	8.6 (3.6 to 15.2)	171.7 (73.9 to 295.6)
Malaysia	High body-mass index	2.4 (0.7 to 4.8)	66.1 (21.3 to 125.9)	2.1 (0.6 to 4.3)	68.2 (21.9 to 129.5)	4.9 (1.6 to 9.1)	120.8 (39.2 to 224.7)	6.3 (2 to 12.3)	127 (41.3 to 236.3)
Malaysia	High fasting plasma glucose	1.4 (0.3 to 3.3)	33.4 (6.2 to 79.2)	1.1 (0.2 to 2.8)	34.5 (6.4 to 81.5)	2.4 (0.5 to 5.6)	53.6 (10.3 to 126.8)	2.9 (0.5 to 7)	56.5 (11 to 134)
Maldives	All risk factors	2.9 (1.3 to 5.4)	79.7 (34.4 to 147.4)	2.3 (1 to 4.3)	82 (35.4 to 151.8)	3.6 (1.9 to 5.4)	85.5 (46.8 to 128.4)	5.3 (2.7 to 8.5)	90.8 (49.9 to 136.9)
Maldives	Behavioral risks	1.3 (0.6 to 2.1)	37.2 (15.3 to 63.6)	1.1 (0.4 to 1.9)	38.3 (15.8 to 65.2)	1.1 (0.6 to 1.6)	27 (14.8 to 41.1)	1.7 (0.8 to 2.8)	28.7 (15.7 to 43.3)
Maldives	Alcohol use	0 (0 to 0.1)	1.2 (0 to 3.5)	0 (0 to 0.1)	1.2 (0 to 3.6)	0.1 (0 to 0.1)	2.4 (0.8 to 4.4)	0.2 (0 to 0.3)	2.5 (0.9 to 4.7)
Maldives	Diet high in red meat	0.1 (0 to 0.1)	2.6 (1 to 4.3)	0.1 (0 to 0.1)	2.7 (1 to 4.4)	0.1 (0 to 0.2)	2.9 (0.8 to 4.5)	0.2 (0.1 to 0.3)	3.1 (0.9 to 4.8)
Maldives	Low physical activity	0.2 (0.1 to 0.3)	4 (1.7 to 8.3)	0.1 (0.1 to 0.3)	4.1 (1.7 to 8.5)	0.1 (0.1 to 0.3)	3 (1.4 to 5.9)	0.2 (0.1 to 0.4)	3.2 (1.5 to 6.2)
Maldives	Smoking	0.3 (0.1 to 0.5)	7.2 (3.1 to 14)	0.2 (0.1 to 0.4)	7.4 (3.2 to 14.4)	0.2 (0.1 to 0.3)	4.5 (2.5 to 7.1)	0.3 (0.1 to 0.5)	4.8 (2.6 to 7.6)
Maldives	Secondhand smoke	0.7 (0.1 to 1.4)	22.9 (4.1 to 46.9)	0.6 (0.1 to 1.3)	23.5 (4.2 to 48)	0.5 (0.1 to 1)	14.8 (3.6 to 26)	0.9 (0.2 to 1.7)	15.8 (3.9 to 27.6)
Maldives	Metabolic risks	1.8 (0.5 to 4)	45.6 (11.5 to 104)	1.3 (0.3 to 3)	47 (11.8 to 106.9)	2.7 (1.1 to 4.6)	63.6 (26.6 to 106.4)	3.9 (1.5 to 6.9)	67.4 (28.2 to 112.9)
Maldives	High body-mass index	0.9 (0.1 to 2.6)	25.1 (3.9 to 71.6)	0.7 (0.1 to 2)	25.8 (4.1 to 73.8)	1.8 (0.5 to 3.5)	45.1 (14.1 to 86.2)	2.7 (0.8 to 5.4)	47.9 (14.9 to 91.4)
Maldives	High fasting plasma glucose	0.9 (0.2 to 2.3)	21.7 (4.1 to 56.3)	0.7 (0.1 to 1.7)	22.4 (4.2 to 57.9)	1.1 (0.2 to 2.4)	21.6 (4 to 49.9)	1.3 (0.2 to 3.3)	22.9 (4.2 to 53)
Mali	All risk factors	1.8 (1.1 to 2.7)	49.3 (30.7 to 74.1)	1.1 (0.6 to 1.9)	50.5 (31.5 to 75.7)	2.8 (1.6 to 4.5)	73.1 (41.4 to 114.4)	2.1 (1.1 to 3.4)	75.2 (42.4 to 117.5)
Mali	Behavioral risks	1.1 (0.7 to 1.4)	33.3 (22 to 45.1)	0.7 (0.4 to 1.1)	34.1 (22.4 to 45.9)	1.4 (0.9 to 2)	42.5 (27.1 to 61.3)	1.1 (0.6 to 1.7)	43.6 (27.8 to 63.1)
Mali	Alcohol use	0.3 (0.2 to 0.5)	10.9 (7.3 to 15.3)	0.2 (0.1 to 0.4)	11.1 (7.5 to 15.7)	0.5 (0.3 to 0.7)	14.4 (8.8 to 21.6)	0.4 (0.2 to 0.6)	14.8 (9.2 to 22.2)
Mali	Diet high in red meat	0.4 (0.1 to 0.5)	11.2 (2.6 to 16.4)	0.2 (0.1 to 0.4)	11.5 (2.7 to 16.8)	0.5 (0.2 to 0.8)	14.9 (4.7 to 23.3)	0.4 (0.1 to 0.7)	15.3 (4.8 to 24)
Mali	Low physical activity	0.1 (0.1 to 0.2)	2.3 (1.3 to 4.7)	0.1 (0 to 0.1)	2.4 (1.4 to 4.8)	0.1 (0.1 to 0.2)	2.8 (1.6 to 5.3)	0.1 (0 to 0.2)	2.9 (1.6 to 5.5)
Mali	Smoking	0 (0 to 0.1)	1.2 (0.5 to 2.2)	0 (0 to 0.1)	1.2 (0.5 to 2.2)	0.1 (0 to 0.1)	2.1 (0.9 to 3.7)	0.1 (0 to 0.1)	2.1 (0.9 to 3.8)
Mali	Secondhand smoke	0.3 (0.1 to 0.5)	8.6 (2 to 15.2)	0.2 (0 to 0.3)	8.8 (2.1 to 15.4)	0.3 (0.1 to 0.6)	9.7 (2.2 to 17.8)	0.3 (0.1 to 0.5)	10 (2.2 to 18.4)
Mali	Metabolic risks	0.8 (0.2 to 1.6)	17.2 (4.3 to 38.6)	0.4 (0.1 to 1)	17.7 (4.4 to 39.3)	1.5 (0.5 to 3.1)	33.4 (8.6 to 71.4)	1 (0.3 to 2.1)	34.4 (8.8 to 73.5)
Mali	High body-mass index	0.2 (0 to 0.5)	4.4 (-0.5 to 12)	0.1 (0 to 0.3)	4.6 (-0.5 to 12.3)	0.5 (0.1 to 1.2)	9.8 (-2.9 to 25.2)	0.3 (0 to 0.8)	10.1 (-2.9 to 25.9)

Location	Risk factor	1990				2019			
		Deaths	YLLs (Years of Life Lost)	YLDs (Years Lived with Disability)	DALYs (Disability-Adjusted Life Years)	Deaths	YLLs (Years of Life Lost)	YLDs (Years Lived with Disability)	DALYs (Disability-Adjusted Life Years)
Mali	High fasting plasma glucose	0.5 (0.1 to 1.3)	13.1 (2.4 to 32)	0.3 (0.1 to 0.8)	13.4 (2.4 to 32.8)	1 (0.2 to 2.5)	24.7 (4.9 to 59.7)	0.7 (0.1 to 1.8)	25.4 (5.1 to 61.3)
Malta	All risk factors	10 (7.5 to 12.9)	256 (195.1 to 319.1)	17.3 (11.2 to 24.7)	273.3 (209.2 to 339.2)	6.5 (4.7 to 8.9)	157.9 (114.9 to 212.2)	18.2 (11.1 to 27.5)	176.1 (128.1 to 237.5)
Malta	Behavioral risks	7.1 (5.8 to 8.3)	198.5 (162.2 to 231.1)	13.2 (8.5 to 18.7)	211.7 (172.6 to 247.1)	4.2 (3.3 to 5.2)	115.3 (92.1 to 141.8)	13.1 (8.3 to 19.2)	128.4 (102.6 to 156.9)
Malta	Alcohol use	2.7 (2.1 to 3.4)	76.6 (59.8 to 96)	5.1 (3.2 to 7.4)	81.6 (63.5 to 102.1)	1.8 (1.3 to 2.3)	49.6 (36.6 to 63.8)	5.6 (3.4 to 8.5)	55.2 (40.8 to 70.4)
Malta	Diet high in red meat	1.5 (0.7 to 2)	41.8 (19.2 to 56.1)	2.8 (1.2 to 4.4)	44.6 (20.8 to 60)	0.9 (0.4 to 1.3)	23.9 (10.4 to 34)	2.7 (1.1 to 4.4)	26.6 (11.7 to 37.8)
Malta	Low physical activity	1 (0.3 to 1.6)	22.5 (7.1 to 39.5)	1.6 (0.5 to 3)	24.1 (7.5 to 42.5)	0.6 (0.2 to 1.1)	14.4 (4.8 to 25)	1.7 (0.6 to 3.2)	16.1 (5.3 to 28.2)
Malta	Smoking	1.9 (1.3 to 2.6)	56.7 (39.5 to 75.9)	3.7 (2.1 to 5.7)	60.4 (41.8 to 81)	1 (0.7 to 1.3)	29.4 (20.9 to 39.7)	3.3 (1.9 to 5.1)	32.7 (23 to 44.2)
Malta	Secondhand smoke	0.7 (0.2 to 1.2)	20.2 (4.6 to 35.7)	1.3 (0.3 to 2.5)	21.5 (4.9 to 37.8)	0.3 (0.1 to 0.5)	8.8 (2.1 to 15.6)	1 (0.2 to 1.9)	9.8 (2.4 to 17.3)
Malta	Metabolic risks	3.7 (1.3 to 7.1)	72.6 (21.1 to 146.8)	5.2 (1.5 to 10.8)	77.9 (22.9 to 157.2)	2.9 (1 to 5.5)	53.5 (14.3 to 109.5)	6.4 (1.7 to 13.4)	59.9 (16 to 121.1)
Malta	High body-mass index	1.3 (0.3 to 2.8)	22.3 (-0.9 to 53.1)	1.6 (0 to 3.7)	23.9 (-0.8 to 56.5)	1 (0.2 to 2)	13.6 (-3.8 to 35.8)	1.6 (-0.3 to 4)	15.2 (-4.3 to 39.8)
Malta	High fasting plasma glucose	2.5 (0.5 to 5.5)	52.9 (10.2 to 118.3)	3.8 (0.7 to 9.2)	56.7 (10.8 to 126.2)	2.1 (0.4 to 4.6)	42.4 (8.3 to 96.3)	5.1 (1 to 12.1)	47.5 (9.5 to 107.7)
Marshall Islands	All risk factors	7.1 (3.9 to 11.2)	197.1 (111.8 to 309.5)	5.4 (2.8 to 8.9)	202.6 (114.7 to 316.8)	15 (7.4 to 25.2)	409.7 (200.4 to 699.4)	12 (5.7 to 20.6)	421.7 (207.1 to 717)
Marshall Islands	Behavioral risks	2.5 (1.6 to 3.5)	75.4 (47.1 to 107.2)	2 (1.2 to 3)	77.4 (48.3 to 110.2)	4.1 (2.4 to 6.5)	123 (68.5 to 202.1)	3.5 (1.9 to 6.1)	126.5 (70.8 to 207.9)
Marshall Islands	Alcohol use	0.2 (0.1 to 0.4)	7.7 (3.1 to 13.9)	0.2 (0.1 to 0.4)	7.9 (3.1 to 14.3)	0.4 (0.2 to 0.9)	16.4 (6.3 to 32.9)	0.5 (0.2 to 1)	16.8 (6.5 to 34)
Marshall Islands	Diet high in red meat	0.6 (0.2 to 0.9)	18.7 (4.9 to 28.5)	0.5 (0.1 to 0.8)	19.2 (5.1 to 29.4)	1 (0.2 to 1.7)	30.1 (8.2 to 52.9)	0.9 (0.2 to 1.6)	31 (8.5 to 54.5)
Marshall Islands	Low physical activity	0.4 (0.2 to 0.8)	10.3 (3.7 to 20.4)	0.3 (0.1 to 0.6)	10.6 (3.8 to 21.1)	0.8 (0.3 to 1.5)	18.5 (6.1 to 38.7)	0.6 (0.2 to 1.2)	19 (6.3 to 39.8)
Marshall Islands	Smoking	0.5 (0.3 to 0.7)	13.7 (7.4 to 22.2)	0.4 (0.2 to 0.6)	14 (7.7 to 22.7)	0.7 (0.4 to 1.3)	20.5 (10.6 to 36.4)	0.6 (0.3 to 1.1)	21.1 (10.9 to 37.4)
Marshall Islands	Secondhand smoke	0.9 (0.2 to 1.6)	28.2 (6.1 to 50)	0.7 (0.2 to 1.4)	29 (6.3 to 51.4)	1.4 (0.3 to 2.7)	42.7 (8.5 to 87.2)	1.2 (0.2 to 2.5)	43.9 (8.8 to 89.5)
Marshall Islands	Metabolic risks	5.2 (1.9 to 9.4)	136.1 (51.4 to 249)	3.8 (1.4 to 7.3)	139.9 (53 to 255.8)	12.1 (4.6 to 22.4)	320.7 (117.7 to 613)	9.4 (3.4 to 17.9)	330.2 (121.1 to 631.2)
Marshall Islands	High body-mass index	2.7 (0.7 to 5.7)	71.3 (19.5 to 149.6)	2 (0.5 to 4.3)	73.3 (20.2 to 153.7)	6.1 (1.7 to 12.7)	158.5 (45.5 to 327.1)	4.7 (1.3 to 9.8)	163.2 (46.5 to 337)
Marshall Islands	High fasting plasma glucose	2.8 (0.6 to 6.3)	74.1 (15 to 169.2)	2.1 (0.4 to 4.9)	76.1 (15.4 to 174.7)	7.3 (1.7 to 16.8)	194.4 (43 to 458.6)	5.7 (1.3 to 13.5)	200.1 (44.3 to 470.6)
Mauritania	All risk factors	2.4 (1.3 to 3.8)	57.5 (30.5 to 91.6)	1.4 (0.7 to 2.4)	59 (31.2 to 93.9)	3.1 (1.7 to 5)	66.1 (33.4 to 109.5)	2.2 (1.1 to 3.9)	68.4 (34.5 to 113.3)
Mauritania	Behavioral risks	1.3 (0.7 to 1.9)	36.7 (20 to 56.5)	0.9 (0.5 to 1.4)	37.5 (20.5 to 57.7)	1.3 (0.8 to 2)	35.4 (19.7 to 53)	1.1 (0.6 to 1.8)	36.6 (20.4 to 54.5)
Mauritania	Alcohol use	0 (0 to 0)	0.4 (0 to 1.1)	0 (0 to 0)	0.4 (0 to 1.1)	0 (0 to 0)	0 (0 to 0)	0 (0 to 0)	0 (0 to 0)
Mauritania	Diet high in red meat	0.5 (0.1 to 0.8)	14.8 (4.3 to 24.2)	0.3 (0.1 to 0.6)	15.1 (4.4 to 24.8)	0.5 (0.1 to 0.8)	14 (4.2 to 22.8)	0.4 (0.1 to 0.8)	14.5 (4.3 to 23.6)
Mauritania	Low physical activity	0.3 (0.1 to 0.6)	7.4 (2.9 to 14.7)	0.2 (0.1 to 0.4)	7.6 (3 to 15.1)	0.4 (0.1 to 0.7)	8.3 (2.9 to 16)	0.3 (0.1 to 0.6)	8.6 (3 to 16.5)
Mauritania	Smoking	0.1 (0.1 to 0.2)	4.1 (1.8 to 7.5)	0.1 (0 to 0.2)	4.2 (1.8 to 7.7)	0.2 (0.1 to 0.3)	4.4 (1.8 to 7.8)	0.1 (0.1 to 0.3)	4.5 (1.9 to 8.1)
Mauritania	Secondhand smoke	0.4 (0.1 to 0.7)	11 (2.4 to 21.3)	0.2 (0.1 to 0.5)	11.2 (2.5 to 21.8)	0.3 (0.1 to 0.6)	9.7 (1.9 to 18.1)	0.3 (0.1 to 0.6)	10 (2 to 18.6)
Mauritania	Metabolic risks	1.2 (0.3 to 2.3)	22.6 (2.7 to 49)	0.6 (0.1 to 1.3)	23.2 (3 to 50.5)	1.9 (0.6 to 3.7)	33.2 (5.4 to 71.9)	1.2 (0.2 to 2.6)	34.4 (5.6 to 74.3)
Mauritania	High body-mass index	0.6 (0.1 to 1.3)	10 (-3.5 to 28.5)	0.3 (0 to 0.8)	10.4 (-3.5 to 29.1)	1 (0.2 to 2)	14 (-4.7 to 36.9)	0.5 (-0.1 to 1.4)	14.5 (-4.7 to 38.2)
Mauritania	High fasting plasma glucose	0.6 (0.1 to 1.4)	13.1 (2.3 to 32.5)	0.3 (0.1 to 0.9)	13.5 (2.4 to 33.3)	1 (0.2 to 2.5)	20.7 (3.9 to 52.3)	0.7 (0.1 to 1.8)	21.4 (4 to 53.8)
Mauritius	All risk factors	3.4 (2.1 to 4.9)	89.7 (56.5 to 127.4)	3.6 (2 to 5.7)	93.4 (58.5 to 133)	8.1 (4.7 to 12.1)	209.4 (122.3 to 311.5)	11.2 (5.9 to 17.7)	220.5 (129.7 to 327.6)
Mauritius	Behavioral risks	1.3 (0.8 to 1.6)	37.1 (24 to 49.2)	1.5 (0.9 to 2.3)	38.6 (25 to 51.2)	2.2 (1.4 to 3)	63.9 (39.8 to 89.8)	3.4 (1.9 to 5.4)	67.3 (41.9 to 94.8)
Mauritius	Alcohol use	0.4 (0.2 to 0.5)	11.2 (7.1 to 15.8)	0.4 (0.3 to 0.7)	11.7 (7.4 to 16.5)	0.6 (0.4 to 1)	19.4 (11 to 30)	1 (0.5 to 1.8)	20.5 (11.5 to 31.7)
Mauritius	Diet high in red meat	0.2 (0 to 0.3)	6.7 (1.2 to 9.1)	0.3 (0 to 0.4)	7 (1.3 to 9.5)	0.4 (0.1 to 0.6)	12.3 (2.5 to 19.2)	0.7 (0.1 to 1.1)	13 (2.6 to 20.2)
Mauritius	Low physical activity	0.1 (0.1 to 0.2)	2.4 (1.3 to 4.5)	0.1 (0 to 0.2)	2.5 (1.4 to 4.7)	0.2 (0.1 to 0.4)	4.9 (2.4 to 9.3)	0.3 (0.1 to 0.6)	5.1 (2.5 to 9.7)
Mauritius	Smoking	0.1 (0.1 to 0.2)	3.6 (1.9 to 5.7)	0.1 (0.1 to 0.2)	3.7 (2 to 6)	0.2 (0.1 to 0.3)	4.9 (2.7 to 7.8)	0.3 (0.1 to 0.5)	5.2 (2.9 to 8.2)
Mauritius	Secondhand smoke	0.5 (0.1 to 0.8)	14.8 (3.5 to 25.6)	0.6 (0.1 to 1.1)	15.4 (3.6 to 26.8)	0.8 (0.2 to 1.5)	24.8 (5.9 to 45)	1.3 (0.3 to 2.6)	26.1 (6.2 to 47.1)

Location	Risk factor	1990				2019			
		Deaths	YLLs (Years of Life Lost)	YLDs (Years Lived with Disability)	DALYs (Disability-Adjusted Life Years)	Deaths	YLLs (Years of Life Lost)	YLDs (Years Lived with Disability)	DALYs (Disability-Adjusted Life Years)
Mauritius	Metabolic risks	2.4 (1.1 to 4)	58.9 (25.9 to 99.9)	2.4 (1 to 4.3)	61.3 (26.9 to 104.2)	6.6 (3 to 10.7)	161.8 (72.9 to 266.4)	8.6 (3.8 to 14.7)	170.4 (77.1 to 280.5)
Mauritius	High body-mass index	1.6 (0.5 to 2.9)	40.7 (13.4 to 75)	1.7 (0.5 to 3.2)	42.4 (14.1 to 78.2)	4.3 (1.4 to 8)	108.1 (35.9 to 197.4)	5.7 (1.8 to 10.4)	113.8 (38 to 208.6)
Mauritius	High fasting plasma glucose	0.9 (0.2 to 2.1)	21.5 (4.1 to 49.2)	0.9 (0.2 to 2.1)	22.4 (4.3 to 50.8)	2.9 (0.6 to 6.4)	69.3 (14.6 to 153.9)	3.7 (0.8 to 8.9)	73 (15.4 to 161.6)
Mexico	All risk factors	3.2 (2.1 to 4.5)	77.3 (50.2 to 109.6)	3.2 (1.9 to 4.9)	80.5 (52.5 to 114.3)	3.3 (2 to 5.1)	82.7 (48.8 to 127.2)	5.3 (2.9 to 8.6)	88 (52.5 to 134.7)
Mexico	Behavioral risks	1.8 (1.4 to 2.1)	52.1 (40.2 to 63)	2 (1.3 to 2.8)	54.1 (41.7 to 65.4)	1.6 (1.2 to 2.1)	49.3 (36.3 to 65.2)	3 (1.9 to 4.3)	52.3 (38.7 to 68.8)
Mexico	Alcohol use	0.5 (0.4 to 0.7)	18.1 (13.9 to 22.5)	0.7 (0.4 to 1)	18.8 (14.4 to 23.3)	0.6 (0.5 to 0.9)	21.3 (15.4 to 28.6)	1.3 (0.8 to 1.8)	22.5 (16.4 to 30.1)
Mexico	Diet high in red meat	0.4 (0.2 to 0.6)	13 (5.5 to 16.9)	0.5 (0.2 to 0.8)	13.5 (5.7 to 17.6)	0.5 (0.2 to 0.7)	14.5 (6.1 to 21.5)	0.9 (0.4 to 1.4)	15.4 (6.5 to 22.5)
Mexico	Low physical activity	0.1 (0 to 0.2)	3 (1.3 to 6.4)	0.1 (0.1 to 0.3)	3.2 (1.3 to 6.6)	0.1 (0.1 to 0.2)	3 (1.3 to 6.4)	0.2 (0.1 to 0.4)	3.2 (1.3 to 6.7)
Mexico	Smoking	0.4 (0.3 to 0.6)	10.9 (6.4 to 16.1)	0.5 (0.2 to 0.7)	11.4 (6.7 to 16.8)	0.2 (0.1 to 0.3)	5.5 (2.8 to 8.7)	0.4 (0.2 to 0.6)	5.8 (3 to 9.2)
Mexico	Secondhand smoke	0.3 (0.1 to 0.6)	10.4 (2.6 to 17.9)	0.4 (0.1 to 0.7)	10.8 (2.6 to 18.6)	0.2 (0.1 to 0.4)	7.6 (1.6 to 13.6)	0.5 (0.1 to 0.9)	8.1 (1.8 to 14.4)
Mexico	Metabolic risks	1.6 (0.5 to 3.2)	29.8 (2.5 to 65.7)	1.4 (0.3 to 3)	31.2 (2.8 to 68.3)	1.9 (0.6 to 3.7)	38.4 (4.4 to 83)	2.7 (0.5 to 5.8)	41 (4.9 to 87.9)
Mexico	High body-mass index	0.4 (0 to 0.9)	1.8 (-10.4 to 13.3)	0.2 (-0.3 to 0.7)	2.1 (-10.6 to 13.9)	0.6 (0 to 1.3)	5.2 (-10.4 to 21.1)	0.5 (-0.4 to 1.5)	5.7 (-10.8 to 22.2)
Mexico	High fasting plasma glucose	1.3 (0.3 to 2.8)	29.5 (5.9 to 63.3)	1.3 (0.3 to 2.9)	30.8 (6.2 to 65.7)	1.5 (0.3 to 3.2)	35.4 (6.9 to 79.3)	2.3 (0.4 to 5.4)	37.7 (7.4 to 84.7)
Micronesia (Federated States of)	All risk factors	9.5 (5.5 to 14.5)	267.3 (156.1 to 406.2)	7.1 (3.8 to 11.3)	274.4 (160.6 to 416.6)	17 (8.6 to 30)	455.2 (224.9 to 803.7)	14.9 (7.3 to 26.6)	470.1 (233 to 824.6)
Micronesia (Federated States of)	Behavioral risks	3.7 (2.3 to 5.3)	110.4 (68.9 to 160.4)	2.9 (1.6 to 4.4)	113.3 (70.8 to 164.8)	5.5 (3 to 9.3)	154.7 (78.9 to 274.1)	5.1 (2.5 to 9.2)	159.7 (82.2 to 283.5)
Micronesia (Federated States of)	Alcohol use	0.2 (0.1 to 0.3)	5.8 (3 to 9.7)	0.1 (0.1 to 0.2)	5.9 (3.1 to 9.9)	0.2 (0.1 to 0.3)	6 (2.1 to 11.9)	0.2 (0.1 to 0.4)	6.2 (2.2 to 12.3)
Micronesia (Federated States of)	Diet high in red meat	0.7 (0.2 to 1.1)	21.8 (6 to 35.1)	0.6 (0.1 to 1)	22.3 (6.2 to 35.9)	1 (0.2 to 1.9)	30.1 (7.1 to 60.2)	1 (0.2 to 2)	31.1 (7.4 to 61.9)
Micronesia (Federated States of)	Low physical activity	0.5 (0.2 to 1)	13 (4.4 to 26.1)	0.4 (0.1 to 0.7)	13.4 (4.5 to 26.9)	0.9 (0.3 to 1.8)	20.7 (6.1 to 46.5)	0.7 (0.2 to 1.6)	21.4 (6.3 to 47.9)
Micronesia (Federated States of)	Smoking	1.4 (0.7 to 2.3)	41 (19.6 to 67.3)	1.1 (0.5 to 1.9)	42.1 (20.1 to 69.1)	2.2 (1 to 4)	59.2 (26.1 to 109.1)	2 (0.9 to 3.7)	61.2 (26.9 to 112.8)
Micronesia (Federated States of)	Secondhand smoke	1.1 (0.3 to 2)	34.6 (8 to 61.9)	0.9 (0.2 to 1.7)	35.4 (8.2 to 63.6)	1.5 (0.3 to 3.3)	46.5 (9 to 103.2)	1.5 (0.3 to 3.2)	48 (9.3 to 106.2)
Micronesia (Federated States of)	Metabolic risks	6.7 (2.7 to 11.7)	182.4 (73.4 to 313.3)	4.9 (1.9 to 8.8)	187.4 (75.4 to 321.6)	13.3 (5.4 to 24.9)	348.8 (140.2 to 657.1)	11.4 (4.6 to 21.5)	360.2 (144.8 to 679.5)
Micronesia (Federated States of)	High body-mass index	5.2 (1.7 to 9.7)	143.4 (48.7 to 262.3)	3.9 (1.3 to 7.3)	147.3 (49.9 to 269.5)	9.1 (2.8 to 17.9)	241.5 (74.5 to 464.2)	7.8 (2.5 to 15.9)	249.4 (76.9 to 476.9)
Micronesia (Federated States of)	High fasting plasma glucose	1.9 (0.3 to 4.5)	49.2 (8.6 to 118.9)	1.3 (0.2 to 3.5)	50.5 (8.8 to 121.9)	5.6 (1.1 to 14.3)	142.1 (27.8 to 368.5)	4.7 (0.9 to 11.9)	146.9 (28.6 to 379.9)
Monaco	All risk factors	8.8 (5.5 to 12.6)	221.4 (136.9 to 320.9)	21.7 (11.9 to 34.9)	243.1 (150.4 to 351.2)	10.1 (6.1 to 14.4)	238.3 (138.8 to 347.6)	32.7 (17.8 to 50.8)	271 (157.4 to 394.5)
Monaco	Behavioral risks	6.8 (4.2 to 9.7)	190.5 (115.6 to 274.5)	18.4 (10 to 29.7)	208.9 (126.5 to 301.1)	7 (4.1 to 10.2)	188 (107.2 to 281.4)	25.7 (13.9 to 41.1)	213.7 (122.1 to 320.6)
Monaco	Alcohol use	2.5 (0 to 4.7)	71.9 (1.1 to 137)	7 (0.1 to 14.5)	78.9 (1.2 to 149.8)	2.7 (0 to 5.2)	74.5 (0 to 144.5)	10.2 (0 to 21)	84.7 (0 to 162.7)
Monaco	Diet high in red meat	1.8 (0.8 to 2.7)	47.6 (22.8 to 73.5)	4.6 (2.1 to 7.9)	52.2 (24.9 to 80.8)	1.9 (0.9 to 2.9)	50.1 (23.2 to 78.1)	6.9 (3.1 to 11.7)	56.9 (27 to 89.4)
Monaco	Low physical activity	0.6 (0.2 to 1.1)	12.4 (3.8 to 24.8)	1.2 (0.4 to 2.8)	13.6 (4.2 to 27.5)	0.7 (0.2 to 1.3)	14.3 (4.6 to 27.5)	2 (0.6 to 4.2)	16.3 (5.2 to 31.9)
Monaco	Smoking	2.1 (1.3 to 3.1)	61.5 (38.3 to 88.5)	5.9 (3.2 to 9.5)	67.4 (41.8 to 98.2)	1.9 (1.2 to 2.9)	53.8 (32.8 to 79.5)	7.3 (4 to 12.4)	61.1 (37.5 to 90)
Monaco	Secondhand smoke	0.6 (0.1 to 1)	17.1 (3.9 to 31.3)	1.6 (0.3 to 3)	18.7 (4.2 to 34)	0.5 (0.1 to 0.8)	13.9 (3.2 to 25.7)	1.9 (0.4 to 3.8)	15.8 (3.5 to 29.2)
Monaco	Metabolic risks	2.5 (0.8 to 5.1)	40.4 (2.8 to 93.5)	4.3 (0.6 to 9.4)	44.7 (3.7 to 101.9)	3.9 (1.4 to 7.4)	65.3 (13.6 to 136)	9.1 (1.8 to 19)	74.4 (15.4 to 151.6)
Monaco	High body-mass index	1.5 (0.3 to 3.4)	19.4 (-9.8 to 56.9)	2.1 (-0.7 to 5.9)	21.5 (-10.6 to 62.5)	1.9 (0.4 to 4)	25.3 (-6.9 to 64.7)	3.5 (-1 to 8.7)	28.7 (-8.2 to 73.5)
Monaco	High fasting plasma glucose	1.1 (0.2 to 2.7)	22.4 (4 to 56.3)	2.3 (0.4 to 6.1)	24.8 (4.4 to 62.5)	2.1 (0.4 to 5.1)	43.1 (7.9 to 104.8)	6 (1.1 to 15.3)	49.2 (9.1 to 120)
Mongolia	All risk factors	1.5 (0.9 to 2.1)	43.6 (25.5 to 63.8)	1.4 (0.8 to 2.3)	45 (26.5 to 65.6)	2.1 (1.3 to 3.1)	55.7 (33.8 to 84)	2.3 (1.3 to 3.7)	58 (35 to 87.8)
Mongolia	Behavioral risks	1.2 (0.7 to 1.8)	38 (23.3 to 57)	1.2 (0.7 to 1.9)	39.2 (24 to 58.6)	1.5 (1 to 2.2)	46 (29 to 68.2)	1.9 (1 to 3.1)	47.8 (30.1 to 71.4)
Mongolia	Alcohol use	0.2 (0.1 to 0.3)	7.3 (4.1 to 11.2)	0.2 (0.1 to 0.3)	7.5 (4.2 to 11.6)	0.4 (0.3 to 0.7)	14.8 (8.9 to 22.5)	0.6 (0.3 to 0.9)	15.4 (9.2 to 23.3)
Mongolia	Diet high in red meat	0.6 (0.3 to 1.1)	19 (7.9 to 34.8)	0.6 (0.2 to 1.1)	19.6 (8.2 to 36)	0.7 (0.3 to 1.1)	19.4 (8.5 to 34.3)	0.8 (0.3 to 1.5)	20.2 (8.8 to 35.6)
Mongolia	Low physical activity	0.1 (0 to 0.1)	1.4 (0.8 to 2.8)	0 (0 to 0.1)	1.4 (0.9 to 2.9)	0.1 (0 to 0.1)	1.6 (0.9 to 3.2)	0.1 (0 to 0.1)	1.7 (0.9 to 3.4)
Mongolia	Smoking	0.1 (0 to 0.1)	2.4 (1.1 to 3.9)	0.1 (0 to 0.1)	2.4 (1.1 to 4)	0.1 (0.1 to 0.2)	3.1 (1.5 to 5.2)	0.1 (0.1 to 0.2)	3.3 (1.6 to 5.4)

Location	Risk factor	1990				2019			
		Deaths	YLLs (Years of Life Lost)	YLDs (Years Lived with Disability)	DALYs (Disability-Adjusted Life Years)	Deaths	YLLs (Years of Life Lost)	YLDs (Years Lived with Disability)	DALYs (Disability-Adjusted Life Years)
Mongolia	Secondhand smoke	0.3 (0.1 to 0.5)	9.9 (2.3 to 18.2)	0.3 (0.1 to 0.6)	10.2 (2.4 to 18.6)	0.3 (0.1 to 0.6)	10 (2.2 to 18.5)	0.4 (0.1 to 0.8)	10.4 (2.3 to 19.3)
Mongolia	Metabolic risks	0.4 (0.1 to 0.8)	6.4 (-2 to 16.7)	0.3 (0 to 0.7)	6.7 (-2 to 17.3)	0.7 (0.2 to 1.3)	11.2 (-0.6 to 26.9)	0.5 (0 to 1.2)	11.7 (-0.6 to 27.8)
Mongolia	High body-mass index	0.2 (0 to 0.5)	2 (-5 to 10.1)	0.1 (-0.1 to 0.4)	2.2 (-5.1 to 10.4)	0.4 (0 to 0.8)	4 (-4.8 to 13.8)	0.2 (-0.2 to 0.6)	4.2 (-5.1 to 14.4)
Mongolia	High fasting plasma glucose	0.2 (0 to 0.4)	4.5 (0.7 to 11.2)	0.2 (0 to 0.4)	4.7 (0.8 to 11.6)	0.3 (0.1 to 0.8)	7.6 (1.3 to 19.3)	0.3 (0.1 to 0.9)	7.9 (1.4 to 20)
Montenegro	All risk factors	7.7 (5.5 to 10.5)	203.6 (146.2 to 277.2)	12.9 (7.8 to 19.7)	216.5 (154.9 to 297.1)	9.4 (6.6 to 12.9)	239.8 (169.4 to 328)	19.7 (12.1 to 29.4)	259.4 (182 to 351)
Montenegro	Behavioral risks	5.7 (4.3 to 7.4)	166.7 (126 to 215.6)	10.4 (6.5 to 15.7)	177.1 (133.7 to 229.8)	6.4 (4.8 to 8.1)	181.2 (136 to 234.3)	14.9 (9.5 to 22)	196.1 (145.8 to 253.5)
Montenegro	Alcohol use	2.5 (1.7 to 3.5)	77 (49.9 to 108)	4.8 (2.7 to 7.4)	81.8 (53.1 to 114.5)	2.2 (1.5 to 3.1)	66.6 (45 to 92.6)	5.5 (3.2 to 8.4)	72.1 (48.7 to 100.1)
Montenegro	Diet high in red meat	1 (0.4 to 1.5)	29.8 (12.8 to 43.6)	1.9 (0.7 to 3)	31.7 (13.4 to 46.1)	1.2 (0.5 to 1.8)	34.4 (15.6 to 50.7)	2.8 (1.2 to 4.6)	37.3 (17.1 to 54.8)
Montenegro	Low physical activity	0.2 (0.1 to 0.4)	5.4 (2.9 to 9.7)	0.3 (0.2 to 0.7)	5.7 (3.1 to 10.3)	0.3 (0.2 to 0.5)	6.2 (3.3 to 10.7)	0.5 (0.3 to 1)	6.7 (3.6 to 11.6)
Montenegro	Smoking	2 (1.3 to 2.8)	57.8 (38.4 to 81.3)	3.6 (2 to 5.9)	61.4 (40.7 to 86.5)	2.6 (1.9 to 3.5)	75 (52.5 to 103.8)	6.2 (3.6 to 9.7)	81.2 (56.8 to 112.6)
Montenegro	Secondhand smoke	0.5 (0.1 to 0.9)	13.8 (3.2 to 24.7)	0.9 (0.2 to 1.6)	14.6 (3.4 to 26.1)	0.7 (0.2 to 1.2)	18.2 (4.2 to 33.1)	1.5 (0.3 to 2.9)	19.7 (4.5 to 35.8)
Montenegro	Metabolic risks	2.7 (1 to 5)	49.7 (8.1 to 103.4)	3.3 (0.6 to 6.9)	53 (8.9 to 109.9)	4 (1.5 to 7.6)	79.5 (22.5 to 162.9)	6.4 (1.6 to 13)	85.9 (24.6 to 175.7)
Montenegro	High body-mass index	1.3 (0.2 to 2.7)	19.6 (-7.2 to 51.2)	1.3 (-0.4 to 3.4)	20.9 (-7.5 to 53.9)	1.8 (0.4 to 3.7)	29.5 (-4 to 70.7)	2.3 (-0.5 to 5.7)	31.8 (-4.6 to 76.3)
Montenegro	High fasting plasma glucose	1.5 (0.3 to 3.4)	32.5 (5.8 to 77.9)	2.1 (0.4 to 5.2)	34.6 (6.2 to 82.4)	2.5 (0.5 to 5.5)	54.6 (10.4 to 126.8)	4.5 (0.8 to 10.9)	59.1 (11.3 to 136)
Morocco	All risk factors	2.2 (1.1 to 3.5)	60 (27 to 100.7)	1.8 (0.8 to 3.1)	61.8 (27.7 to 103.7)	4.1 (2 to 7)	105.9 (42.7 to 195.2)	4.4 (1.7 to 8.4)	110.3 (44.5 to 203.4)
Morocco	Behavioral risks	1.3 (0.7 to 1.8)	42.7 (24.1 to 63.4)	1.2 (0.6 to 1.9)	43.9 (24.8 to 65.2)	1.7 (0.9 to 2.6)	54.8 (29.5 to 85.2)	2.3 (1.2 to 3.7)	57.1 (30.8 to 88.9)
Morocco	Alcohol use	0 (0 to 0)	0.2 (0.1 to 0.3)	0 (0 to 0)	0.2 (0.1 to 0.3)	0 (0 to 0)	0.2 (0.1 to 0.3)	0 (0 to 0)	0.2 (0.1 to 0.3)
Morocco	Diet high in red meat	0.3 (0.1 to 0.5)	11.3 (2.1 to 17)	0.3 (0.1 to 0.5)	11.6 (2.2 to 17.5)	0.5 (0.1 to 0.9)	18 (3.8 to 30.3)	0.7 (0.2 to 1.3)	18.7 (3.9 to 31.6)
Morocco	Low physical activity	0.3 (0.1 to 0.5)	8.1 (3.4 to 15.5)	0.2 (0.1 to 0.5)	8.4 (3.5 to 15.9)	0.4 (0.2 to 0.8)	12.4 (4.6 to 25.3)	0.5 (0.2 to 1.1)	12.9 (4.8 to 26.5)
Morocco	Smoking	0.1 (0.1 to 0.1)	2.9 (1.5 to 4.7)	0.1 (0 to 0.1)	2.9 (1.6 to 4.8)	0.1 (0.1 to 0.2)	2.9 (1.5 to 4.8)	0.1 (0.1 to 0.2)	3 (1.5 to 5)
Morocco	Secondhand smoke	0.6 (0.2 to 1.1)	21.1 (5.4 to 37.5)	0.6 (0.1 to 1.1)	21.7 (5.5 to 38.5)	0.7 (0.1 to 1.2)	22.7 (5.2 to 43.3)	0.9 (0.2 to 1.9)	23.6 (5.4 to 45.1)
Morocco	Metabolic risks	1 (0.1 to 2.2)	18.7 (-10.1 to 55.1)	0.7 (-0.1 to 1.8)	19.4 (-10.1 to 56.3)	2.6 (0.5 to 5.5)	55.1 (-9 to 138.1)	2.3 (-0.4 to 5.9)	57.4 (-9.5 to 143.7)
Morocco	High body-mass index	0.2 (-0.3 to 0.8)	-3 (-22.5 to 12.9)	0 (-0.5 to 0.5)	-2.9 (-22.6 to 13.7)	0.7 (-0.4 to 1.9)	0.3 (-39 to 32.8)	0 (-1.7 to 1.4)	0.3 (-40.6 to 34.2)
Morocco	High fasting plasma glucose	0.8 (0.1 to 1.9)	22.3 (4 to 53.4)	0.7 (0.1 to 1.7)	22.9 (4.1 to 54.8)	2 (0.4 to 4.7)	57.6 (11 to 139.4)	2.5 (0.4 to 6.2)	60.1 (11.5 to 145.1)
Mozambique	All risk factors	1.4 (0.8 to 2.2)	32.7 (18.4 to 52.3)	0.8 (0.4 to 1.3)	33.5 (18.9 to 53.5)	2.8 (1.5 to 4.7)	65 (34 to 109.9)	1.7 (0.9 to 3)	66.8 (34.9 to 112.7)
Mozambique	Behavioral risks	0.8 (0.5 to 1.1)	22.3 (13.7 to 32.4)	0.5 (0.3 to 0.7)	22.7 (14 to 33.1)	1.5 (1 to 2.3)	43.7 (25.9 to 67.7)	1.1 (0.6 to 1.8)	44.8 (26.7 to 69.5)
Mozambique	Alcohol use	0.2 (0.1 to 0.4)	7.2 (3.3 to 12.2)	0.1 (0.1 to 0.3)	7.4 (3.3 to 12.4)	0.7 (0.4 to 1.1)	21.1 (10.7 to 35)	0.5 (0.3 to 0.9)	21.6 (11 to 35.9)
Mozambique	Diet high in red meat	0.1 (0 to 0.2)	3.2 (0.9 to 5)	0.1 (0 to 0.1)	3.3 (1 to 5.1)	0.2 (0.1 to 0.4)	5.8 (1.3 to 10.1)	0.1 (0 to 0.3)	5.9 (1.3 to 10.4)
Mozambique	Low physical activity	0.1 (0 to 0.2)	1.7 (1 to 4.2)	0 (0 to 0.1)	1.7 (1.1 to 4.3)	0.1 (0.1 to 0.2)	2.4 (1.4 to 5.6)	0.1 (0 to 0.1)	2.5 (1.4 to 5.8)
Mozambique	Smoking	0.1 (0.1 to 0.2)	3.3 (1.5 to 5.6)	0.1 (0 to 0.1)	3.4 (1.5 to 5.7)	0.2 (0.1 to 0.4)	4.5 (1.9 to 8)	0.1 (0 to 0.2)	4.6 (2 to 8.3)
Mozambique	Secondhand smoke	0.2 (0 to 0.4)	7.3 (1.5 to 13.7)	0.1 (0 to 0.3)	7.4 (1.5 to 14)	0.4 (0.1 to 0.7)	11 (2.2 to 21.3)	0.3 (0.1 to 0.5)	11.3 (2.2 to 21.9)
Mozambique	Metabolic risks	0.6 (0.1 to 1.4)	11 (0.4 to 28.1)	0.3 (0 to 0.7)	11.3 (0.4 to 28.8)	1.4 (0.3 to 3)	23 (0.5 to 59.3)	0.7 (0.1 to 1.7)	23.7 (0.7 to 60.9)
Mozambique	High body-mass index	0.1 (0 to 0.3)	-0.3 (-3.9 to 3.3)	0 (0 to 0.1)	-0.3 (-4 to 3.4)	0.3 (-0.1 to 0.9)	0.8 (-11.1 to 13.5)	0.1 (-0.2 to 0.4)	0.9 (-11.2 to 13.9)
Mozambique	High fasting plasma glucose	0.5 (0.1 to 1.3)	11.5 (2.1 to 27.9)	0.3 (0 to 0.7)	11.7 (2.2 to 28.6)	1.1 (0.2 to 2.6)	22.6 (3.9 to 56.7)	0.6 (0.1 to 1.6)	23.3 (4 to 58.1)
Myanmar	All risk factors	6 (3.4 to 9.9)	178.1 (93.6 to 297.2)	4.1 (2.1 to 6.8)	182.1 (95.9 to 303)	4.6 (2.7 to 7.1)	118.6 (69.4 to 185.5)	4.1 (2.2 to 6.7)	122.7 (71.6 to 191.9)
Myanmar	Behavioral risks	3.4 (2 to 5.1)	103.6 (56.9 to 162.5)	2.3 (1.3 to 3.8)	106 (58.6 to 166.1)	1.6 (1.1 to 2.3)	44.7 (28.2 to 63.8)	1.5 (0.9 to 2.4)	46.2 (29.2 to 65.8)
Myanmar	Alcohol use	0.1 (0 to 0.2)	2.8 (1.1 to 5.4)	0.1 (0 to 0.1)	2.9 (1.1 to 5.5)	0.2 (0.1 to 0.3)	6.2 (3.6 to 9.8)	0.2 (0.1 to 0.3)	6.4 (3.8 to 10.1)
Myanmar	Diet high in red meat	0.3 (0.1 to 0.5)	10 (2.6 to 17.1)	0.2 (0.1 to 0.4)	10.2 (2.7 to 17.5)	0.3 (0.1 to 0.5)	9.5 (1.9 to 14.8)	0.3 (0.1 to 0.5)	9.8 (2 to 15.4)
Myanmar	Low physical activity	0.2 (0.1 to 0.4)	5.6 (3.1 to 12.9)	0.1 (0.1 to 0.3)	5.7 (3.2 to 13.2)	0.1 (0.1 to 0.2)	2.7 (1.5 to 5.6)	0.1 (0 to 0.2)	2.8 (1.6 to 5.8)
Myanmar	Smoking	1.5 (0.9 to 2.4)	38.1 (20.4 to 60.2)	0.9 (0.5 to 1.6)	39 (21.1 to 61.7)	0.5 (0.3 to 0.8)	10.8 (6.4 to 16.4)	0.4 (0.2 to 0.7)	11.2 (6.6 to 17)

Location	Risk factor	1990				2019			
		Deaths	YLLs (Years of Life Lost)	YLDs (Years Lived with Disability)	DALYs (Disability-Adjusted Life Years)	Deaths	YLLs (Years of Life Lost)	YLDs (Years Lived with Disability)	DALYs (Disability-Adjusted Life Years)
Myanmar	Secondhand smoke	1.4 (0.3 to 2.7)	50 (10.6 to 100.6)	1.1 (0.2 to 2.1)	51.1 (10.9 to 103.3)	0.5 (0.1 to 1)	17.1 (3.9 to 32)	0.6 (0.1 to 1.1)	17.7 (4.1 to 33.1)
Myanmar	Metabolic risks	2.9 (0.9 to 6.3)	82.6 (24.3 to 180.1)	1.9 (0.5 to 4.1)	84.6 (24.8 to 184)	3.3 (1.3 to 5.7)	82 (33.1 to 143.5)	2.9 (1.2 to 5.2)	84.8 (34.4 to 148.2)
Myanmar	High body-mass index	1.2 (0.2 to 3.2)	34.4 (5.6 to 88.6)	0.8 (0.1 to 2.1)	35.2 (5.7 to 90.9)	2 (0.6 to 4)	51.8 (15 to 103.4)	1.8 (0.5 to 3.7)	53.6 (15.5 to 107.3)
Myanmar	High fasting plasma glucose	1.8 (0.3 to 4.5)	50.2 (9.4 to 126)	1.2 (0.2 to 3)	51.4 (9.6 to 129)	1.5 (0.3 to 3.3)	34.7 (6.4 to 79.2)	1.2 (0.2 to 3)	36 (6.6 to 81.7)
Namibia	All risk factors	3.6 (2.4 to 5.3)	95.4 (58.7 to 139.8)	2.4 (1.4 to 3.8)	97.8 (60.4 to 143.2)	7.7 (4.7 to 11.5)	195.5 (115.7 to 299.7)	6.6 (3.5 to 10.6)	202.2 (121.3 to 309.8)
Namibia	Behavioral risks	2.5 (1.7 to 3.4)	73.3 (47 to 103.2)	1.8 (1 to 2.6)	75.1 (48 to 105.4)	4.9 (3.2 to 7.2)	142 (88.3 to 215.3)	4.7 (2.6 to 7.5)	146.7 (91.4 to 221.2)
Namibia	Alcohol use	0.9 (0.5 to 1.3)	28.4 (16 to 43.6)	0.7 (0.3 to 1.1)	29.1 (16.3 to 44.5)	2.4 (1.5 to 3.8)	77.7 (46.5 to 121.9)	2.5 (1.3 to 4.3)	80.2 (48.2 to 125.6)
Namibia	Diet high in red meat	0.6 (0.2 to 0.9)	17.8 (6 to 27)	0.4 (0.1 to 0.7)	18.2 (6.2 to 27.7)	0.9 (0.3 to 1.5)	27 (8.2 to 47)	0.9 (0.3 to 1.6)	27.9 (8.5 to 48.4)
Namibia	Low physical activity	0.2 (0.1 to 0.3)	3.8 (1.9 to 7)	0.1 (0 to 0.2)	3.9 (2 to 7.2)	0.3 (0.1 to 0.6)	7.3 (3.2 to 14.2)	0.3 (0.1 to 0.5)	7.5 (3.4 to 14.9)
Namibia	Smoking	0.6 (0.4 to 0.9)	13.6 (7.1 to 21.3)	0.4 (0.2 to 0.6)	14 (7.3 to 22)	0.9 (0.5 to 1.3)	17.5 (9.5 to 28.1)	0.6 (0.3 to 1.1)	18.2 (9.8 to 29.2)
Namibia	Secondhand smoke	0.4 (0.1 to 0.8)	14 (3.3 to 25.9)	0.3 (0.1 to 0.6)	14.3 (3.4 to 26.4)	0.7 (0.1 to 1.3)	20.9 (4.5 to 41)	0.7 (0.1 to 1.4)	21.6 (4.6 to 42.3)
Namibia	Metabolic risks	1.3 (0.4 to 2.7)	26.1 (1.7 to 59.5)	0.8 (0.1 to 1.7)	26.8 (1.9 to 61.2)	3.3 (1 to 6.5)	63.8 (10.4 to 137.1)	2.3 (0.4 to 5)	66.1 (11 to 141.5)
Namibia	High body-mass index	0.4 (0 to 1)	5 (-8.9 to 18.4)	0.2 (-0.1 to 0.6)	5.2 (-9.1 to 18.9)	1.3 (0.1 to 2.7)	17.2 (-14.3 to 49.1)	0.7 (-0.4 to 1.8)	17.8 (-14.7 to 50.9)
Namibia	High fasting plasma glucose	0.9 (0.2 to 2.2)	21.9 (3.9 to 51.5)	0.6 (0.1 to 1.5)	22.5 (4 to 52.9)	2.1 (0.4 to 5.1)	49.5 (8.8 to 118.8)	1.7 (0.3 to 4.3)	51.3 (9.1 to 123.2)
Nauru	All risk factors	11.4 (6 to 17.9)	307.7 (164.8 to 480)	10.4 (5.5 to 17.1)	318.1 (171.4 to 496)	16.7 (9.2 to 25.3)	446.1 (238.7 to 689.9)	18.1 (9.5 to 28.7)	464.2 (250.2 to 713.4)
Nauru	Behavioral risks	4.8 (2.7 to 7.2)	144.4 (82.1 to 218.5)	4.8 (2.6 to 7.7)	149.2 (84.7 to 226.2)	6.5 (4 to 9.7)	195.1 (116.3 to 292.2)	7.9 (4.3 to 12.6)	203 (120.4 to 304.3)
Nauru	Alcohol use	0.4 (0.2 to 0.8)	16 (7.6 to 28.1)	0.5 (0.2 to 0.9)	16.5 (7.8 to 29)	0.9 (0.4 to 1.4)	30.7 (14.8 to 52.4)	1.2 (0.5 to 2.2)	31.9 (15.4 to 54.7)
Nauru	Diet high in red meat	1 (0.3 to 1.6)	29.6 (9.1 to 49.8)	1 (0.3 to 1.7)	30.6 (9.4 to 51.3)	1.2 (0.3 to 1.9)	34.9 (9.6 to 59.3)	1.4 (0.4 to 2.5)	36.3 (9.9 to 61.3)
Nauru	Low physical activity	0.5 (0.2 to 1)	11.4 (4 to 23.1)	0.4 (0.1 to 0.8)	11.8 (4.1 to 23.9)	0.7 (0.3 to 1.4)	16.5 (6 to 34.9)	0.7 (0.2 to 1.5)	17.2 (6.2 to 36.4)
Nauru	Smoking	2 (0.9 to 3.3)	57.8 (27.1 to 97.1)	1.9 (0.8 to 3.5)	59.8 (28.1 to 100.3)	2.6 (1.4 to 4.3)	77.3 (38.8 to 128.9)	3.1 (1.4 to 5.5)	80.5 (40.3 to 133.9)
Nauru	Secondhand smoke	1.3 (0.3 to 2.4)	38.9 (8.3 to 76)	1.3 (0.3 to 2.5)	40.2 (8.5 to 78)	1.6 (0.3 to 3.1)	48.9 (9.9 to 94.6)	2 (0.4 to 3.9)	50.8 (10.4 to 98.7)
Nauru	Metabolic risks	7.8 (3 to 14.1)	196.7 (76.9 to 347.3)	6.8 (2.5 to 12.2)	203.4 (79.3 to 362.4)	12.1 (4.7 to 20.7)	303 (117.3 to 531.7)	12.4 (4.7 to 22.3)	315.4 (123 to 551.7)
Nauru	High body-mass index	6.1 (2.1 to 12.1)	154.7 (51.5 to 304)	5.3 (1.7 to 10.5)	160 (53.5 to 313.6)	8.4 (2.5 to 15.6)	208.1 (61.9 to 385.1)	8.5 (2.4 to 16.3)	216.6 (64.7 to 400.4)
Nauru	High fasting plasma glucose	2.1 (0.4 to 5.2)	53.6 (8.8 to 131.9)	1.9 (0.3 to 4.9)	55.4 (9.1 to 136.8)	4.8 (1 to 11)	121.7 (24 to 284.3)	5 (1 to 12.6)	126.7 (25.3 to 295.4)
Nepal	All risk factors	1.9 (1 to 3)	52.9 (28.1 to 84.4)	1.2 (0.6 to 2)	54.1 (28.9 to 86.4)	3.3 (1.9 to 5.2)	87.4 (50.1 to 140.5)	2.8 (1.5 to 4.9)	90.2 (51.6 to 145)
Nepal	Behavioral risks	1.4 (0.8 to 2.1)	39.6 (22.3 to 59.5)	0.9 (0.5 to 1.5)	40.5 (22.8 to 60.8)	1.9 (1.2 to 2.8)	55.2 (34.5 to 80.6)	1.7 (1 to 2.7)	56.9 (35.7 to 83.3)
Nepal	Alcohol use	0 (0 to 0.1)	1.7 (0.5 to 3.3)	0 (0 to 0.1)	1.7 (0.5 to 3.4)	0.4 (0.2 to 0.6)	13.6 (7.1 to 21.9)	0.4 (0.2 to 0.7)	14 (7.3 to 22.7)
Nepal	Diet high in red meat	0.2 (0 to 0.4)	7.7 (1.4 to 13.2)	0.2 (0 to 0.3)	7.8 (1.4 to 13.4)	0.3 (0.1 to 0.6)	10.2 (2 to 17.1)	0.3 (0.1 to 0.6)	10.6 (2 to 17.6)
Nepal	Low physical activity	0.1 (0.1 to 0.2)	3.1 (1.5 to 6)	0.1 (0 to 0.2)	3.2 (1.5 to 6.2)	0.2 (0.1 to 0.3)	4.5 (2.3 to 8.3)	0.1 (0.1 to 0.3)	4.6 (2.4 to 8.6)
Nepal	Smoking	0.6 (0.3 to 1.1)	16.5 (6.9 to 28.4)	0.4 (0.2 to 0.7)	16.9 (7.1 to 29)	0.7 (0.3 to 1.1)	16.3 (7.5 to 26.8)	0.5 (0.2 to 0.9)	16.8 (7.8 to 27.7)
Nepal	Secondhand smoke	0.4 (0.1 to 0.7)	11.9 (2.7 to 22.7)	0.3 (0.1 to 0.5)	12.2 (2.7 to 23.2)	0.4 (0.1 to 0.8)	12.9 (2.8 to 24.2)	0.4 (0.1 to 0.8)	13.3 (2.9 to 25)
Nepal	Metabolic risks	0.6 (0.1 to 1.3)	14.9 (3 to 35.1)	0.4 (0.1 to 0.9)	15.2 (3 to 35.9)	1.5 (0.4 to 3.3)	36.1 (7.5 to 82.3)	1.2 (0.3 to 2.8)	37.3 (7.8 to 84.9)
Nepal	High body-mass index	0.1 (0 to 0.3)	1.5 (-0.7 to 5.8)	0 (0 to 0.2)	1.5 (-0.7 to 5.9)	0.4 (0 to 0.9)	5.9 (-3.6 to 19.2)	0.2 (-0.1 to 0.6)	6.1 (-3.7 to 19.8)
Nepal	High fasting plasma glucose	0.5 (0.1 to 1.2)	13.5 (2.2 to 33.4)	0.3 (0.1 to 0.8)	13.8 (2.3 to 34.1)	1.2 (0.2 to 2.9)	31 (5.6 to 75.2)	1 (0.2 to 2.5)	32 (5.8 to 78)
Netherlands	All risk factors	12 (10 to 14.1)	315.8 (268 to 363.3)	28.3 (19 to 38.6)	344.1 (292.1 to 396.3)	8.3 (6.6 to 10.2)	191.3 (155 to 230.5)	27.8 (17.9 to 42.2)	219.1 (176.7 to 265.3)
Netherlands	Behavioral risks	10 (8.6 to 11.4)	280.8 (242 to 317.7)	24.9 (16.8 to 34.4)	305.8 (261.9 to 347.4)	6.4 (5.5 to 7.5)	161.5 (137.1 to 186.5)	23.5 (14.8 to 35.1)	184.9 (155.6 to 215.5)
Netherlands	Alcohol use	4.9 (4 to 5.9)	137.8 (112.8 to 163.8)	12.2 (8 to 17.3)	150 (122.8 to 178.7)	3.5 (2.7 to 4.2)	87.5 (69.7 to 105.6)	12.7 (7.8 to 19.4)	100.2 (80.3 to 122)

Location	Risk factor	1990				2019			
		Deaths	YLLs (Years of Life Lost)	YLDs (Years Lived with Disability)	DALYs (Disability-Adjusted Life Years)	Deaths	YLLs (Years of Life Lost)	YLDs (Years Lived with Disability)	DALYs (Disability-Adjusted Life Years)
Netherlands	Diet high in red meat	1.7 (0.9 to 2.3)	47.8 (24.5 to 64.1)	4.2 (1.9 to 6.7)	52.1 (26.4 to 70.2)	1.3 (0.6 to 1.7)	32.1 (16.3 to 44)	4.6 (2.1 to 7.8)	36.8 (19.1 to 50.8)
Netherlands	Low physical activity	0.2 (0.1 to 0.5)	5 (2.7 to 10.1)	0.5 (0.2 to 1)	5.5 (2.9 to 11.1)	0.2 (0.1 to 0.4)	3.9 (1.9 to 7.6)	0.6 (0.3 to 1.2)	4.4 (2.2 to 8.7)
Netherlands	Smoking	3.8 (2.8 to 4.9)	106.8 (79 to 138)	9.5 (5.7 to 14.2)	116.3 (85.5 to 149.2)	1.9 (1.4 to 2.5)	46.4 (33.1 to 60.1)	6.8 (3.9 to 10.8)	53.2 (37.8 to 69.4)
Netherlands	Secondhand smoke	0.6 (0.1 to 1.1)	20.1 (4.5 to 35.1)	1.7 (0.4 to 3.3)	21.8 (5 to 38)	0.3 (0.1 to 0.5)	9 (2.2 to 15.8)	1.3 (0.3 to 2.5)	10.3 (2.5 to 18)
Netherlands	Metabolic risks	2.8 (1 to 5.1)	50.7 (12.6 to 101.5)	4.9 (1.3 to 9.7)	55.6 (13.8 to 110.4)	2.5 (0.9 to 4.6)	42.1 (10.4 to 84.6)	6.2 (1.5 to 13)	48.3 (11.8 to 95.9)
Netherlands	High body-mass index	1.4 (0.3 to 2.8)	21.7 (-2.1 to 51.6)	2.1 (-0.1 to 4.8)	23.7 (-2.1 to 56.2)	1.2 (0.3 to 2.6)	17.1 (-2.2 to 43.4)	2.5 (-0.2 to 6.3)	19.6 (-2.3 to 49.8)
Netherlands	High fasting plasma glucose	1.5 (0.3 to 3.4)	30.7 (5.9 to 70.2)	2.9 (0.5 to 7.2)	33.7 (6.4 to 77)	1.4 (0.3 to 3.2)	26.5 (5.1 to 61.7)	3.9 (0.7 to 10.1)	30.5 (5.9 to 70.7)
New Zealand	All risk factors	11.3 (9.4 to 13.2)	308.8 (257 to 357)	28.6 (19.3 to 39.9)	337.4 (281.9 to 392.2)	6.5 (5.2 to 8.1)	166 (132.8 to 199.2)	25.3 (15.5 to 37.2)	191.3 (151.3 to 231.3)
New Zealand	Behavioral risks	9.7 (8.3 to 11)	280.1 (238 to 319.6)	25.4 (16.9 to 35.1)	305.4 (258.9 to 349.9)	5.1 (4.3 to 6)	142.1 (120.3 to 165.2)	21 (13.3 to 30.7)	163.1 (136.2 to 190.5)
New Zealand	Alcohol use	5.1 (4.1 to 6.3)	151.9 (123.7 to 185.1)	13.6 (9.1 to 19.3)	165.5 (134 to 201.3)	2.8 (2.2 to 3.4)	79.1 (63.6 to 95.3)	11.6 (7.2 to 17.3)	90.7 (73 to 110.4)
New Zealand	Diet high in red meat	2 (1 to 2.9)	57.2 (28.1 to 83.2)	5.2 (2.4 to 8.5)	62.4 (30.4 to 91.2)	1.2 (0.6 to 1.7)	32.4 (15.7 to 46.6)	4.8 (2.2 to 8.1)	37.2 (18.3 to 53.5)
New Zealand	Low physical activity	0.4 (0.1 to 0.7)	8.8 (3.6 to 17.9)	0.9 (0.3 to 2)	9.6 (3.9 to 19.8)	0.3 (0.1 to 0.6)	7 (2.3 to 14)	1.1 (0.4 to 2.4)	8.1 (2.7 to 16.3)
New Zealand	Smoking	2.9 (2.1 to 3.7)	80.3 (57.8 to 104.3)	7.4 (4.4 to 11.1)	87.7 (63.2 to 114)	1.1 (0.8 to 1.5)	30.3 (20.8 to 40.6)	4.6 (2.5 to 7.4)	34.9 (23.7 to 47)
New Zealand	Secondhand smoke	0.5 (0.1 to 0.9)	16.6 (4.1 to 28.6)	1.4 (0.3 to 2.6)	17.9 (4.4 to 31.1)	0.3 (0.1 to 0.4)	8.2 (1.8 to 14.7)	1.1 (0.2 to 2.2)	9.4 (2.1 to 16.4)
New Zealand	Metabolic risks	2.3 (0.7 to 4.2)	42.1 (5.5 to 88.6)	4.6 (1.1 to 9.2)	46.8 (6.7 to 97.9)	1.9 (0.7 to 3.6)	33 (3.8 to 69)	5.9 (1.3 to 12.2)	38.9 (5.7 to 80.1)
New Zealand	High body-mass index	1.2 (0.2 to 2.6)	17.9 (-8.4 to 48.1)	2.3 (-0.2 to 5.5)	20.2 (-8.7 to 53.3)	1 (0.1 to 2)	12.4 (-7.6 to 33.9)	2.6 (-0.3 to 6.2)	15 (-7.9 to 39.2)
New Zealand	High fasting plasma glucose	1.1 (0.2 to 2.5)	25.4 (4.7 to 58.4)	2.5 (0.4 to 6.2)	27.9 (5.2 to 64.3)	1 (0.2 to 2.3)	21.8 (4 to 50.3)	3.5 (0.6 to 8.8)	25.4 (4.9 to 58.6)
Nicaragua	All risk factors	1.1 (0.6 to 1.7)	28.2 (14.7 to 45.6)	1.1 (0.5 to 2)	29.3 (15.3 to 47.4)	2.4 (1.3 to 4)	53.9 (28.5 to 91.1)	3.5 (1.6 to 6.2)	57.3 (30.3 to 96.5)
Nicaragua	Behavioral risks	0.5 (0.3 to 0.7)	16.4 (10.8 to 22)	0.6 (0.3 to 0.9)	17 (11.2 to 22.9)	0.8 (0.5 to 1)	20.8 (13.6 to 29)	1.3 (0.7 to 1.9)	22 (14.5 to 30.6)
Nicaragua	Alcohol use	0.1 (0.1 to 0.1)	3.5 (2.4 to 4.8)	0.1 (0.1 to 0.2)	3.6 (2.5 to 4.9)	0.2 (0.1 to 0.3)	5.8 (4 to 8.1)	0.3 (0.2 to 0.5)	6.2 (4.3 to 8.6)
Nicaragua	Diet high in red meat	0.1 (0 to 0.2)	3.4 (0.7 to 4.9)	0.1 (0 to 0.2)	3.5 (0.7 to 5.1)	0.1 (0 to 0.2)	3.8 (0.8 to 6)	0.2 (0.1 to 0.4)	4 (0.8 to 6.3)
Nicaragua	Low physical activity	0 (0 to 0.1)	1 (0.6 to 2.2)	0 (0 to 0.1)	1 (0.6 to 2.3)	0.1 (0 to 0.1)	1.6 (0.9 to 3.4)	0.1 (0 to 0.2)	1.7 (0.9 to 3.6)
Nicaragua	Smoking	0.1 (0.1 to 0.2)	3.8 (2 to 6)	0.1 (0.1 to 0.2)	3.9 (2.1 to 6.2)	0.1 (0.1 to 0.2)	3.8 (1.9 to 5.9)	0.2 (0.1 to 0.4)	4 (2 to 6.3)
Nicaragua	Secondhand smoke	0.2 (0 to 0.3)	5.2 (1.2 to 9.1)	0.2 (0 to 0.3)	5.4 (1.3 to 9.4)	0.2 (0.1 to 0.4)	6.4 (1.4 to 11.5)	0.4 (0.1 to 0.7)	6.7 (1.5 to 12.1)
Nicaragua	Metabolic risks	0.6 (0.2 to 1.2)	12.6 (0.4 to 29.1)	0.6 (0.1 to 1.3)	13.2 (0.5 to 30.2)	1.8 (0.7 to 3.4)	35.3 (9.7 to 72.3)	2.3 (0.7 to 5)	37.6 (10.5 to 76.8)
Nicaragua	High body-mass index	0.2 (0 to 0.4)	1.2 (-5.2 to 7.2)	0.1 (-0.1 to 0.4)	1.3 (-5.3 to 7.5)	0.6 (0.1 to 1.3)	8.9 (-2.8 to 22.4)	0.7 (-0.1 to 1.6)	9.6 (-2.9 to 24.1)
Nicaragua	High fasting plasma glucose	0.5 (0.1 to 1.1)	11.9 (2.2 to 26.7)	0.5 (0.1 to 1.2)	12.4 (2.3 to 27.9)	1.3 (0.3 to 2.8)	28.1 (5.6 to 63.9)	1.8 (0.3 to 4.3)	29.9 (5.9 to 68.7)
Niger	All risk factors	0.9 (0.5 to 1.5)	24.6 (14 to 38.4)	0.6 (0.3 to 1)	25.2 (14.3 to 39.3)	1.4 (0.7 to 2.5)	33.7 (16.8 to 60)	0.9 (0.4 to 1.7)	34.6 (17.3 to 61.3)
Niger	Behavioral risks	0.6 (0.3 to 0.9)	18 (9.7 to 27.4)	0.4 (0.2 to 0.6)	18.4 (10 to 28.1)	0.7 (0.4 to 1.1)	19.5 (10.2 to 31.4)	0.5 (0.2 to 0.9)	20 (10.5 to 32.2)
Niger	Alcohol use	0.1 (0 to 0.2)	2.9 (1.3 to 5)	0.1 (0 to 0.1)	2.9 (1.3 to 5.1)	0.1 (0 to 0.2)	3.3 (1.4 to 6.1)	0.1 (0 to 0.2)	3.3 (1.5 to 6.2)
Niger	Diet high in red meat	0.3 (0.1 to 0.4)	8 (2.1 to 12.6)	0.2 (0 to 0.3)	8.1 (2.1 to 12.9)	0.3 (0.1 to 0.5)	8.5 (2.1 to 14.8)	0.2 (0.1 to 0.4)	8.7 (2.2 to 15.2)
Niger	Low physical activity	0.1 (0 to 0.2)	2 (1 to 3.7)	0 (0 to 0.1)	2 (1 to 3.8)	0.1 (0 to 0.2)	2.2 (1 to 4.4)	0.1 (0 to 0.1)	2.3 (1.1 to 4.5)
Niger	Smoking	0 (0 to 0)	0.5 (0.2 to 0.9)	0 (0 to 0)	0.5 (0.2 to 0.9)	0 (0 to 0)	0.7 (0.3 to 1.3)	0 (0 to 0)	0.7 (0.3 to 1.4)
Niger	Secondhand smoke	0.2 (0 to 0.3)	5.1 (1.2 to 9.5)	0.1 (0 to 0.2)	5.2 (1.2 to 9.8)	0.2 (0 to 0.3)	5.3 (1.2 to 10.4)	0.1 (0 to 0.3)	5.5 (1.3 to 10.7)
Niger	Metabolic risks	0.4 (0.1 to 0.8)	7 (0.7 to 17)	0.2 (0 to 0.4)	7.2 (0.7 to 17.4)	0.8 (0.2 to 1.7)	15.1 (3 to 35.3)	0.5 (0.1 to 1)	15.6 (3.1 to 36.1)
Niger	High body-mass index	0.1 (0 to 0.4)	2.3 (-1.3 to 7.5)	0.1 (0 to 0.2)	2.3 (-1.3 to 7.7)	0.3 (0 to 0.6)	4.1 (-1.7 to 11.8)	0.1 (0 to 0.4)	4.3 (-1.7 to 12.1)
Niger	High fasting plasma glucose	0.2 (0 to 0.6)	4.9 (0.8 to 12.3)	0.1 (0 to 0.3)	5 (0.8 to 12.7)	0.5 (0.1 to 1.4)	11.3 (1.9 to 30)	0.3 (0.1 to 0.9)	11.7 (2 to 31)
Nigeria	All risk factors	2.4 (1.3 to 3.8)	59 (33.1 to 94.2)	1.5 (0.8 to 2.6)	60.6 (34.4 to 96.6)	4 (2.3 to 6.4)	92.1 (50.4 to 148.3)	2.9 (1.5 to 4.8)	95 (52 to 152.8)
Nigeria	Behavioral risks	1.4 (0.9 to 2.1)	40 (25.7 to 60.6)	1 (0.6 to 1.6)	41 (26.5 to 61.9)	2.1 (1.4 to 3)	55.9 (34.9 to 82.5)	1.7 (1 to 2.7)	57.6 (36.5 to 84.8)

Location	Risk factor	1990				2019			
		Deaths	YLLs (Years of Life Lost)	YLDs (Years Lived with Disability)	DALYs (Disability-Adjusted Life Years)	Deaths	YLLs (Years of Life Lost)	YLDs (Years Lived with Disability)	DALYs (Disability-Adjusted Life Years)
Nigeria	Alcohol use	0.7 (0.4 to 1.1)	19.9 (11.3 to 32.5)	0.5 (0.2 to 0.9)	20.4 (11.6 to 33.3)	1.1 (0.7 to 1.8)	30.6 (17.9 to 47.3)	1 (0.5 to 1.6)	31.6 (18.7 to 48.8)
Nigeria	Diet high in red meat	0.2 (0 to 0.4)	6.3 (1.3 to 10.6)	0.2 (0 to 0.3)	6.5 (1.4 to 10.9)	0.3 (0.1 to 0.6)	9.8 (2 to 17)	0.3 (0.1 to 0.5)	10.1 (2.1 to 17.3)
Nigeria	Low physical activity	0.2 (0.1 to 0.3)	4 (2 to 7.7)	0.1 (0 to 0.2)	4.2 (2.1 to 7.9)	0.3 (0.1 to 0.5)	5.9 (2.7 to 11.3)	0.2 (0.1 to 0.4)	6.1 (2.8 to 11.6)
Nigeria	Smoking	0.1 (0.1 to 0.2)	2.7 (1.2 to 4.7)	0.1 (0 to 0.1)	2.8 (1.3 to 4.9)	0.1 (0.1 to 0.2)	2.5 (1.2 to 4.5)	0.1 (0 to 0.1)	2.6 (1.3 to 4.7)
Nigeria	Secondhand smoke	0.3 (0.1 to 0.5)	8.1 (1.9 to 15.4)	0.2 (0 to 0.4)	8.3 (1.9 to 15.7)	0.3 (0.1 to 0.5)	8.5 (1.8 to 16.2)	0.2 (0 to 0.5)	8.7 (1.8 to 16.6)
Nigeria	Metabolic risks	1 (0.3 to 2.2)	20.6 (3.8 to 47.2)	0.6 (0.1 to 1.3)	21.2 (3.9 to 48.3)	2.1 (0.7 to 4.2)	39.5 (8.6 to 86.5)	1.3 (0.3 to 2.9)	40.8 (9.1 to 88.7)
Nigeria	High body-mass index	0.3 (0 to 0.9)	5.5 (-2.4 to 16.6)	0.2 (0 to 0.5)	5.7 (-2.4 to 17.1)	0.9 (0.1 to 2)	13.7 (-5.4 to 37.4)	0.5 (-0.1 to 1.2)	14.1 (-5.5 to 38.4)
Nigeria	High fasting plasma glucose	0.7 (0.1 to 1.7)	15.4 (2.7 to 39.5)	0.4 (0.1 to 1.1)	15.9 (2.8 to 40.3)	1.3 (0.2 to 3.1)	27.1 (4.6 to 65.8)	0.9 (0.1 to 2.2)	28 (4.7 to 67.9)
Niue	All risk factors	9 (5.1 to 13.6)	236.3 (137.2 to 358.7)	8.9 (4.9 to 14.2)	245.2 (142.7 to 370.7)	13.3 (7 to 21.1)	337.8 (182.7 to 533.8)	17.5 (9.1 to 28.6)	355.3 (194 to 561.3)
Niue	Behavioral risks	3.1 (2 to 4.6)	93.2 (55.8 to 139.8)	3.5 (1.9 to 5.4)	96.7 (57.9 to 145.6)	3.8 (2.2 to 5.5)	109.2 (62.6 to 162.7)	5.7 (3.1 to 9)	114.9 (66.5 to 170.5)
Niue	Alcohol use	0.4 (0.1 to 0.7)	14.6 (5.1 to 26.2)	0.5 (0.2 to 1)	15.1 (5.2 to 27.2)	0.5 (0.1 to 1)	19 (4.9 to 35.8)	1 (0.3 to 2)	20 (5.2 to 37.7)
Niue	Diet high in red meat	0.7 (0.2 to 1.1)	20.7 (6.5 to 34.2)	0.8 (0.2 to 1.3)	21.5 (6.7 to 35.3)	0.9 (0.3 to 1.4)	25.1 (7.6 to 42.3)	1.3 (0.4 to 2.4)	26.4 (8.1 to 44.5)
Niue	Low physical activity	0.4 (0.2 to 0.8)	9.1 (3.4 to 18.9)	0.3 (0.1 to 0.7)	9.4 (3.5 to 19.6)	0.6 (0.2 to 1.1)	13 (4.4 to 26.5)	0.7 (0.2 to 1.4)	13.7 (4.6 to 28.1)
Niue	Smoking	0.8 (0.4 to 1.3)	23.5 (11.9 to 37.6)	0.9 (0.4 to 1.5)	24.3 (12.4 to 39.2)	0.9 (0.5 to 1.4)	24.2 (12.6 to 39)	1.3 (0.6 to 2.1)	25.4 (13.4 to 41.3)
Niue	Secondhand smoke	1 (0.2 to 1.9)	30.5 (6.8 to 57.5)	1.1 (0.2 to 2.2)	31.6 (7 to 59.7)	1.2 (0.3 to 2.2)	34 (7.5 to 65.6)	1.8 (0.4 to 3.5)	35.7 (7.9 to 68.8)
Niue	Metabolic risks	6.7 (2.8 to 11.5)	165.1 (70.3 to 284.1)	6.3 (2.6 to 11)	171.4 (73.3 to 295.4)	10.9 (4.7 to 18.5)	263.4 (112.3 to 458)	13.6 (6 to 24.4)	277 (118.6 to 478.7)
Niue	High body-mass index	4.8 (1.6 to 9.1)	118.1 (39.7 to 221.5)	4.5 (1.5 to 8.4)	122.6 (41.3 to 229.7)	7.3 (2.3 to 13.9)	172.6 (55 to 324.2)	8.8 (2.7 to 16.8)	181.4 (58.6 to 338.4)
Niue	High fasting plasma glucose	2.5 (0.5 to 5.7)	60 (11.3 to 140.3)	2.3 (0.4 to 5.7)	62.3 (11.7 to 145.4)	5 (1.1 to 11.4)	122.2 (26.6 to 284.2)	6.4 (1.3 to 15.5)	128.6 (27.6 to 297.6)
North Macedonia	All risk factors	6.2 (4.5 to 8.1)	171.8 (125.1 to 222.8)	7.9 (4.9 to 11.6)	179.7 (132.1 to 232.3)	7.9 (5 to 11.9)	186 (118.3 to 278.6)	13 (7.4 to 21.2)	199 (126.9 to 298.2)
North Macedonia	Behavioral risks	4.4 (3.5 to 5.3)	138.1 (107.6 to 168.9)	6.1 (3.8 to 8.7)	144.2 (112.2 to 176.5)	4.3 (3.1 to 5.8)	118.9 (82.4 to 160.9)	8.4 (5 to 12.9)	127.2 (88.3 to 172.4)
North Macedonia	Alcohol use	1.6 (1.2 to 2.1)	52.1 (38.2 to 67.8)	2.3 (1.4 to 3.4)	54.4 (39.9 to 70.6)	1.1 (0.7 to 1.6)	31.8 (20.2 to 45.8)	2.2 (1.3 to 3.6)	34 (21.6 to 49.4)
North Macedonia	Diet high in red meat	0.6 (0.1 to 0.8)	18.5 (4.8 to 25.3)	0.8 (0.2 to 1.3)	19.3 (5 to 26.5)	0.6 (0.2 to 1)	17.9 (4.3 to 28.3)	1.3 (0.3 to 2.2)	19.1 (4.7 to 30.3)
North Macedonia	Low physical activity	0.2 (0.1 to 0.3)	5 (2.9 to 8.8)	0.2 (0.1 to 0.5)	5.2 (3.1 to 9.2)	0.3 (0.1 to 0.5)	5.9 (3.2 to 10)	0.4 (0.2 to 0.8)	6.3 (3.4 to 10.7)
North Macedonia	Smoking	1.7 (1.1 to 2.3)	52 (33.8 to 73)	2.3 (1.3 to 3.5)	54.3 (35.3 to 75.8)	1.8 (1.2 to 2.6)	51.2 (33.7 to 74.3)	3.6 (2 to 5.9)	54.8 (36.1 to 79.4)
North Macedonia	Secondhand smoke	0.7 (0.2 to 1.2)	21.4 (4.9 to 37)	0.9 (0.2 to 1.8)	22.3 (5.2 to 38.7)	0.8 (0.2 to 1.4)	20.5 (4.9 to 37.4)	1.4 (0.3 to 2.8)	21.9 (5.2 to 40.1)
North Macedonia	Metabolic risks	2.2 (0.7 to 4.2)	42.8 (4.3 to 91.9)	2.2 (0.4 to 4.7)	45 (4.7 to 96.3)	4.3 (1.6 to 8.3)	82 (23.8 to 172.8)	5.7 (1.5 to 11.9)	87.7 (25.7 to 185.1)
North Macedonia	High body-mass index	0.9 (0.1 to 2)	11.9 (-11.3 to 36.6)	0.7 (-0.4 to 2)	12.7 (-11.9 to 38.7)	1.7 (0.4 to 3.4)	25.7 (-3.3 to 63.4)	1.7 (-0.3 to 4.2)	27.4 (-3.6 to 67.2)
North Macedonia	High fasting plasma glucose	1.4 (0.3 to 3.2)	32.8 (6.1 to 76.5)	1.6 (0.3 to 3.9)	34.4 (6.5 to 80.3)	2.8 (0.6 to 6.5)	61.2 (12.4 to 143.4)	4.3 (0.9 to 10.7)	65.5 (13.2 to 151.5)
Northern Mariana Islands	All risk factors	11.8 (6.7 to 17.4)	290.8 (170.6 to 429.3)	13.3 (6.9 to 20.7)	304.1 (177.6 to 448.2)	11.6 (6.6 to 16.5)	278.5 (167.1 to 397.4)	16.6 (9.2 to 26)	295.1 (176.4 to 418.8)
Northern Mariana Islands	Behavioral risks	3.6 (2.3 to 5.1)	101.4 (63.5 to 144.9)	4.6 (2.5 to 7.2)	106 (66.1 to 150.9)	3.2 (2.1 to 4.5)	86.3 (55.1 to 125.3)	5.2 (2.9 to 8.1)	91.5 (58 to 132.6)
Northern Mariana Islands	Alcohol use	0.2 (0 to 0.4)	5.8 (0 to 14.1)	0.3 (0 to 0.7)	6.1 (0 to 14.7)	0.2 (0 to 0.5)	7 (0 to 17)	0.4 (0 to 1.1)	7.5 (0 to 18.1)
Northern Mariana Islands	Diet high in red meat	1 (0.4 to 1.6)	30.1 (10.7 to 47.5)	1.4 (0.5 to 2.3)	31.5 (11.1 to 49.8)	0.9 (0.3 to 1.3)	24.9 (8.5 to 38.4)	1.5 (0.5 to 2.5)	26.4 (9 to 40.6)
Northern Mariana Islands	Low physical activity	0.5 (0.2 to 1)	12.2 (4.5 to 24.1)	0.6 (0.2 to 1.2)	12.8 (4.7 to 25.3)	0.6 (0.2 to 1)	12.3 (4.5 to 23.2)	0.7 (0.3 to 1.5)	13.1 (4.8 to 24.7)
Northern Mariana Islands	Smoking	1 (0.5 to 1.5)	26.2 (13.4 to 41.7)	1.2 (0.5 to 2.1)	27.4 (14.1 to 43.5)	0.8 (0.4 to 1.3)	20.5 (10.3 to 33.7)	1.2 (0.6 to 2.2)	21.8 (10.9 to 35.9)
Northern Mariana Islands	Secondhand smoke	1.1 (0.2 to 2.1)	32.3 (7.3 to 59.5)	1.5 (0.3 to 2.8)	33.8 (7.6 to 62.5)	0.9 (0.2 to 1.6)	25.7 (6.5 to 47.2)	1.5 (0.4 to 3)	27.3 (6.9 to 50.1)
Northern Mariana Islands	Metabolic risks	9.3 (4 to 15.1)	218.1 (93.9 to 355.4)	9.9 (4.2 to 16.8)	228 (98 to 372.4)	9.5 (4.4 to 14.7)	218.7 (103.2 to 340)	13 (5.8 to 21.9)	231.7 (109.6 to 358.2)
Northern Mariana Islands	High body-mass index	7.8 (2.8 to 13.7)	180.7 (66.8 to 312.5)	8.2 (2.9 to 14.9)	189 (71.3 to 326.9)	7.2 (2.7 to 12.5)	165 (62.2 to 285.4)	9.7 (3.4 to 17.5)	174.7 (66.6 to 302.7)

Location	Risk factor	1990				2019			
		Deaths	YLLs (Years of Life Lost)	YLDs (Years Lived with Disability)	DALYs (Disability-Adjusted Life Years)	Deaths	YLLs (Years of Life Lost)	YLDs (Years Lived with Disability)	DALYs (Disability-Adjusted Life Years)
Northern Mariana Islands	High fasting plasma glucose	2.3 (0.4 to 5.2)	52.3 (9.9 to 123.6)	2.4 (0.4 to 5.8)	54.7 (10.4 to 128.6)	3.3 (0.6 to 7.3)	74.6 (13.8 to 171.4)	4.5 (0.8 to 10.6)	79.1 (14.5 to 180.7)
Norway	All risk factors	7.4 (5.8 to 9)	193.6 (155.9 to 231.1)	14.4 (9.4 to 20.3)	207.9 (167.3 to 247.4)	4.8 (3.6 to 6.2)	111.1 (85.1 to 140.7)	15.9 (9.7 to 24.1)	127.1 (96.7 to 160.9)
Norway	Behavioral risks	5.8 (4.8 to 6.6)	164.3 (138.7 to 188.4)	11.9 (7.9 to 17.1)	176.2 (148.3 to 202.9)	3.3 (2.7 to 3.9)	85.7 (71.1 to 100.8)	12.2 (7.7 to 18.4)	97.9 (80.6 to 116.5)
Norway	Alcohol use	2.4 (1.9 to 3)	71 (55.7 to 86.5)	5.1 (3.3 to 7.5)	76.2 (59.5 to 93.2)	1.7 (1.3 to 2.1)	44.1 (34.3 to 55)	6.2 (3.8 to 9.5)	50.3 (38.9 to 63.1)
Norway	Diet high in red meat	1.1 (0.5 to 1.4)	29.6 (13.7 to 39)	2.2 (0.9 to 3.4)	31.7 (14.8 to 42.1)	0.7 (0.4 to 1)	18.6 (9 to 25.7)	2.6 (1.2 to 4.5)	21.2 (10.3 to 29.4)
Norway	Low physical activity	0.4 (0.1 to 0.7)	8.4 (2.8 to 16.9)	0.7 (0.2 to 1.5)	9.1 (3 to 18.2)	0.3 (0.1 to 0.5)	5.5 (1.8 to 10.7)	0.8 (0.2 to 1.8)	6.3 (2 to 12.3)
Norway	Smoking	2 (1.4 to 2.7)	57.9 (40.1 to 77.3)	4.2 (2.4 to 6.4)	62 (42.6 to 82.6)	0.8 (0.6 to 1)	20.1 (14.4 to 26.8)	2.9 (1.7 to 4.7)	23 (16.2 to 30.6)
Norway	Secondhand smoke	0.5 (0.1 to 0.8)	14.9 (3.6 to 25.6)	1.1 (0.2 to 2)	16 (3.9 to 27.8)	0.2 (0 to 0.3)	5.4 (1.3 to 9.4)	0.7 (0.2 to 1.5)	6.1 (1.4 to 10.7)
Norway	Metabolic risks	2.1 (0.7 to 4)	39 (9.4 to 79.3)	3.2 (0.8 to 6.8)	42.2 (10.4 to 86.4)	1.9 (0.7 to 3.5)	32.9 (9.7 to 67)	4.9 (1.4 to 10.1)	37.8 (11.2 to 75.5)
Norway	High body-mass index	0.8 (0.2 to 1.7)	11.6 (-3.1 to 30.8)	1 (-0.1 to 2.4)	12.5 (-3.3 to 32.9)	0.7 (0.2 to 1.4)	10.1 (-0.8 to 24.2)	1.5 (0 to 3.7)	11.6 (-0.7 to 27.6)
Norway	High fasting plasma glucose	1.3 (0.3 to 3)	28.5 (5.3 to 65.2)	2.3 (0.4 to 5.7)	30.9 (5.7 to 70.7)	1.2 (0.2 to 2.7)	24.1 (4.6 to 53.6)	3.5 (0.6 to 8.4)	27.6 (5.3 to 61.7)
Oman	All risk factors	1.7 (0.9 to 3.1)	43 (20.4 to 78.1)	1.9 (0.9 to 3.5)	44.9 (21.4 to 81.5)	3.9 (2.1 to 6.3)	84.5 (44.2 to 136.8)	6.1 (3 to 10.6)	90.6 (47.3 to 146.5)
Oman	Behavioral risks	0.8 (0.5 to 1.3)	22.7 (12.6 to 36.9)	1 (0.5 to 1.6)	23.7 (13.2 to 38.3)	1.3 (0.8 to 1.8)	33.1 (20.5 to 45.4)	2.4 (1.4 to 3.8)	35.6 (21.8 to 49)
Oman	Alcohol use	0 (0 to 0)	0.8 (0.3 to 1.3)	0 (0 to 0.1)	0.8 (0.4 to 1.4)	0 (0 to 0.1)	1.3 (0.7 to 2)	0.1 (0 to 0.2)	1.4 (0.8 to 2.1)
Oman	Diet high in red meat	0.3 (0.1 to 0.5)	8.7 (2.2 to 15.2)	0.4 (0.1 to 0.7)	9.1 (2.3 to 15.8)	0.5 (0.2 to 0.7)	13.7 (4.7 to 19.9)	1 (0.3 to 1.6)	14.8 (5 to 21.4)
Oman	Low physical activity	0.2 (0.1 to 0.4)	4.9 (1.7 to 9.7)	0.2 (0.1 to 0.5)	5.2 (1.8 to 10.2)	0.4 (0.2 to 0.7)	9 (3.3 to 15.9)	0.7 (0.2 to 1.3)	9.6 (3.6 to 17.3)
Oman	Smoking	0.1 (0 to 0.1)	2.2 (1.2 to 3.7)	0.1 (0 to 0.2)	2.3 (1.2 to 3.9)	0.1 (0.1 to 0.1)	2.1 (1.3 to 3.2)	0.2 (0.1 to 0.3)	2.2 (1.3 to 3.4)
Oman	Secondhand smoke	0.2 (0.1 to 0.4)	6.7 (1.5 to 13.4)	0.3 (0.1 to 0.6)	7 (1.6 to 13.9)	0.3 (0.1 to 0.5)	8 (1.8 to 14.8)	0.6 (0.1 to 1.1)	8.6 (1.9 to 16.1)
Oman	Metabolic risks	1 (0.3 to 2.2)	22 (4 to 51.2)	1 (0.2 to 2.3)	22.9 (4.1 to 52.9)	2.8 (1.1 to 5.2)	56.2 (16 to 109.9)	4 (1.2 to 8.2)	60.2 (17.2 to 116.9)
Oman	High body-mass index	0.3 (0 to 0.8)	5.3 (-3.3 to 16.7)	0.2 (-0.1 to 0.7)	5.5 (-3.3 to 17.4)	1.3 (0.3 to 2.6)	22.1 (-1.2 to 51.1)	1.5 (-0.2 to 3.4)	23.6 (-1.2 to 54.4)
Oman	High fasting plasma glucose	0.7 (0.1 to 1.8)	17.4 (2.9 to 44.4)	0.8 (0.1 to 2)	18.2 (3 to 46.3)	1.7 (0.3 to 3.9)	37.9 (7.4 to 86.3)	2.8 (0.5 to 6.8)	40.8 (8 to 93.3)
Pakistan	All risk factors	4.4 (2.2 to 8)	116.6 (60.6 to 208.2)	2.8 (1.4 to 5.2)	119.5 (62.1 to 213.8)	8.6 (4.4 to 15.2)	211.3 (103.6 to 385.4)	6.3 (3 to 11.5)	217.7 (106.7 to 393.1)
Pakistan	Behavioral risks	2.6 (1.4 to 4.3)	76.4 (41.8 to 122.2)	1.8 (0.9 to 3.1)	78.2 (42.8 to 124.9)	3.7 (2.2 to 5.5)	104.6 (58.7 to 159.3)	3 (1.6 to 4.9)	107.6 (61.1 to 163.6)
Pakistan	Alcohol use	0.1 (0 to 0.1)	1.7 (1 to 2.8)	0 (0 to 0.1)	1.8 (1 to 2.8)	0.1 (0.1 to 0.2)	3.9 (2.3 to 6.1)	0.1 (0.1 to 0.2)	4 (2.4 to 6.2)
Pakistan	Diet high in red meat	0.6 (0.1 to 1)	17.7 (3.5 to 29.4)	0.4 (0.1 to 0.7)	18.1 (3.7 to 30.1)	1 (0.2 to 1.5)	29.2 (6.5 to 47.9)	0.8 (0.2 to 1.4)	30.1 (6.7 to 49.2)
Pakistan	Low physical activity	0.4 (0.2 to 0.7)	8.4 (4 to 15.3)	0.2 (0.1 to 0.4)	8.6 (4.1 to 15.7)	0.6 (0.3 to 1)	13.1 (6.6 to 22.8)	0.4 (0.2 to 0.7)	13.5 (6.9 to 23.3)
Pakistan	Smoking	0.6 (0.3 to 1)	15 (7.6 to 25.9)	0.4 (0.2 to 0.7)	15.4 (7.8 to 26.5)	0.6 (0.3 to 1)	15.8 (8.2 to 26.2)	0.5 (0.2 to 0.8)	16.3 (8.5 to 26.8)
Pakistan	Secondhand smoke	1.2 (0.3 to 2.2)	35.7 (8.3 to 67.9)	0.8 (0.2 to 1.6)	36.5 (8.5 to 69.4)	1.5 (0.3 to 2.7)	45.3 (10.6 to 82.5)	1.3 (0.3 to 2.5)	46.5 (10.9 to 84.6)
Pakistan	Metabolic risks	1.9 (0.4 to 4.6)	43.9 (6.5 to 109.9)	1.1 (0.2 to 2.8)	45 (6.7 to 112.6)	5.3 (1.6 to 11.7)	115.3 (22.1 to 274.8)	3.6 (0.8 to 8.4)	118.8 (23 to 281.7)
Pakistan	High body-mass index	0.4 (0 to 1.2)	4.5 (-6 to 19)	0.2 (-0.1 to 0.6)	4.7 (-6.1 to 19.6)	1.2 (0 to 3)	14.1 (-19 to 49.1)	0.6 (-0.4 to 1.7)	14.6 (-19.3 to 50.8)
Pakistan	High fasting plasma glucose	1.6 (0.3 to 4.2)	39.9 (6.4 to 105.2)	1 (0.2 to 2.7)	40.9 (6.7 to 107.8)	4.2 (0.9 to 10.3)	104.4 (20.3 to 258.9)	3.1 (0.6 to 8)	107.5 (21 to 265.3)
Palau	All risk factors	11.2 (6.2 to 16.9)	283 (159.2 to 419.2)	11.3 (6 to 18)	294.4 (166.8 to 436.1)	14.2 (8.3 to 21)	352 (208 to 523.1)	18.5 (10.2 to 29.7)	370.5 (220.6 to 550)
Palau	Behavioral risks	3.5 (2.2 to 5.2)	100.7 (59.5 to 152.3)	4 (2.1 to 6.6)	104.7 (61.9 to 157.6)	3.8 (2.4 to 5.6)	106.6 (65.1 to 157.6)	5.6 (3.1 to 9)	112.2 (68.8 to 167.6)
Palau	Alcohol use	0.3 (0 to 0.5)	8.9 (1.1 to 18.6)	0.4 (0 to 0.8)	9.3 (1.2 to 19.2)	0.4 (0.1 to 0.7)	12.4 (3.2 to 25)	0.7 (0.2 to 1.4)	13.1 (3.3 to 26.7)
Palau	Diet high in red meat	0.9 (0.2 to 1.4)	26.6 (7.4 to 43.3)	1.1 (0.3 to 1.9)	27.7 (7.8 to 45.2)	1 (0.3 to 1.6)	28.4 (9.1 to 45.9)	1.5 (0.5 to 2.6)	29.9 (9.6 to 48)
Palau	Low physical activity	0.5 (0.2 to 1)	11.6 (4.3 to 23)	0.5 (0.2 to 1)	12 (4.5 to 24)	0.7 (0.2 to 1.2)	14.7 (5.2 to 29.2)	0.8 (0.3 to 1.7)	15.5 (5.5 to 30.8)
Palau	Smoking	0.8 (0.4 to 1.2)	21.1 (11 to 35.3)	0.8 (0.4 to 1.6)	21.9 (11.4 to 36.8)	0.7 (0.4 to 1.1)	18.3 (9.1 to 30)	1 (0.5 to 1.7)	19.3 (9.6 to 31.3)
Palau	Secondhand smoke	1.3 (0.3 to 2.3)	37.2 (7.6 to 68.9)	1.5 (0.3 to 2.9)	38.7 (7.8 to 71.9)	1.3 (0.3 to 2.3)	37.7 (9.1 to 69.3)	2 (0.4 to 3.9)	39.6 (9.7 to 73)

Location	Risk factor	1990				2019			
		Deaths	YLLs (Years of Life Lost)	YLDs (Years Lived with Disability)	DALYs (Disability-Adjusted Life Years)	Deaths	YLLs (Years of Life Lost)	YLDs (Years Lived with Disability)	DALYs (Disability-Adjusted Life Years)
Palau	Metabolic risks	8.6 (3.7 to 14.4)	207.1 (89 to 338.5)	8.3 (3.4 to 14.3)	215.4 (92.7 to 352)	11.7 (5.4 to 18.7)	277.8 (128.7 to 444.2)	14.5 (6.5 to 25)	292.3 (136.4 to 467.9)
Palau	High body-mass index	6.6 (2.3 to 11.7)	158 (54.8 to 277.8)	6.3 (2.1 to 11.6)	164.3 (57.3 to 286.6)	8.2 (3 to 14.7)	190.7 (70.3 to 337.5)	9.9 (3.5 to 19.1)	200.6 (74.9 to 358.1)
Palau	High fasting plasma glucose	2.7 (0.5 to 6.2)	64.1 (12 to 149.7)	2.6 (0.5 to 6.4)	66.7 (12.4 to 155.1)	4.9 (1 to 10.7)	117.1 (23.5 to 265)	6.2 (1.2 to 14.6)	123.3 (24.7 to 278.4)
Palestine	All risk factors	3.2 (1.6 to 5.6)	79.3 (38.3 to 140)	3 (1.4 to 5.5)	82.3 (40 to 145)	5.8 (3.1 to 9.4)	133.8 (69.7 to 216.4)	6.8 (3.3 to 11.8)	140.6 (73.4 to 227.7)
Palestine	Behavioral risks	1.7 (0.9 to 2.7)	49.1 (26.9 to 79.1)	1.8 (0.9 to 3.1)	50.9 (28 to 81.9)	2.2 (1.4 to 3.1)	60.8 (36 to 88.1)	3.1 (1.7 to 5)	63.9 (37.7 to 92.9)
Palestine	Alcohol use	0.1 (0.1 to 0.2)	3.1 (1.7 to 5.3)	0.1 (0.1 to 0.2)	3.2 (1.8 to 5.5)	0.2 (0.1 to 0.3)	5.6 (3.8 to 8.1)	0.3 (0.2 to 0.4)	5.9 (4 to 8.6)
Palestine	Diet high in red meat	0.3 (0.1 to 0.5)	8.6 (1.6 to 14.9)	0.3 (0.1 to 0.6)	8.9 (1.6 to 15.4)	0.3 (0.1 to 0.5)	9.2 (1.8 to 13.9)	0.5 (0.1 to 0.8)	9.6 (1.9 to 14.6)
Palestine	Low physical activity	0.4 (0.1 to 0.7)	8.9 (3.3 to 17.6)	0.4 (0.1 to 0.7)	9.3 (3.4 to 18.3)	0.6 (0.2 to 1)	13 (5 to 23)	0.7 (0.2 to 1.3)	13.6 (5.3 to 24.1)
Palestine	Smoking	0.2 (0.1 to 0.3)	5.1 (2.6 to 8.7)	0.2 (0.1 to 0.3)	5.3 (2.7 to 9)	0.2 (0.2 to 0.4)	5.9 (3.6 to 8.7)	0.3 (0.2 to 0.5)	6.2 (3.8 to 9.2)
Palestine	Secondhand smoke	0.8 (0.2 to 1.4)	24.9 (5.8 to 47.5)	0.9 (0.2 to 1.8)	25.8 (6 to 49.2)	1 (0.2 to 1.7)	29 (6.7 to 51.6)	1.5 (0.3 to 2.8)	30.5 (7.1 to 53.9)
Palestine	Metabolic risks	1.7 (0.4 to 3.7)	33.2 (-0.2 to 84.1)	1.3 (0 to 3.2)	34.5 (-0.2 to 87.6)	3.9 (1.4 to 7.7)	80.2 (19.1 to 164)	4.1 (0.9 to 8.7)	84.2 (19.8 to 172.3)
Palestine	High body-mass index	0.6 (-0.1 to 1.4)	4.7 (-15.1 to 25.3)	0.2 (-0.5 to 1)	4.9 (-15.6 to 26.3)	1.3 (0.2 to 2.6)	19 (-9.3 to 50.4)	0.9 (-0.5 to 2.5)	19.9 (-9.9 to 53.2)
Palestine	High fasting plasma glucose	1.2 (0.2 to 3.1)	30 (5.1 to 76.9)	1.2 (0.2 to 3)	31.1 (5.3 to 79.5)	2.9 (0.6 to 6.5)	65.8 (13.4 to 150.3)	3.4 (0.7 to 8.3)	69.2 (14.2 to 158)
Panama	All risk factors	2.4 (1.6 to 3.4)	62.9 (41.9 to 86.6)	2.9 (1.8 to 4.6)	65.9 (43.9 to 91.1)	3.1 (1.8 to 5.1)	75.4 (43.4 to 122.9)	5.4 (2.9 to 9.4)	80.8 (46.7 to 131.9)
Panama	Behavioral risks	1.5 (1.1 to 1.8)	44.2 (33.2 to 53.9)	2 (1.3 to 2.7)	46.2 (34.8 to 56.3)	1.5 (1 to 2)	43.2 (29.3 to 59.2)	2.9 (1.7 to 4.5)	46.1 (31.3 to 63.1)
Panama	Alcohol use	0.5 (0.4 to 0.7)	18 (13.5 to 23.1)	0.8 (0.5 to 1.1)	18.8 (14 to 24.2)	0.6 (0.4 to 0.9)	20.8 (13.8 to 29.6)	1.4 (0.8 to 2.2)	22.1 (15 to 31.7)
Panama	Diet high in red meat	0.4 (0.2 to 0.6)	13.4 (5 to 17.8)	0.6 (0.2 to 0.9)	14 (5.3 to 18.6)	0.5 (0.2 to 0.7)	13.8 (4.9 to 21.4)	0.9 (0.3 to 1.6)	14.8 (5.2 to 22.9)
Panama	Low physical activity	0.1 (0 to 0.1)	1.6 (1 to 3.6)	0.1 (0 to 0.2)	1.7 (1 to 3.8)	0.1 (0 to 0.2)	1.8 (1 to 3.8)	0.1 (0.1 to 0.3)	1.9 (1 to 4.1)
Panama	Smoking	0.3 (0.2 to 0.4)	6.6 (4.2 to 9.6)	0.3 (0.2 to 0.5)	6.9 (4.4 to 10.1)	0.2 (0.1 to 0.3)	4.3 (2.5 to 6.8)	0.3 (0.2 to 0.5)	4.6 (2.7 to 7.3)
Panama	Secondhand smoke	0.2 (0 to 0.4)	6.6 (1.5 to 11.4)	0.3 (0.1 to 0.5)	6.9 (1.6 to 12)	0.1 (0 to 0.3)	4.3 (1.1 to 8.1)	0.3 (0.1 to 0.6)	4.6 (1.1 to 8.6)
Panama	Metabolic risks	1.1 (0.3 to 2.2)	21 (3 to 46.8)	1.1 (0.2 to 2.5)	22.1 (3.3 to 49.1)	1.8 (0.6 to 3.7)	35.9 (6.5 to 80.7)	2.8 (0.6 to 6.1)	38.7 (7 to 87)
Panama	High body-mass index	0.3 (0 to 0.7)	2.8 (-5.3 to 11.2)	0.2 (-0.1 to 0.6)	3 (-5.5 to 11.9)	0.6 (0.1 to 1.3)	7.2 (-6.8 to 22.4)	0.7 (-0.2 to 1.8)	7.8 (-7.1 to 24.3)
Panama	High fasting plasma glucose	0.8 (0.2 to 1.9)	18.8 (3.4 to 41.9)	0.9 (0.2 to 2.2)	19.7 (3.6 to 44.2)	1.3 (0.3 to 3.1)	30.5 (5.9 to 72.5)	2.3 (0.4 to 5.6)	32.8 (6.5 to 76.8)
Papua New Guinea	All risk factors	9.2 (5.3 to 14)	285.3 (165.8 to 432.3)	6.8 (3.7 to 11.1)	292.1 (170.2 to 442.1)	12.5 (6.9 to 20.1)	380.6 (212 to 618.1)	10.2 (5.4 to 16.9)	390.8 (216.8 to 633.2)
Papua New Guinea	Behavioral risks	4.5 (2.7 to 6.4)	147.8 (85.9 to 214.2)	3.5 (1.9 to 5.4)	151.3 (87.8 to 219)	4.9 (3 to 7.3)	158.8 (93.9 to 238.8)	4.2 (2.3 to 6.7)	163 (96.1 to 244.7)
Papua New Guinea	Alcohol use	0.4 (0.2 to 0.7)	14.6 (6.1 to 25.4)	0.3 (0.1 to 0.6)	14.9 (6.2 to 26)	0.4 (0.1 to 0.7)	14.5 (4.9 to 27.3)	0.4 (0.1 to 0.7)	14.9 (5 to 28.1)
Papua New Guinea	Diet high in red meat	0.9 (0.2 to 1.4)	29.9 (7.4 to 48.1)	0.7 (0.2 to 1.2)	30.6 (7.6 to 49)	1 (0.2 to 1.6)	32.7 (7.2 to 52.8)	0.9 (0.2 to 1.5)	33.5 (7.4 to 54)
Papua New Guinea	Low physical activity	0.4 (0.2 to 0.8)	12.3 (4.9 to 24.7)	0.3 (0.1 to 0.7)	12.6 (5 to 25.3)	0.5 (0.2 to 1.1)	15.3 (6 to 31.8)	0.4 (0.2 to 1)	15.7 (6.2 to 32.7)
Papua New Guinea	Smoking	1.4 (0.7 to 2.3)	42.2 (19.8 to 70)	1 (0.4 to 1.8)	43.2 (20.3 to 71.5)	1.4 (0.7 to 2.3)	41.2 (20.1 to 67.7)	1.1 (0.5 to 2)	42.4 (20.6 to 69.7)
Papua New Guinea	Secondhand smoke	1.6 (0.4 to 2.9)	56 (12.6 to 100.7)	1.3 (0.3 to 2.5)	57.3 (13 to 103.2)	1.8 (0.5 to 3.4)	62 (15.7 to 115.1)	1.6 (0.4 to 3.1)	63.6 (16.1 to 118.2)
Papua New Guinea	Metabolic risks	5.4 (1.9 to 10.3)	157.8 (56 to 299.5)	3.8 (1.3 to 7.4)	161.6 (57.2 to 307.4)	8.6 (3.2 to 16.1)	251.2 (93.9 to 473.3)	6.8 (2.5 to 13.2)	258 (97.3 to 490.7)
Papua New Guinea	High body-mass index	3.5 (0.9 to 7.6)	102.2 (27.6 to 217.3)	2.5 (0.6 to 5.3)	104.6 (28.3 to 222.2)	4.9 (1.2 to 10.5)	142.4 (36.9 to 303.9)	3.8 (1 to 8)	146.3 (37.9 to 310.4)
Papua New Guinea	High fasting plasma glucose	2.1 (0.4 to 5)	62.3 (11.3 to 152.4)	1.5 (0.3 to 3.7)	63.8 (11.6 to 156.1)	4.2 (0.8 to 9.8)	124.2 (22.9 to 299)	3.4 (0.6 to 8.6)	127.6 (23.6 to 307)
Paraguay	All risk factors	3.4 (2.5 to 4.4)	89.8 (65.6 to 117.2)	3.2 (2 to 4.7)	93 (68.1 to 121.2)	4.7 (3.1 to 7.1)	115.3 (73.5 to 174.5)	6.2 (3.6 to 9.6)	121.5 (77.9 to 184.9)
Paraguay	Behavioral risks	2.6 (2 to 3.2)	75.6 (59.2 to 93.4)	2.6 (1.7 to 3.7)	78.2 (61.1 to 96.5)	3 (2.1 to 4.1)	83.9 (56.6 to 116.3)	4.3 (2.5 to 6.6)	88.2 (59.6 to 122)
Paraguay	Alcohol use	1 (0.7 to 1.2)	31.4 (23.5 to 40.2)	1.1 (0.7 to 1.5)	32.5 (24.3 to 41.3)	1 (0.7 to 1.5)	32.6 (20.9 to 47)	1.6 (0.9 to 2.6)	34.3 (21.9 to 49.2)
Paraguay	Diet high in red meat	0.8 (0.4 to 1.1)	22.6 (10.9 to 32.2)	0.8 (0.4 to 1.2)	23.4 (11.4 to 33.4)	1 (0.4 to 1.5)	27.5 (12.4 to 42.1)	1.4 (0.6 to 2.4)	28.9 (12.9 to 44.6)
Paraguay	Low physical activity	0.1 (0 to 0.1)	1.7 (1 to 3.6)	0.1 (0 to 0.1)	1.8 (1 to 3.8)	0.1 (0.1 to 0.3)	2.7 (1.4 to 5.6)	0.1 (0.1 to 0.3)	2.9 (1.5 to 5.9)
Paraguay	Smoking	0.7 (0.4 to 0.9)	16 (9.9 to 23.2)	0.6 (0.3 to 1)	16.6 (10.3 to 24)	0.7 (0.5 to 1.1)	15.7 (9.3 to 24.6)	0.9 (0.5 to 1.4)	16.5 (9.8 to 26)
Paraguay	Secondhand smoke	0.3 (0.1 to 0.6)	10 (2.4 to 17.3)	0.3 (0.1 to 0.6)	10.3 (2.5 to 17.9)	0.4 (0.1 to 0.7)	10.9 (2.7 to 20.7)	0.6 (0.1 to 1.1)	11.5 (2.8 to 21.8)

Location	Risk factor	1990				2019			
		Deaths	YLLs (Years of Life Lost)	YLDs (Years Lived with Disability)	DALYs (Disability-Adjusted Life Years)	Deaths	YLLs (Years of Life Lost)	YLDs (Years Lived with Disability)	DALYs (Disability-Adjusted Life Years)
Paraguay	Metabolic risks	1 (0.3 to 2)	17.9 (0.7 to 40.8)	0.8 (0.1 to 1.7)	18.7 (0.9 to 42.4)	2 (0.7 to 4.2)	38 (6.1 to 89.1)	2.2 (0.5 to 4.9)	40.2 (6.6 to 94.2)
Paraguay	High body-mass index	0.4 (0 to 0.9)	3.3 (-6.9 to 14)	0.2 (-0.2 to 0.6)	3.5 (-7.1 to 14.7)	0.7 (0.1 to 1.8)	8.8 (-7.9 to 29.3)	0.6 (-0.3 to 1.8)	9.4 (-8.2 to 30.9)
Paraguay	High fasting plasma glucose	0.7 (0.1 to 1.5)	15.2 (2.9 to 35.5)	0.6 (0.1 to 1.4)	15.8 (3 to 37.2)	1.4 (0.3 to 3.2)	31.1 (6 to 74.6)	1.7 (0.3 to 4.4)	32.8 (6.3 to 78.7)
Peru	All risk factors	1.9 (1.3 to 2.7)	52.5 (33.9 to 72.3)	1.6 (0.9 to 2.4)	54.1 (35 to 74.3)	2 (1.2 to 3.2)	50 (29.9 to 80)	2.8 (1.5 to 4.7)	52.7 (31.5 to 84.1)
Peru	Behavioral risks	1.2 (0.9 to 1.6)	40 (28.4 to 52.4)	1.1 (0.7 to 1.7)	41.2 (29.2 to 53.8)	1.1 (0.7 to 1.5)	32.9 (21.5 to 48.7)	1.8 (1 to 2.8)	34.7 (22.5 to 51.2)
Peru	Alcohol use	0.7 (0.5 to 1)	24.4 (16.1 to 33.7)	0.7 (0.4 to 1.1)	25 (16.6 to 34.6)	0.6 (0.4 to 0.9)	20.9 (12.6 to 31.7)	1.1 (0.6 to 1.8)	22 (13.2 to 33.3)
Peru	Diet high in red meat	0.2 (0 to 0.3)	5.8 (1.1 to 8.5)	0.2 (0 to 0.3)	6 (1.1 to 8.7)	0.2 (0 to 0.3)	5.4 (1 to 8.9)	0.3 (0.1 to 0.5)	5.7 (1 to 9.3)
Peru	Low physical activity	0.1 (0 to 0.1)	1.4 (0.9 to 3.9)	0 (0 to 0.1)	1.5 (1 to 4)	0.1 (0 to 0.1)	1.3 (0.7 to 3.1)	0.1 (0 to 0.2)	1.4 (0.8 to 3.3)
Peru	Smoking	0.1 (0.1 to 0.2)	2.9 (1.3 to 4.8)	0.1 (0 to 0.2)	3 (1.4 to 4.9)	0.1 (0 to 0.1)	2.1 (1 to 3.6)	0.1 (0.1 to 0.2)	2.2 (1.1 to 3.8)
Peru	Secondhand smoke	0.2 (0.1 to 0.4)	6.8 (1.6 to 12.3)	0.2 (0 to 0.4)	7 (1.7 to 12.7)	0.1 (0 to 0.3)	4.3 (1 to 8.1)	0.2 (0 to 0.5)	4.5 (1.1 to 8.5)
Peru	Metabolic risks	0.8 (0.2 to 1.5)	13.6 (-0.7 to 31.6)	0.5 (0.1 to 1.1)	14.1 (-0.6 to 32.6)	1.1 (0.3 to 2.2)	18.5 (1.6 to 43.3)	1.1 (0.2 to 2.4)	19.6 (1.8 to 46)
Peru	High body-mass index	0.3 (0 to 0.8)	4 (-6 to 14.1)	0.2 (-0.1 to 0.5)	4.2 (-6.1 to 14.6)	0.5 (0 to 1.2)	6.1 (-5.8 to 20.3)	0.4 (-0.3 to 1.2)	6.5 (-6.1 to 21.4)
Peru	High fasting plasma glucose	0.4 (0.1 to 1)	10 (1.8 to 23.8)	0.3 (0.1 to 0.8)	10.3 (1.9 to 24.4)	0.6 (0.1 to 1.5)	13.3 (2.4 to 33.5)	0.8 (0.1 to 2.1)	14.1 (2.6 to 35)
Philippines	All risk factors	6.1 (4 to 8.5)	156.4 (103.9 to 215.6)	4.8 (2.8 to 7.1)	161.2 (107.1 to 221.8)	6.9 (4.3 to 10.4)	189.6 (119.1 to 284)	7 (4 to 11)	196.6 (124.4 to 293.4)
Philippines	Behavioral risks	3.3 (2.4 to 4.2)	92.9 (65.8 to 119.1)	2.8 (1.8 to 4.1)	95.7 (67.9 to 122.7)	3.2 (2.2 to 4.4)	97.3 (65.9 to 136.7)	3.6 (2.1 to 5.5)	100.8 (69 to 139.6)
Philippines	Alcohol use	1.2 (0.9 to 1.5)	37.1 (27.4 to 47.6)	1.1 (0.7 to 1.6)	38.2 (28.2 to 49.1)	1.3 (0.9 to 1.8)	41.6 (27.7 to 58.2)	1.5 (0.9 to 2.4)	43.1 (29.2 to 59.7)
Philippines	Diet high in red meat	0.5 (0.1 to 0.6)	13.5 (3.1 to 18.8)	0.4 (0.1 to 0.7)	13.9 (3.2 to 19.4)	0.6 (0.2 to 1)	19.4 (6 to 31)	0.7 (0.2 to 1.2)	20.1 (6.3 to 31.8)
Philippines	Low physical activity	0.1 (0.1 to 0.2)	2.8 (1.9 to 6.8)	0.1 (0 to 0.2)	2.9 (1.9 to 7.1)	0.1 (0.1 to 0.2)	2.7 (1.6 to 6.7)	0.1 (0.1 to 0.2)	2.8 (1.7 to 6.9)
Philippines	Smoking	0.8 (0.5 to 1.1)	16.8 (9.4 to 25.3)	0.5 (0.3 to 0.9)	17.3 (9.7 to 26)	0.6 (0.4 to 0.9)	13.4 (7.6 to 21.2)	0.5 (0.3 to 0.9)	13.9 (7.9 to 21.9)
Philippines	Secondhand smoke	0.9 (0.2 to 1.6)	27.5 (6.6 to 47.6)	0.8 (0.2 to 1.5)	28.4 (6.8 to 48.9)	0.8 (0.2 to 1.5)	25.5 (5.8 to 46.4)	0.9 (0.2 to 1.8)	26.4 (6 to 47.6)
Philippines	Metabolic risks	3.2 (1.2 to 5.8)	73.7 (28.6 to 131.4)	2.3 (0.9 to 4.3)	76.1 (29.4 to 135.7)	4.3 (1.8 to 7.7)	107.9 (46.3 to 193)	4.1 (1.6 to 7.5)	111.9 (47.7 to 200.2)
Philippines	High body-mass index	1.9 (0.5 to 4)	47.3 (12 to 99.4)	1.5 (0.4 to 3.2)	48.7 (12.4 to 102.4)	3 (1 to 5.7)	77.3 (25.8 to 146.2)	2.9 (0.8 to 5.9)	80.2 (26.6 to 151.2)
Philippines	High fasting plasma glucose	1.4 (0.3 to 3.2)	29 (5.4 to 65.8)	0.9 (0.2 to 2.2)	29.9 (5.6 to 68)	1.5 (0.3 to 3.6)	35.9 (6.9 to 86.4)	1.4 (0.2 to 3.4)	37.3 (7.3 to 89.1)
Poland	All risk factors	6.1 (4.6 to 7.7)	166.1 (128.5 to 205.1)	7.3 (4.7 to 10.4)	173.4 (133.6 to 214.1)	6.1 (4.2 to 8.6)	150.1 (104.1 to 203.9)	11.5 (7 to 17.5)	161.6 (113.6 to 219.5)
Poland	Behavioral risks	4.2 (3.4 to 5)	129.8 (106.7 to 152.2)	5.6 (3.7 to 7.8)	135.5 (110.7 to 158.9)	3.8 (2.8 to 5.1)	106.4 (77.8 to 141.6)	8.3 (5.3 to 12.2)	114.7 (84.6 to 150.7)
Poland	Alcohol use	1.2 (0.9 to 1.6)	42.9 (32.8 to 53.4)	1.8 (1.2 to 2.7)	44.8 (34.4 to 55.7)	1.2 (0.8 to 1.6)	37.1 (25.1 to 51.8)	2.9 (1.8 to 4.5)	40 (27.5 to 54.4)
Poland	Diet high in red meat	1.1 (0.5 to 1.4)	30.9 (15.6 to 41)	1.3 (0.6 to 2.1)	32.2 (16.2 to 42.8)	1 (0.5 to 1.5)	25.7 (12.3 to 39)	2 (0.9 to 3.3)	27.7 (13.4 to 41.7)
Poland	Low physical activity	0.3 (0.1 to 0.4)	6.1 (3.1 to 10.5)	0.3 (0.1 to 0.5)	6.4 (3.3 to 11)	0.3 (0.1 to 0.5)	5.5 (2.6 to 9.7)	0.4 (0.2 to 0.8)	5.9 (2.9 to 10.4)
Poland	Smoking	1.5 (1 to 1.9)	45.2 (31.6 to 59.9)	2 (1.2 to 3)	47.2 (33 to 62.3)	1.4 (0.9 to 2)	37.7 (24.8 to 53.9)	2.9 (1.7 to 4.7)	40.6 (27.1 to 57.4)
Poland	Secondhand smoke	0.6 (0.1 to 1)	16.6 (3.9 to 28.8)	0.7 (0.2 to 1.3)	17.3 (4.1 to 30.1)	0.4 (0.1 to 0.7)	10.1 (2 to 18.8)	0.8 (0.2 to 1.5)	10.9 (2.2 to 20.1)
Poland	Metabolic risks	2.3 (0.9 to 4.2)	44.6 (10.4 to 87.7)	2 (0.5 to 4.1)	46.6 (11 to 91.7)	2.8 (1.1 to 5.1)	55.2 (18.6 to 104.6)	4 (1.2 to 8.1)	59.2 (20.1 to 110.8)
Poland	High body-mass index	1.1 (0.2 to 2.2)	17.2 (-3.7 to 39.8)	0.8 (-0.1 to 1.9)	18 (-3.9 to 41.7)	1.5 (0.4 to 2.9)	25.5 (2.8 to 55.5)	1.8 (0.1 to 4.1)	27.3 (3 to 59.2)
Poland	High fasting plasma glucose	1.3 (0.2 to 2.8)	29.3 (5.5 to 65.5)	1.3 (0.2 to 3.2)	30.7 (5.7 to 68.3)	1.5 (0.3 to 3.5)	32.5 (6.2 to 75.7)	2.5 (0.4 to 6)	35 (6.6 to 80.6)
Portugal	All risk factors	6.9 (5.3 to 8.8)	188.3 (148.5 to 231.8)	13.1 (8.3 to 18.8)	201.3 (157.9 to 249.1)	5.1 (3.7 to 7)	125.1 (93.1 to 164)	16.6 (9.6 to 26)	141.7 (105.8 to 188.2)
Portugal	Behavioral risks	5.2 (4.3 to 6)	156.6 (129.6 to 180.9)	10.6 (7 to 15)	167.3 (138.1 to 193.9)	3.3 (2.7 to 4)	93.3 (76.1 to 111.8)	12.3 (7.5 to 18.7)	105.6 (85.2 to 127.9)
Portugal	Alcohol use	2.7 (2.1 to 3.3)	83.6 (65.8 to 102.1)	5.6 (3.7 to 8.1)	89.2 (70.1 to 108.7)	1.5 (1.1 to 1.9)	43.2 (33.7 to 54.2)	5.7 (3.4 to 8.8)	48.8 (37.7 to 61.7)
Portugal	Diet high in red meat	1.1 (0.5 to 1.4)	31.8 (14 to 41.9)	2.2 (0.9 to 3.4)	34 (14.8 to 44.8)	0.9 (0.5 to 1.3)	25.6 (12.6 to 35.5)	3.4 (1.5 to 5.9)	29 (14.1 to 40.5)
Portugal	Low physical activity	0.4 (0.1 to 0.7)	9.2 (3.1 to 18.4)	0.7 (0.2 to 1.5)	9.9 (3.3 to 19.9)	0.3 (0.1 to 0.6)	6.7 (2.1 to 12.8)	0.9 (0.3 to 1.9)	7.5 (2.4 to 14.5)

Location	Risk factor	1990				2019			
		Deaths	YLLs (Years of Life Lost)	YLDs (Years Lived with Disability)	DALYs (Disability-Adjusted Life Years)	Deaths	YLLs (Years of Life Lost)	YLDs (Years Lived with Disability)	DALYs (Disability-Adjusted Life Years)
Portugal	Smoking	0.8 (0.5 to 1)	24.4 (15.9 to 33.7)	1.6 (0.9 to 2.6)	26.1 (17.1 to 36.1)	0.5 (0.4 to 0.7)	16.9 (10.7 to 23.7)	2.2 (1.1 to 3.7)	19.1 (12.1 to 26.7)
Portugal	Secondhand smoke	0.7 (0.2 to 1.2)	21.5 (5.1 to 36.7)	1.4 (0.3 to 2.6)	22.9 (5.4 to 39.7)	0.3 (0.1 to 0.6)	9.5 (2.3 to 16.7)	1.2 (0.3 to 2.4)	10.8 (2.6 to 18.8)
Portugal	Metabolic risks	2.2 (0.7 to 4.3)	39.5 (5.5 to 85.7)	3 (0.6 to 6.6)	42.5 (6.3 to 91.7)	2.2 (0.8 to 4.3)	39.9 (9.7 to 82.7)	5.4 (1.3 to 12.2)	45.4 (11 to 92.9)
Portugal	High body-mass index	0.7 (0.1 to 1.6)	7.8 (-9.4 to 25.8)	0.7 (-0.5 to 2)	8.5 (-9.9 to 27.2)	0.7 (0.1 to 1.5)	8.9 (-4.6 to 24)	1.2 (-0.6 to 3.4)	10.1 (-4.9 to 27)
Portugal	High fasting plasma glucose	1.5 (0.3 to 3.4)	33.2 (6.4 to 75)	2.5 (0.4 to 5.9)	35.6 (6.9 to 80.8)	1.6 (0.3 to 3.5)	32.9 (6.5 to 73.3)	4.5 (0.8 to 10.9)	37.4 (7.6 to 83.2)
Puerto Rico	All risk factors	4.5 (3 to 6.4)	112.5 (73.6 to 158.9)	7 (4.1 to 10.9)	119.5 (78.3 to 169.3)	4.6 (2.7 to 7.5)	110.2 (64.2 to 180.5)	10.7 (5.6 to 18.4)	120.9 (69.8 to 198.7)
Puerto Rico	Behavioral risks	2.4 (1.9 to 3)	75 (59.2 to 91.4)	4.4 (2.9 to 6.4)	79.4 (62.7 to 96.5)	2.1 (1.5 to 2.9)	63 (44.7 to 86.4)	5.8 (3.6 to 8.8)	68.9 (48.4 to 94.6)
Puerto Rico	Alcohol use	0.9 (0.7 to 1.2)	31.5 (23.4 to 41.9)	1.8 (1.1 to 2.7)	33.3 (24.7 to 43.9)	0.7 (0.5 to 1)	25.8 (17.5 to 36.4)	2.3 (1.4 to 3.6)	28.1 (19.2 to 39.6)
Puerto Rico	Diet high in red meat	0.4 (0.1 to 0.6)	13.2 (3.5 to 19)	0.8 (0.2 to 1.3)	14 (3.7 to 20.2)	0.4 (0.1 to 0.6)	12.1 (3.2 to 19.4)	1.1 (0.3 to 2)	13.2 (3.5 to 21.2)
Puerto Rico	Low physical activity	0.4 (0.1 to 0.8)	10.7 (3.4 to 19.6)	0.7 (0.2 to 1.3)	11.3 (3.6 to 20.9)	0.4 (0.1 to 0.7)	9.9 (3 to 18.2)	1 (0.3 to 2)	10.9 (3.3 to 20.1)
Puerto Rico	Smoking	0.5 (0.4 to 0.8)	14.9 (9.1 to 22.4)	0.9 (0.5 to 1.4)	15.8 (9.7 to 23.6)	0.5 (0.3 to 0.7)	12.3 (7.6 to 18.7)	1.2 (0.6 to 2)	13.5 (8.3 to 20.4)
Puerto Rico	Secondhand smoke	0.3 (0.1 to 0.5)	8.8 (2.1 to 15.4)	0.5 (0.1 to 0.9)	9.4 (2.2 to 16.3)	0.2 (0 to 0.4)	6.3 (1.5 to 11.8)	0.6 (0.1 to 1.1)	6.8 (1.7 to 12.9)
Puerto Rico	Metabolic risks	2.3 (0.7 to 4.4)	42.8 (3.6 to 92.8)	3 (0.5 to 6.3)	45.8 (4.2 to 98.5)	2.8 (1 to 5.6)	53.8 (8.1 to 120.8)	5.5 (1.1 to 12.3)	59.3 (9.1 to 132.5)
Puerto Rico	High body-mass index	0.8 (0 to 1.7)	6.9 (-13.4 to 28.4)	0.7 (-0.6 to 2.1)	7.6 (-14 to 29.9)	1 (0.1 to 2.1)	10.7 (-13.4 to 35.4)	1.3 (-0.9 to 3.8)	12.1 (-14.1 to 39.4)
Puerto Rico	High fasting plasma glucose	1.7 (0.3 to 3.7)	38.2 (7.5 to 85.7)	2.5 (0.5 to 5.8)	40.7 (8 to 91.9)	2 (0.4 to 4.7)	46.6 (9.4 to 109.3)	4.6 (0.9 to 11.1)	51.1 (10.4 to 120.3)
Qatar	All risk factors	7.1 (3.7 to 12.2)	158.8 (76.1 to 270.3)	6.6 (3.1 to 11.9)	165.4 (79.4 to 281.2)	13.6 (7.3 to 21.9)	250.4 (126.5 to 412.6)	20.4 (9.6 to 35.2)	270.8 (137.2 to 443.2)
Qatar	Behavioral risks	2.5 (1.6 to 3.7)	68.2 (42.6 to 99.2)	2.7 (1.6 to 4.4)	71 (44.4 to 102.8)	3.3 (2 to 4.7)	70.7 (43.1 to 103)	5.9 (3.2 to 9.4)	76.5 (46.6 to 110.6)
Qatar	Alcohol use	0.2 (0.1 to 0.3)	5 (3 to 7.7)	0.2 (0.1 to 0.3)	5.2 (3.2 to 8.1)	0.2 (0.1 to 0.2)	3.9 (2.2 to 5.9)	0.3 (0.2 to 0.5)	4.2 (2.3 to 6.4)
Qatar	Diet high in red meat	0.8 (0.2 to 1.2)	22.2 (7 to 35.1)	0.9 (0.3 to 1.5)	23.1 (7.4 to 36.5)	1 (0.3 to 1.5)	22.5 (6.5 to 35.5)	1.9 (0.5 to 3.2)	24.4 (7.3 to 38.7)
Qatar	Low physical activity	0.7 (0.3 to 1.3)	16.2 (5.8 to 29.3)	0.7 (0.2 to 1.3)	16.9 (6.1 to 30.9)	1.2 (0.5 to 2.1)	21.7 (8.4 to 38.1)	1.8 (0.6 to 3.4)	23.5 (9 to 41.5)
Qatar	Smoking	0.2 (0.1 to 0.2)	4.3 (2.4 to 7)	0.2 (0.1 to 0.3)	4.5 (2.5 to 7.3)	0.2 (0.1 to 0.2)	3.7 (2.1 to 6)	0.3 (0.1 to 0.5)	4 (2.2 to 6.4)
Qatar	Secondhand smoke	0.8 (0.2 to 1.4)	22.8 (5.6 to 42.1)	0.9 (0.2 to 1.7)	23.7 (5.8 to 43.6)	0.9 (0.2 to 1.6)	21.3 (4.9 to 39.2)	1.7 (0.4 to 3.3)	23 (5.4 to 42.2)
Qatar	Metabolic risks	5.1 (1.7 to 9.9)	99.5 (19.2 to 209.8)	4.3 (1 to 9.3)	103.8 (20.3 to 219)	11.4 (4.9 to 19.8)	197.6 (71.4 to 365.3)	16 (5.4 to 30.6)	213.6 (76.9 to 394)
Qatar	High body-mass index	1.9 (0.2 to 4.1)	28.9 (-13.9 to 77.3)	1.3 (-0.4 to 3.3)	30.2 (-14.3 to 80.5)	4.2 (1.4 to 7.6)	61.3 (3.2 to 126.3)	4.6 (0.1 to 10.2)	65.9 (3.3 to 136.6)
Qatar	High fasting plasma glucose	3.5 (0.7 to 8.2)	78.2 (16.1 to 182.2)	3.3 (0.7 to 8)	81.5 (16.7 to 189.3)	8.3 (1.9 to 16.9)	157 (36 to 331.5)	13 (2.9 to 28)	170.1 (38.6 to 355.8)
Republic of Korea	All risk factors	1.8 (1.4 to 2.3)	56.3 (43.6 to 69.7)	3.6 (2.3 to 5.2)	59.9 (46.4 to 74.4)	2.3 (1.7 to 3)	63.2 (47.6 to 80.8)	9.1 (5.5 to 13.8)	72.3 (54.1 to 92.6)
Republic of Korea	Behavioral risks	1.5 (1.2 to 1.8)	49.2 (39.5 to 58.9)	3 (1.9 to 4.3)	52.1 (41.8 to 62.1)	1.7 (1.4 to 2.1)	51.1 (40.8 to 62.7)	7.2 (4.5 to 10.7)	58.3 (46.5 to 71.9)
Republic of Korea	Alcohol use	0.9 (0.7 to 1.2)	31.4 (24.9 to 38.9)	1.9 (1.2 to 2.7)	33.3 (26.3 to 41.3)	1.1 (0.8 to 1.4)	32.5 (24.5 to 41.9)	4.6 (2.8 to 7.1)	37 (27.7 to 48.4)
Republic of Korea	Diet high in red meat	0.2 (0 to 0.2)	5.8 (1.4 to 7.8)	0.4 (0.1 to 0.6)	6.2 (1.4 to 8.4)	0.3 (0.1 to 0.5)	10.2 (4.3 to 14.2)	1.5 (0.6 to 2.4)	11.7 (4.9 to 16.4)
Republic of Korea	Low physical activity	0.1 (0 to 0.2)	2 (0.9 to 4.3)	0.1 (0.1 to 0.3)	2.2 (0.9 to 4.7)	0.1 (0 to 0.2)	2.4 (1 to 5.1)	0.4 (0.1 to 0.8)	2.8 (1.1 to 5.9)
Republic of Korea	Smoking	0.1 (0.1 to 0.2)	4.5 (3 to 6.3)	0.3 (0.2 to 0.5)	4.8 (3.2 to 6.7)	0.1 (0.1 to 0.2)	3.7 (2.4 to 5.1)	0.5 (0.3 to 0.8)	4.2 (2.8 to 5.9)
Republic of Korea	Secondhand smoke	0.3 (0.1 to 0.4)	8.7 (2.1 to 14.9)	0.5 (0.1 to 1)	9.2 (2.3 to 15.9)	0.2 (0 to 0.3)	5.8 (1.4 to 10)	0.8 (0.2 to 1.5)	6.6 (1.7 to 11.4)
Republic of Korea	Metabolic risks	0.4 (0.1 to 0.9)	8.8 (0.4 to 20.5)	0.7 (0.1 to 1.7)	9.5 (0.5 to 21.9)	0.7 (0.2 to 1.5)	15 (3.5 to 31.4)	2.3 (0.5 to 5.1)	17.2 (4.2 to 36.1)
Republic of Korea	High body-mass index	0.1 (0 to 0.3)	0.6 (-3.3 to 4)	0.1 (-0.1 to 0.4)	0.8 (-3.3 to 4.5)	0.2 (0 to 0.5)	3.1 (-1.4 to 8.7)	0.5 (-0.2 to 1.3)	3.5 (-1.5 to 9.9)
Republic of Korea	High fasting plasma glucose	0.3 (0.1 to 0.8)	8.4 (1.6 to 19.5)	0.6 (0.1 to 1.5)	9 (1.7 to 20.8)	0.5 (0.1 to 1.2)	12.3 (2.4 to 27.7)	1.9 (0.3 to 4.7)	14.2 (2.8 to 32.1)
Republic of Moldova	All risk factors	5.3 (4.1 to 6.7)	162.7 (124.9 to 200.8)	7 (4.6 to 9.9)	169.7 (130.4 to 210.2)	4.2 (2.9 to 5.8)	112.4 (78.7 to 153.9)	7 (4.3 to 10.6)	119.4 (83.5 to 162.9)
Republic of Moldova	Behavioral risks	4.1 (3.3 to 4.8)	136.7 (111 to 163.6)	5.7 (3.8 to 8)	142.4 (115.5 to 169.8)	2.6 (2 to 3.3)	78.5 (60.2 to 102)	4.8 (3.1 to 7)	83.3 (63.8 to 108.3)
Republic of Moldova	Alcohol use	2.8 (2.2 to 3.4)	95.3 (75.1 to 116.8)	3.9 (2.6 to 5.7)	99.3 (78.2 to 122)	1.7 (1.3 to 2.3)	52.9 (39.5 to 70.6)	3.2 (2 to 4.8)	56.1 (41.8 to 75.1)

Location	Risk factor	1990				2019			
		Deaths	YLLs (Years of Life Lost)	YLDs (Years Lived with Disability)	DALYs (Disability-Adjusted Life Years)	Deaths	YLLs (Years of Life Lost)	YLDs (Years Lived with Disability)	DALYs (Disability-Adjusted Life Years)
Republic of Moldova	Diet high in red meat	0.6 (0.2 to 0.7)	18.5 (5.2 to 24.4)	0.8 (0.2 to 1.2)	19.2 (5.4 to 25.5)	0.3 (0.1 to 0.5)	10.1 (2.3 to 15)	0.6 (0.1 to 1)	10.7 (2.4 to 15.9)
Republic of Moldova	Low physical activity	0.1 (0.1 to 0.2)	3.6 (2.3 to 6.8)	0.2 (0.1 to 0.3)	3.8 (2.4 to 7.2)	0.1 (0.1 to 0.2)	2.9 (1.7 to 5.2)	0.2 (0.1 to 0.4)	3.1 (1.8 to 5.5)
Republic of Moldova	Smoking	0.3 (0.2 to 0.4)	8.7 (5.2 to 13.4)	0.4 (0.2 to 0.6)	9.1 (5.4 to 14)	0.2 (0.2 to 0.3)	6.7 (4.2 to 9.6)	0.4 (0.2 to 0.7)	7.2 (4.5 to 10.3)
Republic of Moldova	Secondhand smoke	0.5 (0.1 to 0.9)	19.1 (4.6 to 32.9)	0.8 (0.2 to 1.4)	19.9 (4.7 to 34.3)	0.3 (0.1 to 0.6)	10.2 (2.3 to 18.3)	0.6 (0.1 to 1.1)	10.8 (2.5 to 19.2)
Republic of Moldova	Metabolic risks	1.5 (0.4 to 2.9)	31.3 (-1.3 to 69.6)	1.6 (0.2 to 3.4)	32.9 (-1.2 to 72.8)	1.9 (0.7 to 3.4)	40.2 (10.4 to 77.8)	2.7 (0.8 to 5.3)	42.8 (11.4 to 82.4)
Republic of Moldova	High body-mass index	0.7 (0 to 1.6)	8.7 (-13.2 to 31.6)	0.6 (-0.4 to 1.6)	9.2 (-13.7 to 33.7)	1.1 (0.2 to 2)	19.1 (-1.5 to 41.7)	1.3 (0 to 2.8)	20.4 (-1.6 to 44.1)
Republic of Moldova	High fasting plasma glucose	0.9 (0.2 to 2)	23.7 (4.5 to 54.3)	1.1 (0.2 to 2.6)	24.8 (4.7 to 56.7)	0.9 (0.2 to 2.2)	23 (4.3 to 55.4)	1.5 (0.3 to 3.7)	24.5 (4.6 to 59)
Romania	All risk factors	4.5 (3.5 to 5.6)	128.8 (99.6 to 157.4)	5.7 (3.8 to 8.1)	134.5 (103.7 to 163.1)	5.5 (4.1 to 7.4)	140.9 (104.4 to 189.1)	9.5 (6 to 14.3)	150.4 (111.5 to 201.7)
Romania	Behavioral risks	3.5 (2.8 to 4.1)	110.1 (89 to 129.7)	4.7 (3.1 to 6.7)	114.8 (92.9 to 135.1)	3.9 (2.9 to 5)	110.4 (83 to 142.4)	7.5 (4.8 to 11.1)	117.9 (88.5 to 151.6)
Romania	Alcohol use	1.5 (1.2 to 1.9)	49.9 (37.7 to 62)	2.1 (1.3 to 3.1)	52 (39.4 to 64.5)	1.7 (1.2 to 2.3)	49.9 (35.6 to 67.3)	3.4 (2.1 to 5.2)	53.3 (37.8 to 71.8)
Romania	Diet high in red meat	0.7 (0.3 to 0.9)	21 (8.2 to 27.6)	0.9 (0.3 to 1.4)	21.9 (8.5 to 28.9)	1 (0.5 to 1.4)	27.3 (13.2 to 40.7)	1.9 (0.8 to 3.1)	29.1 (14.1 to 43.5)
Romania	Low physical activity	0.2 (0.1 to 0.3)	4.7 (2.6 to 8.2)	0.2 (0.1 to 0.4)	5 (2.7 to 8.7)	0.3 (0.1 to 0.5)	5.7 (2.8 to 10.5)	0.4 (0.2 to 0.8)	6.1 (3 to 11.3)
Romania	Smoking	0.9 (0.6 to 1.3)	29 (18.8 to 40.3)	1.2 (0.7 to 2)	30.2 (19.5 to 41.9)	0.8 (0.5 to 1.2)	24.8 (15.6 to 35.3)	1.7 (0.9 to 2.7)	26.5 (16.6 to 37.8)
Romania	Secondhand smoke	0.5 (0.1 to 0.8)	14.9 (3.5 to 26.2)	0.6 (0.1 to 1.2)	15.5 (3.7 to 27.2)	0.4 (0.1 to 0.8)	12.9 (3.1 to 22.9)	0.9 (0.2 to 1.7)	13.7 (3.4 to 24.5)
Romania	Metabolic risks	1.3 (0.4 to 2.4)	23.6 (0.6 to 51.2)	1.2 (0.1 to 2.5)	24.8 (1 to 53.4)	2.1 (0.8 to 3.9)	38.9 (8.9 to 80.1)	2.5 (0.4 to 5.3)	41.5 (9.2 to 85.5)
Romania	High body-mass index	0.7 (0 to 1.4)	9.1 (-7.9 to 26.5)	0.5 (-0.3 to 1.4)	9.6 (-8.1 to 27.9)	1.1 (0.2 to 2.2)	17.2 (-4.3 to 41.8)	1 (-0.4 to 2.7)	18.3 (-4.9 to 44.4)
Romania	High fasting plasma glucose	0.6 (0.1 to 1.4)	15.4 (2.8 to 35.9)	0.7 (0.1 to 1.8)	16.1 (3 to 37.8)	1 (0.2 to 2.4)	23.5 (4.1 to 54.9)	1.6 (0.3 to 4)	25.1 (4.4 to 58.5)
Russian Federation	All risk factors	3.7 (2.8 to 4.6)	108.7 (82.2 to 133.8)	5.8 (3.8 to 8.2)	114.6 (86.7 to 140.4)	4.8 (3.3 to 6.5)	126.2 (90.2 to 170.1)	9.9 (6.2 to 14.8)	136.1 (97.9 to 182.3)
Russian Federation	Behavioral risks	2.8 (2.2 to 3.3)	92.5 (74.8 to 109.1)	4.9 (3.3 to 6.8)	97.4 (78.2 to 114.3)	3.2 (2.4 to 4)	96.3 (72.4 to 124.3)	7.6 (4.9 to 11.4)	103.9 (78.4 to 132.5)
Russian Federation	Alcohol use	1.3 (1 to 1.6)	46.3 (36.8 to 55.9)	2.4 (1.6 to 3.5)	48.7 (38.4 to 58.7)	1.6 (1.1 to 2.1)	50.7 (36.6 to 67)	4 (2.5 to 6.2)	54.8 (40.1 to 71.6)
Russian Federation	Diet high in red meat	0.8 (0.4 to 1)	24.2 (12.2 to 32.8)	1.3 (0.6 to 2)	25.5 (12.8 to 34.5)	0.7 (0.3 to 1)	19.1 (8 to 28.1)	1.5 (0.6 to 2.5)	20.6 (8.6 to 30.4)
Russian Federation	Low physical activity	0.1 (0.1 to 0.2)	2.8 (1.8 to 5.1)	0.2 (0.1 to 0.3)	3 (1.9 to 5.4)	0.2 (0.1 to 0.3)	3.3 (1.9 to 6)	0.3 (0.1 to 0.5)	3.6 (2.1 to 6.4)
Russian Federation	Smoking	0.3 (0.2 to 0.5)	10.9 (6.1 to 16.5)	0.6 (0.3 to 1)	11.5 (6.4 to 17.5)	0.6 (0.3 to 0.8)	17.3 (10.1 to 25.3)	1.4 (0.7 to 2.3)	18.7 (11 to 27)
Russian Federation	Secondhand smoke	0.5 (0.1 to 0.8)	15.5 (3.9 to 26.7)	0.8 (0.2 to 1.5)	16.3 (4 to 28.1)	0.4 (0.1 to 0.8)	13.6 (3.2 to 25.1)	1.1 (0.2 to 2)	14.7 (3.4 to 26.7)
Russian Federation	Metabolic risks	1.1 (0.3 to 2)	19 (-2.4 to 43)	1.1 (-0.1 to 2.4)	20.1 (-2.5 to 45.4)	1.9 (0.7 to 3.4)	36 (6.6 to 71.7)	2.7 (0.4 to 5.6)	38.7 (7.1 to 76.8)
Russian Federation	High body-mass index	0.6 (0 to 1.4)	7.8 (-9.6 to 25)	0.5 (-0.5 to 1.4)	8.3 (-10.1 to 26.5)	1.2 (0.3 to 2.5)	19.9 (-3.8 to 47.6)	1.4 (-0.3 to 3.5)	21.4 (-4.3 to 50.4)
Russian Federation	High fasting plasma glucose	0.5 (0.1 to 1.1)	11.8 (2.1 to 27.4)	0.7 (0.1 to 1.6)	12.5 (2.2 to 29)	0.8 (0.1 to 1.8)	17.6 (3.2 to 42)	1.4 (0.2 to 3.6)	19 (3.4 to 44.9)
Rwanda	All risk factors	3.7 (2.4 to 5.2)	105.3 (70 to 151.4)	2.2 (1.3 to 3.4)	107.6 (71.4 to 154.9)	3.9 (2.6 to 5.6)	94.9 (62.5 to 138.3)	2.8 (1.6 to 4.4)	97.6 (64.4 to 142.1)
Rwanda	Behavioral risks	2.9 (2 to 3.9)	89.2 (60.6 to 124.7)	1.8 (1.1 to 2.7)	91.1 (62 to 127.2)	2.7 (1.9 to 3.7)	72.9 (50.5 to 103.6)	2.1 (1.2 to 3.2)	75 (52 to 106.7)
Rwanda	Alcohol use	1.9 (1.3 to 2.7)	63.2 (40.9 to 89.7)	1.3 (0.7 to 1.9)	64.4 (41.6 to 91.6)	1.5 (1 to 2.2)	46.4 (30.2 to 67)	1.3 (0.7 to 2.1)	47.7 (30.9 to 68.8)
Rwanda	Diet high in red meat	0.2 (0 to 0.3)	5.2 (1.4 to 8.6)	0.1 (0 to 0.2)	5.3 (1.5 to 8.7)	0.2 (0 to 0.3)	5.9 (1.3 to 9.8)	0.2 (0 to 0.3)	6 (1.3 to 10)
Rwanda	Low physical activity	0.1 (0.1 to 0.2)	2.5 (1.5 to 6.4)	0.1 (0 to 0.1)	2.6 (1.5 to 6.5)	0.1 (0.1 to 0.2)	2.2 (1.3 to 5)	0.1 (0 to 0.1)	2.3 (1.4 to 5.1)
Rwanda	Smoking	0.5 (0.2 to 0.7)	9.3 (4.5 to 15.1)	0.2 (0.1 to 0.4)	9.5 (4.6 to 15.5)	0.7 (0.4 to 1)	12.5 (6.8 to 19.2)	0.4 (0.2 to 0.7)	12.9 (6.9 to 19.8)
Rwanda	Secondhand smoke	0.4 (0.1 to 0.7)	12.2 (2.7 to 24.1)	0.2 (0.1 to 0.5)	12.4 (2.7 to 24.5)	0.3 (0.1 to 0.5)	8.8 (2.1 to 16.7)	0.2 (0.1 to 0.5)	9 (2.2 to 17.1)
Rwanda	Metabolic risks	0.9 (0.2 to 2)	18.5 (0.5 to 44.9)	0.5 (0.1 to 1.1)	19 (0.6 to 46)	1.4 (0.4 to 2.8)	25.4 (4.7 to 56.2)	0.8 (0.2 to 1.7)	26.2 (5 to 57.9)
Rwanda	High body-mass index	0.2 (0 to 0.7)	1.7 (-6.9 to 11.1)	0.1 (-0.1 to 0.3)	1.8 (-6.9 to 11.3)	0.5 (0.1 to 1.3)	7.4 (-5 to 22.9)	0.3 (-0.1 to 0.7)	7.7 (-5 to 23.5)
Rwanda	High fasting plasma glucose	0.7 (0.1 to 1.7)	17 (2.9 to 42.5)	0.4 (0.1 to 1)	17.4 (2.9 to 43.5)	0.9 (0.2 to 2.1)	18.7 (3.2 to 45.8)	0.6 (0.1 to 1.4)	19.2 (3.3 to 47.1)
Saint Kitts and Nevis	All risk factors	9.4 (5.6 to 14.1)	227.9 (135.8 to 343.4)	10.9 (6 to 17.3)	238.8 (142.8 to 360.8)	7.4 (4.1 to 12)	163.9 (87.8 to 267.5)	11.5 (5.8 to 20)	175.4 (94.3 to 287.1)
Saint Kitts and Nevis	Behavioral risks	4.6 (2.9 to 5.9)	138.9 (82.3 to 178.2)	6.2 (3.4 to 8.9)	145.1 (86.1 to 186.9)	2.8 (1.7 to 4.2)	72.1 (40.1 to 116)	4.9 (2.5 to 8)	77 (42.5 to 122.4)
Saint Kitts and Nevis	Alcohol use	1.7 (0.1 to 2.5)	59.9 (4.1 to 84.6)	2.6 (0.2 to 4)	62.5 (4.2 to 88.2)	0.8 (0 to 1.7)	23.5 (0 to 53.6)	1.5 (0 to 3.7)	25.1 (0 to 57.3)

Location	Risk factor	1990				2019			
		Deaths	YLLs (Years of Life Lost)	YLDs (Years Lived with Disability)	DALYs (Disability-Adjusted Life Years)	Deaths	YLLs (Years of Life Lost)	YLDs (Years Lived with Disability)	DALYs (Disability-Adjusted Life Years)
Saint Kitts and Nevis	Diet high in red meat	1 (0.2 to 1.4)	29.6 (6.8 to 40.8)	1.3 (0.3 to 2.1)	30.9 (7.2 to 42.7)	0.7 (0.2 to 1.1)	18.5 (4.1 to 30.4)	1.2 (0.3 to 2.2)	19.8 (4.4 to 32.3)
Saint Kitts and Nevis	Low physical activity	0.7 (0.3 to 1.4)	16.7 (6.1 to 33.3)	0.8 (0.3 to 1.6)	17.5 (6.4 to 34.9)	0.6 (0.2 to 1.1)	12.7 (4.2 to 24.7)	0.9 (0.3 to 1.9)	13.6 (4.5 to 26.5)
Saint Kitts and Nevis	Smoking	0.5 (0.3 to 0.7)	12.2 (7.6 to 18.1)	0.6 (0.3 to 0.9)	12.8 (8.1 to 19.1)	0.4 (0.2 to 0.5)	8.3 (5.2 to 12.5)	0.6 (0.3 to 1)	8.9 (5.6 to 13.6)
Saint Kitts and Nevis	Secondhand smoke	0.8 (0.2 to 1.5)	26.3 (5.6 to 46.7)	1.2 (0.3 to 2.2)	27.4 (6 to 48.6)	0.4 (0.1 to 0.8)	11.8 (2.5 to 22.7)	0.8 (0.2 to 1.6)	12.6 (2.7 to 24.2)
Saint Kitts and Nevis	Metabolic risks	5.3 (1.7 to 10.6)	98.6 (13.6 to 218.5)	5.2 (1.1 to 11.4)	103.8 (14.6 to 229)	5.1 (1.8 to 9.9)	101.2 (30.4 to 203.2)	7.3 (2.1 to 14.9)	108.5 (32.6 to 216.3)
Saint Kitts and Nevis	High body-mass index	1.4 (-0.1 to 3.3)	10.6 (-31.8 to 50)	0.9 (-0.9 to 3)	11.5 (-32.2 to 53.1)	1.8 (0.4 to 3.8)	31.6 (-4.2 to 75.1)	2.4 (0 to 5.5)	34 (-4.3 to 81)
Saint Kitts and Nevis	High fasting plasma glucose	4.1 (0.8 to 8.9)	92.5 (18.5 to 204.2)	4.5 (0.9 to 10.5)	97 (19.5 to 214.4)	3.5 (0.8 to 7.8)	75.2 (16 to 171.7)	5.3 (1.1 to 13)	80.5 (17.1 to 185.9)
Saint Lucia	All risk factors	7.7 (5.1 to 11.3)	203 (135.7 to 286.1)	8.1 (4.7 to 12.5)	211.1 (141 to 297.4)	6.5 (3.9 to 10.2)	160.4 (98.9 to 249.5)	9 (5 to 15)	169.4 (104.7 to 262.6)
Saint Lucia	Behavioral risks	4.4 (3.4 to 5.2)	134.2 (105.4 to 161.3)	5.1 (3.4 to 7.1)	139.3 (109.7 to 167.6)	3.2 (2.4 to 4)	95.2 (71.1 to 119.7)	5.1 (3.3 to 7.4)	100.3 (74.6 to 126.4)
Saint Lucia	Alcohol use	2.3 (1.8 to 2.9)	76.5 (59.5 to 96.4)	2.8 (1.8 to 4.1)	79.4 (61.9 to 100.1)	1.6 (1.2 to 2.1)	53.8 (38.8 to 71.7)	2.8 (1.8 to 4.3)	56.6 (40.9 to 75.3)
Saint Lucia	Diet high in red meat	0.9 (0.3 to 1.2)	27.3 (9.9 to 37.1)	1 (0.3 to 1.7)	28.4 (10.3 to 38.6)	0.7 (0.2 to 1)	19.4 (6.3 to 28.5)	1 (0.3 to 1.7)	20.5 (6.6 to 30.1)
Saint Lucia	Low physical activity	0.4 (0.2 to 0.8)	9.7 (3.6 to 19.1)	0.4 (0.1 to 0.8)	10.1 (3.7 to 19.9)	0.4 (0.1 to 0.7)	8.9 (2.9 to 17.4)	0.5 (0.2 to 1.1)	9.4 (3 to 18.5)
Saint Lucia	Smoking	0.6 (0.4 to 0.8)	15.6 (9.5 to 22.8)	0.6 (0.3 to 1)	16.2 (9.9 to 23.8)	0.4 (0.2 to 0.6)	9.7 (5.9 to 14.4)	0.5 (0.3 to 0.9)	10.2 (6.3 to 15.2)
Saint Lucia	Secondhand smoke	0.4 (0.1 to 0.7)	13 (3 to 22.9)	0.5 (0.1 to 0.9)	13.4 (3.1 to 23.7)	0.3 (0.1 to 0.5)	8.5 (2 to 15.6)	0.4 (0.1 to 0.9)	9 (2.1 to 16.4)
Saint Lucia	Metabolic risks	4 (1.2 to 7.8)	81.4 (14 to 175.4)	3.6 (0.8 to 7.7)	84.9 (14.8 to 181.6)	3.8 (1.1 to 7.5)	76.1 (9.5 to 167.4)	4.5 (0.9 to 10)	80.7 (10.3 to 177.1)
Saint Lucia	High body-mass index	0.7 (-0.1 to 1.8)	6.4 (-16.4 to 31.4)	0.5 (-0.4 to 1.6)	6.8 (-16.8 to 32.7)	0.9 (0 to 2.1)	6.6 (-20.3 to 33.9)	0.6 (-0.8 to 2.2)	7.2 (-21.2 to 35.8)
Saint Lucia	High fasting plasma glucose	3.4 (0.7 to 7.3)	78.1 (16.7 to 171.8)	3.3 (0.7 to 7.4)	81.4 (17.2 to 178.7)	3.2 (0.7 to 6.9)	73.9 (15.5 to 163.5)	4.2 (0.9 to 9.9)	78.1 (16.3 to 172)
Saint Vincent and the Grenadines	All risk factors	6.2 (3.8 to 9.6)	152.4 (94 to 231.5)	6.3 (3.6 to 10.3)	158.7 (98 to 241.1)	8.1 (4.8 to 12.7)	194.5 (114.7 to 302.6)	9.4 (5.1 to 15.7)	203.9 (120.8 to 319.6)
Saint Vincent and the Grenadines	Behavioral risks	3 (2.3 to 3.8)	89.6 (68.1 to 111.9)	3.5 (2.3 to 5)	93.1 (70.9 to 116)	3.7 (2.8 to 4.7)	109.4 (82.2 to 139.6)	5 (3.2 to 7.4)	114.4 (86.5 to 146.6)
Saint Vincent and the Grenadines	Alcohol use	1.3 (1 to 1.7)	44.3 (33.3 to 57.5)	1.7 (1.1 to 2.4)	46 (34.6 to 59.6)	1.8 (1.3 to 2.3)	59.8 (43.7 to 79.2)	2.7 (1.7 to 4)	62.4 (45.9 to 82.6)
Saint Vincent and the Grenadines	Diet high in red meat	0.5 (0.1 to 0.6)	13.3 (2.5 to 18.8)	0.5 (0.1 to 0.9)	13.9 (2.6 to 19.6)	0.6 (0.1 to 0.9)	17.5 (3.6 to 25.6)	0.8 (0.2 to 1.3)	18.3 (3.8 to 26.9)
Saint Vincent and the Grenadines	Low physical activity	0.5 (0.2 to 0.8)	10 (3.8 to 19.9)	0.4 (0.2 to 0.9)	10.5 (4 to 20.8)	0.6 (0.2 to 1)	12.7 (4 to 24.1)	0.6 (0.2 to 1.3)	13.3 (4.2 to 25.5)
Saint Vincent and the Grenadines	Smoking	0.4 (0.3 to 0.6)	10.9 (6.7 to 15.9)	0.5 (0.3 to 0.7)	11.4 (6.9 to 16.6)	0.4 (0.3 to 0.6)	10.2 (5.9 to 15.4)	0.5 (0.3 to 0.8)	10.7 (6.2 to 16.1)
Saint Vincent and the Grenadines	Secondhand smoke	0.5 (0.1 to 0.9)	14.5 (3.2 to 26.2)	0.6 (0.1 to 1.1)	15.1 (3.4 to 27.2)	0.5 (0.1 to 0.8)	14.1 (3.3 to 25.5)	0.6 (0.1 to 1.2)	14.8 (3.5 to 26.6)
Saint Vincent and the Grenadines	Metabolic risks	3.5 (1 to 7.1)	69.8 (11.7 to 154.9)	3.1 (0.7 to 7.1)	73 (12.1 to 160.5)	4.9 (1.6 to 9.9)	96.3 (16.7 to 213.3)	5 (1.1 to 10.8)	101.3 (17.8 to 222.7)
Saint Vincent and the Grenadines	High body-mass index	0.7 (-0.1 to 1.7)	5.9 (-15 to 27.5)	0.4 (-0.4 to 1.4)	6.3 (-15.4 to 28.8)	1.2 (0 to 2.8)	9.8 (-24.5 to 45)	0.8 (-0.9 to 2.5)	10.6 (-25.6 to 46.9)
Saint Vincent and the Grenadines	High fasting plasma glucose	2.9 (0.6 to 6.5)	66.1 (13.3 to 150)	2.8 (0.6 to 6.7)	69 (13.8 to 155.4)	4 (0.8 to 8.6)	91.5 (18.7 to 202.8)	4.5 (0.9 to 10.3)	96 (19.6 to 212.8)
Samoa	All risk factors	7.8 (4.4 to 11.7)	215.3 (121.7 to 321.7)	6.9 (3.6 to 10.9)	222.2 (125.9 to 331.5)	10.2 (5.4 to 17.8)	279.4 (152.3 to 475.7)	10.7 (5.4 to 18.8)	290.1 (158.3 to 493.9)
Samoa	Behavioral risks	2.8 (1.8 to 4)	83.1 (52.6 to 122.5)	2.6 (1.6 to 4.1)	85.7 (54.3 to 126.3)	3.3 (1.9 to 5.6)	97.3 (55.4 to 164.5)	3.7 (2 to 6.5)	101 (57.5 to 171.2)
Samoa	Alcohol use	0.2 (0.1 to 0.4)	8.6 (4.6 to 14.1)	0.3 (0.1 to 0.5)	8.9 (4.8 to 14.6)	0.3 (0.1 to 0.7)	12.2 (5.4 to 24.2)	0.5 (0.2 to 1)	12.7 (5.6 to 25)
Samoa	Diet high in red meat	0.6 (0.2 to 0.9)	18.4 (5.8 to 29.6)	0.6 (0.2 to 1)	19 (6.1 to 30.4)	0.7 (0.2 to 1.3)	22.1 (6.5 to 41.4)	0.8 (0.2 to 1.7)	22.9 (6.7 to 42.8)
Samoa	Low physical activity	0.4 (0.1 to 0.7)	9.8 (3.3 to 19.7)	0.3 (0.1 to 0.7)	10.1 (3.4 to 20.4)	0.5 (0.1 to 1)	12.2 (3.7 to 26.4)	0.5 (0.1 to 1.1)	12.6 (3.8 to 27.3)
Samoa	Smoking	0.8 (0.5 to 1.3)	23.9 (13.9 to 37.6)	0.8 (0.4 to 1.2)	24.6 (14.3 to 38.7)	1 (0.6 to 1.7)	27.1 (15.5 to 47.6)	1 (0.5 to 1.8)	28.1 (16.2 to 49.7)
Samoa	Secondhand smoke	0.9 (0.2 to 1.6)	27.1 (6 to 51.4)	0.8 (0.2 to 1.6)	27.9 (6.2 to 53.1)	0.9 (0.2 to 2.1)	29.3 (6.4 to 64.9)	1.1 (0.2 to 2.4)	30.4 (6.6 to 67.5)
Samoa	Metabolic risks	5.8 (2.4 to 9.7)	153.8 (60.8 to 255.8)	5 (1.9 to 8.8)	158.7 (62.8 to 263.1)	8 (3.4 to 15)	211.3 (90.9 to 397.2)	8.1 (3.3 to 15.6)	219.5 (94.4 to 411.2)
Samoa	High body-mass index	4.6 (1.4 to 8.3)	122.3 (39.8 to 219.7)	3.9 (1.2 to 7.4)	126.2 (41.2 to 227.5)	5.7 (1.7 to 11.5)	148.4 (45.7 to 296.4)	5.7 (1.7 to 11.6)	154.1 (47.4 to 305.7)
Samoa	High fasting plasma glucose	1.6 (0.3 to 3.8)	41.9 (8 to 101.2)	1.4 (0.2 to 3.4)	43.2 (8.2 to 105.3)	3.2 (0.7 to 8.4)	84 (17.9 to 219)	3.3 (0.7 to 8.2)	87.3 (18.6 to 226.3)
San Marino	All risk factors	6.4 (4.2 to 8.4)	152.8 (96.3 to 205.6)	15 (8.8 to 22.6)	167.8 (106.4 to 223.7)	6.4 (3.6 to 10.3)	152.4 (81.5 to 255.1)	19.6 (10.8 to 31.8)	172 (94 to 284.9)

Location	Risk factor	1990				2019			
		Deaths	YLLs (Years of Life Lost)	YLDs (Years Lived with Disability)	DALYs (Disability-Adjusted Life Years)	Deaths	YLLs (Years of Life Lost)	YLDs (Years Lived with Disability)	DALYs (Disability-Adjusted Life Years)
San Marino	Behavioral risks	4.9 (3 to 6.6)	129.2 (76.3 to 175.3)	12.5 (6.7 to 19.2)	141.7 (83.4 to 191.3)	4.5 (2.3 to 7.4)	121.2 (60.5 to 201.2)	15.5 (8 to 24.9)	136.7 (70.1 to 224.2)
San Marino	Alcohol use	2.3 (0.2 to 3.5)	61.7 (5.2 to 94)	5.9 (0.5 to 10.1)	67.6 (5.7 to 103.2)	2.3 (0.3 to 4.1)	63.1 (7 to 114.1)	8.1 (0.9 to 14.3)	71.2 (7.8 to 126.3)
San Marino	Diet high in red meat	1 (0.5 to 1.5)	26.6 (12.5 to 39.3)	2.6 (1.2 to 4.2)	29.2 (13.7 to 43.4)	1 (0.4 to 1.8)	26.4 (11.4 to 48.4)	3.4 (1.5 to 5.9)	29.8 (13.1 to 53.6)
San Marino	Low physical activity	0.4 (0.1 to 0.7)	7.5 (2.4 to 15.1)	0.8 (0.2 to 1.7)	8.3 (2.6 to 16.7)	0.4 (0.1 to 0.8)	7.9 (2.3 to 16.9)	1 (0.3 to 2.2)	8.9 (2.7 to 19)
San Marino	Smoking	1.4 (0.9 to 1.9)	37.4 (24.4 to 52.8)	3.6 (1.9 to 5.6)	40.9 (26.7 to 57.8)	1 (0.5 to 1.6)	27.2 (14.8 to 47.5)	3.5 (1.8 to 5.7)	30.7 (17.1 to 52.8)
San Marino	Secondhand smoke	0.4 (0.1 to 0.6)	10.3 (2.4 to 18.8)	1 (0.2 to 1.8)	11.3 (2.6 to 20.3)	0.3 (0.1 to 0.6)	8.5 (1.9 to 17.5)	1.1 (0.2 to 2.1)	9.6 (2.1 to 19.6)
San Marino	Metabolic risks	1.9 (0.7 to 3.6)	31.4 (7.3 to 64)	3.3 (0.8 to 6.6)	34.7 (8.3 to 70)	2.4 (0.8 to 5.3)	41 (7.8 to 100.1)	5.3 (1.1 to 11.7)	46.3 (9 to 111.6)
San Marino	High body-mass index	1 (0.2 to 2.2)	15.1 (-2.1 to 36.3)	1.6 (0 to 3.6)	16.7 (-2 to 40)	1.1 (0.2 to 2.5)	14.4 (-4.4 to 42.7)	1.8 (-0.6 to 5.1)	16.2 (-5.2 to 47.1)
San Marino	High fasting plasma glucose	0.9 (0.2 to 2.2)	17.5 (3.1 to 42.6)	1.8 (0.3 to 4.4)	19.3 (3.4 to 45.9)	1.4 (0.3 to 3.8)	28.5 (4.8 to 78.2)	3.7 (0.6 to 9.8)	32.3 (5.7 to 87.7)
Sao Tome and Principe	All risk factors	1.8 (1.1 to 2.8)	44.9 (27.4 to 68.4)	1.2 (0.7 to 2)	46.1 (28.1 to 70.1)	4 (2.1 to 7.4)	93.9 (48.5 to 173.1)	3.2 (1.6 to 5.8)	97.1 (50.2 to 180)
Sao Tome and Principe	Behavioral risks	1 (0.7 to 1.4)	30.2 (19.9 to 41.9)	0.8 (0.4 to 1.2)	31 (20.4 to 43.1)	2 (1.2 to 3.1)	58.7 (34 to 89.2)	1.9 (1 to 3.1)	60.6 (35.3 to 91.8)
Sao Tome and Principe	Alcohol use	0.7 (0.5 to 1)	21.1 (13.4 to 30.6)	0.5 (0.3 to 0.9)	21.7 (13.7 to 31.4)	1.5 (0.8 to 2.3)	43.4 (24.5 to 68.1)	1.4 (0.7 to 2.3)	44.8 (25.1 to 70.2)
Sao Tome and Principe	Diet high in red meat	0.1 (0 to 0.1)	2.2 (0.9 to 3.3)	0.1 (0 to 0.1)	2.3 (0.9 to 3.4)	0.1 (0 to 0.2)	3.9 (1.3 to 7.1)	0.1 (0 to 0.2)	4 (1.4 to 7.3)
Sao Tome and Principe	Low physical activity	0.1 (0.1 to 0.2)	2.4 (1.3 to 4.4)	0.1 (0 to 0.1)	2.5 (1.4 to 4.6)	0.2 (0.1 to 0.4)	4.4 (2 to 8.9)	0.1 (0.1 to 0.3)	4.5 (2.1 to 9.3)
Sao Tome and Principe	Smoking	0.1 (0 to 0.1)	1.3 (0.6 to 2.3)	0 (0 to 0.1)	1.4 (0.6 to 2.3)	0.1 (0.1 to 0.2)	3 (1.3 to 5.5)	0.1 (0 to 0.2)	3.1 (1.3 to 5.6)
Sao Tome and Principe	Secondhand smoke	0.1 (0 to 0.2)	3.8 (0.8 to 7.4)	0.1 (0 to 0.2)	3.9 (0.8 to 7.6)	0.2 (0 to 0.4)	5.5 (1.2 to 11.8)	0.2 (0 to 0.4)	5.6 (1.2 to 12.2)
Sao Tome and Principe	Metabolic risks	0.9 (0.3 to 1.8)	16 (2.1 to 36)	0.5 (0.1 to 1.1)	16.5 (2.2 to 37.1)	2.1 (0.6 to 5)	39.2 (5.2 to 101.6)	1.4 (0.3 to 3.5)	40.6 (5.5 to 105.2)
Sao Tome and Principe	High body-mass index	0.3 (0 to 0.7)	4 (-4 to 13.1)	0.2 (-0.1 to 0.5)	4.2 (-4 to 13.6)	0.8 (0.1 to 2)	10.2 (-8.5 to 35)	0.4 (-0.2 to 1.3)	10.6 (-8.8 to 36.2)
Sao Tome and Principe	High fasting plasma glucose	0.6 (0.1 to 1.4)	12.5 (2.3 to 30.4)	0.4 (0.1 to 0.9)	12.8 (2.3 to 31.3)	1.4 (0.3 to 4)	30.7 (5.8 to 85.7)	1.1 (0.2 to 2.8)	31.8 (6 to 88.7)
Saudi Arabia	All risk factors	1.8 (0.9 to 3.1)	43.2 (20.1 to 75.8)	1.5 (0.7 to 2.8)	44.8 (21 to 77.9)	3.5 (1.8 to 5.8)	79.2 (35.8 to 137.5)	5.3 (2.2 to 9.7)	84.5 (38.2 to 146.5)
Saudi Arabia	Behavioral risks	0.8 (0.5 to 1.3)	25.7 (15.2 to 39.9)	0.8 (0.5 to 1.3)	26.5 (15.7 to 41)	1.3 (0.8 to 2)	38 (22.3 to 57.4)	2.6 (1.4 to 4.3)	40.6 (23.6 to 61.3)
Saudi Arabia	Alcohol use	0 (0 to 0.1)	1.6 (0.5 to 3)	0 (0 to 0.1)	1.6 (0.5 to 3.1)	0 (0 to 0.1)	1.1 (0.3 to 2.2)	0.1 (0 to 0.2)	1.2 (0.3 to 2.3)
Saudi Arabia	Diet high in red meat	0.2 (0 to 0.3)	6.1 (1.1 to 10.1)	0.2 (0 to 0.3)	6.3 (1.2 to 10.3)	0.2 (0 to 0.4)	7.3 (1.4 to 11.6)	0.5 (0.1 to 0.9)	7.8 (1.5 to 12.4)
Saudi Arabia	Low physical activity	0.3 (0.1 to 0.6)	8.3 (2.7 to 15.6)	0.3 (0.1 to 0.5)	8.5 (2.8 to 16)	0.6 (0.2 to 1)	15.5 (5.1 to 27.8)	1.1 (0.3 to 2)	16.6 (5.4 to 30.1)
Saudi Arabia	Smoking	0.1 (0 to 0.1)	1.5 (0.8 to 2.5)	0 (0 to 0.1)	1.6 (0.8 to 2.5)	0.1 (0.1 to 0.1)	2.3 (1.3 to 3.6)	0.2 (0.1 to 0.3)	2.4 (1.4 to 3.8)
Saudi Arabia	Secondhand smoke	0.3 (0.1 to 0.5)	9.1 (2.1 to 17.9)	0.3 (0.1 to 0.5)	9.3 (2.2 to 18.5)	0.4 (0.1 to 0.8)	13.1 (2.9 to 24)	0.9 (0.2 to 1.7)	13.9 (3.2 to 25.6)
Saudi Arabia	Metabolic risks	1 (0.2 to 2.2)	19.1 (-2.6 to 49.3)	0.8 (0.1 to 1.8)	19.9 (-2.5 to 51.1)	2.4 (0.7 to 4.8)	45.5 (0.6 to 104.5)	3 (-0.1 to 7)	48.5 (0.3 to 110.8)
Saudi Arabia	High body-mass index	0.3 (-0.1 to 0.8)	1.3 (-12.8 to 13.5)	0.2 (-0.2 to 0.6)	1.4 (-13 to 13.9)	0.9 (-0.1 to 1.9)	7.3 (-20.3 to 33.2)	0.3 (-1.7 to 2)	7.6 (-21.9 to 35.1)
Saudi Arabia	High fasting plasma glucose	0.7 (0.1 to 1.8)	18.7 (3.4 to 45.3)	0.7 (0.1 to 1.7)	19.4 (3.5 to 46.9)	1.7 (0.3 to 3.9)	41.8 (8 to 97.1)	2.9 (0.5 to 6.8)	44.7 (8.6 to 104)
Senegal	All risk factors	2.3 (1.2 to 3.7)	54.7 (29.5 to 88.7)	1.4 (0.7 to 2.5)	56.1 (30.3 to 90.9)	3.5 (1.8 to 6.1)	78.5 (37.9 to 135.9)	2.3 (1 to 4.3)	80.9 (39 to 139.5)
Senegal	Behavioral risks	1.1 (0.7 to 1.7)	33.8 (19.1 to 51.2)	0.8 (0.4 to 1.3)	34.6 (19.6 to 52.5)	1.2 (0.7 to 1.9)	35.1 (19.4 to 53.7)	1 (0.5 to 1.6)	36.1 (20 to 55.2)
Senegal	Alcohol use	0.2 (0.1 to 0.2)	5.4 (3.3 to 7.9)	0.1 (0.1 to 0.2)	5.5 (3.4 to 8.1)	0.1 (0.1 to 0.2)	4.7 (2.6 to 7.4)	0.1 (0.1 to 0.2)	4.8 (2.6 to 7.6)
Senegal	Diet high in red meat	0.3 (0.1 to 0.4)	7.8 (1.5 to 12)	0.2 (0 to 0.3)	8 (1.5 to 12.3)	0.3 (0.1 to 0.5)	9.5 (1.8 to 15.7)	0.3 (0 to 0.5)	9.7 (1.9 to 16.2)
Senegal	Low physical activity	0.1 (0.1 to 0.3)	3.5 (1.8 to 6.2)	0.1 (0 to 0.2)	3.6 (1.9 to 6.4)	0.2 (0.1 to 0.4)	4.6 (2.3 to 8.7)	0.1 (0.1 to 0.3)	4.7 (2.4 to 8.9)
Senegal	Smoking	0.1 (0 to 0.1)	1.5 (0.7 to 2.6)	0 (0 to 0.1)	1.5 (0.7 to 2.7)	0.1 (0 to 0.1)	1.7 (0.7 to 2.9)	0 (0 to 0.1)	1.7 (0.8 to 3)
Senegal	Secondhand smoke	0.6 (0.1 to 1)	16.5 (4 to 30.1)	0.4 (0.1 to 0.7)	16.9 (4.1 to 30.9)	0.5 (0.1 to 1)	15.4 (3.2 to 28.6)	0.4 (0.1 to 0.8)	15.8 (3.3 to 29.3)
Senegal	Metabolic risks	1.2 (0.3 to 2.6)	22.4 (1.6 to 52.9)	0.7 (0.1 to 1.5)	23.1 (1.7 to 54)	2.4 (0.8 to 4.9)	46.1 (8.8 to 102.2)	1.4 (0.3 to 3.2)	47.6 (9.3 to 105.6)
Senegal	High body-mass index	0.3 (0 to 0.8)	2.5 (-7.6 to 13.4)	0.1 (-0.1 to 0.4)	2.6 (-7.7 to 13.7)	0.7 (0.1 to 1.6)	9.1 (-7.1 to 29.8)	0.3 (-0.1 to 0.9)	9.4 (-7.2 to 30.8)
Senegal	High fasting plasma glucose	0.9 (0.2 to 2.2)	20.5 (3.8 to 49.6)	0.5 (0.1 to 1.4)	21.1 (3.9 to 50.9)	1.8 (0.3 to 4.1)	38.9 (7.2 to 91.7)	1.2 (0.2 to 2.9)	40.1 (7.4 to 94.2)
Serbia	All risk factors	7.2 (5.1 to 9.6)	188.6 (135.3 to 247.2)	9.1 (5.6 to 13.4)	197.8 (141.7 to 258.4)	9 (5.9 to 13.2)	213.3 (142.9 to 317.2)	16.2 (9.7 to 25.7)	229.5 (154.3 to 339.6)

Location	Risk factor	1990				2019			
		Deaths	YLLs (Years of Life Lost)	YLDs (Years Lived with Disability)	DALYs (Disability-Adjusted Life Years)	Deaths	YLLs (Years of Life Lost)	YLDs (Years Lived with Disability)	DALYs (Disability-Adjusted Life Years)
Serbia	Behavioral risks	4.8 (3.8 to 5.9)	142.7 (112.8 to 176.4)	6.7 (4.3 to 9.7)	149.4 (117.8 to 184.2)	5.2 (3.8 to 6.9)	142 (101.9 to 192.4)	10.9 (6.7 to 16.6)	152.9 (110 to 206.4)
Serbia	Alcohol use	1.5 (1 to 2)	48.1 (34 to 64.1)	2.2 (1.4 to 3.4)	50.3 (35.6 to 66.7)	1.5 (1 to 2.1)	43.5 (29.5 to 62.2)	3.3 (2 to 5.3)	46.8 (31.8 to 67)
Serbia	Diet high in red meat	0.8 (0.2 to 1.1)	23.3 (7.4 to 31.8)	1.1 (0.3 to 1.8)	24.4 (7.7 to 33.4)	0.8 (0.2 to 1.3)	22.1 (6.5 to 34.3)	1.7 (0.5 to 2.9)	23.8 (6.9 to 37.4)
Serbia	Low physical activity	0.2 (0.1 to 0.4)	5.6 (3.3 to 9.8)	0.3 (0.1 to 0.5)	5.9 (3.5 to 10.2)	0.3 (0.2 to 0.5)	6.3 (3.5 to 11)	0.5 (0.2 to 0.9)	6.8 (3.7 to 11.8)
Serbia	Smoking	1.9 (1.3 to 2.7)	57.1 (36.6 to 79.8)	2.7 (1.5 to 4.2)	59.8 (38.1 to 83.3)	2.3 (1.6 to 3.2)	62.9 (41.8 to 90.5)	4.8 (2.7 to 7.8)	67.7 (45 to 97.8)
Serbia	Secondhand smoke	0.7 (0.2 to 1.2)	19.7 (4.6 to 33.9)	0.9 (0.2 to 1.8)	20.6 (4.8 to 35.6)	0.7 (0.2 to 1.3)	18.3 (4.6 to 33.5)	1.4 (0.3 to 2.7)	19.7 (4.9 to 36.3)
Serbia	Metabolic risks	2.9 (1.1 to 5.4)	56.7 (12.7 to 113.3)	2.9 (0.7 to 5.8)	59.6 (13.4 to 118.4)	4.6 (1.8 to 8.8)	88.4 (26.8 to 178.8)	6.6 (1.9 to 13.7)	95 (29.2 to 191.8)
Serbia	High body-mass index	1.4 (0.3 to 2.7)	21.8 (-6.3 to 51.7)	1.2 (-0.2 to 2.9)	23 (-6.4 to 54.4)	2.1 (0.6 to 4.2)	35.8 (2.2 to 79.5)	2.5 (0.1 to 6)	38.3 (2.3 to 85.3)
Serbia	High fasting plasma glucose	1.7 (0.3 to 3.8)	37.4 (7 to 87.4)	1.9 (0.3 to 4.5)	39.3 (7.3 to 91.7)	2.8 (0.5 to 6.3)	57.6 (10.9 to 133.3)	4.4 (0.8 to 10.9)	62 (11.7 to 143.8)
Seychelles	All risk factors	4.2 (2.3 to 6.2)	119.7 (67.7 to 176.7)	4.1 (2.2 to 6.5)	123.8 (70 to 181.9)	8.8 (5.1 to 13.3)	233 (138 to 343.8)	11.3 (6 to 17.5)	244.3 (145 to 360.3)
Seychelles	Behavioral risks	1.3 (0.8 to 1.7)	40.6 (25.2 to 56.1)	1.4 (0.8 to 2.1)	41.9 (26.1 to 57.9)	2.4 (1.5 to 3.2)	70.7 (46.2 to 98.4)	3.5 (2 to 5.3)	74.1 (48.3 to 102.5)
Seychelles	Alcohol use	0.3 (0.2 to 0.4)	10.6 (7.1 to 15.1)	0.3 (0.2 to 0.5)	10.9 (7.3 to 15.6)	0.8 (0.5 to 1.2)	26.1 (16.2 to 38)	1.3 (0.7 to 2)	27.4 (17.2 to 39.9)
Seychelles	Diet high in red meat	0.2 (0 to 0.3)	6 (1.4 to 9.2)	0.2 (0 to 0.3)	6.2 (1.4 to 9.5)	0.3 (0.1 to 0.5)	10.1 (2 to 15.8)	0.5 (0.1 to 0.9)	10.6 (2.1 to 16.5)
Seychelles	Low physical activity	0.1 (0.1 to 0.2)	2.5 (1.5 to 4.9)	0.1 (0 to 0.2)	2.6 (1.6 to 5.1)	0.2 (0.1 to 0.4)	4.3 (2.4 to 7.9)	0.2 (0.1 to 0.4)	4.5 (2.5 to 8.2)
Seychelles	Smoking	0.2 (0.1 to 0.3)	5.3 (2.9 to 8.5)	0.2 (0.1 to 0.3)	5.5 (3 to 8.8)	0.3 (0.2 to 0.5)	8.2 (4.8 to 12.7)	0.4 (0.2 to 0.7)	8.6 (5 to 13.3)
Seychelles	Secondhand smoke	0.5 (0.1 to 0.9)	17.4 (3.8 to 30.5)	0.6 (0.1 to 1.1)	18 (4 to 31.6)	0.8 (0.2 to 1.4)	24.6 (6.2 to 44.2)	1.2 (0.3 to 2.3)	25.8 (6.4 to 46.2)
Seychelles	Metabolic risks	3.2 (1.3 to 5.4)	86.5 (37.1 to 145.3)	3 (1.2 to 5.2)	89.5 (38.4 to 150.7)	7.1 (3.4 to 11.8)	179.7 (83.1 to 295.7)	8.7 (3.8 to 14.8)	188.4 (86.9 to 308.5)
Seychelles	High body-mass index	2.5 (0.9 to 4.5)	70.9 (25.2 to 126.5)	2.4 (0.8 to 4.5)	73.2 (25.9 to 130.5)	4.6 (1.6 to 8.4)	124.8 (43.7 to 221.3)	6 (2 to 10.6)	130.8 (45.8 to 230.3)
Seychelles	High fasting plasma glucose	0.8 (0.2 to 1.9)	19.3 (3.6 to 45.7)	0.7 (0.1 to 1.7)	20 (3.7 to 47.5)	3.1 (0.7 to 6.7)	70.3 (14.4 to 154.4)	3.5 (0.7 to 8.2)	73.7 (15.1 to 161.7)
Sierra Leone	All risk factors	2 (1.3 to 2.8)	52.5 (34.2 to 76.2)	1.3 (0.7 to 2)	53.8 (35 to 77.7)	3.1 (1.8 to 4.8)	78.4 (46.5 to 123.5)	2.2 (1.2 to 3.7)	80.6 (48 to 127.4)
Sierra Leone	Behavioral risks	1.5 (1 to 2.2)	44.6 (29.2 to 64.8)	1 (0.6 to 1.6)	45.6 (29.9 to 66.4)	1.9 (1.2 to 2.9)	55.9 (34.4 to 84.2)	1.5 (0.8 to 2.5)	57.4 (35.1 to 86.5)
Sierra Leone	Alcohol use	0.8 (0.5 to 1.2)	25.1 (15.6 to 37)	0.6 (0.3 to 0.9)	25.7 (16 to 37.8)	1 (0.6 to 1.6)	30.4 (18.6 to 47.2)	0.8 (0.5 to 1.4)	31.2 (19 to 48.4)
Sierra Leone	Diet high in red meat	0.1 (0 to 0.1)	2.2 (0.7 to 3.6)	0.1 (0 to 0.1)	2.3 (0.8 to 3.7)	0.1 (0 to 0.2)	4 (1 to 7.1)	0.1 (0 to 0.2)	4.2 (1.1 to 7.3)
Sierra Leone	Low physical activity	0.1 (0.1 to 0.2)	2.4 (1.3 to 4.4)	0.1 (0 to 0.1)	2.4 (1.3 to 4.5)	0.2 (0.1 to 0.3)	3.6 (1.8 to 6.9)	0.1 (0 to 0.2)	3.7 (1.9 to 7)
Sierra Leone	Smoking	0.2 (0.1 to 0.3)	5.7 (2.3 to 9.8)	0.1 (0.1 to 0.3)	5.8 (2.3 to 10)	0.2 (0.1 to 0.3)	5 (2.1 to 9)	0.1 (0.1 to 0.3)	5.2 (2.2 to 9.2)
Sierra Leone	Secondhand smoke	0.4 (0.1 to 0.7)	11.2 (2.6 to 21.2)	0.3 (0.1 to 0.5)	11.4 (2.7 to 21.7)	0.5 (0.1 to 1)	15 (3.4 to 29.7)	0.4 (0.1 to 0.8)	15.4 (3.5 to 30.4)
Sierra Leone	Metabolic risks	0.5 (0.1 to 1)	9.1 (1.6 to 20.8)	0.3 (0.1 to 0.6)	9.3 (1.7 to 21.4)	1.3 (0.4 to 2.7)	25.3 (5.4 to 58.1)	0.8 (0.2 to 1.7)	26.1 (5.6 to 59.5)
Sierra Leone	High body-mass index	0.2 (0 to 0.5)	3.1 (-1.5 to 9.9)	0.1 (0 to 0.3)	3.2 (-1.6 to 10.2)	0.5 (0.1 to 1.1)	8.1 (-3.5 to 23.4)	0.3 (0 to 0.7)	8.4 (-3.6 to 24)
Sierra Leone	High fasting plasma glucose	0.3 (0.1 to 0.8)	6.1 (1.1 to 15.9)	0.2 (0 to 0.4)	6.2 (1.1 to 16.2)	0.8 (0.1 to 2.1)	17.8 (3.1 to 45.4)	0.5 (0.1 to 1.4)	18.4 (3.2 to 46.6)
Singapore	All risk factors	2.9 (1.9 to 4.2)	80.5 (53.6 to 114.3)	6.1 (3.6 to 9.8)	86.6 (57.4 to 123.2)	2.6 (1.7 to 3.7)	65.5 (42.9 to 92.6)	9.3 (5.2 to 15.2)	74.8 (49 to 105.3)
Singapore	Behavioral risks	1.8 (1.3 to 2.1)	55.1 (41 to 66.8)	4 (2.6 to 5.8)	59.1 (44.5 to 72.5)	1.5 (1.1 to 1.8)	42 (31.4 to 51.7)	5.8 (3.6 to 8.8)	47.9 (35.4 to 59.2)
Singapore	Alcohol use	0.5 (0.4 to 0.6)	16.5 (12 to 21.4)	1.2 (0.7 to 1.8)	17.6 (12.8 to 23)	0.4 (0.3 to 0.6)	13.2 (9.6 to 17.6)	1.8 (1.1 to 2.9)	15.1 (10.7 to 19.9)
Singapore	Diet high in red meat	0.5 (0.2 to 0.7)	16.9 (6.3 to 23.1)	1.2 (0.4 to 2)	18.1 (6.8 to 24.6)	0.6 (0.2 to 0.8)	15.5 (6.6 to 21.3)	2.2 (0.9 to 3.6)	17.7 (7.4 to 24.4)
Singapore	Low physical activity	0.2 (0.1 to 0.3)	4 (1.7 to 8.2)	0.3 (0.1 to 0.7)	4.3 (1.8 to 9)	0.2 (0.1 to 0.3)	3.4 (1.3 to 6.8)	0.5 (0.2 to 1.2)	3.9 (1.5 to 7.8)
Singapore	Smoking	0.4 (0.2 to 0.5)	12 (7.6 to 17.2)	0.9 (0.5 to 1.4)	12.9 (8.1 to 18.5)	0.2 (0.2 to 0.3)	6.8 (4.6 to 9.6)	0.9 (0.5 to 1.6)	7.8 (5.2 to 11)
Singapore	Secondhand smoke	0.3 (0.1 to 0.4)	8.5 (2 to 14.9)	0.6 (0.1 to 1.2)	9.1 (2.2 to 16)	0.2 (0 to 0.3)	5.2 (1.3 to 9.2)	0.7 (0.2 to 1.4)	5.9 (1.5 to 10.5)
Singapore	Metabolic risks	1.3 (0.3 to 2.7)	28.7 (5.2 to 63.1)	2.4 (0.5 to 5.4)	31.1 (5.8 to 67.9)	1.3 (0.4 to 2.5)	26.7 (7.4 to 54.3)	3.9 (1.1 to 8.6)	30.6 (8.7 to 62)
Singapore	High body-mass index	0.2 (0 to 0.5)	2.2 (-4 to 9.4)	0.2 (-0.2 to 0.9)	2.4 (-4.2 to 10.2)	0.4 (0.1 to 0.9)	7.5 (-0.8 to 18.5)	1.1 (-0.1 to 2.7)	8.6 (-0.9 to 21.4)
Singapore	High fasting plasma glucose	1.1 (0.2 to 2.4)	27 (5.3 to 60.2)	2.2 (0.4 to 5.2)	29.2 (5.7 to 65.2)	0.9 (0.2 to 2)	19.9 (3.8 to 46.3)	3 (0.5 to 7.3)	22.9 (4.4 to 53)
Slovakia	All risk factors	6.2 (4.8 to 7.6)	172 (134.7 to 209)	8.9 (5.8 to 12.6)	180.9 (142.3 to 219.1)	5.8 (3.8 to 8.4)	139.7 (92.9 to 203.2)	12.2 (7.3 to 19)	151.9 (100.9 to 219.5)

Location	Risk factor	1990				2019			
		Deaths	YLLs (Years of Life Lost)	YLDs (Years Lived with Disability)	DALYs (Disability-Adjusted Life Years)	Deaths	YLLs (Years of Life Lost)	YLDs (Years Lived with Disability)	DALYs (Disability-Adjusted Life Years)
Slovakia	Behavioral risks	4.6 (3.7 to 5.4)	140 (113.9 to 164.6)	7 (4.7 to 9.9)	147 (119.7 to 172.6)	3.6 (2.6 to 5)	98.7 (69.1 to 136.9)	8.6 (5.2 to 13.3)	107.3 (74.9 to 150.3)
Slovakia	Alcohol use	2.1 (1.7 to 2.6)	67.1 (52.4 to 80.9)	3.4 (2.2 to 4.9)	70.5 (54.9 to 84.9)	1.6 (1.1 to 2.3)	46.9 (31.8 to 66.8)	4.1 (2.5 to 6.3)	51 (34.3 to 72.6)
Slovakia	Diet high in red meat	0.8 (0.3 to 1.1)	24.6 (9.9 to 32.6)	1.3 (0.5 to 2)	25.9 (10.4 to 34.3)	0.8 (0.3 to 1.2)	20.8 (8.6 to 32.7)	1.8 (0.7 to 3.1)	22.6 (9.3 to 35.7)
Slovakia	Low physical activity	0.2 (0.1 to 0.3)	4.5 (2.7 to 7.8)	0.3 (0.1 to 0.5)	4.7 (2.8 to 8.2)	0.2 (0.1 to 0.4)	4.3 (2.1 to 7.4)	0.4 (0.2 to 0.7)	4.6 (2.3 to 8.2)
Slovakia	Smoking	1.2 (0.9 to 1.7)	39.4 (26.4 to 54.2)	1.9 (1.1 to 3)	41.4 (27.7 to 56.8)	0.9 (0.6 to 1.3)	24.6 (15.4 to 37.8)	2.1 (1.2 to 3.5)	26.7 (16.7 to 41.1)
Slovakia	Secondhand smoke	0.6 (0.1 to 1)	17.8 (4.4 to 30.4)	0.9 (0.2 to 1.7)	18.7 (4.6 to 32)	0.4 (0.1 to 0.7)	10.7 (2.6 to 19.9)	0.9 (0.2 to 1.8)	11.6 (2.9 to 21.5)
Slovakia	Metabolic risks	2 (0.8 to 3.6)	41 (9.9 to 78.6)	2.3 (0.6 to 4.6)	43.3 (10.7 to 83.1)	2.6 (1 to 4.9)	51.2 (16.3 to 98.5)	4.5 (1.4 to 8.9)	55.7 (17.8 to 107.2)
Slovakia	High body-mass index	1.1 (0.2 to 2.2)	20 (-2.4 to 45.5)	1.2 (0 to 2.6)	21.2 (-2.4 to 47.4)	1.5 (0.4 to 3)	26.3 (4 to 59.5)	2.3 (0.3 to 5.2)	28.6 (4.3 to 64.9)
Slovakia	High fasting plasma glucose	1 (0.2 to 2.3)	22.6 (4.2 to 52)	1.3 (0.2 to 3.1)	23.8 (4.5 to 54.9)	1.3 (0.2 to 3.1)	27.3 (4.8 to 66.3)	2.5 (0.4 to 6.4)	29.8 (5.2 to 72.4)
Slovenia	All risk factors	8.1 (5.8 to 11.2)	210.8 (148.9 to 292.2)	13.3 (8 to 20.2)	224 (159.1 to 311.2)	5.3 (3.5 to 7.6)	119.8 (79 to 172)	12.4 (6.9 to 19)	132.2 (87.8 to 188.9)
Slovenia	Behavioral risks	6.1 (4.4 to 8.3)	174 (121.7 to 240)	11.1 (6.7 to 16.9)	185 (130 to 255.5)	3.4 (2.3 to 4.7)	86.6 (59.8 to 122.7)	9.3 (5.5 to 14.2)	95.9 (65.8 to 135.8)
Slovenia	Alcohol use	3 (2 to 4.2)	84.2 (55.2 to 118.4)	5.4 (3.1 to 8.3)	89.5 (58.9 to 125.7)	1.2 (0.6 to 2)	31.6 (14 to 51.9)	3.4 (1.5 to 6)	35 (15.8 to 58.5)
Slovenia	Diet high in red meat	1.3 (0.6 to 1.9)	36.2 (16.6 to 54.9)	2.3 (1 to 3.8)	38.5 (17.6 to 58.3)	0.8 (0.4 to 1.3)	20.9 (9.5 to 32.8)	2.2 (0.9 to 3.8)	23.1 (10.6 to 36.1)
Slovenia	Low physical activity	0.2 (0.1 to 0.4)	5.1 (2.7 to 9.1)	0.3 (0.2 to 0.6)	5.4 (2.9 to 9.7)	0.2 (0.1 to 0.4)	3.9 (2 to 7.1)	0.4 (0.2 to 0.7)	4.3 (2.2 to 7.9)
Slovenia	Smoking	1.7 (1.1 to 2.5)	49.5 (31.6 to 77)	3.1 (1.7 to 5.3)	52.6 (33.6 to 81.8)	1.1 (0.7 to 1.6)	29.6 (19.1 to 43.6)	3.2 (1.8 to 5.2)	32.8 (21.1 to 48.6)
Slovenia	Secondhand smoke	0.6 (0.1 to 1.1)	17.1 (3.9 to 31.5)	1.1 (0.2 to 2.1)	18.2 (4.1 to 33.5)	0.3 (0.1 to 0.5)	7.9 (1.7 to 14.4)	0.8 (0.2 to 1.6)	8.7 (1.9 to 15.9)
Slovenia	Metabolic risks	2.6 (1 to 5.1)	49.1 (11.2 to 101.5)	2.9 (0.6 to 6.1)	52 (11.9 to 108.7)	2.4 (0.9 to 4.5)	41.5 (11.9 to 81.7)	4 (1 to 8.1)	45.5 (12.9 to 89.5)
Slovenia	High body-mass index	1.3 (0.3 to 2.8)	20.9 (-2.9 to 51.1)	1.2 (-0.3 to 3)	22.1 (-3.2 to 53.9)	1.2 (0.3 to 2.5)	19 (0 to 43.1)	1.7 (-0.2 to 4)	20.7 (-0.3 to 47.3)
Slovenia	High fasting plasma glucose	1.4 (0.3 to 3.4)	30.2 (5.4 to 73.3)	1.9 (0.3 to 4.7)	32.1 (5.8 to 78.5)	1.3 (0.2 to 2.9)	24.5 (4.5 to 58.2)	2.5 (0.5 to 6)	27 (5 to 64.6)
Solomon Islands	All risk factors	4.2 (2 to 6.9)	123.3 (60.2 to 206.3)	3.4 (1.6 to 6.1)	126.8 (61.8 to 212.2)	22.3 (12.7 to 34.2)	702.6 (401.5 to 1073)	20.4 (11.1 to 33.7)	723.1 (415 to 1110.6)
Solomon Islands	Behavioral risks	1.7 (0.9 to 2.7)	53.5 (28.5 to 84.3)	1.5 (0.7 to 2.5)	55 (29.5 to 86.5)	7.6 (4.7 to 10.9)	251.4 (152.9 to 369)	7.3 (4 to 11.7)	258.7 (157.2 to 379.4)
Solomon Islands	Alcohol use	0.1 (0 to 0.1)	2.7 (0.9 to 5.4)	0.1 (0 to 0.1)	2.8 (0.9 to 5.5)	0.6 (0.2 to 1.1)	23.8 (8.2 to 43.8)	0.7 (0.2 to 1.4)	24.5 (8.4 to 45.3)
Solomon Islands	Diet high in red meat	0.2 (0 to 0.4)	8 (1.5 to 13.7)	0.2 (0 to 0.4)	8.2 (1.6 to 14.1)	1 (0.2 to 1.6)	36 (6.9 to 57.1)	1 (0.2 to 1.8)	37 (7.1 to 58.6)
Solomon Islands	Low physical activity	0.2 (0.1 to 0.4)	5.3 (1.9 to 11.1)	0.2 (0.1 to 0.3)	5.4 (2 to 11.4)	0.9 (0.4 to 1.8)	26.2 (11 to 55)	0.8 (0.3 to 1.8)	27 (11.2 to 56.6)
Solomon Islands	Smoking	0.6 (0.3 to 1.1)	18.3 (8.1 to 31.4)	0.5 (0.2 to 0.9)	18.8 (8.4 to 32.3)	2.4 (1.3 to 3.6)	70.1 (36 to 109.2)	2.1 (1 to 3.5)	72.2 (37.1 to 112.6)
Solomon Islands	Secondhand smoke	0.7 (0.1 to 1.3)	21.4 (4.5 to 41)	0.6 (0.1 to 1.1)	21.9 (4.6 to 41.9)	3 (0.7 to 5.4)	104.8 (24.3 to 191.8)	3 (0.7 to 5.9)	107.8 (25 to 196.8)
Solomon Islands	Metabolic risks	2.7 (0.8 to 5.3)	78.4 (25.5 to 155.8)	2.2 (0.7 to 4.5)	80.6 (26.1 to 160.1)	16.5 (6.9 to 28.6)	502.9 (211.1 to 879)	14.6 (5.8 to 26.6)	517.6 (217.8 to 907.1)
Solomon Islands	High body-mass index	1.9 (0.5 to 4.3)	56.1 (14.3 to 123.3)	1.6 (0.4 to 3.5)	57.7 (14.7 to 126.6)	10.7 (3.4 to 20.6)	317.9 (102.1 to 600.9)	9.2 (2.8 to 17.9)	327.1 (105.4 to 620.3)
Solomon Islands	High fasting plasma glucose	0.9 (0.1 to 2.3)	25.6 (4 to 66.5)	0.7 (0.1 to 1.9)	26.3 (4.2 to 68.5)	7 (1.3 to 15.9)	218.1 (39.9 to 502.1)	6.4 (1.2 to 15.7)	224.4 (41.2 to 518.1)
Somalia	All risk factors	1.2 (0.6 to 2)	30.8 (14.1 to 53.9)	0.7 (0.3 to 1.3)	31.5 (14.5 to 55.6)	1.3 (0.6 to 2.7)	32.9 (13.9 to 66.3)	0.8 (0.3 to 1.5)	33.7 (14.2 to 68)
Somalia	Behavioral risks	0.7 (0.4 to 1.1)	21.5 (10.2 to 35)	0.4 (0.2 to 0.8)	21.9 (10.4 to 35.7)	0.7 (0.3 to 1.2)	19.2 (8.9 to 33.6)	0.4 (0.2 to 0.8)	19.7 (9.2 to 34.3)
Somalia	Alcohol use								
Somalia	Diet high in red meat	0.4 (0.1 to 0.6)	11.1 (3 to 18.9)	0.2 (0.1 to 0.4)	11.3 (3.1 to 19.1)	0.3 (0.1 to 0.6)	9.1 (2 to 17.3)	0.2 (0 to 0.4)	9.3 (2.1 to 17.7)
Somalia	Low physical activity	0.1 (0 to 0.1)	1.4 (0.8 to 3.4)	0 (0 to 0.1)	1.4 (0.8 to 3.5)	0.1 (0 to 0.1)	1.5 (0.8 to 3.5)	0 (0 to 0.1)	1.5 (0.8 to 3.6)
Somalia	Smoking	0.1 (0 to 0.2)	1.8 (0.7 to 3.5)	0 (0 to 0.1)	1.8 (0.7 to 3.6)	0.1 (0 to 0.2)	1.8 (0.7 to 3.7)	0 (0 to 0.1)	1.9 (0.7 to 3.8)
Somalia	Secondhand smoke	0.2 (0 to 0.5)	7.7 (1.5 to 14.9)	0.2 (0 to 0.3)	7.8 (1.5 to 15.2)	0.2 (0.1 to 0.5)	7.2 (1.6 to 14.8)	0.2 (0 to 0.3)	7.4 (1.6 to 15.1)
Somalia	Metabolic risks	0.5 (0.1 to 1.1)	9.9 (0.7 to 25.6)	0.3 (0 to 0.7)	10.2 (0.8 to 26.2)	0.7 (0.1 to 1.8)	14.4 (1.5 to 39.9)	0.4 (0.1 to 1)	14.8 (1.5 to 40.9)
Somalia	High body-mass index	0.1 (0 to 0.2)	0 (-3.3 to 3.1)	0 (0 to 0.1)	0 (-3.4 to 3.2)	0.1 (0 to 0.3)	0.2 (-3.3 to 4.3)	0 (0 to 0.2)	0.2 (-3.4 to 4.4)

Location	Risk factor	1990				2019			
		Deaths	YLLs (Years of Life Lost)	YLDs (Years Lived with Disability)	DALYs (Disability-Adjusted Life Years)	Deaths	YLLs (Years of Life Lost)	YLDs (Years Lived with Disability)	DALYs (Disability-Adjusted Life Years)
Somalia	High fasting plasma glucose	0.4 (0.1 to 1.1)	10 (1.7 to 25.2)	0.2 (0 to 0.6)	10.2 (1.7 to 25.8)	0.6 (0.1 to 1.7)	14.3 (2.3 to 39.8)	0.3 (0.1 to 1)	14.6 (2.4 to 40.8)
South Africa	All risk factors	5 (3.4 to 6.9)	119.4 (81.5 to 161.2)	3.7 (2.2 to 5.4)	123.1 (84.4 to 165.5)	5.9 (3.8 to 8.3)	125.8 (80.8 to 179.2)	4.8 (2.8 to 7.1)	130.6 (83.3 to 185.6)
South Africa	Behavioral risks	3.4 (2.6 to 4.3)	99.5 (77 to 123.6)	2.9 (1.9 to 4.1)	102.4 (79.3 to 127.3)	3.1 (2.4 to 3.8)	78.4 (60.3 to 97.3)	2.9 (1.9 to 4.1)	81.3 (62.3 to 100.7)
South Africa	Alcohol use	1.2 (0.9 to 1.6)	38 (28.3 to 49.2)	1.1 (0.7 to 1.6)	39.1 (29.2 to 50.6)	1.2 (0.8 to 1.5)	31.1 (22.3 to 41.2)	1.1 (0.7 to 1.7)	32.3 (23.1 to 42.8)
South Africa	Diet high in red meat	0.6 (0.2 to 0.9)	18.3 (7.1 to 25)	0.5 (0.2 to 0.8)	18.8 (7.2 to 25.7)	0.7 (0.3 to 1)	18.7 (7.7 to 26.1)	0.7 (0.3 to 1.1)	19.4 (8 to 27)
South Africa	Low physical activity	0.3 (0.1 to 0.5)	6.9 (2.8 to 13)	0.2 (0.1 to 0.4)	7.1 (2.8 to 13.4)	0.3 (0.1 to 0.6)	6.5 (2.8 to 12)	0.3 (0.1 to 0.5)	6.8 (2.9 to 12.4)
South Africa	Smoking	0.9 (0.6 to 1.3)	24.1 (15.3 to 35.2)	0.7 (0.4 to 1.1)	24.8 (15.8 to 36.2)	0.5 (0.4 to 0.8)	13.1 (8.4 to 18.1)	0.5 (0.3 to 0.8)	13.6 (8.8 to 18.8)
South Africa	Secondhand smoke	0.7 (0.2 to 1.2)	20.5 (4.8 to 35.5)	0.6 (0.1 to 1.1)	21.1 (5 to 36.4)	0.5 (0.1 to 0.9)	13.9 (3.4 to 24.6)	0.5 (0.1 to 0.9)	14.4 (3.5 to 25.4)
South Africa	Metabolic risks	1.9 (0.5 to 3.8)	25.4 (-8.2 to 66.6)	1 (0 to 2.3)	26.4 (-8.3 to 69.2)	3.3 (1.3 to 5.9)	56.3 (12.8 to 111.9)	2.2 (0.6 to 4.4)	58.5 (13.6 to 115.9)
South Africa	High body-mass index	0.8 (-0.1 to 1.9)	2.7 (-21.5 to 26.3)	0.3 (-0.4 to 1)	3 (-21.9 to 27.2)	1.6 (0.4 to 3.1)	20.3 (-7.4 to 51.2)	0.9 (-0.2 to 2)	21.2 (-7.5 to 53.3)
South Africa	High fasting plasma glucose	1.2 (0.2 to 2.7)	24.3 (4.6 to 56.7)	0.8 (0.1 to 1.9)	25.1 (4.7 to 58.7)	2 (0.4 to 4.3)	40 (7.8 to 90.8)	1.5 (0.3 to 3.6)	41.6 (8.2 to 93.3)
South Sudan	All risk factors	1.3 (0.7 to 2.1)	30.9 (16.3 to 50.3)	0.8 (0.4 to 1.3)	31.7 (16.8 to 51.5)	1.6 (0.8 to 2.8)	35.4 (16.7 to 61.9)	0.9 (0.4 to 1.7)	36.3 (17.1 to 63.5)
South Sudan	Behavioral risks	0.7 (0.4 to 1)	19.2 (11 to 28.3)	0.4 (0.2 to 0.7)	19.7 (11.3 to 28.9)	0.7 (0.4 to 1.1)	18.2 (9.7 to 29)	0.4 (0.2 to 0.7)	18.6 (10 to 29.8)
South Sudan	Alcohol use	0.1 (0 to 0.2)	3.2 (1.4 to 6)	0.1 (0 to 0.1)	3.3 (1.4 to 6.1)	0.1 (0 to 0.2)	2.6 (1 to 5)	0.1 (0 to 0.1)	2.7 (1.1 to 5.2)
South Sudan	Diet high in red meat	0.2 (0 to 0.3)	6 (1.2 to 10)	0.1 (0 to 0.2)	6.1 (1.2 to 10.2)	0.2 (0 to 0.4)	6 (1.1 to 10.3)	0.1 (0 to 0.3)	6.1 (1.1 to 10.5)
South Sudan	Low physical activity	0.1 (0 to 0.1)	1.5 (0.8 to 3.5)	0 (0 to 0.1)	1.5 (0.9 to 3.5)	0.1 (0 to 0.2)	1.5 (0.8 to 3.6)	0 (0 to 0.1)	1.6 (0.9 to 3.6)
South Sudan	Smoking	0.1 (0 to 0.2)	1.9 (0.8 to 3.5)	0 (0 to 0.1)	2 (0.8 to 3.6)	0.1 (0 to 0.2)	1.8 (0.7 to 3.2)	0 (0 to 0.1)	1.9 (0.7 to 3.3)
South Sudan	Secondhand smoke	0.2 (0.1 to 0.4)	7 (1.6 to 13)	0.1 (0 to 0.3)	7.1 (1.6 to 13.3)	0.2 (0.1 to 0.5)	6.6 (1.5 to 13.2)	0.2 (0 to 0.3)	6.8 (1.5 to 13.5)
South Sudan	Metabolic risks	0.7 (0.2 to 1.4)	12.4 (0.9 to 29.2)	0.3 (0.1 to 0.8)	12.7 (1 to 30.1)	1 (0.3 to 2.1)	18.1 (1.6 to 40.8)	0.5 (0.1 to 1.2)	18.7 (1.8 to 41.8)
South Sudan	High body-mass index	0.2 (0 to 0.6)	2.7 (-4 to 10)	0.1 (0 to 0.3)	2.8 (-4 to 10.4)	0.4 (0 to 1)	5.4 (-3.8 to 17.2)	0.2 (0 to 0.5)	5.6 (-3.8 to 17.7)
South Sudan	High fasting plasma glucose	0.5 (0.1 to 1.1)	10 (1.8 to 24.2)	0.3 (0 to 0.6)	10.2 (1.8 to 24.9)	0.6 (0.1 to 1.5)	13.3 (2.3 to 33.1)	0.4 (0.1 to 0.9)	13.7 (2.4 to 33.9)
Spain	All risk factors	7.2 (5.6 to 9)	200.4 (160.2 to 242.6)	15.1 (9.8 to 21.7)	215.6 (171.9 to 261.1)	4.9 (3.6 to 6.4)	123.1 (94.7 to 154.9)	16.9 (10.5 to 25.6)	140.1 (107 to 177.3)
Spain	Behavioral risks	5.3 (4.5 to 6.2)	167 (141.6 to 192.6)	12.1 (8 to 17.1)	179.1 (151.6 to 207.3)	3.3 (2.8 to 3.9)	96 (80.8 to 112.7)	13 (8.1 to 19.9)	109 (90.6 to 129)
Spain	Alcohol use	2.5 (2 to 3.1)	80.6 (64.6 to 97.6)	5.8 (3.8 to 8.6)	86.4 (69 to 104.9)	1.4 (1 to 1.7)	40.3 (31.3 to 50.7)	5.4 (3.2 to 8.6)	45.7 (35 to 57.6)
Spain	Diet high in red meat	1.2 (0.6 to 1.6)	36.4 (18.4 to 48.8)	2.7 (1.2 to 4.3)	39.1 (19.8 to 52.8)	0.8 (0.4 to 1)	20.5 (10.4 to 28.2)	2.8 (1.2 to 4.7)	23.3 (11.8 to 32.3)
Spain	Low physical activity	0.3 (0.1 to 0.6)	6.8 (2.6 to 14.2)	0.6 (0.2 to 1.2)	7.4 (2.9 to 15.4)	0.2 (0.1 to 0.4)	4.8 (1.7 to 9.6)	0.7 (0.2 to 1.5)	5.4 (1.9 to 11.1)
Spain	Smoking	1.3 (0.9 to 1.7)	44 (30.5 to 58.9)	3.1 (1.8 to 4.6)	47.1 (32.5 to 62.8)	1 (0.8 to 1.3)	32.8 (23.5 to 43.1)	4.4 (2.5 to 7.1)	37.2 (26.6 to 48.8)
Spain	Secondhand smoke	0.5 (0.1 to 0.9)	17 (4.3 to 29)	1.2 (0.3 to 2.2)	18.2 (4.6 to 31.4)	0.3 (0.1 to 0.5)	7.9 (1.8 to 14)	1.1 (0.2 to 2.1)	9 (2.1 to 15.9)
Spain	Metabolic risks	2.3 (0.8 to 4.3)	41.8 (8.1 to 85.3)	3.7 (0.9 to 7.7)	45.5 (9.3 to 92.7)	2 (0.7 to 3.7)	34.9 (8.4 to 70.9)	5 (1.3 to 10.6)	40 (9.4 to 80.5)
Spain	High body-mass index	0.9 (0.1 to 1.9)	12 (-8.2 to 34.2)	1.2 (-0.4 to 3)	13.2 (-8.3 to 37.5)	0.8 (0.2 to 1.7)	11.2 (-3.7 to 28.6)	1.6 (-0.4 to 4.2)	12.8 (-3.8 to 32.5)
Spain	High fasting plasma glucose	1.5 (0.3 to 3.4)	31.8 (6.2 to 71.6)	2.7 (0.5 to 6.5)	34.5 (6.7 to 77.8)	1.3 (0.3 to 2.9)	25.5 (4.8 to 57.5)	3.7 (0.7 to 9.3)	29.2 (5.5 to 66.2)
Sri Lanka	All risk factors	1.9 (1 to 2.9)	47.2 (26.1 to 72.8)	1.7 (0.8 to 2.9)	48.9 (27.1 to 75.5)	3.9 (2 to 6.4)	90 (45.8 to 148.8)	5.2 (2.5 to 8.9)	95.2 (49 to 156.9)
Sri Lanka	Behavioral risks	0.6 (0.3 to 0.8)	16.2 (8.7 to 23.8)	0.6 (0.3 to 0.9)	16.8 (9 to 24.8)	0.8 (0.5 to 1.2)	21.4 (11.9 to 32.9)	1.3 (0.6 to 2.1)	22.7 (12.7 to 34.9)
Sri Lanka	Alcohol use	0.1 (0 to 0.1)	2.3 (1.4 to 3.3)	0.1 (0 to 0.1)	2.4 (1.5 to 3.4)	0.2 (0.1 to 0.3)	6.4 (4 to 9.6)	0.4 (0.2 to 0.6)	6.7 (4.3 to 10.1)
Sri Lanka	Diet high in red meat	0 (0 to 0.1)	1.4 (0.6 to 1.9)	0 (0 to 0.1)	1.4 (0.6 to 1.9)	0.1 (0 to 0.1)	1.7 (0.7 to 2.7)	0.1 (0 to 0.2)	1.8 (0.7 to 2.8)
Sri Lanka	Low physical activity	0.1 (0 to 0.1)	1.3 (0.8 to 2.7)	0 (0 to 0.1)	1.3 (0.8 to 2.8)	0.1 (0 to 0.1)	1.6 (0.9 to 3.3)	0.1 (0 to 0.2)	1.7 (1 to 3.6)
Sri Lanka	Smoking	0.1 (0.1 to 0.1)	2 (1.1 to 3.1)	0.1 (0 to 0.1)	2 (1.2 to 3.2)	0.1 (0 to 0.1)	1.6 (0.9 to 2.7)	0.1 (0 to 0.2)	1.7 (1 to 2.8)
Sri Lanka	Secondhand smoke	0.3 (0.1 to 0.5)	9.6 (2.3 to 17.1)	0.3 (0.1 to 0.6)	10 (2.4 to 17.7)	0.4 (0.1 to 0.7)	10.6 (2.6 to 20.6)	0.6 (0.1 to 1.2)	11.2 (2.8 to 21.8)
Sri Lanka	Metabolic risks	1.4 (0.5 to 2.5)	33 (12.8 to 58.6)	1.2 (0.5 to 2.3)	34.2 (13.4 to 60.6)	3.3 (1.4 to 5.8)	73.4 (30.5 to 131.7)	4.3 (1.7 to 7.7)	77.7 (32.8 to 138.1)
Sri Lanka	High body-mass index	0.8 (0.2 to 1.7)	21.2 (6 to 43.4)	0.8 (0.2 to 1.6)	22 (6.2 to 45)	2 (0.6 to 3.9)	45.6 (13.6 to 90.5)	2.6 (0.7 to 5.4)	48.2 (14.5 to 95.4)

Location	Risk factor	1990				2019			
		Deaths	YLLs (Years of Life Lost)	YLDs (Years Lived with Disability)	DALYs (Disability-Adjusted Life Years)	Deaths	YLLs (Years of Life Lost)	YLDs (Years Lived with Disability)	DALYs (Disability-Adjusted Life Years)
Sri Lanka	High fasting plasma glucose	0.6 (0.1 to 1.4)	13.1 (2.5 to 30)	0.5 (0.1 to 1.3)	13.6 (2.6 to 31.3)	1.6 (0.3 to 3.7)	34 (6.8 to 80.2)	2 (0.4 to 4.8)	36 (7.2 to 85)
Sudan	All risk factors	1.3 (0.7 to 2.5)	36.8 (18 to 67)	1.1 (0.5 to 2)	37.9 (18.7 to 69)	2.5 (1.2 to 4.4)	62.2 (28.4 to 111)	2.4 (1 to 4.4)	64.6 (29.7 to 115.3)
Sudan	Behavioral risks	0.8 (0.5 to 1.3)	25.2 (14.7 to 40.5)	0.7 (0.4 to 1.2)	25.9 (15 to 41.6)	1 (0.6 to 1.6)	31.5 (17.3 to 49.2)	1.2 (0.6 to 1.9)	32.7 (18.1 to 51.3)
Sudan	Alcohol use	0 (0 to 0.1)	1.6 (0.8 to 2.9)	0 (0 to 0.1)	1.7 (0.8 to 2.9)	0 (0 to 0)	0.1 (0 to 0.3)	0 (0 to 0)	0.1 (0 to 0.3)
Sudan	Diet high in red meat	0.2 (0 to 0.3)	5.4 (1.1 to 9.3)	0.1 (0 to 0.3)	5.5 (1.1 to 9.6)	0.2 (0 to 0.4)	7 (1.4 to 12.1)	0.3 (0.1 to 0.5)	7.3 (1.4 to 12.6)
Sudan	Low physical activity	0.3 (0.1 to 0.6)	10.4 (3.3 to 19.3)	0.3 (0.1 to 0.6)	10.7 (3.4 to 19.8)	0.5 (0.2 to 0.9)	14.3 (4.9 to 26.2)	0.5 (0.2 to 1)	14.9 (5.1 to 27.1)
Sudan	Smoking	0.1 (0 to 0.1)	2 (1.1 to 3.5)	0.1 (0 to 0.1)	2.1 (1.1 to 3.6)	0.1 (0.1 to 0.1)	2.5 (1.4 to 4)	0.1 (0 to 0.2)	2.6 (1.5 to 4.2)
Sudan	Secondhand smoke	0.2 (0 to 0.4)	6.4 (1.5 to 12.6)	0.2 (0 to 0.3)	6.6 (1.5 to 13)	0.3 (0.1 to 0.5)	8.4 (2.1 to 16.7)	0.3 (0.1 to 0.6)	8.7 (2.2 to 17.4)
Sudan	Metabolic risks	0.6 (0.1 to 1.5)	12.7 (-1.3 to 35.1)	0.4 (0 to 1.1)	13.2 (-1.2 to 36.2)	1.6 (0.5 to 3.4)	33.5 (1.7 to 78.9)	1.3 (0.1 to 3.1)	34.8 (1.9 to 82.2)
Sudan	High body-mass index	0.1 (-0.1 to 0.5)	0.4 (-7.8 to 8.1)	0.1 (-0.1 to 0.3)	0.5 (-7.9 to 8.3)	0.5 (-0.1 to 1.1)	3.8 (-13.6 to 19.1)	0.1 (-0.5 to 0.8)	3.9 (-14.4 to 19.8)
Sudan	High fasting plasma glucose	0.5 (0.1 to 1.3)	12.6 (2.1 to 34.2)	0.4 (0.1 to 1)	13 (2.2 to 35.2)	1.2 (0.2 to 2.9)	31.4 (6 to 77)	1.2 (0.2 to 3)	32.6 (6.2 to 79.8)
Suriname	All risk factors	3.6 (2.4 to 5.2)	92 (61 to 131.7)	3.3 (2 to 5.3)	95.4 (63.2 to 136.2)	5.1 (3 to 8.1)	124.3 (73.1 to 198.3)	5.5 (3 to 9.4)	129.8 (76.6 to 206.5)
Suriname	Behavioral risks	2.1 (1.5 to 2.6)	61.4 (45.4 to 78.1)	2.1 (1.3 to 3)	63.5 (47.2 to 80.8)	2.4 (1.7 to 3.2)	70.8 (50.6 to 93.5)	3 (1.8 to 4.3)	73.8 (52.6 to 97.2)
Suriname	Alcohol use	0.8 (0.6 to 1.1)	27.7 (20.4 to 36.6)	0.9 (0.6 to 1.3)	28.6 (21 to 37.8)	0.9 (0.6 to 1.2)	30.9 (21.1 to 42)	1.2 (0.8 to 1.9)	32.2 (21.9 to 43.8)
Suriname	Diet high in red meat	0.2 (0 to 0.3)	6 (1.2 to 8.6)	0.2 (0 to 0.3)	6.2 (1.2 to 8.9)	0.2 (0 to 0.4)	7.1 (1.4 to 11)	0.3 (0.1 to 0.5)	7.4 (1.5 to 11.4)
Suriname	Low physical activity	0.3 (0.1 to 0.5)	6 (2.2 to 12)	0.2 (0.1 to 0.5)	6.2 (2.2 to 12.5)	0.4 (0.1 to 0.7)	8.2 (2.6 to 15.9)	0.4 (0.1 to 0.8)	8.6 (2.7 to 16.6)
Suriname	Smoking	0.5 (0.3 to 0.7)	12.2 (7.5 to 18.2)	0.4 (0.2 to 0.7)	12.7 (7.8 to 18.8)	0.5 (0.3 to 0.8)	13.4 (8.4 to 20.5)	0.6 (0.3 to 1)	14 (8.7 to 21.4)
Suriname	Secondhand smoke	0.4 (0.1 to 0.7)	12.6 (3 to 22.6)	0.4 (0.1 to 0.8)	13 (3.1 to 23.4)	0.5 (0.1 to 0.9)	14.8 (3.6 to 27.1)	0.6 (0.1 to 1.1)	15.4 (3.7 to 28.3)
Suriname	Metabolic risks	1.8 (0.6 to 3.4)	35.1 (6.3 to 75.1)	1.4 (0.4 to 3.1)	36.5 (6.8 to 77.9)	3.1 (1 to 6.1)	61.5 (12.5 to 135)	2.9 (0.7 to 6.4)	64.4 (13.4 to 140.9)
Suriname	High body-mass index	0.5 (0 to 1.2)	5.8 (-6.4 to 20.3)	0.3 (-0.1 to 0.9)	6.1 (-6.6 to 21.2)	0.8 (0 to 1.8)	8 (-13.7 to 30.1)	0.5 (-0.4 to 1.6)	8.6 (-14 to 31.8)
Suriname	High fasting plasma glucose	1.3 (0.3 to 2.9)	30.6 (5.8 to 67.9)	1.2 (0.2 to 2.8)	31.7 (6 to 70.6)	2.4 (0.5 to 5.3)	56.6 (11.5 to 127.4)	2.5 (0.5 to 6)	59.2 (12 to 132.2)
Sweden	All risk factors	7.3 (6 to 8.6)	193.5 (163 to 225.4)	19.3 (12.8 to 27.1)	212.8 (178.6 to 248.3)	6.3 (5 to 7.8)	143.5 (115.4 to 174.6)	21.7 (13.7 to 32)	165.3 (132.6 to 201.5)
Sweden	Behavioral risks	6.1 (5.2 to 6.9)	171.1 (148.3 to 194.4)	16.7 (11.3 to 23.5)	187.8 (161.9 to 213.8)	4.8 (4 to 5.5)	117.3 (99.4 to 135)	17.5 (11.1 to 25.4)	134.8 (112.4 to 155.6)
Sweden	Alcohol use	3.4 (2.8 to 4.1)	95.5 (78.6 to 113.3)	9.4 (6.1 to 13.5)	104.9 (86.2 to 124.2)	2.8 (2.2 to 3.4)	69.7 (56.3 to 83.7)	10.4 (6.4 to 15.4)	80.1 (64 to 96.5)
Sweden	Diet high in red meat	1 (0.5 to 1.3)	28.7 (14 to 38)	2.8 (1.2 to 4.4)	31.4 (15.2 to 42)	0.9 (0.4 to 1.2)	21.5 (10.8 to 29.4)	3.2 (1.4 to 5.3)	24.7 (12.3 to 34.2)
Sweden	Low physical activity	0.3 (0.1 to 0.5)	6 (2.3 to 12.4)	0.6 (0.2 to 1.4)	6.6 (2.6 to 13.8)	0.2 (0.1 to 0.5)	4.6 (1.7 to 9.6)	0.7 (0.2 to 1.6)	5.4 (2 to 11.1)
Sweden	Smoking	1.8 (1.3 to 2.3)	51.7 (37.3 to 67)	5.1 (3 to 7.7)	56.8 (40.8 to 74)	1.2 (0.9 to 1.6)	29.2 (21.4 to 37.7)	4.5 (2.6 to 7.1)	33.7 (24.5 to 44)
Sweden	Secondhand smoke	0.2 (0.1 to 0.4)	8.3 (1.9 to 14.6)	0.8 (0.2 to 1.4)	9 (2.1 to 16)	0.1 (0 to 0.2)	4.2 (1 to 7.3)	0.6 (0.1 to 1.2)	4.8 (1.1 to 8.5)
Sweden	Metabolic risks	1.7 (0.6 to 3.3)	31.9 (6.8 to 64.7)	3.6 (1 to 7.3)	35.5 (7.8 to 71.5)	2.1 (0.8 to 3.9)	37.3 (11 to 73.9)	5.9 (1.7 to 12)	43.3 (13 to 84.8)
Sweden	High body-mass index	0.8 (0.2 to 1.7)	12 (-3.1 to 30.7)	1.5 (-0.1 to 3.5)	13.4 (-3.1 to 34)	1 (0.3 to 2)	14.5 (-0.5 to 33.3)	2.4 (0.1 to 5.4)	16.9 (-0.4 to 38.1)
Sweden	High fasting plasma glucose	1 (0.2 to 2.2)	20.9 (3.9 to 48.2)	2.2 (0.4 to 5.4)	23.1 (4.3 to 53)	1.3 (0.2 to 2.9)	24.4 (4.6 to 56.4)	3.8 (0.6 to 9.1)	28.2 (5.4 to 64.3)
Switzerland	All risk factors	12.3 (10.2 to 14.7)	316 (264.8 to 369.8)	25.7 (17.1 to 35.5)	341.7 (286.2 to 400.1)	6.6 (5.3 to 8.3)	150.8 (122.8 to 184.6)	21.3 (13.5 to 31.7)	172.1 (139 to 210.7)
Switzerland	Behavioral risks	10.1 (8.7 to 11.6)	275.8 (237.7 to 315.6)	22.3 (15 to 30.8)	298.1 (256.3 to 341)	5.1 (4.2 to 6)	123.6 (104.5 to 143.9)	17.3 (11 to 25.8)	140.9 (117.8 to 165)
Switzerland	Alcohol use	6.1 (5 to 7.4)	166 (136.7 to 199.2)	13.5 (8.7 to 19)	179.4 (147.1 to 214.7)	3 (2.3 to 3.6)	72.7 (58.2 to 88.9)	10.2 (6.2 to 15.4)	82.9 (66.1 to 102.4)
Switzerland	Diet high in red meat	2 (1 to 2.7)	53 (26.1 to 72.7)	4.3 (2 to 7)	57.3 (28.2 to 78.5)	1 (0.5 to 1.3)	24 (11.9 to 32.9)	3.4 (1.4 to 5.6)	27.4 (13.8 to 37.8)
Switzerland	Low physical activity	0.6 (0.2 to 1)	12.3 (4.1 to 24.2)	1 (0.4 to 2.3)	13.3 (4.5 to 26.2)	0.3 (0.1 to 0.6)	6.6 (2.2 to 12.5)	1 (0.3 to 2)	7.6 (2.5 to 14.4)
Switzerland	Smoking	2 (1.4 to 2.6)	58.3 (41 to 77)	4.6 (2.8 to 6.9)	62.9 (44.2 to 82.9)	1.1 (0.8 to 1.4)	27.4 (19.8 to 35.1)	3.8 (2.2 to 6.1)	31.2 (22.5 to 40.1)

Location	Risk factor	1990				2019			
		Deaths	YLLs (Years of Life Lost)	YLDs (Years Lived with Disability)	DALYs (Disability-Adjusted Life Years)	Deaths	YLLs (Years of Life Lost)	YLDs (Years Lived with Disability)	DALYs (Disability-Adjusted Life Years)
Switzerland	Secondhand smoke	0.6 (0.2 to 1.1)	19.1 (4.7 to 32.7)	1.5 (0.3 to 2.8)	20.6 (5 to 35)	0.2 (0.1 to 0.4)	6.9 (1.7 to 12)	0.9 (0.2 to 1.8)	7.8 (1.9 to 13.8)
Switzerland	Metabolic risks	3.1 (1.2 to 5.8)	57.6 (16.2 to 114.8)	4.8 (1.3 to 9.9)	62.4 (18 to 123.6)	2.2 (0.8 to 4)	38.6 (12.8 to 74.4)	5.6 (1.7 to 11.7)	44.3 (14.3 to 85.2)
Switzerland	High body-mass index	1.4 (0.3 to 2.8)	22.1 (-0.3 to 50.6)	1.7 (-0.1 to 4.2)	23.8 (-0.4 to 55)	0.9 (0.3 to 1.8)	14.5 (1.6 to 31.9)	2.1 (0.3 to 4.7)	16.6 (1.9 to 36.8)
Switzerland	High fasting plasma glucose	1.9 (0.4 to 4.2)	37.5 (7.1 to 86.9)	3.2 (0.6 to 7.9)	40.7 (7.7 to 94)	1.3 (0.3 to 3)	25.7 (4.9 to 57.9)	3.8 (0.7 to 9.6)	29.4 (5.6 to 67.7)
Syrian Arab Republic	All risk factors	1.4 (0.7 to 2.3)	34.3 (16.7 to 57.4)	1.4 (0.7 to 2.5)	35.8 (17.7 to 59.4)	2.4 (1.3 to 4.1)	52.8 (26.3 to 92.9)	3 (1.4 to 5.4)	55.8 (27.7 to 97.7)
Syrian Arab Republic	Behavioral risks	0.8 (0.5 to 1.1)	23.5 (13.7 to 34.7)	0.9 (0.5 to 1.4)	24.4 (14.2 to 36.1)	1 (0.6 to 1.4)	26.4 (14.7 to 40.7)	1.5 (0.8 to 2.5)	27.9 (15.5 to 43.1)
Syrian Arab Republic	Alcohol use	0 (0 to 0)	0.9 (0.6 to 1.4)	0 (0 to 0.1)	1 (0.6 to 1.5)	0 (0 to 0)	0.7 (0.4 to 1.1)	0 (0 to 0.1)	0.7 (0.4 to 1.2)
Syrian Arab Republic	Diet high in red meat	0.2 (0 to 0.3)	5.9 (1.3 to 9.3)	0.2 (0 to 0.4)	6.1 (1.3 to 9.7)	0.2 (0 to 0.4)	6.3 (1.3 to 10.8)	0.4 (0.1 to 0.6)	6.7 (1.3 to 11.3)
Syrian Arab Republic	Low physical activity	0.2 (0.1 to 0.3)	4 (1.5 to 7.6)	0.2 (0.1 to 0.3)	4.2 (1.6 to 8)	0.3 (0.1 to 0.5)	5.7 (2.1 to 11.1)	0.3 (0.1 to 0.7)	6.1 (2.2 to 11.8)
Syrian Arab Republic	Smoking	0.2 (0.1 to 0.2)	4.4 (2.1 to 7.2)	0.2 (0.1 to 0.3)	4.5 (2.2 to 7.5)	0.2 (0.1 to 0.2)	4.1 (2.2 to 6.7)	0.2 (0.1 to 0.4)	4.4 (2.4 to 7.1)
Syrian Arab Republic	Secondhand smoke	0.3 (0.1 to 0.5)	9.2 (2.2 to 17.4)	0.3 (0.1 to 0.6)	9.5 (2.3 to 18.1)	0.3 (0.1 to 0.6)	10.4 (2 to 19.8)	0.6 (0.1 to 1.1)	11 (2.1 to 20.9)
Syrian Arab Republic	Metabolic risks	0.7 (0.1 to 1.6)	12.1 (-3.2 to 32.8)	0.6 (0 to 1.4)	12.8 (-3.1 to 34.1)	1.6 (0.5 to 3.2)	29.1 (3.1 to 67.4)	1.7 (0.2 to 3.9)	30.7 (3.3 to 71.3)
Syrian Arab Republic	High body-mass index	0.2 (-0.1 to 0.6)	0.4 (-9.6 to 10.4)	0.1 (-0.2 to 0.5)	0.5 (-9.8 to 10.8)	0.6 (0 to 1.2)	5.8 (-8 to 20.3)	0.3 (-0.5 to 1.2)	6.1 (-8.5 to 21.4)
Syrian Arab Republic	High fasting plasma glucose	0.5 (0.1 to 1.2)	12.3 (2.1 to 29.5)	0.5 (0.1 to 1.3)	12.8 (2.2 to 30.7)	1.1 (0.2 to 2.7)	25 (5 to 61.6)	1.5 (0.3 to 3.6)	26.5 (5.3 to 64.4)
Taiwan (Province of China)	All risk factors	2.3 (1.5 to 3.2)	66.5 (44.1 to 90.7)	3.7 (2.2 to 5.6)	70.1 (46.6 to 95.8)	3.4 (1.9 to 5.1)	93.5 (55.1 to 141)	9.2 (4.9 to 14.7)	102.8 (61.1 to 155.7)
Taiwan (Province of China)	Behavioral risks	1.1 (0.8 to 1.4)	36.3 (25.5 to 46.4)	2 (1.2 to 2.9)	38.3 (26.7 to 49)	1.3 (0.9 to 1.9)	41.9 (26.7 to 60.7)	4.2 (2.3 to 6.6)	46.1 (29.3 to 66.7)
Taiwan (Province of China)	Alcohol use	0.2 (0.2 to 0.3)	8.2 (6.2 to 10.6)	0.4 (0.3 to 0.7)	8.6 (6.5 to 11.2)	0.2 (0.1 to 0.3)	6.5 (4.1 to 9.5)	0.6 (0.4 to 1)	7.1 (4.5 to 10.5)
Taiwan (Province of China)	Diet high in red meat	0.4 (0.1 to 0.5)	11.9 (4.8 to 15.7)	0.7 (0.2 to 1)	12.6 (5 to 16.5)	0.6 (0.3 to 1)	19.4 (8.9 to 30)	2 (0.8 to 3.2)	21.4 (9.8 to 33.1)
Taiwan (Province of China)	Low physical activity	0.1 (0 to 0.1)	1.8 (1 to 3.4)	0.1 (0.1 to 0.2)	1.9 (1.1 to 3.6)	0.1 (0 to 0.2)	2.5 (1.2 to 5)	0.3 (0.1 to 0.5)	2.8 (1.4 to 5.5)
Taiwan (Province of China)	Smoking	0.1 (0.1 to 0.2)	3.9 (2.3 to 6)	0.2 (0.1 to 0.4)	4.1 (2.5 to 6.4)	0.1 (0.1 to 0.2)	3.8 (2 to 6.2)	0.4 (0.2 to 0.7)	4.2 (2.2 to 6.7)
Taiwan (Province of China)	Secondhand smoke	0.4 (0.1 to 0.6)	12.4 (3.1 to 21.3)	0.7 (0.1 to 1.2)	13.1 (3.3 to 22.4)	0.4 (0.1 to 0.6)	11.5 (2.5 to 21.1)	1.2 (0.2 to 2.2)	12.7 (2.8 to 23.5)
Taiwan (Province of China)	Metabolic risks	1.3 (0.5 to 2.3)	34.4 (13.8 to 60.8)	1.9 (0.7 to 3.5)	36.3 (14.7 to 64)	2.3 (0.9 to 4.1)	58.5 (23.3 to 104.7)	5.7 (2.1 to 10.4)	64.2 (25.7 to 114.9)
Taiwan (Province of China)	High body-mass index	0.9 (0.3 to 1.9)	23.9 (7.1 to 47.7)	1.3 (0.4 to 2.8)	25.3 (7.4 to 50.1)	1.6 (0.5 to 3.2)	43.2 (13.1 to 82.4)	4.1 (1.2 to 8.4)	47.3 (14.3 to 90.4)
Taiwan (Province of China)	High fasting plasma glucose	0.5 (0.1 to 1.1)	12 (2.3 to 27.3)	0.7 (0.1 to 1.7)	12.7 (2.4 to 28.8)	0.8 (0.1 to 1.8)	18.4 (3.4 to 44.1)	1.8 (0.3 to 4.7)	20.3 (3.8 to 48.6)
Tajikistan	All risk factors	1.9 (1.3 to 2.8)	58.7 (37.8 to 82.8)	2 (1.1 to 3)	60.7 (39.2 to 85.6)	2.4 (1.3 to 3.9)	60.8 (31.8 to 100.7)	2.4 (1.2 to 4.2)	63.2 (33.1 to 104.2)
Tajikistan	Behavioral risks	1.3 (0.9 to 1.7)	44.4 (30.7 to 58.1)	1.4 (0.8 to 2.1)	45.8 (31.6 to 60)	1 (0.6 to 1.3)	30 (19.1 to 41.4)	1.1 (0.6 to 1.7)	31.2 (19.7 to 43)
Tajikistan	Alcohol use	0.5 (0.3 to 0.6)	18 (12.3 to 24.5)	0.5 (0.3 to 0.9)	18.6 (12.6 to 25.3)	0.3 (0.2 to 0.4)	9.6 (5.9 to 14.4)	0.3 (0.2 to 0.6)	10 (6.1 to 14.9)
Tajikistan	Diet high in red meat	0.3 (0.1 to 0.4)	9.9 (2 to 13.9)	0.3 (0.1 to 0.5)	10.2 (2.1 to 14.4)	0.3 (0 to 0.4)	7.7 (1.5 to 12)	0.3 (0.1 to 0.5)	8 (1.5 to 12.4)
Tajikistan	Low physical activity	0.1 (0.1 to 0.2)	2.3 (1.5 to 4.8)	0.1 (0 to 0.2)	2.4 (1.6 to 4.9)	0.1 (0.1 to 0.2)	2.4 (1.4 to 4.6)	0.1 (0.1 to 0.2)	2.5 (1.5 to 4.8)
Tajikistan	Smoking	0.1 (0.1 to 0.2)	3.8 (2.2 to 5.8)	0.1 (0.1 to 0.2)	4 (2.3 to 6)	0.1 (0.1 to 0.1)	2.6 (1.6 to 4)	0.1 (0.1 to 0.2)	2.7 (1.6 to 4.2)
Tajikistan	Secondhand smoke	0.3 (0.1 to 0.6)	11.9 (2.8 to 21.3)	0.4 (0.1 to 0.7)	12.3 (2.9 to 22.1)	0.3 (0.1 to 0.5)	8.5 (1.9 to 15.5)	0.3 (0.1 to 0.6)	8.8 (2 to 16.2)
Tajikistan	Metabolic risks	0.7 (0.2 to 1.5)	15.5 (-0.2 to 37.4)	0.6 (0.1 to 1.4)	16.1 (-0.1 to 38.9)	1.5 (0.5 to 3)	32.7 (6.7 to 70.4)	1.3 (0.3 to 3)	34.1 (7 to 73.4)
Tajikistan	High body-mass index	0.2 (0 to 0.6)	1.8 (-7.2 to 10.5)	0.1 (-0.2 to 0.4)	1.9 (-7.4 to 10.8)	0.4 (0 to 0.9)	5.3 (-4.9 to 16.7)	0.2 (-0.1 to 0.7)	5.6 (-5 to 17.6)
Tajikistan	High fasting plasma glucose	0.5 (0.1 to 1.2)	14 (2.5 to 33.1)	0.5 (0.1 to 1.2)	14.5 (2.6 to 34.2)	1.2 (0.2 to 2.6)	28.4 (5.4 to 65.3)	1.1 (0.2 to 2.7)	29.5 (5.6 to 68.1)
Thailand	All risk factors	2.4 (1.4 to 3.4)	67.3 (42 to 96.5)	2.4 (1.3 to 3.7)	69.6 (43.6 to 100.1)	3.6 (2 to 5.8)	98.2 (56.8 to 155.2)	6 (3.1 to 10)	104.2 (60.7 to 165)
Thailand	Behavioral risks	1.2 (0.9 to 1.7)	38.1 (25.8 to 51)	1.3 (0.8 to 2)	39.4 (26.8 to 52.5)	1.2 (0.8 to 1.8)	37.9 (24.1 to 55)	2.3 (1.3 to 3.8)	40.2 (25.4 to 59.1)
Thailand	Alcohol use	0.4 (0.3 to 0.5)	12.3 (9.1 to 16.6)	0.4 (0.3 to 0.6)	12.8 (9.4 to 17.1)	0.5 (0.3 to 0.7)	15 (9.7 to 21.9)	0.9 (0.5 to 1.5)	16 (10.4 to 23.2)
Thailand	Diet high in red meat	0.2 (0 to 0.3)	6.1 (1.2 to 8.9)	0.2 (0 to 0.4)	6.3 (1.2 to 9.1)	0.2 (0 to 0.4)	7.7 (1.5 to 12.6)	0.5 (0.1 to 0.9)	8.2 (1.6 to 13.3)
Thailand	Low physical activity	0.1 (0 to 0.1)	1.8 (1.1 to 3.6)	0.1 (0 to 0.1)	1.9 (1.1 to 3.7)	0.1 (0 to 0.2)	2.1 (1.1 to 4)	0.1 (0.1 to 0.3)	2.2 (1.2 to 4.3)
Thailand	Smoking	0.2 (0.1 to 0.3)	5.7 (3.3 to 8.7)	0.2 (0.1 to 0.4)	5.9 (3.5 to 9.1)	0.1 (0.1 to 0.2)	2.9 (1.6 to 4.6)	0.2 (0.1 to 0.3)	3.1 (1.7 to 4.9)
Thailand	Secondhand smoke	0.4 (0.1 to 0.8)	13.8 (3.2 to 24.9)	0.5 (0.1 to 0.9)	14.3 (3.3 to 25.7)	0.4 (0.1 to 0.7)	11.6 (2.8 to 22)	0.7 (0.2 to 1.4)	12.3 (3 to 23.3)

Location	Risk factor	1990				2019			
		Deaths	YLLs (Years of Life Lost)	YLDs (Years Lived with Disability)	DALYs (Disability-Adjusted Life Years)	Deaths	YLLs (Years of Life Lost)	YLDs (Years Lived with Disability)	DALYs (Disability-Adjusted Life Years)
Thailand	Metabolic risks	1.3 (0.4 to 2.4)	32.7 (11.6 to 62)	1.2 (0.4 to 2.4)	33.9 (12 to 63.9)	2.7 (1.1 to 4.7)	67.1 (26.7 to 119.6)	4.1 (1.7 to 7.3)	71.2 (28.4 to 127)
Thailand	High body-mass index	0.8 (0.2 to 1.8)	21.6 (4.9 to 47.3)	0.8 (0.2 to 1.8)	22.3 (5.2 to 48.5)	2 (0.6 to 3.8)	51.8 (16.2 to 98.5)	3.1 (1 to 6.1)	55 (17.5 to 104.3)
Thailand	High fasting plasma glucose	0.5 (0.1 to 1.2)	12.1 (2.3 to 29.3)	0.4 (0.1 to 1.1)	12.6 (2.3 to 30.2)	0.8 (0.2 to 2)	18.7 (3.5 to 45.8)	1.2 (0.2 to 3)	19.9 (3.7 to 48.2)
Timor-Leste	All risk factors	1.9 (1 to 3.1)	52.9 (29 to 89.8)	1.3 (0.7 to 2.1)	54.2 (29.7 to 91.6)	3.1 (1.6 to 5.1)	82.3 (43.2 to 137.2)	2.5 (1.2 to 4.5)	84.9 (44.9 to 141.2)
Timor-Leste	Behavioral risks	1 (0.5 to 1.6)	31.3 (14.6 to 50)	0.7 (0.3 to 1.2)	32 (14.9 to 51.4)	1.3 (0.7 to 2.1)	39.4 (20.7 to 63.8)	1.2 (0.6 to 2)	40.6 (21.2 to 65.9)
Timor-Leste	Alcohol use	0.1 (0 to 0.1)	2.6 (1.3 to 4.5)	0.1 (0 to 0.1)	2.6 (1.3 to 4.6)	0.2 (0.1 to 0.4)	7.2 (3.8 to 11.8)	0.2 (0.1 to 0.4)	7.4 (3.9 to 12.1)
Timor-Leste	Diet high in red meat	0.2 (0 to 0.3)	5.6 (1 to 9.3)	0.1 (0 to 0.2)	5.8 (1 to 9.5)	0.3 (0 to 0.4)	7.8 (1.5 to 13.7)	0.2 (0 to 0.4)	8 (1.5 to 14.1)
Timor-Leste	Low physical activity	0.1 (0 to 0.2)	2 (1.1 to 4.5)	0.1 (0 to 0.1)	2.1 (1.1 to 4.6)	0.1 (0.1 to 0.2)	2.5 (1.3 to 5.1)	0.1 (0 to 0.2)	2.6 (1.4 to 5.3)
Timor-Leste	Smoking	0.1 (0.1 to 0.2)	3.2 (1.3 to 5.8)	0.1 (0 to 0.2)	3.2 (1.3 to 6)	0.1 (0.1 to 0.2)	3.3 (1.5 to 5.9)	0.1 (0 to 0.2)	3.4 (1.5 to 6)
Timor-Leste	Secondhand smoke	0.6 (0.1 to 1.1)	18.6 (4.3 to 35.6)	0.4 (0.1 to 0.8)	19.1 (4.4 to 36.4)	0.6 (0.1 to 1.2)	19.8 (4.4 to 37.8)	0.6 (0.1 to 1.2)	20.4 (4.5 to 38.8)
Timor-Leste	Metabolic risks	0.9 (0.3 to 2)	23.5 (6.5 to 51.7)	0.6 (0.2 to 1.3)	24.1 (6.7 to 52.9)	1.9 (0.6 to 3.8)	46.7 (14.7 to 94.3)	1.5 (0.5 to 3.1)	48.2 (15.2 to 97)
Timor-Leste	High body-mass index	0.5 (0.1 to 1.3)	13 (2.1 to 34.5)	0.3 (0 to 0.8)	13.3 (2.2 to 35.3)	0.8 (0.2 to 2.1)	22.1 (4.6 to 55)	0.7 (0.1 to 1.7)	22.8 (4.7 to 56.6)
Timor-Leste	High fasting plasma glucose	0.5 (0.1 to 1.1)	11 (1.9 to 26.9)	0.3 (0 to 0.7)	11.3 (1.9 to 27.6)	1.1 (0.2 to 2.7)	26.1 (4.7 to 63.9)	0.8 (0.1 to 2.1)	26.9 (4.8 to 65.8)
Togo	All risk factors	2 (1.4 to 2.8)	53.4 (36.2 to 73.9)	1.4 (0.8 to 2.1)	54.8 (37.1 to 75.6)	2.6 (1.6 to 4.1)	62.6 (37.6 to 97.4)	1.9 (1 to 3.1)	64.5 (38.9 to 100.5)
Togo	Behavioral risks	1.4 (1 to 1.8)	41.3 (29.3 to 54.8)	1 (0.6 to 1.5)	42.3 (30.1 to 56)	1.4 (1 to 2)	40.4 (26.6 to 58.3)	1.1 (0.7 to 1.8)	41.6 (27.3 to 59.9)
Togo	Alcohol use	0.8 (0.5 to 1)	23.6 (15.7 to 32.8)	0.6 (0.3 to 0.9)	24.1 (16.1 to 33.6)	0.7 (0.4 to 1.1)	21.5 (12.6 to 32.4)	0.6 (0.3 to 1)	22.1 (13 to 33.5)
Togo	Diet high in red meat	0.1 (0 to 0.2)	4.2 (1 to 6.6)	0.1 (0 to 0.2)	4.3 (1 to 6.7)	0.2 (0 to 0.3)	4.7 (1.1 to 7.9)	0.1 (0 to 0.2)	4.9 (1.2 to 8.1)
Togo	Low physical activity	0.1 (0.1 to 0.2)	2.9 (1.6 to 5.4)	0.1 (0 to 0.1)	3 (1.6 to 5.5)	0.2 (0.1 to 0.3)	3.6 (1.8 to 6.7)	0.1 (0.1 to 0.2)	3.7 (1.9 to 6.9)
Togo	Smoking	0.2 (0.1 to 0.2)	3.8 (2.2 to 5.6)	0.1 (0.1 to 0.2)	3.9 (2.3 to 5.8)	0.2 (0.1 to 0.3)	3.9 (2.3 to 6.1)	0.1 (0.1 to 0.2)	4 (2.4 to 6.3)
Togo	Secondhand smoke	0.2 (0.1 to 0.4)	8.1 (1.9 to 14.9)	0.2 (0 to 0.4)	8.3 (1.9 to 15.2)	0.2 (0.1 to 0.5)	7.9 (1.7 to 14.9)	0.2 (0 to 0.4)	8.1 (1.8 to 15.3)
Togo	Metabolic risks	0.7 (0.2 to 1.4)	13.4 (2.1 to 30.1)	0.4 (0.1 to 0.9)	13.8 (2.2 to 31)	1.3 (0.4 to 2.7)	24.1 (3.6 to 54.3)	0.8 (0.2 to 1.8)	24.9 (3.9 to 55.8)
Togo	High body-mass index	0.3 (0 to 0.6)	4.1 (-2.8 to 12.1)	0.1 (0 to 0.4)	4.2 (-2.8 to 12.5)	0.6 (0.1 to 1.2)	7.8 (-5.5 to 22.6)	0.3 (-0.1 to 0.7)	8 (-5.7 to 23.1)
Togo	High fasting plasma glucose	0.4 (0.1 to 1)	9.6 (1.8 to 22.8)	0.3 (0 to 0.7)	9.8 (1.8 to 23.5)	0.8 (0.1 to 1.9)	17.1 (2.9 to 41.7)	0.5 (0.1 to 1.4)	17.6 (3 to 43.2)
Tokelau	All risk factors	8.2 (4 to 13.6)	228.5 (114.1 to 378.5)	6.8 (3.4 to 11.9)	235.3 (117.7 to 388.2)	13.1 (7.1 to 21)	345.3 (187.1 to 552.1)	14.2 (7.3 to 23.9)	359.5 (193.8 to 574.2)
Tokelau	Behavioral risks	3.2 (1.6 to 4.9)	97.4 (48.7 to 150.5)	2.8 (1.3 to 4.8)	100.3 (50.5 to 154.4)	4.1 (2.5 to 6.1)	120.8 (71.2 to 183.3)	5 (2.6 to 8)	125.7 (73.9 to 189.4)
Tokelau	Alcohol use	0.3 (0.1 to 0.6)	11.3 (4.1 to 21.9)	0.3 (0.1 to 0.6)	11.6 (4.2 to 22.6)	0.5 (0.2 to 0.9)	18.2 (7.9 to 33.5)	0.7 (0.3 to 1.4)	18.9 (8.2 to 34.7)
Tokelau	Diet high in red meat	0.7 (0.2 to 1.2)	21.3 (5.2 to 37.4)	0.6 (0.1 to 1.2)	21.9 (5.3 to 38.4)	0.9 (0.2 to 1.5)	27.4 (7.3 to 45.7)	1.1 (0.3 to 2)	28.5 (7.6 to 47.6)
Tokelau	Low physical activity	0.4 (0.1 to 0.8)	9.2 (3.2 to 19)	0.3 (0.1 to 0.6)	9.5 (3.3 to 19.8)	0.6 (0.2 to 1.1)	13.4 (5 to 27.2)	0.6 (0.2 to 1.2)	14 (5.2 to 28.3)
Tokelau	Smoking	0.9 (0.4 to 1.5)	25.7 (10.9 to 44.8)	0.8 (0.3 to 1.4)	26.5 (11.3 to 46.1)	1 (0.5 to 1.7)	28 (14.4 to 48.7)	1.2 (0.5 to 2.1)	29.2 (15 to 50.7)
Tokelau	Secondhand smoke	1.1 (0.2 to 2.2)	34.8 (7.8 to 67.9)	1 (0.2 to 2)	35.8 (8 to 69.8)	1.3 (0.3 to 2.5)	40 (9.5 to 77.7)	1.6 (0.4 to 3.3)	41.6 (9.8 to 80.6)
Tokelau	Metabolic risks	5.7 (2.1 to 10.9)	150 (55.4 to 286.8)	4.6 (1.7 to 8.8)	154.6 (57.5 to 294.3)	10.2 (4.4 to 17.9)	257.2 (108 to 450)	10.6 (4.4 to 19.3)	267.8 (112.1 to 466.1)
Tokelau	High body-mass index	4 (1.2 to 8.6)	105.5 (31.8 to 224)	3.2 (0.9 to 6.8)	108.7 (32.6 to 231.8)	6.9 (2.4 to 13.1)	172.2 (59.5 to 318.9)	7 (2.3 to 13.4)	179.3 (62.5 to 332.3)
Tokelau	High fasting plasma glucose	2.1 (0.3 to 5.1)	53.6 (8.5 to 133.8)	1.6 (0.3 to 4.3)	55.2 (8.8 to 137.9)	4.3 (0.8 to 10.1)	109.4 (19.3 to 259.2)	4.5 (0.8 to 11.3)	113.9 (20 to 268.8)
Tonga	All risk factors	10.4 (6.1 to 15.2)	290 (168.2 to 427.1)	8.9 (4.6 to 13.9)	298.9 (173.1 to 440.7)	13.1 (7.1 to 20.1)	346.7 (189.2 to 530.2)	12.9 (6.8 to 21.1)	359.6 (196.2 to 550.6)
Tonga	Behavioral risks	3.2 (2 to 4.5)	93.5 (57.8 to 133)	2.9 (1.6 to 4.4)	96.4 (59.4 to 136.9)	3.5 (2.2 to 5)	99.9 (60.2 to 146.9)	3.7 (2.1 to 6.2)	103.7 (62.2 to 152)
Tonga	Alcohol use	0.1 (0 to 0.3)	5 (1.6 to 9.7)	0.1 (0 to 0.3)	5.1 (1.7 to 10)	0.3 (0.1 to 0.5)	9 (3.1 to 17.8)	0.3 (0.1 to 0.7)	9.4 (3.2 to 18.5)
Tonga	Diet high in red meat	0.7 (0.2 to 1.1)	22.9 (6.4 to 35.5)	0.7 (0.2 to 1.1)	23.5 (6.6 to 36.5)	0.8 (0.2 to 1.3)	24.7 (7 to 39.9)	0.9 (0.3 to 1.6)	25.6 (7.2 to 41.4)
Tonga	Low physical activity	0.5 (0.2 to 0.9)	11 (4.2 to 22.4)	0.4 (0.1 to 0.7)	11.4 (4.3 to 23.3)	0.6 (0.2 to 1.1)	13.6 (4.8 to 27.2)	0.5 (0.2 to 1.1)	14.1 (5 to 28.4)
Tonga	Smoking	0.9 (0.5 to 1.3)	21.9 (11.6 to 34.1)	0.7 (0.4 to 1.2)	22.6 (12.1 to 35.2)	0.8 (0.5 to 1.3)	20.2 (10.7 to 32.4)	0.8 (0.4 to 1.3)	21 (11.1 to 33.7)

Location	Risk factor	1990				2019			
		Deaths	YLLs (Years of Life Lost)	YLDs (Years Lived with Disability)	DALYs (Disability-Adjusted Life Years)	Deaths	YLLs (Years of Life Lost)	YLDs (Years Lived with Disability)	DALYs (Disability-Adjusted Life Years)
Tonga	Secondhand smoke	1.2 (0.3 to 2.1)	36.7 (8.2 to 66.3)	1.1 (0.2 to 2.1)	37.8 (8.5 to 68.1)	1.2 (0.3 to 2.2)	36.8 (9.3 to 67.7)	1.3 (0.3 to 2.6)	38.1 (9.7 to 70)
Tonga	Metabolic risks	8.1 (3.5 to 13.1)	221.4 (99 to 360.5)	6.8 (2.8 to 11.4)	228.2 (102.3 to 370.9)	10.7 (4.8 to 17.9)	277.3 (123.6 to 464.9)	10.3 (4.6 to 18)	287.6 (129 to 480.1)
Tonga	High body-mass index	6.2 (2.2 to 11)	173.8 (62.4 to 302)	5.3 (1.8 to 9.4)	179.1 (64.3 to 311.2)	7.9 (2.9 to 13.9)	206.4 (75.6 to 364.6)	7.6 (2.7 to 14.2)	214.1 (78.6 to 375.3)
Tonga	High fasting plasma glucose	2.4 (0.5 to 5.7)	63.8 (12 to 150)	2 (0.4 to 4.9)	65.8 (12.4 to 154.4)	3.9 (0.8 to 9.1)	98.2 (18.3 to 231.8)	3.7 (0.7 to 9.4)	101.9 (18.9 to 240.4)
Trinidad and Tobago	All risk factors	7.4 (4.8 to 10.6)	186.2 (119.9 to 266.2)	7.7 (4.5 to 12.1)	193.9 (124.4 to 276.3)	7 (4.1 to 11.4)	170.6 (99.3 to 277.7)	9.3 (5 to 15.8)	179.9 (104.7 to 290.6)
Trinidad and Tobago	Behavioral risks	3.9 (3.1 to 4.8)	116 (91.7 to 140.6)	4.5 (3.1 to 6.2)	120.5 (95.5 to 145.6)	3.4 (2.4 to 4.8)	99.9 (67.5 to 140.6)	5.2 (3.2 to 8)	105.1 (70.8 to 147.7)
Trinidad and Tobago	Alcohol use	1.8 (1.4 to 2.3)	58.8 (45.4 to 73.3)	2.2 (1.5 to 3.2)	61 (47.1 to 76.5)	1.6 (1.1 to 2.3)	51.3 (33.5 to 73.5)	2.6 (1.5 to 4.1)	53.9 (35.3 to 77.5)
Trinidad and Tobago	Diet high in red meat	0.4 (0.1 to 0.6)	12.5 (2.3 to 17.1)	0.5 (0.1 to 0.8)	12.9 (2.4 to 17.7)	0.4 (0.1 to 0.7)	12.6 (2.4 to 20.8)	0.7 (0.1 to 1.2)	13.2 (2.5 to 21.9)
Trinidad and Tobago	Low physical activity	0.6 (0.2 to 1)	12.4 (4.2 to 23.8)	0.5 (0.2 to 1)	12.9 (4.4 to 24.7)	0.6 (0.2 to 1)	12.7 (3.9 to 24.6)	0.7 (0.2 to 1.4)	13.4 (4.2 to 25.8)
Trinidad and Tobago	Smoking	0.8 (0.5 to 1.1)	21 (13.1 to 30.6)	0.8 (0.5 to 1.4)	21.8 (13.7 to 31.9)	0.5 (0.3 to 0.8)	13.5 (7.7 to 21.2)	0.7 (0.4 to 1.2)	14.3 (8.2 to 22.4)
Trinidad and Tobago	Secondhand smoke	0.6 (0.1 to 1)	18 (4.1 to 31.4)	0.7 (0.2 to 1.3)	18.7 (4.3 to 32.7)	0.5 (0.1 to 1)	15.6 (3.8 to 29.5)	0.8 (0.2 to 1.6)	16.4 (4 to 30.9)
Trinidad and Tobago	Metabolic risks	4.1 (1.3 to 7.8)	82 (14.1 to 171.6)	3.7 (0.9 to 7.9)	85.7 (15 to 179.6)	4.2 (1.4 to 8.4)	83 (13.1 to 184.2)	4.8 (1.2 to 10.6)	87.8 (14.2 to 192.7)
Trinidad and Tobago	High body-mass index	1.1 (0 to 2.4)	10 (-19.7 to 39.4)	0.7 (-0.5 to 2)	10.7 (-20.3 to 41.3)	1.3 (0.1 to 2.9)	14.9 (-17.9 to 50.2)	1.1 (-0.7 to 3.1)	16 (-18.7 to 53.2)
Trinidad and Tobago	High fasting plasma glucose	3.2 (0.7 to 6.8)	76.1 (15.2 to 163.6)	3.2 (0.6 to 7.3)	79.3 (15.9 to 169.4)	3.2 (0.6 to 7.3)	73.6 (14.7 to 172.2)	4.1 (0.8 to 9.9)	77.6 (15.4 to 181.6)
Tunisia	All risk factors	2.2 (1.2 to 3.6)	53.7 (27.9 to 87.4)	2.3 (1.1 to 3.9)	56 (29.1 to 91.3)	3.3 (1.7 to 5.7)	75.2 (35.7 to 133.8)	5.1 (2.3 to 9.9)	80.3 (38.2 to 142.4)
Tunisia	Behavioral risks	1.1 (0.6 to 1.6)	33.9 (18.7 to 49.3)	1.4 (0.7 to 2.2)	35.2 (19.3 to 51.1)	1.3 (0.7 to 1.9)	38.6 (22.1 to 59.2)	2.6 (1.3 to 4.3)	41.2 (23.4 to 63.3)
Tunisia	Alcohol use	0.1 (0.1 to 0.2)	4.7 (3.1 to 6.7)	0.2 (0.1 to 0.3)	4.9 (3.2 to 6.9)	0.2 (0.1 to 0.4)	8.6 (5.4 to 12.9)	0.6 (0.3 to 1)	9.2 (5.7 to 13.8)
Tunisia	Diet high in red meat	0.3 (0.1 to 0.4)	7.9 (1.5 to 12)	0.3 (0.1 to 0.5)	8.2 (1.6 to 12.4)	0.3 (0.1 to 0.5)	8.4 (1.5 to 13.9)	0.6 (0.1 to 1)	9 (1.6 to 14.9)
Tunisia	Low physical activity	0.1 (0.1 to 0.2)	2.4 (1.4 to 4.5)	0.1 (0.1 to 0.2)	2.5 (1.4 to 4.7)	0.1 (0.1 to 0.2)	3 (1.5 to 5.5)	0.2 (0.1 to 0.4)	3.2 (1.6 to 6)
Tunisia	Smoking	0.1 (0.1 to 0.2)	3.3 (1.9 to 5.2)	0.1 (0.1 to 0.2)	3.4 (2 to 5.4)	0.1 (0.1 to 0.2)	3.1 (1.8 to 5)	0.2 (0.1 to 0.4)	3.4 (2 to 5.4)
Tunisia	Secondhand smoke	0.5 (0.1 to 0.9)	16.6 (3.7 to 29)	0.7 (0.1 to 1.2)	17.2 (3.9 to 30)	0.5 (0.1 to 1)	16.7 (3.9 to 32.3)	1.1 (0.3 to 2.2)	17.9 (4.1 to 34.3)
Tunisia	Metabolic risks	1.2 (0.3 to 2.6)	21.4 (-0.4 to 52.9)	1 (0.1 to 2.4)	22.4 (-0.3 to 55.4)	2.2 (0.7 to 4.5)	39.5 (2.7 to 93)	2.7 (0.1 to 6.6)	42.1 (2.8 to 99)
Tunisia	High body-mass index	0.5 (0 to 1.1)	4 (-9.4 to 17.4)	0.2 (-0.3 to 0.8)	4.2 (-9.7 to 18.2)	0.7 (-0.1 to 1.7)	5.4 (-15.7 to 27)	0.3 (-1.3 to 1.8)	5.7 (-16.9 to 28.7)
Tunisia	High fasting plasma glucose	0.8 (0.2 to 1.9)	18.4 (3.3 to 44.2)	0.8 (0.1 to 2)	19.2 (3.5 to 46.1)	1.6 (0.3 to 3.7)	36.4 (7 to 88.9)	2.5 (0.5 to 6.5)	39 (7.5 to 95)
Turkey	All risk factors	2.5 (1.6 to 3.7)	65.5 (39.3 to 97.2)	2.3 (1.3 to 3.7)	67.8 (40.6 to 100.3)	2.9 (1.7 to 4.5)	65.3 (36.7 to 101.1)	4.4 (2.3 to 7.3)	69.8 (39.3 to 107.1)
Turkey	Behavioral risks	1.5 (1 to 2.1)	48.1 (32.1 to 66.3)	1.6 (0.9 to 2.4)	49.7 (33.3 to 68.5)	1.5 (1 to 2)	41.8 (28.2 to 56.3)	2.8 (1.7 to 4.4)	44.6 (30.1 to 60.7)
Turkey	Alcohol use	0.3 (0.2 to 0.4)	9.2 (6.4 to 13)	0.3 (0.2 to 0.5)	9.5 (6.6 to 13.4)	0.3 (0.2 to 0.4)	8.7 (5.7 to 12.5)	0.6 (0.4 to 0.9)	9.3 (6.1 to 13.4)
Turkey	Diet high in red meat	0.2 (0 to 0.4)	7.6 (1.5 to 11.6)	0.2 (0 to 0.4)	7.8 (1.6 to 12)	0.2 (0 to 0.4)	7 (1.5 to 10.6)	0.5 (0.1 to 0.8)	7.4 (1.6 to 11.4)
Turkey	Low physical activity	0.2 (0.1 to 0.3)	4.3 (1.9 to 8)	0.2 (0.1 to 0.3)	4.4 (2 to 8.3)	0.2 (0.1 to 0.4)	5.1 (2 to 9.6)	0.4 (0.1 to 0.7)	5.4 (2.2 to 10.3)
Turkey	Smoking	0.4 (0.3 to 0.7)	14.2 (8.7 to 22)	0.5 (0.2 to 0.8)	14.7 (9 to 22.7)	0.4 (0.3 to 0.6)	12.5 (7.7 to 17.9)	0.8 (0.4 to 1.3)	13.3 (8.1 to 19.1)
Turkey	Secondhand smoke	0.5 (0.1 to 0.9)	15.5 (3.4 to 28.6)	0.5 (0.1 to 1)	16 (3.5 to 29.7)	0.4 (0.1 to 0.7)	10.7 (2.4 to 19.6)	0.7 (0.2 to 1.4)	11.5 (2.6 to 20.8)
Turkey	Metabolic risks	1.1 (0.3 to 2.2)	20 (-0.6 to 47.7)	0.8 (0 to 1.8)	20.9 (-0.5 to 49.3)	1.7 (0.5 to 3.2)	26.8 (1.6 to 60.2)	1.8 (0.1 to 4.2)	28.6 (1.8 to 63.5)
Turkey	High body-mass index	0.5 (0 to 1.1)	4.6 (-10 to 19)	0.3 (-0.2 to 0.8)	4.9 (-10.3 to 19.8)	0.8 (0.1 to 1.6)	7.2 (-10.5 to 24.9)	0.4 (-0.8 to 1.7)	7.6 (-11.2 to 26.3)
Turkey	High fasting plasma glucose	0.7 (0.1 to 1.6)	16.4 (3 to 39.4)	0.6 (0.1 to 1.5)	17 (3.1 to 40.8)	1 (0.2 to 2.3)	21.4 (3.9 to 50.9)	1.5 (0.3 to 3.9)	22.9 (4.2 to 54.7)
Turkmenistan	All risk factors	2.3 (1.5 to 3.1)	64.5 (41.2 to 88.1)	2.3 (1.3 to 3.5)	66.9 (43.1 to 90.7)	2.7 (1.7 to 4)	76.5 (45.5 to 114.9)	3.6 (2 to 5.9)	80.2 (47.8 to 120)
Turkmenistan	Behavioral risks	1.5 (1 to 2)	50.5 (34.2 to 64.7)	1.7 (1 to 2.5)	52.2 (35.4 to 67.1)	1.8 (1.2 to 2.5)	60.5 (40.6 to 82.7)	2.8 (1.7 to 4.3)	63.3 (42.2 to 86.7)
Turkmenistan	Alcohol use	0.2 (0.1 to 0.4)	9 (5.2 to 13.7)	0.3 (0.2 to 0.5)	9.3 (5.4 to 14.1)	0.5 (0.3 to 0.7)	18.5 (12 to 26.6)	0.8 (0.5 to 1.4)	19.3 (12.5 to 27.9)
Turkmenistan	Diet high in red meat	0.6 (0.3 to 0.8)	20.1 (9.2 to 26.8)	0.7 (0.3 to 1.1)	20.8 (9.5 to 27.7)	0.7 (0.3 to 1.1)	24 (11.2 to 37)	1.1 (0.5 to 1.9)	25.1 (11.7 to 38.7)
Turkmenistan	Low physical activity	0.1 (0.1 to 0.2)	2.2 (1.4 to 4.3)	0.1 (0 to 0.2)	2.3 (1.5 to 4.5)	0.1 (0.1 to 0.2)	2.4 (1.4 to 4.6)	0.1 (0.1 to 0.2)	2.5 (1.5 to 4.9)
Turkmenistan	Smoking	0.2 (0.1 to 0.3)	6.1 (3.7 to 9.2)	0.2 (0.1 to 0.3)	6.3 (3.8 to 9.6)	0.1 (0.1 to 0.2)	4.9 (2.6 to 7.7)	0.2 (0.1 to 0.4)	5.1 (2.7 to 8.1)

Location	Risk factor	1990				2019			
		Deaths	YLLs (Years of Life Lost)	YLDs (Years Lived with Disability)	DALYs (Disability-Adjusted Life Years)	Deaths	YLLs (Years of Life Lost)	YLDs (Years Lived with Disability)	DALYs (Disability-Adjusted Life Years)
Turkmenistan	Secondhand smoke	0.5 (0.1 to 0.8)	15.4 (3.7 to 26.5)	0.5 (0.1 to 1)	15.9 (3.8 to 27.3)	0.4 (0.1 to 0.8)	14.3 (3.1 to 25.7)	0.7 (0.1 to 1.3)	15 (3.3 to 26.7)
Turkmenistan	Metabolic risks	0.8 (0.2 to 1.7)	15.9 (-1.5 to 38.2)	0.7 (0 to 1.5)	16.6 (-1.5 to 39.7)	1.1 (0.2 to 2.2)	18.5 (-7.1 to 51.7)	1 (-0.3 to 2.5)	19.5 (-7.6 to 53.8)
Turkmenistan	High body-mass index	0.4 (0 to 1)	3.9 (-9.2 to 16.8)	0.2 (-0.2 to 0.8)	4.1 (-9.5 to 17.6)	0.4 (-0.2 to 1)	-1.5 (-19.7 to 14.3)	0 (-1 to 0.8)	-1.5 (-20.9 to 14.9)
Turkmenistan	High fasting plasma glucose	0.5 (0.1 to 1.1)	12.5 (2.3 to 29.2)	0.5 (0.1 to 1.2)	13 (2.4 to 30.3)	0.7 (0.1 to 1.8)	20.9 (3.8 to 50.7)	1 (0.2 to 2.6)	21.9 (4 to 52.9)
Tuvalu	All risk factors	8.1 (4.4 to 12.7)	229.3 (128.4 to 357.3)	6.3 (3.5 to 10.1)	235.6 (131.6 to 366.5)	12.7 (6.6 to 20.5)	336.7 (174.7 to 545.9)	11.7 (5.9 to 19.8)	348.4 (183.3 to 561.2)
Tuvalu	Behavioral risks	3.4 (2.1 to 5)	105.1 (63.2 to 155.8)	2.8 (1.6 to 4.4)	108 (65 to 159.7)	4.2 (2.5 to 6.4)	120.6 (71.9 to 189.6)	4.2 (2.3 to 6.9)	124.7 (74.1 to 195.6)
Tuvalu	Alcohol use	0.2 (0.1 to 0.4)	8.8 (4 to 15.3)	0.2 (0.1 to 0.4)	9.1 (4.1 to 15.7)	0.3 (0.1 to 0.6)	11.7 (4.9 to 21.5)	0.4 (0.2 to 0.8)	12.1 (5.1 to 22.2)
Tuvalu	Diet high in red meat	0.7 (0.2 to 1.1)	21.6 (5.4 to 34.5)	0.6 (0.1 to 1)	22.2 (5.6 to 35.4)	0.9 (0.2 to 1.5)	25.9 (6.7 to 45.1)	0.9 (0.2 to 1.6)	26.8 (6.9 to 46.6)
Tuvalu	Low physical activity	0.4 (0.2 to 0.8)	9.6 (3.7 to 19.9)	0.3 (0.1 to 0.6)	9.9 (3.8 to 20.3)	0.6 (0.2 to 1.2)	13.1 (4.6 to 28.4)	0.5 (0.2 to 1.1)	13.6 (4.8 to 29.5)
Tuvalu	Smoking	1.1 (0.6 to 1.8)	32.2 (16.4 to 52.8)	0.9 (0.4 to 1.6)	33.1 (16.9 to 54.1)	1.3 (0.6 to 2.1)	34.1 (16.4 to 58.2)	1.2 (0.6 to 2.1)	35.3 (16.9 to 60.1)
Tuvalu	Secondhand smoke	1.2 (0.3 to 2.2)	38 (8.4 to 69.2)	1 (0.2 to 1.9)	39 (8.6 to 70.9)	1.4 (0.3 to 2.7)	41.6 (8.6 to 82.9)	1.4 (0.3 to 2.8)	43 (8.9 to 85.1)
Tuvalu	Metabolic risks	5.3 (2 to 9.9)	142.3 (52.3 to 264.7)	4 (1.5 to 7.4)	146.3 (53.7 to 271)	9.8 (3.8 to 17.5)	246.8 (94.5 to 447.8)	8.6 (3.3 to 16.6)	255.5 (98.2 to 463.2)
Tuvalu	High body-mass index	3.5 (1 to 7.5)	94.5 (27.7 to 197.8)	2.7 (0.7 to 5.6)	97.1 (28.5 to 203.3)	6 (1.8 to 12)	152.9 (46.9 to 299.9)	5.3 (1.6 to 10.5)	158.2 (49 to 312.7)
Tuvalu	High fasting plasma glucose	2.1 (0.4 to 5)	55.5 (10.6 to 133.6)	1.6 (0.3 to 3.9)	57.1 (11 to 136.6)	4.6 (1 to 10.9)	116.1 (23.6 to 280.7)	4.1 (0.8 to 9.8)	120.2 (24.5 to 290.4)
Uganda	All risk factors	2.3 (1.5 to 3.4)	63 (40.1 to 91)	1.5 (0.9 to 2.4)	64.5 (41.1 to 93.5)	4.3 (2.8 to 6.3)	114.9 (75.8 to 168.1)	3.3 (1.9 to 5.3)	118.2 (77.8 to 172.6)
Uganda	Behavioral risks	1.6 (1.1 to 2.2)	49.7 (32.4 to 69.1)	1.1 (0.7 to 1.7)	50.9 (33.1 to 70.6)	2.8 (2 to 3.9)	85.7 (58.3 to 119.7)	2.3 (1.4 to 3.5)	88.1 (60 to 123.1)
Uganda	Alcohol use	1.1 (0.7 to 1.5)	33.7 (21.7 to 47.9)	0.8 (0.4 to 1.2)	34.5 (22.1 to 49.2)	1.9 (1.3 to 2.8)	60.9 (40.7 to 88.5)	1.6 (1 to 2.6)	62.5 (41.6 to 90.7)
Uganda	Diet high in red meat	0.2 (0 to 0.3)	5.7 (1.1 to 9.2)	0.1 (0 to 0.2)	5.8 (1.1 to 9.4)	0.3 (0.1 to 0.5)	10.5 (2 to 16.5)	0.3 (0.1 to 0.5)	10.8 (2 to 17)
Uganda	Low physical activity	0.1 (0 to 0.1)	1.7 (1 to 4)	0 (0 to 0.1)	1.7 (1 to 4.1)	0.1 (0.1 to 0.2)	2.6 (1.6 to 6.2)	0.1 (0 to 0.2)	2.7 (1.6 to 6.3)
Uganda	Smoking	0.1 (0.1 to 0.2)	2.9 (1.3 to 4.9)	0.1 (0 to 0.1)	3 (1.3 to 5.1)	0.2 (0.1 to 0.4)	4.7 (2.1 to 8)	0.1 (0.1 to 0.3)	4.9 (2.2 to 8.3)
Uganda	Secondhand smoke	0.2 (0.1 to 0.4)	7.5 (1.7 to 14.2)	0.2 (0 to 0.3)	7.6 (1.7 to 14.4)	0.3 (0.1 to 0.6)	10 (2.2 to 18.9)	0.3 (0.1 to 0.5)	10.3 (2.3 to 19.4)
Uganda	Metabolic risks	0.7 (0.2 to 1.6)	14.9 (2.5 to 35.4)	0.4 (0.1 to 1)	15.3 (2.5 to 36.3)	1.7 (0.5 to 3.5)	33.2 (3.8 to 77.7)	1 (0.2 to 2.4)	34.2 (4.1 to 79.8)
Uganda	High body-mass index	0.1 (0 to 0.4)	1.4 (-2.7 to 6.7)	0.1 (0 to 0.2)	1.4 (-2.7 to 6.9)	0.5 (0 to 1.2)	5.8 (-10.7 to 22.7)	0.2 (-0.2 to 0.7)	6 (-10.9 to 23.3)
Uganda	High fasting plasma glucose	0.6 (0.1 to 1.5)	13.7 (2.5 to 33.5)	0.4 (0.1 to 0.9)	14.1 (2.6 to 34.4)	1.2 (0.2 to 2.9)	28.3 (5.2 to 67.5)	0.8 (0.1 to 2.1)	29.1 (5.4 to 69.6)
Ukraine	All risk factors	6 (4.6 to 7.5)	183.8 (138.3 to 226.4)	7.3 (4.7 to 10.5)	191.1 (144.1 to 235.7)	5.3 (3.6 to 7.4)	153.2 (104 to 213.7)	7.8 (4.7 to 11.8)	161.1 (110 to 223.5)
Ukraine	Behavioral risks	4.5 (3.6 to 5.3)	155 (124 to 183.2)	6 (3.9 to 8.3)	161 (128.9 to 190.5)	3.5 (2.5 to 4.7)	117.1 (81.1 to 156.4)	5.9 (3.7 to 8.8)	123 (86.2 to 163)
Ukraine	Alcohol use	2.3 (1.8 to 2.8)	83.1 (66.6 to 101.5)	3.2 (2 to 4.5)	86.3 (69.2 to 105.1)	2 (1.4 to 2.7)	68.8 (47.6 to 94)	3.5 (2.1 to 5.3)	72.3 (50.4 to 97.5)
Ukraine	Diet high in red meat	1 (0.4 to 1.3)	32.3 (13 to 42.5)	1.3 (0.5 to 2)	33.5 (13.5 to 44.2)	0.6 (0.2 to 1)	19.9 (6.7 to 30.7)	1 (0.3 to 1.7)	20.9 (7.1 to 31.9)
Ukraine	Low physical activity	0.1 (0.1 to 0.3)	3.6 (2.4 to 7.5)	0.2 (0.1 to 0.3)	3.7 (2.5 to 7.8)	0.1 (0.1 to 0.2)	2.8 (1.7 to 5.8)	0.2 (0.1 to 0.3)	2.9 (1.8 to 6.1)
Ukraine	Smoking	0.6 (0.4 to 0.9)	21.4 (11.3 to 32.5)	0.8 (0.4 to 1.4)	22.2 (11.8 to 33.7)	0.5 (0.3 to 0.8)	16.8 (9 to 26.5)	0.8 (0.4 to 1.4)	17.6 (9.4 to 27.7)
Ukraine	Secondhand smoke	0.8 (0.2 to 1.4)	26.7 (6.6 to 46.4)	1 (0.2 to 1.9)	27.8 (6.8 to 48.1)	0.5 (0.1 to 1)	17.4 (4.2 to 32)	0.9 (0.2 to 1.8)	18.3 (4.4 to 34)
Ukraine	Metabolic risks	1.8 (0.5 to 3.5)	32.8 (-5 to 75.3)	1.5 (-0.1 to 3.3)	34.2 (-5.1 to 78.4)	2 (0.7 to 3.9)	42.3 (8.1 to 87.7)	2.2 (0.4 to 4.5)	44.5 (8.3 to 91.6)
Ukraine	High body-mass index	1.1 (0 to 2.4)	12.6 (-17.5 to 43.1)	0.7 (-0.6 to 2)	13.3 (-18.2 to 45.2)	1.3 (0.3 to 2.7)	22.4 (-5 to 54)	1.2 (-0.2 to 2.9)	23.5 (-5.2 to 56.6)
Ukraine	High fasting plasma glucose	0.8 (0.1 to 1.8)	21.2 (3.9 to 49.6)	0.9 (0.2 to 2.2)	22.1 (4.1 to 51.6)	0.8 (0.2 to 2)	21.5 (4 to 53)	1.1 (0.2 to 3.1)	22.7 (4.1 to 55.3)
United Arab Emirates	All risk factors	7 (3.7 to 12.3)	175.8 (96.5 to 297)	6.5 (3.4 to 11.4)	182.3 (100.6 to 307.6)	7.7 (4.2 to 12.5)	184 (95.4 to 304.4)	9.2 (4.7 to 16.1)	193.2 (100.3 to 320.5)
United Arab Emirates	Behavioral risks	3.4 (2.1 to 5.3)	100 (62.4 to 150)	3.5 (2.1 to 5.6)	103.6 (64.9 to 155.5)	2.8 (1.8 to 4)	82.3 (51.3 to 118.3)	4.1 (2.4 to 6.4)	86.3 (53.8 to 124.7)
United Arab Emirates	Alcohol use	0.7 (0.4 to 1.1)	22.1 (14 to 34.4)	0.7 (0.4 to 1.2)	22.9 (14.5 to 35.5)	0.3 (0.2 to 0.5)	11.6 (6.5 to 18.4)	0.6 (0.3 to 1)	12.2 (6.8 to 19.3)
United Arab Emirates	Diet high in red meat	1.2 (0.5 to 2)	35.1 (15.7 to 56.7)	1.2 (0.5 to 2.1)	36.4 (16.3 to 58.8)	0.7 (0.2 to 1.2)	21.4 (5.6 to 34.1)	1.1 (0.3 to 1.9)	22.5 (5.9 to 36)
United Arab Emirates	Low physical activity	0.7 (0.2 to 1.3)	16.1 (5.6 to 30.5)	0.6 (0.2 to 1.2)	16.7 (5.8 to 31.7)	0.7 (0.3 to 1.3)	18.2 (6.6 to 32.8)	0.9 (0.3 to 1.8)	19.2 (6.9 to 34.3)

Location	Risk factor	1990				2019			
		Deaths	YLLs (Years of Life Lost)	YLDs (Years Lived with Disability)	DALYs (Disability-Adjusted Life Years)	Deaths	YLLs (Years of Life Lost)	YLDs (Years Lived with Disability)	DALYs (Disability-Adjusted Life Years)
United Arab Emirates	Smoking	0.3 (0.2 to 0.5)	8.1 (4.2 to 14)	0.3 (0.1 to 0.5)	8.4 (4.4 to 14.5)	0.3 (0.2 to 0.5)	9.2 (5.2 to 15)	0.4 (0.2 to 0.8)	9.6 (5.4 to 15.7)
United Arab Emirates	Secondhand smoke	0.8 (0.2 to 1.6)	24.4 (5.8 to 46.6)	0.9 (0.2 to 1.7)	25.2 (6.1 to 48.1)	0.8 (0.2 to 1.6)	25.5 (6 to 47.2)	1.2 (0.3 to 2.4)	26.8 (6.4 to 49.4)
United Arab Emirates	Metabolic risks	4.1 (1.3 to 8.8)	87.7 (20.3 to 194.5)	3.4 (0.9 to 7.4)	91.1 (21.2 to 201.2)	5.5 (1.9 to 10.3)	114.3 (24.5 to 237.7)	5.8 (1.3 to 12.2)	120.2 (25.6 to 249.9)
United Arab Emirates	High body-mass index	1.6 (0.2 to 3.6)	29 (-7 to 76.7)	1.1 (-0.2 to 2.9)	30.1 (-7 to 79.1)	2 (0.1 to 4.2)	31.1 (-21.3 to 88.2)	1.5 (-1.1 to 4.2)	32.6 (-22.2 to 92.3)
United Arab Emirates	High fasting plasma glucose	2.8 (0.5 to 7)	64.3 (11.6 to 162.1)	2.5 (0.4 to 6.3)	66.8 (12.1 to 168.1)	3.9 (0.8 to 8.4)	93.8 (19.2 to 207.8)	4.9 (1 to 11.2)	98.7 (20.3 to 218.9)
United Kingdom	All risk factors	12.6 (10.4 to 15.1)	328.3 (273 to 383.8)	24.6 (16.5 to 34.2)	353 (291.6 to 412.5)	8.2 (6.3 to 10.5)	187.2 (145.8 to 234.3)	24.8 (15.1 to 37.6)	212 (164.6 to 267.1)
United Kingdom	Behavioral risks	10 (8.6 to 11.4)	281.6 (242.4 to 317.5)	20.9 (14.2 to 29.3)	302.6 (258.3 to 340.5)	5.5 (4.7 to 6.4)	141.9 (120.5 to 163.3)	18.5 (11.8 to 28)	160.4 (135.7 to 185.6)
United Kingdom	Alcohol use	4.4 (3.6 to 5.4)	130.3 (107 to 155.8)	9.6 (6.3 to 13.6)	139.9 (114.9 to 166.9)	2.7 (2.1 to 3.4)	72.4 (57.9 to 87.7)	9.4 (5.8 to 14.4)	81.8 (65.1 to 99.6)
United Kingdom	Diet high in red meat	1.6 (0.7 to 2.1)	43.9 (21.3 to 58)	3.3 (1.4 to 5.1)	47.2 (22.6 to 62.6)	0.8 (0.4 to 1.1)	21.5 (9.7 to 29.1)	2.8 (1.1 to 4.6)	24.3 (11.1 to 32.9)
United Kingdom	Low physical activity	0.6 (0.2 to 1.1)	14 (4.6 to 26.8)	1.1 (0.4 to 2.3)	15.1 (5 to 28.9)	0.5 (0.2 to 0.8)	9.4 (3.1 to 17.3)	1.3 (0.4 to 2.6)	10.7 (3.6 to 19.5)
United Kingdom	Smoking	3.9 (2.9 to 5)	107.3 (79.7 to 135.8)	8 (4.9 to 11.7)	115.3 (85.1 to 146.8)	1.8 (1.3 to 2.3)	44.2 (32.5 to 56.7)	5.8 (3.4 to 9)	50 (36.4 to 64.5)
United Kingdom	Secondhand smoke	0.7 (0.1 to 1.2)	20.8 (4.6 to 36)	1.5 (0.3 to 2.7)	22.3 (5 to 38.6)	0.3 (0.1 to 0.5)	9.2 (2.1 to 16)	1.2 (0.2 to 2.2)	10.4 (2.4 to 18)
United Kingdom	Metabolic risks	3.5 (1.4 to 6.6)	65 (16 to 129.7)	5.2 (1.3 to 10.3)	70.2 (17.6 to 139)	3.5 (1.3 to 6.4)	60.7 (16.5 to 120.1)	8.4 (2.5 to 17.7)	69 (19.1 to 136.2)
United Kingdom	High body-mass index	1.6 (0.4 to 3.4)	25.1 (-4.9 to 60.7)	2.1 (-0.2 to 4.8)	27.1 (-5.4 to 64.9)	1.3 (0.3 to 2.6)	17.5 (-3.5 to 41.8)	2.6 (-0.2 to 5.9)	20.1 (-3.6 to 46.9)
United Kingdom	High fasting plasma glucose	2 (0.4 to 4.5)	42.4 (7.8 to 96.1)	3.3 (0.6 to 8.1)	45.7 (8.4 to 103.4)	2.3 (0.5 to 5.1)	46 (9.2 to 101.5)	6.2 (1.2 to 15.3)	52.2 (10.4 to 116.5)
United Republic of Tanzania	All risk factors	2.3 (1.6 to 3.1)	54.7 (38.1 to 74.7)	1.4 (0.9 to 2.1)	56.1 (39 to 76.7)	3.4 (2.3 to 4.9)	79.1 (52.3 to 113.1)	2.3 (1.4 to 3.6)	81.4 (53.9 to 116.3)
United Republic of Tanzania	Behavioral risks	1.7 (1.2 to 2.2)	44.6 (32.2 to 59.2)	1.1 (0.7 to 1.6)	45.7 (33.1 to 60.4)	2.2 (1.6 to 3)	57.4 (40.8 to 77.9)	1.6 (1 to 2.5)	59 (42.1 to 79.8)
United Republic of Tanzania	Alcohol use	0.8 (0.6 to 1.2)	23.5 (16 to 32.9)	0.6 (0.3 to 0.8)	24.1 (16.4 to 33.8)	1.1 (0.8 to 1.5)	29.9 (20.8 to 41.3)	0.8 (0.5 to 1.3)	30.8 (21.3 to 42.6)
United Republic of Tanzania	Diet high in red meat	0.2 (0 to 0.3)	5 (0.9 to 7.7)	0.1 (0 to 0.2)	5.2 (0.9 to 7.9)	0.2 (0 to 0.4)	6.2 (1.2 to 9.9)	0.2 (0 to 0.3)	6.4 (1.2 to 10.1)
United Republic of Tanzania	Low physical activity	0.1 (0 to 0.1)	1.5 (0.9 to 3.3)	0 (0 to 0.1)	1.5 (0.9 to 3.4)	0.1 (0.1 to 0.2)	2 (1.2 to 4.6)	0.1 (0 to 0.1)	2.1 (1.3 to 4.7)
United Republic of Tanzania	Smoking	0.4 (0.2 to 0.6)	8.2 (3.6 to 13.2)	0.2 (0.1 to 0.4)	8.5 (3.7 to 13.5)	0.5 (0.3 to 0.9)	11.3 (5.1 to 18.6)	0.3 (0.1 to 0.6)	11.7 (5.3 to 19.1)
United Republic of Tanzania	Secondhand smoke	0.3 (0.1 to 0.5)	8.4 (2 to 15.2)	0.2 (0 to 0.4)	8.6 (2 to 15.6)	0.4 (0.1 to 0.6)	10.5 (2.3 to 18.7)	0.3 (0.1 to 0.6)	10.8 (2.4 to 19.2)
United Republic of Tanzania	Metabolic risks	0.7 (0.2 to 1.4)	11.8 (2.1 to 26.6)	0.3 (0.1 to 0.7)	12.1 (2.1 to 27.4)	1.4 (0.5 to 2.7)	25.1 (4.6 to 53.9)	0.8 (0.2 to 1.7)	25.9 (4.7 to 55.5)
United Republic of Tanzania	High body-mass index	0.3 (0 to 0.7)	4.3 (-1.6 to 12.4)	0.1 (0 to 0.4)	4.4 (-1.6 to 12.8)	0.6 (0.1 to 1.3)	8.9 (-4 to 23.9)	0.3 (-0.1 to 0.8)	9.2 (-4.2 to 24.6)
United Republic of Tanzania	High fasting plasma glucose	0.4 (0.1 to 1)	7.7 (1.4 to 18.6)	0.2 (0 to 0.5)	7.9 (1.4 to 19.1)	0.8 (0.2 to 1.9)	16.9 (3.2 to 40.3)	0.5 (0.1 to 1.3)	17.4 (3.3 to 41.7)
United States Virgin Islands	All risk factors	7.1 (4.3 to 10.6)	169.3 (95 to 254.8)	8.3 (4.4 to 13.6)	177.6 (100.2 to 268)	7.9 (4.6 to 11.8)	173.4 (96.3 to 268.1)	11.4 (5.9 to 19.2)	184.8 (103.4 to 284.2)
United States Virgin Islands	Behavioral risks	3.9 (2.1 to 5.5)	111.1 (57 to 160.2)	5.1 (2.5 to 8.3)	116.3 (59.5 to 167.8)	3.8 (2.1 to 5.5)	102.1 (51.9 to 150.2)	6.4 (3.1 to 10.4)	108.5 (54.8 to 159.9)
United States Virgin Islands	Alcohol use	1.9 (0 to 3.3)	57 (0.5 to 98.8)	2.6 (0 to 5)	59.6 (0.5 to 103)	1.9 (0.2 to 3.3)	54.5 (5 to 97.4)	3.4 (0.3 to 6.5)	57.8 (5.3 to 104.2)
United States Virgin Islands	Diet high in red meat	0.7 (0.2 to 1)	19 (4.9 to 28.8)	0.9 (0.2 to 1.5)	19.9 (5 to 30.6)	0.7 (0.2 to 1.1)	18.7 (5.4 to 29.6)	1.2 (0.3 to 2)	19.8 (5.8 to 31.5)
United States Virgin Islands	Low physical activity	0.4 (0.1 to 0.8)	8.3 (3.3 to 16.7)	0.4 (0.2 to 0.9)	8.7 (3.5 to 17.4)	0.4 (0.2 to 0.8)	8.7 (3.2 to 17.4)	0.6 (0.2 to 1.3)	9.3 (3.4 to 18.6)
United States Virgin Islands	Smoking	0.8 (0.5 to 1.1)	20.3 (13.1 to 30.1)	1 (0.5 to 1.6)	21.3 (13.8 to 31.6)	0.7 (0.5 to 1)	16.2 (10.5 to 23.8)	1.1 (0.6 to 1.7)	17.3 (11.1 to 25.4)
United States Virgin Islands	Secondhand smoke	0.4 (0.1 to 0.7)	12 (2.8 to 21.9)	0.5 (0.1 to 1)	12.5 (3 to 22.9)	0.3 (0.1 to 0.6)	9.4 (2.2 to 17.8)	0.6 (0.1 to 1.1)	9.9 (2.3 to 18.9)
United States Virgin Islands	Metabolic risks	3.6 (1.3 to 6.9)	66.8 (10.4 to 141.6)	3.6 (0.8 to 7.6)	70.4 (11.1 to 148.1)	4.7 (1.8 to 8.7)	82.5 (17.1 to 168.8)	5.8 (1.4 to 12.2)	88.3 (19.3 to 181.6)
United States Virgin Islands	High body-mass index	1.6 (0.3 to 3.3)	21 (-11.7 to 58.5)	1.3 (-0.3 to 3.3)	22.4 (-12.3 to 62.1)	1.9 (0.3 to 3.7)	22.6 (-15.5 to 60)	1.9 (-0.5 to 4.5)	24.5 (-15.8 to 63.7)
United States Virgin Islands	High fasting plasma glucose	2.2 (0.4 to 5.1)	49 (9.6 to 114.7)	2.5 (0.5 to 6.1)	51.5 (10 to 120.3)	3.1 (0.6 to 6.9)	65.3 (13.4 to 147.8)	4.4 (0.8 to 10.4)	69.6 (14.4 to 156.6)
United States of America	All risk factors	9 (7 to 11.3)	239.8 (188.8 to 294.7)	26.7 (17.7 to 37.3)	266.5 (210.1 to 326.9)	6.3 (4.7 to 8.3)	150.2 (114 to 193)	23.3 (14.4 to 34.4)	173.4 (131.6 to 220.7)
United States of America	Behavioral risks	6.5 (5.5 to 7.4)	191.6 (163.4 to 217.8)	20.4 (14 to 28.3)	212 (180.6 to 241.9)	4 (3.4 to 4.6)	108.1 (91.6 to 124)	16 (10.4 to 22.9)	124.1 (104.5 to 143.4)

Location	Risk factor	1990				2019			
		Deaths	YLLs (Years of Life Lost)	YLDs (Years Lived with Disability)	DALYs (Disability-Adjusted Life Years)	Deaths	YLLs (Years of Life Lost)	YLDs (Years Lived with Disability)	DALYs (Disability-Adjusted Life Years)
United States of America	Alcohol use	2.7 (2.2 to 3.2)	83 (68.5 to 98.8)	8.7 (5.9 to 12.2)	91.7 (75.1 to 109.6)	1.9 (1.5 to 2.3)	54.1 (43.5 to 64.7)	7.9 (5 to 11.6)	62 (49.8 to 75.5)
United States of America	Diet high in red meat	1.4 (0.7 to 1.9)	40.4 (20.7 to 54.1)	4.3 (2.1 to 6.7)	44.7 (22.6 to 60)	0.9 (0.4 to 1.2)	23.9 (12.1 to 32.5)	3.5 (1.5 to 5.8)	27.4 (13.8 to 37.5)
United States of America	Low physical activity	0.4 (0.1 to 0.7)	8.4 (3.1 to 17.7)	1 (0.4 to 2.2)	9.4 (3.4 to 19.7)	0.2 (0.1 to 0.4)	4.8 (1.8 to 9.9)	0.7 (0.3 to 1.7)	5.6 (2.1 to 11.5)
United States of America	Smoking	2.3 (1.6 to 3)	67.1 (47.6 to 87)	7.2 (4.3 to 10.9)	74.4 (53.2 to 96.1)	1.1 (0.8 to 1.5)	29.6 (20.9 to 38.6)	4.4 (2.6 to 6.7)	34.1 (24.3 to 44.6)
United States of America	Secondhand smoke	0.4 (0.1 to 0.7)	12.6 (3 to 22.2)	1.3 (0.3 to 2.3)	13.9 (3.3 to 24.4)	0.2 (0 to 0.3)	5.5 (1.3 to 9.5)	0.8 (0.2 to 1.5)	6.2 (1.5 to 10.8)
United States of America	Metabolic risks	3.1 (1.2 to 5.8)	62.7 (13.3 to 125.4)	8.1 (2.3 to 16)	70.8 (15.9 to 139.9)	2.9 (1.1 to 5.3)	53.9 (12.8 to 105)	9.3 (2.8 to 18.4)	63.3 (15.9 to 121.6)
United States of America	High body-mass index	1.3 (0.2 to 2.8)	20 (-7.7 to 51.2)	3 (-0.1 to 6.8)	23 (-8.1 to 58.7)	1.2 (0.3 to 2.5)	17.5 (-5.2 to 41.9)	3.5 (0 to 7.9)	20.9 (-5 to 48.9)
United States of America	High fasting plasma glucose	1.9 (0.4 to 4.3)	45.7 (8.7 to 101.8)	5.4 (1 to 12.9)	51.1 (9.6 to 114.4)	1.9 (0.4 to 4)	39.8 (7.9 to 85.2)	6.4 (1.2 to 15)	46.2 (9.1 to 100.2)
Uruguay	All risk factors	10.8 (8.7 to 12.9)	288.2 (235.3 to 339.4)	13.1 (8.8 to 18.4)	301.4 (247 to 353.8)	9.2 (7.2 to 11.6)	222.1 (175.3 to 274.8)	15.2 (9.4 to 23.2)	237.3 (187 to 293.4)
Uruguay	Behavioral risks	9 (7.4 to 10.5)	255.9 (212.1 to 299.7)	11.5 (7.5 to 16)	267.4 (221.2 to 313.1)	6.8 (5.6 to 8)	180.2 (150 to 212.8)	12.2 (7.5 to 18)	192.3 (160.1 to 226.9)
Uruguay	Alcohol use	3.8 (3.1 to 4.7)	110.6 (89.3 to 134.4)	4.9 (3.2 to 7.1)	115.6 (93.3 to 140.5)	3 (2.3 to 3.8)	81.4 (63.7 to 101)	5.5 (3.3 to 8.3)	86.9 (67.9 to 108.1)
Uruguay	Diet high in red meat	2.4 (1.1 to 3.3)	66.3 (32 to 95.1)	3 (1.4 to 4.9)	69.3 (33.6 to 99.8)	1.7 (0.8 to 2.4)	44.5 (22.1 to 62.5)	3 (1.3 to 5.1)	47.5 (23.1 to 67.4)
Uruguay	Low physical activity	0.2 (0.1 to 0.5)	5.5 (2.9 to 11.4)	0.3 (0.1 to 0.6)	5.8 (3.1 to 11.9)	0.2 (0.1 to 0.5)	5.2 (2.4 to 10.6)	0.4 (0.2 to 0.8)	5.6 (2.6 to 11.3)
Uruguay	Smoking	2.4 (1.6 to 3.2)	69.1 (44.4 to 94.8)	3.1 (1.7 to 4.8)	72.1 (46.3 to 99.2)	1.8 (1.2 to 2.4)	49.3 (33.5 to 67.3)	3.3 (1.8 to 5.4)	52.7 (35.7 to 71.9)
Uruguay	Secondhand smoke	1.1 (0.3 to 1.9)	30.6 (7.4 to 52)	1.4 (0.3 to 2.6)	32 (7.8 to 54.5)	0.7 (0.2 to 1.2)	17.4 (4.1 to 30.3)	1.2 (0.3 to 2.3)	18.6 (4.4 to 32.7)
Uruguay	Metabolic risks	2.3 (0.8 to 4.3)	42.8 (8.3 to 86.5)	2.2 (0.6 to 4.3)	45 (9.1 to 90.5)	3.1 (1.2 to 5.8)	55.3 (13.6 to 111.4)	4 (1 to 8.3)	59.3 (15 to 117.9)
Uruguay	High body-mass index	1.4 (0.3 to 3)	23.6 (-3.6 to 57.6)	1.2 (0 to 2.8)	24.8 (-3.4 to 60.3)	1.6 (0.4 to 3.3)	24.6 (-3.4 to 58.4)	1.8 (-0.1 to 4.4)	26.4 (-3.4 to 63.1)
Uruguay	High fasting plasma glucose	0.9 (0.2 to 2.1)	20.3 (3.5 to 47)	1 (0.2 to 2.5)	21.3 (3.7 to 49.3)	1.6 (0.3 to 3.7)	33 (6.2 to 77)	2.4 (0.4 to 6)	35.4 (6.6 to 82.7)
Uzbekistan	All risk factors	1.9 (1.2 to 2.6)	50.8 (31.2 to 71.2)	2.1 (1.1 to 3.2)	52.9 (32.3 to 73.9)	3.3 (2 to 5)	84.5 (48.1 to 131.2)	4.1 (2.1 to 6.9)	88.6 (50.6 to 137.2)
Uzbekistan	Behavioral risks	1.2 (0.8 to 1.5)	39.4 (25.8 to 51)	1.5 (0.8 to 2.2)	40.8 (26.7 to 52.9)	1.7 (1.1 to 2.2)	52.3 (34.7 to 71.9)	2.5 (1.4 to 3.8)	54.8 (36.7 to 74.5)
Uzbekistan	Alcohol use	0.3 (0.1 to 0.4)	9.3 (5.5 to 13.7)	0.3 (0.2 to 0.6)	9.7 (5.7 to 14.1)	0.4 (0.3 to 0.6)	14.7 (9.1 to 21.4)	0.7 (0.4 to 1.1)	15.4 (9.7 to 22.4)
Uzbekistan	Diet high in red meat	0.5 (0.2 to 0.6)	16 (6 to 21.1)	0.6 (0.2 to 1)	16.6 (6.3 to 21.9)	0.7 (0.3 to 1)	21.7 (9 to 31.6)	1 (0.4 to 1.7)	22.7 (9.3 to 32.8)
Uzbekistan	Low physical activity	0.1 (0.1 to 0.2)	2.1 (1.4 to 4.2)	0.1 (0.1 to 0.2)	2.2 (1.5 to 4.3)	0.1 (0.1 to 0.2)	2.9 (1.7 to 5.5)	0.1 (0.1 to 0.3)	3 (1.8 to 5.7)
Uzbekistan	Smoking	0.1 (0 to 0.1)	2.1 (1.2 to 3.5)	0.1 (0 to 0.1)	2.2 (1.2 to 3.6)	0.1 (0.1 to 0.1)	2.7 (1.4 to 4.5)	0.1 (0.1 to 0.2)	2.8 (1.4 to 4.7)
Uzbekistan	Secondhand smoke	0.3 (0.1 to 0.6)	11.1 (2.7 to 19.4)	0.4 (0.1 to 0.8)	11.6 (2.8 to 20.3)	0.4 (0.1 to 0.7)	12.4 (3 to 21.9)	0.6 (0.1 to 1.1)	13 (3.1 to 23)
Uzbekistan	Metabolic risks	0.7 (0.2 to 1.5)	12.4 (-4 to 31.4)	0.6 (0 to 1.4)	13 (-4 to 33.1)	1.8 (0.6 to 3.5)	35.2 (2.3 to 78.9)	1.8 (0.1 to 4.1)	37 (2.6 to 83.2)
Uzbekistan	High body-mass index	0.4 (-0.1 to 0.9)	2.5 (-9.9 to 14.9)	0.2 (-0.3 to 0.8)	2.8 (-10.2 to 15.4)	0.7 (0 to 1.6)	6.9 (-13.1 to 26.7)	0.4 (-0.6 to 1.4)	7.3 (-13.6 to 28.2)
Uzbekistan	High fasting plasma glucose	0.4 (0.1 to 0.9)	10.2 (1.8 to 24.1)	0.4 (0.1 to 1.1)	10.6 (1.9 to 25.1)	1.2 (0.2 to 2.7)	30 (5.4 to 68.8)	1.5 (0.3 to 3.6)	31.5 (5.6 to 71.9)
Vanuatu	All risk factors	4.7 (2.3 to 7.6)	124.4 (62.4 to 201)	3.5 (1.7 to 6)	127.8 (64.3 to 206.4)	8.9 (4.1 to 15.1)	236.7 (113.8 to 400.4)	6.6 (3.1 to 11.4)	243.3 (117 to 410.6)
Vanuatu	Behavioral risks	1.6 (0.9 to 2.5)	47.9 (24.9 to 75.2)	1.3 (0.6 to 2.1)	49.1 (25.5 to 77.2)	2.4 (1.3 to 3.8)	71.5 (37.3 to 118.2)	1.9 (0.9 to 3.3)	73.4 (38.2 to 120.9)
Vanuatu	Alcohol use	0.1 (0 to 0.1)	2.4 (1.1 to 4.2)	0.1 (0 to 0.1)	2.4 (1.2 to 4.3)	0.1 (0 to 0.2)	3.8 (1.6 to 7.2)	0.1 (0 to 0.2)	3.9 (1.7 to 7.4)
Vanuatu	Diet high in red meat	0.6 (0.2 to 1)	19.4 (6.4 to 32.1)	0.5 (0.2 to 0.9)	19.9 (6.6 to 32.9)	0.9 (0.3 to 1.6)	29.1 (10.8 to 51)	0.8 (0.3 to 1.4)	29.9 (11.1 to 52.2)
Vanuatu	Low physical activity	0.2 (0.1 to 0.3)	3.9 (1.7 to 8.2)	0.1 (0 to 0.2)	4 (1.8 to 8.4)	0.3 (0.1 to 0.6)	6.4 (2.7 to 13)	0.2 (0.1 to 0.4)	6.6 (2.8 to 13.3)
Vanuatu	Smoking	0.2 (0.1 to 0.3)	4.9 (2.2 to 8.3)	0.1 (0.1 to 0.3)	5 (2.3 to 8.5)	0.2 (0.1 to 0.4)	5.3 (2.6 to 9.1)	0.2 (0.1 to 0.3)	5.5 (2.6 to 9.4)
Vanuatu	Secondhand smoke	0.6 (0.1 to 1.1)	19 (4.5 to 36.3)	0.5 (0.1 to 1)	19.5 (4.6 to 37.4)	0.9 (0.2 to 1.8)	29.1 (6.1 to 59.2)	0.8 (0.2 to 1.6)	29.8 (6.2 to 60.7)
Vanuatu	Metabolic risks	3.4 (1.2 to 6.3)	84.8 (32.1 to 159.1)	2.4 (0.9 to 4.7)	87.3 (33.2 to 162.9)	7.1 (2.6 to 13.3)	181.2 (67.5 to 344)	5.1 (1.9 to 9.5)	186.3 (70 to 352.9)
Vanuatu	High body-mass index	2.5 (0.8 to 5.1)	61.4 (19 to 128.6)	1.8 (0.5 to 3.8)	63.2 (19.5 to 132.5)	4.6 (1.3 to 9)	116 (34 to 226)	3.3 (0.9 to 6.5)	119.2 (34.8 to 231.5)
Vanuatu	High fasting plasma glucose	1.1 (0.2 to 2.8)	27.8 (4.6 to 70.6)	0.8 (0.1 to 2.1)	28.6 (4.7 to 72.7)	3.1 (0.6 to 7.7)	80 (16.3 to 202.8)	2.2 (0.4 to 5.7)	82.3 (16.7 to 207.6)
Venezuela (Bolivarian Republic of)	All risk factors	3.4 (2.3 to 4.6)	89.2 (63 to 119.3)	3.7 (2.3 to 5.3)	92.8 (65.2 to 124)	4.2 (2.5 to 6.7)	104 (59.9 to 169.6)	6.8 (3.7 to 11.7)	110.8 (64 to 179.1)

Location	Risk factor	1990				2019			
		Deaths	YLLs (Years of Life Lost)	YLDs (Years Lived with Disability)	DALYs (Disability-Adjusted Life Years)	Deaths	YLLs (Years of Life Lost)	YLDs (Years Lived with Disability)	DALYs (Disability-Adjusted Life Years)
Venezuela (Bolivarian Republic of)	Behavioral risks	2.2 (1.7 to 2.6)	67 (52.7 to 80.7)	2.6 (1.7 to 3.7)	69.6 (54.9 to 83.8)	2.1 (1.4 to 3)	64 (42.4 to 90.7)	4 (2.4 to 6.1)	67.9 (45 to 96.3)
Venezuela (Bolivarian Republic of)	Alcohol use	0.8 (0.6 to 1.1)	28.1 (21.9 to 35.6)	1.1 (0.7 to 1.5)	29.2 (22.7 to 37)	0.8 (0.5 to 1.1)	25.3 (16.6 to 37)	1.5 (0.9 to 2.4)	26.8 (17.6 to 39.1)
Venezuela (Bolivarian Republic of)	Diet high in red meat	0.4 (0.1 to 0.5)	12.3 (3.8 to 16.2)	0.5 (0.1 to 0.7)	12.8 (3.9 to 16.9)	0.5 (0.2 to 0.8)	16.1 (5 to 25.6)	1 (0.3 to 1.7)	17.1 (5.3 to 27.1)
Venezuela (Bolivarian Republic of)	Low physical activity	0.1 (0 to 0.2)	1.9 (1.1 to 4.2)	0.1 (0 to 0.2)	2 (1.2 to 4.3)	0.1 (0 to 0.2)	2.4 (1.2 to 5.2)	0.2 (0.1 to 0.3)	2.6 (1.3 to 5.5)
Venezuela (Bolivarian Republic of)	Smoking	0.7 (0.4 to 0.9)	17.7 (10.5 to 25.3)	0.7 (0.4 to 1.2)	18.4 (10.9 to 26.4)	0.5 (0.3 to 0.7)	12 (6.5 to 19.3)	0.8 (0.4 to 1.3)	12.7 (6.9 to 20.5)
Venezuela (Bolivarian Republic of)	Secondhand smoke	0.4 (0.1 to 0.6)	11.2 (2.6 to 19.2)	0.4 (0.1 to 0.8)	11.6 (2.7 to 20)	0.4 (0.1 to 0.7)	11.4 (2.6 to 21.7)	0.7 (0.2 to 1.4)	12.1 (2.7 to 23)
Venezuela (Bolivarian Republic of)	Metabolic risks	1.4 (0.4 to 2.7)	26.3 (3.1 to 57.7)	1.2 (0.2 to 2.7)	27.5 (3.3 to 60.1)	2.3 (0.7 to 4.7)	45.6 (6.9 to 105.5)	3.2 (0.7 to 7.4)	48.8 (7.6 to 111.5)
Venezuela (Bolivarian Republic of)	High body-mass index	0.4 (0 to 1)	4.2 (-8.2 to 16.8)	0.3 (-0.2 to 0.9)	4.5 (-8.4 to 17.7)	0.7 (0 to 1.7)	8.7 (-10.4 to 29.1)	0.8 (-0.4 to 2.3)	9.4 (-10.6 to 31.5)
Venezuela (Bolivarian Republic of)	High fasting plasma glucose	1 (0.2 to 2.3)	23.1 (4.5 to 52.5)	1 (0.2 to 2.4)	24.1 (4.7 to 54.8)	1.7 (0.3 to 4.1)	39.1 (7.7 to 96.2)	2.6 (0.5 to 6.5)	41.7 (8.2 to 102.7)
Viet Nam	All risk factors	2.5 (1.3 to 4.1)	70.3 (37.2 to 113.4)	2.1 (1 to 3.4)	72.4 (38.3 to 116.5)	5.2 (2.9 to 7.9)	136.6 (78.4 to 207.3)	6.8 (3.6 to 11.1)	143.5 (82 to 216.8)
Viet Nam	Behavioral risks	1.4 (0.7 to 2.1)	41.9 (20.9 to 65.7)	1.2 (0.5 to 2)	43.1 (21.3 to 67.5)	2.3 (1.4 to 3.4)	70.4 (42.4 to 104)	3.5 (1.9 to 5.7)	73.9 (44.4 to 108.8)
Viet Nam	Alcohol use	0 (0 to 0.1)	1.5 (0.5 to 2.7)	0 (0 to 0.1)	1.5 (0.5 to 2.8)	0.5 (0.3 to 0.7)	15.8 (10.3 to 23.4)	0.8 (0.4 to 1.3)	16.6 (10.8 to 24.5)
Viet Nam	Diet high in red meat	0.3 (0.1 to 0.5)	10.1 (1.9 to 15.7)	0.3 (0.1 to 0.5)	10.4 (2 to 16.1)	0.8 (0.3 to 1.2)	23.2 (7.8 to 36.5)	1.2 (0.4 to 2)	24.4 (8.2 to 38.3)
Viet Nam	Low physical activity	0.1 (0.1 to 0.2)	2.4 (1.4 to 5.1)	0.1 (0 to 0.2)	2.4 (1.4 to 5.2)	0.1 (0.1 to 0.3)	2.9 (1.7 to 6.6)	0.1 (0.1 to 0.3)	3.1 (1.8 to 6.9)
Viet Nam	Smoking	0.1 (0.1 to 0.2)	2.9 (1.4 to 4.8)	0.1 (0 to 0.2)	3 (1.5 to 4.9)	0.2 (0.1 to 0.3)	3.5 (1.8 to 5.7)	0.2 (0.1 to 0.3)	3.6 (1.9 to 6)
Viet Nam	Secondhand smoke	0.8 (0.2 to 1.5)	26.1 (5.7 to 48)	0.7 (0.2 to 1.4)	26.8 (5.9 to 49.4)	0.9 (0.2 to 1.7)	28 (6.2 to 51.8)	1.4 (0.3 to 2.7)	29.4 (6.5 to 54.2)
Viet Nam	Metabolic risks	1.2 (0.4 to 2.5)	30.7 (9.1 to 63.1)	0.9 (0.3 to 2)	31.7 (9.4 to 65.1)	3.2 (1.2 to 5.8)	74.5 (26.5 to 136.7)	3.7 (1.3 to 7.1)	78.2 (27.8 to 143.5)
Viet Nam	High body-mass index	0.5 (0.1 to 1.5)	14.2 (2.5 to 38.5)	0.4 (0.1 to 1.1)	14.6 (2.5 to 39.6)	1.8 (0.4 to 4)	45.1 (11.1 to 100.2)	2.2 (0.5 to 4.8)	47.3 (11.6 to 104.6)
Viet Nam	High fasting plasma glucose	0.7 (0.1 to 1.7)	17.2 (3.1 to 40.6)	0.5 (0.1 to 1.3)	17.7 (3.1 to 42)	1.5 (0.3 to 3.5)	32.5 (5.8 to 77.4)	1.7 (0.3 to 4.1)	34.2 (6.1 to 81.2)
Yemen	All risk factors	1.2 (0.6 to 2.3)	33.6 (16.6 to 64.3)	1 (0.5 to 1.8)	34.6 (17.1 to 66.3)	2.1 (1.1 to 3.5)	55.7 (28.5 to 96.8)	1.9 (1 to 3.4)	57.6 (29.5 to 99.4)
Yemen	Behavioral risks	0.8 (0.4 to 1.4)	24.5 (13 to 43)	0.7 (0.3 to 1.2)	25.2 (13.3 to 44)	1.1 (0.7 to 1.7)	34.7 (20.5 to 52.7)	1.1 (0.6 to 1.9)	35.9 (21.2 to 54.6)
Yemen	Alcohol use	0.1 (0 to 0.1)	2.1 (1.1 to 3.8)	0.1 (0 to 0.1)	2.2 (1.1 to 3.9)	0 (0 to 0.1)	1.4 (0.8 to 2.3)	0 (0 to 0.1)	1.4 (0.9 to 2.4)
Yemen	Diet high in red meat	0.1 (0 to 0.2)	3.8 (0.7 to 7.3)	0.1 (0 to 0.2)	3.9 (0.7 to 7.5)	0.2 (0 to 0.3)	5.9 (1.1 to 10.1)	0.2 (0 to 0.3)	6.1 (1.1 to 10.5)
Yemen	Low physical activity	0.1 (0 to 0.3)	3.8 (1.3 to 8.1)	0.1 (0 to 0.3)	3.9 (1.3 to 8.3)	0.2 (0.1 to 0.4)	5.7 (2.2 to 11.3)	0.2 (0.1 to 0.4)	5.9 (2.3 to 11.6)
Yemen	Smoking	0.2 (0.1 to 0.4)	6.7 (3 to 13)	0.2 (0.1 to 0.4)	6.9 (3 to 13.3)	0.3 (0.2 to 0.5)	9.7 (5.7 to 15.4)	0.3 (0.2 to 0.5)	10 (5.9 to 15.8)
Yemen	Secondhand smoke	0.3 (0.1 to 0.6)	8.9 (2 to 18.4)	0.2 (0.1 to 0.5)	9.1 (2 to 18.8)	0.4 (0.1 to 0.8)	13.1 (2.9 to 25)	0.4 (0.1 to 0.8)	13.5 (3 to 25.8)
Yemen	Metabolic risks	0.4 (0.1 to 1.2)	10 (0.5 to 28.5)	0.3 (0.1 to 0.9)	10.4 (0.5 to 29.5)	1.1 (0.2 to 2.3)	23.2 (0.4 to 55.6)	0.8 (0.1 to 2)	24.1 (0.5 to 57.2)
Yemen	High body-mass index	0.1 (0 to 0.3)	1.1 (-3.7 to 6)	0.1 (-0.1 to 0.2)	1.1 (-3.7 to 6.2)	0.2 (-0.1 to 0.6)	1.2 (-8.6 to 10.7)	0.1 (-0.3 to 0.4)	1.3 (-8.8 to 11)
Yemen	High fasting plasma glucose	0.3 (0 to 1)	9.2 (1.3 to 26.5)	0.3 (0 to 0.8)	9.4 (1.4 to 27.4)	0.9 (0.2 to 2.1)	22.6 (4.3 to 56.3)	0.8 (0.1 to 2)	23.4 (4.4 to 58.1)
Zambia	All risk factors	2.7 (1.7 to 4.1)	70.2 (43.7 to 104.1)	1.6 (0.9 to 2.6)	71.9 (44.6 to 106.1)	3.4 (2.1 to 5.1)	81.1 (49.5 to 126.5)	2.4 (1.3 to 3.9)	83.5 (50.7 to 130.1)
Zambia	Behavioral risks	1.9 (1.3 to 2.7)	55.6 (36.3 to 78.7)	1.2 (0.7 to 1.9)	56.8 (37.1 to 80.3)	2.1 (1.5 to 3)	57.9 (37.9 to 84.4)	1.6 (1 to 2.6)	59.6 (39 to 86.6)
Zambia	Alcohol use	0.9 (0.6 to 1.3)	28.1 (18.8 to 40.3)	0.6 (0.4 to 0.9)	28.7 (19.1 to 41.2)	1 (0.6 to 1.5)	28.8 (17.9 to 43)	0.8 (0.4 to 1.3)	29.6 (18.4 to 44.3)
Zambia	Diet high in red meat	0.3 (0 to 0.4)	8.1 (1.4 to 12.8)	0.2 (0 to 0.3)	8.2 (1.4 to 13.1)	0.3 (0.1 to 0.5)	8.7 (1.6 to 14.4)	0.2 (0 to 0.4)	9 (1.7 to 14.7)
Zambia	Low physical activity	0.1 (0.1 to 0.2)	3 (1.7 to 5.7)	0.1 (0 to 0.1)	3 (1.7 to 5.8)	0.1 (0.1 to 0.3)	3.3 (1.8 to 6.3)	0.1 (0 to 0.2)	3.4 (1.9 to 6.5)
Zambia	Smoking	0.3 (0.1 to 0.4)	4.4 (2.1 to 7.5)	0.1 (0.1 to 0.2)	4.5 (2.1 to 7.7)	0.3 (0.2 to 0.5)	5 (2.4 to 8.2)	0.2 (0.1 to 0.3)	5.1 (2.5 to 8.5)
Zambia	Secondhand smoke	0.4 (0.1 to 0.8)	14.1 (2.9 to 26.1)	0.3 (0.1 to 0.6)	14.4 (3 to 26.6)	0.5 (0.1 to 0.9)	14.3 (3.1 to 26.8)	0.4 (0.1 to 0.8)	14.7 (3.2 to 27.7)
Zambia	Metabolic risks	0.9 (0.2 to 1.9)	16.4 (-0.7 to 40.7)	0.5 (0.1 to 1.1)	16.8 (-0.6 to 41.8)	1.4 (0.4 to 3)	26 (2.4 to 60.5)	0.8 (0.1 to 1.9)	26.8 (2.6 to 62.2)
Zambia	High body-mass index	0.2 (0 to 0.6)	0.9 (-7.5 to 8.7)	0.1 (-0.1 to 0.3)	0.9 (-7.6 to 9)	0.5 (0 to 1.2)	6.3 (-8.4 to 21.7)	0.2 (-0.2 to 0.7)	6.5 (-8.5 to 22.4)
Zambia	High fasting plasma glucose	0.7 (0.1 to 1.6)	15.8 (2.8 to 37.3)	0.4 (0.1 to 1)	16.2 (2.8 to 38.2)	0.9 (0.2 to 2.3)	20.5 (3.6 to 51.3)	0.6 (0.1 to 1.6)	21.1 (3.7 to 53)
Zimbabwe	All risk factors	3 (1.8 to 4.6)	71.5 (43.3 to 106.4)	2.2 (1.2 to 3.5)	73.6 (44.5 to 109)	5.2 (2.7 to 8.8)	123.3 (61.5 to 212.3)	3.6 (1.7 to 6.4)	126.9 (63.2 to 218.7)
Zimbabwe	Behavioral risks	1.6 (1.1 to 2.2)	45.8 (30.3 to 61.5)	1.3 (0.8 to 2)	47.1 (31.2 to 63.4)	2.3 (1.4 to 3.4)	66.6 (39.3 to 99.8)	1.8 (1 to 2.9)	68.4 (40.2 to 102.5)

Location	Risk factor	1990				2019			
		Deaths	YLLs (Years of Life Lost)	YLDs (Years Lived with Disability)	DALYs (Disability-Adjusted Life Years)	Deaths	YLLs (Years of Life Lost)	YLDs (Years Lived with Disability)	DALYs (Disability-Adjusted Life Years)
Zimbabwe	Alcohol use	0.6 (0.4 to 0.8)	18.3 (11.7 to 25.7)	0.5 (0.3 to 0.8)	18.8 (12.1 to 26.4)	0.8 (0.4 to 1.2)	24.3 (13.7 to 38.2)	0.6 (0.3 to 1.1)	25 (14 to 39.3)
Zimbabwe	Diet high in red meat	0.3 (0 to 0.4)	7.5 (1.4 to 11.2)	0.2 (0 to 0.4)	7.7 (1.4 to 11.5)	0.4 (0.1 to 0.7)	12.7 (2.3 to 21.7)	0.3 (0.1 to 0.6)	13 (2.4 to 22.3)
Zimbabwe	Low physical activity	0.1 (0.1 to 0.2)	2.2 (1.4 to 5.1)	0.1 (0 to 0.1)	2.3 (1.4 to 5.2)	0.2 (0.1 to 0.3)	3.6 (2 to 7.6)	0.1 (0.1 to 0.2)	3.7 (2.1 to 7.8)
Zimbabwe	Smoking	0.3 (0.2 to 0.5)	6.1 (3.6 to 9.5)	0.2 (0.1 to 0.3)	6.3 (3.8 to 9.8)	0.4 (0.2 to 0.6)	7.3 (4.1 to 12)	0.2 (0.1 to 0.4)	7.5 (4.2 to 12.3)
Zimbabwe	Secondhand smoke	0.4 (0.1 to 0.8)	13.3 (3 to 23.5)	0.4 (0.1 to 0.7)	13.7 (3.1 to 24.2)	0.7 (0.1 to 1.2)	21 (4.3 to 39.5)	0.6 (0.1 to 1.1)	21.5 (4.5 to 40.5)
Zimbabwe	Metabolic risks	1.5 (0.5 to 3.1)	28.3 (3.3 to 62.7)	0.9 (0.2 to 2.1)	29.3 (3.5 to 65)	3.2 (1 to 6.6)	62 (8.6 to 145.3)	1.9 (0.4 to 4.5)	63.9 (9 to 150)
Zimbabwe	High body-mass index	0.5 (0 to 1.2)	6.6 (-7.1 to 22.9)	0.3 (-0.1 to 0.7)	6.9 (-7.1 to 23.7)	0.9 (0 to 2)	9 (-15.5 to 35.7)	0.4 (-0.3 to 1.2)	9.4 (-15.7 to 36.8)
Zimbabwe	High fasting plasma glucose	1.1 (0.2 to 2.5)	22.7 (4.1 to 53.2)	0.7 (0.1 to 1.7)	23.4 (4.2 to 54.8)	2.4 (0.5 to 5.8)	55.7 (10.4 to 135.3)	1.6 (0.3 to 4.1)	57.4 (10.7 to 139)

Supplementary table 3. The global, regional, SDI quintiles, and national age-standardized estimates of QCI by sex and rQCI for females, in 1990 and 2019.

Scale	Location	1990				2019			
		QCI		rQCI		QCI		rQCI	
		Female	Male	Female	Male	Female	Male	Female	Male
**Global**	Global	72.9	56.3	73.9	NA	78.7	69.9	82.2	NA
**WHO regions**	African Region	19.0	15.9	22.2	NA	32.1	31.8	37.8	NA
	Eastern Mediterranean Region	33.9	20.5	30.4	NA	52.1	40.4	54.7	NA
	European Region	78.6	75.9	75.7	NA	88.9	85.7	87.8	NA
	Region of the Americas	83.6	87.6	82.7	NA	88.1	87.5	87.3	NA
	South-East Asia Region	28.6	23.3	24.3	NA	48.3	48.0	48.2	NA
	Western Pacific Region	71.5	51.0	69.2	NA	85.2	72.8	86.1	NA
**SDI quintiles**	High SDI	87.2	89.9	84.0	NA	95.7	94.3	92.7	NA
	High-middle SDI	71.2	66.4	68.3	NA	82.9	78.1	83.2	NA
	Low SDI	13.9	11.0	12.0	NA	27.3	24.4	28.4	NA
	Low-middle SDI	27.4	19.5	25.8	NA	45.5	42.8	48.3	NA
	Middle SDI	48.3	33.6	45.4	NA	69.7	64.5	71.5	NA
**Countries**	Afghanistan	17.8	13.6	10.0	NA	22.9	26.9	19.6	NA
	Albania	73.8	63.8	62.0	NA	82.8	76.4	80.1	NA
	Algeria	55.0	29.7	49.9	NA	70.5	57.5	69.2	NA
	American Samoa	46.6	54.3	42.5	NA	51.0	53.8	51.0	NA
	Andorra	91.1	77.7	84.8	NA	97.1	88.7	92.9	NA
	Angola	8.1	12.7	5.5	NA	22.1	26.3	24.4	NA
	Antigua and Barbuda	61.2	42.8	60.8	NA	68.8	54.9	72.8	NA
	Argentina	55.3	47.9	54.0	NA	70.8	64.5	72.2	NA
	Armenia	56.0	74.9	52.4	NA	75.3	65.5	73.0	NA
	Australia	88.1	90.7	83.3	NA	97.3	96.2	93.5	NA
	Austria	86.0	82.4	82.1	NA	94.1	89.3	91.1	NA
	Azerbaijan	50.8	71.9	45.1	NA	62.9	60.8	61.4	NA
	Bahamas	56.7	39.7	56.5	NA	66.3	50.0	67.3	NA
	Bahrain	48.8	62.1	48.3	NA	73.2	72.3	76.4	NA
	Bangladesh	17.2	9.9	12.5	NA	46.3	38.2	45.6	NA
	Barbados	61.0	44.8	62.0	NA	70.9	56.9	74.2	NA
	Belarus	71.5	66.1	65.7	NA	83.6	75.5	82.3	NA
	Belgium	81.9	81.2	80.0	NA	94.7	90.0	91.6	NA
	Belize	55.2	36.2	52.6	NA	61.6	45.1	61.4	NA
	Benin	16.4	17.0	14.8	NA	25.3	24.3	27.0	NA
	Bermuda	67.2	52.0	71.5	NA	85.0	72.5	88.2	NA
	Bhutan	17.4	9.9	11.0	NA	48.5	39.8	49.0	NA
	Bolivia (Plurinational State of)	18.3	11.7	13.3	NA	46.5	35.6	48.2	NA
	Bosnia and Herzegovina	68.6	94.7	59.5	NA	74.8	72.6	75.5	NA
	Botswana	27.3	30.2	28.2	NA	44.6	44.4	48.8	NA
	Brazil	47.2	40.2	45.3	NA	67.8	55.9	68.8	NA
	Brunei Darussalam	73.6	46.3	64.9	NA	79.7	61.4	77.6	NA
	Bulgaria	76.2	86.6	72.8	NA	80.7	81.9	81.6	NA

Scale	Location	1990				2019			
		QCI		rQCI		QCI		rQCI	
		Female	Male	Female	Male	Female	Male	Female	Male
	Burkina Faso	10.7	17.0	13.1	NA	22.8	24.4	26.3	NA
	Burundi	4.9	3.2	4.7	NA	20.0	16.5	20.1	NA
	Cabo Verde	32.4	36.6	31.7	NA	52.5	48.0	57.2	NA
	Cambodia	24.3	11.1	15.7	NA	44.1	32.8	41.9	NA
	Cameroon	14.4	19.0	14.5	NA	27.9	27.8	30.9	NA
	Canada	88.0	93.3	87.3	NA	95.8	93.4	94.3	NA
	Central African Republic	3.3	8.0	2.8	NA	5.3	13.1	5.2	NA
	Chad	14.5	14.6	12.2	NA	17.5	18.0	18.8	NA
	Chile	63.4	56.7	59.6	NA	80.5	76.2	81.0	NA
	China	61.1	54.8	56.0	NA	84.5	73.4	85.8	NA
	Colombia	52.5	36.3	52.5	NA	76.1	61.8	77.5	NA
	Comoros	10.8	9.6	10.4	NA	24.6	21.9	26.9	NA
	Congo	7.2	13.6	8.9	NA	22.9	28.4	27.1	NA
	Cook Islands	53.8	52.7	54.4	NA	67.8	67.3	70.6	NA
	Costa Rica	67.5	48.5	68.7	NA	78.3	64.1	81.6	NA
	Côte d'Ivoire	17.4	19.2	17.9	NA	24.5	25.4	27.7	NA
	Croatia	74.5	77.6	74.3	NA	84.5	85.1	85.8	NA
	Cuba	68.2	48.6	69.3	NA	78.3	65.4	81.6	NA
	Cyprus	85.0	66.4	78.8	NA	94.1	93.4	93.6	NA
	Czechia	73.5	75.7	72.9	NA	88.0	87.5	87.3	NA
	Democratic People's Republic of Korea	57.1	42.2	50.6	NA	58.9	46.7	55.4	NA
	Democratic Republic of the Congo	9.5	16.6	9.2	NA	17.3	24.7	19.5	NA
	Denmark	74.9	79.4	73.1	NA	91.7	93.0	90.0	NA
	Djibouti	16.1	14.5	16.7	NA	28.1	25.8	32.6	NA
	Dominica	55.5	41.1	55.8	NA	58.3	44.6	60.4	NA
	Dominican Republic	48.7	29.5	44.2	NA	61.3	44.3	61.3	NA
	Ecuador	37.3	25.4	33.8	NA	62.8	53.3	64.4	NA
	Egypt	51.3	56.7	41.3	NA	62.5	64.0	60.0	NA
	El Salvador	47.9	29.1	42.2	NA	68.9	51.8	70.2	NA
	Equatorial Guinea	5.3	8.0	3.1	NA	32.1	35.4	39.0	NA
	Eritrea	3.5	1.6	1.8	NA	16.6	16.5	17.9	NA
	Estonia	72.0	63.2	68.7	NA	86.4	77.3	86.5	NA
	Eswatini	22.1	23.9	25.1	NA	28.3	27.9	33.3	NA
	Ethiopia	2.1	3.6	1.2	NA	26.0	23.7	28.5	NA
	Fiji	36.0	40.0	31.0	NA	41.0	44.6	40.7	NA
	Finland	89.8	78.9	83.9	NA	98.8	92.6	95.2	NA
	France	84.5	79.2	79.6	NA	96.4	91.4	92.2	NA
	Gabon	17.8	21.7	19.1	NA	34.2	35.1	38.3	NA
	Gambia	26.3	20.6	24.3	NA	28.2	26.6	29.9	NA
	Georgia	61.2	69.1	58.9	NA	70.1	59.9	66.7	NA
	Germany	83.1	81.4	78.1	NA	92.9	92.0	90.8	NA

Scale	Location	1990				2019			
		QCI		rQCI		QCI		rQCI	
		Female	Male	Female	Male	Female	Male	Female	Male
	Ghana	16.7	21.8	16.9	NA	33.4	31.0	36.1	NA
	Greece	89.9	87.7	83.5	NA	93.0	87.7	90.8	NA
	Greenland	62.6	25.9	56.8	NA	75.6	65.3	72.4	NA
	Grenada	50.0	36.2	49.7	NA	62.0	54.9	64.9	NA
	Guam	59.5	62.1	58.7	NA	65.9	64.6	64.3	NA
	Guatemala	34.6	27.5	27.9	NA	55.5	50.6	56.0	NA
	Guinea	9.8	14.4	10.0	NA	19.4	20.1	20.9	NA
	Guinea-Bissau	8.7	10.8	7.1	NA	19.0	19.5	19.3	NA
	Guyana	41.0	27.3	38.5	NA	51.5	38.4	50.8	NA
	Haiti	15.1	10.1	11.1	NA	28.7	20.5	26.2	NA
	Honduras	39.1	22.4	33.9	NA	53.3	35.9	56.2	NA
	Hungary	68.1	68.8	67.1	NA	83.1	83.0	82.7	NA
	Iceland	89.0	79.3	85.8	NA	100.0	93.7	93.8	NA
	India	25.6	21.7	21.3	NA	43.4	49.4	43.1	NA
	Indonesia	32.7	33.5	22.7	NA	50.8	41.1	45.0	NA
	Iran (Islamic Republic of)	60.4	56.3	54.2	NA	75.1	74.5	74.1	NA
	Iraq	44.6	40.1	39.6	NA	64.8	58.3	62.2	NA
	Ireland	81.6	80.8	79.2	NA	95.8	92.9	93.3	NA
	Israel	78.3	74.1	74.3	NA	91.6	85.5	89.0	NA
	Italy	89.1	99.7	84.6	NA	98.6	93.6	93.9	NA
	Jamaica	60.6	48.0	60.0	NA	67.4	54.0	68.3	NA
	Japan	98.9	85.1	88.5	NA	99.8	91.7	95.2	NA
	Jordan	49.9	41.6	45.6	NA	72.6	66.7	73.1	NA
	Kazakhstan	56.5	63.1	51.6	NA	70.5	67.0	69.6	NA
	Kenya	23.7	18.5	24.0	NA	27.0	25.2	29.4	NA
	Kiribati	19.7	22.6	10.5	NA	23.8	26.8	19.9	NA
	Kuwait	68.9	61.9	67.5	NA	84.5	76.1	83.5	NA
	Kyrgyzstan	50.0	71.5	44.9	NA	65.4	60.7	62.9	NA
	Lao People's Democratic Republic	11.2	6.4	4.2	NA	34.6	26.1	31.6	NA
	Latvia	67.9	63.5	63.8	NA	79.4	75.0	79.3	NA
	Lebanon	54.6	43.2	51.8	NA	78.6	70.2	81.1	NA
	Lesotho	21.5	22.4	22.5	NA	17.8	21.5	22.4	NA
	Liberia	11.9	15.5	11.2	NA	25.1	25.6	27.3	NA
	Libya	56.5	44.0	50.1	NA	65.4	59.2	64.5	NA
	Lithuania	73.5	68.2	68.8	NA	81.3	75.0	79.0	NA
	Luxembourg	81.9	79.4	79.5	NA	95.7	93.5	91.9	NA
	Madagascar	12.5	12.9	9.4	NA	21.1	20.7	21.2	NA
	Malawi	13.2	12.4	14.8	NA	21.0	21.8	25.4	NA
	Malaysia	41.4	31.9	38.5	NA	64.7	53.3	66.1	NA
	Maldives	34.0	27.6	30.7	NA	70.7	64.0	72.5	NA
	Mali	11.3	17.6	9.0	NA	26.2	25.6	25.8	NA

Scale	Location	1990				2019			
		QCI		rQCI		QCI		rQCI	
		Female	Male	Female	Male	Female	Male	Female	Male
	Malta	77.6	80.5	75.1	NA	92.2	94.5	89.2	NA
	Marshall Islands	31.8	26.6	23.1	NA	32.9	31.6	29.6	NA
	Mauritania	13.8	17.3	13.1	NA	34.8	33.7	37.6	NA
	Mauritius	57.5	46.7	53.0	NA	67.1	56.7	66.6	NA
	Mexico	53.3	46.2	52.0	NA	71.5	59.9	72.8	NA
	Micronesia (Federated States of)	28.9	31.1	21.2	NA	39.8	39.5	38.9	NA
	Monaco	88.2	81.3	85.9	NA	92.1	86.5	92.6	NA
	Mongolia	48.1	50.5	38.1	NA	55.0	56.0	54.1	NA
	Montenegro	74.8	75.0	73.0	NA	81.4	78.1	81.4	NA
	Morocco	36.7	32.9	30.1	NA	58.3	49.0	55.8	NA
	Mozambique	5.9	60.0	6.7	NA	16.0	49.9	20.5	NA
	Myanmar	13.3	10.7	7.4	NA	46.6	31.7	43.2	NA
	Namibia	19.5	17.0	18.2	NA	38.9	32.3	41.3	NA
	Nauru	45.5	42.9	40.5	NA	53.4	47.7	52.4	NA
	Nepal	13.7	7.1	7.8	NA	38.1	30.0	37.3	NA
	Netherlands	85.8	86.3	83.8	NA	95.6	91.9	93.5	NA
	New Zealand	86.8	31.7	84.5	NA	97.4	38.0	94.0	NA
	Nicaragua	53.3	30.8	46.7	NA	69.0	54.4	71.9	NA
	Niger	12.7	13.0	10.5	NA	22.0	21.6	23.4	NA
	Nigeria	18.6	33.9	18.9	NA	31.4	38.6	34.8	NA
	Niue	51.9	48.2	47.7	NA	63.0	57.8	65.2	NA
	North Macedonia	65.8	66.5	59.0	NA	76.5	77.8	76.5	NA
	Northern Mariana Islands	61.0	77.6	59.1	NA	72.1	74.0	70.9	NA
	Norway	84.0	79.8	78.5	NA	98.1	96.1	93.2	NA
	Oman	63.4	41.7	55.6	NA	77.1	66.7	78.1	NA
	Pakistan	16.4	14.7	12.4	NA	31.2	27.4	30.5	NA
	Palau	53.4	55.6	51.9	NA	64.2	62.0	65.7	NA
	Palestine	50.9	50.8	47.7	NA	62.5	60.0	64.4	NA
	Panama	59.9	39.8	59.2	NA	74.4	59.8	76.6	NA
	Papua New Guinea	19.1	23.4	11.4	NA	28.5	26.7	22.9	NA
	Paraguay	48.2	33.6	45.4	NA	64.1	50.8	66.0	NA
	Peru	37.6	25.5	33.0	NA	67.3	54.5	67.7	NA
	Philippines	36.4	37.3	35.0	NA	48.8	43.7	47.4	NA
	Poland	61.0	69.1	56.9	NA	78.8	80.0	79.4	NA
	Portugal	82.4	68.5	76.2	NA	96.6	88.7	91.9	NA
	Puerto Rico	71.3	53.8	71.1	NA	83.4	70.1	84.9	NA
	Qatar	54.2	52.0	52.4	NA	74.8	75.9	81.3	NA
	Republic of Korea	93.4	63.3	73.7	NA	95.7	85.2	93.4	NA
	Republic of Moldova	61.6	61.9	56.1	NA	74.5	66.4	72.3	NA
	Romania	64.2	66.3	57.3	NA	75.8	76.0	75.4	NA
	Russian Federation	73.1	63.2	66.4	NA	81.1	78.1	80.2	NA

Scale	Location	1990				2019			
		QCI		rQCI		QCI		rQCI	
		Female	Male	Female	Male	Female	Male	Female	Male
	Rwanda	1.8	3.3	3.1	NA	26.8	23.9	30.3	NA
	Saint Kitts and Nevis	56.7	38.8	59.5	NA	69.5	54.0	75.7	NA
	Saint Lucia	51.0	33.5	50.8	NA	65.9	50.6	67.3	NA
	Saint Vincent and the Grenadines	52.2	36.0	52.7	NA	58.9	43.4	60.7	NA
	Samoa	44.3	40.4	37.0	NA	52.4	47.7	49.1	NA
	San Marino	90.8	88.5	85.8	NA	93.3	93.0	91.4	NA
	Sao Tome and Principe	24.2	26.4	22.9	NA	37.1	37.4	40.2	NA
	Saudi Arabia	51.7	45.9	38.0	NA	76.2	72.1	75.5	NA
	Senegal	14.9	17.7	14.4	NA	26.6	25.8	27.8	NA
	Serbia	65.1	64.6	61.6	NA	77.1	75.3	79.2	NA
	Seychelles	48.3	41.4	42.3	NA	62.8	54.5	62.4	NA
	Sierra Leone	15.8	16.2	15.1	NA	23.2	24.0	26.0	NA
	Singapore	86.1	69.8	78.8	NA	96.2	90.2	93.0	NA
	Slovakia	68.9	69.6	65.2	NA	81.1	81.1	83.1	NA
	Slovenia	73.9	71.8	72.8	NA	88.2	86.2	87.3	NA
	Solomon Islands	37.6	31.5	26.0	NA	34.9	36.6	30.5	NA
	Somalia	6.1	6.7	NA	NA	8.2	12.7	NA*	NA
	South Africa	33.3	37.4	32.2	NA	43.8	43.8	47.5	NA
	South Sudan	10.7	9.7	10.3	NA	16.0	16.4	17.9	NA
	Spain	85.7	91.7	78.8	NA	96.8	95.3	92.5	NA
	Sri Lanka	52.4	38.7	47.0	NA	69.3	61.5	70.4	NA
	Sudan	36.9	25.2	26.4	NA	50.8	44.7	47.7	NA
	Suriname	46.4	29.7	44.4	NA	54.7	40.3	55.6	NA
	Sweden	90.8	91.3	86.2	NA	97.1	96.7	94.0	NA
	Switzerland	83.1	83.3	81.2	NA	97.0	98.0	93.0	NA
	Syrian Arab Republic	61.8	58.4	49.8	NA	69.6	69.7	68.4	NA
	Taiwan (Province of China)	75.0	55.0	67.9	NA	89.1	75.5	86.3	NA
	Tajikistan	48.3	58.2	39.8	NA	51.1	51.1	48.7	NA
	Thailand	50.6	34.3	44.2	NA	75.6	64.6	72.3	NA
	Timor-Leste	20.5	13.0	14.1	NA	35.9	28.9	35.0	NA
	Togo	18.3	21.3	18.9	NA	27.4	27.0	30.3	NA
	Tokelau	37.5	37.2	31.3	NA	53.3	49.7	53.4	NA
	Tonga	39.4	50.2	34.5	NA	49.7	54.0	47.8	NA
	Trinidad and Tobago	51.0	42.3	51.8	NA	64.6	49.3	66.0	NA
	Tunisia	58.3	47.7	53.5	NA	76.1	68.4	75.7	NA
	Turkey	48.8	39.2	41.3	NA	75.4	68.9	75.8	NA
	Turkmenistan	51.1	66.5	44.0	NA	63.9	57.1	45.0	NA
	Tuvalu	30.5	30.8	25.0	NA	43.0	40.8	42.9	NA
	Uganda	13.7	9.6	15.0	NA	27.7	23.7	28.3	NA
	Ukraine	59.0	80.4	51.7	NA	69.5	68.0	64.6	NA
	United Arab Emirates	50.6	42.2	45.6	NA	65.6	59.9	62.9	NA

Scale	Location	1990				2019			
		QCI		rQCI		QCI		rQCI	
		Female	Male	Female	Male	Female	Male	Female	Male
	United Kingdom	80.6	81.4	78.7	NA	93.6	90.9	91.8	NA
	United Republic of Tanzania	16.4	13.7	20.0	NA	25.4	24.8	30.3	NA
	United States of America	89.6	97.2	88.3	NA	97.0	98.6	94.0	NA
	United States Virgin Islands	59.1	40.6	61.1	NA	69.9	52.4	73.4	NA
	Uruguay	59.0	55.2	59.1	NA	75.4	66.3	75.7	NA
	Uzbekistan	60.0	84.4	50.6	NA	64.2	63.2	62.0	NA
	Vanuatu	32.5	28.5	25.2	NA	27.6	29.4	25.4	NA
	Venezuela (Bolivarian Republic of)	53.1	32.6	52.0	NA	71.4	54.5	73.3	NA
	Viet Nam	36.7	19.5	32.2	NA	63.4	49.4	63.7	NA
	Yemen	37.6	25.5	27.8	NA	44.0	41.7	40.3	NA
	Zambia	8.3	8.8	12.1	NA	27.5	23.8	31.3	NA
	Zimbabwe	29.9	35.4	32.2	NA	25.3	30.3	27.5	NA

* NA: not applicable due to lack of data for rQCI calculation in males and country Somalia.
